# Photochemical Ring Opening of Cyclopropyl Silyl Ethers Enables β–β Coupling, Radical‐Polar Crossover and Annulation

**DOI:** 10.1002/anie.7669030

**Published:** 2026-07-23

**Authors:** Jacop Rydén, Eilidh Young, Wilma Olsson, Eskil Flodén, Mattias Tan, Sebastian Clementson

**Affiliations:** ^1^ Centre For Analysis and Synthesis Department of Chemistry Lund University Lund Sweden

**Keywords:** Giese reaction, photoredox catalysis, radical–polar crossover, total synthesis

## Abstract

A photochemical radical ring opening strategy is reported in which cyclopropyl silyl ethers undergo single‐electron oxidation and fragmentation under visible‐light photoredox conditions to furnish β‐carbonyl radicals. These intermediates engage electron‐deficient alkenes in a controlled intermolecular addition process that combines radical ring opening with C–C bond formation. The reaction delivers β–β coupling products across a broad substrate scope, including simple and complex alkenes and substrates that undergo regioselective 1,6‐addition. The resulting adducts can be further advanced through downstream annulation chemistry, whereas under modified conditions, the same intermediates can undergo radical–polar crossover and subsequent aldol cyclization, enabling access to three separate product classes from a common cyclopropyl precursor. The synthetic utility of the method is demonstrated through divergent entry into the carbocyclic frameworks of the kaurene and phyllocladane natural product families and the first total synthesis of (−)‐*ent*‐melicodenone C.

## Graphical abstract

A photochemical platform enables β–β coupling of cyclopropyl silyl ethers with electron‐deficient alkenes. The resulting adducts serve as entry points into annulative and radical–polar crossover pathways, providing rapid access to fused, bridged, and spirocyclic architectures, including late‐stage scaffold diversification to diterpene‐like carbocyclic frameworks.

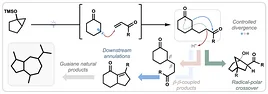

Access to β‐substituted carbonyl compounds remains a central challenge in radical chemistry, particularly in contexts requiring rapid access to complex molecular scaffolds. Visible‐light photoredox catalysis has enabled new approaches to β‐functionalization through controlled single‐electron pathways [1, 2, 3, 4]. Yet productive interception of β‐radicals with electron‐deficient alkenes remains problematic. The partners that enable high‐value Giese additions (e.g., acrylates, vinyl ketones, maleimides) are prone to oligomerization, often resulting in poor mass balance and limited synthetic utility [5, 6]. In many photoredox systems, persistent radical concentrations, mismatched rates of radical capture versus propagation, and the absence of built‐in termination pathways further aggravate this problem [7, 8, 9]. Here, we combine β‐carbonyl radical generation from cyclopropyl silyl ethers with immediate Giese addition in a tandem process that enables β–β coupling across a broad set of acceptors while furnishing adducts amenable to downstream diversification.

Radical ring‐opening strategies have provided powerful solutions for forging complex carbon frameworks, most notably exemplified by the Dowd–Beckwith ring expansion [10, 11] and its modern descendants [12, 13]. Shenvi and co‐workers demonstrated the continued synthetic relevance of controlled radical ring openings in their concise syntheses of GB22, GB13, and himgaline, where strategic C–C bond cleavage enabled rapid access to highly reduced polycyclic architectures (Figure 1a) [14]. Despite these advances, the interception of such ring‐opened intermediates in a controlled bond‐forming event remains comparatively underdeveloped.

**FIGURE 1:**
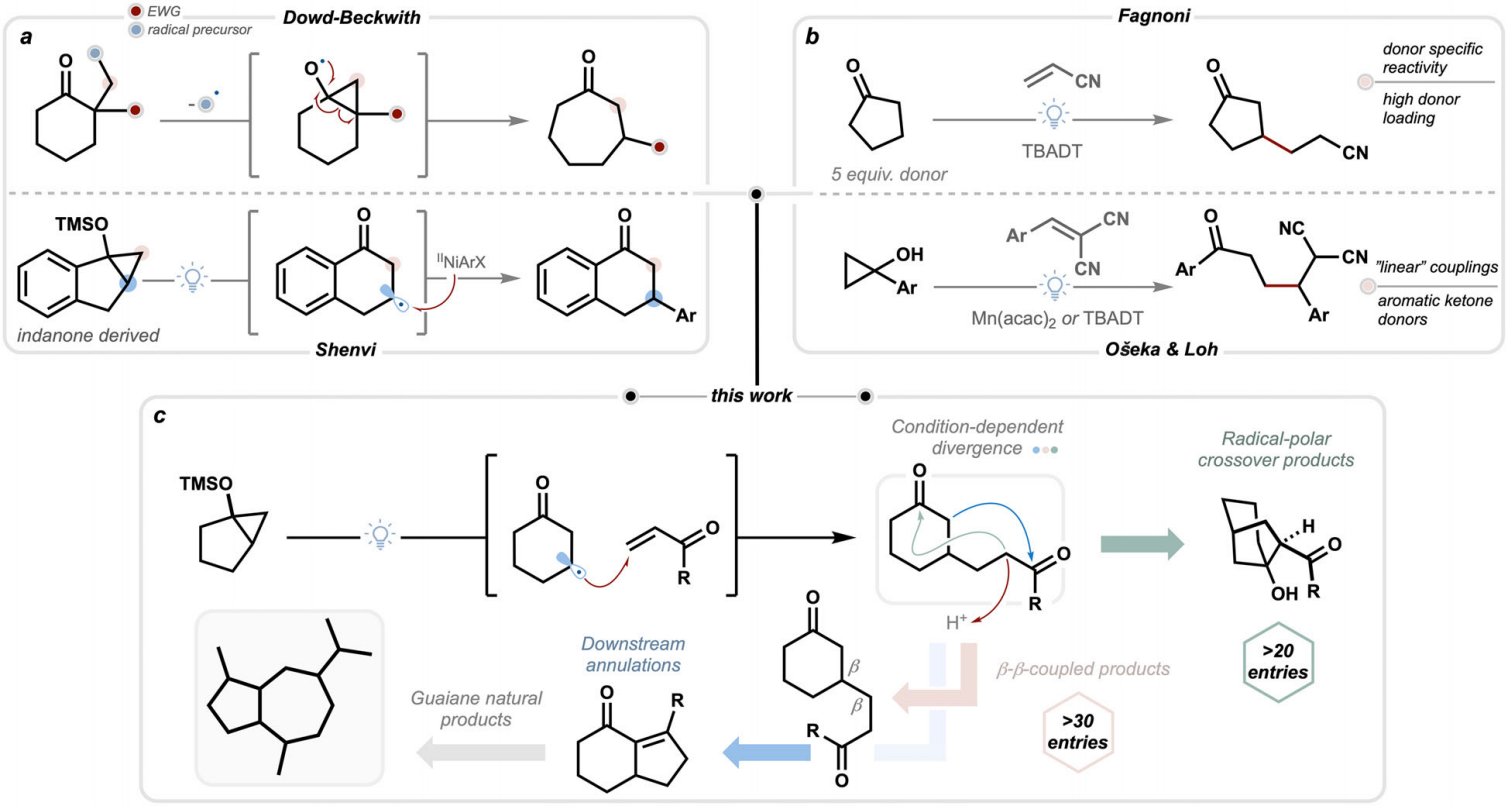
(a) Radical ring expansions and openings. (b) Intermolecular β−β couplings. (c) This work.

Other studies have extended radical logic toward direct β‐ and γ‐carbonyl C–C bond formation; Ti(III)‐catalyzed reductive umpolung has enabled the formation of β‐substituted products via conjugate alkylation of α, β‐unsaturated carbonyls [15]. Recently, Juliá and co‐workers reported a ferrioxalate‐based photocatalytic platform that enables reductive iron‐mediated transformations, including the cross‐coupling of electron‐deficient alkenes to form β‐substituted carbonyl products [16]. Complementary strategies based on strained‐ring C–C bond cleavage [17] or allylic C–H activation have also provided access to challenging β‐ and γ‐substituted carbonyl motifs [18]. Fagnoni and co‐workers demonstrated β‐selective alkylation of cyclopentanones via photoexcited decatungstate catalysis, exploiting hydrogen abstraction and radical addition to electron‐deficient alkenes [19]. Ošeka and Loh independently harnessed the ring‐opening of cyclopropanols to access β‐ketoalkyl radicals, which undergo Giese addition to activated alkenes [20, 21] (Figure 1b), while Li and co‐workers reported a three‐component alkene difunctionalization of cyclopropanols to furnish 5‐oxo peroxides [22]. Collectively, these advances underscore the growing ability to access β‐radical intermediates and forge C–C bonds under mild conditions. However, these strategies typically terminate in a single bond‐forming event and have not been developed into condition‐divergent platforms that channel a common intermediate into different product classes.

Herein, we report a platform in which radical ring opening and intermolecular Giese addition are merged within a single catalytic manifold, enabling β–β bond formation that serves not as a terminus, but as an entry point into scaffold diversification through annulative, Michael, and radical–polar crossover (RPC) pathways (Figure 1c).

Based on pioneering studies by Mattay [6, 23] and Booker–Milburn [24], we envision that the cyclopropyl substrate undergoes single‐electron oxidation by the excited photocatalyst. The resulting radical cation fragments to generate a β‐carbonyl radical, which can engage in a Giese‐type addition to an electron‐deficient alkene and is subsequently quenched by reductive turnover of the photocatalyst (Figure 2a). Model cyclopropyl silyl ether **1** was selected for optimization studies using methyl vinyl ketone (MVK) as the electrophilic alkene coupling partner (Figure 2b). Under optimal conditions employing 10 mol% Mes‐Acr‐MeBF_4_ under blue LED (450 nm) irradiation in MeCN/hexafluoroisopropanol (HFIP) (7:3, 50 mM), the β–β‐coupling product **2** was isolated in 93% yield (entry 1). Initial studies employed water as a co‐solvent, affording β–β‐coupled product **2** in moderate yield (60%, entry 4). Replacing water with HFIP proved beneficial, presumably due to Brønsted acid activation of the electron‐deficient alkene [25] and delivered improved yields (entry 1). Dilution from 0.15 M to 50 mM resulted in a substantial increase in yield (entry 2). Employing 4CzIPN resulted in only trace formation of **2** (entry 3), although formation of the corresponding cyclohexanone from cyclopropyl silyl ether **1** was observed. Reducing the loading of Mes‐Acr‐MeBF_4_ to 5 mol% led to only a modest decrease in yield (78%, entry 5), while decreasing the equivalents of cyclopropyl silyl ether **1** from 1.5 to 1.2 still afforded **2** in synthetically useful yield (74%, entry 6). In the absence of light (entry 7), or in the presence of radical scavenger TEMPO (entry 9), no reactivity was observed in accordance with the proposed radical reaction pathway. To further interrogate the behavior of the putative radical intermediate, cyclopropyl silyl ether **3** was subjected to the standard reaction conditions. Substituted cyclohexanone **5** was obtained as the major product in 35% yield (Figure 2c). This outcome is consistent with a pathway in which a primary radical intermediate (**4**) engages in intramolecular addition to the unactivated alkene prior to intermolecular coupling with the MVK electrophile, supporting the involvement of a radical pathway.

**FIGURE 2:**
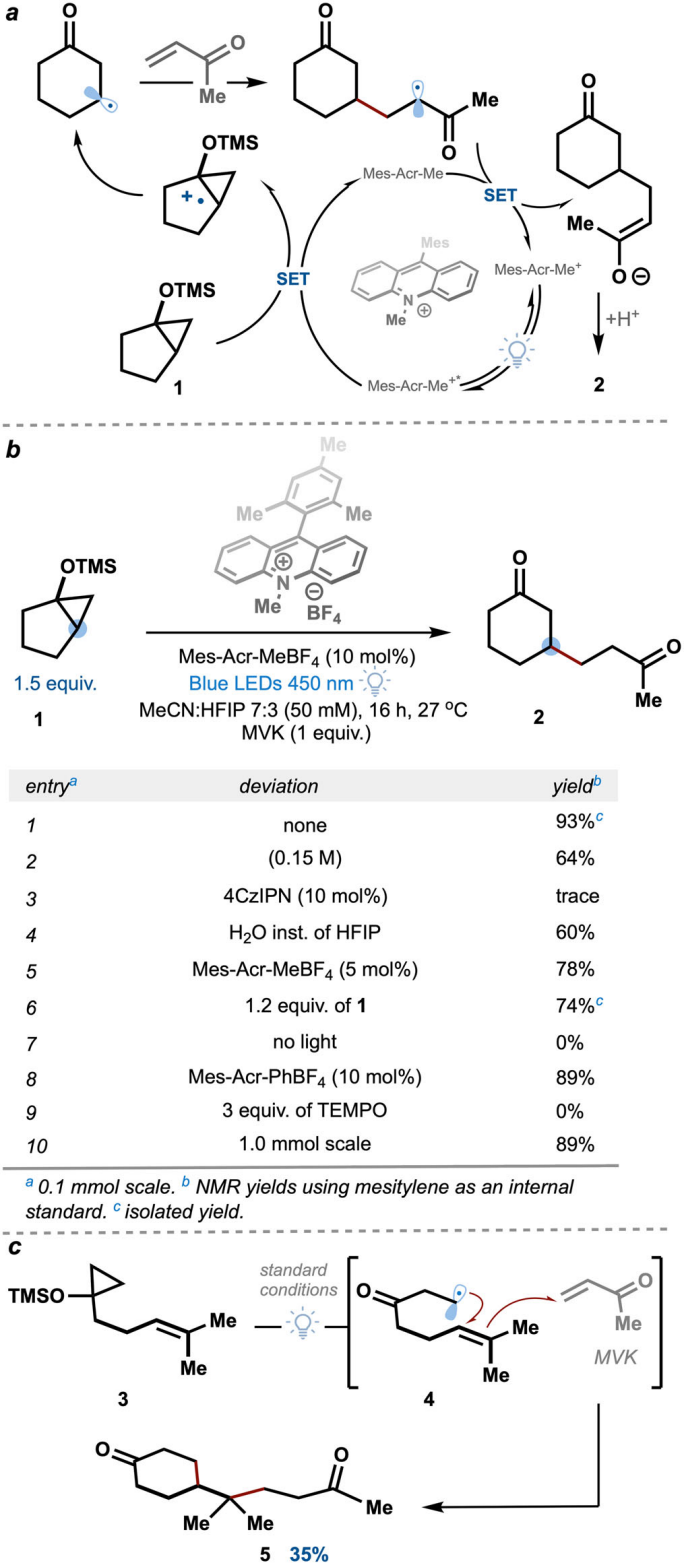
(a) Envisioned catalytic cycle. (b) Reaction optimization. (c) Tandem reaction.

The cyclopropyl silyl ethers are readily prepared in two steps and a single purification from the corresponding ketones (see Supporting Information for details). Under the optimized conditions, the β–β coupling proceeded with a broad range of electron‐deficient alkenes (Table 1a), typically using 1.5 equivalents of the cyclopropyl silyl ether, although lower and higher loadings were employed in selected cases depending on substrate class. Alkenes bearing ketone (**2**), ester (**6**), nitrile (**11**), aldehyde (**10**), sulfone (**17**), and amide (**18**) functionalities were well tolerated. Cyclic enones likewise furnished products **7–9**. Substituted acrylates, including methyl isopropenyl ketone and dimethyl maleate, were also competent partners (**15–16**). Employing (*R*)‐carvone as the electrophile afforded the β–β‐coupled product **12**, with full site‐selectivity for the internal electron‐poor alkene. The β–β coupling also enabled the formation of quaternary carbon centers; reaction between **1** and (*R*)‐pulegone afforded **13** in 35% yield as a mixture of diastereomers, while using a more substituted cyclopropyl silyl ether (**SI3)** was necessary to obtain **14**. Both lactone (**19**) and lactam (**20**) derived alkenes were also amenable to the β–β‐coupling, as were highly activated acceptors such as benzylidene malonitrile (**21**).

**TABLE 1:** Reaction scope. Yields are isolated. Yields in parentheses correspond to quantitative NMR. a) Alkene scope. b) Cyclopropyl silyl ether (ketone) scope. c) Comparison to previous methods. *^a^* 1.2 equiv. of cyclopropyl silyl ether. *^b^* 2.0 equiv. of cyclopropyl silyl ether. *^c^* After the addition of DBU.

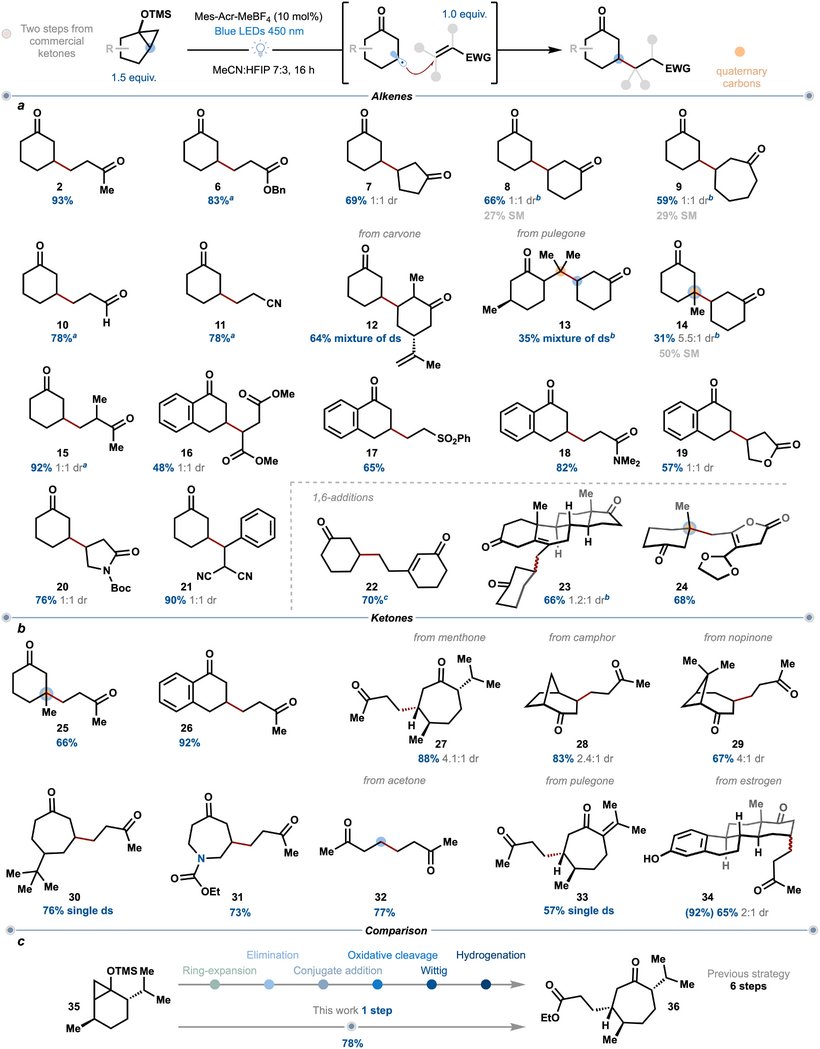

Notably, regioselective 1,6‐addition was achieved with conjugated enones, as exemplified by δ‐selective coupling of 3‐vinyl‐2‐cyclohexenone to furnish **22**. Furthermore, the 1,6‐addition approach could be extended to afford steroid‐derived **23** in 66% yield. Employing a γ,δ‐unsaturated butenolide electrophile also afforded the 1,6‐addition product **24** in 68% yield. Across all substrates examined, no significant alkene polymerization was observed, underscoring the controlled radical character of the transformation.

The scope of cyclopropyl silyl ethers was investigated, using MVK as the alkene coupling partner (Table 1b). β‐Substitution of the incipient ketone radical was well tolerated, enabling formation of quaternary centers (**25**). Benzannulated β‐β‐coupled product **26** was generated in excellent yield (92%). Bulky substituents were also accommodated, including tert‐butyl–substituted substrate **30**, and carbamate‐containing system (**31**). Product **30** was obtained as a single detectable diastereomer from the sterically hindered *tert*‐butyl‐substituted substrate, reflecting pronounced steric control in the β–β coupling, although the relative configuration could not be unambiguously assigned. Acetone‐derived cyclopropyl silyl ether effectively formed linear 1,6‐diketone **32**. Finally, natural product‐derived cyclopropyl silyl ethers proved competent substrates, affording β–β‐coupled products in good yields and with pronounced diastereoselectivity in all cases examined (**27**‐**29**, **33**, **34**, and **36**).

The utility of this methodology is highlighted by the rapid construction of cycloheptanone intermediate **36** in a single step (Table 1c). Cycloheptanone **36** was previously accessed in six steps from the same cyclopropyl silyl ether **35** by Chen and co‐workers, during their studies toward *ent*‐nanolobatolide [26]. This comparison highlights how β–β coupling can replace multistep sequences with a single bond‐forming event, delivering terpenoid‐relevant building blocks. Beyond providing access to versatile 1,6‐diketone intermediates, our β–β‐coupling method offers a convenient and mild alternative to the Helquist annulation [27] for the construction of fused bicyclic scaffolds (Scheme 1a). Exposure of β–β‐coupled product **2** to basic conditions triggered intramolecular aldol condensation, delivering 6/5‐fused bicycle **37** in quantitative yield. This annulation proceeds without preformed organometallic reagents, highlighting the intrinsic reactivity embedded in the β–β adducts. Menthone‐derived β–β‐coupled product **27** also underwent aldol condensation, affording **38** in 75% yield.

**SCHEME 1:**
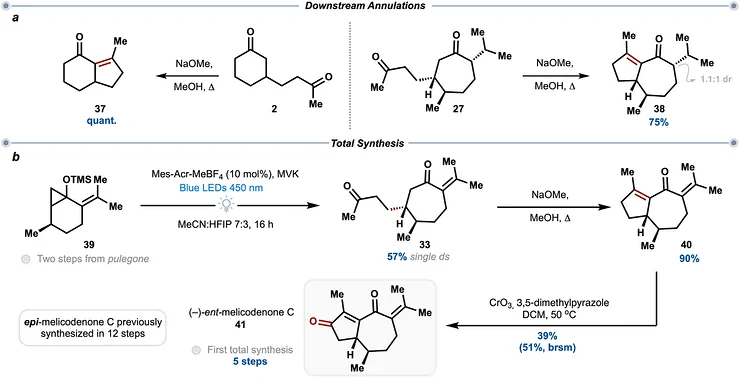
(a) Approach to annulated 6/5 and 7/5 ring systems. (b) First total synthesis of (−)‐*ent*‐melicodenone C (**41**).

The synthetic potential of this annulative manifold is exemplified by the concise total synthesis of (−)‐*ent*‐melicodenone C [28] (**41**) from pulegone‐derived cyclopropyl silyl ether **39** (Scheme 1b). Photochemical ring opening and β–β‐coupling of **39** with MVK afforded the cycloheptanone product **33** as a single diastereomer (57%). Subsequent intramolecular aldol condensation afforded 5/7‐fused bicycle **40** in 90% yield. Final allylic oxidation afforded (−)‐*ent*‐melicodenone C (**41**) in 39% isolated yield, or 51% based on recovered starting material (BRSM). Overall, the synthesis was achieved in 5 steps from (*R*)‐pulegone; by comparison, access to the related *epi*‐melicodenone C framework previously required 12 steps [29].

Next, we investigated the fate of the β‐radical intermediate under modified reaction conditions. A deuterium‐labeling experiment with model cyclopropyl silyl ether **1** in a 7:3 MeCN/D_2_O (Scheme 2a) revealed a pronounced change in reaction outcome. Whereas protic conditions favor formation of the linear β–β‐coupled product, the use of D_2_O resulted in only minor formation of **43**, with bridged bicycle **44** obtained as the major product (73% yield, single diastereomer). This outcome is consistent with a solvent‐dependent radical–polar crossover (RPC) pathway [30, 31, 32, 33, 34, 35] wherein the intermediate radical is reduced by the photocatalyst to generate enolate **42**. Under protic conditions, this species is rapidly quenched to furnish the linear product **2**; however, in the presence of the less‐acidic D_2_O [36], the increased lifetime of enolate **42** enables intramolecular aldol reaction, facilitating the formation of bridged bicycle **44**. In contrast, omission any protic co‐solvent strongly suppressed the formation of **44** (see Supporting Information for optimization studies). These observations reveal a condition‐dependent divergence of the β‐β manifold and provide access to a bridged product class.

**SCHEME 2:**
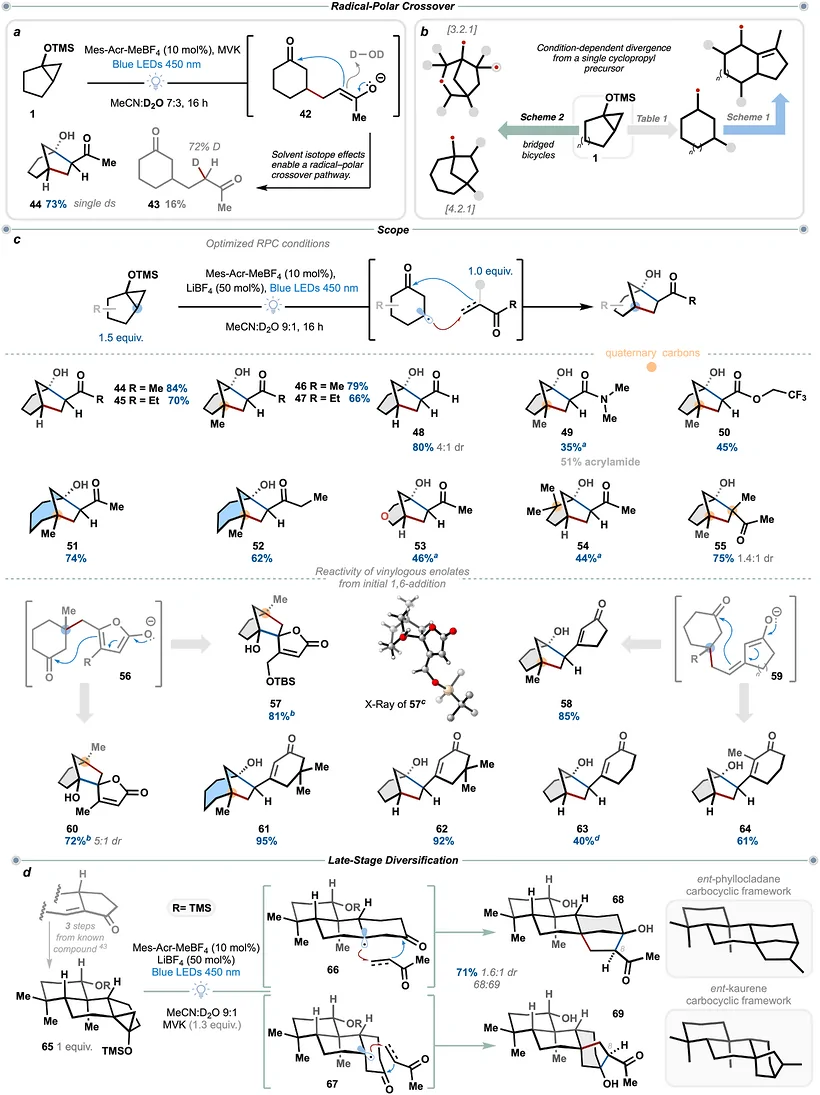
(a) Solvent isotope effects. (b) Divergent reactivity. (c) Radical–polar crossover reactions. (d) Late‐stage scaffold diversification. ^a^ 2.0 equiv. of cyclopropyl silyl ether. ^b^ Table 1 conditions used. ^c^ TBS‐protons and rotational disorder are omitted for clarity. ^d^ Without LiBF_4_.

We then sought to exploit this reactivity as a branching point for divergent scaffold formation. This manifold thus enables access to separate molecular scaffolds from a common cyclopropyl precursor under different reaction conditions (Scheme 2b). With optimized RPC conditions in hand (MeCN/D_2_O 9:1, 50 mol% LiBF_4_), we explored the generality of this transformation (Scheme 2c). A range of cyclopropyl silyl ethers and electron‐deficient alkenes underwent this transformation efficiently, furnishing diverse bridged bicyclic products in generally good yields and excellent diastereoselectivity. Ketone, aldehyde, amide, and ester‐containing substrates (**44‐50**) were well tolerated, affording [3.2.1] bicycles in synthetically useful yields (35–84%). Notably, sterically encumbered ketone **55** was obtained in 75% yield, setting two quaternary centers in one operation. Substitution on the cyclopropyl framework, including heteroatom incorporation (ether), and gem‐dimethyl substitution, was likewise accommodated (**53**, **54**). In addition, the manifold proved competent for the formation of more demanding [4.2.1] bridged systems (**51**, **52**), further expanding the scaffold classes accessible under these conditions.

In the case of γ,δ‐unsaturated butenolide electrophiles, the reaction furnishes γ‐spirocyclic products **57** and **60** with high efficiency, enabling a marked increase in molecular complexity within a single annulative transformation (Scheme 2c). The structure and relative stereochemistry of representative product **57** were confirmed by single‐crystal x‐ray diffraction [37]. Notably, the increase in structural complexity observed here [38, 39] highlights the capacity of this reaction manifold to access densely functionalized scaffolds (see Supporting Information for details).

The RPC‐derived branch further extends to 1,6‐addition substrates, enabling vinylogous enolate reactivity. Reactions with vinyl cyclopentenone‐ and cyclohexenone‐based acceptors afforded bridged bicycles **58** and **62–64** in 40%–92% yield as single diastereomers, constituting unusual examples of undirected intramolecular vinylogous aldol reactivity arising from this reaction manifold [40, 41, 42]. This branch also enables access to a [4.2.1] bridged bicycle (**61**), underscoring the structural diversity accessible herein.

Finally, the application of this transformation in late‐stage scaffold diversification was demonstrated using an advanced cyclopropyl intermediate (**65**), which underwent a ring opening / RPC sequence in 71% isolated yield to furnish diastereomeric products corresponding to divergent entries into the carbocyclic skeletons of the phyllocladane (**68**) and kaurene (**69**) natural product families (Scheme 2d) [43, 44, 45, 46, 47]. Notably, in this late‐stage application, cyclopropyl silyl ether **65** was employed as the limiting reagent, further supporting the practical utility of the approach in target‐oriented synthesis settings.

In conclusion, we have established a photochemical platform for divergent scaffold formation in which cyclopropyl silyl ethers undergo radical ring opening to generate β‐carbonyl radicals, followed by controlled intermolecular alkene addition. Beyond enabling β–β coupling without significant polymerization, this platform allows the common intermediate to be redirected into either annulative reactivity or radical–polar crossover pathways, thereby providing access to three different product classes from a common cyclopropyl precursor. Conditions that favor radical–polar crossover further extend the scope of the transformation to bridged and spirocyclic architectures, including products arising from vinylogous aldol reactivity. The synthetic utility of this strategy is further demonstrated through late‐stage scaffold diversification to the kaurene and phyllocladane frameworks, and the 5‐step total synthesis of (−)‐*ent*‐melicodenone C (**41**).

## Conflicts of Interest

The authors declare no conflicts of interest.

## Supporting information




**Supporting File**: Detailed experimental procedures, photochemical setup, compound characterization data, and x‐ray crystallographic data (PDF).

## Data Availability

The data that supports the findings of this study are available in the Supporting Information of this article.
