# Supplementary material for: Photochemical Ring Opening of Cyclopropyl Silyl Ethers Enables β–β Coupling, Radical‐Polar Crossover and Annulation
Source: Angew Chem Int Ed Engl. 2026 Jul 23;65(37):e7669030. doi: 10.1002/anie.7669030 (PMC13548954; doi:10.1002/anie.7669030)
Supplement: Supplementary file 1 [file ANIE-65-e7669030-s001.pdf]

Supporting Information

Photochemical Ring Opening of Cyclopropyl Silyl Ethers Enables β–β Coupling, Radical-Polar Crossover and Annulation.

Jacop Rydén[a], Eilidh Young[a], Wilma Olsson[a], Eskil Flodén, Mattias Tan[a], and Sebastian Clementson*[a]

a) Centre for Analysis and Synthesis, Department of Chemistry, Lund University Box
124, SE-221 00 Lund, Sweden.
E-mail: sebastian.clementson@chem.lu.se

Table of Content

I. General procedures S3

II. Experimental Section S5

Cyclopropyl Silyl Ether Preparation S5

Method A1 ....S5

Method A2 ....S5

Practical Accessibility..S5

Cyclopropyl Silyl Ether Synthesis and Characterization S6

Electron-Deficient Alkenes S10

Synthesis and Characterization..S10

Photochemical Procedures S11

Reaction Optimization S11

Deuterium Labeling and TEMPO Trapping S13

Method B1 (for Volatile Alkenes)..S15

Method B2 (for Non-volatile Alkenes) S15

Method C1 (for Volatile Alkenes) S15

Method C2 (for Non-volatile Alkenes) S16

Synthesis and Characterization of β–β coupled Products S16

Synthesis and Characterization of Radical-polar Crossover Products..S31

Late-stage Diversification..S40

Synthesis and Characterization of Compounds S22, 65, 68 and 69..S40

Downstream Reactions..S44

Derivatization Attempts S44

Synthesis and Characterization of Compounds 37, 38, 40 and 41 ....S44

III. X-ray Crystallography Data S48

X-ray Crystallographic Structure of 57 ....S48

X-ray Crystallographic Tables..S49

IV. Supplementary References S59

V. Copies of ^{1}H, ^{13}C and 2D NMR of Compounds S5-S7, S9-S13, S15-S16, S21-S23, 1-3, 5-41, 43-55, 57-58, 60-65, and 68-69 ....S60

SAFETY STATEMENT: No unusual or unexpected safety issues were encountered in this work. Sealed vessels used at elevated temperatures should be able to withstand pressure. These vessels were inspected for damage prior to each reaction.

I. General procedures

Synthesis, general: All reactions were carried out in oven-dried glassware under a nitrogen atmosphere, unless otherwise stated. Reactions were heated using a metal block on a hotplate with feedback temperature control, unless otherwise stated. Room temperature (rt) refers to temperatures in a range between 20 – 22 °C. Mixtures were concentrated under reduced pressure by rotary evaporation. Reactions were monitored by TLC using aluminum-backed plates (Merck 60F_{254} silica gel) and visualized with UV-light (254 nm) and/or staining with alkaline $KMnO_4$, phosphomolybdic acid stain, anisaldehyde stain, or DDQ stain (10% in toluene). Flash column chromatography was performed using silica gel (Acros 40 – 60 µm, 60 Å). Preparatory TLC was performed on pre-coated glass backed Macherey-Nagel 0.25 mm silica gel 60 plates. Petroleum ether (PE) refers to the fraction boiling at 40 – 60 °C. For known compounds (references given) synthesized through new or modified procedures, copies of ^{1}H NMR spectra are generally provided. Yields refer to chromatographically and spectroscopically (^{1}H NMR and ^{13}C NMR) homogeneous material, unless otherwise stated.

Reagents and solvents: Anhydrous solvents (THF, DCM, Et_2O, toluene) were freshly collected after filtration through alumina columns on a solvent-purification system. Anhydrous DMSO was obtained as extra dry and used as received. Acetonitrile was used as received from *Fisher Scientific*. All other solvents and reagents were bought from commercial suppliers and used as received.

NMR spectroscopy: NMR spectra were recorded on a Bruker Ultrashield 400 plus (^{1}H at 400 MHz and $^{13}C\{^{1}H\}$ at 101 MHz) or on a Bruker Avance II (^{1}H at 500 MHz and $^{13}C\{^{1}H\}$ at 126 MHz) and processed using MestReNova version 14.2.0. Chemical shifts are given in ppm downfield from $SiMe_4$ using the residual signal of the solvent as reference: $CDCl_3$: δ = 7.26 for ^{1}H and δ = 77.16 for $^{13}C\{^{1}H\}$; C_6D_6: δ = 7.16 for ^{1}H and δ = 128.06 for $^{13}C\{^{1}H\}$. ^{1}H NMR spectra are reported as follows: chemical shifts (δ, ppm), multiplicity (s = singlet, d = doublet, t = triplet, q = quartet, sept. = septet, dd = doublet of doublets, dt = doublet of triplets, m = multiplet, br. = broad, app. = apparent), coupling constant (Hz) and integration. $^{13}C\{^{1}H\}$ NMR spectra are reported in chemical shifts. When two or fewer signals overlap in ^{13}C NMR, these signals are clearly noted. If three or more signals overlap, this is written as *multiple overlapping signals*.

Mass spectrometry: Mass spectra (HRMS) were recorded on a Micromass Q-TOF spectrometer (ESI) or on a LTQ Velos PRO orbitrap mass spectrometer (Thermo Scientific) (ESI).

IR spectroscopy: IR spectra were recorded on a Bruker ALPHA-P in ATR mode and selected peaks are reported as follows: wavenumbers (cm^{-1}).

Photoreactor setup: All photochemical reactions were irradiated using a PennOC/PennPhD Integrated Photoreactor (IPR). For these experiments, the 450 nm blue LED module (M2 photoreactor) was employed. Unless otherwise noted, reactions were stirred at 650 rpm and cooled using the maximum fan speed setting throughout irradiation. Reactions were routinely performed with multiple samples simultaneously positioned within the reactor, without any observable impact on reaction efficiency or reproducibility. Irradiation times were determined empirically for each substrate combination and are reported in the corresponding experimental procedures. Under these conditions, reaction vessels were maintained near rt (approximately 27 °C) for the duration of irradiation.

For certain reactions that required a higher temperature, an 8 mL vial was used in combination with the small vial-insertion – so that the vial had a shorter distance to the lamp. The fan was also put at minimum speed. This gave a temperature of approximately 40 °C.

Determination of diastereomeric ratio: Diastereomeric ratios were, when possible, determined by relative integration of diagnostic signals in the ^{1}H NMR spectra. For several compounds, no discernible or well-

resolved signals were observed in the ^{1}H NMR spectra, and the diastereomeric ratios were therefore estimated based on relative signal intensities in the ^{13}C NMR spectra. Although the ^{13}C NMR spectra were not recorded under quantitative conditions, the close structural similarity of the diastereomers allows estimation of their relative amounts by comparison of corresponding carbon signals. Ratios determined in this manner are reported as ca.

Yields determined by quantitative NMR spectroscopy were obtained by comparison of relevant product signals to the aromatic signal of mesitylene (1.0 equiv.) used as an internal standard.

Complexity quantification: Molecular complexity was quantified using the Böttcher molecular complexity score. Scores were calculated by submitting SMILES strings to the MolComplex web tool (https://www.molcomplex.org). Böttcher complexity scores for compounds **S1**, **S2**, **S3**, **S4**, and **57** were calculated based on SMILES (**Figure S1**).

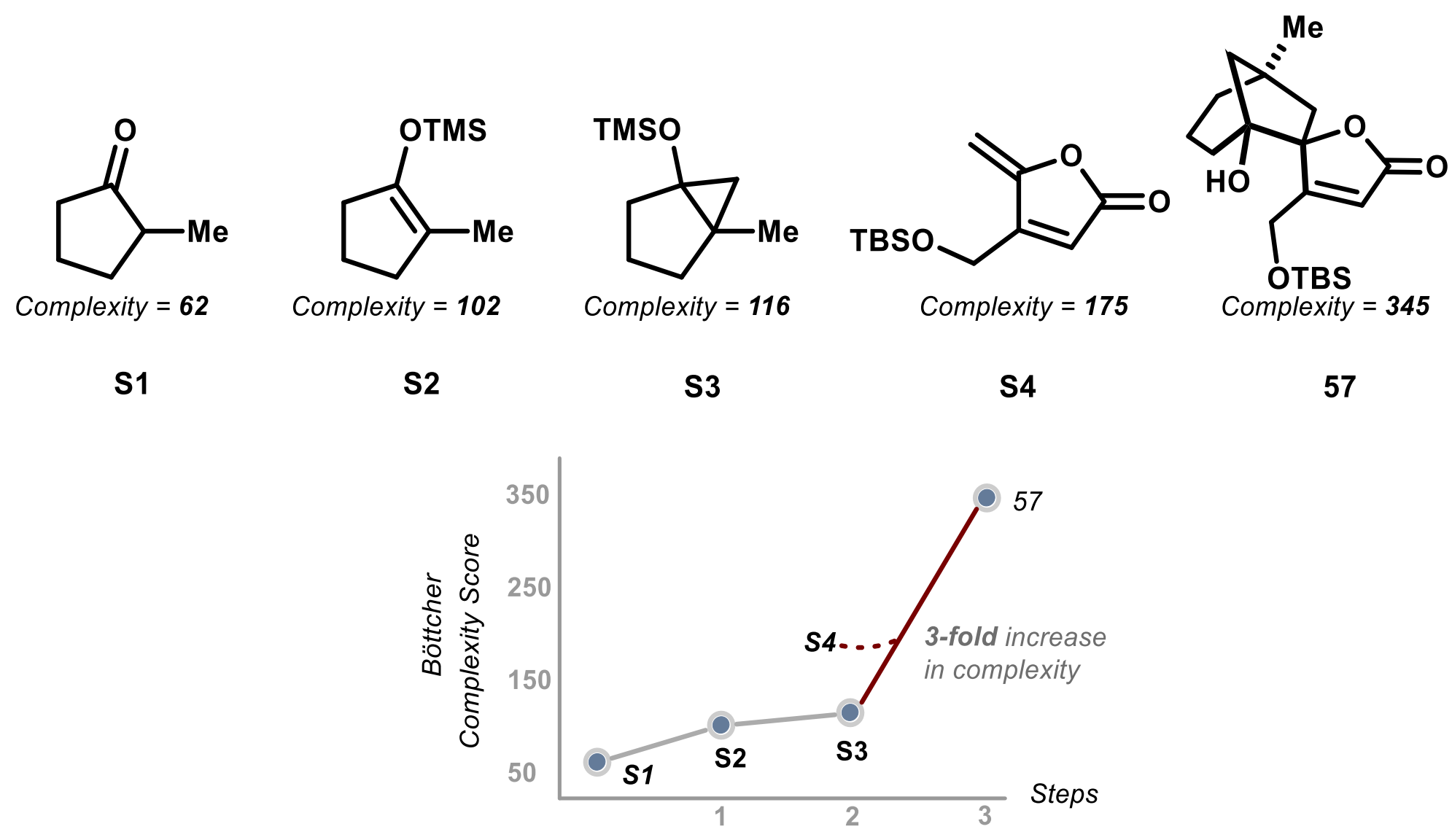

Figure S1. Depiction of the structural complexity of compounds **S1**, **S2**, **S3**, **S4** and **57**.

II. Experimental Section

Cyclopropyl Silyl Ether Preparation

Method A1

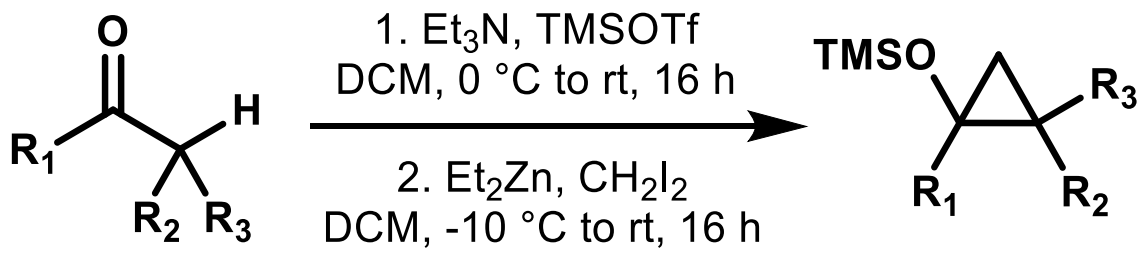

Under an inert atmosphere, Et_3N (4.0 equiv.) was added to a solution of ketone (1.0 equiv.) and dry DCM (0.2 M). The solution was cooled to 0 °C, and TMSOTf (1.5 equiv.) was added dropwise. After stirring at rt for 16 h, 10% aq. $NaHCO_3$ was added. The organic layer was separated, and the aqueous layer was further extracted with EtOAc. The combined organic layers were then dried over Na_2SO_4, filtered, and concentrated under reduced pressure. The obtained crude was used in the next step without further purification.

Under an inert atmosphere, the resulting silyl enol ether (1.0 equiv.) and CH_2I_2 (1.5 equiv.) were dissolved in dry DCM (0.1 M). The solution was cooled to −10 °C, and Et_2Zn (1.5 equiv., 1 M in *n*-hexane) was added dropwise. The solution was stirred at rt for 16 h, after which cold sat. aq. NH_4Cl was added to the mixture. The formed solids were filtered off. The organic layer was separated, and the aqueous layer was further extracted with DCM. The combined organic layers were then dried over $MgSO_4$, filtered, and concentrated under reduced pressure. The obtained crude was purified by flash column chromatography to afford pure cyclopropyl silyl ether.

Method A2

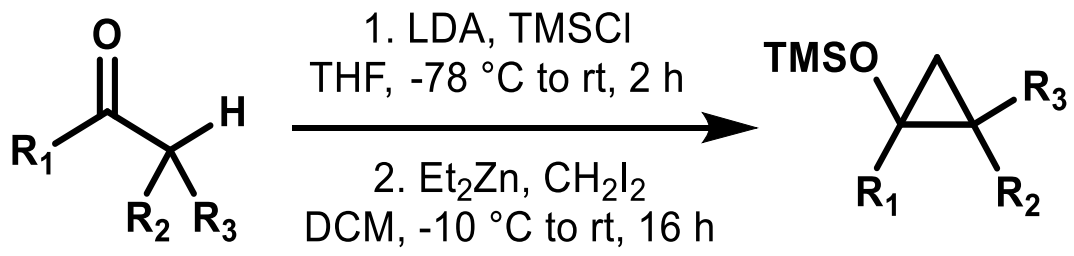

Under an inert atmosphere, *n*-BuLi (1.2 equiv., 2.5 M in *n*-hexane) was added to a solution of diisopropylamine (1.5 equiv.) in dry THF (0.25 M) at −78 °C. After stirring for 20 min, the ketone (1.0 equiv.) was added neat and dropwise (solid ketones were dissolved in minimal THF and added dropwise). After another 30 min, TMSCl (1.2 equiv.) was added, and the reaction was left to stir for one hour at −78 °C. Then, at rt Et_3N, 2% aq. $NaHCO_3$ and EtOAc were added. The organic layer was separated, and the aqueous layer was further extracted with EtOAc. The combined organic layers were then dried over Na_2SO_4, filtered, and concentrated under reduced pressure. The obtained crude was used in the next step without further purification.

Under an inert atmosphere, the resulting silyl enol ether (1.0 equiv.) and CH_2I_2 (1.5 equiv.) were dissolved in dry DCM (0.1 M). The solution was cooled to −10 °C, and Et_2Zn (1.5 equiv., 1 M in *n*-hexane) was added dropwise. The solution was stirred at rt for 16 h, after which cold sat. aq. NH_4Cl was added to the mixture. The formed solids were filtered off. The organic layer was separated, and the aqueous layer was further extracted with DCM. The combined organic layers were then dried over $MgSO_4$, filtered, and concentrated under reduced pressure. The obtained crude was purified by flash column chromatography to afford pure cyclopropyl silyl ether.

Practical Accessibility

The cyclopropyl silyl ethers used in this study were prepared in a modular two-step sequence from the corresponding ketones, requiring one single purification. With the exception of compound **3**, which presented chemoselectivity issues during the cyclopropanation step, the cyclopropyl silyl ether substrates were obtained in 33–98% yield over two steps. In terms of handling, most cyclopropyl silyl ethers were

stable for storage at −20 °C for up to approximately 4 months, after which desilylation started to be noticeable. Cyclopropyl silyl ethers containing heteroatoms in the ring were generally less stable and were therefore used immediately after preparation. In the coupling studies, reactions were most commonly conducted with 1.5 equivalents of the cyclopropyl partner; in selected cases, 1.2 equivalents proved sufficient, whereas 2.0 equivalents were beneficial in more demanding cases. Importantly, in the late-stage applications leading to the kaurene and phyllocladane frameworks (**Scheme 2**), the cyclopropyl silyl ether was employed as the limiting reagent (1.0 equiv.), highlighting that this manifold also remains effective when the cyclopropyl represents the more valuable of the two components.

Cyclopropyl Silyl Ether Synthesis and Characterization

The relative stereochemistry of the cyclopropyl silyl ethers was not determined, and in several cases, these were isolated as diastereomeric mixtures.

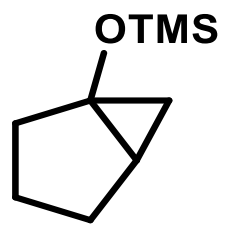

1

Cyclopropyl silyl ether 1. Compound **1** was prepared from cyclopentanone according to *Method A1,* and the analytical data (^{1}H, ^{13}C NMR) were in agreement with those previously described.[1]

^{1}H NMR (400 MHz, $CDCl_3$) δ 1.98 – 1.90 (m, 1H), 1.90 – 1.82 (m, 1H), 1.60 (dt, J = 12.8, 7.8 Hz, 1H), 1.47 (dd, J = 12.3, 7.8 Hz, 1H), 1.28 (dt, J = 9.1, 4.4 Hz, 1H), 1.16 – 0.99 (m, 1H), 0.88 – 0.81 (m, 1H), 0.75 (ddd, J = 9.3, 5.5, 1.8 Hz, 1H), 0.58 – 0.51 (m, 1H), 0.13 (s, 9H).

^{13}C NMR (101 MHz, $CDCl_3$) δ 65.2, 34.0, 26.5, 23.5, 21.4, 14.6, 1.1.

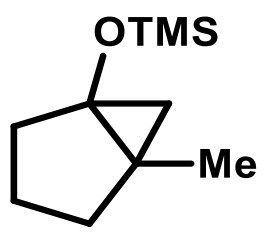

S3

Cyclopropyl silyl ether S3. The silyl enol ether was prepared from 2-methylcyclopentanone according to the one-step procedure reported by Nicolaou *et al*.[2] The crude silyl enol ether was then used to prepare compound **S3** according to the second step of *Method A1/A2*. Analytical data (^{1}H, ^{13}C NMR, HRMS) were in agreement with those previously described.[3]

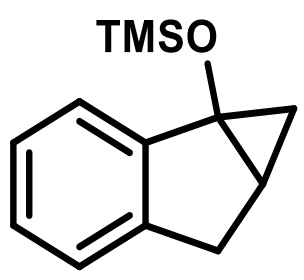

S5

Cyclopropyl silyl ether S5. Compound **S5** was prepared from 1-indanone according to the reported procedure by Shenvi *et al.*, and the analytical data (^{1}H, ^{13}C NMR) were in agreement with those previously described.[4]

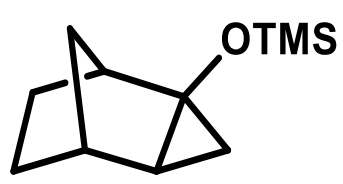

S6

Cyclopropyl silyl ether S6. Compound **S6** was prepared from norcamphor according to *Method A1*, and the analytical data (^{1}H, ^{13}C NMR, HRMS) were in agreement with those previously described.[5]

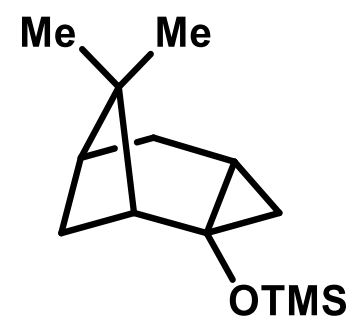

S7

Cyclopropyl silyl ether S7. Compound **S7** was prepared from nopinone according to *Method A1*, and the analytical data (^{1}H, ^{13}C NMR, HRMS) were in agreement with those previously described.[5]

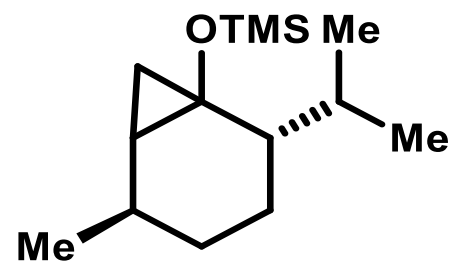

35

Cyclopropyl silyl ether 35. Compound **35** was prepared from (−)-menthone according to *Method A2,* and the analytical data (^{1}H, ^{13}C NMR, HRMS) were in agreement with those previously described.[6]

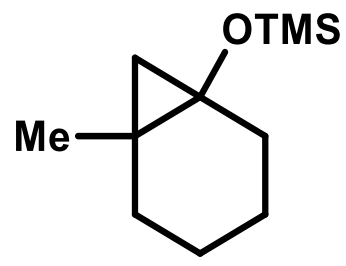

S8

Cyclopropyl silyl ether S8. Compound **S8** was prepared from 2-methylcyclohexan-1-one according to *Method A1,* and the analytical data (^{1}H, ^{13}C NMR, HRMS) were in agreement with those previously described.[1]

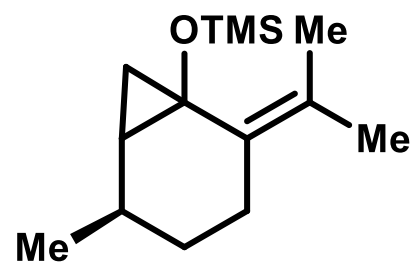

39

Cyclopropyl silyl ether 39. Compound **39** was prepared from (*R)*-pulegone according to *Method A2.* Purification by flash column chromatography (silica gel, 0 to 5% EtOAc in PE) afforded pure **39** (1.22 g, 5.12 mmol, 33% over two steps) as a colorless oil.

R_f 0.24 (silica gel, PE)

^{1}H NMR (400 MHz, $CDCl_3$) δ 2.16 – 2.08 (m, 1H), 2.06 – 1.95 (m, 1H), 1.88 (d, *J* = 2.0 Hz, 3H), 1.80 – 1.69 (m, 1H), 1.63 (s, 3H), 1.36 – 1.27 (m, 1H), 1.18 (d, *J* = 7.1 Hz, 3H), 1.16 – 1.10 (m, 1H), 1.02 (dd, *J* = 10.4, 4.8 Hz, 1H), 0.93 – 0.88 (m, 1H), 0.23 (dd, *J* = 6.3, 4.8 Hz, 1H), 0.06 (s, 9H).

^{13}C NMR (101 MHz, $CDCl_3$) δ 131.7, 130.6, 55.8, 28.5, 28.2, 27.1, 22.9, 22.2, 22.0, 20.2, 19.9, 1.0.

HRMS (ESI+) *m*/*z*: calcd. for $C_{14}H_{26}NaOSi^+$ $[M+Na]^+$: 261.1645; found: 261.1644, (Δ = −0.38 ppm)

IR (ATR)/cm^{-1} 2957, 2920, 2872, 1451, 1401, 1372, 1247, 1189, 1105, 1037, 1026, 925, 868, 835, 748

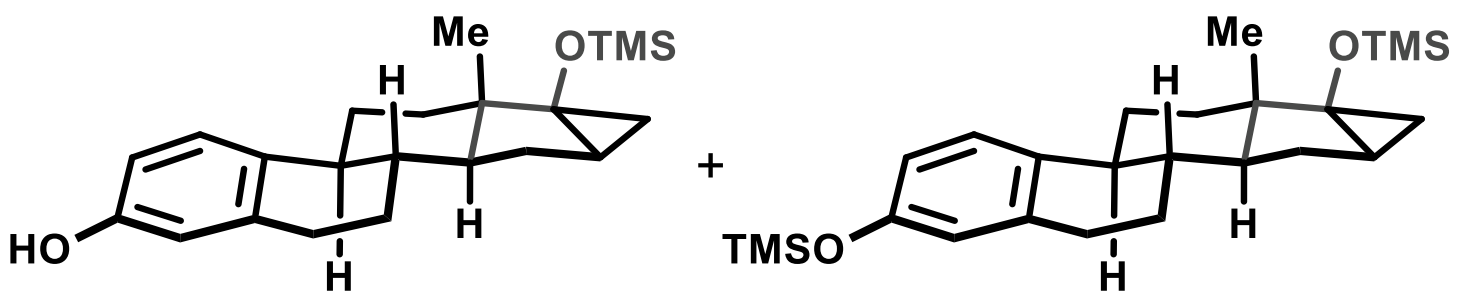

S9a S9b

Cyclopropyl silyl ethers S9a and **S9b.** Compounds **S9a** and **S9b** were prepared from estrone according to *Method A1*. Purification by flash column chromatography (silica gel, 2% to 10% EtOAc in PE) afforded **S9a** (89.9 mg, 0.252 mmol, 25% over two steps) and **S9b** (109 mg, 0.254 mmol, 25%, over two steps) as white crystals and white powder, respectively (Combined yield 50% over two steps).

R_f 0.48 (**S9a**); 0.67 (**S9b**) (silica gel, 10% EtOAc in PE)

^{1}H NMR (400 MHz, $CDCl_3$) δ 7.13 (d, *J* = 8.4 Hz, 1H), 6.62 (dd, *J* = 8.4, 2.7 Hz, 1H), 6.55 (d, *J* = 2.6 Hz, 1H), 4.65 (s, 1H), 2.87 – 2.76 (m, 2H), 2.37 – 2.27 (m, 1H), 2.13 (td, *J* = 10.9, 4.6 Hz, 1H), 1.87 – 1.76 (m, 2H), 1.56 – 1.42 (m, 5H), 1.32 – 1.23 (m, 2H), 1.00 (dd, *J* = 6.2, 4.0 Hz, 1H), 0.96 (s, 3H), 0.88 – 0.80 (m, 1H), 0.74 (dd, *J* = 9.0, 6.3 Hz, 1H), 0.14 (s, 9H). (**S9a**)

^{1}H NMR (400 MHz, $CDCl_3$) δ 7.11 (d, *J* = 8.4 Hz, 1H), 6.62 (dd, *J* = 8.4, 2.3 Hz, 1H), 6.55 (d, *J* = 2.3 Hz, 1H), 2.83 – 2.73 (m, 2H), 2.36 – 2.28 (m, 1H), 2.14 (dt, *J* = 10.6, 5.4 Hz, 1H), 1.87 – 1.77 (m, 2H), 1.56 – 1.43 (m, 5H), 1.30 – 1.25 (m, 2H), 1.00 (dd, *J* = 5.9, 4.0 Hz, 1H), 0.97 (s, 3H), 0.88 – 0.85 (m, 1H), 0.74 (dd, *J* = 9.0, 6.3 Hz, 1H), 0.26 (s, 9H), 0.15 (s, 9H). (**S9b**)

^{13}C NMR (126 MHz, $CDCl_3$) δ 153.2, 138.2, 132.9, 126.3, 115.1, 112.5, 69.9, 46.6, 44.5, 43.3, 37.6, 33.5, 29.5, 27.3, 26.3, 25.8, 21.3, 15.3, 13.5, 1.3. (**S9a**)

^{13}C NMR (101 MHz, $CDCl_3$) δ 152.9, 137.8, 133.5, 126.0, 120.0, 117.1, 70.0, 46.7, 44.6, 43.4, 37.6, 33.6, 29.9, 27.5, 26.3, 25.9, 21.4, 15.5, 13.6, 1.4, 0.3. (**S9b**)

HRMS (ESI+) *m*/z: calcd. for $C_{22}H_{33}O_2Si^+$ [M+H]$^+$: 357.2244; found: 357.2244, (Δ = 0 ppm) (**S9a**); *m*/z: calcd. for $C_{25}H_{41}O_2Si_2^+$ [M+H]$^+$: 429.2640; found: 429.2648, (Δ = 1.86 ppm) (**S9b**)

IR (ATR)/cm^{-1} 3363, 2919, 2855, 1500, 1440, 1250, 1215, 872, 837, 750 (**S9a**); 2956, 2925, 2861, 1607, 1495, 1285, 1249, 1232, 1112, 953, 880, 835, 739 (**S9b**)

MP 165-170 °C (**S9a**); 67 °C (**S9b**)

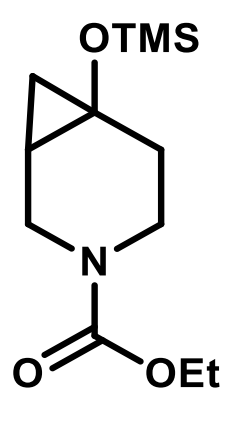

S10

Cyclopropyl silyl ether S10. Compound **S10** was prepared from ethyl 4-oxopiperidine-1-carboxylate according to *Method A1*. Purification by flash column chromatography (silica gel, 5% to 30% EtOAc in PE) afforded **S10** (741 mg, 2.88 mmol, 40% over two steps) as a colorless oil.

R_f 0.29 (silica gel, 10% EtOAc in PE)

^{1}H NMR (400 MHz, $CDCl_3$) δ 4.08 (q, *J* = 7.1 Hz, 2H), 3.84 – 3.73 (m, 1H), 3.55 – 3.35 (m, 2H), 3.08 – 2.94 (m, 1H), 2.18 – 2.01 (m, 2H), 1.22 (app. t, *J* = 7.1 Hz, 4H), 0.92 (dd, *J* = 10.4, 6.0 Hz, 1H), 0.47 (t, *J* = 5.8 Hz, 1H), 0.12 (s, 9H). (Summary of rotamers)

^{13}**C NMR** (126 MHz, $CDCl_3$) δ 155.8, 61.3, 54.5, 42.4, 40.2, 31.5, 31.1, 18.9, 16.5, 14.8, 1.3. (Summary of rotamers, note: one signal split in two (31.5 and 31.1))

HRMS (ESI+) *m*/z: calcd. for $C_{12}H_{24}NO_3Si^+$ $[M+H]^+$: 258.1520; found: 258.1521, (Δ = 0.39 ppm)

IR (ATR)/cm^{-1} 2957, 2864, 1697, 1428, 1248, 1185, 1119, 932, 836, 752

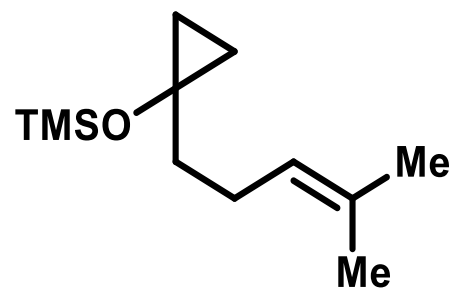

3

Cyclopropyl silyl ether 3. Compound **3** was prepared from 6-methylhept-5-en-2-one according to *Method A2*. Purification by flash column chromatography (silica gel, 0% to 5% EtOAc in PE) afforded **3** (387 mg, 1.82 mmol, 12% over two steps) as a colorless oil.

$\mathbf{R_f}$ 0.17 (silica gel, PE)

^{1}**H NMR** (400 MHz, $CDCl_3$) δ 5.13 (dddd, *J* = 7.2, 5.8, 2.7, 1.4 Hz, 1H), 2.18 (app. q, *J* = 7.4 Hz, 2H), 1.68 (d, *J* = 1.3 Hz, 3H), 1.61 (s, 3H), 1.54 – 1.47 (m, 2H), 0.74 – 0.68 (m, 2H), 0.45 – 0.40 (m, 2H), 0.13 (s, 9H).

^{13}**C NMR** (101 MHz, $CDCl_3$) δ 131.6, 124.5, 57.2, 39.5, 25.8, 24.9, 17.7, 13.2, 1.4.

HRMS (ESI+) *m*/z: calcd. for $C_{12}H_{25}OSi^+$ $[M+H]^+$: 213.1669; found: 213.1664, (Δ = −2.35 ppm)

IR (ATR)/cm^{-1} 3005, 2960, 2924, 2858, 1454, 1321, 1250, 1029, 944, 864, 838, 751

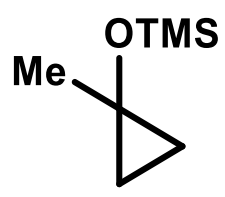

S11

Cyclopropyl silyl ether S11. From commercially acquired (isopropenyloxy)trimethylsilane, compound **S11** was prepared according to the second step in *Method A1/A2* (in pentane). The crude mixture was used directly in the photochemical reaction. Crude analytical data (^{1}H, ^{13}C NMR, HRMS) were in agreement with those previously described[7] (note: volatile compound).

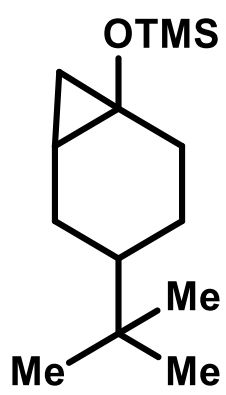

S12

Cyclopropyl silyl ether S12. Compound **S12** was prepared from 4-(*tert*-butyl)cyclohexan-1-one according to *Method A1*. Purification by flash column chromatography (silica gel, 0% to 5% EtOAc in PE) afforded **S12** (1.51 g, 6.30 mmol, 60% over two steps) as a colorless oil.

$\mathbf{R_f}$ 0.52 (silica gel, 5% EtOAc in PE)

^{1}**H NMR** (400 MHz, $CDCl_3$) δ 2.27 – 2.10 (m, 1H), 1.94 – 1.78 (m, 1H), 1.75 – 1.55 (m, 1H), 1.52 – 1.38 (m, 1H), 1.34 – 1.20 (m, 1H), 1.12 – 0.90 (m, 2H), 0.84 – 0.80 (m, 1H), 0.78 (app. d, *J* = 8.5 Hz, 9H), 0.64 – 0.54 (m, 1H), 0.30 – 0.21 (m, 1H), 0.16 – 0.07 (s, 9H). (Summary of diastereomers)

^{13}C NMR (101 MHz, $CDCl_3$) δ 57.0, 55.8, 44.5, 40.9, 35.3, 33.2, 32.3, 32.2, 27.5, 27.3, 26.7, 24.4, 24.3, 22.9, 22.1, 19.0, 18.63, 18.56, 1.48, 1.46. (Summary of diastereomers)

HRMS (ESI+) *m*/z: calcd. for $C_{14}H_{29}OSi^+$ [M+H]$^+$: 241.1982; found: 241.1977, (Δ = −2.07 ppm)

IR (ATR)/cm^{-1} 2994, 2954, 2862, 1467, 1366, 1249, 1206, 1164, 1074, 945, 925, 860, 836, 750

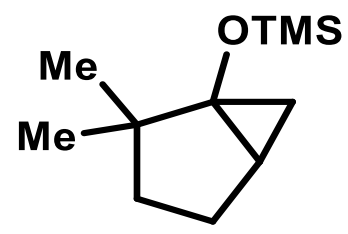

S13

Cyclopropyl silyl ether S13. Compound **S13** was prepared from 2,2-dimethylcyclopentan-1-one according to *Method A1*. Purification by flash column chromatography (silica gel, 0% to 5% EtOAc in PE) afforded **S13** (874 mg, 4.41 mmol, 98% over two steps) as a colorless oil.

R_f 0.60 (silica gel, 5% EtOAc in PE)

^{1}H NMR (500 MHz, $CDCl_3$) δ 1.91 (tdd, *J* = 11.9, 7.7, 4.1 Hz, 1H), 1.44 – 1.35 (m, 2H), 1.30 – 1.25 (m, 1H), 1.02 (s, 6H), 1.01 – 0.96 (m, 1H), 0.67 (dd, *J* = 9.0, 5.7 Hz, 1H), 0.60 (dd, *J* = 5.9, 4.2 Hz, 1H), 0.11 (s, 9H).

^{13}C NMR (126 MHz, $CDCl_3$) δ 70.7, 41.0, 35.9, 24.2, 24.2, 24.0, 23.7, 13.8, 1.4.

HRMS (ESI+) *m*/z: calcd. for $C_{12}H_{23}O_3Si^-$ [M+HCOO]$^-$: 243.1422; found: 243.1427, (Δ = 2.06 ppm)

IR (ATR)/cm^{-1} 2957, 2868, 1470, 1251, 1215, 1148, 1132, 960, 904, 883, 840

Electron-Deficient Alkenes

Synthesis and Characterization

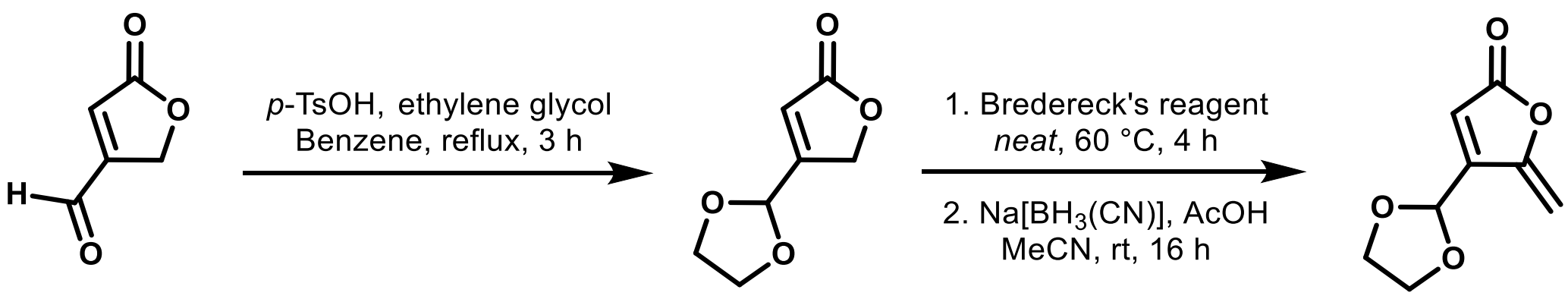

S14 **S15**, 65% **S16**, 36%

Acetal product S15. A solution of *p*-TsOH (618 mg, 3.25 mmol), ethylene glycol (7.27 mL, 130 mmol), aldehyde **S14** (7.29 g, 65.0 mmol) (prepared according to the procedure of Lattmann *et al.*[8]), and benzene (550 mL) was refluxed for 3 h in a Dean-Stark apparatus. Subsequently, the reaction mixture was concentrated under reduced pressure, and the crude mixture was purified by flash column chromatography (silica gel, 20% EtOAc in PE) to afford **S15** (6.60 g, 42.3 mmol, 65%).

^{1}H NMR (400 MHz, $CDCl_3$) δ 6.17 (td, *J* = 2.0, 1.0 Hz, 1H), 5.73 (d, *J* = 0.9 Hz, 1H), 4.88 (dd, *J* = 2.1, 0.7 Hz, 2H), 4.08 – 3.96 (m, 4H).

^{13}C NMR (101 MHz, $CDCl_3$) δ 172.9, 164.9, 118.5, 98.3, 70.3, 65.6.

HRMS (ESI+) *m*/z: calcd. for $C_7H_9O_4^+$ [M+H]$^+$: 157.0495; found: 157.0496, (Δ = 0.64 ppm)

IR (ATR)/cm^{-1} 3490, 3114, 2954, 2895, 1777, 1726, 1310, 1227, 1140, 1077, 1025, 940, 880

Lactone product S16. Under an inert atmosphere, Bredereck's reagent (2.04 mL, 9.88 mmol) was added neat to acetal **S15** (1.54 g, 9.85 mmol). The blue mixture was heated to 60 °C and left to stir for 4 h. The brown crude mixture was directly purified by flash column chromatography (silica gel, 40% to 60% EtOAc in PE) to afford an enamine intermediate, which was directly used in the subsequent step.

To a solution of the enamine intermediate and MeCN (200 mL), Na[BH_3(CN)] (804 mg, 12.8 mmol) was added. Subsequently, acetic acid (8.78 mL, 153 mmol) was added dropwise to the mixture. The yellow solution was covered in aluminium foil and was left to stir for 16 h at rt. Then, 10% aq. $NaHCO_3$ (30 mL) and ether (30 mL) were added. The organic layer was separated, and the aqueous layer was further extracted with ether (2 x 15 mL) and EtOAc (15 mL). The combined organic layers were then dried over Na_2SO_4, filtered, and concentrated under reduced pressure. The crude mixture was purified by flash column chromatography (silica gel, 10% to 40% EtOAc in PE) to afford **S16** (596 mg, 3.55 mmol, 36% over two steps) as a colorless oil. *Compound **S16** rapidly polymerized upon concentration and was therefore used immediately after purification. As a result, IR and HRMS data could not be reliably obtained and are not reported. Additionally, this complicated the acquisition of longer ^{13}C NMR.*

^{1}H NMR (400 MHz, $CDCl_3$) δ 6.30 – 6.27 (m, 1H), 5.85 (d, *J* = 1.2 Hz, 1H), 5.29 (dd, *J* = 2.7, 1.8 Hz, 1H), 5.17 (dd, *J* = 2.7, 0.9 Hz, 1H), 4.05 (app. s, 4H).

^{13}C NMR (101 MHz, $CDCl_3$) δ 168.2, 154.5, 152.8, 118.8, 97.9, 97.6, 65.5.

Photochemical Procedures

Reaction Optimization

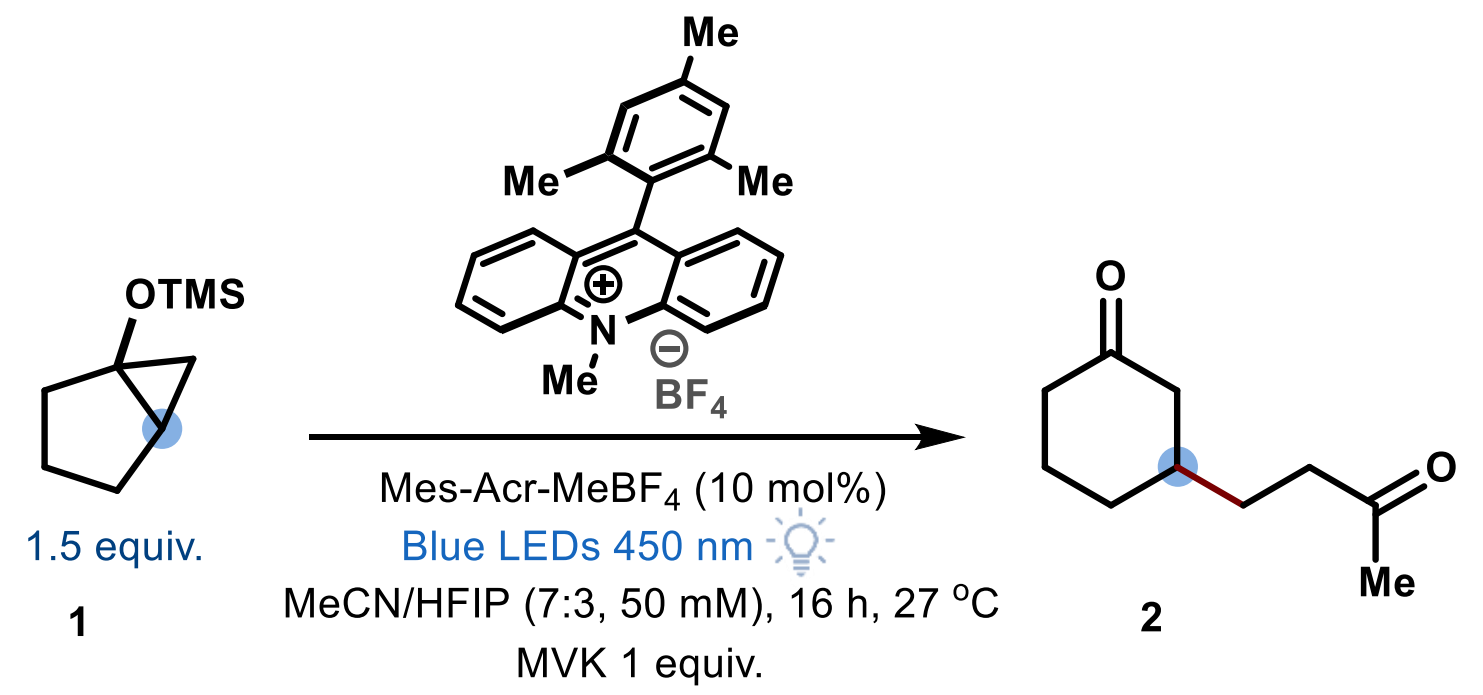

entry[a]	*deviation*	*yield*[b]
1	none	93%[c]
2	(0.15 M)	64%
3	4CzIPN (10 mol%)	trace
4	H_2O inst. of HFIP	60%
5	Mes-Acr-MeBF_4 (5 mol%)	78%
6	1.2 equiv. of **1**	74%[c]
7	no light	0%
8	Mes-Acr-PhBF_4 (10 mol%)	89%
9	3 equiv. of TEMPO	0%
10	MeOH inst. of HFIP	43%
11	(75 mM)	76%
12	Mes-Acr-PhBF_4 (10 mol%)	89%
13	1.8 equiv. of **1**, (75 mM)	85%
14	1.0 mmol scale	89%

[a] 0.1 mmol scale. [b] NMR yields using mesitylene as an internal standard. [c] isolated yield.

Figure S2. Complete reaction optimization of the β,β–coupling.

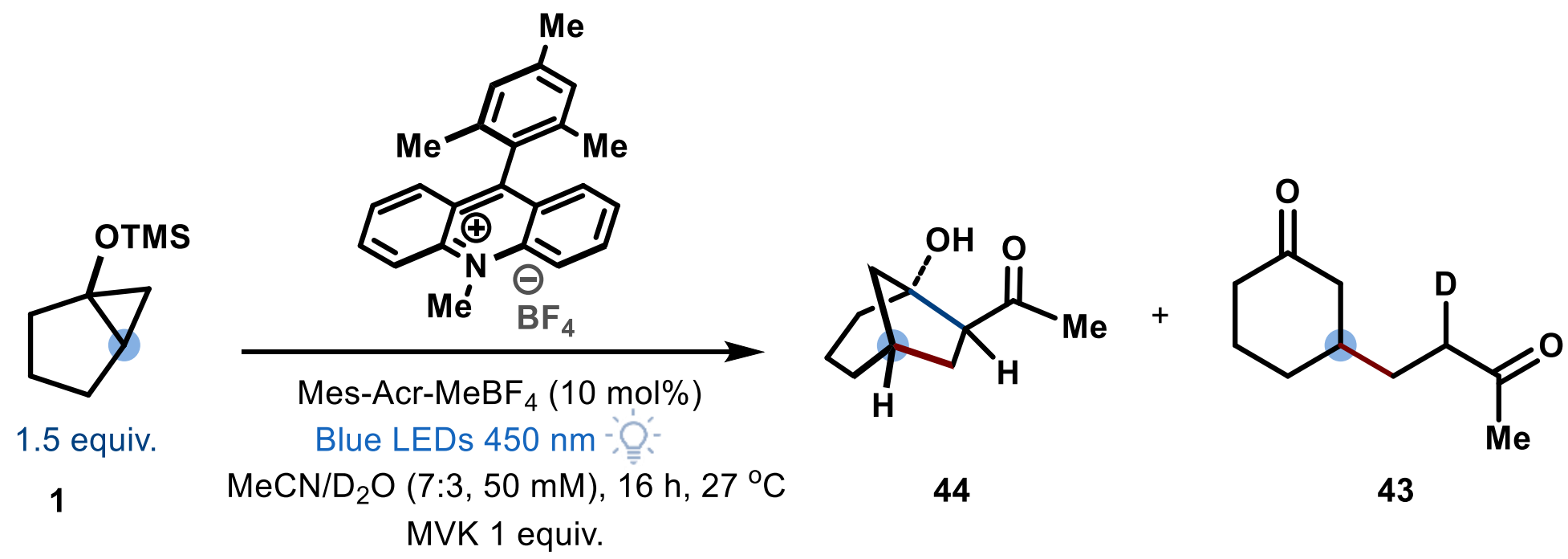

entry[a]	*deviation*	*yield*[b] ***44***	***43***	*44/43 ratio*
1	none	73%[c]	16%	4.6
2	H_2O inst. of D_2O	30%	60%[d]	0.5
3	HFIP inst. of D_2O	nr	93%[d]	-
4	*t*-BuOH inst. of D_2O	46%	52%	0.9
5	iPrOH inst. of D_2O	34%	59%	0.6
6	*t*-AmOH inst. of D_2O	26%	32%	0.8
7	Neopentyl OH inst. of D_2O	28%	33%	0.9
8	MeCN/D_2O (9:1)	57%	9%	6.3
9	15 mol% $Mg(OTf)_2$	30%	19%	1.6
10	15 mol% $LiBF_4$	48%	12%	4.0
11	15 mol% $LiBF_4$, *t*-AmOH inst. of D_2O	15%	42%	0.4
12	50 mol% $LiBF_4$	59%	12%	4.9
13	**50 mol% $LiBF_4$, (9:1)**	**79%**	**11%**	**7.2**
14	1 equiv. $LiBF_4$, (9:1)	78%	12%	6.5
15	MeOD inst. of D_2O (9:1)	30%	41%	0.7
16	50 mol% $Cu[MeCN]_4PF_6$, (9:1)	26%	46%	0.6
17	50 mol% $MgCl_2$, (9:1)	66%	18%	3.7
18	**50 mol% $ZnCl_2$, (9:1)**	**84%**	**14%**	**6.0**
19	50 mol% $LiBF_4$, 3 vol% D_2O in MeCN	41%	6%	6.8
20	50 mol% $LiBF_4$, 5 vol% D_2O in MeCN	61%	7%	8.7
21	50 mol% $ZnCl_2$, 5 vol% D_2O in MeCN	59%	8%	7.4
22	50 mol% $LiBF_4$, no D_2O	3%	nr	-
23	50 mol% $LiBF_4$, (9:1), 420 nm	52%	9%	5.8

[a] *0.1 mmol scale.* [b] *NMR yields using mesitylene as an internal standard.* [c] *isolated yield.* [d] *No D-incorporation.*

Figure S3. Complete reaction optimization of the radical-polar crossover reaction. Most successful entries are highlighted in bold.

Photocatalysts used in this work

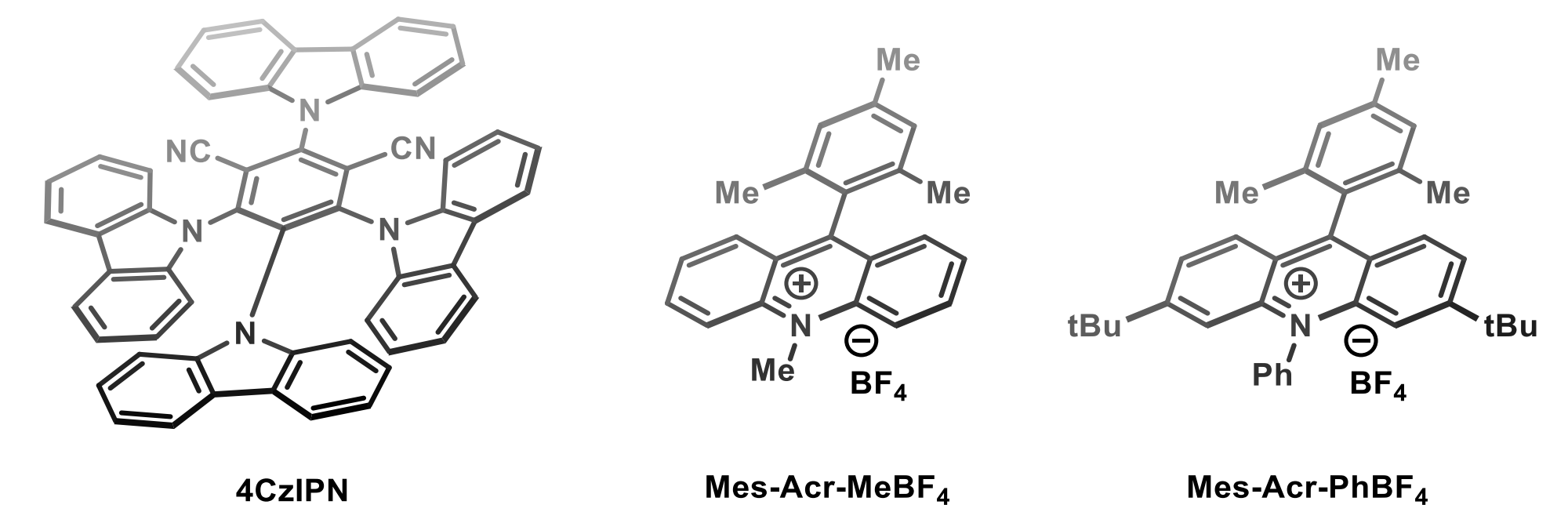

Figure S4. The photocatalysts used in the optimization studies.

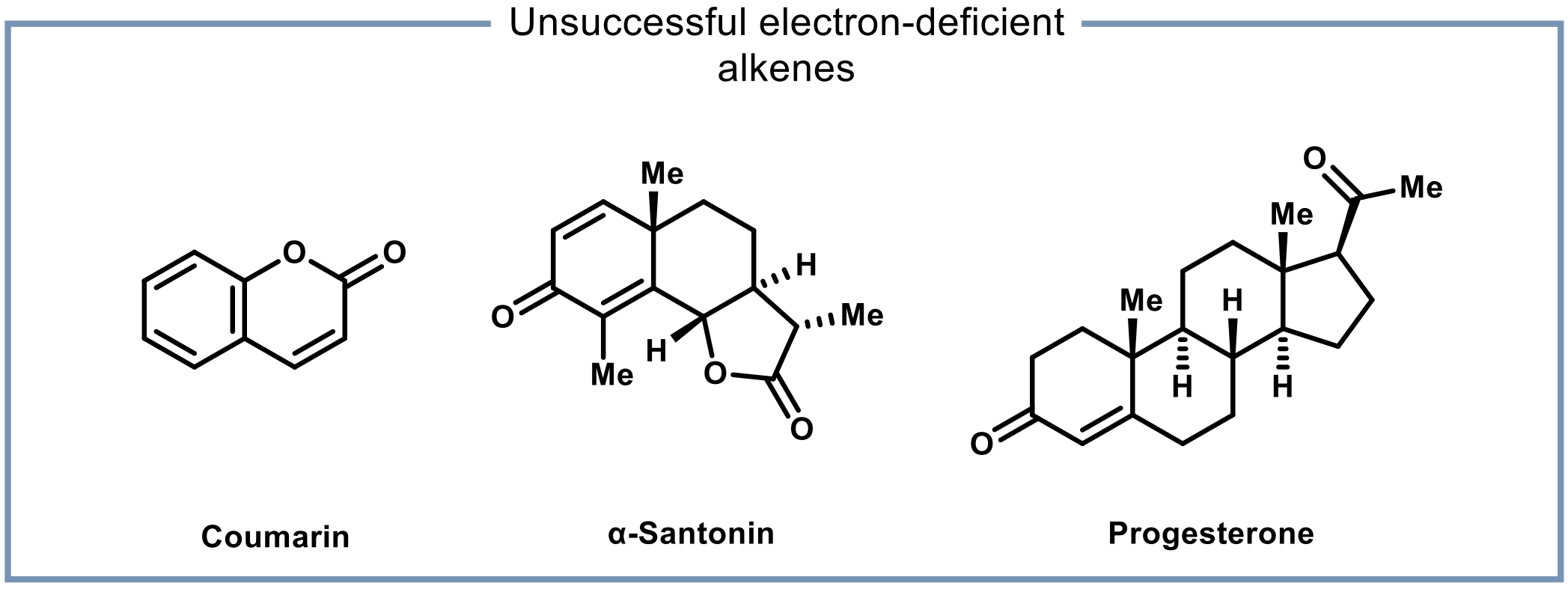

Figure S5. The unsuccessful alkenes that yielded minimal to no desired product in the β,β–coupling.

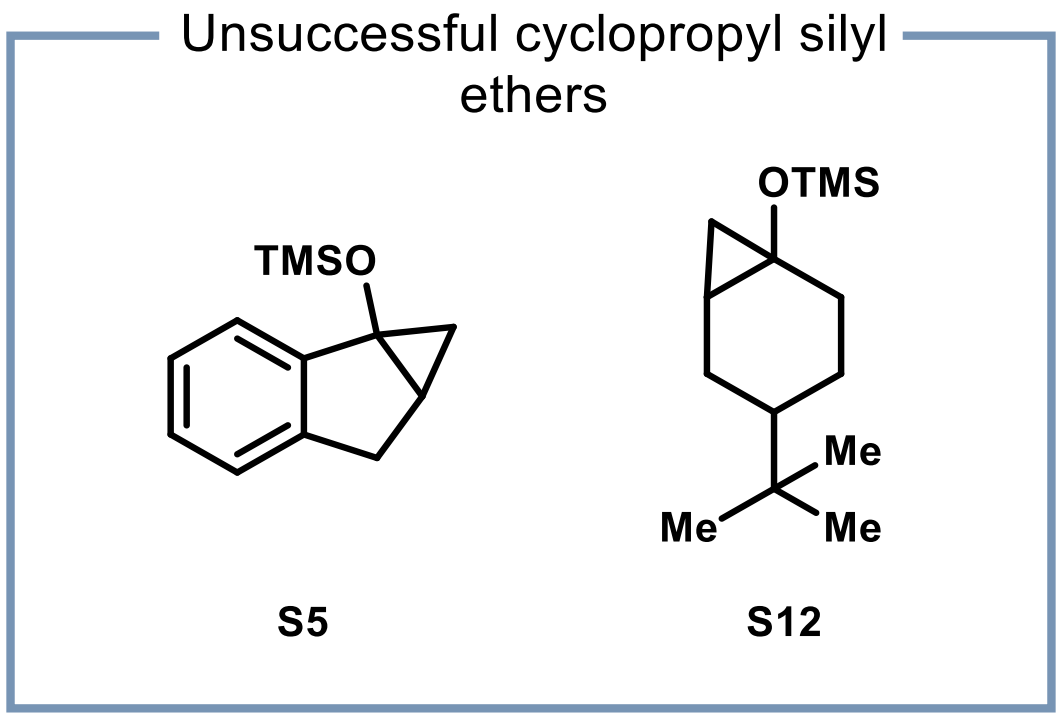

Figure S6. The unsuccessful cyclopropyl silyl ethers that yielded minimal to no desired product in the radical-polar crossover reaction.

Deuterium Labeling and TEMPO Trapping

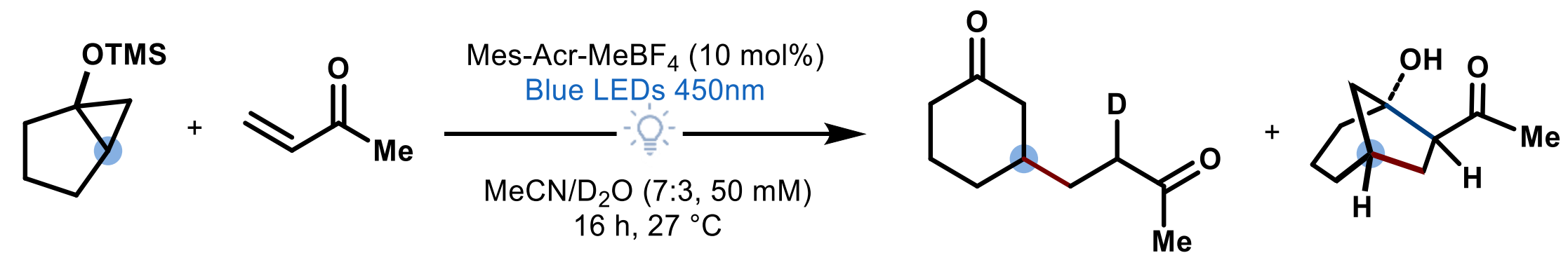

1, 1.5 equiv. **MVK** **43**, 16% **44**, 73%

β–β coupled product 43 and **Radical-polar crossover product 44.** Compounds **43** and **44** were prepared from cyclopropyl silyl ether **1** (0.23 mmol) and methyl vinyl ketone (0.15 mmol) according to *Method B1* (D_2O instead of HFIP). Purification by flash column chromatography (silica gel, 10% to 60% EtOAc in PE) afforded **43** (4.1 mg, 0.024 mmol, 16%) and **44** (18.5 mg, 0.110 mmol, 73%) as a faint yellow oil and a faint yellow viscous oil, respectively.

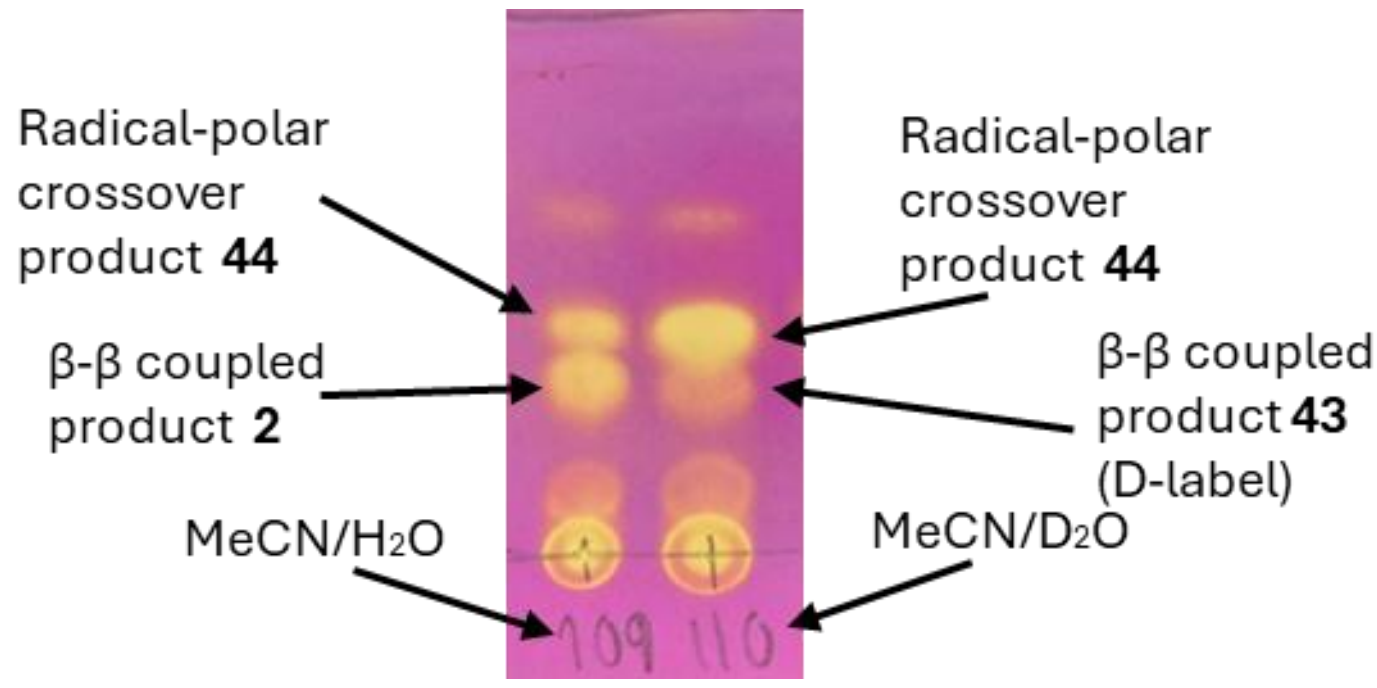

Figure S7. TLC analysis demonstrating the influence of co-solvent identity on product distribution. Use of MeCN/H_2O (left) favors formation of β–β coupled product **2** (more polar), whereas MeCN/D_2O (right) leads to preferential formation of radical-polar crossover product **44** (less polar).

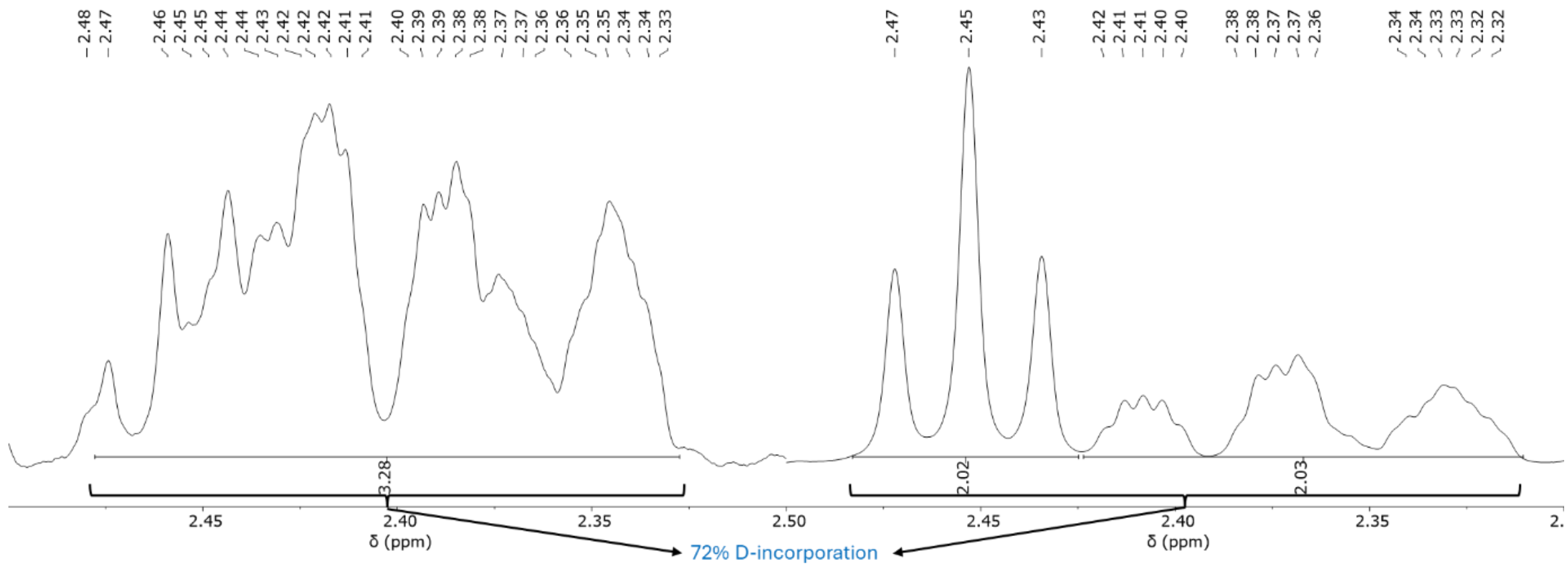

Figure S8. Comparison of expanded ^{1}H NMR spectra of D-labeled compound **43** (left, MeCN/D_2O) and compound **2** (right, MeCN/HFIP), respectively. Attenuation of the α-proton signal in the MeCN/D_2O sample indicates major deuterium incorporation at the α-position.

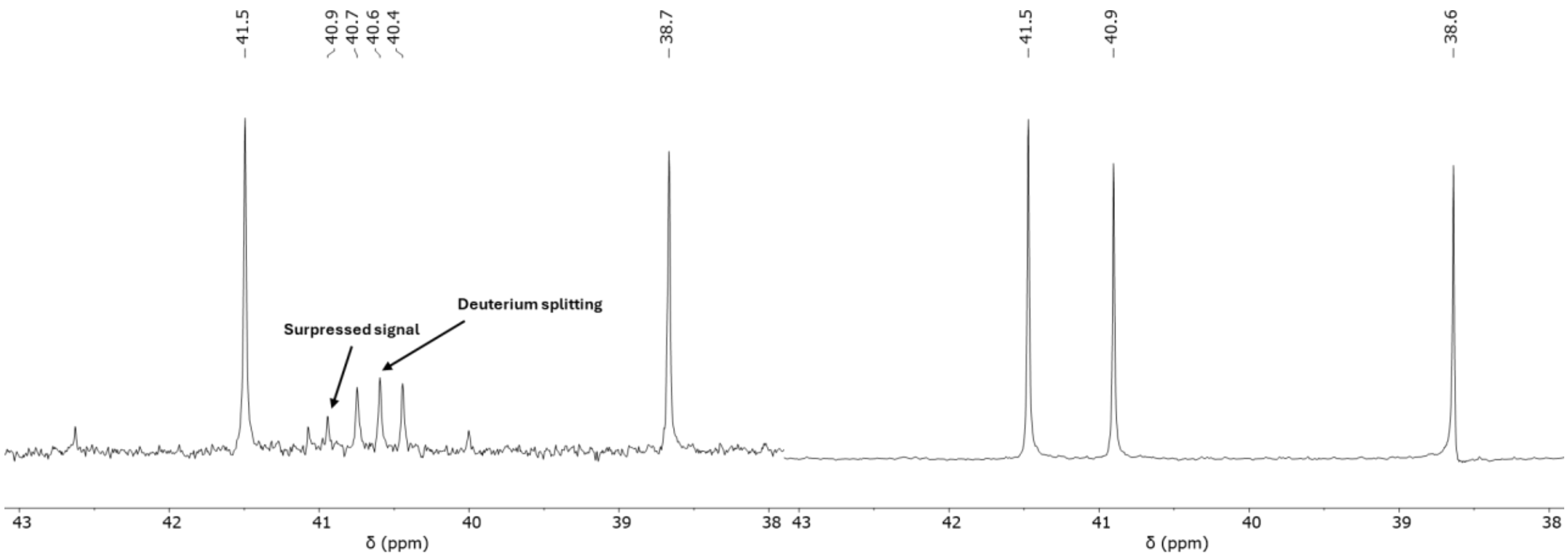

Figure S9. Comparison of expanded ^{13}C NMR spectra of D-labeled compound **43** (left, MeCN/D_2O) and compound **2** (right, MeCN/HFIP), respectively. Splitting of the carbon signal in the MeCN/D_2O sample indicates major deuterium incorporation at the α-position.

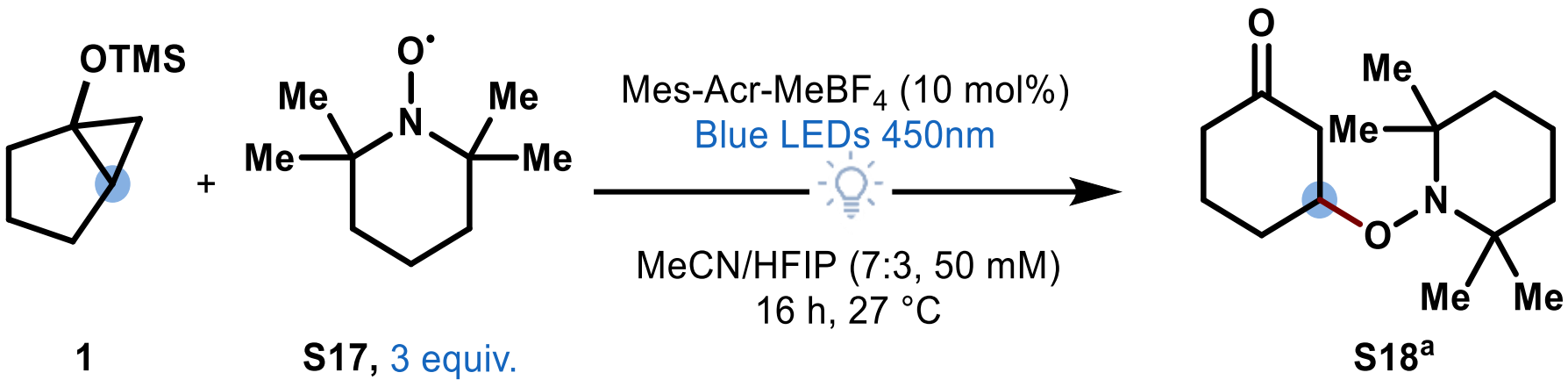

Scheme S1. TEMPO trapping of the β-keto radical. [a] Compound **S18** could not be isolated in meaningful quantities and was only observed as the H^+-adduct by LC-MS in the crude reaction mixture.

Method B1 (for Volatile Alkenes)

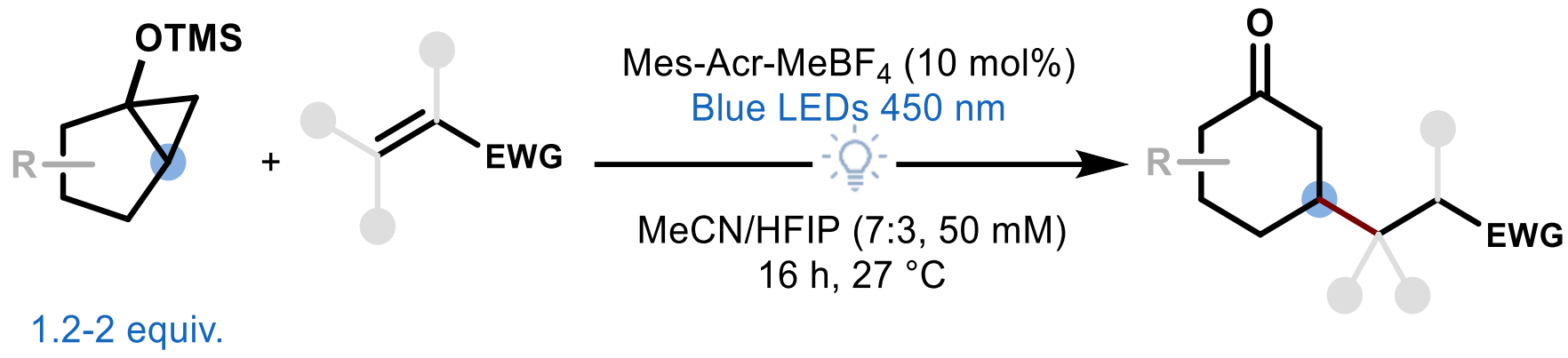

1.2-2 equiv.

Cyclopropyl silyl ether (1.2-2.0 equiv.) and Mes-Acr-MeBF$_4$ (10 mol%) were dissolved in MeCN/HFIP (7:3, 50 mM). The reaction mixture was degassed for 5 min, after which the electron-deficient alkene (1.0 equiv.) was added. The resulting solution was irradiated with 450 nm LEDs for 16 h. Upon completion, the reaction mixture was concentrated under reduced pressure, and the residue was purified by flash column chromatography.

Method B2 (for Non-volatile Alkenes)

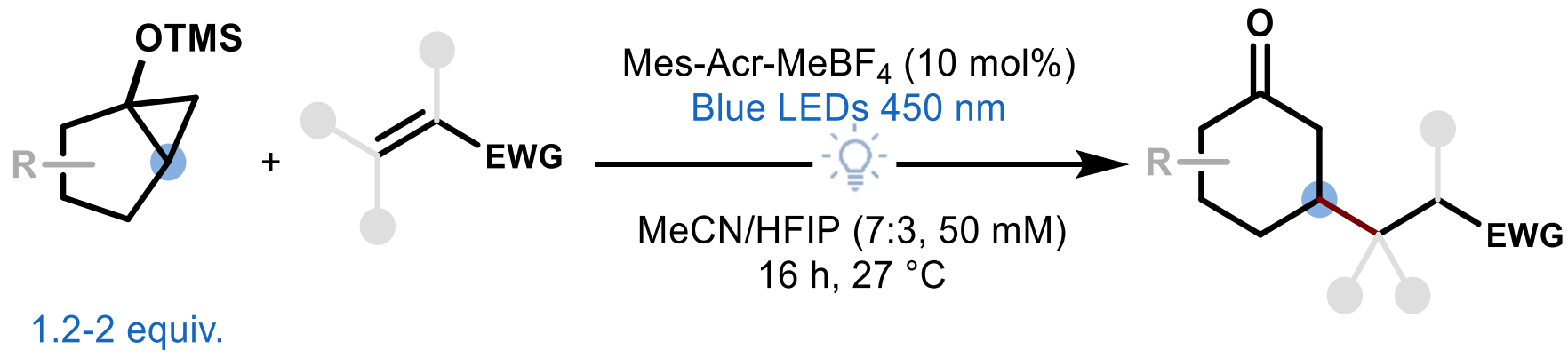

1.2-2 equiv.

Cyclopropyl silyl ether (1.2–2.0 equiv.), Mes-Acr-MeBF$_4$ (10 mol%), and the electron-deficient alkene (1.0 equiv.) were dissolved in MeCN/HFIP (7:3, 50 mM). The resulting solution was degassed for 5 min and then irradiated with 450 nm LEDs for 16 h. Upon completion, the reaction mixture was concentrated under reduced pressure, and the crude residue was purified by flash column chromatography.

Method C1 (for Volatile Alkenes)

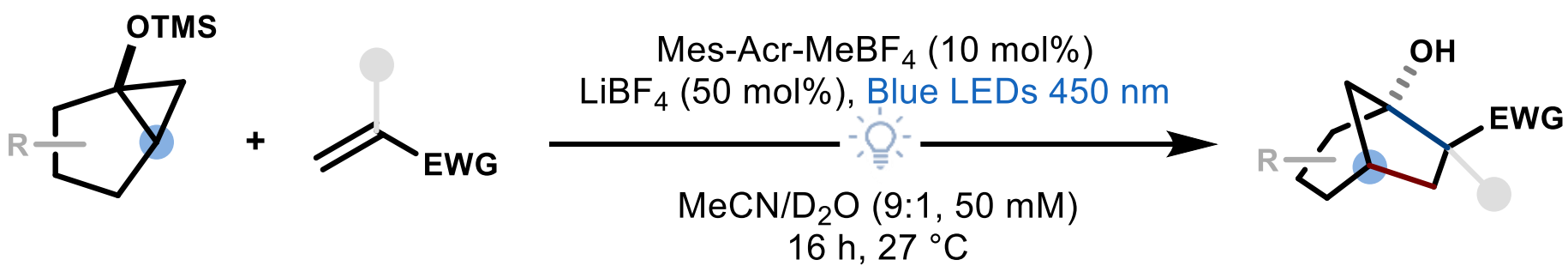

1.5-2 equiv.

Cyclopropyl silyl ether (1.5-2.0 equiv.), Mes-Acr-MeBF$_4$ (10 mol%), and LiBF$_4$ (50 mol%) were dissolved in MeCN/D_2O (9:1, 50 mM). The reaction mixture was degassed for 5 min, after which the electron-deficient alkene (1.0 equiv.) was added. The resulting solution was irradiated with 450 nm LEDs for 16 h. Upon completion, the reaction mixture was concentrated under reduced pressure, and the residue was purified by flash column chromatography.

Method C2 (for Non-volatile Alkenes)

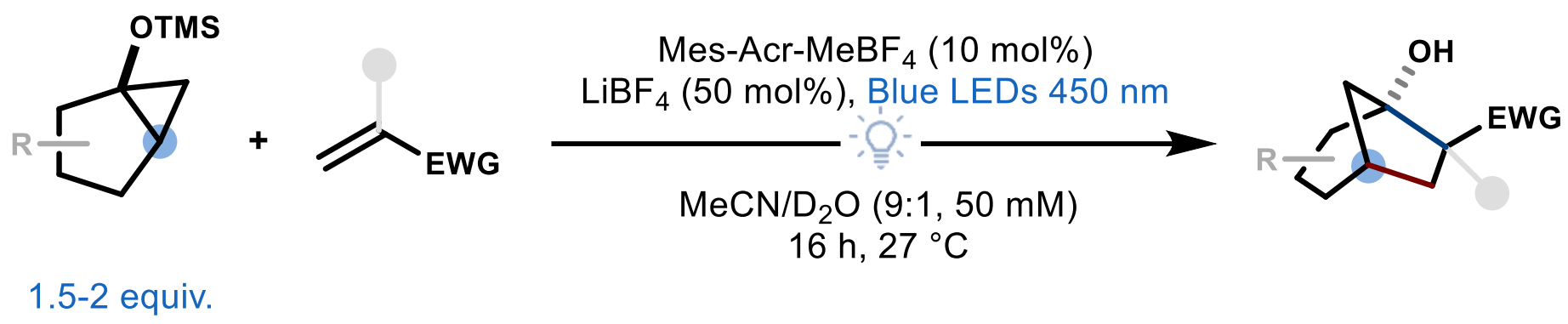

Cyclopropyl silyl ether (1.5–2.0 equiv.), Mes-Acr-MeBF_4 (10 mol%), LiBF_4 (50 mol%), and the electron-deficient alkene (1.0 equiv.) were dissolved in MeCN/D_2O (9:1, 50 mM). The resulting solution was degassed for 5 min and then irradiated with 450 nm LEDs for 16 h. Upon completion, the reaction mixture was concentrated under reduced pressure, and the crude residue was purified by flash column chromatography.

Synthesis and Characterization of β–β coupled Products

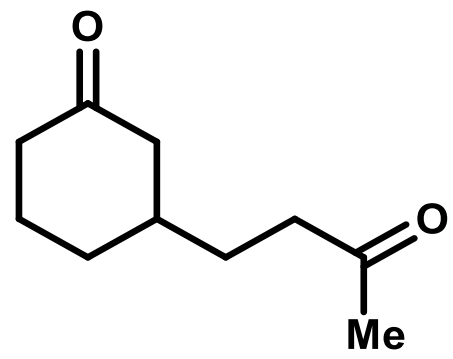

2

β–β coupled product 2. Compound **2** was prepared from cyclopropyl silyl ether **1** (0.15 mmol) and methyl vinyl ketone (0.10 mmol) according to *Method B1*. Purification by flash column chromatography (silica gel, 10% to 100% EtOAc in PE) afforded **2** (15.6 mg, 0.093 mmol, 93%) as a faint yellow oil.

R_f 0.33 (silica gel, 40% EtOAc in PE)

^{1}H NMR (400 MHz, $CDCl_3$) δ 2.45 (t, *J* = 7.6 Hz, 2H), 2.42 – 2.31 (m, 2H), 2.30 – 2.19 (m, 1H), 2.14 (s, 3H), 2.08 – 1.95 (m, 2H), 1.92 – 1.84 (m, 1H), 1.80 – 1.51 (m, 4H), 1.39 – 1.27 (m, 1H).

^{13}C NMR (101 MHz, $CDCl_3$) δ 211.5, 208.5, 48.0, 41.5, 40.9, 38.6, 31.3, 30.2, 30.1, 25.2.

HRMS (ESI−) *m/z*: calcd. for $C_{10}H_{15}O_2^-$ [M-H]$^-$: 167.1078; found: 167.1078, (Δ = 0 ppm)

IR (ATR)/cm^{-1} 2931, 2864, 1705, 1419, 1358, 1226, 1160

*Under otherwise identical conditions, compound **2** was prepared on a 1.0 mmol scale in 89% yield (150 mg, 0.89 mmol).*

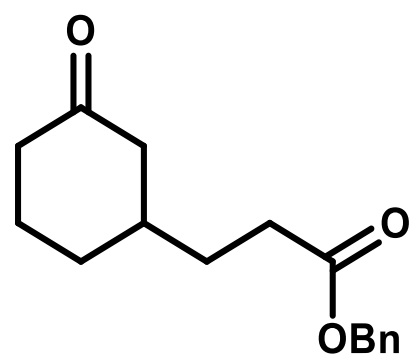

6

β–β coupled product 6. Compound **6** was prepared from cyclopropyl silyl ether **1** (0.12 mmol) and benzyl acrylate (0.10 mmol) according to *Method B1*. Purification by flash column chromatography (silica gel, 10% to 100 % EtOAc in PE) afforded **6** (21.6 mg, 0.083 mmol, 83%) as a faint yellow oil.

R_f 0.31 (silica gel, 40% EtOAc in PE)

^{1}H NMR (400 MHz, $CDCl_3$) δ 7.44 – 7.27 (m, 5H), 5.11 (s, 2H), 2.44 – 2.33 (m, 4H), 2.30 – 2.20 (m, 1H), 2.08 – 1.96 (m, 2H), 1.92 – 1.85 (m, 1H), 1.81 – 1.59 (m, 4H), 1.39 – 1.27 (m, 1H).

^{13}C NMR (101 MHz, $CDCl_3$) δ 211.4, 173.3, 136.0, 128.7, 128.4, 66.5, 47.9, 41.5, 38.6, 31.7, 31.6, 31.1, 29.9, 25.2.

HRMS (ESI−) *m*/z: calcd. for $C_{16}H_{19}O_3^-$ [M-H]$^-$: 259.1340; found: 259.1342, (Δ = 0.77 ppm)

IR (ATR)/cm^{-1} 2927, 2864, 1731, 1708, 1454, 1149, 1102, 965, 748, 697

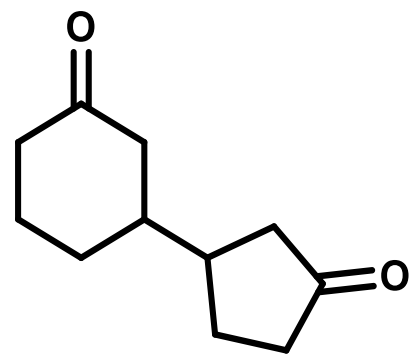

7

β–β coupled product 7. Compound **7** was prepared from cyclopropyl silyl ether **1** (0.15 mmol) and cyclopent-2-en-1-one (0.10 mmol) according to *Method B2*. Purification by flash column chromatography (silica gel, 10% to 100% EtOAc in PE) afforded **7** (12.4 mg, 0.069 mmol, 69%, ca. 1:1 dr) as a faint yellow oil.

R_f 0.38 (silica gel, 40% EtOAc in PE)

^{1}H NMR (400 MHz, $CDCl_3$) δ 2.56 (ddt, *J* = 13.8, 4.0, 2.0 Hz, 0.5H), 2.49 – 1.97 (m, 10H), 1.94 – 1.62 (m, 3.5H), 1.58 – 1.36 (m, 2H). (Summary of diastereomers)

^{13}C NMR (126 MHz, $CDCl_3$) δ 218.4, 218.3, 211.1, 211.0, 47.0, 46.2, 44.8, 44.7, 43.6, 43.2, 42.9 (two overlapping signals), 41.50, 41.47, 38.92, 38.89, 30.3, 29.2, 27.7, 27.6, 25.20, 25.19. (Summary of diastereomers)

HRMS (ESI−) *m*/z: calcd. for $C_{11}H_{15}O_2^-$ [M-H]$^-$: 179.1078; found: 179.1079, (Δ = 0.56 ppm)

IR (ATR)/cm^{-1} 2955, 2934, 2865, 1736, 1705, 1404, 1315, 1227, 1165, 1099

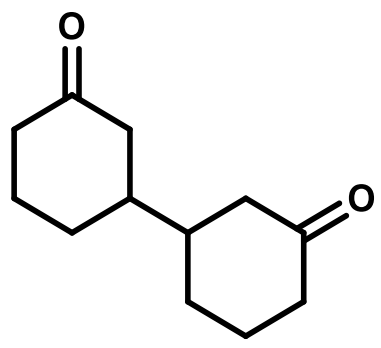

8

β–β coupled product 8. Compound **8** was prepared from cyclopropyl silyl ether **1** (0.60 mmol) and cyclohex-2-en-1-one (0.30 mmol) according to *Method B2* (24 h, 40 °C). Purification by flash column chromatography (silica gel, 10% to 60% EtOAc in PE) afforded **8** (38.5 mg, 0.198 mmol, 66%, ca. 1:1 dr) as a faint yellow solid and recovered cyclohex-2-en-1-one (7.8 mg, 0.081 mmol, 27%).

R_f 0.36 (silica gel, 40% EtOAc in PE)

^{1}H NMR (400 MHz, $CDCl_3$) δ 2.49 – 2.32 (m, 4H), 2.32 – 2.18 (m, 2H), 2.16 – 2.01 (m, 4H), 1.96 – 1.85 (m, 2H), 1.80 – 1.53 (m, 4H), 1.47 – 1.31 (m, 2H). (Summary of diastereomers)

^{13}C NMR (126 MHz, $CDCl_3$) δ 211.42, 211.39, 45.5, 45.0, 43.93, 43.86, 41.5 (two overlapping signals), 28.6, 28.4, 25.4, 25.3. (Summary of diastereomers)

HRMS (ESI+) *m*/z: calcd. for $C_{12}H_{19}O_2^+$ [M+H]$^+$: 195.1380; found: 195.1376, (Δ = −2.05 ppm)

IR (ATR)/cm^{-1} 2933, 2862, 1703, 1448, 1422, 1346, 1317, 1274, 1225, 1167

MP 55 °C

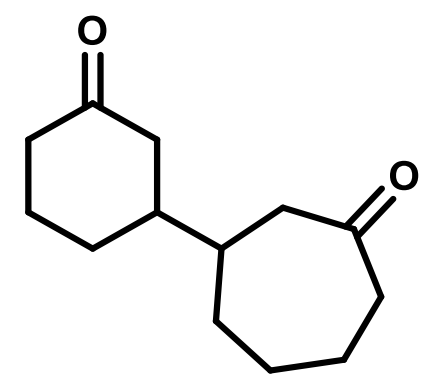

9

β–β coupled product 9. Compound **9** was prepared from cyclopropyl silyl ether **1** (0.60 mmol) and cyclohept-2-en-1-one (0.30 mmol) according to *Method B2* (24 h, 40 °C). Purification by flash column chromatography (silica gel, 10% to 60% EtOAc in PE) afforded **9** (36.9 mg, 0.177 mmol, 59%, ca. 1:1 dr) as a yellow viscous oil and recovered cyclohept-2-en-1-one (9.6 mg, 0.087 mmol, 29%).

R_f 0.42 (silica gel, 40% EtOAc in PE)

^{1}H NMR (400 MHz, $CDCl_3$) δ 2.53 – 2.27 (m, 6H), 2.26 – 2.14 (m, 1H), 2.13 – 2.01 (m, 2H), 1.98 – 1.71 (m, 5H), 1.70 – 1.51 (m, 3H), 1.50 – 1.28 (m, 3H). (Summary of diastereomers)

^{13}C NMR (126 MHz, $CDCl_3$) δ 214.2, 214.1, 211.5, 47.1, 46.9, 45.2, 45.1, 44.8, 44.6, 43.8, 41.5, 40.62, 40.59, 33.9, 33.8, 29.0, 28.6, 28.2, 28.0, 25.4, 24.6, 24.5. (Summary of diastereomers, multiple overlapping signals)

HRMS (ESI+) *m*/z: calcd. for $C_{13}H_{20}NaO_2^+$ $[M+Na]^+$: 231.1356; found: 231.1351, (Δ = −2.16 ppm)

IR (ATR)/cm^{-1} 2925, 2858, 1697, 1447, 1346, 1275, 1261, 1226, 1185

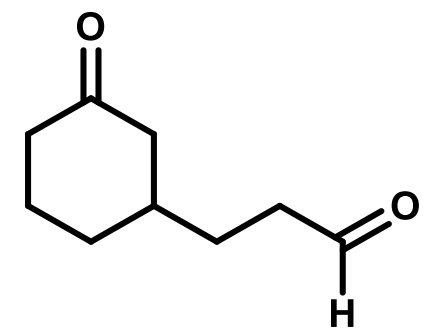

10

β–β coupled product 10. Compound **10** was prepared from cyclopropyl silyl ether **1** (0.18 mmol) and acrolein (0.15 mmol) according to *Method B1*. Purification by flash column chromatography (silica gel, 10% to 100% EtOAc in PE) afforded **10** (18.0 mg, 0.117 mmol, 78%) as a transparent oil.

R_f 0.29 (silica gel, 40% EtOAc in PE)

^{1}H NMR (400 MHz, $CDCl_3$) δ 9.77 (t, *J* = 1.5 Hz, 1H), 2.47 (td, *J* = 7.5, 1.5 Hz, 2H), 2.46 – 2.36 (m, 1H), 2.40 – 2.30 (m, 1H), 2.31 – 2.18 (m, 1H), 2.09 – 1.97 (m, 2H), 1.95 – 1.84 (m, 1H), 1.85 – 1.71 (m, 1H), 1.73 – 1.57 (m, 3H), 1.41 – 1.27 (m, 1H).

^{13}C NMR (101 MHz, $CDCl_3$) δ 211.2, 201.8, 47.9, 41.4, 41.3, 38.6, 31.2, 28.5, 25.2.

HRMS (ESI−) *m*/z: calcd. for $C_9H_{13}O_2^-$ $[M-H]^-$: 153.0921; found: 153.0922, (Δ = 0.65 ppm)

IR (ATR)/cm^{-1} 2932, 2867, 1702, 1450, 1418, 1226, 1155, 1102, 1059, 947, 866

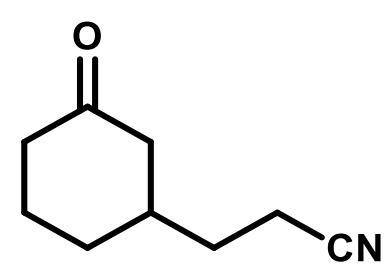

11

β–β coupled product 11. Compound **11** was prepared from cyclopropyl silyl ether **1** (0.18 mmol) and acrylonitrile (0.15 mmol) according to *Method B1* (using Mes-Acr-$PhBF_4$). Purification by flash column

chromatography (silica gel, 10% to 100% EtOAc in PE) afforded **11** (17.7 mg, 0.117 mmol, 78%) as a ruby red oil.

R_f 0.25 (silica gel, 40% EtOAc in PE)

^{1}H NMR (500 MHz, $CDCl_3$) δ 2.50 – 2.42 (m, 1H), 2.39 (td, *J* = 7.3, 2.5 Hz, 3H), 2.33 – 2.22 (m, 1H), 2.11 – 2.00 (m, 2H), 2.01 – 1.92 (m, 2H), 1.78 – 1.64 (m, 3H), 1.39 (dtd, *J* = 14.5, 11.3, 3.7 Hz, 1H).

^{13}C NMR (126 MHz, $CDCl_3$) δ 210.1, 119.3, 47.3, 41.3, 37.9, 31.8, 30.6, 24.9, 14.9.

HRMS (ESI−) *m*/z: calcd. for $C_9H_{12}NO^-$ [M-H]$^-$: 150.0924; found: 150.0926, (Δ = 1.33 ppm)

IR (ATR)/cm^{-1} 2930, 2866, 2245, 1708, 1450, 1424, 1228

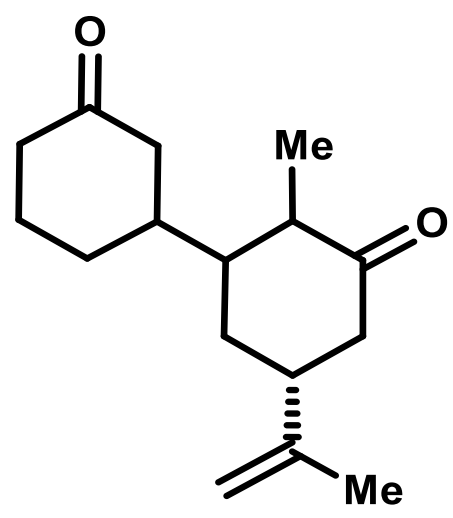

12

β–β coupled product 12. Compound **12** was prepared from cyclopropyl silyl ether **1** (0.15 mmol) and (*R*)-(−)-carvone (0.10 mmol) according to *Method B2*. Purification by flash column chromatography (silica gel, 10% to 100% EtOAc in PE) afforded **12** (15.9 mg, 0.064 mmol, 64%, as a mixture of diastereomers) as a faint yellow oil.

R_f 0.41 (silica gel, 40% EtOAc in PE)

^{1}H NMR (400 MHz, $CDCl_3$) δ 4.92 – 4.67 (m, 2H), 2.76 – 2.66 (m, 1H), 2.66 – 2.42 (m, 3H), 2.44 – 2.32 (m, 2H), 2.34 – 2.19 (m, 2H), 2.15 – 1.89 (m, 4H), 1.81 – 1.69 (m, 4H), 1.70 – 1.56 (m, 2H), 1.35 – 1.21 (m, 1H), 1.12 – 1.02 (m, 3H). (Summary of diastereomers)

^{13}C NMR (126 MHz, $CDCl_3$) δ 214.9, 214.7, 214.6, 214.5, 213.1, 212.8, 211.4, 211.3, 211.2, 211.1, 147.4, 147.3, 146.5, 146.4, 146.2, 112.6, 112.50, 112.47, 112.3, 110.28, 110.27, 47.5, 47.2, 46.9, 46.54, 46.52, 46.46, 46.23, 46.18, 46.0, 45.80, 45.7, 45.6, 45.4, 44.9, 44.8, 44.04, 44.02, 43.9, 43.6, 42.9, 42.2, 41.9, 41.61, 41.56, 41.49, 41.46, 41.4, 41.2, 40.7, 40.5, 40.42, 40.41, 40.3, 40.00, 39.95, 39.6, 39.54, 39.48, 39.30, 30.26, 29.7, 29.6, 28.6, 28.5, 28.0, 27.2, 27.1, 26.4, 25.5, 25.40, 25.32, 25.29, 25.2, 25.1, 22.8, 22.0, 21.9, 21.7, 20.6, 20.5, 20.4, 15.8, 13.3, 13.0, 11.8, 11.7, 11.3, 11.2. (Summary of diastereomers, multiple overlapping signals)

HRMS (ESI−) *m*/z: calcd. for $C_{16}H_{23}O_2^-$ [M-H]$^-$: 247.1704; found: 247.1703, (Δ = −0.40 ppm)

IR (ATR)/cm^{-1} 2962, 2937, 2870, 1705, 1448, 1427, 1230, 897

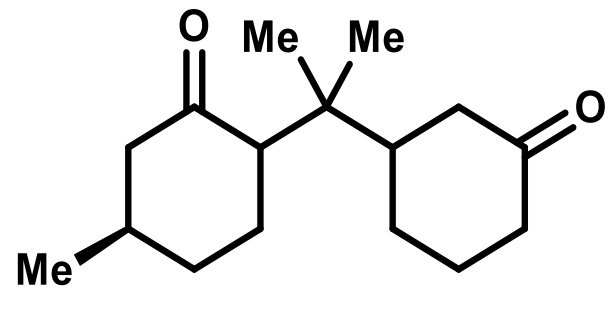

13

β–β coupled product 13. Compound **13** was prepared from cyclopropyl silyl ether **1** (0.20 mmol) and (*R*)-(+)-pulegone (0.10 mmol) according to *Method B2* (24 h, 40 °C). Purification by flash column chromatography (silica gel, 10% to 100% EtOAc in PE) afforded **13** (8.8 mg, 0.035 mmol, 35%, as a mixture of diastereomers) as a faint yellow oil.

R_f 0.49 (silica gel, 40% EtOAc in PE)

^{1}H NMR (400 MHz, $CDCl_3$) δ 2.40 – 2.17 (m, 5H), 2.14 – 1.97 (m, 4H), 1.91 – 1.65 (m, 4H), 1.52 – 1.25 (m, 4H), 1.13 – 0.76 (m, 9H). (Summary of diastereomers)

^{13}C NMR (126 MHz, $CDCl_3$) δ 212.8, 212.7, 212.0, 211.9, 55.8, 55.7, 52.8, 52.7, 44.5, 44.3, 43.2, 43.19, 43.17, 43.15, 41.5, 36.9, 36.7, 35.0, 35.0, 28.4, 28.3, 25.74, 25.72, 25.68, 25.5, 25.4, 22.5, 20.9, 20.7, 20.6, 20.2. (Summary of diastereomers, multiple overlapping signals)

HRMS (ESI−) *m*/*z*: calcd. for $C_{17}H_{27}O_4^-$ [M+HCOO]$^-$: 295.1915; found: 295.1915, (Δ = 0 ppm)

IR (ATR)/cm^{-1} 2952, 2927, 2868, 1707, 1455, 1366, 1229

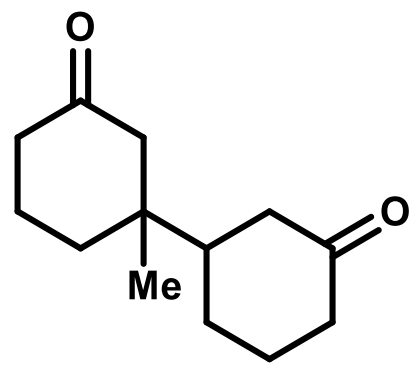

14

β–β coupled product 14. Compound **14** was prepared from cyclopropyl silyl ether **S3** (0.60 mmol) and cyclohex-2-en-1-one (0.30 mmol) according to *Method B2* (24 h, 40 °C). Purification by flash column chromatography (silica gel, 10% to 50% EtOAc in PE) afforded **14** (19.4 mg, 0.093 mmol, 31%, 5.5:1 dr) as a reddish oil and recovered cyclohex-2-en-1-one (14.4 mg, 0.150 mmol, 50%).

R_f 0.48 (silica gel, 40% EtOAc in PE)

^{1}H NMR (400 MHz, $CDCl_3$) δ 2.44 – 2.35 (m, 2H), 2.34 – 2.19 (m, 4H), 2.15 – 2.04 (m, 2H), 1.97 – 1.79 (m, 3H), 1.67 – 1.52 (m, 4H), 1.41 – 1.23 (m, 2H), 0.92 – 0.85 (m, 3H). (Summary of diastereomers)

^{13}C NMR (101 MHz, $CDCl_3$) δ 212.0, 211.9, 52.0, 47.5, 42.8, 41.4, 41.1 (two overlapping signals), 34.2, 25.4, 25.2, 21.8, 20.5. (major)

HRMS (ESI+) *m*/*z*: calcd. for $C_{13}H_{21}O_2^+$ [M+H]$^+$: 209.1536; found: 209.1535, (Δ = −0.48 ppm)

IR (ATR)/cm^{-1} 2956, 2939, 2871, 1705, 1458, 1423, 1317, 1276, 1261, 1229

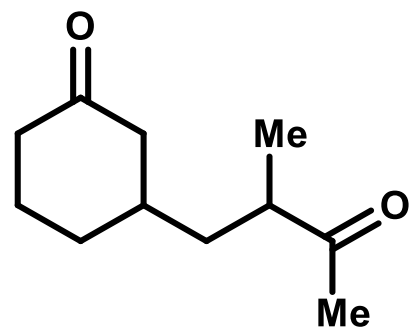

15

β–β coupled product 15. Compound **15** was prepared from cyclopropyl silyl ether **1** (0.12 mmol) and 3-methylbut-3-en-2-one (0.10 mmol) according to *Method B1*. Purification by flash column chromatography (silica gel, 10% to 100% EtOAc in PE) afforded **15** (16.8 mg, 0.092 mmol, 92%, 1:1 dr) as a faint yellow oil.

R_f 0.36 (silica gel, 40% EtOAc in PE)

^{1}H NMR (400 MHz, $CDCl_3$) δ 2.66 – 2.51 (m, 1H), 2.41 – 2.30 (m, 2H), 2.29 – 2.18 (m, 1H), 2.13 (d, *J* = 4.7 Hz, 3H), 2.07 – 1.94 (m, 2H), 1.91 – 1.83 (m, 1H), 1.82 – 1.57 (m, 3H), 1.37 – 1.19 (m, 2H), 1.08 (d, *J* = 7.0 Hz, 3H). (Summary of diastereomers)

^{13}C NMR (101 MHz, $CDCl_3$) δ 212.22, 212.20, 211.4, 211.3, 48.3, 47.9, 44.3 (two overlapping signals), 41.48, 41.46, 39.4, 39.3, 36.9, 36.7, 31.7, 31.3, 28.4, 28.0, 25.22, 25.15, 17.1, 16.7. (Summary of diastereomers)

HRMS (ESI−) *m*/*z*: calcd. for $C_{11}H_{17}O_2^-$ [M-H]$^-$: 181.1234; found: 181.1235, (Δ = 0.55 ppm)

IR (ATR)/cm^{-1} 2930, 2873, 1704, 1449, 1422, 1355, 1227, 1166, 955

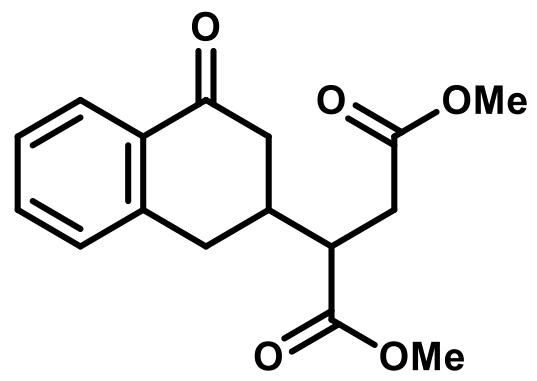

16

β–β coupled product 16. Compound **16** was prepared from cyclopropyl silyl ether **S5** (0.15 mmol) and dimethyl maleate (0.10 mmol) according to *Method B2*. Purification by flash column chromatography (silica gel, 10% to 60% EtOAc in PE) afforded **16** (13.9 mg, 0.048 mmol, 48%, ca. 1:1 dr) as a brown thick oil.

R_f 0.19 (silica gel, 30% EtOAc in PE)

^{1}H NMR (400 MHz, $CDCl_3$) δ 8.02 (d, *J* = 7.8 Hz, 1H), 7.49 (t, *J* = 7.5 Hz, 1H), 7.33 (t, *J* = 7.6 Hz, 1H), 7.24 (app. s, 1H), 3.74 (s, 3H), 3.69 (s, 3H), 3.04 – 2.83 (m, 4H), 2.80 – 2.70 (m, 1H), 2.58 – 2.49 (m, 2H), 2.48 – 2.38 (m, 1H). (Summary of diastereomers)

^{13}C NMR (101 MHz, $CDCl_3$) δ 197.0, 196.9, 173.7, 172.2, 142.71, 142.67, 134.01, 133.99, 132.1, 129.1, 129.0, 127.4, 127.3, 127.24, 127.22, 52.22, 52.20, 52.17, 45.61, 45.58, 43.1, 42.6, 37.1, 37.0, 33.8, 33.6, 33.5. (Summary of diastereomers, multiple overlapping signals)

HRMS (ESI+) *m*/*z*: calcd. for $C_{16}H_{18}NaO_5^+$ [M+Na]$^+$: 313.1046; found: 313.1048, (Δ = 0.64 ppm)

IR (ATR)/cm^{-1} 3004, 2952, 2921, 2851, 1729, 1681, 1600, 1436, 1290, 1261, 1193, 1163, 1022, 848, 798, 764

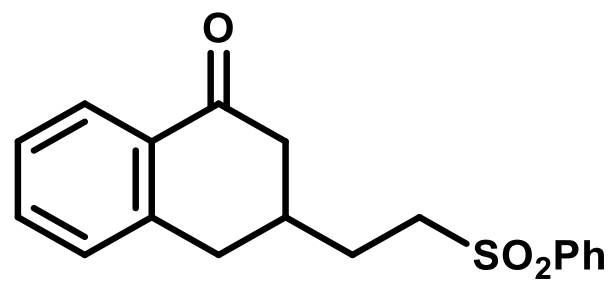

17

β–β coupled product 17. Compound **17** was prepared from cyclopropyl silyl ether **S5** (0.15 mmol) and phenyl vinyl sulfone (0.10 mmol) according to *Method B2*. Purification by flash column chromatography (silica gel, 10% to 40% EtOAc in PE) afforded **17** (20.4 mg, 0.065 mmol, 65%) as a white solid.

R_f 0.35 (silica gel, 40% EtOAc in PE)

^{1}H NMR (400 MHz, $CDCl_3$) δ 8.02 (d, *J* = 7.8 Hz, 1H), 7.98 – 7.92 (m, 2H), 7.74 – 7.68 (m, 1H), 7.66 – 7.59 (m, 2H), 7.51 (td, *J* = 7.5, 1.5 Hz, 1H), 7.34 (t, *J* = 7.5 Hz, 1H), 7.25 (d, *J* = 7.7 Hz, 1H), 3.20 (t, *J* = 7.7 Hz, 2H), 3.06 – 2.98 (m, 1H), 2.80 – 2.70 (m, 2H), 2.40 – 2.27 (m, 2H), 2.02 – 1.86 (m, 2H).

^{13}C NMR (126 MHz, $CDCl_3$) δ 197.0, 142.5, 139.1, 134.1, 134.0, 132.2, 129.6, 129.0, 128.2, 127.3, 127.2, 53.9, 44.8, 35.8, 34.4, 28.3.

HRMS (ESI+) *m*/*z*: calcd. for $C_{18}H_{22}NO_3S^+$ [M+NH$_4$]$^+$: 332.1315; found: 332.1312, (Δ = −0.90 ppm)

IR (ATR)/cm^{-1} 3064, 2924, 2852, 1681, 1601, 1447, 1290, 1148, 1087, 765, 739

MP 150-155 °C

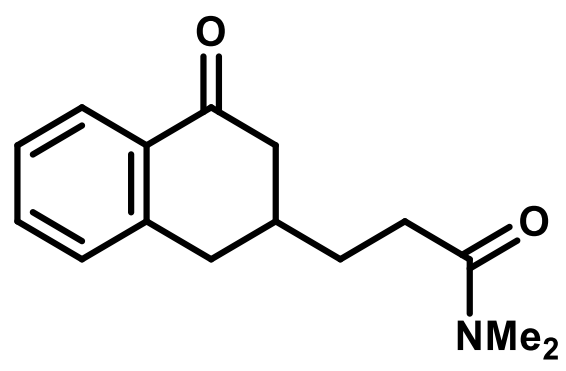

18

β–β coupled product 18. Compound **18** was prepared from cyclopropyl silyl ether **S5** (0.23 mmol) and *N,N*-dimethyl acrylamide (0.15 mmol) according to *Method B2*. Purification by flash column chromatography (silica gel, 10% to 100% EtOAc in PE) afforded **18** (30.2 mg, 0.123 mmol, 82%) as a faint yellow oil.

R$_f$ 0.15 (silica gel, 67% EtOAc in PE)

^{1}H NMR (400 MHz, $CDCl_3$) δ 8.00 (d, *J* = 7.7 Hz, 1H), 7.47 (td, *J* = 7.5, 1.3 Hz, 1H), 7.30 (t, *J* = 7.5 Hz, 1H), 7.25 (app. s, 1H), 3.09 – 3.03 (m, 1H), 3.01 (s, 3H), 2.94 (s, 3H), 2.82 – 2.71 (m, 2H), 2.44 – 2.32 (m, 3H), 2.31 – 2.20 (m, 1H), 1.94 – 1.82 (m, 1H), 1.82 – 1.72 (m, 1H).

^{13}C NMR (101 MHz, $CDCl_3$) δ 198.2, 172.3, 143.6, 133.7, 132.4, 129.1, 127.1, 126.8, 45.2, 37.3, 36.4, 35.6, 35.2, 30.9, 30.4.

HRMS (ESI+) *m*/*z*: calcd. for $C_{15}H_{20}NO_2^+$ [M+H]$^+$: 246.1489; found: 246.1490, (Δ = 0.41 ppm)

IR (ATR)/cm^{-1} 2928, 1679, 1637, 1601, 1455, 1399, 1289, 1145, 1059, 765

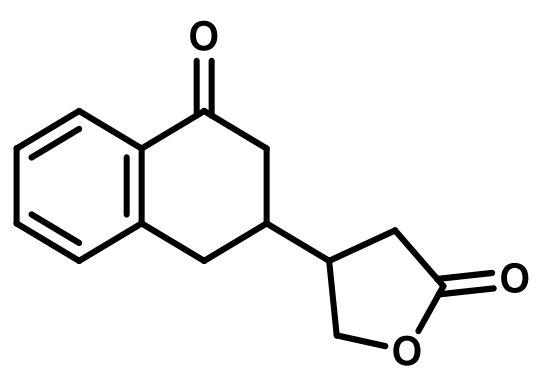

19

β–β coupled product 19. Compound **19** was prepared from cyclopropyl silyl ether **S5** (0.15 mmol) and 2(5H)-furanone (0.10 mmol) according to *Method B2*. Purification by flash column chromatography (silica gel, 1% to 10% MeCN in a 1:1 mixture of PE/DCM) afforded **19** (13.1 mg, 0.057 mmol, 57%, ca. 1:1 dr) as a white solid.

R$_f$ 0.19 (silica gel, 30% EtOAc in PE)

^{1}H NMR (400 MHz, $CDCl_3$) δ 8.06 (dt, *J* = 7.9, 2.0 Hz, 1H), 7.55 (tt, *J* = 7.5, 1.7 Hz, 1H), 7.38 (t, *J* = 7.6 Hz, 1H), 7.29 (t, *J* = 7.2 Hz, 1H), 4.59 – 4.52 (m, 1H), 4.17 – 4.06 (m, 1H), 3.10 – 2.95 (m, 1H), 2.90 – 2.75 (m, 2H), 2.75 – 2.61 (m, 2H), 2.50 – 2.41 (m, 1H), 2.41 – 2.27 (m, 2H). (Summary of diastereomers)

^{13}C NMR (126 MHz, $CDCl_3$) δ 196.4, 196.3, 176.2, 176.1, 142.0, 141.9, 134.3, 134.2, 132.2, 132.1, 129.1, 129.0, 127.50, 127.48, 127.42, 127.40, 71.5, 71.3, 43.6, 43.0, 40.4 (two overlapping signals), 39.0 (two overlapping signals), 34.4, 33.7, 32.7, 32.6. (Summary of diastereomers)

HRMS (ESI+) *m*/*z*: calcd. for $C_{14}H_{15}O_3^+$ [M+H]$^+$: 231.1016; found: 231.1016, (Δ = 0 ppm)

IR (ATR)/cm^{-1} 2958, 2920, 2852, 1772, 1679, 1602, 1289, 1262, 1175, 1017, 766

MP 150 °C

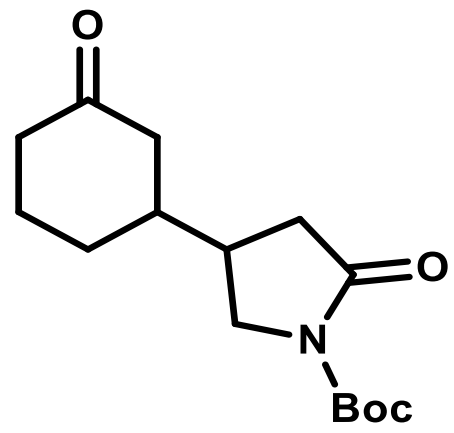

20

β–β coupled product 20. Compound **20** was prepared from cyclopropyl silyl ether **1** (0.15 mmol) and *tert*-butyl 2-oxo-2,5-dihydro-1H-pyrrole-1-carboxylate (0.10 mmol) according to *Method B2*. Purification by flash column chromatography (silica gel, 10% to 100% EtOAc in PE) afforded **20** (21.4 mg, 0.076 mmol, 76%, 1:1 dr) as a faint yellow oil.

R_f 0.50 (silica gel, 40% EtOAc in PE)

^{1}H NMR (400 MHz, $CDCl_3$) δ 3.97 – 3.81 (m, 1H), 3.39 – 3.26 (m, 1H), 2.66 – 2.49 (m, 1H), 2.46 – 2.35 (m, 2H), 2.35 – 2.14 (m, 3H), 2.13 – 2.00 (m, 2H), 1.94 – 1.85 (m, 1H), 1.79 – 1.61 (m, 2H), 1.51 (dd, *J* = 2.8, 1.0 Hz, 9H), 1.46 – 1.33 (m, 1H). (Summary of diastereomers)

^{13}C NMR (101 MHz, $CDCl_3$) δ 210.01, 209.96, 173.1, 172.9, 150.1, 149.9, 83.3, 50.3, 46.0, 45.7, 43.1, 41.30, 41.28, 37.8, 37.5, 36.54, 36.52, 29.3, 28.9, 28.13, 28.12, 25.00, 24.95. (Summary of diastereomers, multiple overlapping signals)

HRMS (ESI−) *m*/*z*: calcd. for $C_{15}H_{22}NO_4^-$ [M-H]$^-$: 280.1554; found: 280.1555, (Δ = 0.36 ppm)

IR (ATR)/cm^{-1} 2977, 2933, 2871, 1779, 1748, 1707, 1367, 1308, 1151, 1118, 1018, 853, 779, 731

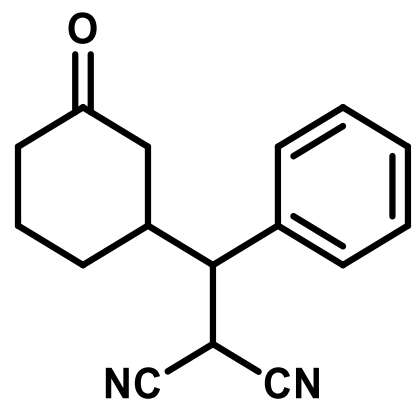

21

β–β coupled products 21a and **21b.** Compounds **21a** and **21b** were prepared from cyclopropyl silyl ether **1** (0.23 mmol) and benzylidenemalononitrile (0.15 mmol) according to *Method B2*. Purification by flash column chromatography (silica gel, 10% to 100% EtOAc in PE) afforded diastereomers **21a** (17.4 mg, 0.069 mmol, 46%) and **21b** (16.7 mg, 0.066 mmol, 44%) as faint orange viscous oils (combined yield 90%; ca. dr 1:1).

R_f 0.17 (**21a**); 0.22 (**21b**) (silica gel, 40% EtOAc in PE)

^{1}H NMR (400 MHz, $CDCl_3$) δ 7.47 – 7.37 (m, 3H), 7.31 – 7.26 (m, 2H), 4.19 (d, *J* = 5.4 Hz, 1H), 3.06 (dd, *J* = 9.7, 5.4 Hz, 1H), 2.61 – 2.49 (m, 1H), 2.49 – 2.38 (m, 1H), 2.30 – 2.23 (m, 1H), 2.23 – 2.14 (m, 3H), 1.94 – 1.75 (m, 2H), 1.59 – 1.48 (m, 1H). (**21a**)

^{1}H NMR (400 MHz, $CDCl_3$) δ 7.48 – 7.39 (m, 3H), 7.36 – 7.28 (m, 2H), 4.16 (d, *J* = 6.2 Hz, 1H), 3.05 (dd, *J* = 9.0, 6.1 Hz, 1H), 2.69 – 2.59 (m, 1H), 2.62 – 2.50 (m, 1H), 2.49 – 2.38 (m, 1H), 2.31 – 2.18 (m, 1H), 2.18 – 2.07 (m, 1H), 2.06 – 1.94 (m, 1H), 1.80 – 1.68 (m, 1H), 1.72 – 1.58 (m, 1H), 1.36 – 1.23 (m, 1H). (**21b**)

^{13}C NMR (101 MHz, $CDCl_3$) δ 208.8, 135.2, 129.7, 129.5, 128.1, 111.7 (two overlapping signals), 51.7, 45.1, 40.8, 40.3, 29.5, 27.5, 24.5. (**21a**)

^{13}C NMR (101 MHz, $CDCl_3$) δ 208.7, 135.2, 129.6, 129.4, 128.4, 111.7, 111.5, 51.2, 45.9, 41.0, 40.2, 28.2, 27.2, 24.3. (**21b**)

HRMS (ESI+) *m*/z: calcd. for $C_{16}H_{17}N_2O^+$ $[M+H]^+$: 253.1335; found: 253.1337, (Δ = 0.79 ppm) (**21a**)

IR (ATR)/cm^{-1} 3033, 2948, 2869, 2253, 1707, 1454, 1316, 1276, 1233, 1145, 1109, 750, 707 (**21a**)

*The relative configuration of the isolated diastereomers **21a** and **21b** was not determined.*

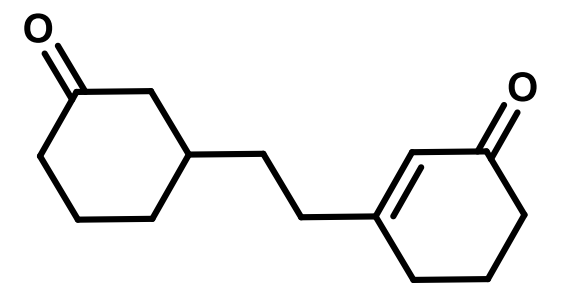

22

1,6-addition product 22. Compound **22** was prepared from cyclopropyl silyl ether **1** (0.10 mmol) and 3-vinylcyclohex-2-en-1-one (0.07 mmol) (prepared according to the procedure of Krasutsky *et al.*[9]) according to *Method B2*. The solvent was removed under reduced pressure. The residue was then redissolved in THF (1.3 mL), and DBU (0.20 mmol) was added. The mixture was left to stir for 6 h at 65 °C. After concentrating the reaction mixture under reduced pressure, the crude was purified by flash column chromatography (silica gel, 10 to 100% EtOAc in PE) to afford **22** (10.8 mg, 0.049 mmol, 70%) as a thick yellow oil.

R_f 0.49 (silica gel, 40% EtOAc in PE)

^{1}**H NMR** (400 MHz, $CDCl_3$) δ 5.85 (app. t, *J* = 1.4 Hz, 1H), 2.46 – 2.40 (m, 1H), 2.39 – 2.32 (m, 3H), 2.29 – 2.20 (m, 4H), 2.08 – 1.89 (m, 5H), 1.84 – 1.43 (m, 5H), 1.41 – 1.32 (m, 1H).

^{13}**C NMR** (101 MHz, $CDCl_3$) δ 211.4, 199.9, 165.8, 125.9, 48.0, 41.5, 38.7, 37.4, 35.2, 33.7, 31.2, 29.8, 25.2, 22.8.

HRMS (ESI+) *m*/z: calcd. for $C_{14}H_{21}O_2^+$ $[M+H]^+$: 221.1536; found: 221.1537, (Δ = 0.45 ppm)

IR (ATR)/cm^{-1} 2935, 2867, 1709, 1666, 1624, 1603, 1454, 1426, 1348, 1325, 1258, 1192

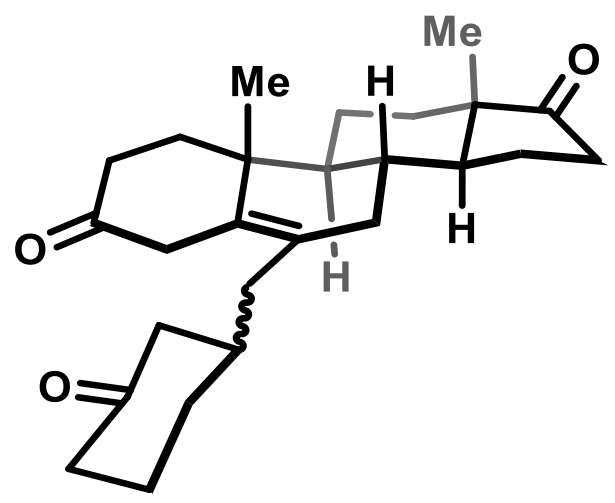

23

1,6-addition product 23. Compound **23** was prepared from cyclopropyl silyl ether **1** (0.40 mmol) and 6-methyleneandrost-4-ene-3,17-dione (0.20 mmol) according to *Method B2* (24 h, 40 °C). Purification by flash column chromatography (silica gel, 10% to 100% EtOAc in PE) afforded **23** (52.3 mg, 0.132 mmol, 66%, 1.2:1 dr) as a faint yellow solid.

R_f 0.50 (silica gel, 40% EtOAc in PE)

^{1}**H NMR** (500 MHz, $CDCl_3$) δ 3.30 (ddd, *J* = 16.9, 5.5, 1.5 Hz, 1H), 3.09 – 2.97 (m, 1H), 2.52 – 2.41 (m, 3H), 2.35 – 2.23 (m, 4H), 2.13 – 1.88 (m, 11H), 1.73 – 1.64 (m, 3H), 1.57 – 1.49 (m, 3H), 1.34 – 1.25 (m, 4H), 1.17 (app. d, *J* = 4.0 Hz, 3H), 0.91 – 0.89 (m, 3H). (Summary of diastereomers)

^{13}**C NMR** (126 MHz, $CDCl_3$) δ 220.73, 220.71, 211.7, 211.6, 210.5, 210.41, 133.7, 128.59, 128.55, 51.8, 51.7, 49.1, 49.0, 48.2, 47.8, 47.6, 43.07, 43.05, 41.51, 41.49, 40.3, 40.2, 38.34, 38.28, 37.94, 37.88, 37.2, 37.1, 36.3, 36.1, 36.0, 35.9, 31.9, 31.52, 31.48, 31.47, 31.4, 25.6, 25.5, 21.9, 20.9, 20.04, 20.02, 13.76, 13.75. (Summary of diastereomers, multiple overlapping signals)

HRMS (ESI+) *m*/z: calcd. for $C_{26}H_{37}O_3^+$ $[M+H]^+$: 397.2737; found: 397.2738, (Δ = 0.25 ppm)

IR (ATR)/cm^{-1} 2943, 2859, 1736, 1710, 1670, 1455, 1275

MP 90-95 °C

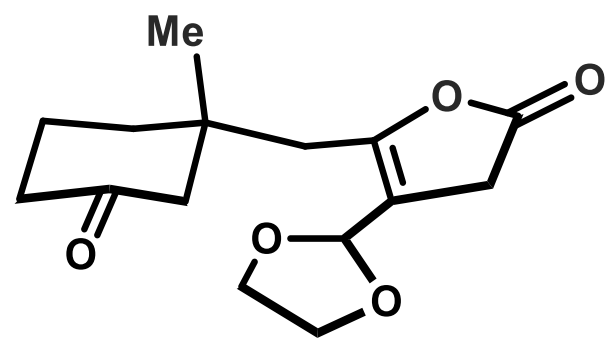

24

1,6-addition product 24. Compound **24** was prepared from cyclopropyl silyl ether **S3** (2.71 mmol) and lactone **S16** (2.03 mmol) according to *Method B2* (2,2,2-trifluoroethanol instead of HFIP). Purification by flash column chromatography (silica gel, 20% to 80% EtOAc in PE) afforded **24** (387 mg, 1.38 mmol, 68%) as a faint yellow oil.

R$_f$ 0.19 (silica gel, 40% EtOAc in PE)

^{1}H NMR (400 MHz, C_6D_6) δ 5.18 (app. s, 1H), 3.43 – 3.19 (m, 4H), 2.93 – 2.91 (m, 1H), 2.02 – 1.88 (m, 4H), 1.84 – 1.74 (m, 1H), 1.42 – 1.21 (m, 5H), 1.13 – 1.07 (m, 1H), 0.72 (s, 3H).

^{13}C NMR (101 MHz, C_6D_6) δ 208.6, 173.8, 152.5, 113.6, 98.1, 65.17, 65.15, 53.3, 40.6, 38.9, 37.8, 35.9, 31.8, 25.1, 22.0.

HRMS (ESI+) *m*/z: calcd. for $C_{15}H_{21}O_5^+$ $[M+H]^+$: 281.1384; found: 281.1384, (Δ = 0 ppm)

IR (ATR)/cm^{-1} 2955, 2898, 1802, 1705, 1275, 1260, 1212, 1150, 1087, 998, 953, 921

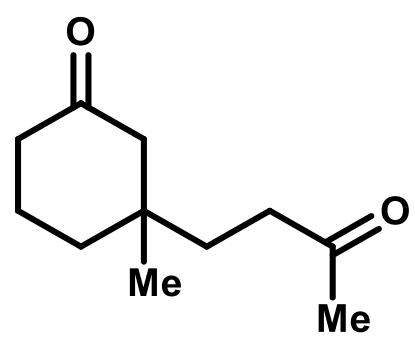

25

β–β coupled product 25. Compound **25** was prepared from cyclopropyl silyl ether **S3** (0.15 mmol) and methyl vinyl ketone (0.10 mmol) according to *Method B1*. Purification by flash column chromatography (silica gel, 10% to 60% EtOAc in PE) afforded **25** (12.0 mg, 0.066 mmol, 66%) as a faint yellow oil.

R$_f$ 0.48 (silica gel, 40% EtOAc in PE)

^{1}H NMR (400 MHz, $CDCl_3$) δ 2.40 (td, *J* = 7.5, 2.8 Hz, 2H), 2.30 – 2.24 (m, 2H), 2.19 – 2.07 (m, 5H), 1.92 – 1.81 (m, 2H), 1.60 – 1.53 (m, 4H), 0.89 (s, 3H).

^{13}C NMR (101 MHz, $CDCl_3$) δ 211.8, 208.7, 53.3, 41.0, 38.2, 38.1, 36.1, 35.2, 30.2, 24.5, 22.1.

HRMS (ESI+) *m*/z: calcd. for $C_{11}H_{18}NaO_2^+$ $[M+Na]^+$: 205.1199; found: 205.1200, (Δ = 0.49 ppm)

IR (ATR)/cm^{-1} 2955, 2925, 2874, 2851, 1706, 1455, 1420, 1355, 1312, 1229, 1164

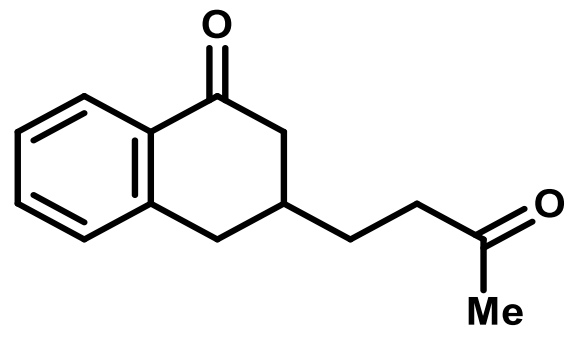

26

β–β coupled product 26. Compound **26** was prepared from cyclopropyl silyl ether **S5** (0.15 mmol) and methyl vinyl ketone (0.10 mmol) according to *Method B1*. Purification by flash column chromatography (silica gel, 10% to 60% EtOAc in PE) afforded **26** (19.9 mg, 0.092 mmol, 92%) as a faint red solid.

$\mathbf{R_f}$ 0.33 (silica gel, 30% EtOAc in PE)

^{1}H NMR (400 MHz, $CDCl_3$) δ 8.01 (dd, *J* = 7.8, 1.4 Hz, 1H), 7.48 (td, *J* = 7.5, 1.5 Hz, 1H), 7.31 (t, *J* = 7.6 Hz, 1H), 7.25 (d, *J* = 7.9 Hz, 1H), 3.01 (ddd, *J* = 16.2, 4.1, 1.9 Hz, 1H), 2.81 – 2.67 (m, 2H), 2.55 (t, *J* = 7.5 Hz, 2H), 2.33 (dd, *J* = 16.4, 11.9 Hz, 1H), 2.25 – 2.18 (m, 1H), 2.18 (s, 3H), 1.83 – 1.65 (m, 2H).

^{13}C NMR (126 MHz, $CDCl_3$) δ 208.2, 198.0, 143.4, 133.8, 132.4, 129.1, 127.2, 127.0, 45.2, 40.8, 36.2, 35.0, 30.2, 29.4.

HRMS (ESI+) *m*/z: calcd. for $C_{14}H_{17}O_2^+$ $[M+H]^+$: 217.1223; found: 217.1224, (Δ = 0.46 ppm)

IR (ATR)/cm^{-1} 2925, 2857, 1712, 1680, 1601, 1455, 1358, 1288, 1158, 1024, 764

MP 65 °C

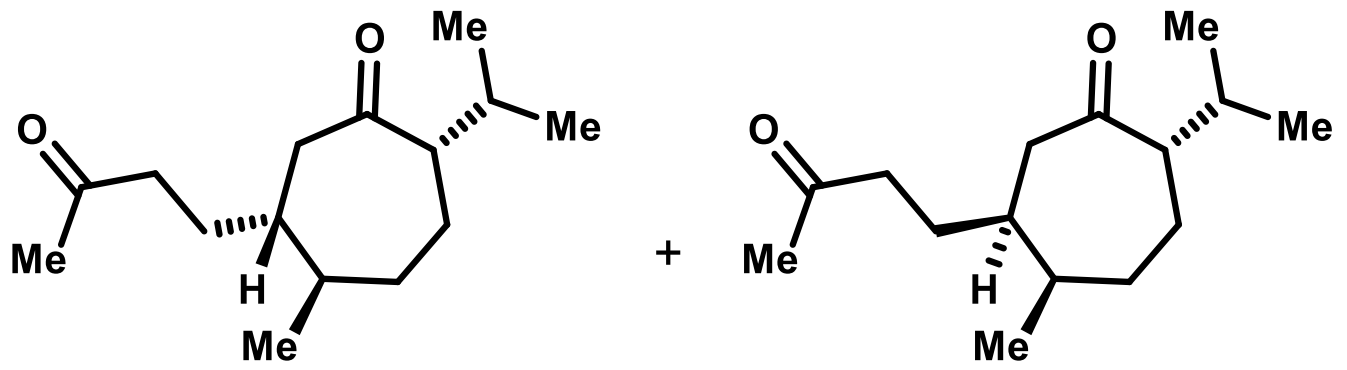

27a, *major* **27b**, *minor*

β–β coupled products 27a and **27b.** Compounds **27a** and **27b** were prepared from cyclopropyl silyl ether **35** (0.23 mmol) and methyl vinyl ketone (0.15 mmol) according to *Method B1*. Purification by flash column chromatography (silica gel, 10% to 60% EtOAc in PE) afforded diastereomers **27a** (25.3 mg, 0.106 mmol, 71%) and **27b** (6.2 mg, 0.026 mmol, 17%) as orange oils (combined yield 88%; 4.1:1 isolated dr).

$\mathbf{R_f}$ 0.29 (**27a**); 0.43 (**27b**) (silica gel, 20% EtOAc in PE)

^{1}H NMR (400 MHz, $CDCl_3$) δ 2.56 – 2.35 (m, 3H), 2.15 (s, 3H), 2.12 – 2.09 (m, 1H), 2.06 – 1.96 (m, 2H), 1.95 – 1.85 (m, 1H), 1.83 – 1.70 (m, 2H), 1.46 – 1.35 (m, 2H), 1.33 – 1.27 (m, 1H), 1.25 – 1.18 (m, 1H), 1.06 – 0.98 (m, 1H), 0.94 (d, *J* = 6.5 Hz, 3H), 0.91 (d, *J* = 6.7 Hz, 3H), 0.84 (d, *J* = 6.8 Hz, 3H). (**27a**)

^{1}H NMR (400 MHz, $CDCl_3$) δ 2.43 (t, *J* = 7.1 Hz, 2H), 2.14 (s, 3H), 2.10 – 2.04 (m, 3H), 1.98 – 1.92 (m, 1H), 1.89 – 1.83 (m, 1H), 1.62 – 1.57 (m, 2H), 1.48 – 1.41 (m, 3H), 1.35 – 1.27 (m, 2H), 1.04 (d, *J* = 6.2 Hz, 3H), 0.89 (d, *J* = 6.4 Hz, 3H), 0.85 (d, *J* = 6.5 Hz, 3H). (**27b**)

^{13}C NMR (101 MHz, $CDCl_3$) δ 216.5, 208.8, 60.7, 45.0, 44.1, 40.8, 35.3, 31.4, 30.2, 28.4, 27.0, 21.5, 21.2, 19.9. (**27a**)

^{13}C NMR (126 MHz, $CDCl_3$) δ 213.6, 209.5, 58.1, 57.3, 44.5, 40.5, 35.0, 29.6, 26.4, 25.6, 22.0, 21.7, 20.9, 19.0. (**27b**)

HRMS (ESI+) *m*/z: calcd. for $C_{15}H_{27}O_2^+$ $[M+H]^+$: 239.2006; found: 239.2007, (Δ = 0.42 ppm) (**27a**)

IR (ATR)/cm^{-1} 2958, 2929, 2873, 1698, 1459, 1339, 1214, 1164, 948 (**27a**); 2956, 2929, 2871, 1704, 1457, 1366, 1223, 1191, 948 (**27b**)

$([\alpha])_{D}^{23}$ = −80 (*c* = 0.90, $CHCl_3$) (**27a**); 40 (*c* = 0.90, $CHCl_3$) (**27b**)

*The relative configuration of the major diastereomer **27a** was assigned based on the stereochemical assignment in the downstream condensation product (**38a**) one step later, whose structure was unambiguously confirmed by 2D NMR (see Supporting Information (SI), pp. **S157**).*

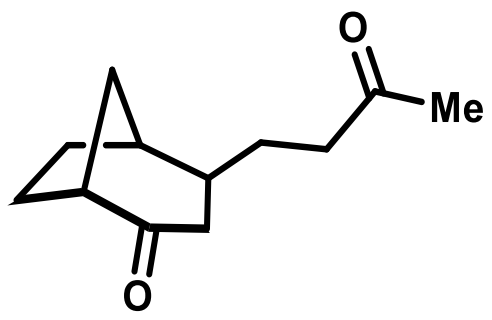

28

β–β coupled product 28. Compound **28** was prepared from cyclopropyl silyl ether **S6** (0.15 mmol) and methyl vinyl ketone (0.10 mmol) according to *Method B1*. Purification by flash column chromatography (silica gel, 10% to 60% EtOAc in PE) afforded **28** (16.1 mg, 0.083 mmol, 83%, 2.4:1 dr) as a faint yellow oil.

R_f 0.18 (silica gel, 20% EtOAc in PE)

^{1}H NMR (400 MHz, $CDCl_3$) δ 2.67 (t, *J* = 5.7 Hz, 1H), 2.54 – 2.37 (m, 3H), 2.26 – 2.21 (m, 1H), 2.17 – 2.09 (m, 3H), 2.08 – 1.97 (m, 1H), 1.97 – 1.89 (m, 2H), 1.88 – 1.80 (m, 2H), 1.79 – 1.69 (m, 2H), 1.65 – 1.60 (m, 2H), 1.58 – 1.50 (m, 1H). (Summary of diastereomers)

^{13}C NMR (101 MHz, $CDCl_3$) δ 214.2, 213.4, 208.5, 208.4, 51.3, 50.6, 42.0, 41.7, 41.6, 41.2, 41.1, 40.3, 38.53, 38.51, 38.4, 32.9, 30.12, 30.08, 29.5, 28.2, 28.1, 27.8, 27.5, 22.9. (Summary of diastereomers)

HRMS (ESI+) *m*/*z*: calcd. for $C_{12}H_{19}O_2^+$ $[M+H]^+$: 195.1380; found: 195.1381, (Δ = 0.51 ppm)

IR (ATR)/cm^{-1} 2948, 2873, 1705, 1452, 1422, 1357, 1260, 1239, 1158, 1105, 1069

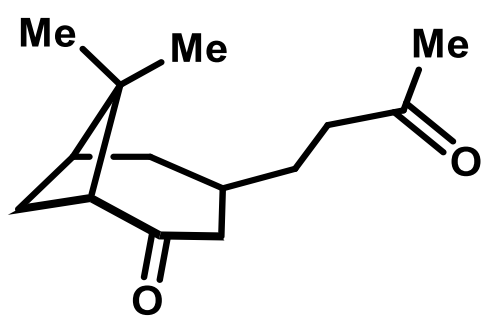

29

β–β coupled product 29. Compound **29** was prepared from cyclopropyl silyl ether **S7** (0.15 mmol) and methyl vinyl ketone (0.10 mmol) according to *Method B1*. Purification by flash column chromatography (silica gel, 10% to 60% EtOAc in PE) afforded **29** (14.9 mg, 0.067 mmol, 67%, 4:1 dr) as a faint yellow oil.

R_f 0.2 (silica gel, 20% EtOAc in PE)

^{1}H NMR (400 MHz, $CDCl_3$) δ 2.86 (t, *J* = 13.0 Hz, 1H), 2.67 – 2.62 (m, 1H), 2.56 – 2.50 (m, 1H), 2.49 (t, *J* = 7.7 Hz, 2H), 2.37 – 2.31 (m, 1H), 2.30 – 2.23 (m, 1H), 2.21 – 2.17 (m, 1H), 2.15 (s, 3H), 1.78 (d, *J* = 12.2 Hz, 1H), 1.71 – 1.57 (m, 2H), 1.56 – 1.45 (m, 2H), 1.36 – 1.32 (m, 3H), 0.98 (s, 3H). (Summary of diastereomers)

^{13}C NMR (101 MHz, $CDCl_3$) δ 215.4, 212.7, 208.5, 208.4, 61.3, 59.3, 49.2, 47.3, 44.0, 41.6, 41.3, 41.2, 39.7, 36.0, 32.8, 32.5, 32.4, 31.6, 31.2, 30.6, 30.5, 30.1, 28.4, 22.7, 21.0, 20.9. (Summary of diastereomers)

HRMS (ESI+) *m*/*z*: calcd. for $C_{14}H_{23}O_2^+$ $[M+H]^+$: 223.1693; found: 223.1694, (Δ = 0.45 ppm)

IR (ATR)/cm^{-1} 2953, 2925, 2871, 1714, 1688, 1369, 1260, 1220

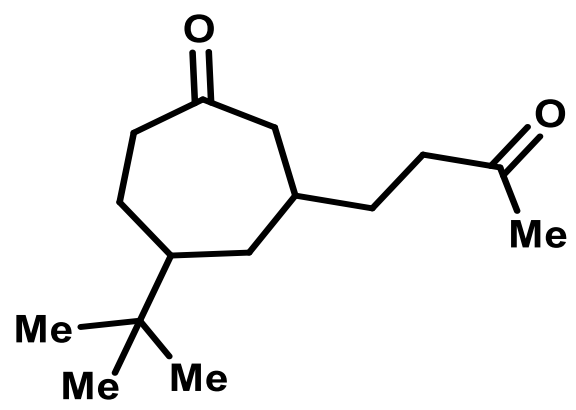

30

β–β coupled product 30. Compound **30** was prepared from cyclopropyl silyl ether **S12** (0.15 mmol) and methyl vinyl ketone (0.10 mmol) according to *Method B1*. Purification by flash column chromatography (silica gel, 10% to 40% EtOAc in PE) afforded diastereomerically pure **30** (18.1 mg, 0.076 mmol, 76%) as a colorless oil.

R_f 0.5 (silica gel, 30% EtOAc in PE)

^{1}H NMR (400 MHz, $CDCl_3$) δ 2.51 – 2.36 (m, 5H), 2.15 (s, 3H), 2.03 – 1.91 (m, 2H), 1.68 – 1.54 (m, 3H), 1.41 – 1.33 (m, 1H), 1.11 – 0.92 (m, 3H), 0.87 (s, 9H).

^{13}C NMR (101 MHz, $CDCl_3$) δ 214.4, 208.6, 50.9, 49.6, 43.3, 41.3, 38.3, 36.2, 33.9, 32.0, 30.2, 27.6, 26.3.

HRMS (ESI+) *m*/z: calcd. for $C_{15}H_{27}O_2^+$ [M+H]$^+$: 239.2006; found: 239.2009, (Δ = 1.25 ppm)

IR (ATR)/cm^{-1} 2951, 2868, 1703, 1366, 1216, 1163

*The relative configuration of the isolated diastereomer of **30** was not determined.*

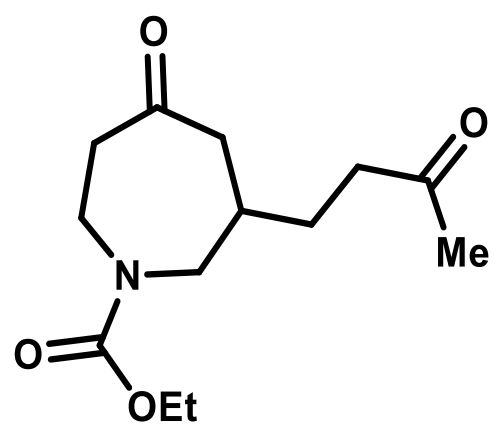

31

β–β coupled product 31. Compound **31** was prepared from cyclopropyl silyl ether **S10** (0.23 mmol) and methyl vinyl ketone (0.15 mmol) according to *Method B1*. Purification by flash column chromatography (silica gel, 40% to 100% EtOAc in PE) afforded **31** (28.1 mg, 0.110 mmol, 73%) as a white viscous oil.

R_f 0.18 (silica gel, 60% EtOAc in PE)

^{1}H NMR (500 MHz, $CDCl_3$) δ 4.12 (q, *J* = 6.9 Hz, 2H), 3.85 – 3.65 (m, 2H), 3.62 – 3.50 (m, 1H), 3.34 – 3.17 (m, 1H), 2.73 – 2.46 (m, 6H), 2.14 (s, 3H), 1.83 (s, 1H), 1.63 – 1.44 (m, 2H), 1.25 (t, *J* = 7.1 Hz, 3H). (Summary of rotamers)

^{13}C NMR (126 MHz, $CDCl_3$) δ 210.7, 210.5, 208.1, 207.9, 155.6, 155.5, 61.8, 54.1, 53.9, 48.8, 48.4, 44.0, 43.9, 43.6, 43.3, 41.1, 40.9, 36.8, 36.7, 30.2, 26.5, 26.4, 14.8. (Summary of rotamers, multiple overlapping signals)

HRMS (ESI+) *m*/z: calcd. for $C_{13}H_{22}NO_4^+$ [M+H]$^+$: 256.1543; found: 256.1545, (Δ = 0.78 ppm)

IR (ATR)/cm^{-1} 2959, 2924, 2856, 1690, 1474, 1423, 1260, 1237, 1169, 1096, 1024

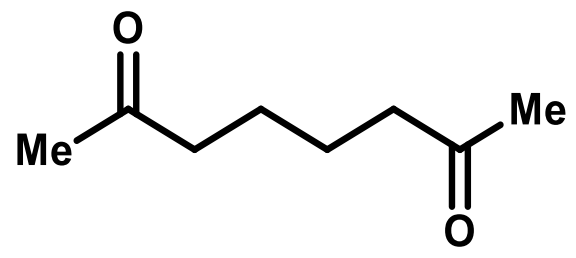

32

β–β coupled product 32. Compound **32** was prepared from cyclopropyl silyl ether **S11** (0.23 mmol) and methyl vinyl ketone (0.15 mmol) according to *Method B1*. Purification by flash column chromatography (silica gel, 10% to 50% EtOAc in PE) afforded **32** (16.5 mg, 0.116 mmol, 77%) as a faint yellow oil. Analytical data (^{1}H, ^{13}C NMR, HRMS) were in agreement with those previously described.[10]

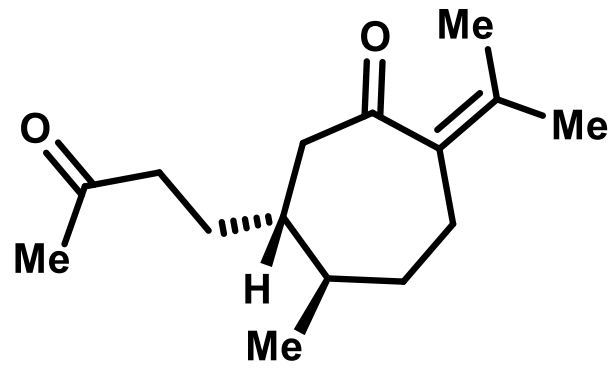

33

β–β coupled product 33. Compound **33** was prepared from cyclopropyl silyl ether **39** (0.15 mmol) and methyl vinyl ketone (0.10 mmol) according to *Method B1*. Purification by flash column chromatography (silica gel, 10% to 50% EtOAc in PE) afforded diastereomerically pure **33** (13.5 mg, 0.057 mmol, 57%) as an orange oil.

R_f 0.29 (silica gel, 20% EtOAc in PE)

^{1}H NMR (400 MHz, $CDCl_3$) δ 2.54 – 2.47 (m, 2H), 2.47 – 2.43 (m, 2H), 2.42 – 2.34 (m, 1H), 2.27 (dd, *J* = 15.7, 9.8 Hz, 1H), 2.14 (s, 3H), 1.90 (s, 3H), 1.87 – 1.79 (m, 2H), 1.77 (s, 3H), 1.60 – 1.56 (m, 1H), 1.55 – 1.47 (m, 1H), 1.45 – 1.39 (m, 1H), 1.36 – 1.29 (m, 1H), 1.00 (d, *J* = 6.7 Hz, 3H).

^{13}C NMR (101 MHz, $CDCl_3$) δ 208.9, 207.3, 139.7, 136.6, 46.6, 41.3, 40.8, 38.4, 34.3, 30.2, 27.7, 25.8, 22.7, 21.9, 20.3.

HRMS (ESI+) *m*/z: calcd. for $C_{15}H_{25}O_2^+$ $[M+H]^+$: 237.1849; found: 237.1850, (Δ = 0.42 ppm)

IR (ATR)/cm^{-1} 2956, 2924, 2873, 1714, 1676, 1616, 1446, 1362, 1220, 1163

*The relative configuration of **33** was assigned based on the stereochemical assignment (−)-ent-*melicodenone C *(**41**) two steps later, whose NMR data matched the reported literature.*

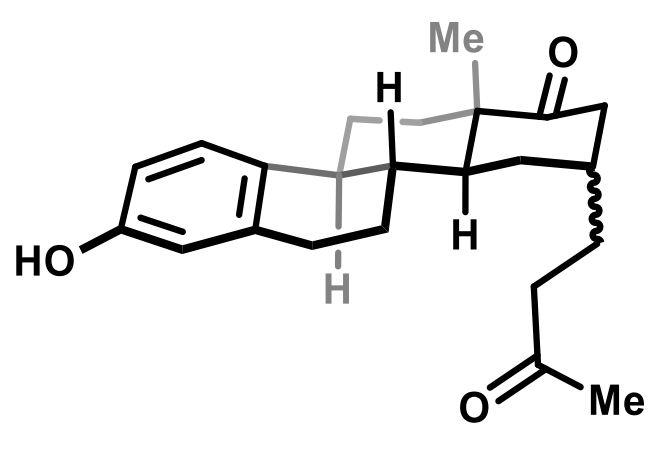

34

β–β coupled product 34. Compound **34** was prepared from cyclopropyl silyl ether **S9a** (0.15 mmol) and methyl vinyl ketone (0.10 mmol) according to *Method B1*. Purification by flash column chromatography (silica gel, 10% to 50% EtOAc in PE) afforded **34** (23.0 mg, 0.065 mmol, 65%, 2:1 dr) as a white solid.

R_f 0.39 (silica gel, 40% EtOAc in PE)

^{1}H NMR (400 MHz, $CDCl_3$) δ 7.16 (dd, *J* = 8.4, 2.8 Hz, 1H), 6.64 (dd, *J* = 8.4, 2.8 Hz, 1H), 6.57 (d, *J* = 2.7 Hz, 1H), 4.93 – 4.80 (m, 1H), 2.82 (dd, *J* = 8.8, 4.3 Hz, 2H), 2.78 – 2.67 (m, 1H), 2.52 – 2.47 (m, 1H), 2.46 – 2.34 (m, 2H), 2.28 – 2.21 (m, 1H), 2.18 – 2.14 (m, 4H), 2.06 – 1.98 (m, 1H), 1.88 (tt, *J* = 12.6, 3.4 Hz, 2H), 1.77 – 1.68 (m, 2H), 1.59 – 1.54 (m, 2H), 1.47 – 1.39 (m, 2H), 1.31 – 1.21 (m, 3H), 1.12 – 1.07 (m, 3H). (Summary of diastereomers)

^{13}C NMR (126 MHz, $CDCl_3$) δ 216.4, 215.6, 208.6, 208.5, 153.6, 138.1, 138.0, 132.60, 132.59, 126.74, 126.67, 115.1, 115.0, 113.03, 113.00, 49.1, 48.0, 47.9, 44.1, 43.4, 43.2, 43.1, 41.8, 41.6, 40.9, 39.0, 38.8, 38.2, 33.5, 32.7, 32.5, 30.9, 30.18, 30.15, 30.01, 29.96, 28.4, 28.2, 26.7, 26.6, 26.0, 25.9, 17.0, 16.9. (Summary of diastereomers, two unspecified overlapping signals)

HRMS (ESI+) *m*/z: calcd. for $C_{23}H_{31}O_3^+$ $[M+H]^+$: 355.2268; found: 355.2266, (Δ = −0.56 ppm)

IR (ATR)/cm^{-1} 3375, 2926, 2864, 1699, 1612, 1501, 1448, 1357, 1260, 1157, 1095, 1017, 867, 800, 749, 701

MP 160 °C

Quantitative ^{1}H NMR (qNMR) analysis using mesitylene (1.0 equiv., 3 H, integral = 1.50 at 6.81 ppm) as an internal standard indicated a yield of 92%, based on product signals of 1 H (integral = 0.46) and 2 H (integral = 0.92).

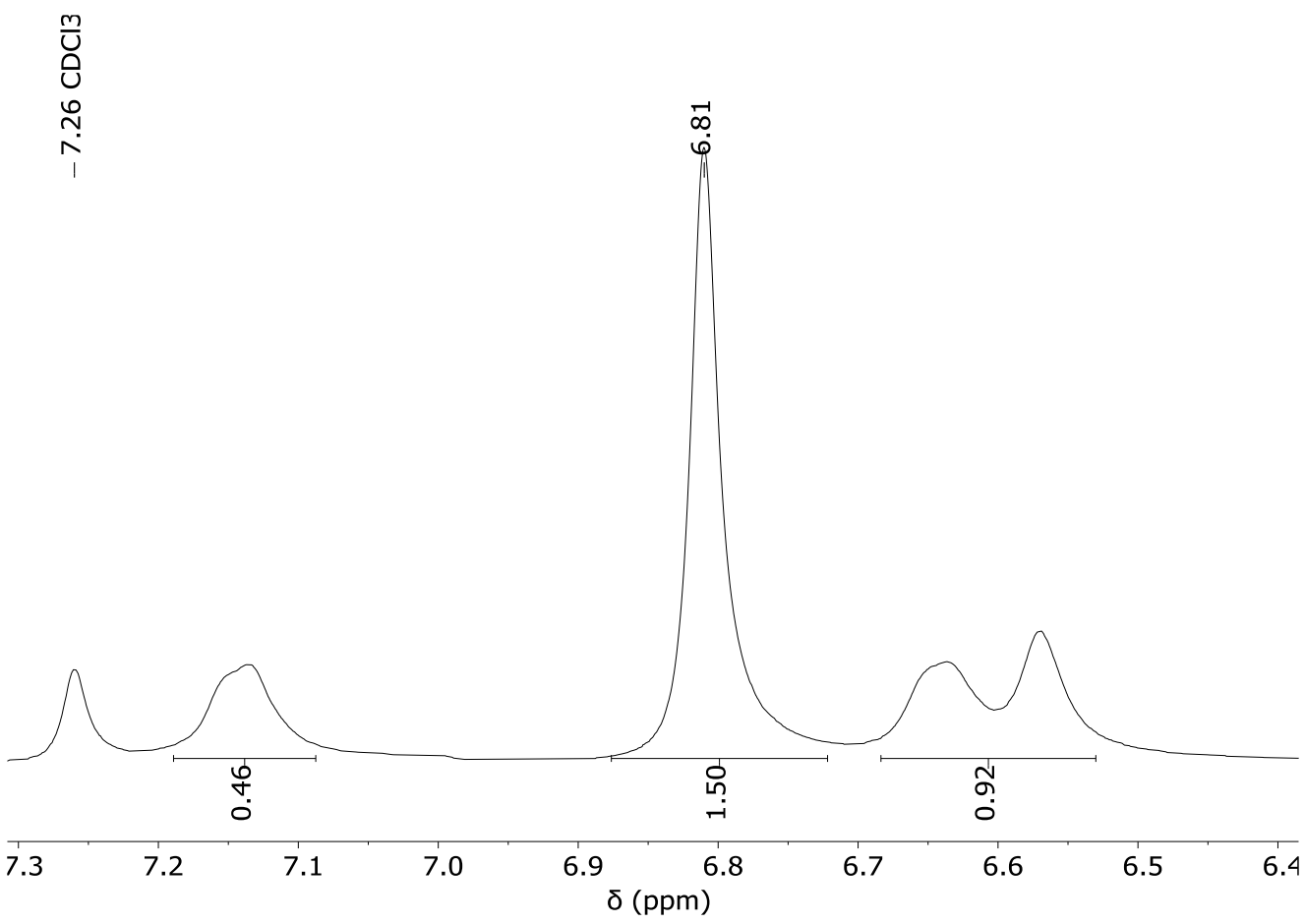

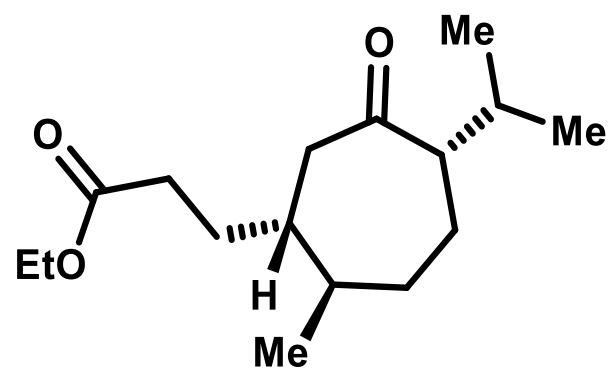

36

β–β coupled product 36. Compound **36** was prepared from cyclopropyl silyl ether **35** (0.27 mmol) and ethyl acrylate (0.13 mmol) according to *Method B*1. Purification by flash column chromatography (silica gel, 10% to 50% EtOAc in PE) afforded diastereomerically pure **36** (27.2 mg, 0.101 mmol, 78%) as a colorless oil. Analytical data (^{1}H, ^{13}C NMR, HRMS) were in agreement with those previously described.[6]

R_f 0.48 (silica gel, 30% EtOAc in PE)

^{1}H NMR (500 MHz, $CDCl_3$) δ 4.13 (q, *J* = 7.1 Hz, 2H), 2.51 (t, *J* = 11.1 Hz, 1H), 2.38 (ddd, *J* = 15.6, 10.2, 5.4 Hz, 1H), 2.25 (ddd, *J* = 16.0, 10.0, 6.5 Hz, 1H), 2.16 (dd, *J* = 11.2, 2.0 Hz, 1H), 2.06 – 1.93 (m, 3H), 1.84 – 1.72 (m, 2H), 1.56 – 1.49 (m, 1H), 1.42 – 1.31 (m, 3H), 1.26 (t, *J* = 7.1 Hz, 3H), 1.06 – 1.01 (m, 1H), 0.96 (d, *J* = 6.1 Hz, 3H), 0.92 (d, *J* = 6.7 Hz, 3H), 0.85 (d, *J* = 6.7 Hz, 3H).

^{13}C NMR (126 MHz, $CDCl_3$) δ 216.3, 173.8, 60.7, 60.6, 44.9, 43.9, 40.6, 35.3, 31.4, 31.3, 29.9, 27.0, 21.4, 21.2, 19.9, 14.4.

HRMS (ESI+) *m/z*: calcd. for $C_{16}H_{28}NaO_3^+$ $[M+Na]^+$: 291.1931; found: 291.1924, (Δ = −2.40 ppm)

IR (ATR)/cm^{-1} 2958, 2928, 2872, 1745, 1702, 1461, 1276, 1261, 1172

$([\alpha])_D^{23}$ = −97 (*c* = 0.75, $CHCl_3$); **$([\alpha])_D^{25}$ =** −87.5 (*c* = 0.62, $CHCl_3$, lit.[46])

A close-eluting side-product complicated the isolation; in order to obtain a pure sample, the purification had to be done four times.

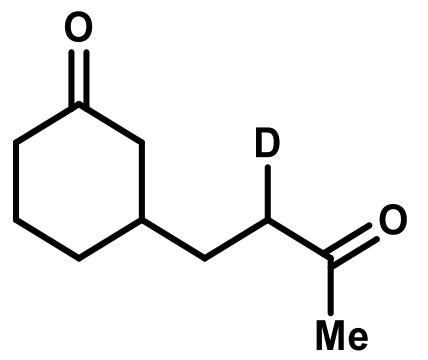

43

β–β coupled product 43. Compound **43** was prepared from cyclopropyl silyl ether **1** (0.23 mmol) and methyl vinyl ketone (0.15 mmol) according to *Method B1* (D_2O instead of HFIP). Purification by flash column chromatography (silica gel, 10% to 60% EtOAc in PE) afforded **43** (4.1 mg, 0.024 mmol, 16%) as a faint yellow oil.

^{1}H NMR (500 MHz, $CDCl_3$) δ 2.48 – 2.33 (m, 3H), 2.30 – 2.22 (m, 1H), 2.15 (s, 3H), 2.08 – 1.97 (m, 2H), 1.93 – 1.86 (m, 1H), 1.79 – 1.70 (m, 1H), 1.68 – 1.53 (m, 3H), 1.39 – 1.30 (m, 1H).

^{13}C NMR (126 MHz, $CDCl_3$) δ 211.4, 208.5, 48.0, 41.5, 40.6 (t, *J* = 19.0 Hz), 38.7, 31.4, 30.2, 30.1, 25.3.

HRMS (ESI+) *m/z*: calcd. for $C_{10}H_{15}DNaO_2^+$ $[M+Na]^+$: 192.1105; found: 192.1106, (Δ = 0.52 ppm)

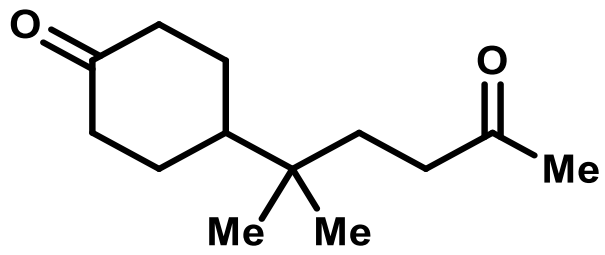

5

Tandem product 5. Compound **5** was prepared from cyclopropyl silyl ether **3** (0.23 mmol) and methyl vinyl ketone (0.15 mmol) according to *Method B1*. Purification by flash column chromatography (silica gel, 10% to 50% EtOAc in PE) afforded **5** (11.1 mg, 0.053 mmol, 35%) as a faint orange oil.

R_f 0.42 (silica gel, 40% EtOAc in PE)

^{1}H NMR (500 MHz, $CDCl_3$) δ 2.44 – 2.38 (m, 4H), 2.29 (td, *J* = 14.0, 5.7 Hz, 2H), 2.17 (s, 3H), 2.03 (dd, *J* = 10.1, 5.3 Hz, 2H), 1.67 – 1.59 (m, 2H), 1.57 – 1.54 (m, 1H), 1.53 – 1.42 (m, 2H), 0.87 (s, 6H).

^{13}C NMR (126 MHz, $CDCl_3$) δ 212.3, 209.3, 45.0, 41.4, 38.7, 34.6, 33.9, 30.2, 27.2, 24.6.

HRMS (ESI+) *m/z*: calcd. for $C_{13}H_{23}O_2^+$ $[M+H]^+$: 211.1693; found: 211.1696, (Δ = 1.42 ppm)

IR (ATR)/cm^{-1} 2955, 2872, 1710, 1702, 1419, 1367, 1217, 1163

Synthesis and Characterization of Radical-polar Crossover Products

*The relative configuration of products **44–55, 58**, and **61**-**64** was assigned based on 2D NMR analysis of selected representative compounds (**44, 46, 48a, 50**-**51, 58, 61** and **63**). These compounds exhibited consistent NMR correlations; accordingly, the same relative configuration was assigned to the full series (see SI, pp. **S141**-**S150**). This assignment is supported by the observation of three and four NOE correlations, respectively, between protons of the bicyclic ring system and the α-CH proton, or vinyl CH proton signal, in the bicyclo[3.2.1] (**44-50, 53-55, 58, 62**-**64**) and bicyclo[4.2.1] (**51-52, 61**) series, whereas*

such correlations would not be expected for the other possible diastereomer. Deviations from this assignment are indicated explicitly where applicable. Note: retro-aldol reaction was sometimes observed when using old and non-basified $CDCl_3$ for NMR analysis. Likewise, extended heating during rotary evaporation sometimes gave retro-aldol reactions. Removal of traces of solvents was best performed by blowing N_2 onto the purified compounds.

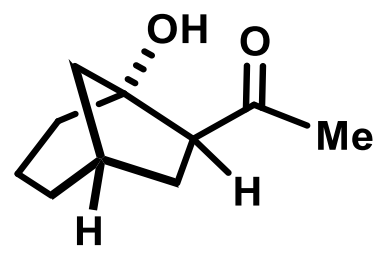

44

Radical-polar crossover product 44. Compound **44** was prepared from cyclopropyl silyl ether **1** (0.23 mmol) and methyl vinyl ketone (0.15 mmol) according to *Method C1* ($ZnCl_2$ instead of $LiBF_4$). Purification by flash column chromatography (silica gel, 10% to 60% EtOAc in PE) afforded diastereomerically pure **44** (21.2 mg, 0.126 mmol, 84%) as a faint yellow viscous oil.

R_f 0.44 (silica gel, 40% EtOAc in PE)

^{1}H NMR (500 MHz, $CDCl_3$) δ 3.14 (app. d, *J* = 6.0 Hz, 1H), 2.83 (dd, *J* = 9.7, 5.8 Hz, 1H), 2.26 – 2.20 (m, 1H), 2.17 (s, 3H), 2.05 – 1.99 (m, 1H), 1.99 – 1.93 (m, 1H), 1.77 – 1.67 (m, 3H), 1.62 – 1.50 (m, 2H), 1.48 – 1.40 (m, 1H), 1.36 – 1.28 (m, 2H).

^{13}C NMR (126 MHz, $CDCl_3$) δ 213.1, 80.4, 54.8, 44.6, 39.8, 33.5, 32.9, 31.5, 30.6, 19.8.

HRMS (ESI+) *m*/z: calcd. for $C_{10}H_{16}NaO_2^+$ $[M+Na]^+$: 191.1043; found: 191.1046, (Δ = 1.57 ppm)

IR (ATR)/cm^{-1} 3433, 2929, 2854, 1698, 1447, 1356, 1290, 1170, 1126, 1104, 971

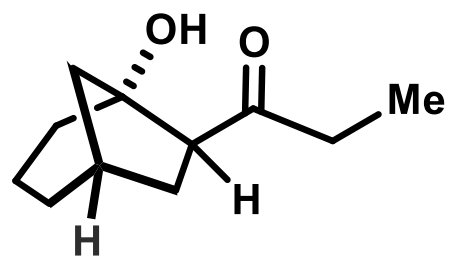

45

Radical-polar crossover product 45. Compound **45** was prepared from cyclopropyl silyl ether **1** (0.23 mmol) and ethyl vinyl ketone (0.15 mmol) according to *Method C1*. Purification by flash column chromatography (silica gel, 10% to 60% EtOAc in PE) afforded diastereomerically pure **45** (19.1 mg, 0.105 mmol, 70%) as a yellow viscous oil.

R_f 0.49 (silica gel, 40% EtOAc in PE)

^{1}H NMR (500 MHz, $CDCl_3$) δ 3.01 (s, 1H), 2.83 (dd, *J* = 9.7, 5.8 Hz, 1H), 2.47 (qd, *J* = 7.3, 5.5 Hz, 2H), 2.27 – 2.19 (m, 1H), 2.09 – 2.01 (m, 1H), 1.98 – 1.89 (m, 1H), 1.77 – 1.66 (m, 3H), 1.63 – 1.50 (m, 2H), 1.47 – 1.41 (m, 1H), 1.36 – 1.28 (m, 2H), 1.04 (t, *J* = 7.3 Hz, 3H).

^{13}C NMR (126 MHz, $CDCl_3$) δ 215.7, 80.5, 54.0, 44.8, 39.7, 37.4, 33.6, 33.2, 30.7, 19.8, 7.6.

HRMS (ESI−) *m*/z: calcd. for $C_{11}H_{17}O_2^-$ $[M-H]^-$: 181.1234; found: 181.1236, (Δ = 1.10 ppm)

IR (ATR)/cm^{-1} 3464, 2927, 2854, 1709, 1458, 1376, 1363, 1132, 1104

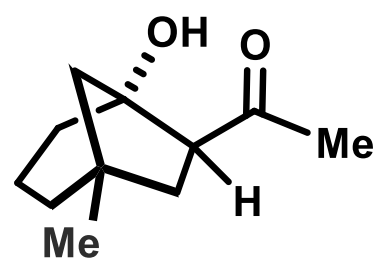

46

Radical-polar crossover product 46. Compound **46** was prepared from cyclopropyl silyl ether **S3** (0.23 mmol) and methyl vinyl ketone (0.15 mmol) according to *Method C1*. Purification by flash column chromatography (silica gel, 10% to 60% EtOAc in PE) afforded diastereomerically pure **46** (21.6 mg, 0.119 mmol, 79%) as a faint yellow viscous oil.

R$_f$ 0.48 (silica gel, 40% EtOAc in PE)

^{1}H NMR (500 MHz, $CDCl_3$) δ 3.30 (s, 1H), 2.87 (dd, *J* = 9.8, 6.0 Hz, 1H), 2.16 (s, 3H), 1.88 (dt, *J* = 10.7, 2.6 Hz, 1H), 1.78 – 1.67 (m, 3H), 1.61 – 1.48 (m, 3H), 1.35 – 1.26 (m, 1H), 1.27 – 1.16 (m, 2H), 1.01 (s, 3H).

^{13}C NMR (126 MHz, $CDCl_3$) δ 213.2, 80.8, 56.1, 51.1, 39.8, 39.0, 38.9, 38.0, 31.4, 27.4, 20.5.

HRMS (ESI−) *m*/z: calcd. for $C_{12}H_{19}O_4^-$ $[M+HCOO]^-$: 227.1289; found: 227.1287 (Δ = −0.88 ppm)

IR (ATR)/cm^{-1} 3444, 2933, 2687, 1699, 1457, 1357, 1171, 1089, 1058

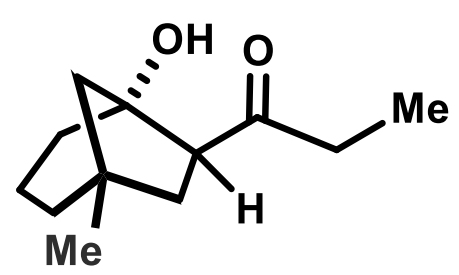

47

Radical-polar crossover product 47. Compound **47** was prepared from cyclopropyl silyl ether **S3** (0.23 mmol) and ethyl vinyl ketone (0.15 mmol) according to *Method C1*. Purification by flash column chromatography (silica gel, 10% to 60% EtOAc in PE) afforded diastereomerically pure **47** (19.4 mg, 0.099 mmol, 66%) as a yellow viscous oil.

R$_f$ 0.54 (silica gel, 40% EtOAc in PE)

^{1}H NMR (400 MHz, $CDCl_3$) δ 3.09 (s, 1H), 2.86 (dd, *J* = 9.7, 6.0 Hz, 1H), 2.45 (qd, *J* = 7.3, 4.4 Hz, 2H), 1.92 (dt, *J* = 10.6, 2.6 Hz, 1H), 1.80 – 1.63 (m, 3H), 1.62 – 1.46 (m, 3H), 1.34 – 1.26 (m, 1H), 1.26 – 1.14 (m, 2H), 1.03 (t, *J* = 7.2 Hz, 3H), 1.00 (s, 3H).

^{13}C NMR (126 MHz, $CDCl_3$) δ 215.7, 80.8, 55.4, 51.3, 40.1, 39.0, 38.9, 38.0, 37.4, 27.3, 20.5, 7.6.

HRMS (ESI−) *m*/z: calcd. for $C_{12}H_{19}O_2^-$ $[M-H]^-$: 195.1391; found: 195.1389, (Δ = −1.02 ppm)

IR (ATR)/cm^{-1} 3464, 2934, 2867, 1702, 1456, 1362, 1162, 1116, 1088, 1053

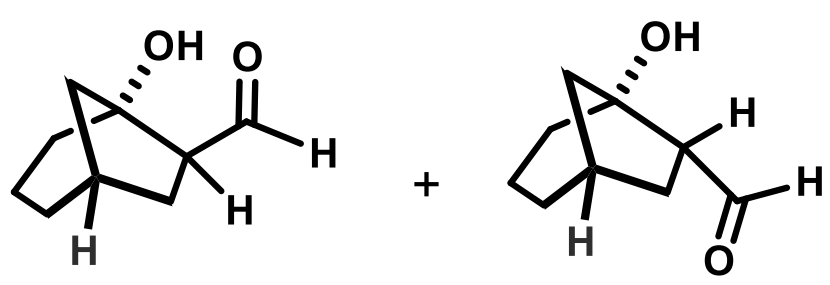

48a, *major* **48b**, *minor*

Radical-polar crossover product 48. Compound **48** was prepared from cyclopropyl silyl ether **1** (0.23 mmol) and acrolein (0.15 mmol) according to *Method C1*. Purification by flash column chromatography (silica gel, 20% to 70% EtOAc in PE) afforded diastereomers **48a** (14.8 mg, 0.096 mmol, 64%) and **48b** (3.7 mg, 0.024 mmol, 16%) as orange oils (combined yield 80%; 4:1 isolated dr).

R$_f$ 0.37 (silica gel, 40% EtOAc in PE)

^{1}H NMR (500 MHz, $CDCl_3$) δ 9.71 (d, *J* = 3.1 Hz, 1H), 2.68 – 2.58 (m, 1H), 2.38 – 2.30 (m, 1H), 2.28 (s, 1H), 2.23 – 2.16 (m, 1H), 1.95 (dddd, *J* = 10.7, 5.3, 3.1, 1.8 Hz, 1H), 1.83 – 1.75 (m, 1H), 1.77 – 1.68 (m, 1H), 1.68 – 1.51 (m, 3H), 1.51 – 1.43 (m, 1H), 1.43 – 1.29 (m, 2H). (**48a**)

^{13}C NMR (126 MHz, $CDCl_3$) δ 204.7, 81.7, 55.5, 44.8, 41.2, 34.0, 30.7, 29.0, 19.8. (**48a**)

HRMS (ESI−) *m*/z: calcd. for $C_9H_{13}O_2^-$ [M−H]$^-$: 153.0921; found: 153.0921, (Δ = 0 ppm)

IR (ATR)/cm^{-1} 3396, 2932, 2868, 2855, 1714, 1457, 1337, 1126, 1003, 984

*Only the major diastereomer, **48a**, was characterized due to stability issues of the minor diastereomer, **48b**.*

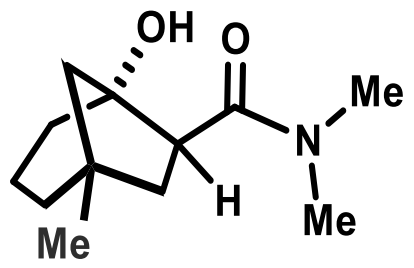

49

Radical-polar crossover product 49. Compound **49** was prepared from cyclopropyl silyl ether **S3** (0.30 mmol) and *N,N*-dimethylacrylamide (0.15 mmol) according to *Method C2*. Purification by flash column chromatography (silica gel, 40% to 100% EtOAc in PE) afforded diastereomerically pure **49** (11.1 mg, 0.053 mmol, 35%) as a yellow solid and recovered *N,N*-dimethylacrylamide (7.6 mg, 0.077 mmol, 51%).

R_f 0.30 (silica gel, 75% EtOAc in PE)

^{1}H NMR (500 MHz, $CDCl_3$) δ 4.91 (s, 1H), 3.01 (s, 3H), 2.94 (s, 3H), 2.78 (dd, *J* = 9.8, 5.9 Hz, 1H), 2.13 (dt, *J* = 10.7, 2.4 Hz, 1H), 1.84 (ddd, *J* = 13.2, 9.8, 2.2 Hz, 1H), 1.80 – 1.74 (m, 1H), 1.69 – 1.64 (m, 1H), 1.63 – 1.52 (m, 2H), 1.49 (ddd, *J* = 13.1, 6.0, 1.7 Hz, 1H), 1.33 – 1.28 (m, 1H), 1.27 – 1.20 (m, 2H), 1.01 (s, 3H).

^{13}C NMR (126 MHz, $CDCl_3$) δ 176.8, 80.0, 51.8, 46.3, 41.3, 38.8, 38.6, 38.2, 37.5, 35.5, 27.4, 20.5.

HRMS (ESI−) *m*/z: calcd. for $C_{13}H_{22}NO_4^-$ [M+HCOO]$^-$: 256.1554; found: 256.1553, (Δ = −0.39 ppm)

IR (ATR)/cm^{-1} 3401, 2946, 2926, 2902, 2862, 1617, 1455, 1412, 1166, 1141, 1058

MP 115 – 120 °C

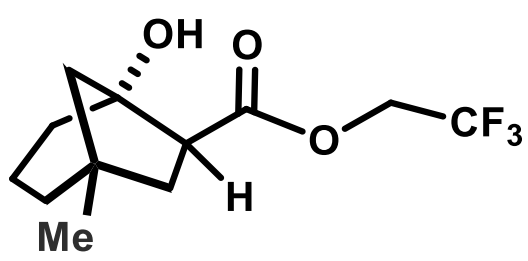

50

Radical-polar crossover product 50. Compound **50** was prepared from cyclopropyl silyl ether **S3** (0.23 mmol) and 2,2,2-trifluoroethyl acrylate (0.15 mmol) according to *Method C1*. Purification by flash column chromatography (silica gel, 10% to 60% EtOAc in PE) afforded diastereomerically pure **50** (18.0 mg, 0.068 mmol, 45%) as a transparent viscous oil.

R_f 0.67 (silica gel, 40% EtOAc in PE)

^{1}H NMR (500 MHz, $CDCl_3$) δ 4.59 – 4.36 (m, 2H), 2.80 (dd, *J* = 9.2, 5.6, 1H), 2.23 (s, 1H), 2.00 (dt, *J* = 10.7, 2.6 Hz, 1H), 1.83 (ddd, *J* = 13.5, 5.6, 1.8 Hz, 1H), 1.81 – 1.68 (m, 3H), 1.63 (td, *J* = 12.2, 5.2 Hz, 1H), 1.59 – 1.46 (m, 1H), 1.37 – 1.28 (m, 1H), 1.29 – 1.17 (m, 2H), 1.06 (s, 3H).

^{13}C NMR (126 MHz, $CDCl_3$) δ 174.2,123.2 (q, *J* = 277.2 Hz), 80.9, 60.51 (q, *J* = 36.4 Hz), 50.9, 50.2, 40.0, 39.6, 39.3, 37.8, 27.2, 20.5.

HRMS (ESI−) *m*/z: calcd. for $C_{12}H_{16}F_3O_3^-$ [M−H]$^-$: 265.1057; found: 265.1065, (Δ = 3.02 ppm)

IR (ATR)/cm^{-1} 3468, 2948, 2870, 1750, 1457, 1412, 1279, 1161, 1066

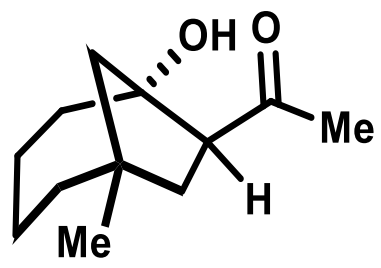

51

Radical-polar crossover product 51. Compound **51** was prepared from crude cyclopropyl silyl ether **S8** (0.23 mmol) and methyl vinyl ketone (0.15 mmol) according to *Method C1*. Purification by flash column chromatography (silica gel, 10% to 60% EtOAc in PE) afforded diastereomerically pure **51** (21.8 mg, 0.111 mmol, 74%) as a faint yellow viscous oil.

$\mathbf{R_f}$ 0.55 (silica gel, 40% EtOAc in PE)

^{1}H NMR (500 MHz, $CDCl_3$) δ 3.09 (dd, *J* = 9.6, 7.1 Hz, 1H), 2.63 (s, 1H), 2.19 (s, 3H), 2.01 – 1.92 (m, 3H), 1.78 – 1.66 (m, 4H), 1.64 – 1.56 (m, 4H), 1.45 – 1.39 (m, 1H), 1.08 (s, 3H).

^{13}C NMR (126 MHz, $CDCl_3$) δ 212.7, 83.5, 61.0, 51.4, 43.5, 43.4, 42.9, 39.2, 32.2, 31.8, 24.9, 23.5.

HRMS (ESI−) *m*/z: calcd. for $C_{12}H_{19}O_2^-$ [M−H]$^-$: 195.1391; found: 195.1392, (Δ = 0.51 ppm)

IR (ATR)/cm^{-1} 3455, 2924, 2863, 1702, 1458, 1358, 1170, 1082

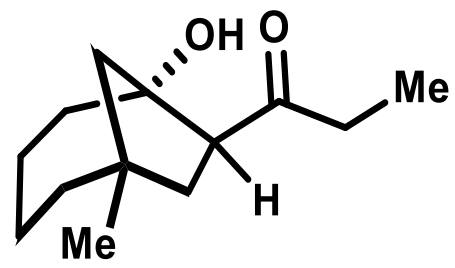

52

Radical-polar crossover product 52. Compound **52** was prepared from crude cyclopropyl silyl ether **S8** (0.23 mmol) and ethyl vinyl ketone (0.15 mmol) according to *Method C1*. Purification by flash column chromatography (silica gel, 10% to 60% EtOAc in PE) afforded diastereomerically pure **52** (19.6 mg, 0,093 mmol, 62%) as a yellow viscous oil.

$\mathbf{R_f}$ 0.59 (silica gel, 40% EtOAc in PE)

^{1}H NMR (500 MHz, $CDCl_3$) δ 3.10 (dd, *J* = 9.5, 7.1 Hz, 1H), 2.57 (s, 1H), 2.55 – 2.39 (m, 2H), 2.01 – 1.90 (m, 3H), 1.78 – 1.66 (m, 3H), 1.64 – 1.55 (m, 4H), 1.45 – 1.38 (m, 2H), 1.08 (s, 3H), 1.04 (t, *J* = 7.2 Hz, 3H).

^{13}C NMR (126 MHz, $CDCl_3$) δ 215.5, 83.7, 60.2, 51.6, 43.7, 43.3, 43.0, 39.3, 38.3, 31.8, 24.9, 23.6, 7.6.

HRMS (ESI−) *m*/z: calcd. for $C_{14}H_{23}O_4^-$ [M+HCOO]$^-$: 255.1602; found: 255.1604, (Δ = 0.78 ppm)

IR (ATR)/cm^{-1} 3473, 2924, 2863, 1704, 1458, 1362, 1117

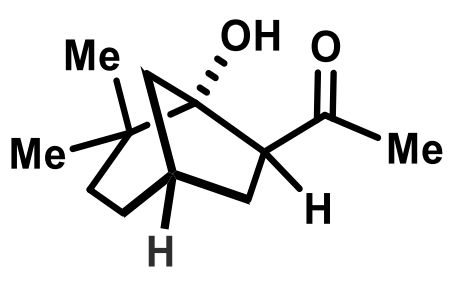

54

Radical-polar crossover product 54. Compound **54** was prepared from crude cyclopropyl silyl ether **S13** (0.30 mmol) and methyl vinyl ketone (0.15 mmol) according to *Method C1*. Purification by flash column chromatography (silica gel, 40% to 100% EtOAc in PE) afforded diastereomerically pure **54** (13.0 mg, 0.066 mmol, 44%) as a faint yellow viscous oil.

$\mathbf{R_f}$ 0.55 (silica gel, 40% EtOAc in PE)

^{1}H NMR (500 MHz, $CDCl_3$) δ 3.14 (dd, *J* = 9.5, 5.6 Hz, 1H), 2.38 (s, 1H), 2.19 (s, 3H), 2.12 – 2.06 (m, 1H), 1.75 – 1.71 (m, 1H), 1.65 (ddd, *J* = 11.3, 5.0, 1.7 Hz, 1H), 1.58 – 1.50 (m, 3H), 1.37 – 1.29 (m, 3H), 1.03 (s, 3H), 1.01 (s, 3H).

^{13}C NMR (126 MHz, $CDCl_3$) δ 213.1, 85.0, 51.7, 39.4, 39.1, 34.3, 33.7, 32.5, 31.9, 28.4, 24.7, 21.2.

HRMS (ESI−) *m*/z: calcd. for $C_{12}H_{19}O_2^-$ [M−H]$^-$: 195.1391; found: 195.1393, (Δ = 1.02 ppm)

IR (ATR)/cm^{-1} 3491, 2926, 2862, 1708, 1459, 1359, 1275, 1261, 1181

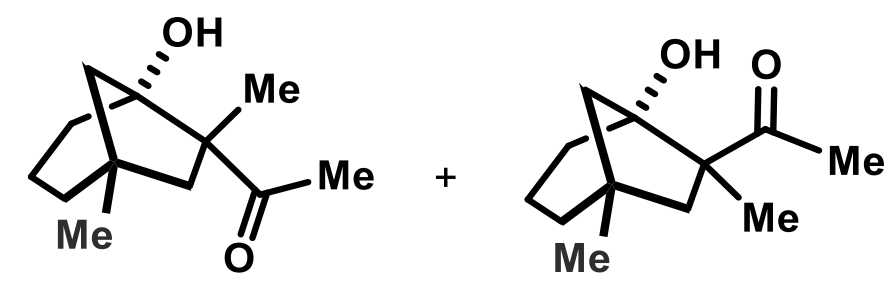

55a, *major* **55b**, *minor*

Radical-polar crossover product 55. Compound **55** was prepared from crude cyclopropyl silyl ether **S3** (0.23 mmol) and 3-methylbut-3-en-2-one (0.15 mmol) according to *Method C1*. Purification by flash column chromatography (silica gel, 10% to 60% EtOAc in PE) afforded **55a**, and **55b** (22.1 mg, 0.113 mmol, 75%, ca. 1.4:1 dr) as a yellow viscous oil.

R*f* 0.53 (silica gel, 40% EtOAc in PE)

^{1}H NMR (400 MHz, $CDCl_3$) δ 4.27 (s, 0.5H), 2.27 (dd, *J* = 13.8, 2.0 Hz, 0.5H), 2.24 – 2.14 (two s, 3H), 2.01 (s, 0.5H), 1.99 – 1.92 (m, 1H), 1.88 – 1.68 (m, 2H), 1.63 – 1.45 (m, 2H), 1.39 (dd, *J* = 14.2, 1.9 Hz, 0.5H), 1.32 – 1.17 (m, 7H), 1.02 – 0.97 (two s, 3H). (Summary of diastereomers)

^{13}C NMR (126 MHz, $CDCl_3$) δ 216.8, 213.5, 81.3, 80.4, 59.0, 56.9, 51.2, 50.5, 45.0, 44.8, 39.0, 38.5, 37.1, 37.0, 36.4, 35.4, 28.3, 28.2, 27.3, 27.1, 25.4, 20.4, 20.3, 16.5. (Summary of diastereomers)

HRMS (ESI−) *m*/z: calcd. for $C_{12}H_{19}O_2^-$ [M−H]$^-$: 195.1391; found: 195.1391, (Δ = 0 ppm)

IR (ATR)/cm^{-1} 3455, 2943, 2866, 1689, 1455, 1453, 1127, 1084, 1042

*Note: The relative configuration of the major diastereomer **55a** was assigned based on 2D NMR of the diastereomeric mixture (see SI, pp. **S146**-**S147**).*

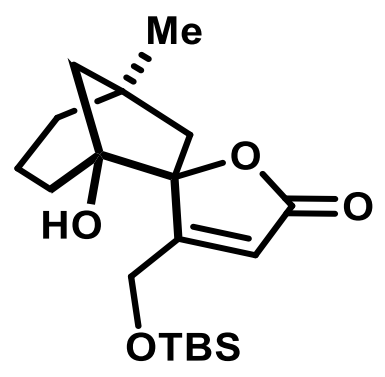

57

Radical-polar crossover product 57. Compound **57** was prepared from cyclopropyl silyl ether **S3** (0.15 mmol) and butenolide **S4** (0.10 mmol) (prepared according to the procedure of Husinec *et al.*[11]) according to *Method B2*. Purification by flash column chromatography (silica gel, 5% to 40% EtOAc in PE) afforded diastereomerically pure **57** (28.6 mg, 0.081 mmol, 81%) as a white solid.

R*f* 0.36 (silica gel, 30% EtOAc in PE)

^{1}H NMR (400 MHz, $CDCl_3$) δ 6.17 (t, *J* = 2.0 Hz, 1H), 4.54 (d, *J* = 2.0 Hz, 2H), 2.14 (dt, *J* = 10.9, 2.5 Hz, 1H), 2.04 (dd, *J* = 15.2, 1.9 Hz, 1H), 1.93 – 1.76 (m, 3H), 1.74 – 1.66 (m, 1H), 1.44 – 1.35 (m, 4H), 1.11 (s, 3H), 0.93 (s, 9H), 0.11 (s, 6H).

^{13}C NMR (101 MHz, $CDCl_3$) δ 171.2, 171.0, 119.3, 97.3, 82.3, 60.3, 52.1, 44.1, 38.0, 36.9, 33.2, 27.5, 25.9, 19.6, 18.4, -5.4, -5.3.

HRMS (ESI+) *m*/z: calcd. for $C_{19}H_{33}O_4Si^+$ $[M+H]^+$: 353.2143; found: 353.2140, (Δ = −0.85 ppm)

IR (ATR)/cm^{-1} 3432, 2951, 2929, 2856, 1746, 1461, 1255, 1137, 1092, 975, 951, 836, 777

MP 127 °C

The relative configuration of **57** *was assigned based on X-ray diffraction (see SI, pp.* ***S48-S58****).*

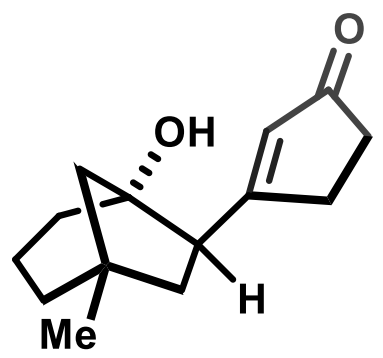

58

Radical-polar crossover product 58. Compound **58** was prepared from crude cyclopropyl silyl ether **S3** (0.23 mmol) and 3-vinylcyclopent-2-en-1-one (0.15 mmol (prepared according to the procedure of Kawabata *et al*.[12]) according to *Method C2*. Purification by flash column chromatography (silica gel, 40% to 100% EtOAc in PE) afforded diastereomerically pure **58** (28.1 mg, 0.128 mmol, 85%) as a faint yellow solid.

R_f 0.38 (silica gel, 75% EtOAc in PE)

^{1}H NMR (400 MHz, $CDCl_3$) δ 5.98 (app. t, *J* = 1.8 Hz, 1H), 2.83 (dd, *J* = 9.2, 5.6 Hz, 1H), 2.80 – 2.73 (m, 1H), 2.70 – 2.61 (m, 1H), 2.42 – 2.37 (m, 2H), 1.84 – 1.55 (m, 7H), 1.37 – 1.22 (m, 3H), 1.05 (s, 3H).

^{13}C NMR (126 MHz, $CDCl_3$) δ 210.4, 186.7, 130.5, 81.7, 51.1, 50.1, 42.8, 40.7, 38.8, 38.0, 35.6, 32.3, 27.5, 20.6.

HRMS (ESI−) *m*/z: calcd. for $C_{14}H_{19}O_2^-$ $[M-H]^-$: 219.1391; found: 219.1393, (Δ = 0.91 ppm)

IR (ATR)/cm^{-1} 3405, 2940, 2866, 1705, 1673, 1603, 1457, 1276, 1259, 1169, 1084

MP 80 – 85 °C

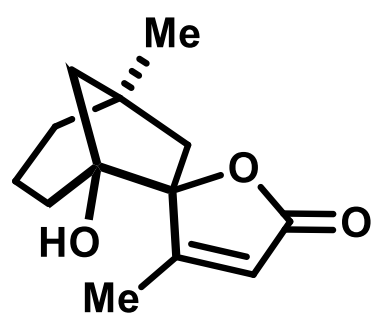

60

Radical-polar crossover product 60. Compound **60** was prepared from crude cyclopropyl silyl ether **S3** (0.23 mmol) and 4-methyl-5-methylenefuran-2(5H)-one (0.15 mmol) (prepared according to the procedure of Argade and Haval[13]) according to *Method B2*. The obtained crude was stirred in silica/DCM overnight. Subsequently, purification by flash column chromatography (silica gel, 10% to 60% EtOAc in PE) afforded diastereomerically pure **60** (24.0 mg, 0.108 mmol, 72%) as an off-white viscous oil.

R_f 0.28 (silica gel, 30% EtOAc in PE)

^{1}H NMR (400 MHz, $CDCl_3$) δ 5.85 (app. q, *J* = 1.5 Hz, 1H), 2.26 (s, 1H), 2.22 (d, *J* = 1.5 Hz, 3H), 2.16 (dt, *J* = 10.8, 2.4 Hz, 1H), 2.09 (dd, *J* = 15.2, 1.9 Hz, 1H), 1.87 – 1.73 (m, 5H), 1.50 – 1.42 (m, 1H), 1.42 – 1.35 (m, 2H), 1.12 (s, 3H).

^{13}C NMR (126 MHz, $CDCl_3$) δ 171.5, 167.1, 120.9, 98.8, 82.6, 52.3, 43.2, 38.4, 36.9, 32.8, 27.6, 19.7, 16.2.

HRMS (ESI−) *m*/z: calcd. for $C_{13}H_{17}O_3^-$ $[M-H]^-$: 221.1183; found: 221.1186, (Δ = 1.36 ppm)

IR (ATR)/cm^{-1} 3444, 2941, 2863, 1751, 1453, 1380, 1337, 1259, 1134, 1094, 962

*The relative configuration of **60** was assigned by analogy to a structurally closely related compound **57** whose relative configuration was established by X-ray diffraction. (see SI, pp. **S48**-**S58**).*

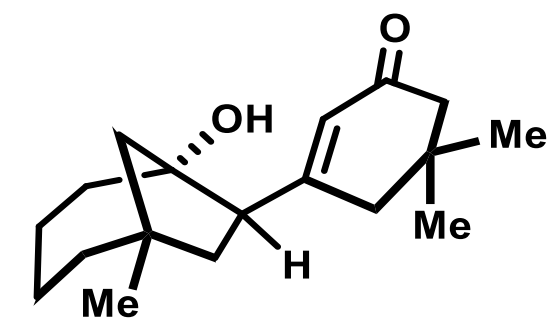

61

Radical-polar crossover product 61. Compound **61** was prepared from crude cyclopropyl silyl ether **S8** (0.23 mmol) and 5,5-dimethyl-3-vinylcyclohex-2-en-1-one (0.15 mmol) (prepared according to the procedure of Kawabata *et al*.[12]) according to *Method C2*. Purification by flash column chromatography (silica gel, 40% to 100% EtOAc in PE) afforded diastereomerically pure **61** (39.4 mg, 0.143 mmol, 95%) as a yellow viscous oil.

R$_f$ 0.47 (silica gel, 75% EtOAc in PE)

^{1}H NMR (500 MHz, $CDCl_3$) δ 5.91 (s, 1H), 2.80 (t, *J* = 8.6 Hz, 1H), 2.39 – 2.27 (m, 2H), 2.21 (s, 2H), 2.01 (dd, *J* = 13.0, 1.7 Hz, 1H), 1.89 – 1.82 (m, 2H), 1.79 – 1.70 (m, 2H), 1.66 – 1.55 (m, 5H), 1.46 – 1.42 (m, 2H), 1.11 (s, 3H), 1.03 (s, 3H), 1.03 (s, 3H).

^{13}C NMR (126 MHz, $CDCl_3$) δ 200.5, 166.5, 125.6, 84.4, 57.5, 51.5, 51.4, 45.1, 44.6, 44.5, 42.8, 38.8, 33.9, 31.7, 28.5, 24.8, 23.8.

HRMS (ESI−) *m*/z: calcd. for $C_{18}H_{27}O_2^-$ [M−H]$^-$: 275.2017; found: 275.2019, (Δ = 0.73 ppm)

IR (ATR)/cm^{-1} 3446, 2946, 2925, 2865, 1658, 1459, 1368, 1277, 1260, 1077

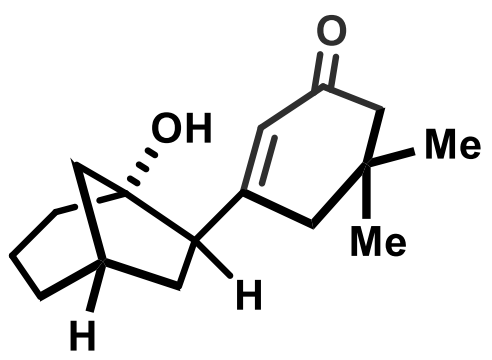

62

Radical-polar crossover product 62. Compound **62** was prepared from crude cyclopropyl silyl ether **1** (0.23 mmol) and 5,5-dimethyl-3-vinylcyclohex-2-en-1-one (0.15 mmol) (prepared according to the procedure of Kawabata *et al*.[12]) according to *Method C2*. Purification by flash column chromatography (silica gel, 40% to 100% EtOAc in PE) afforded diastereomerically pure **62** (36.5 mg, 0.138 mmol, 92%) as a yellow viscous oil.

R$_f$ 0.42 (silica gel, 75% EtOAc in PE)

^{1}H NMR (400 MHz, $CDCl_3$) δ 5.94 (s, 1H), 2.53 (dd, *J* = 9.2, 5.7 Hz, 1H), 2.37 – 2.28 (m, 2H), 2.29 – 2.19 (m, 3H), 2.03 – 1.91 (m, 2H), 1.76 – 1.62 (m, 3H), 1.64 – 1.41 (m, 3H), 1.40 – 1.29 (m, 2H), 1.06 (s, 3H), 1.02 (s, 3H).

^{13}C NMR (126 MHz, $CDCl_3$) 200.3, 167.2, 125.8, 81.6, 52.4, 51.3, 44.84, 44.82, 41.9, 34.5, 33.8, 33.4, 30.8, 29.2, 27.9, 20.0.

HRMS (ESI−) *m*/z: calcd. for $C_{16}H_{23}O_2^-$ [M−H]$^-$: 247.1704; found: 247.1703, (Δ = -0.40 ppm)

IR (ATR)/cm^{-1} 3432, 2934, 2869, 1658, 1461, 1379, 1276, 1261, 1130

*Trace amounts of an epimer of **62** were observed after prolonged storage of the neat compound.*

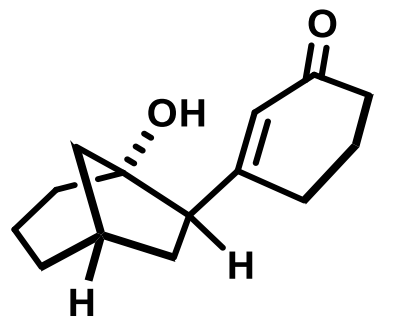

63

Radical-polar crossover product 63. Compound **63** was prepared from cyclopropyl silyl ether **1** (0.38 mmol) and 3-vinylcyclohex-2-en-1-one (0.25 mmol) (prepared according to the procedure of Krasutsky *et al.*[9]) according to *Method B2* (D_2O instead of HFIP). Purification by flash column chromatography (silica gel, 40% to 100% EtOAc in PE) afforded diastereomerically pure **63** (22.3 mg, 0.101 mmol, 40%) as a white viscous oil.

R_f 0.35 (silica gel, 75% EtOAc in PE)

^{1}H NMR (400 MHz, $CDCl_3$) δ 5.94 (s, 1H), 2.54 (dd, J = 9.2, 5.8 Hz, 1H), 2.51 – 2.41 (m, 1H), 2.43 – 2.31 (m, 3H), 2.30 – 2.24 (m, 1H), 2.06 – 1.92 (m, 3H), 1.96 – 1.89 (m, 1H), 1.79 – 1.53 (m, 4H), 1.51 – 1.30 (m, 4H).

^{13}C NMR (126 MHz, $CDCl_3$) δ 200.1, 170.0, 126.5, 81.5, 52.5, 44.7, 41.6, 37.6, 34.6, 33.3, 31.2, 30.7, 22.8, 20.0.

HRMS (ESI+) m/z: calcd. for $C_{14}H_{21}O_2^+$ [M+H]$^+$: 221.1536; found: 221.1537, (Δ = 0.45 ppm)

IR (ATR)/cm^{-1} 3415, 2931, 2868, 1708, 1652, 1604, 1455, 1380, 1327, 1255, 1192, 1126, 1105

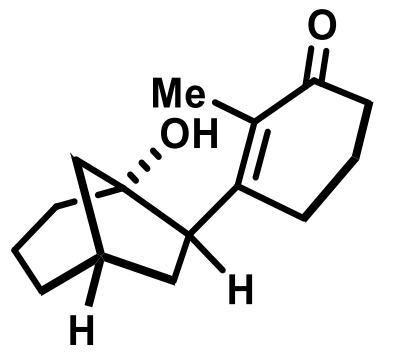

64

Radical-polar crossover product 64. Compound **64** was prepared from crude cyclopropyl silyl ether **1** (0.23 mmol) and 2-methyl-3-vinylcyclohex-2-en-1-one (0.15 mmol) (prepared according to the procedure of Kawabata *et al.*[12]) according to *Method C2*. Purification by flash column chromatography (silica gel, 40% to 100% EtOAc in PE) afforded diastereomerically pure **64** (21.4 mg, 0.092 mmol, 61%) as a faint-yellow viscous oil.

R_f 0.38 (silica gel, 75% EtOAc in PE)

^{1}H NMR (500 MHz, $CDCl_3$) δ 3.22 (dd, J = 9.2, 6.2 Hz, 1H), 2.51 – 2.28 (m, 5H), 2.09 – 1.96 (m, 2H), 1.98 – 1.86 (m, 2H), 1.83 (t, J = 1.7 Hz, 3H), 1.82 – 1.61 (m, 5H), 1.53 – 1.45 (m, 1H), 1.43 – 1.35 (m, 2H).

^{13}C NMR (126 MHz, $CDCl_3$) δ 199.6, 160.1, 132.8, 80.9, 47.8, 45.7, 43.0, 38.2, 34.1, 33.6, 31.0, 28.3, 22.7, 20.5, 11.6.

HRMS (ESI+) m/z: calcd. for $C_{15}H_{23}O_2^+$ [M+H]$^+$: 235.1693; found: 235.1684, (Δ = −3.83 ppm)

IR (ATR)/cm^{-1} 3432, 2931, 2869, 1647, 1605, 1358, 1276, 1261, 1127

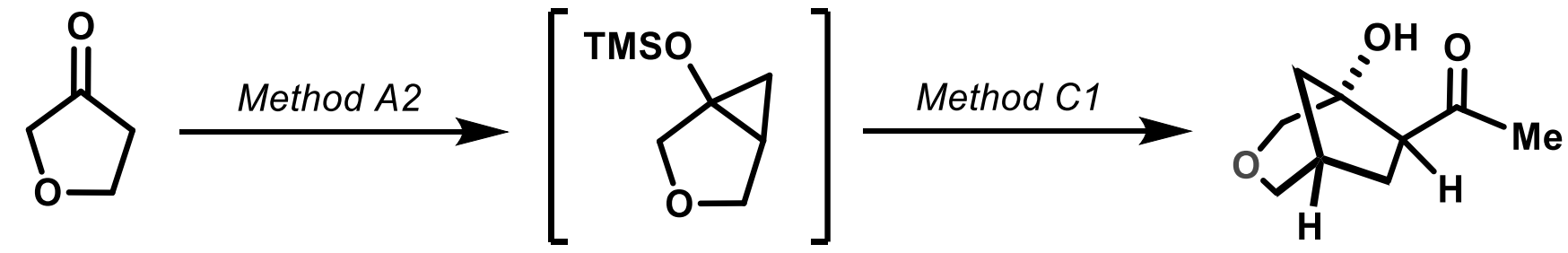

S19 **S20** (*not isolated*) **53**

Radical-polar crossover product 53. Compound **53** was prepared from crude cyclopropyl silyl ether **S20** **(**0.29 mmol) (prepared from **S19** according to *Method A2, 5 equiv. Et_2Zn, 2 days*) and methyl vinyl ketone (0.15 mmol) according to *Method C1*. Purification by flash column chromatography (silica gel, 10% to 60% EtOAc in PE) afforded diastereomerically pure **53** (11.7 mg, 0.069 mmol, 46%) as a yellow viscous oil.

R_f 0.40 (silica gel, 40% EtOAc in PE)

^{1}H NMR (500 MHz, $CDCl_3$) δ 3.64 (dd, *J* = 10.2, 2.9 Hz, 1H), 3.60 (dt, *J* = 10.7, 2.4 Hz, 1H), 3.42 (d, *J* = 9.0 Hz, 1H), 3.40 (d, *J* = 7.3 Hz, 1H), 3.08 (dd, *J* = 9.9, 6.0 Hz, 1H), 2.20 (s, 3H), 2.19 – 2.11 (m, 3H), 2.04 (ddd, *J* = 12.4, 9.8, 2.2 Hz, 1H), 1.96 – 1.89 (m, 1H), 1.61 – 1.58 (m, 1H).

^{13}C NMR (126 MHz, $CDCl_3$) δ 213.3, 77.5, 72.2, 53.2, 42.1, 34.6, 32.2, 31.0. (One signal overlaps with the residual solvent signal)

HRMS (ESI−) *m/z*: calcd. for $C_{10}H_{15}O_5^-$ $[M+HCOO]^-$: 215.0925; found: 215.0927, (Δ = 0.93 ppm)

IR (ATR)/cm^{-1} 3416, 2952, 2853, 1706, 1357, 1276, 1168, 1084

*The crude cyclopropyl silyl ether **S20** was used directly in the reaction due to stability issues; its presence was confirmed by HRMS,* (ESI−) m/z: calcd. for $C_8H_{15}O_2Si^-$ $[M-H]^-$: 171.0847; found: 171.0855, (Δ = 4.68 ppm).

Late-stage Diversification

Synthesis and Characterization of Compounds S22, 65, 68 and 69

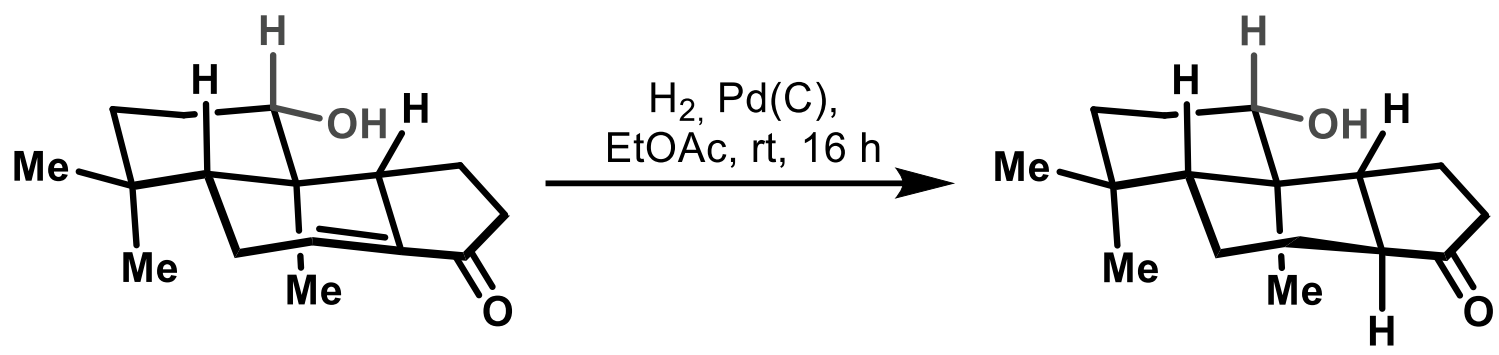

S21 **S22**, quant.

Ketone S22. A solution of enone **S21** (447 mg, 1.80 mmol) (prepared according to the procedure of Tanino *et al.*[14]) and Pd/C (45 mg, 10 wt%) in EtOAc (10 mL) was stirred under H_2 atmosphere (balloon) at rt for 16 h. The reaction mixture was then filtered through Celite®, washed with EtOAc, and concentrated under reduced pressure to afford ketone **S22** (450 mg, 1.80 mmol, quant.) as a viscous oil.

^{1}H NMR (400 MHz, $CDCl_3$) δ 3.44 (dd, *J* = 11.2, 4.6 Hz, 1H), 2.38 – 2.28 (m, 1H), 2.21 – 2.10 (m, 2H), 2.09 – 2.01 (m, 1H), 1.86 (tdd, *J* = 11.8, 4.9, 1.5 Hz, 1H), 1.79 – 1.58 (m, 5H), 1.47 – 1.39 (m, 2H), 1.34 (td, *J* = 12.9, 4.1 Hz, 2H), 1.20 (d, *J* = 10.5 Hz, 1H), 1.14 – 1.06 (m, 1H), 0.93 (s, 3H), 0.86 (s, 3H), 0.84 (s, 3H).

^{13}C NMR (126 MHz, $CDCl_3$) δ 219.4, 80.0, 55.6, 54.4, 50.1, 42.6, 40.3, 37.6, 33.3, 32.9, 29.1, 25.8, 24.2, 21.5, 21.5, 8.5.

HRMS (ESI−) *m/z*: calcd. for $C_{18}H_{29}O_4^-$ $[M+CH_3COO]^-$: 309.2071; found: 309.2081, (Δ = 3.23 ppm)

IR (ATR)/cm^{-1} 3467, 2935, 2867, 1732, 1460, 1370, 1275, 1265, 1163

*The analytical data (^{1}H, ^{13}C NMR) of starting material **S21** were in agreement with those previously described.[14] See SI, pp. **S130** for ^{1}H- and ^{13}C NMR.*

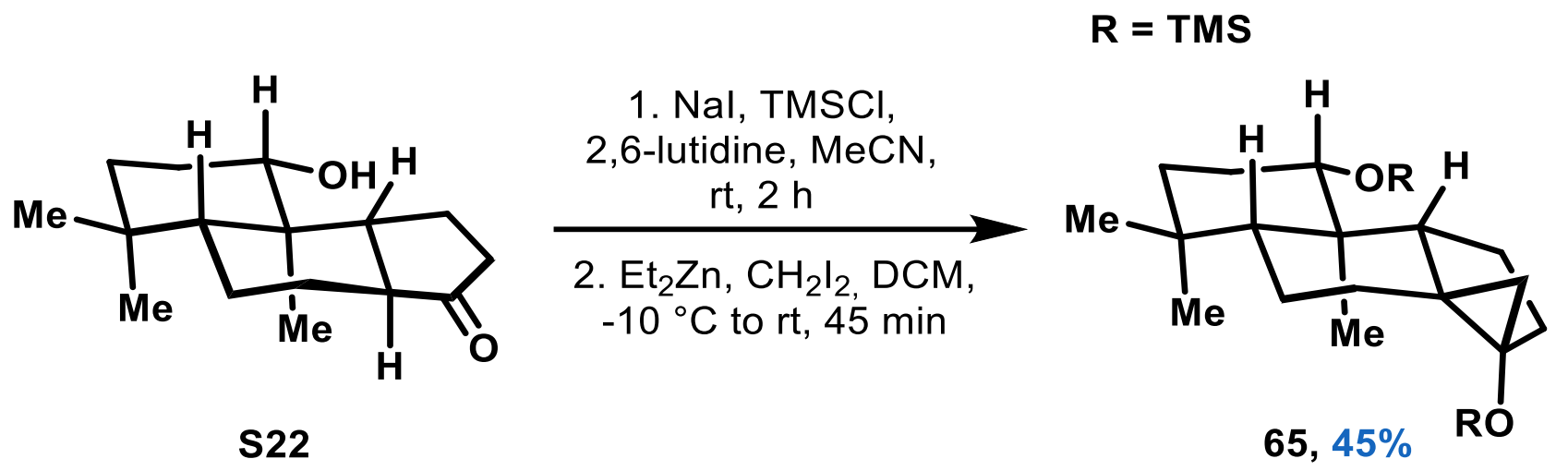

Cyclopropyl silyl ether 65. Under an inert atmosphere, 2,6-lutidine (0.70 mL, 6.00 mmol) and TMSCl (0.57 mL, 4.50 mmol) was added dropwise to a solution of ketone **S22** (375 mg, 1.50 mmol), NaI (675 mg, 4.50 mmol), and dry MeCN (10 mL). After stirring at rt for 2 h, Et_3N (1 mL), PE (10 mL), sat. aq. $NaHCO_3$ (10 mL) was added. The organic layer was then separated, and the aqueous layer was further extracted with EtOAc (3 x 5 mL). The combined organic layers were then dried over Na_2SO_4, filtered, and concentrated under reduced pressure. The obtained crude was used in the next step without further purification.

Under an inert atmosphere, Et_2Zn (4.00 mL, 1 M in *n*-hexane, 4.00 mmol) was added to a solution of the resulting silyl enol ether (ca. 592 mg, 1.50 mmol) and dry DCM (15 mL) at −10 °C. Then, CH_2I_2 (0.64 mL, 8.00 mmol) was added dropwise. The solution was then stirred at rt for 45 min, after which sat. aq. NH_4Cl was added to the mixture. The organic layer was then separated, and the aqueous layer was further extracted with DCM (3 x 10 mL). The combined organic layers were then dried over $MgSO_4$, filtered, and concentrated under reduced pressure. Purification by flash column chromatography (silica gel, 10% EtOAc in PE) of the obtained crude afforded **65** (275 mg, 0.68 mmol, 45% over two steps) as a white solid.

R_f 0.69 (silica gel, 10% EtOAc in PE)

^{1}H NMR (400 MHz, $CDCl_3$) δ 3.29 (dd, *J* = 11.5, 3.7 Hz, 1H), 2.25 (dd, *J* = 13.9, 7.7 Hz, 1H), 1.84 – 1.72 (m, 2H), 1.71 – 1.60 (m, 2H), 1.60 – 1.54 (m, 1H), 1.50 (d, *J* = 8.2 Hz, 1H), 1.44 – 1.24 (m, 5H), 1.07 – 0.97 (m, 1H), 0.90 (s, 3H), 0.86 (s, 3H), 0.83 (s, 3H), 0.82 – 0.78 (m, 1H), 0.49 (d, *J* = 4.7 Hz, 1H), 0.23 (dd, *J* = 4.8, 1.8 Hz, 1H), 0.12 (s, 9H), 0.08 (s, 9H).

^{13}C NMR (126 MHz, $CDCl_3$) δ 83.6, 70.3, 54.1, 52.9, 42.5, 40.3, 35.5, 33.7, 33.3, 30.6, 29.0, 28.5, 24.6, 23.0, 22.6, 21.2, 9.2, 1.3, 0.7.

HRMS (ESI−) *m/z*: calcd. for $C_{23}H_{43}O_2Si_2^-$ [M−H]$^-$: 407.2807; found: 407.2803, (Δ = −0.98 ppm)

IR (ATR)/cm^{-1} 2950, 2900, 2864, 1439, 1354, 1341, 1250, 1223, 1106, 1079,1052, 935, 848

MP 60 – 65 °C

The relative configuration of ***65*** *was assigned based on 2D NMR (See SI, pp.* ***S152****).*

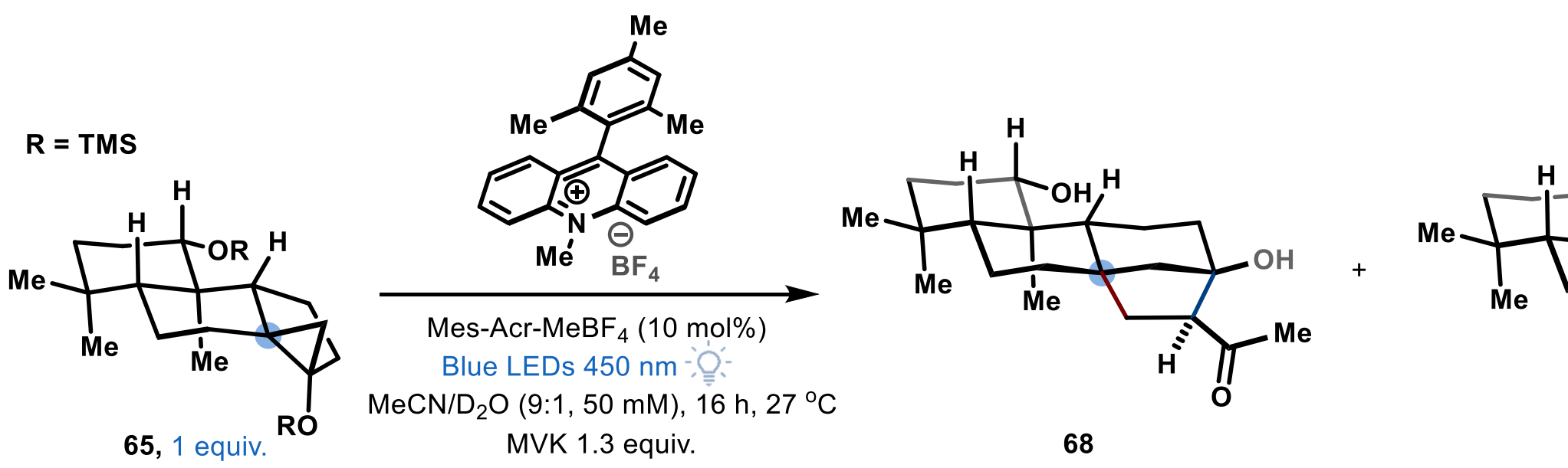

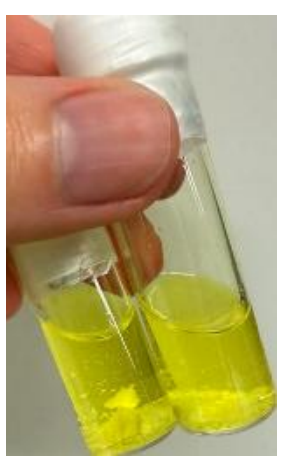

entry[a]	deviation	yield[b] 68 + 69	dr
1	none	71%	1.6:1
2	MeCN/D_2O/DCE (9:2:1)	57%	1.7:1
3	MeCN/D_2O/DCE (8:1:1)	62%	1.5:1
4	MeCN/D_2O/DCE (8:1:1), 40 °C	43%	1.4:1

[a] *0.1 mmol scale.* [b] *Isolated yield.*

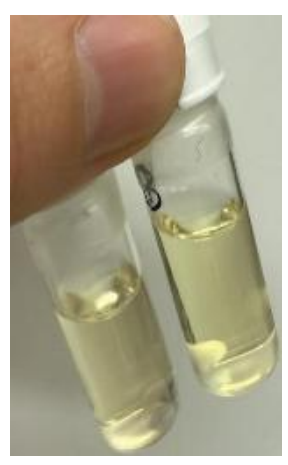

Figure S10. Complete reaction optimization of the radical-polar crossover reaction to afford the carbocyclic frameworks, *ent*-phyllocladane (**68**) and *ent*-kaurene (**69**), from **65**.

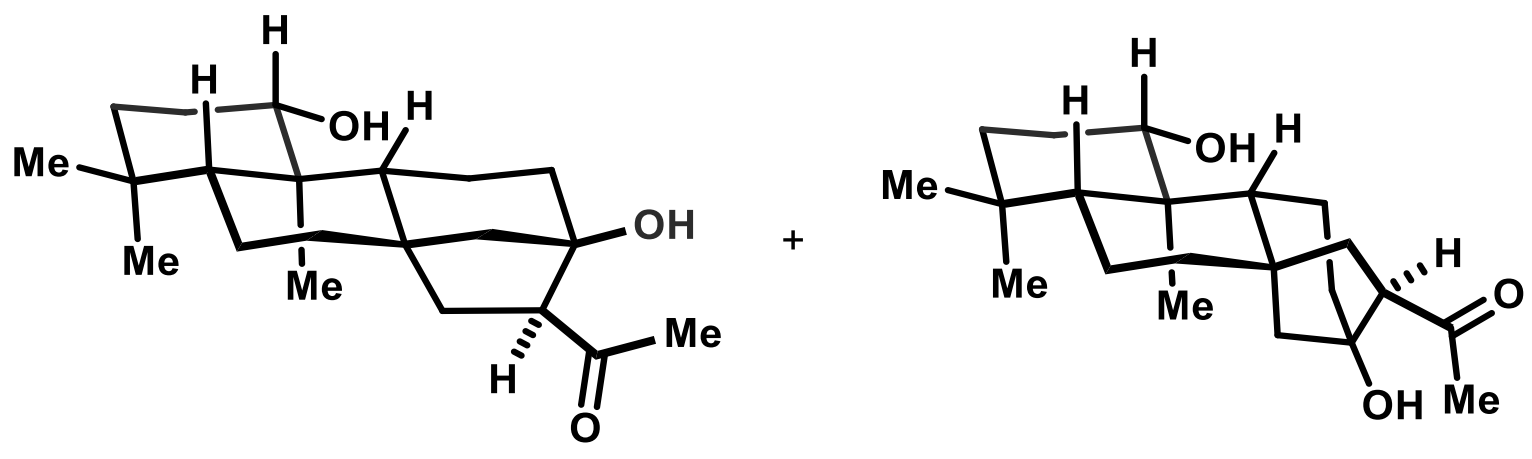

68 **69**

Radical-polar crossover products 68 and **69.** Cyclopropyl silyl ether **65** (0.12 mmol), Mes-Acr-$MeBF_4$ (10 mol%), and $LiBF_4$ (50 mol%) were dissolved in MeCN/D_2O (9:1, 50 mM). The reaction mixture was degassed for 5 min, after which methyl vinyl ketone (0.16 mmol) was added. The resulting solution was irradiated with 450 nm LEDs for 16 h. Upon completion, the reaction mixture was concentrated under reduced pressure. Purification by flash column chromatography (silica gel, 50% EtOAc in PE) of the obtained crude afforded an inseparable diastereomeric mixture of **68**, and **69** (29.0 mg, 0.087 mmol, 71%, ca. 1.6:1 dr) as an off-white solid.

$\mathbf{R_f}$ 0.35 (silica gel, 50% EtOAc in PE)

^{1}H NMR (500 MHz, $CDCl_3$) δ 3.32 (m, 1H), 3.08 (m, 0.5H), 2.85 (m, 1H), 2.44 (app. dtd, *J* = 14.1, 5.0, 1.7 Hz, 0.5H), 2.30 (app. ddd, *J* = 13.6, 9.9, 2.3 Hz, 0.5H), 2.16 – 2.15 (two s, 3H), 1.90 (app. dd, *J* = 10.8, 3.1 Hz, 1H), 1.81 – 1.71 (m, 1H), 1.69 – 1.58 (m, 3H), 1.58 – 1.47 (m, 5H), 1.42 – 1.32 (m, 3H), 1.32 – 1.23 (m, 2.5H), 1.22 – 1.12 (m, 2H), 1.09 (s, 1.5H), 0.99 (s, 1.5H), 0.85 – 0.82 (two s, 3H), 0.81 – 0.78 (two s, 3H), 0.78 – 0.68 (m, 1H). (Summary of diastereomers)

^{13}C NMR (126 MHz, $CDCl_3$) δ 213.6, 213.2, 82.1, 80.9 (two overlapping signals), 79.0, 56.6, 56.2, 55.42, 55.40, 55.0, 54.8, 54.7, 45.4, 44.9, 44.22, 44.16, 43.9, 43.1, 42.0, 41.2, 39.9, 39.84, 39.78, 39.0, 37.2, 33.4, 33.3, 33.1, 31.6, 31.4, 30.2, 29.7, 23.5, 22.0, 21.7, 21.6, 21.4, 20.8, 19.7, 13.6, 10.8. (Summary of diastereomers)

HRMS (ESI−) *m*/*z*: calcd. for $C_{21}H_{33}O_3^-$ [M−H]$^-$: 333.2435; found: 333.2437, (Δ = 0.60 ppm)

IR (ATR)/cm^{-1} 3448, 2924, 2852, 1700, 1458, 1360, 1176, 1017

MP 53 – 58 °C

*The relative configuration of **68** and **69** was assigned based on 2D NMR of the products in the subsequent step, **S23a** and **S23b** (see SI, pp. **S153**-**S156**).*

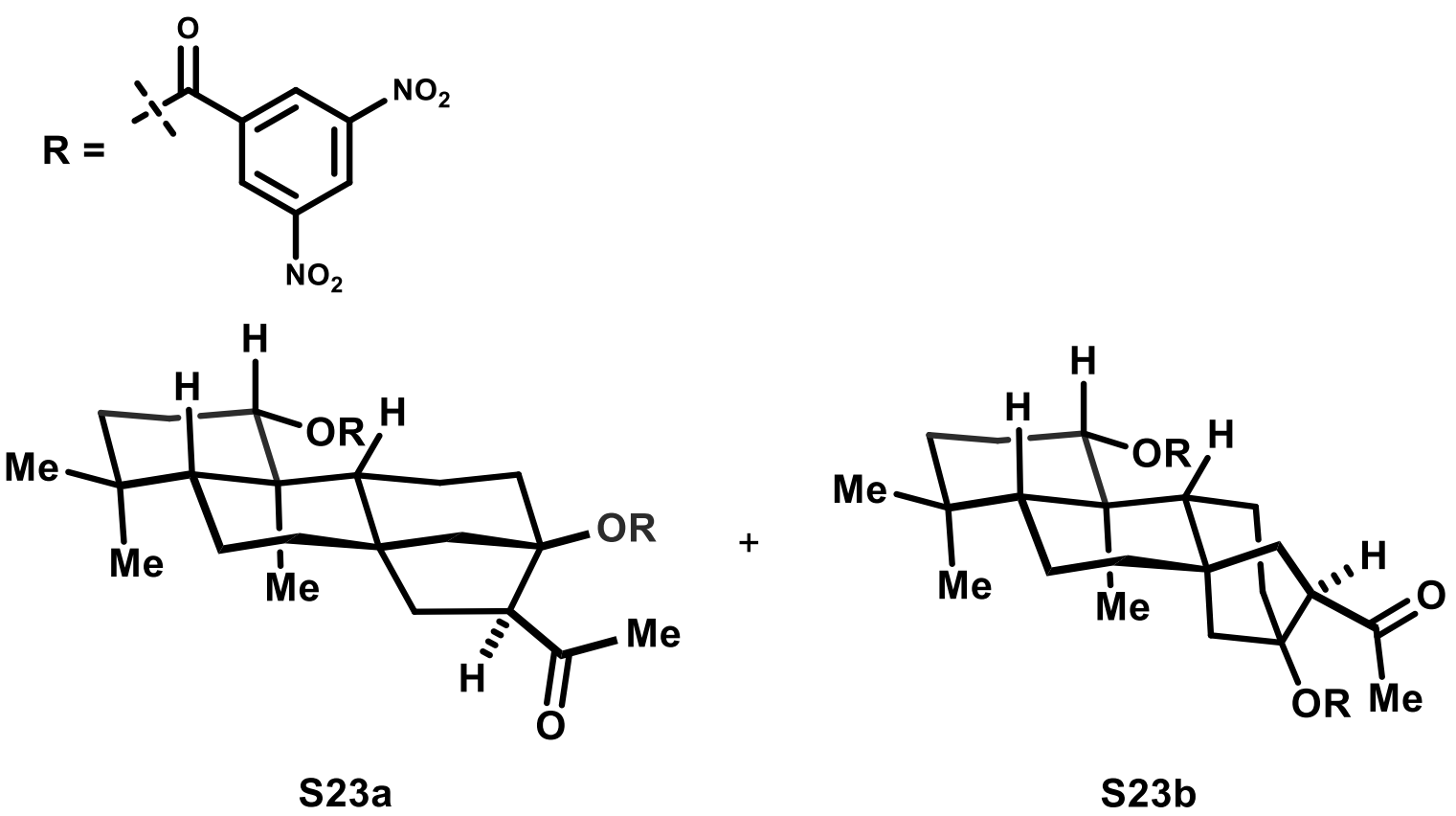

S23a **S23b**

Dibenzoyl products S23a and **S23b.** 4-dimethylaminopyridine (5.5 mg, 0.05 mmol) and 3,5-dinitrobenzoyl chloride (27 mg, 0.12 mmol) was added portion wise to a solution of diol **68**/**69** (30 mg, 0.09 mmol), Et_3N

(3.8 μL, 0.27 mmol), and DCM (1 mL). The resulting mixture was then stirred at rt for 1 h, after which sat. aq. $NaHCO_3$ (1 mL) was added to the mixture. The organic layer was separated, and the aqueous layer was extracted further with DCM (3 x 1 mL). The combined organic layers were washed with brine (2 mL) and then dried over $MgSO_4$, filtered, concentrated under reduced pressure. Purification by preparative thin-layer chromatography (10% EtOAc in toluene) of the obtained crude afforded diastereomers **S23a** (24.0 mg, 33.3 μmol, 37%) and **S23b** (14.6 mg, 20.2 μmol, 22%) as transparent viscous oils (combined yield 59%; ca. 1.6:1 dr).

R_f 0.73 (**S23a**); 0.65 (**S23b**) (silica gel, 10% EtOAc in toluene)

^{1}H NMR (500 MHz, $CDCl_3$) δ 9.25 (t, *J* = 2.1 Hz, 1H), 9.16 (t, *J* = 2.1 Hz, 1H), 9.12 (d, *J* = 2.1 Hz, 2H), 8.98 (d, *J* = 2.2 Hz, 2H), 4.94 (dd, *J* = 10.0, 5.8 Hz, 1H), 3.59 (dd, *J* = 9.5, 6.2 Hz, 1H), 2.48 – 2.40 (m, 1H), 2.38 (dd, *J* = 11.0, 3.3 Hz, 1H), 2.23 (s, 3H), 2.11 – 2.05 (m, 1H), 1.86 – 1.80 (m, 3H), 1.77 (d, *J* = 10.9 Hz, 1H), 1.71 – 1.65 (m, 4H), 1.61 – 1.57 (m, 1H), 1.51 – 1.43 (m, 5H), 1.35 (s, 3H), 1.03 (d, *J* = 10.6 Hz, 1H), 0.94 (s, 3H), 0.91 (s, 3H). (**S23a**)

^{1}H NMR (500 MHz, $CDCl_3$) δ 9.22 (t, *J* = 2.1 Hz, 1H), 9.19 (t, *J* = 2.1 Hz, 1H), 9.11 (d, *J* = 2.1 Hz, 2H), 9.04 (d, *J* = 2.2 Hz, 2H), 4.96 (dd, *J* = 10.8, 4.8 Hz, 1H), 3.48 (dd, *J* = 9.3, 6.0 Hz, 1H), 2.58 (d, *J* = 11.0 Hz, 1H), 2.20 (s, 3H), 2.15 – 2.09 (m, 2H), 2.07 – 2.00 (m, 2H), 1.95 – 1.88 (m, 1H), 1.87 – 1.79 (m, 1H), 1.75 – 1.67 (m, 4H), 1.62 (dd, *J* = 13.5, 6.0 Hz, 2H), 1.55 – 1.52 (m, 2H), 1.50 (s, 3H), 1.46 (dd, *J* = 13.7, 4.3 Hz, 1H), 1.38 (d, *J* = 7.9 Hz, 1H), 0.99 – 0.96 (m, 1H), 0.94 (s, 3H), 0.93 (s, 3H). (**S23b**)

^{13}C NMR (126 MHz, $CDCl_3$) δ 209.0, 162.0, 161.9, 149.0, 148.7, 134.64, 134.61, 129.5, 129.4, 122.6, 122.3, 88.7, 85.9, 55.5 (two overlapping signals), 55.1, 51.8, 43.0, 43.0, 40.5, 38.9, 37.3, 35.8, 33.1, 33.0, 31.6, 25.0, 23.0, 21.6, 19.5, 12.3. (**S23a**)

^{13}C NMR (126 MHz, $CDCl_3$) δ 209.3, 162.0, 161.9, 149.0,148.7, 134.7, 134.6, 129.6, 129.3, 122.6, 122.4, 90.5, 87.4, 55.2, 54.3, 53.3, 44.3, 44.2, 43.8, 42.4, 41.3, 39.2, 34.8, 33.3, 33.2, 31.7, 25.4, 22.2, 21.6, 20.5, 14.9. (**S23b**)

HRMS (ESI+) *m*/*z*: calcd. for $C_{35}H_{42}N_5O_{13}^+$ $[M+NH_4]^+$: 740.2774; found: 740.2770, (Δ = −0.54 ppm) (**S23a**); *m*/*z*: calcd. for $C_{35}H_{42}N_5O_{13}^+$ $[M+NH_4]^+$: 740.2774; found: 740.2783, (Δ = 1.22 ppm) (**S23b**)

IR (ATR)/cm^{-1} 3104, 2949, 2873, 2854, 1724, 1628, 1545, 1461, 1344, 1279, 1171, 1076, 920, 730, 721 (**S23a**); 3106, 2948, 2931, 2873, 2856, 1724, 1629, 1546, 1461, 1344, 1280, 1170, 1075, 920, 730, 721 (**S23b**)

*This entry was performed in order to separate the diastereomers formed in the previous step. The relative configuration of diastereomers **S23a** and **S23b** was assigned based on 2D NMR (see SI, pp. **S153**-**S156**).*

Downstream Reactions

Derivatization Attempts

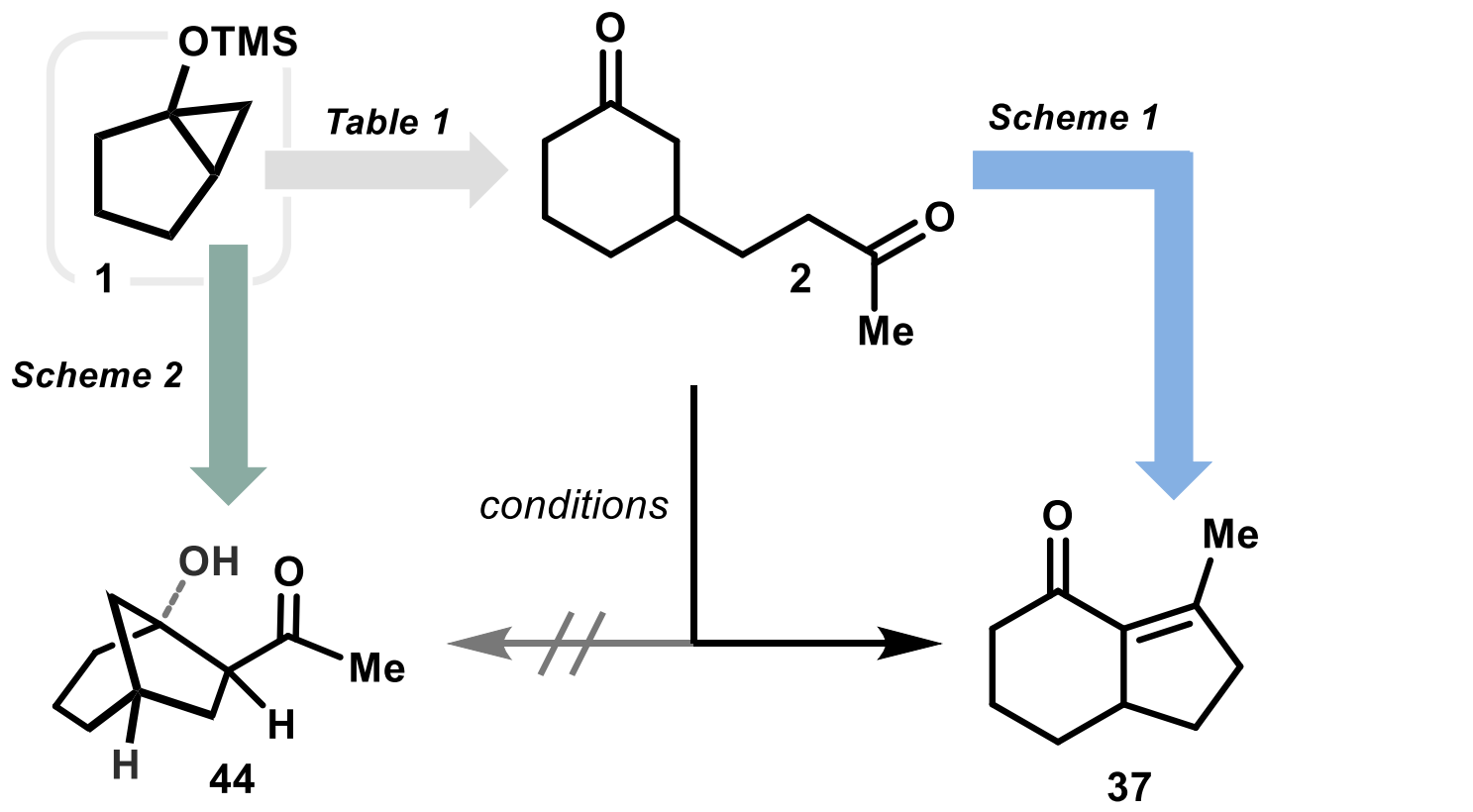

entry	conditions	outcome[a]
1	*p*TsOH, Tol, Δ	57% **37**, 0% **44**, 10% **2**
2	LDA (1.5 equiv.), $LiBF_4$ (50 mol%), THF, -78 °C - rt	9% **37**, 0% **44**, 24% **2**
3	LiHMDS (1.5 equiv), $LiBF_4$ (50 mol%), THF, -78 °C - rt	15% **37**, 0% **44**, 65% **2**
4	LDA (2.2 equiv.), THF, -78 °C - rt	Complex mixture, 0% **44**
5	Pyrrolidine (1.5 equiv.), *p*TsOH (1.5 equiv.), MeCN, Δ	Decomp., 21% **37**, 0% **44**
6	NaOMe, MeOH, Δ	quant. **37**[b]

[a] *Based on crude NMR yield using mesitylene as internal standard*
[b] *Isolated yield*

Figure S11. Attempted derivatization of **2** to form **37** or **44**.

Synthesis and Characterization of Compounds 37, 38, 40 and 41

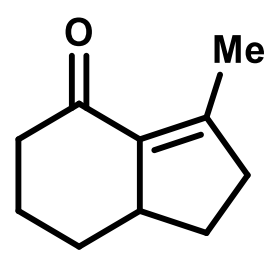

37

Condensation product 37. NaOMe (75 µL, 25 wt% in MeOH, 0.33 mmol) was added slowly to a solution of β–β coupled product **2** (17 mg, 0.10 mmol) and dry MeOH (1 mL). The resulting mixture was then heated at 70 °C for 3 h. Sat. aq. NH_4Cl (2 mL) and EtOAc (5 mL) were added to the mixture at rt. The organic layer was separated, and the aqueous layer was extracted further with EtOAc (3 x 5 mL). The combined organic layers were dried over $MgSO_4$, filtered, concentrated under reduced pressure, and purification by flash column chromatography (silica gel, *n*-Heptane/EtOAc, 9:1 to 7:3), afforded the aldol product **37** (15.0 mg, 0.100 mmol, 100%) as faint yellow oil.

R_f 0.55 (silica gel, 40% EtOAc in PE)

^{1}H NMR (400 MHz, $CDCl_3$) δ 2.95 – 2.79 (m, 1H), 2.51 – 2.37 (m, 2H), 2.33 – 2.18 (m, 2H), 2.18 – 2.09 (m, 1H), 2.07 (s, 3H), 2.06 – 1.91 (m, 2H), 1.83 – 1.66 (m, 1H), 1.54 – 1.39 (m, 1H), 1.30 – 1.21 (m, 1H).

^{13}C NMR (101 MHz, $CDCl_3$) δ 200.9, 153.2, 135.9, 47.3, 41.5, 38.7, 32.4, 31.6, 24.4, 16.5.

HRMS (ESI–) *m*/*z*: calcd. for $C_{10}H_{13}O^-$ [M-H]$^-$: 149.0972; found: 149.0973 (Δ = 0.67 ppm)

IR (ATR)/cm^{-1} 2925, 2854, 1711, 1675, 1629, 1375, 1261

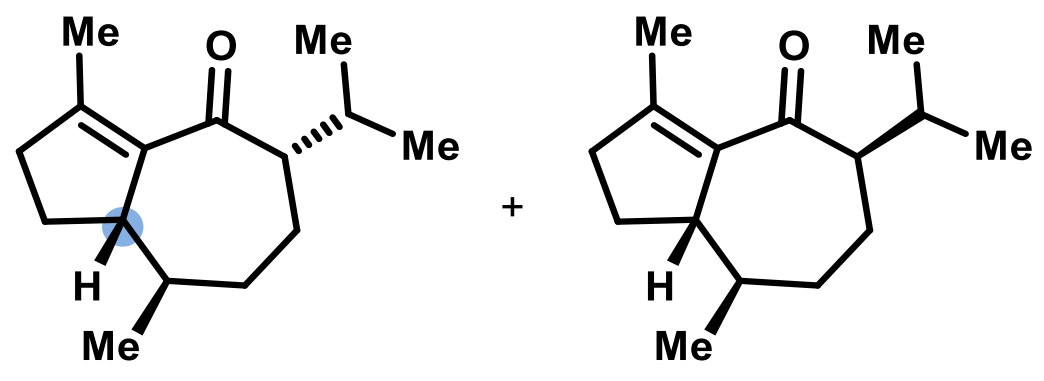

38a **38b**

Condensation product 38. NaOMe (1.12 mL, 25 wt% in MeOH, 4.89 mmol) was added slowly to a solution of β–β coupled product **27a** (338 mg, 1.42 mmol) (the major product from the previous step) and dry MeOH (15 mL). The resulting mixture was then heated to 70 °C for 5 h. Sat. aq. NH_4Cl (10 mL) and EtOAc (10 mL) were added to the mixture at rt. The organic layer was separated, and the aqueous layer was further extracted with EtOAc (3 x 10 mL). The combined organic layers were washed with brine (10 mL), dried over $MgSO_4$, filtered, concentrated under reduced pressure. Purification by flash column chromatography (silica gel, 10% to 30% EtOAc in *n*-Heptane) afforded diastereomers **38a** (123 mg, 0.560 mmol, 39%) and **38b** (112 mg, 0.507 mmol, 36%) as yellow viscous oils (combined yield 75%; dr 1.1:1).

R_f 0.40 (**38a**); 0.45 (**38b**) (silica gel, 10% EtOAc in *n*-Heptane)

^{1}H NMR (500 MHz, $CDCl_3$) δ 2.75 – 2.67 (m, 1H), 2.29 (d, *J* = 8.8 Hz, 2H), 2.14 – 2.06 (m, 2H), 2.00 (s, 3H), 1.97 – 1.87 (m, 2H), 1.67 – 1.58 (m, 2H), 1.38 – 1.27 (m, 3H), 0.92 (d, *J* = 6.6 Hz, 3H), 0.90 (d, *J* = 6.0 Hz, 3H), 0.80 (d, *J* = 6.1 Hz, 3H). (**38a**)

^{1}H NMR (400 MHz, $CDCl_3$) δ 2.74 – 2.57 (m, 1H), 2.41 – 2.23 (m, 2H), 2.23 – 2.16 (m, 1H), 2.12 – 2.06 (m, 1H), 2.01 (s, 3H), 1.95 – 1.84 (m, 2H), 1.45 (dq, *J* = 12.5, 8.8 Hz, 1H), 1.35 – 1.20 (m, 4H), 0.98 – 0.85 (m, 9H). (**38b**)

^{13}C NMR (126 MHz, $CDCl_3$) δ 207.2, 156.2, 140.4, 62.1, 52.4, 39.4, 38.1, 33.6, 30.3, 26.8, 25.9, 21.8, 21.5, 20.0, 17.2. (**38a**)

^{13}C NMR (101 MHz, $CDCl_3$) δ 205.8, 153.2, 141.3, 58.6, 52.3, 39.9, 39.6, 38.2, 29.3, 27.9, 27.5, 21.7, 21.2, 19.2, 16.9. (**38b**)

HRMS (ESI+) *m*/*z*: calcd. for $C_{15}H_{25}O^+$ $[M+H]^+$: 221.1900; found: 221.1901, (Δ = 0.45 ppm) (**38a**)

IR (ATR)/cm^{-1} 3005, 2958, 2927, 1716, 1688, 1455, 1359, 1276, 1261, 1106, 1029 (**38a**)

*The relative configuration (highlighted in blue) of **38a** was assigned based on 2D NMR (see SI, pp. **S157**).*

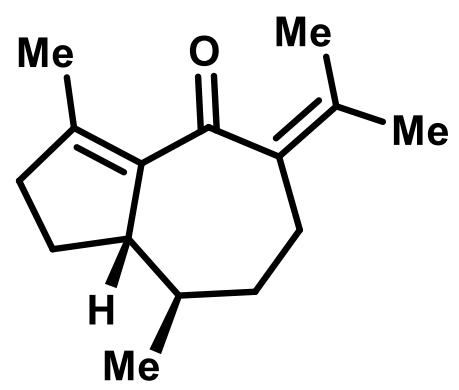

40

Condensation product 40. NaOMe (75 µL, 25 wt% in MeOH, 0.33 mmol) was added slowly to a solution of β–β coupled product **33** (24 mg, 0.10 mmol) and dry MeOH (1.1 mL). The resulting mixture was then heated to 70 °C for 3 h. Sat. aq. NH_4Cl (10 mL) and EtOAc (10 mL) were added to the mixture at rt. The organic layer was separated, and the aqueous layer was further extracted with EtOAc (3 x 10 mL). The combined organic layers were filtered through a syringe filter and dried over $MgSO_4$. The obtained filtrate was concentrated under reduced pressure, and purification by flash column chromatography (silica gel, 10% to 30% EtOAc in *n*-Heptane) afforded the aldol product **40** (19.7 mg, 0.090 mmol, 90%) as faint yellow oil.

R_f 0.70 (silica gel, 20% EtOAc in *n*-Heptane)

^{1}H NMR (500 MHz, $CDCl_3$) δ 2.57 – 2.51 (m, 1H), 2.50 – 2.42 (m, 2H), 2.33 – 2.24 (m, 1H), 2.11 (app. d, *J* = 1.1 Hz, 3H), 2.01 – 1.93 (m, 2H), 1.92 – 1.85 (m, 1H), 1.79 (s, 3H), 1.73 (s, 3H), 1.65 (ddt, *J* = 12.9, 9.0, 4.6 Hz, 1H), 1.59 – 1.51 (m, 1H), 1.34 – 1.27 (m, 1H), 0.96 (d, *J* = 6.6 Hz, 3H).

^{13}C NMR (126 MHz, $CDCl_3$) δ 199.3, 155.5, 139.7, 138.2, 135.0, 52.5, 38.5, 38.4, 36.8, 28.6, 27.4, 21.9, 21.1, 20.5, 17.1.

HRMS (ESI+) *m*/*z*: calcd. for $C_{15}H_{23}O^+$ $[M+H]^+$: 219.1743; found: 219.1744, (Δ = 0.46 ppm)

IR (ATR)/cm^{-1} 2954, 2922, 2855, 1662, 1608, 1456, 1373, 1259, 1207, 1184, 1090, 1022, 801

$([\alpha])_D^{23}$ = −25 (*c* = 0.90, $CHCl_3$)

*The relative configuration of **40** was assigned based on the stereochemical assignment of (−)-ent-melicodenone C (**41**), in the subsequent step, whose NMR data matched the reported literature.*

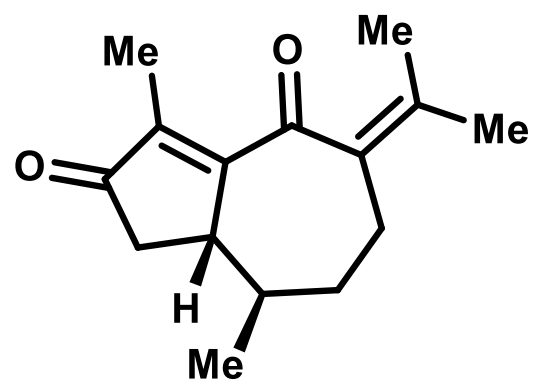

41

(−)-*ent*-melicodenone C (41). CrO_3 (464 mg, 4.64 mmol) and 3,5-dimethylpyrazole (940 mg, 9.78 mmol) were dissolved in a solution of aldol product **40** (46 mg, 0.21 mmol) and DCM (7 mL). The resulting mixture was then heated to 50 °C in a tightly closed vial. After 36 h, the reaction was diluted with sat. aq. $Na_2S_2O_3$ (10 mL) and Et_2O (10 mL). Organic layer was separated and then filtered through a pad of silica. The obtained filtrate was concentrated under reduced pressure, and purified by flash column chromatography (silica gel, 10% to 50% EtOAc in PE) affording (−)-*ent*-melicodenone C (**41**) (19.1 mg, 0.082 mmol, 39%) as a yellow solid. Recovered starting material was resubjected to the reaction conditions, affording an additional 6.0 mg of **41**, corresponding to a combined yield of 25.1 mg (0.108 mmol, 51%, brsm).

R_f 0.50 (silica gel, 25% EtOAc in PE)

^{1}H NMR (400 MHz, $CDCl_3$) δ 2.74 – 2.61 (m, 3H), 2.27 (dd, *J* = 20.6, 3.4 Hz, 1H), 2.13 – 2.06 (m, 1H), 1.99 (dd, *J* = 9.4, 4.5 Hz, 1H), 1.95 (s, 6H), 1.84 (s, 3H), 1.53 – 1.45 (m, 1H), 1.44 – 1.36 (m, 1H), 1.07 (d, *J* = 6.4 Hz, 3H).

^{13}C NMR (126 MHz, $CDCl_3$) δ 209.0, 198.6, 166.7, 144.1, 142.5, 135.8, 45.5, 42.8, 42.1, 35.5, 27.1, 22.7, 21.8, 21.7, 9.8.

HRMS (ESI+) *m*/*z*: calcd. for $C_{15}H_{24}NO_2^+$ $[M+NH_4]^+$: 250.1802; found: 250.1810, (Δ = 3.20 ppm)

IR (ATR)/cm^{-1} 2955, 2924, 2855, 1710, 1663, 1608, 1457, 1374,1285, 1191, 1140, 964

MP 77 °C

$([\alpha])_D^{23}$ = −15 (*c* = 0.75, $CHCl_3$); **$([\alpha])_D^{25}$** = +17.8 (melicodenone C, lit.[15])

The isolation report[15] does not provide solvent or concentration details for the optical rotation measurement.

Table S1. Comparison of isolated and synthetic ^{1}H and ^{13}C NMR values of melicodenone C.

Ref[15] **^{1}H NMR** (500 MHz, $CDCl_3$)	Compound **41** **^{1}H NMR** (400 MHz, $CDCl_3$)	Ref[15] **^{13}C NMR** (125 MHz, $CDCl_3$)	Compound **41** **^{13}C NMR** (126 MHz, $CDCl_3$)
2.71 (ddd, *J* = 16.2, 9.3, 1.7 Hz, 1H)<br>2.66 – 2.62 (m, 1H)<br>2.65 (dd, *J* = 20.6, 6.6 Hz, 1H)	2.74 – 2.61 (m, 3H)	208.9	209.0
2.28 (dd, *J* = 20.6, 3.1 Hz, 1H)	2.27 (dd, *J* = 20.6, 3.4 Hz, 1H)	198.5	198.6
2.11 (ddd, *J* = 16.2, 10.2, 1.4 Hz, 1H)	2.13 – 2.06 (m, 1H)	166.6	166.7
2.00 (dddd, *J* = 14.0, 9.3, 7.2, 1.4 Hz, 1H)	1.99 (dd, *J* = 9.4, 4.5 Hz, 1H)	144.0	144.1
1.96 (s, 6H)	1.95 (s, 6H)	142.4	142.5
1.84 (s, 3H)	1.84 (s, 3H)	135.7	135.8
1.54 – 1.46 (m, 1H)	1.53 – 1.45 (m, 1H)	45.4	45.5
1.41 (dddd, *J* = 14.0, 10.2, 8.6, 1.7 Hz, 1H)	1.44 – 1.36 (m, 1H)	42.7	42.8
1.08 (d, *J* = 6.6 Hz, 3H)	1.07 (d, *J* = 6.4 Hz, 3H)	42.0	42.1
		35.4	35.5
		26.9	27.1
		22.6	22.7
		21.7	21.8
		21.6	21.7
		9.6	9.8

III. X-ray Crystallography Data

Crystallographic studies were carried out on single crystals, which were coated with NVH oil, mounted at the end of a MiTeGen Dual-Thickness Micromount, and placed in the nitrogen cold stream of the diffractometer. Data were collected and processed using Oxford Diffraction Xcalibur Eos diffractometer (Mo K_α radiation), operated via CrysAlisPro software.[16] The crystal structures were solved using SHELXT (intrinsic phasing) and refined using SHELXL-2018 (least squares),[17, 18] with data processing carried out in Olex2.[19] Non-hydrogen atoms were refined anisotropically. Hydrogen atoms were placed at calculated positions and refined as riding atoms with isotropic displacement parameters (U_{iso} = 1.2 U_{eq} of the parent atom for CH_2 groups, and U_{iso} = 1.5 U_{eq} of the parent atom for CH_3 groups). For atoms demonstrating high disorder ISOR restraints were applied.

Method

The colorless crystals suitable for X-ray crystallography were obtained from a DCM solution of **57** subjected to pentane diffusion at 23 °C. Structural determination by X-ray diffraction confirmed the relative connectivity of the product. Compound **57** was obtained as a racemate.

X-ray Crystallographic Structure of 57

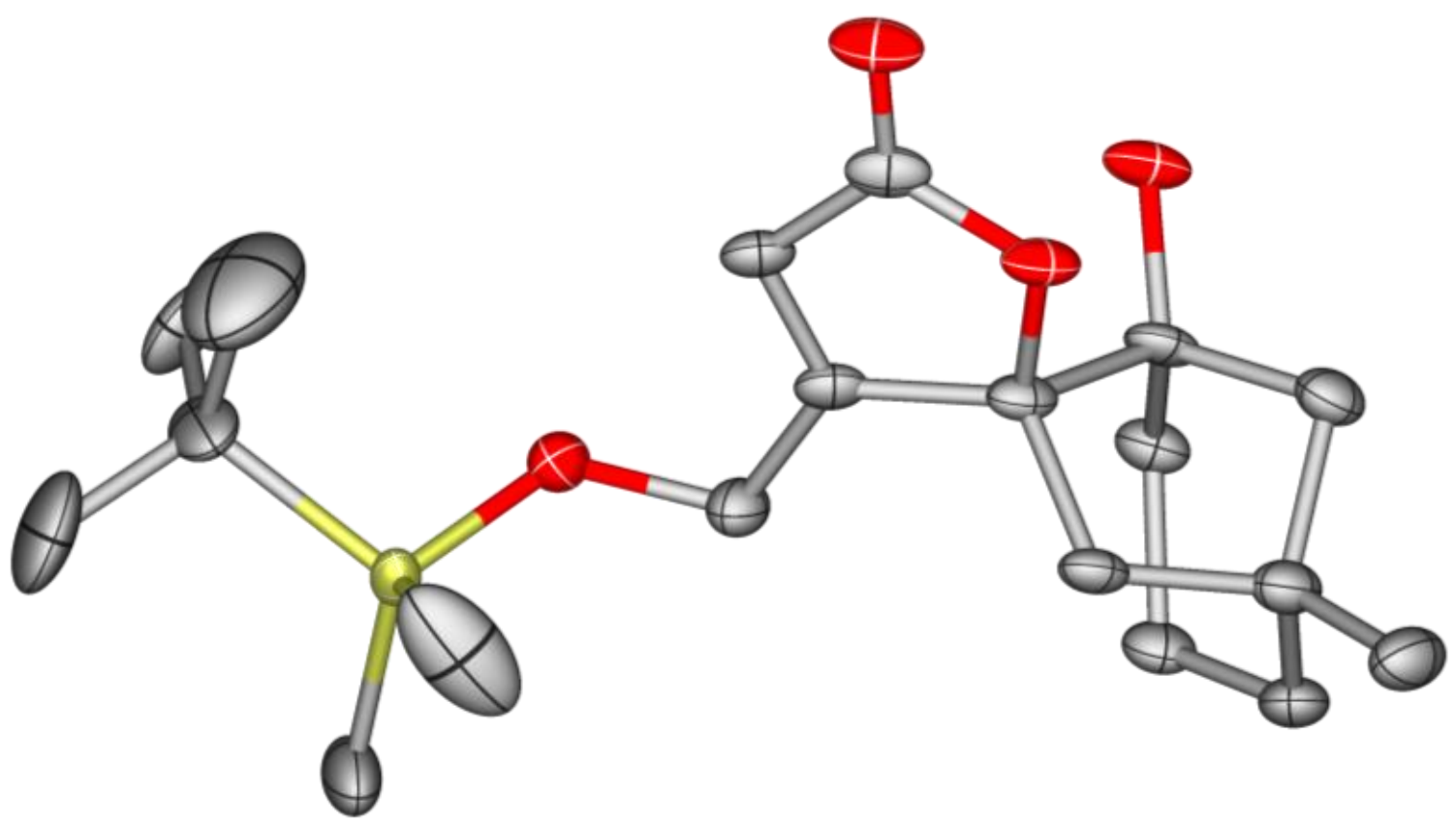

Figure S12. ORTEP plot of **57** recorded at 100(2) K. The thermal ellipsoids are set to 50% probability, the H-atoms and rotational disorder along the OTBS-group are omitted for clarity. The gray atoms are carbon, red: oxygen, and yellow: silicon.

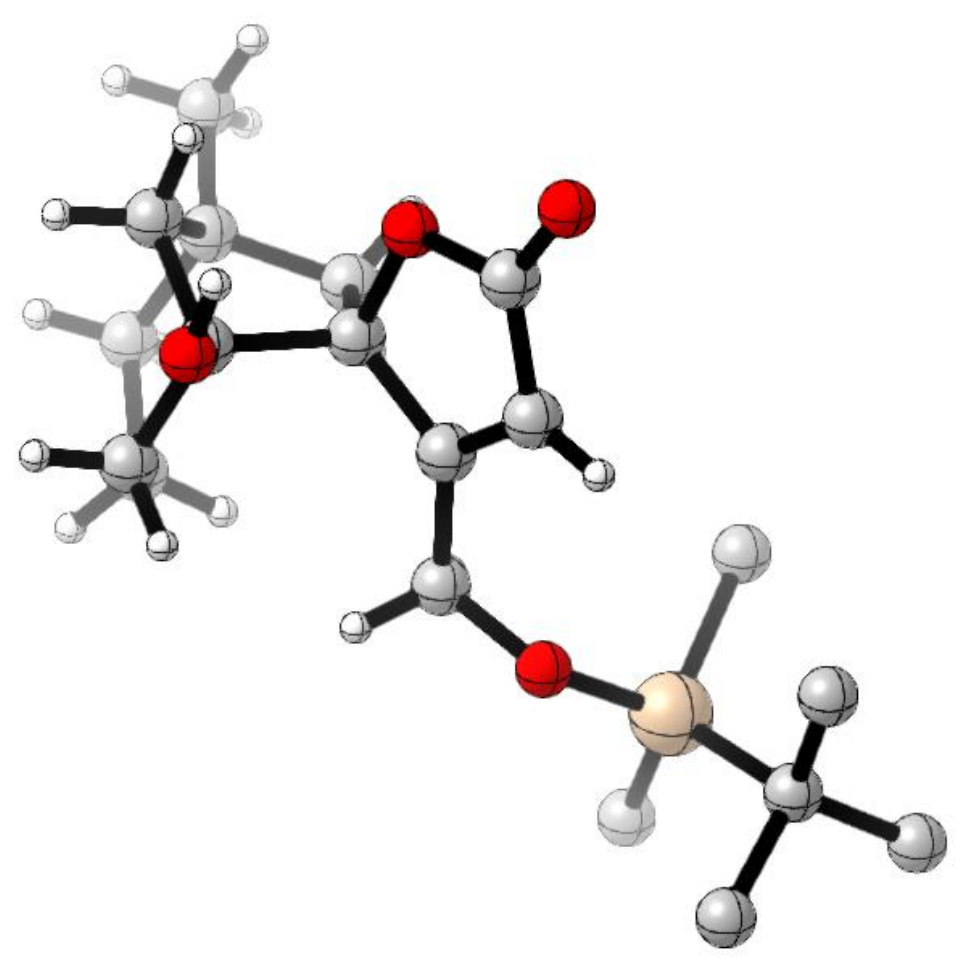

Figure S13. Molecular structure of **57** as obtained from single-crystal X-ray diffraction. Visualization generated using *CYLview20*.

X-ray Crystallographic Tables

Table S2. Crystallographic data for Compound **57.**

Compound	**57**
Empirical formula	$C_{19}H_{32}O_4Si$
Formula weight	352.53
Temperature / K	100(2)
Crystal system	monoclinic
Spacegroup	$P2_1/n$
a / Å	12.6922(2)
b / Å	11.6970(2)
c / Å	13.5008(2)
α / °	90
β / °	96.992(2)
γ / °	90
V / Å^3	1989.43(6)
Z	4
ρ_{calc} g/cm^3	1.177
μ / mm^{-1}	0.136
F(000)	768.0
Crystal size / mm^3	0.38 × 0.23 × 0.14
Radiation (Å)	Mo K_α (λ = 0.71073)
2θ range / °	6.468 to 50.092
Index ranges	$-15 \le h \le 15$, $-13 \le k \le 13$, $-16 \le l \le 16$
Reflections collected	75069
Independent reflections	3513 [R_{int} = 0.1024, R_{sigma} = 0.0346]
Data / restraints / parameters	3513/48/294
Goodness-of-fit on F^2	1.178
Final R indexes [$I \ge 2\sigma$ (I)]	R_1 = 0.0765, wR_2 = 0.1644
Final R indexes [all data]	R_1 = 0.0897, wR_2 = 0.1705
Largest diff. peak/hole / e Å^{-3}	1.00/-0.67

Table S3. Atomic coordinates (×10[4]) and equivalent isotropic displacement parameters (Å^2×10^3) for SC1-Cold(REDUCED). Ueq is defined as one third of the trace of the orthogonalised U^{ij} tensor.

Atom	*x*	*y*	*z*	U(eq)
Si01	1427(2)	6570(3)	1792.9(19)	21.8(7)
O2	4912(2)	8156.0(19)	4591.2(17)	27.1(6)
O4	2484.6(19)	7397(2)	1778.2(19)	29.1(6)
O1	5936(2)	9205(2)	3195.3(19)	31.7(6)
O3	3773(2)	9410(2)	5127.3(19)	33.5(7)
C8	5341(3)	6262(3)	4017(3)	25.0(8)
C12	3916(3)	7647(3)	3091(3)	23.6(8)
C11	3355(3)	8382(3)	3575(3)	25.2(8)
C4	5988(3)	7993(3)	3259(3)	25.9(8)
C9	4990(3)	7485(3)	3682(3)	23.4(8)
C10	3979(3)	8735(3)	4491(3)	27.3(8)
C13	3535(3)	7062(3)	2129(3)	27.7(8)
C2	6564(3)	6220(3)	4011(3)	28.5(8)
C3	6869(3)	7503(3)	4016(3)	29.1(9)
C6	6196(3)	6269(3)	2118(3)	27.9(8)
C5	6161(3)	7584(3)	2217(3)	28.0(8)
C7	6829(3)	5712(3)	3028(3)	30.8(9)
C1	7115(3)	5553(3)	4890(3)	37.3(10)
C16	267(7)	7546(8)	1621(9)	46(3)
C17	-722(17)	6824(18)	1523(18)	74(6)
C18	345(7)	8422(8)	858(7)	53(3)
C19	290(10)	8194(12)	2673(10)	101(5)
C15	1540(7)	5802(8)	2959(5)	83(3)
C14	1370(4)	5510(6)	722(5)	51(2)
C15A	1062(14)	6824(18)	3268(12)	45(5)
Si	1434(14)	6618(14)	1843(14)	97(6)
C14A	1660(13)	5094(13)	1742(13)	38(5)
C16A	316(12)	7360(15)	1095(13)	52(4)

Atom	*x*	*y*	*z*	U(eq)
C17A	-830(20)	7000(20)	1320(30)	62(8)
C18A	280(15)	7510(30)	145(14)	138(9)
C19A	409(13)	8720(13)	1818(16)	86(6)

Table S4. Bond Lengths for SC1-Cold(REDUCED).

Atom	Atom	Length/Å	Atom	Atom	Length/Å
Si01	O4	1.657(4)	C4	C3	1.532(5)
Si01	C16	1.855(9)	C4	C5	1.526(5)
Si01	C15	1.803(7)	C2	C3	1.550(5)
Si01	C14	1.900(7)	C2	C7	1.528(5)
O2	C9	1.471(4)	C2	C1	1.518(5)
O2	C10	1.357(5)	C6	C5	1.546(5)
O4	C13	1.415(4)	C6	C7	1.530(5)
O4	Si	1.626(18)	C16	C17	1.51(2)
O1	C4	1.421(4)	C16	C18	1.464(14)
O3	C10	1.218(4)	C16	C19	1.607(14)
C8	C9	1.549(5)	C15A	Si	2.05(2)
C8	C2	1.554(5)	Si	C14A	1.81(2)
C12	C11	1.336(5)	Si	C16A	1.85(2)
C12	C9	1.505(5)	C16A	C17A	1.59(3)
C12	C13	1.496(5)	C16A	C18A	1.29(2)
C11	C10	1.445(5)	C16A	C19A	1.86(3)
C4	C9	1.569(5)			

Table S5. Anisotropic displacement parameters (Å^2×10^3) for SC1-Cold(REDUCED). The anisotropic displacement factor exponent takes the form: $-2\pi^2[h^2a^{*2}U^{11}+2\ hka^*b^*U^{12}+...]$.

Atom	U11	U22	U33	U23	U13	U12
Si01	17.3(12)	17.9(16)	29.3(12)	6.4(10)	-1.2(9)	-0.9(11)
O2	43.5(16)	15.7(12)	21.1(13)	-3.6(10)	0.2(11)	0.1(11)
O4	27.2(14)	23.1(13)	35.5(15)	-1.2(11)	-3.0(11)	1.9(11)
O1	51.7(17)	14.5(12)	28.2(14)	-1.5(10)	2.7(13)	-5.9(11)
O3	54.4(18)	20.9(13)	27.5(14)	-6.4(11)	14.3(12)	-3.9(12)
C8	35(2)	12.7(16)	26.4(19)	-0.7(14)	-0.6(15)	-1.6(14)
C12	34(2)	12.2(16)	24.8(19)	2.2(14)	4.9(15)	0.4(14)
C11	36(2)	13.1(16)	27.3(19)	1.0(14)	5.6(15)	1.7(15)
C4	38(2)	11.0(16)	27.7(19)	0.5(14)	0.5(16)	-5.7(15)
C9	35(2)	15.0(17)	19.4(18)	-3.2(13)	-0.5(14)	0.5(15)
C10	46(2)	15.2(17)	22.1(18)	3.2(14)	10.3(16)	-2.6(16)
C13	28(2)	23.9(19)	29(2)	-4.6(15)	-2.7(15)	3.4(15)
C2	34(2)	19.0(18)	31(2)	-2.5(15)	-1.8(16)	-0.5(15)
C3	34(2)	21.8(19)	30(2)	-3.7(15)	-3.3(16)	-4.5(16)
C6	35(2)	20.2(18)	28(2)	-6.9(15)	3.2(16)	-2.2(15)
C5	36(2)	19.9(18)	28(2)	-3.1(15)	3.8(16)	-5.0(16)
C7	33(2)	17.1(18)	41(2)	-4.4(16)	0.2(17)	0.9(15)
C1	43(2)	26(2)	39(2)	-2.2(17)	-8.4(18)	7.2(18)
C16	28(4)	40(4)	70(7)	-30(5)	9(5)	8(3)
C17	28(6)	94(13)	100(12)	4(9)	6(8)	26(7)
C18	47(4)	51(5)	61(6)	24(4)	5(4)	29(4)
C19	99(7)	106(8)	97(8)	-34(6)	9(6)	42(6)
C15	105(7)	94(7)	46(4)	27(4)	-11(4)	-55(6)
C14	22(3)	52(4)	77(5)	-30(3)	-3(3)	3(3)
C15A	37(10)	62(13)	36(10)	-2(9)	8(7)	-16(9)
Si	101(12)	33(8)	155(14)	-9(8)	9(9)	5(7)
C14A	41(10)	29(9)	44(10)	-5(7)	5(8)	-12(7)
C16A	39(6)	59(8)	55(7)	13(6)	-4(6)	-19(6)

Atom	U11	U22	U33	U23	U13	U12
C17A	28(11)	38(8)	112(18)	46(10)	-24(9)	-12(8)
C18A	79(11)	256(19)	71(11)	79(12)	-27(8)	-85(12)
C19A	69(9)	56(9)	133(16)	14(9)	11(9)	16(7)

Table S6. Hydrogen atom coordinates (Å×10^4) and isotropic displacement parameters (Å^2×10^3) for SC1-Cold(REDUCED).

**Atom**	***x***	***y***	***z***	**U(eq)**
H1	5978.46	9482.95	3772.33	47
H8A	4988.53	5686.83	3550.86	30
H8B	5154.79	6103.83	4694.61	30
H11	2655.07	8633.82	3351.63	30
H13A	4009.63	7256.76	1623.92	33
H13B	3559.7	6223.16	2228.63	33
H3A	6858.08	7847.2	4684.64	35
H3B	7578.42	7618.4	3797.49	35
H6A	5462.5	5965.22	2038.63	33
H6B	6522.93	6065.69	1512.1	33
H5A	6837.24	7905.64	2045.29	34
H5B	5581.77	7884.95	1729.68	34
H7A	6678.16	4881.36	3020.85	37
H7B	7597.12	5811.83	2985.3	37
H1A	6971.57	5916.16	5513.96	56
H1B	7881.83	5546.03	4856.03	56
H1C	6847.17	4766.54	4867.62	56
H17A	-1348.2	7320.96	1458.6	111
H17B	-721.6	6342.61	2117.45	111
H17C	-740.23	6337.82	930.09	111
H18A	253.42	8065.33	196.24	80
H18B	1042.79	8788.89	971.82	80
H18C	-210.73	8996.56	895.82	80
H19A	948.81	8634.81	2805.8	151
H19B	254.31	7631.14	3204.82	151
H19C	-319.4	8712.53	2648.55	151

**Atom**	***x***	***y***	***z***	**U(eq)**
H15A	2076.31	5199.44	2953.14	125
H15B	853.96	5456.69	3046.6	125
H15C	1751.66	6330.73	3509.82	125
H14A	1246.35	5921.45	85.84	77
H14B	790.76	4966.47	768.39	77
H14C	2044.68	5094.9	758.01	77
H15D	1644.72	6533.98	3743.99	67
H15E	411.16	6399.79	3344.68	67
H15F	951.86	7637.76	3395.33	67
H14D	1902.65	4926.89	1096.09	57
H14E	997.19	4681.95	1796.33	57
H14F	2201.94	4850.96	2280.45	57
H17D	-1362.69	7511.18	972.67	93
H17E	-869.83	7044.88	2044.14	93
H17F	-979.24	6210.22	1097.96	93
H18D	533.43	6818.27	-161.29	208
H18E	732.52	8158.01	14.01	208
H18F	-452.87	7666.61	-140.55	208
H19D	719.65	9315.39	1433.87	129
H19E	857.84	8603.44	2453.67	129
H19F	-302.7	8955.52	1947.6	129

Table S7. Torsion Angles for SC1-Cold(REDUCED).

A	B	C	D	Angle/˚	A	B	C	D	Angle/˚
Si01	O4	C13	C12	-107.5(3)	C13	C12	C9	O2	-174.7(3)
O4	Si01	C16	C17	174.3(11)	C13	C12	C9	C8	-56.4(5)
O4	Si01	C16	C18	42.7(8)	C13	C12	C9	C4	71.3(4)
O4	Si01	C16	C19	-73.8(8)	C2	C8	C9	O2	-96.7(3)
O4	Si	C16A	C17A	-160.9(18)	C2	C8	C9	C12	147.2(3)
O4	Si	C16A	C18A	64(2)	C2	C8	C9	C4	12.9(3)
O4	Si	C16A	C19A	-59.4(13)	C3	C4	C9	O2	72.9(3)
O1	C4	C9	O2	-50.2(3)	C3	C4	C9	C8	-39.1(3)
O1	C4	C9	C8	-162.2(3)	C3	C4	C9	C12	-173.5(3)
O1	C4	C9	C12	63.4(4)	C3	C4	C5	C6	57.1(4)
O1	C4	C3	C2	170.6(3)	C3	C2	C7	C6	-61.8(4)
O1	C4	C5	C6	-177.7(3)	C5	C4	C9	O2	-170.7(3)
C8	C2	C3	C4	-42.9(3)	C5	C4	C9	C8	77.4(3)
C8	C2	C7	C6	49.2(4)	C5	C4	C9	C12	-57.0(4)
C12	C11	C10	O2	-3.4(4)	C5	C4	C3	C2	-70.6(3)
C12	C11	C10	O3	178.1(4)	C5	C6	C7	C2	44.1(4)
C11	C12	C9	O2	4.4(4)	C7	C2	C3	C4	73.0(4)
C11	C12	C9	C8	122.6(3)	C7	C6	C5	C4	-41.7(4)
C11	C12	C9	C4	-109.7(3)	C1	C2	C3	C4	-164.4(3)
C11	C12	C13	O4	0.5(5)	C1	C2	C7	C6	173.6(3)
C9	O2	C10	O3	-175.0(3)	C16	Si01	O4	C13	163.3(5)
C9	O2	C10	C11	6.2(4)	C15	Si01	O4	C13	41.4(5)
C9	C8	C2	C3	18.0(4)	C15	Si01	C16	C17	-66.6(12)
C9	C8	C2	C7	-96.1(3)	C15	Si01	C16	C18	161.8(7)
C9	C8	C2	C1	140.5(3)	C15	Si01	C16	C19	45.3(9)

A	B	C	D	Angle/˚	A	B	C	D	Angle/˚
C9	C12	C11	C10	-0.9(4)	C14	Si01	O4	C13	-77.8(4)
C9	C12	C13	O4	179.4(3)	C14	Si01	C16	C17	56.4(12)
C9	C4	C3	C2	50.7(3)	C14	Si01	C16	C18	-75.2(8)
C9	C4	C5	C6	-54.1(4)	C14	Si01	C16	C19	168.3(7)
C10	O2	C9	C8	-132.5(3)	C15A	Si	C16A	C17A	-50(2)
C10	O2	C9	C12	-6.5(3)	C15A	Si	C16A	C18A	174.6(17)
C10	O2	C9	C4	117.9(3)	C15A	Si	C16A	C19A	51.1(12)
C13	O4	Si	C15A	81.3(11)	Si	O4	C13	C12	-104.1(9)
C13	O4	Si	C14A	-32.4(15)	C14A	Si	C16A	C17A	65(2)
C13	O4	Si	C16A	-170.7(8)	C14A	Si	C16A	C18A	-70(2)
C13	C12	C11	C10	178.1(3)	C14A	Si	C16A	C19A	166.5(13)

IV. Supplementary References

[1] I. Ryu, T. Aya, S. Otani, S. Murai, N. Sonoda, *J. Organomet. Chem.* **1987**, *321*, 279–290.

[2] K. C. Nicolaou, R. Yu, Z. Lu, F. G. Alvarez, *J. Am. Chem. Soc.* **2022**, *144*, 5190–5196.

[3] D. Rosa, A. Orellana, *Org. Lett.* **2011**, *13*, 110–113.

[4] E. M. Landwehr, M. A. Baker, T. Oguma, H. E. Burdge, T. Kawajiri, R. A. Shenvi, *Science* **2022**, *375*, 1270–1274.

[5] V. Patel, A. J. Ragauskas, J. B. Stothers, *Can. J. Chem.* **1986**, *64*, 1440–1449.

[6] H. M. Cheng, W. Tian, P. A. Peixoto, B. Dhudshia, D. Y. K. Chen, *Angew. Chem. Int. Ed.* **2011**, *50*, 4165–4168.

[7] R. F. Cunico, *Organometallics* **1985**, *4*, 2176–2179.

[8] E. Lattmann, H. M. R. Hoffmann, *Synthesis* **1996**, 155–163.

[9] S. G. Krasutsky, S. H. Jacobo, S. R. Tweedie, R. Krishnamoorthy, A. S. Filatov, *Org. Process Res. Dev.* **2015**, *19*, 284–289.

[10] K. Miyamoto, Y. Sei, K. Yamaguchi, M. Ochiai, *J. Am. Chem. Soc.* **2009**, *131*, 1382–1383.

[11] S. Husinec, V. Savić, *J. Serb. Chem. Soc.* **2000**, *65*, 87–93.

[12] R. Hyakutake, N. Gondo, Y. Ueda, T. Yoshimura, T. Furuta, T. Kawabata, *Tetrahedron Lett.* **2016**, *57*, 1321–1324.

[13] K. P. Haval, N. P. Argade, *Synthesis* **2007**, 2198–2202.

[14] T. Suzuki, W. Ikeda, A. Kanno, K. Ikeuchi, K. Tanino, *Chem. Eur. J.* **2023**, *29*, e202203511.

[15] K. Nakashima, M. Oyama, T. Ito, J. R. Witono, D. Darnaedi, T. Tanaka, J. Murata, M. Iinuma, *Chem. Biodivers.* **2012**, *9*, 2195–2202.

[16] *CrysAlisPro*, Agilent Technologies Ltd., Yarnton, UK, **2014**.

[17] G. M. Sheldrick, *Acta Crystallogr., Sect. A* **2015**, *71*, 3–8.

[18] G. M. Sheldrick, *Acta Crystallogr., Sect. C* **2015**, *71*, 3–8.

[19] O. V. Dolomanov, L. J. Bourhis, R. J. Gildea, J. A. K. Howard, H. Puschmann, *J. Appl. Crystallogr.* **2009**, *42*, 339–341.

V. Copies of ^{1}H, ^{13}C and 2D NMR of Compounds S5-S7, S9-S13, S15-S16, S21-S23, 1-3, 5-41, 43-55, 57-58, 60-65, and 68-69

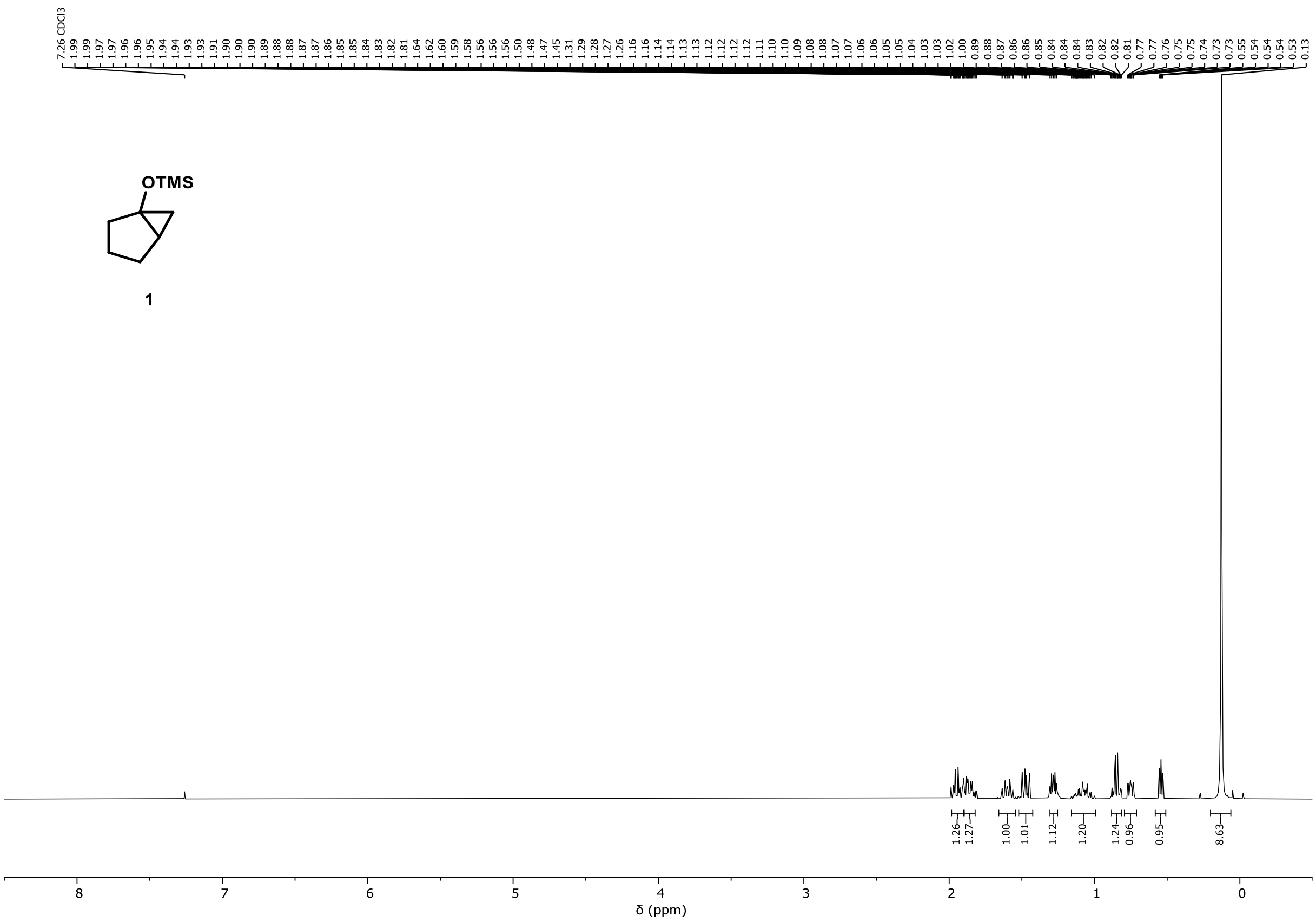
7.26 CDCl3
1.99 1.99 1.97 1.97 1.96 1.95 1.94 1.94 1.93 1.93 1.91 1.90 1.90 1.89 1.88 1.88 1.87 1.87 1.86 1.85 1.85 1.84 1.83 1.82 1.81 1.64 1.62 1.60 1.59 1.58 1.56 1.56 1.56 1.50 1.48 1.47 1.45 1.31 1.29 1.28 1.27 1.26 1.16 1.16 1.14 1.14 1.13 1.13 1.12 1.12 1.12 1.12 1.11 1.10 1.10 1.09 1.08 1.08 1.07 1.07 1.06 1.06 1.05 1.05 1.04 1.03 1.03 1.02 1.00 0.89 0.88 0.87 0.86 0.86 0.85 0.84 0.84 0.84 0.83 0.82 0.82 0.81 0.77 0.77 0.76 0.75 0.75 0.74 0.73 0.73 0.55 0.54 0.54 0.54 0.53 0.13
OTMS
1
1.26 1.27 1.00 1.01 1.12 1.20 1.24 0.96 0.95 8.63
δ (ppm)

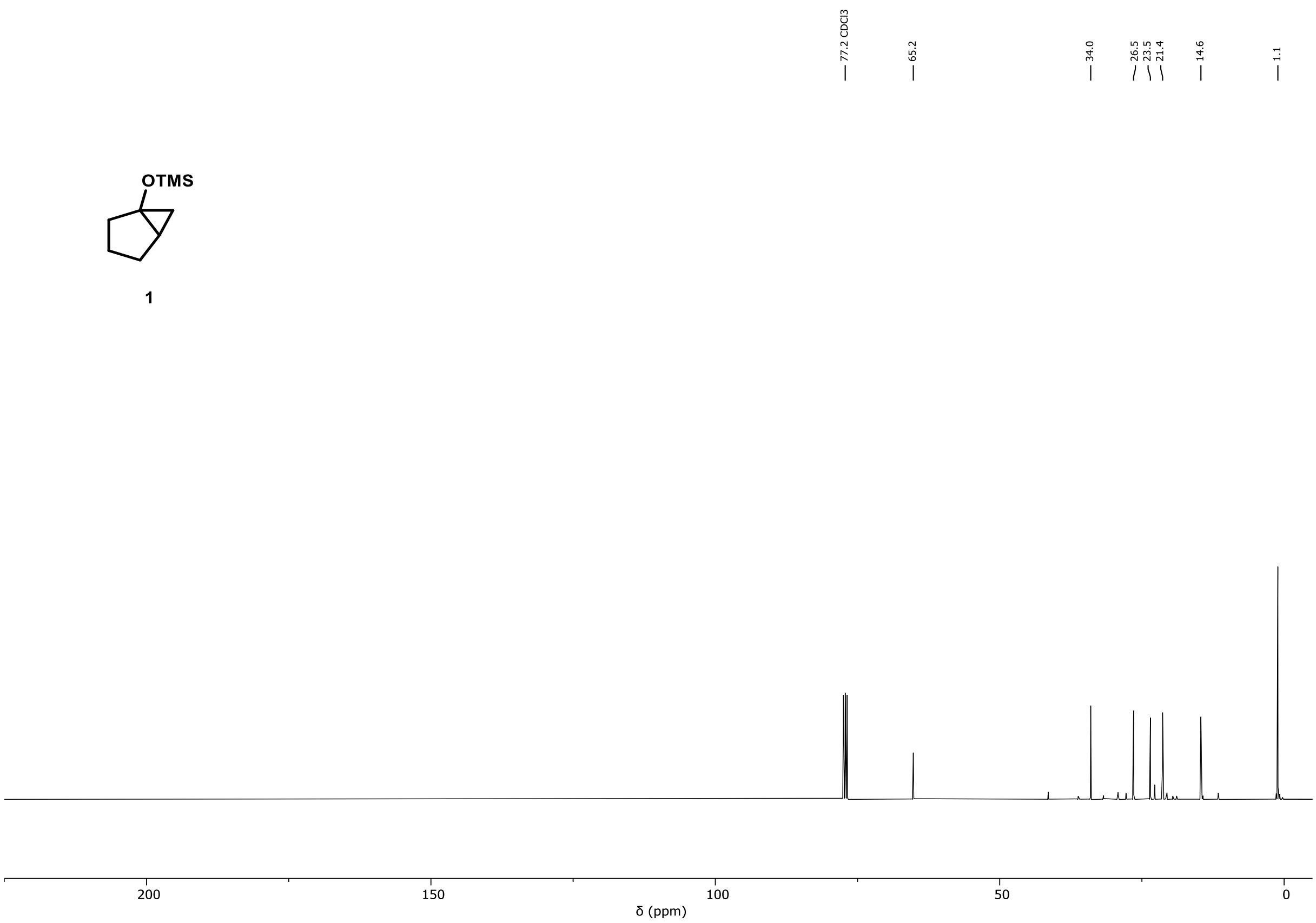
77.2 CDCl3
65.2
34.0
26.5
23.5
21.4
14.6
1.1
OTMS
1
δ (ppm)

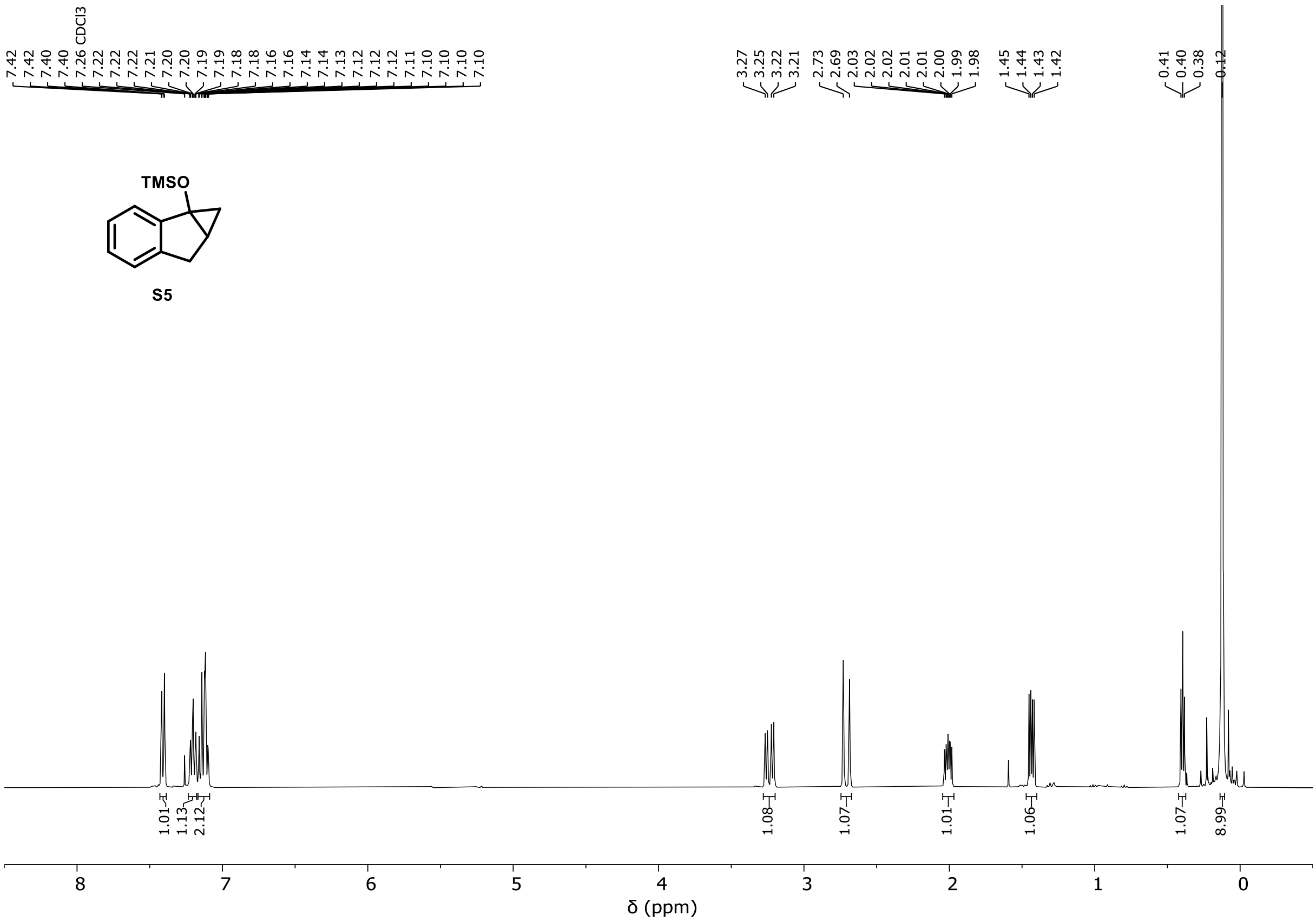
7.42
7.42
7.40
7.40
7.26 CDCl3
7.22
7.22
7.22
7.21
7.20
7.20
7.19
7.19
7.18
7.18
7.16
7.16
7.14
7.14
7.13
7.12
7.12
7.12
7.11
7.10
7.10
7.10
7.10
3.27
3.25
3.22
3.21
2.73
2.69
2.03
2.02
2.02
2.01
2.00
1.99
1.98
1.45
1.44
1.43
1.42
0.41
0.40
0.38
0.12
TMSO
S5
1.01
1.13
2.12
1.08
1.07
1.01
1.06
1.07
8.99
8
7
6
5
4
3
2
1
0
δ (ppm)

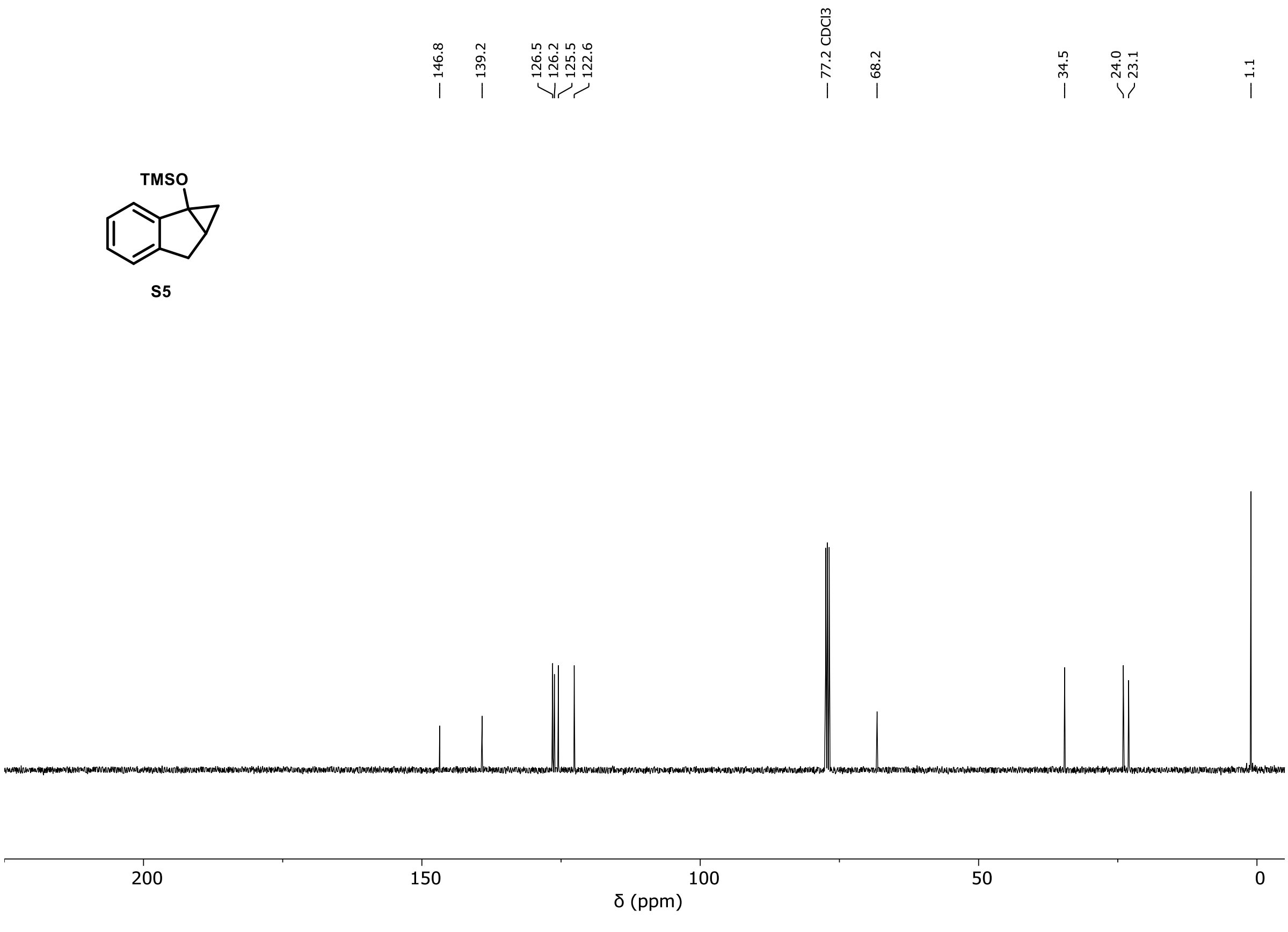
146.8
139.2
126.5
126.2
125.5
122.6
77.2 CDCl3
68.2
34.5
24.0
23.1
1.1
TMSO
S5
200
150
100
50
0
δ (ppm)

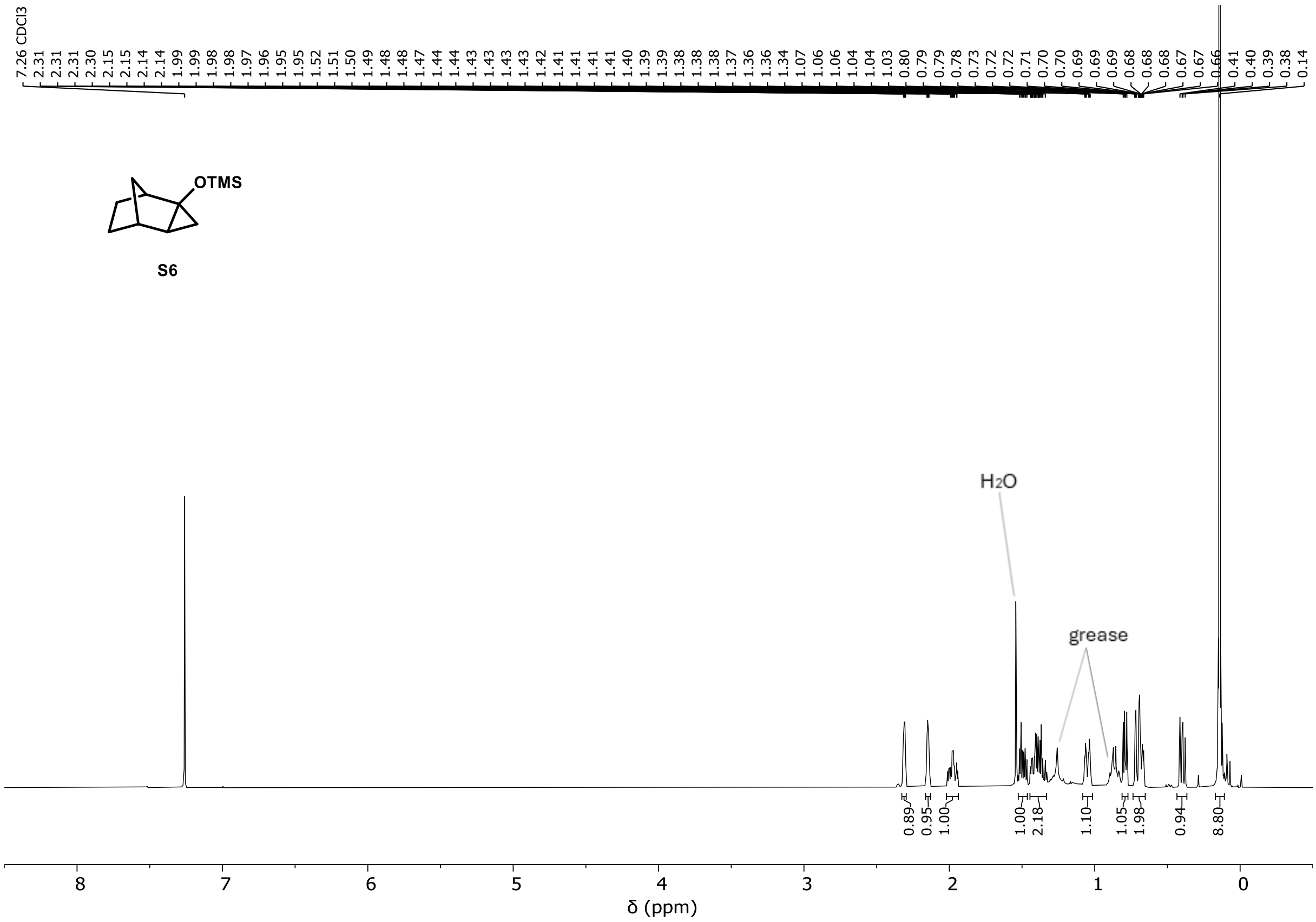
OTMS
S6
H2O
grease
δ (ppm)

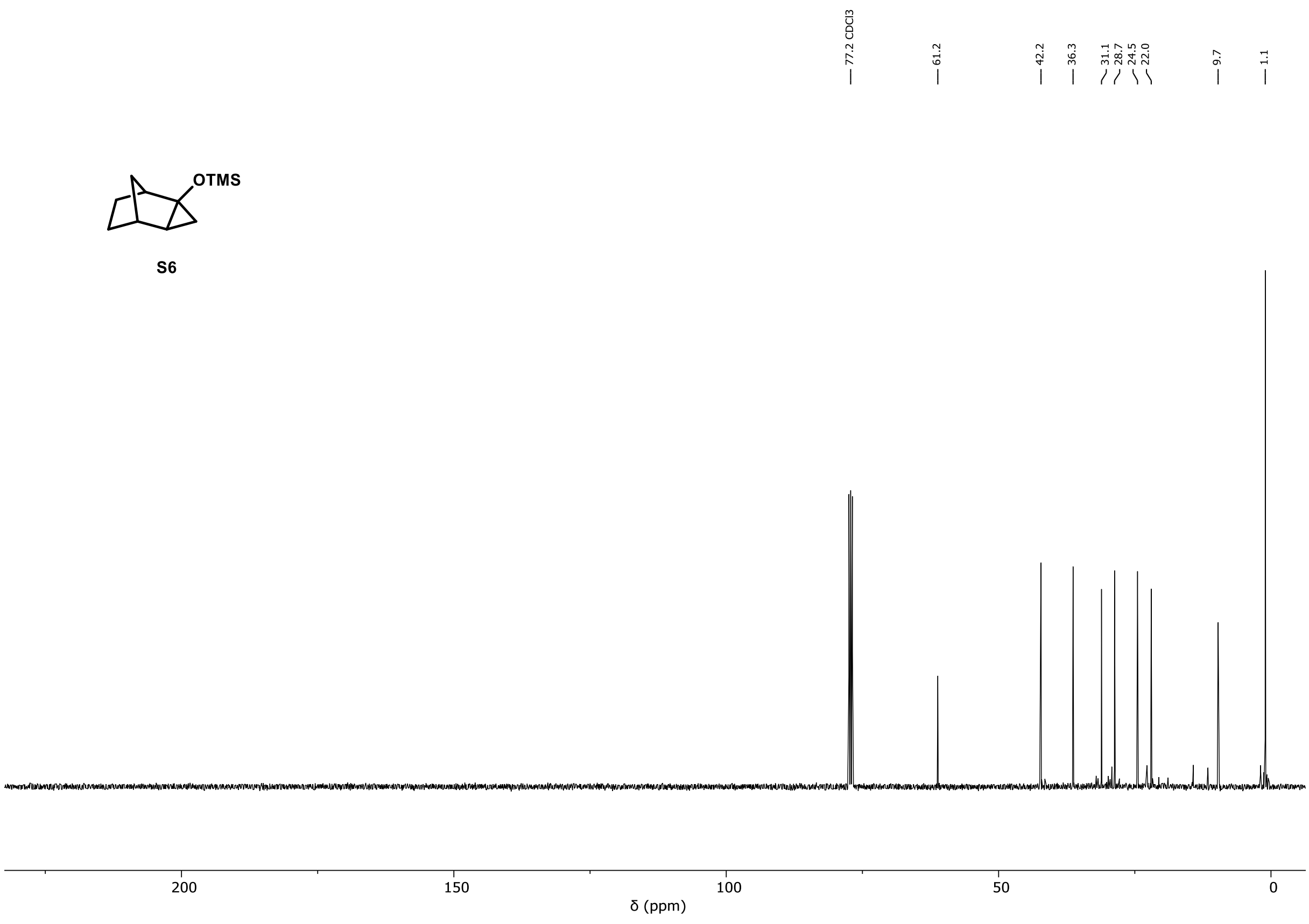
77.2 CDCl3
61.2
42.2
36.3
31.1
28.7
24.5
22.0
9.7
1.1
OTMS
S6
δ (ppm)

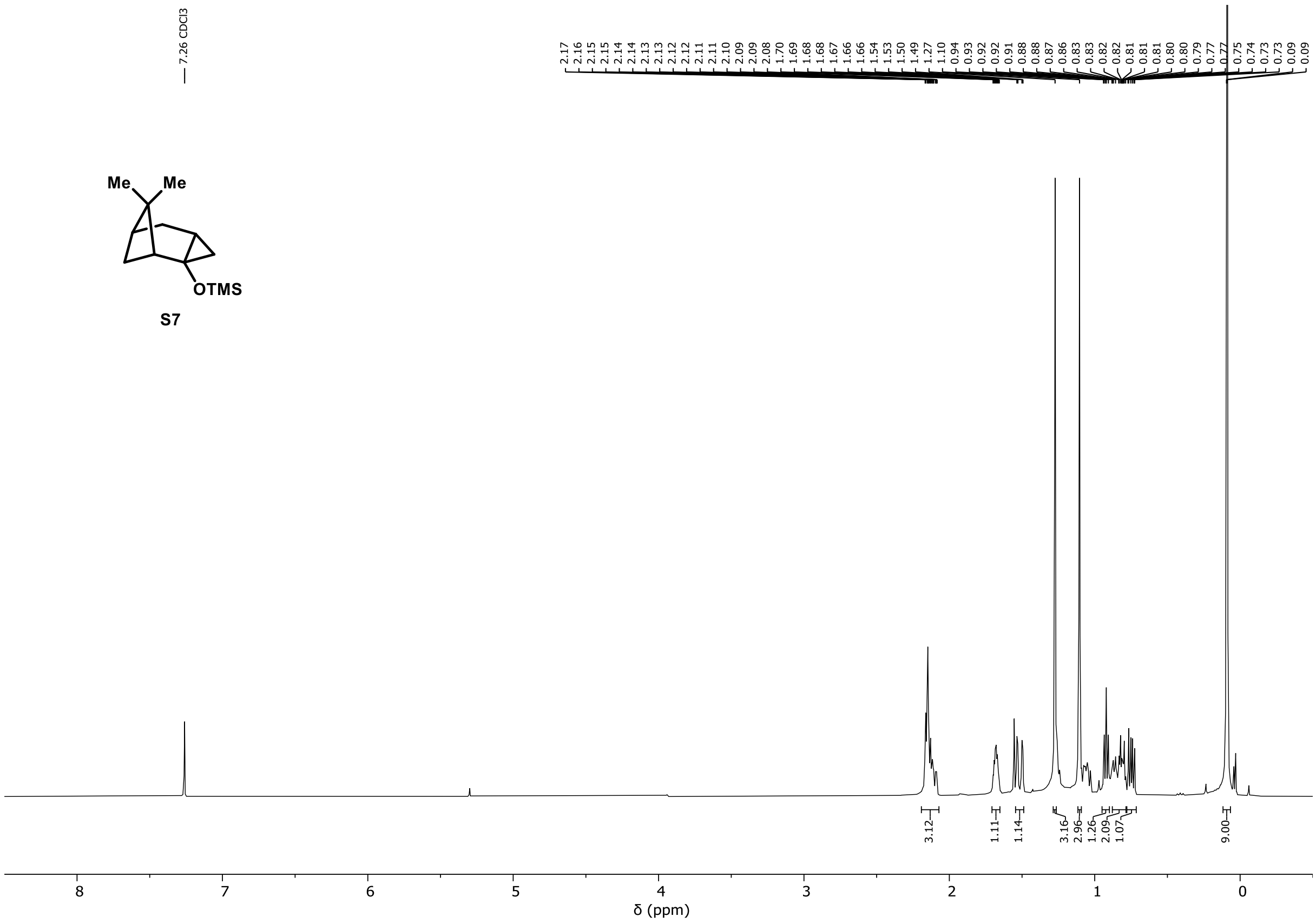
7.26 CDCl3
2.17 2.16 2.15 2.14 2.14 2.13 2.13 2.12 2.11 2.11 2.10 2.09 2.09 2.08 1.70 1.69 1.68 1.68 1.67 1.66 1.66 1.54 1.53 1.50 1.49 1.27 1.10 0.94 0.93 0.92 0.92 0.91 0.88 0.88 0.87 0.86 0.83 0.83 0.82 0.82 0.81 0.81 0.81 0.80 0.80 0.79 0.77 0.77 0.75 0.74 0.73 0.73 0.09 0.09
Me
Me
OTMS
S7
3.12
1.11
1.14
3.16
2.96
1.26
2.09
1.07
9.00
δ (ppm)

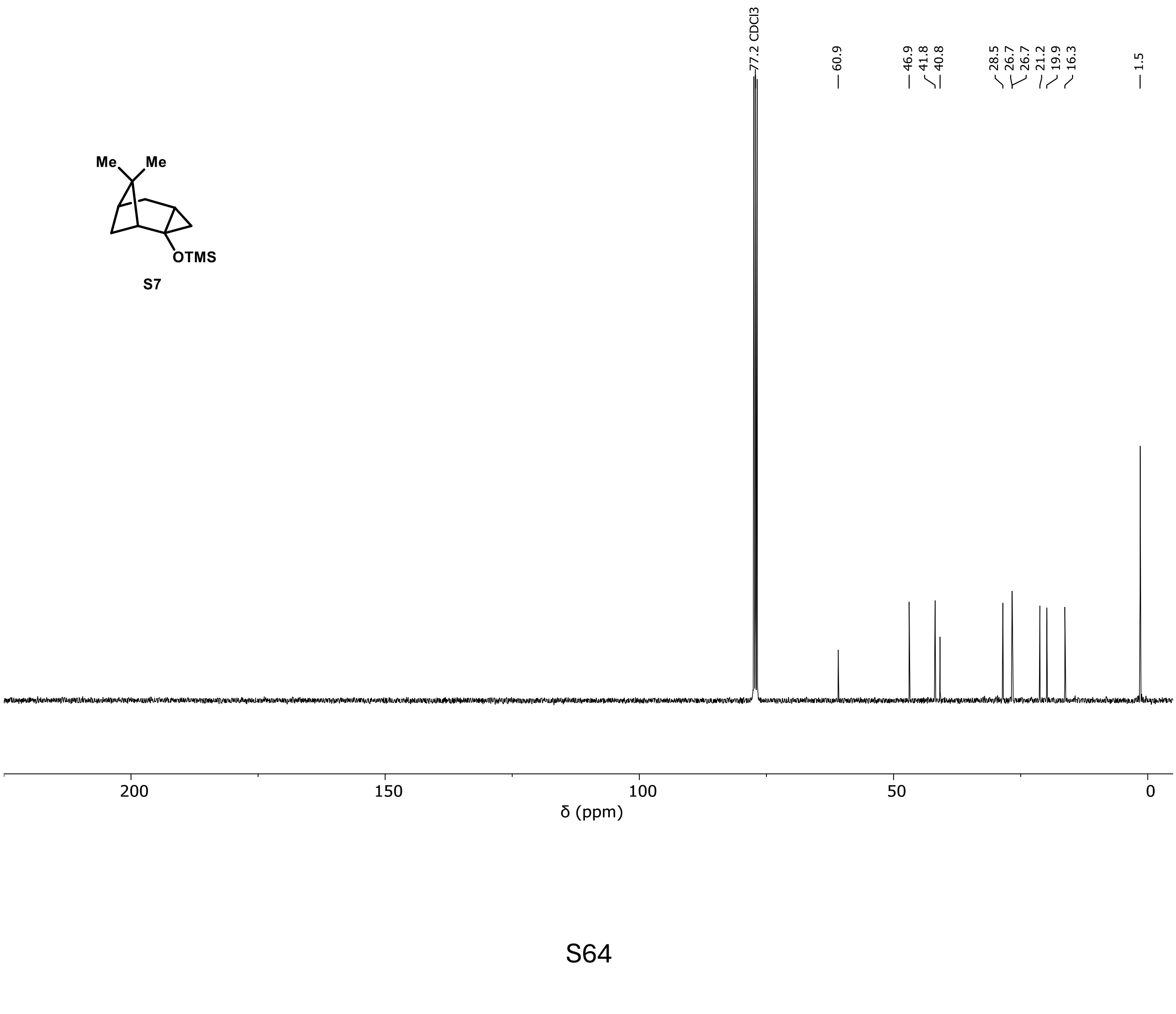
77.2 CDCl3
60.9
46.9
41.8
40.8
28.5
26.7
26.7
21.2
19.9
16.3
1.5
Me
Me
OTMS
S7
δ (ppm)

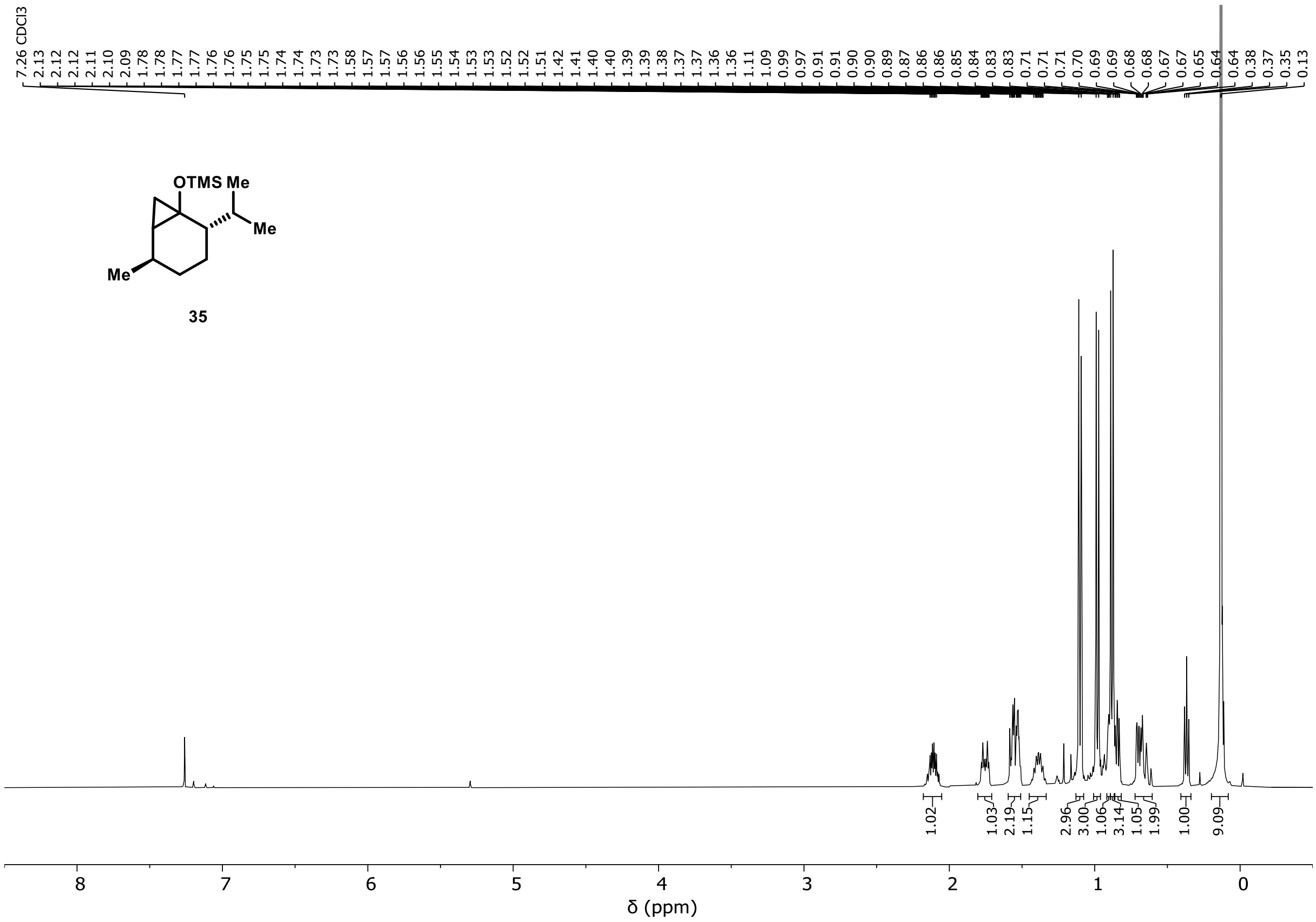
7.26 CDCl3
2.13
2.12
2.12
2.11
2.10
2.09
1.78
1.78
1.77
1.77
1.76
1.76
1.75
1.75
1.74
1.74
1.73
1.73
1.58
1.57
1.57
1.56
1.56
1.55
1.54
1.53
1.53
1.52
1.52
1.51
1.42
1.41
1.40
1.39
1.39
1.38
1.37
1.37
1.36
1.36
1.11
1.09
0.99
0.97
0.91
0.91
0.90
0.90
0.89
0.89
0.87
0.86
0.86
0.85
0.84
0.83
0.83
0.71
0.71
0.71
0.70
0.69
0.69
0.68
0.68
0.67
0.67
0.65
0.64
0.64
0.38
0.37
0.35
0.13
OTMS
Me
Me
Me
35
1.02
1.03
2.19
1.15
2.96
3.00
1.06
3.14
1.05
1.99
1.00
9.09
8
7
6
5
4
3
2
1
0
δ (ppm)

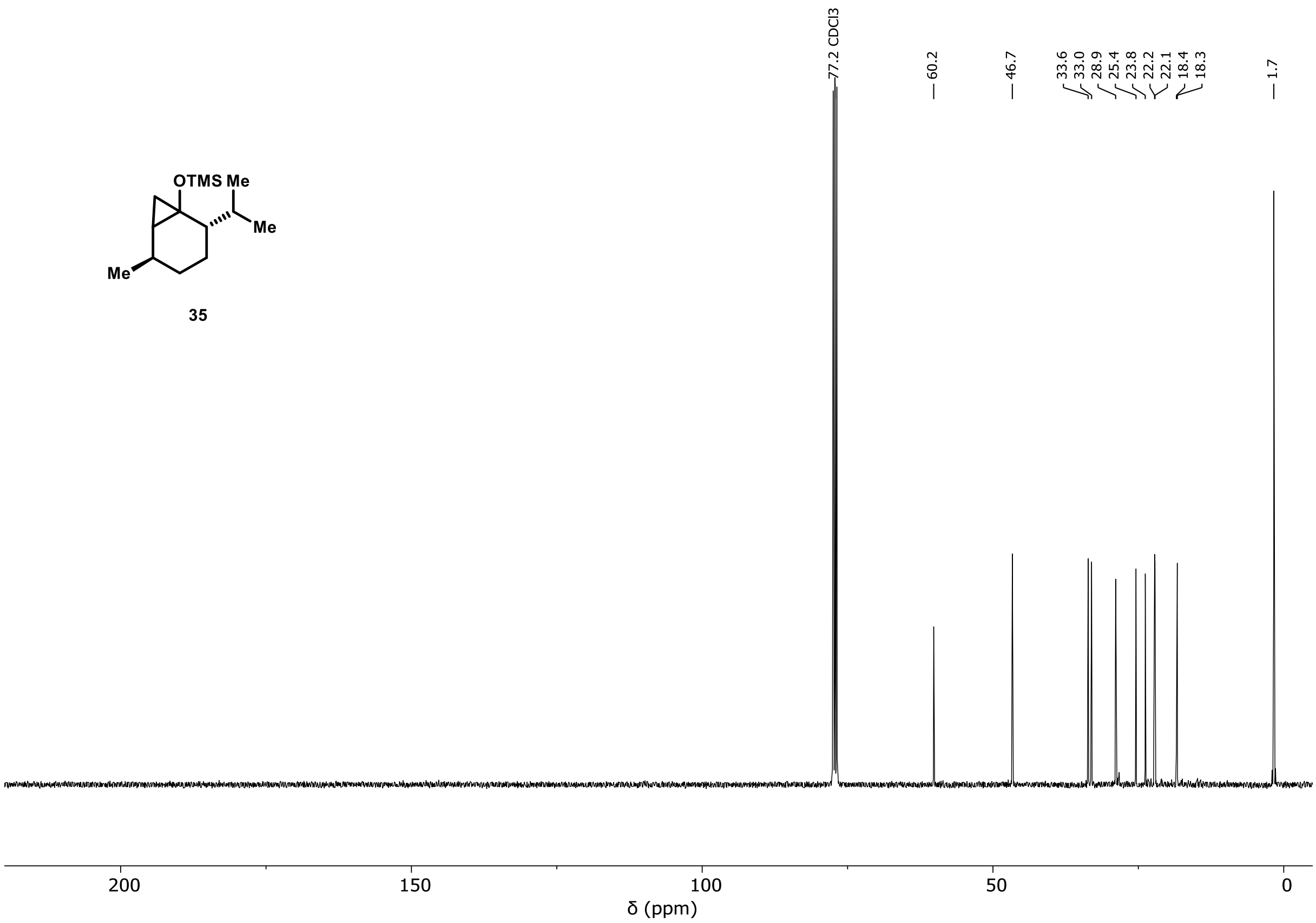
77.2 CDCl3
60.2
46.7
33.6
33.0
28.9
25.4
23.8
22.2
22.1
18.4
18.3
1.7
OTMS
Me
Me
Me
35
200
150
100
50
0
δ (ppm)

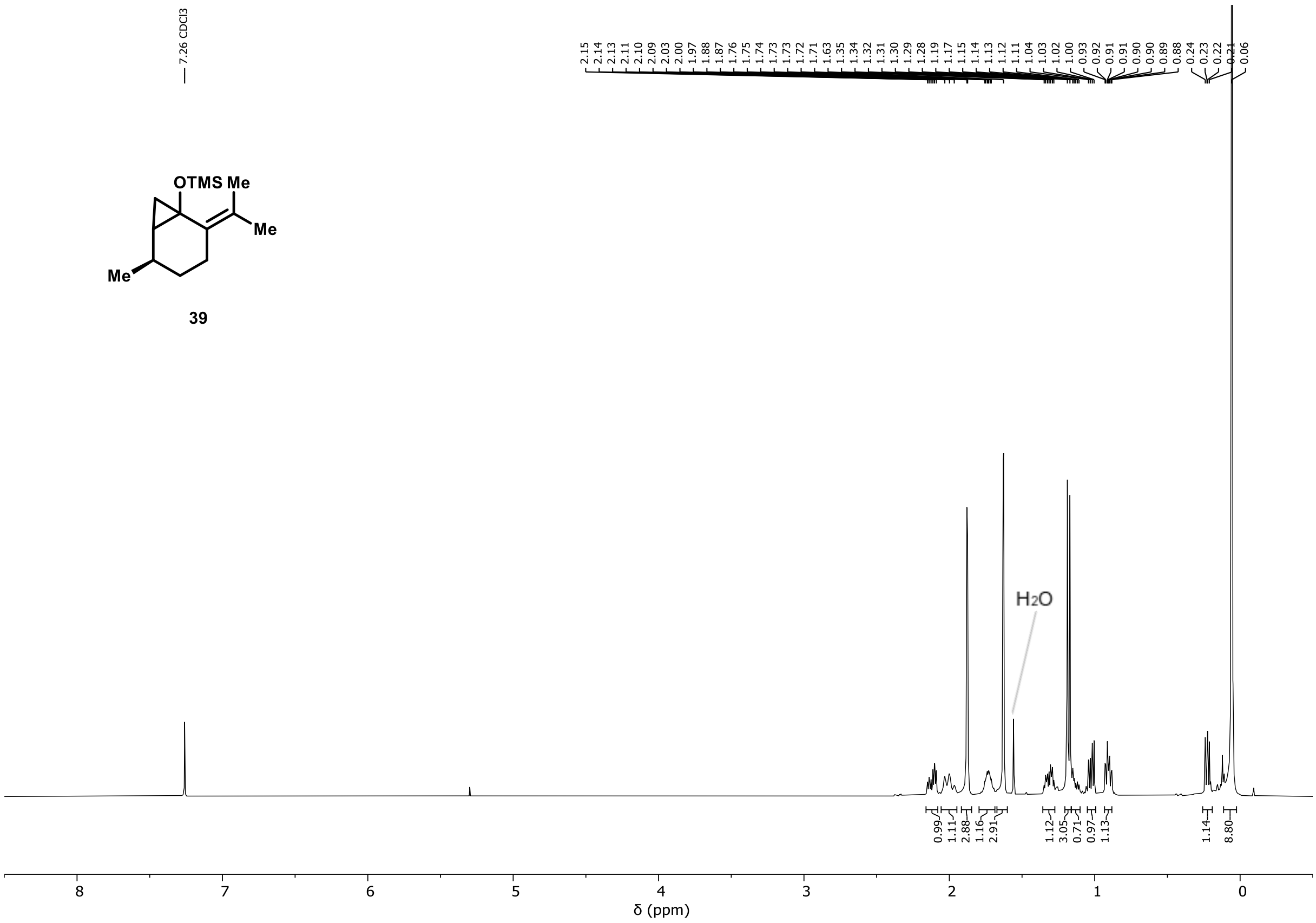
OTMS
Me
Me
Me
7.26 CDCl3
H₂O
δ (ppm)

39

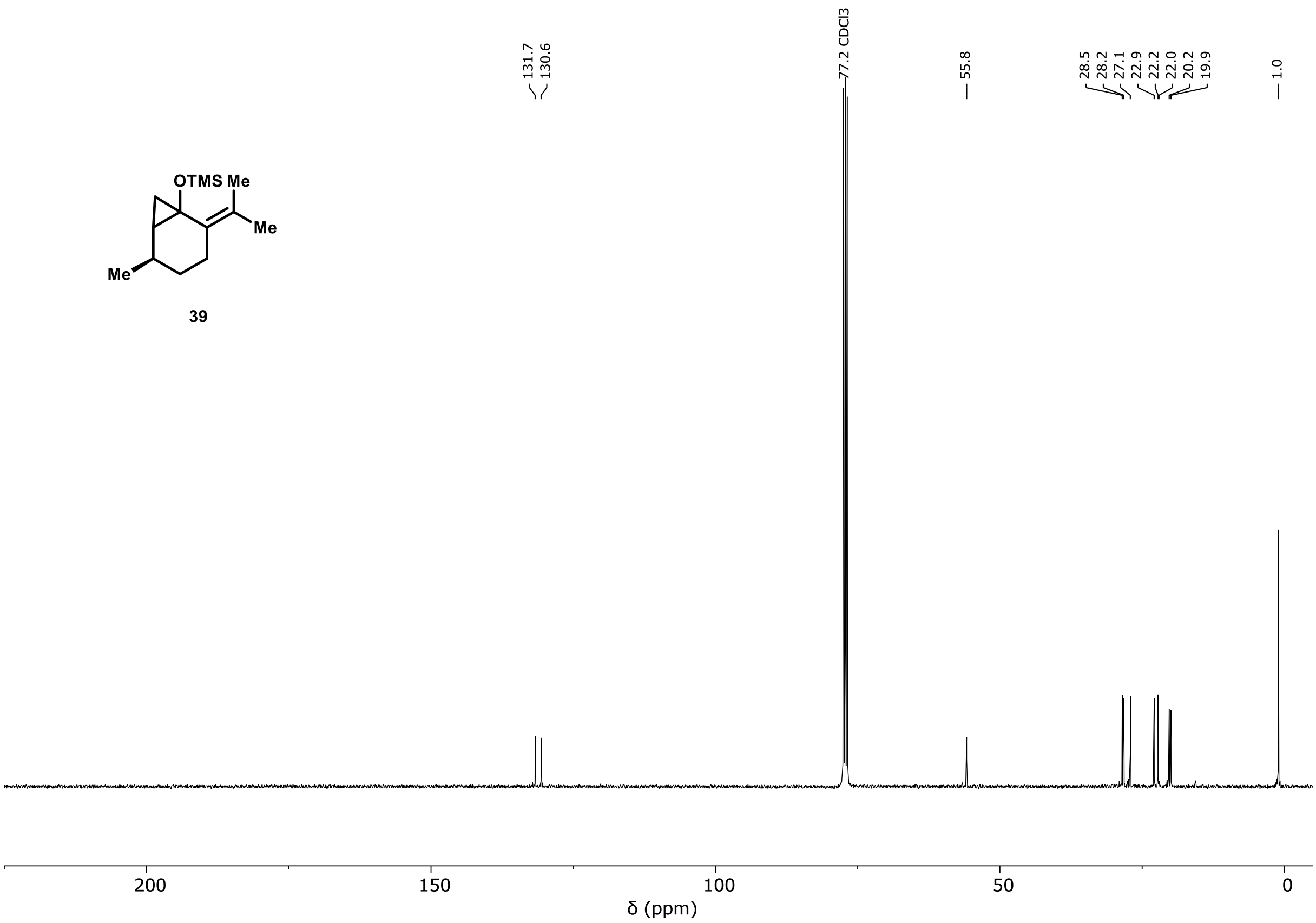
OTMS
Me
Me
Me
131.7
130.6
77.2 CDCl3
55.8
28.5
28.2
27.1
22.9
22.2
22.0
20.2
19.9
1.0
δ (ppm)

39

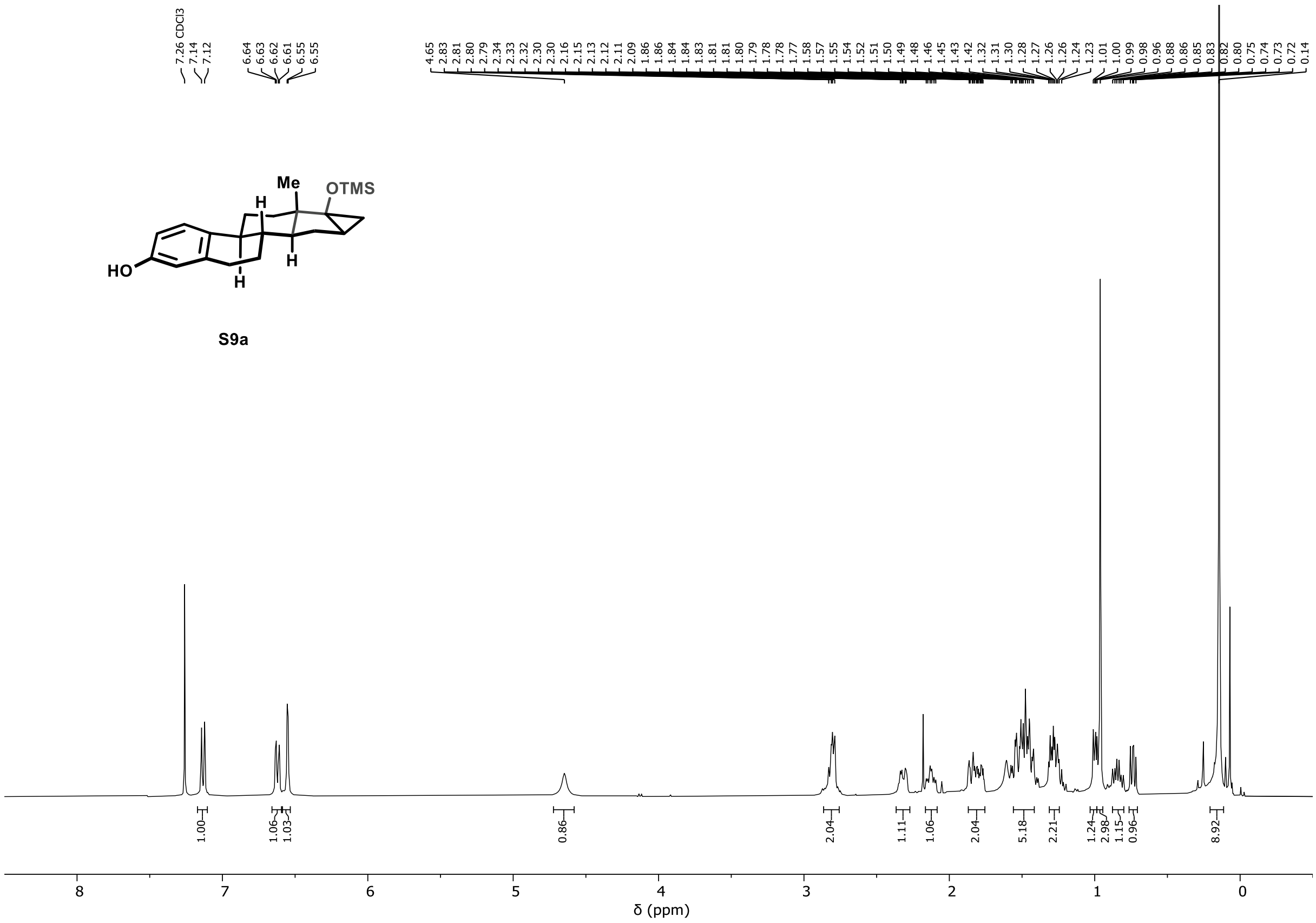
7.26 CDCl3
7.14
7.12
6.64
6.63
6.62
6.61
6.55
6.55
4.65
2.83
2.81
2.80
2.79
2.34
2.33
2.32
2.30
2.30
2.16
2.15
2.13
2.12
2.11
2.09
1.86
1.86
1.84
1.84
1.83
1.81
1.81
1.80
1.79
1.78
1.78
1.77
1.58
1.57
1.55
1.54
1.52
1.51
1.50
1.49
1.48
1.46
1.45
1.43
1.42
1.32
1.31
1.30
1.28
1.27
1.26
1.26
1.24
1.23
1.01
1.00
0.99
0.98
0.96
0.88
0.86
0.85
0.83
0.82
0.80
0.75
0.74
0.73
0.72
0.14
Me
OTMS
H
H
HO
H
S9a
1.00
1.06
1.03
0.86
2.04
1.11
1.06
2.04
5.18
2.21
1.24
2.98
1.15
0.96
8.92
δ (ppm)

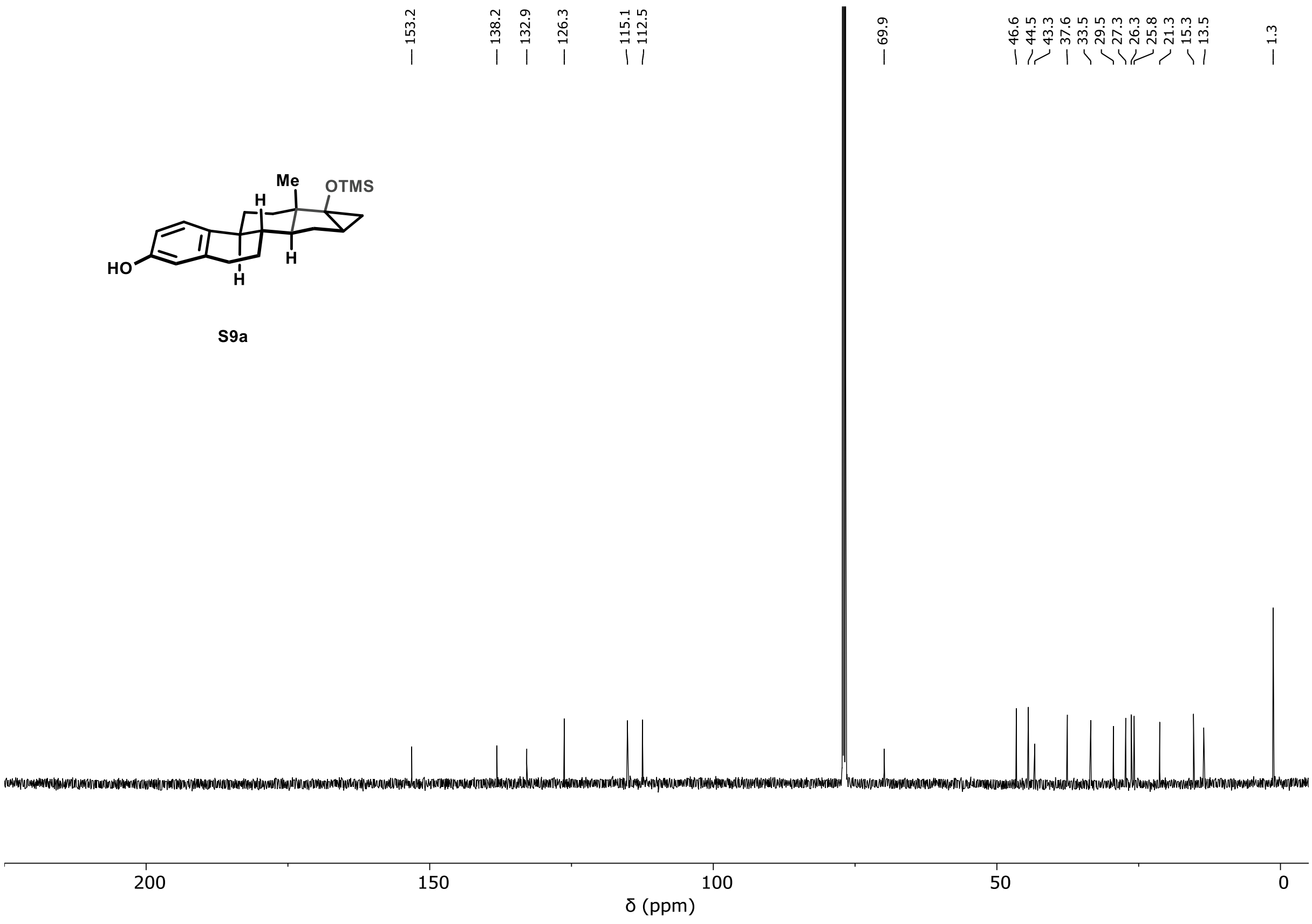
153.2
138.2
132.9
126.3
115.1
112.5
69.9
46.6
44.5
43.3
37.6
33.5
29.5
27.3
26.3
25.8
21.3
15.3
13.5
1.3
Me
OTMS
H
H
HO
H
S9a
δ (ppm)

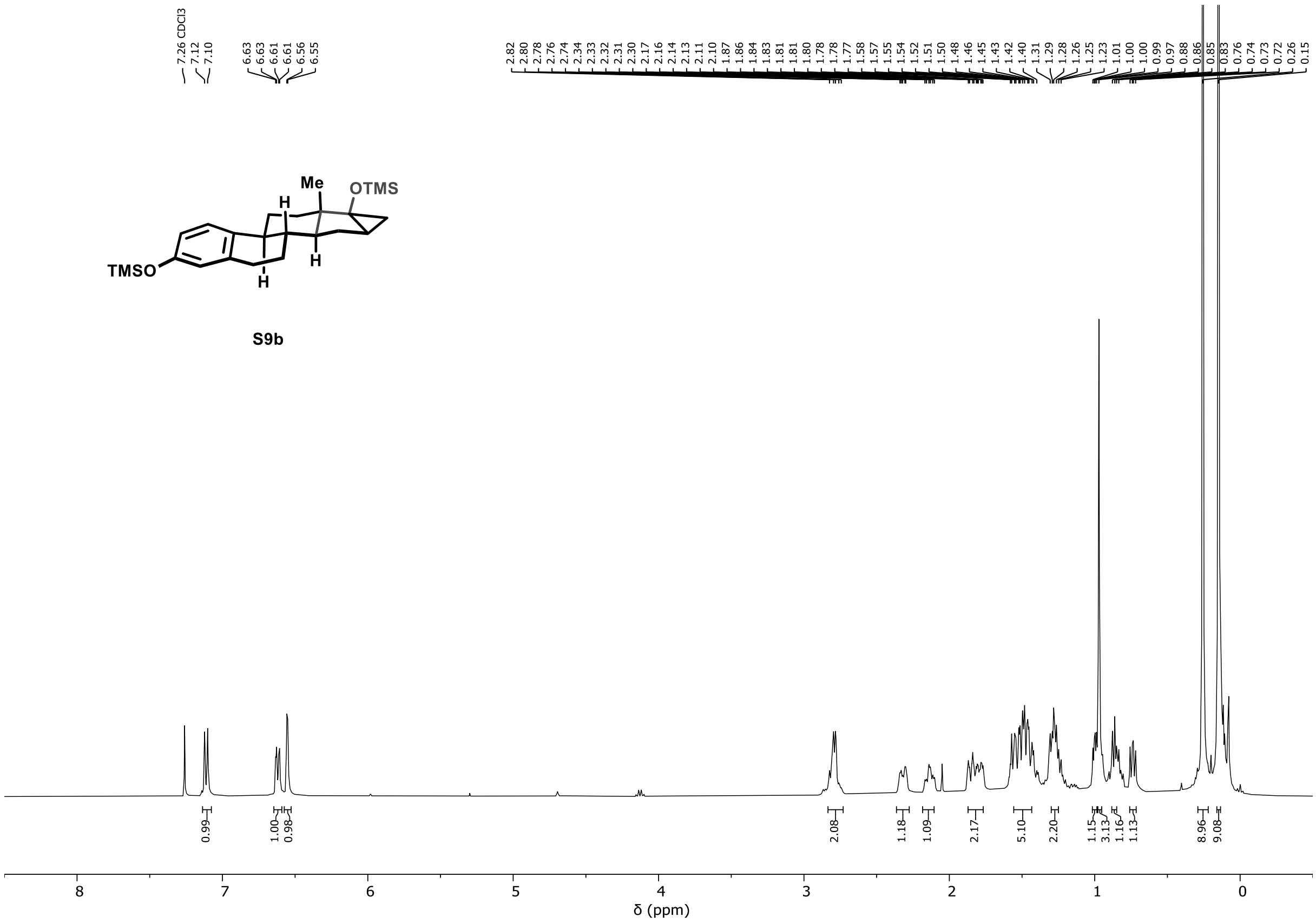
7.26 CDCl3
7.12
7.10
6.63
6.63
6.61
6.61
6.56
6.55
2.82
2.80
2.78
2.76
2.74
2.34
2.33
2.32
2.31
2.30
2.17
2.16
2.14
2.13
2.11
2.10
1.87
1.86
1.84
1.83
1.81
1.81
1.80
1.78
1.78
1.77
1.58
1.57
1.55
1.54
1.52
1.51
1.50
1.48
1.46
1.45
1.43
1.42
1.40
1.31
1.29
1.28
1.26
1.25
1.23
1.01
1.00
1.00
0.99
0.97
0.88
0.86
0.85
0.83
0.76
0.74
0.73
0.72
0.26
0.15
Me
OTMS
H
H
H
TMSO
S9b
0.99
1.00
0.98
2.08
1.18
1.09
2.17
5.10
2.20
1.15
3.13
1.16
1.13
8.96
9.08
δ (ppm)

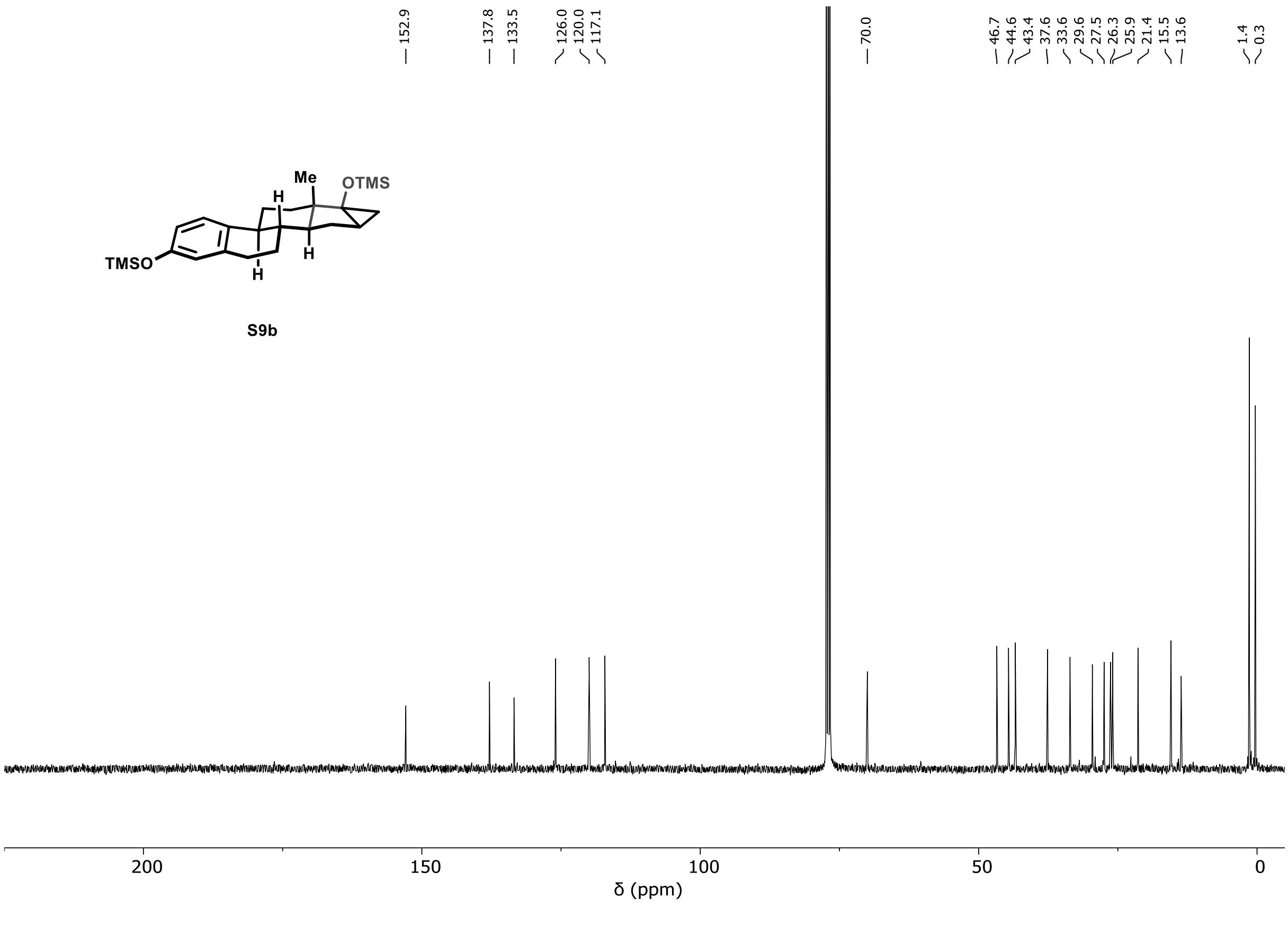
152.9
137.8
133.5
126.0
120.0
117.1
70.0
46.7
44.6
43.4
37.6
33.6
29.6
27.5
26.3
25.9
21.4
15.5
13.6
1.4
0.3
Me
OTMS
H
H
H
TMSO
S9b
δ (ppm)

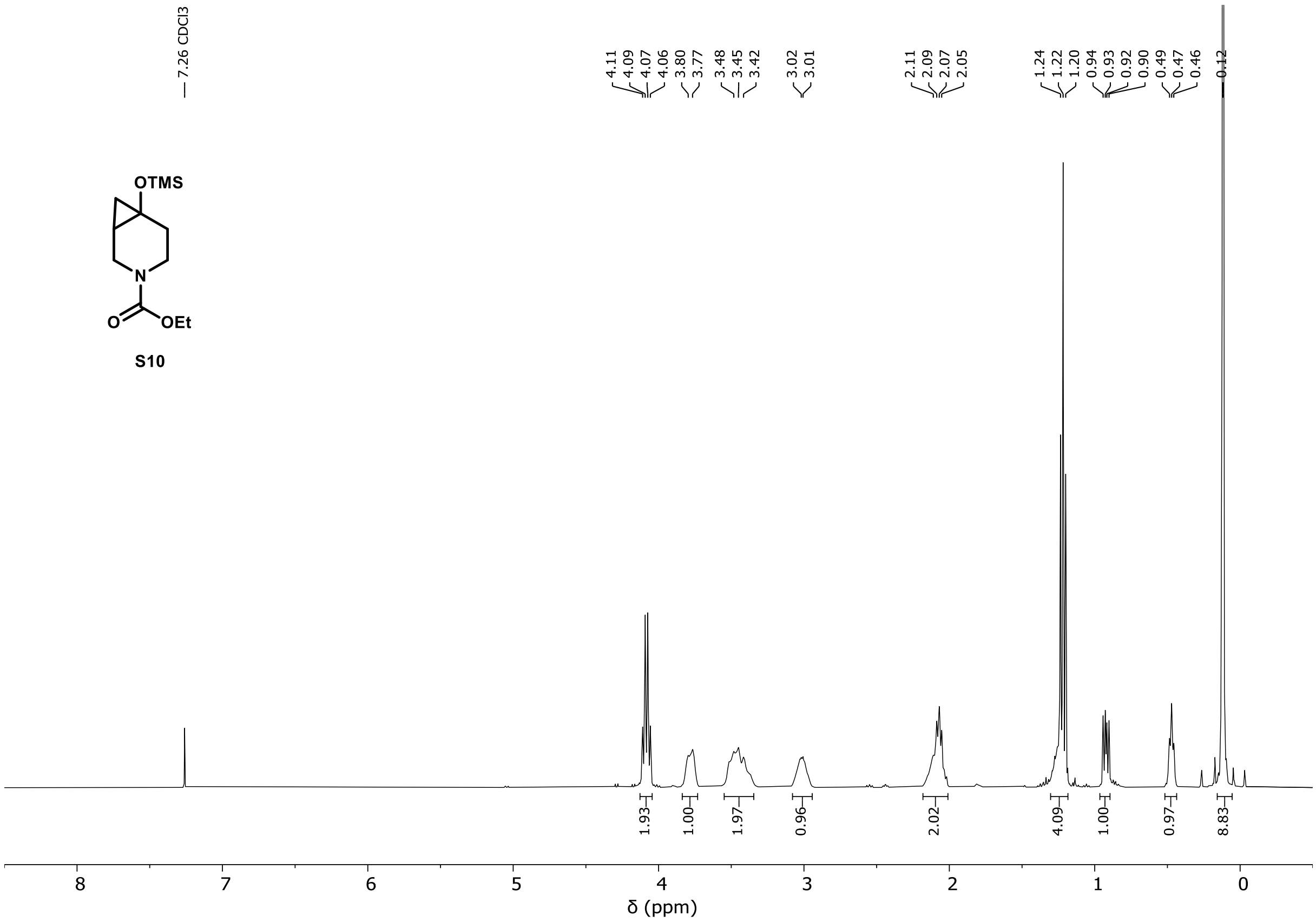
7.26 CDCl3
4.11
4.09
4.07
4.06
3.80
3.77
3.48
3.45
3.42
3.02
3.01
2.11
2.09
2.07
2.05
1.24
1.22
1.20
0.94
0.93
0.92
0.90
0.49
0.47
0.46
0.12
OTMS
N
O
OEt
S10
1.93
1.00
1.97
0.96
2.02
4.09
1.00
0.97
8.83
8 7 6 5 4 3 2 1 0
δ (ppm)

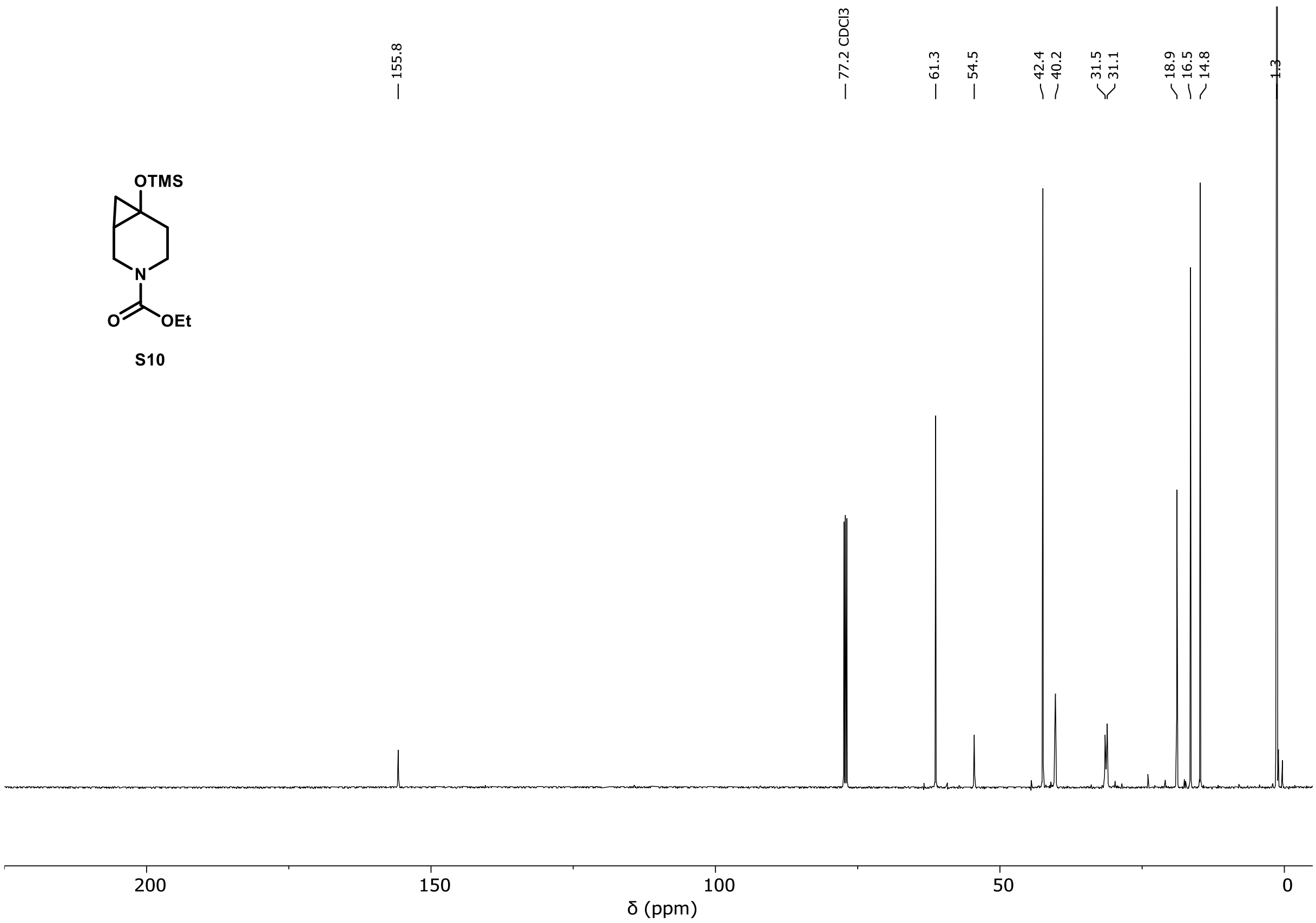
155.8
77.2 CDCl3
61.3
54.5
42.4
40.2
31.5
31.1
18.9
16.5
14.8
1.3
OTMS
N
O
OEt
S10
200 150 100 50 0
δ (ppm)

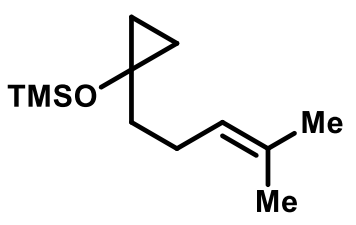

3

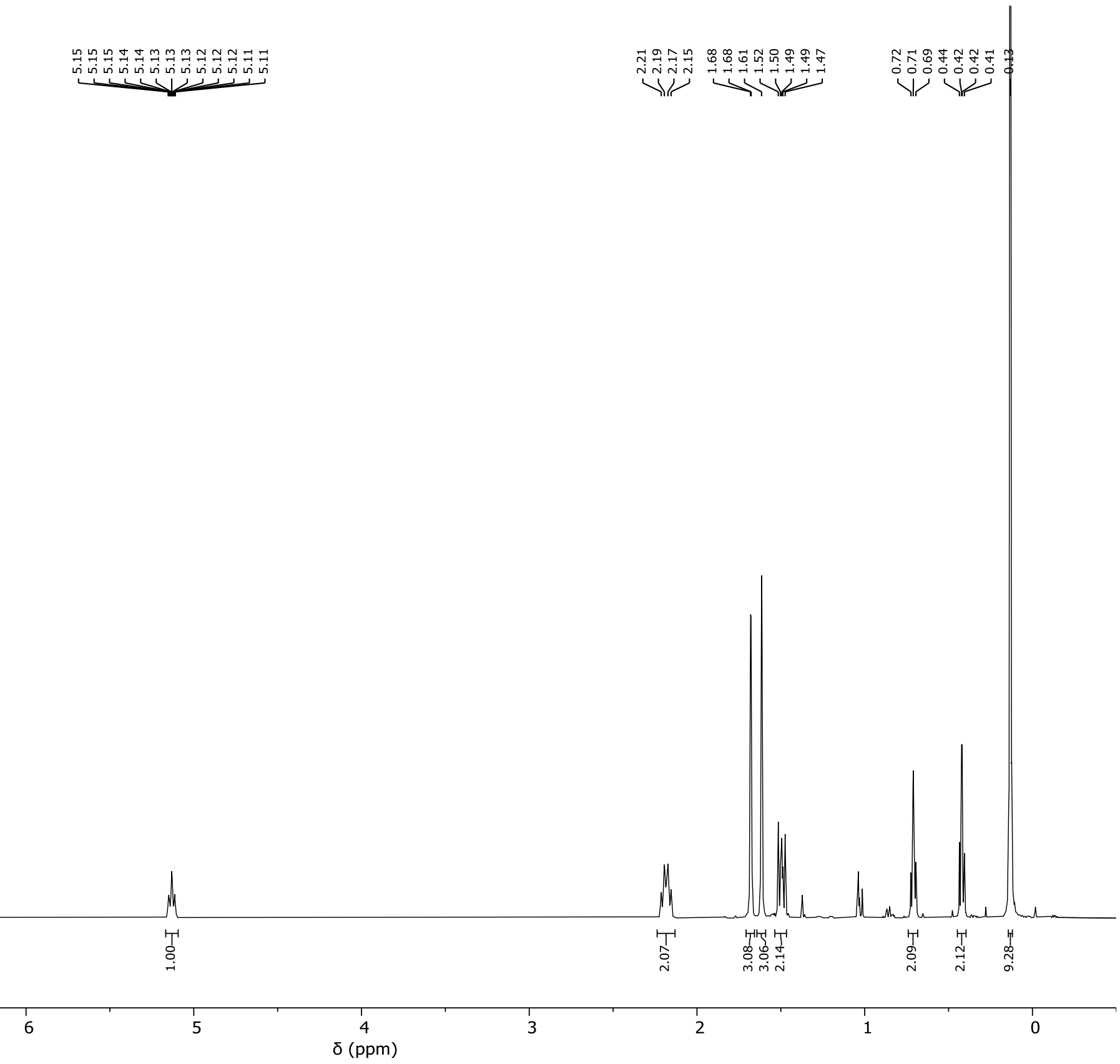

7.26 CDCl3
5.15
5.15
5.15
5.14
5.14
5.13
5.13
5.13
5.12
5.12
5.12
5.11
5.11
2.21
2.19
2.17
2.15
1.68
1.68
1.61
1.52
1.50
1.49
1.49
1.47
0.72
0.71
0.69
0.44
0.42
0.42
0.41
0.13
1.00
2.07
3.08
3.06
2.14
2.09
2.12
9.28
δ (ppm)

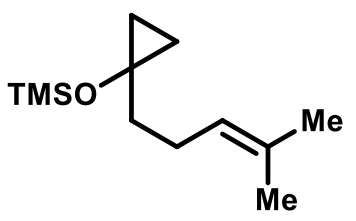

3

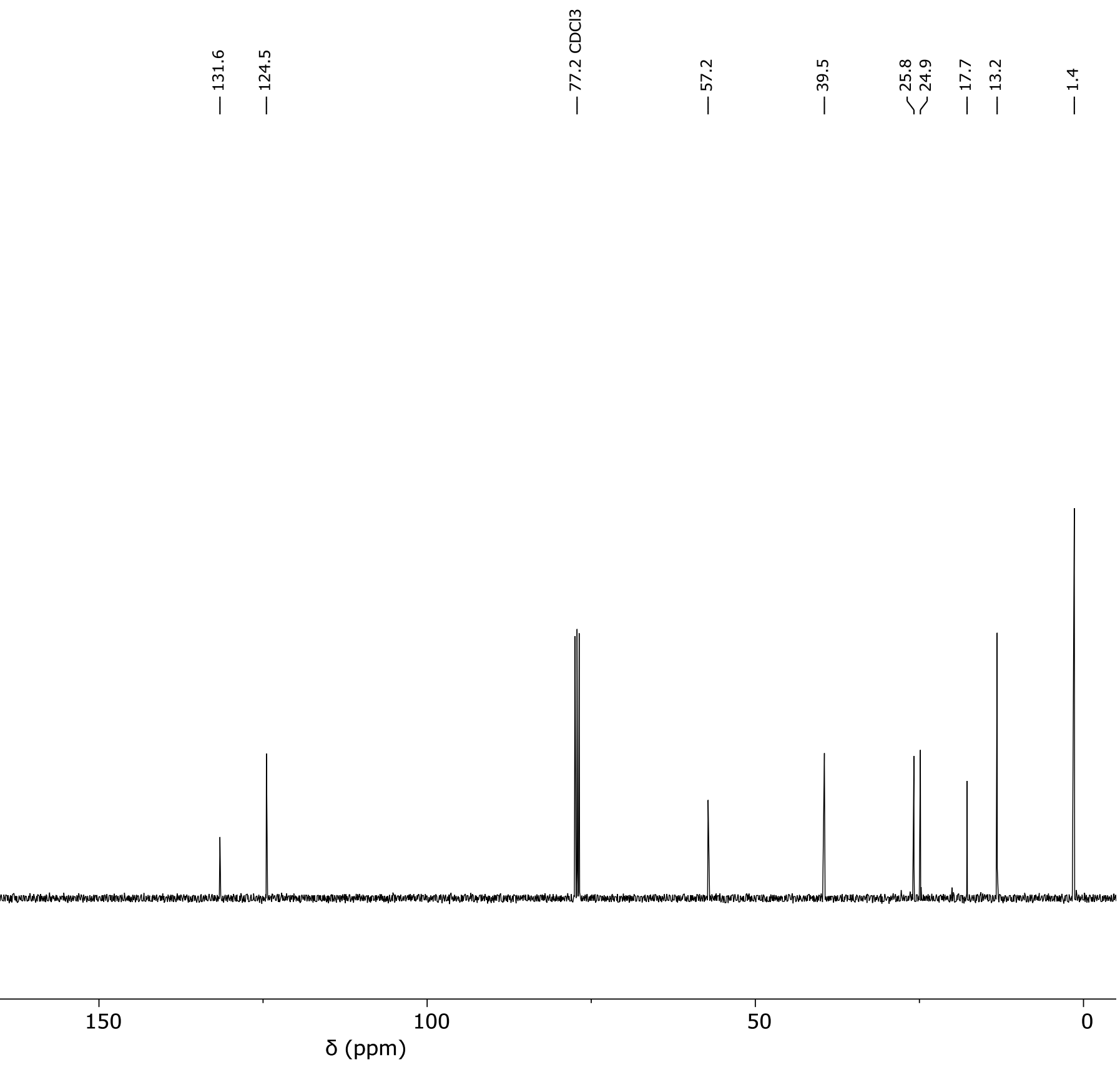

131.6
124.5
77.2 CDCl3
57.2
39.5
25.8
24.9
17.7
13.2
1.4
δ (ppm)

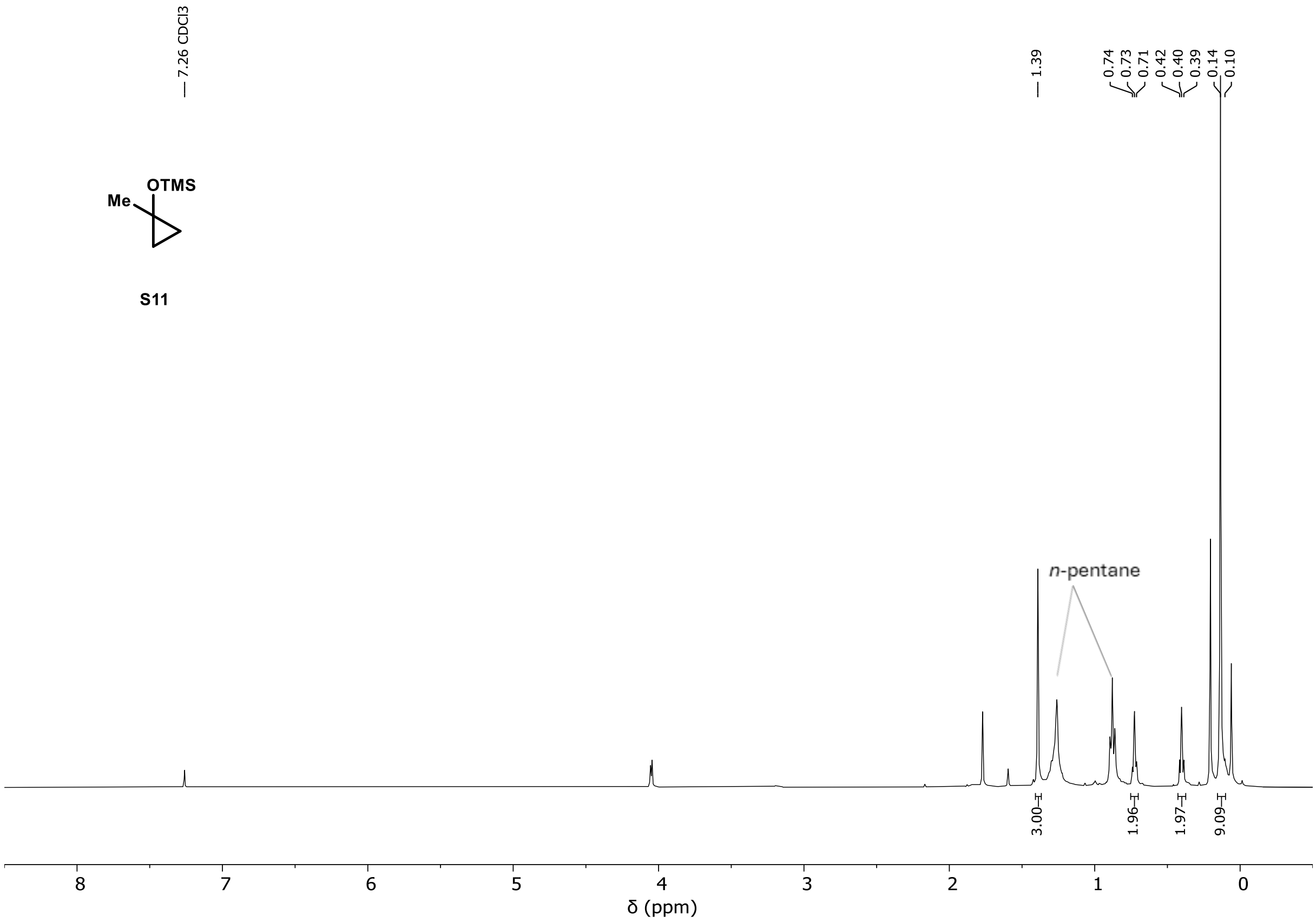
7.26 CDCl3
1.39
0.74
0.73
0.71
0.42
0.40
0.39
0.14
0.10
Me
OTMS
S11
n-pentane
3.00
1.96
1.97
9.09
δ (ppm)

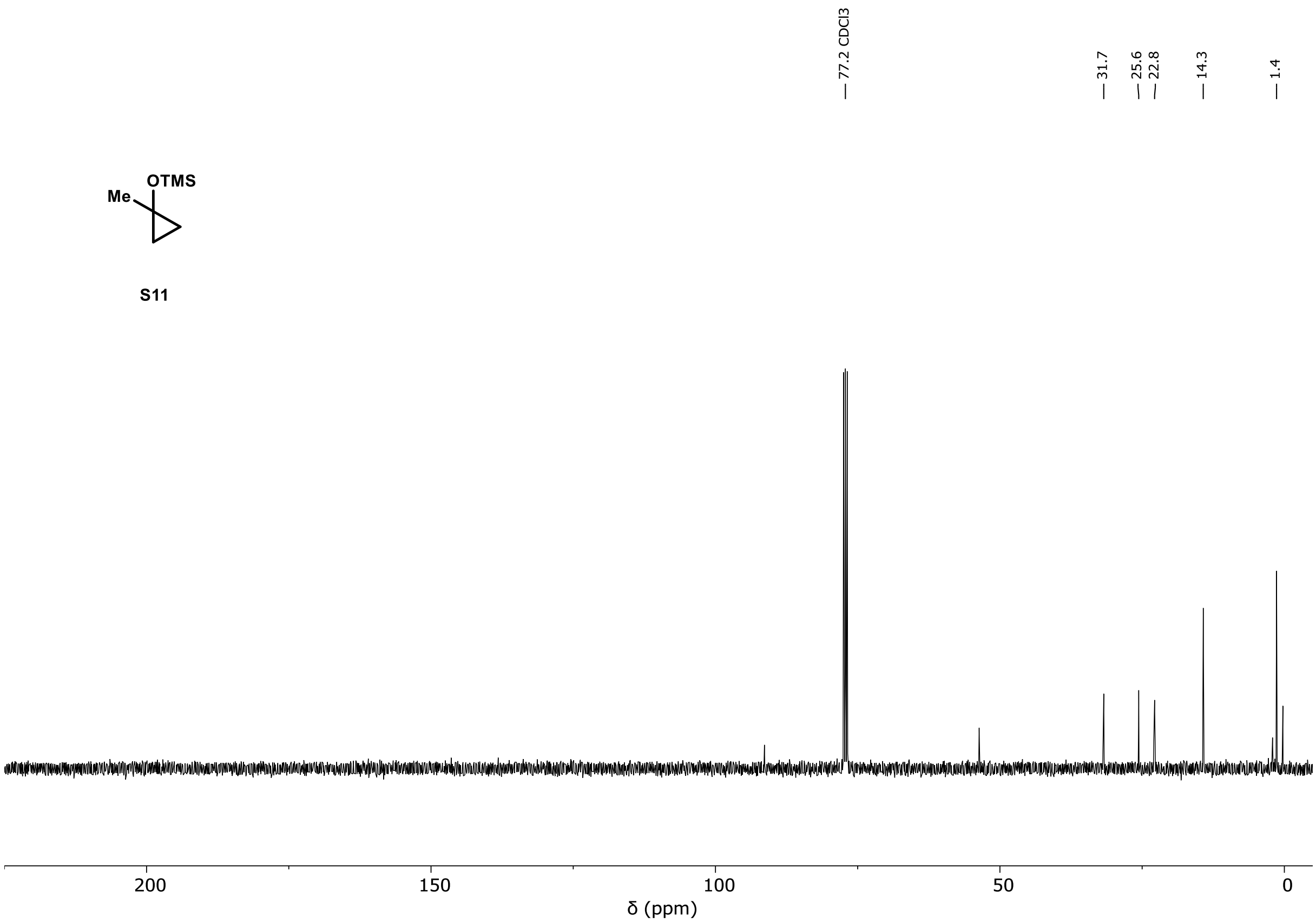
77.2 CDCl3
31.7
25.6
22.8
14.3
1.4
Me
OTMS
S11
δ (ppm)

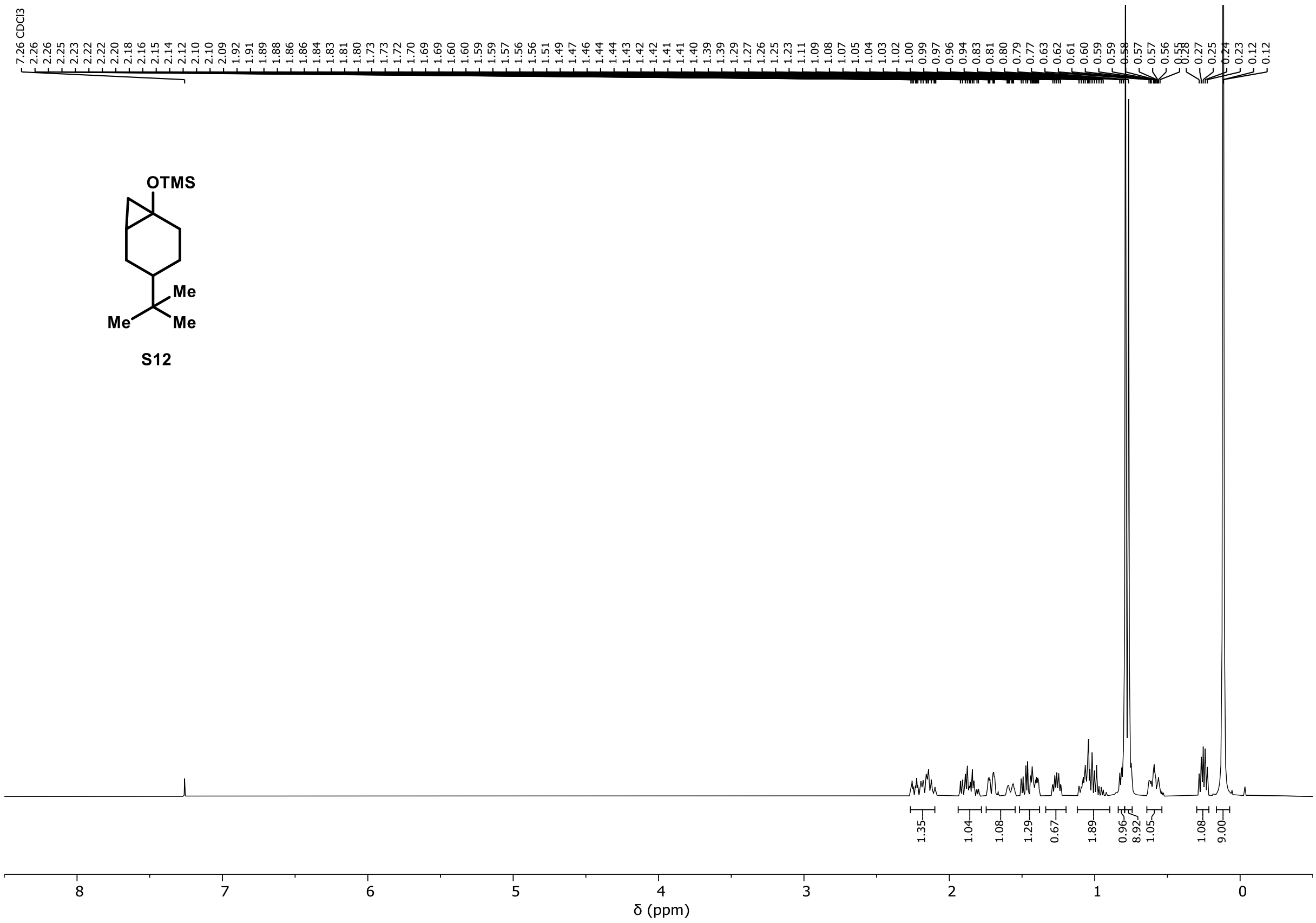

7.26 CDCl3
2.26
2.26
2.25
2.23
2.22
2.22
2.20
2.18
2.16
2.15
2.14
2.12
2.10
2.10
2.09
1.92
1.91
1.89
1.88
1.86
1.86
1.84
1.83
1.81
1.80
1.73
1.73
1.72
1.70
1.69
1.69
1.60
1.60
1.59
1.59
1.57
1.56
1.56
1.51
1.49
1.47
1.46
1.44
1.44
1.43
1.42
1.41
1.41
1.40
1.39
1.39
1.29
1.27
1.26
1.25
1.23
1.11
1.09
1.08
1.07
1.05
1.04
1.03
1.02
1.00
0.99
0.97
0.96
0.94
0.83
0.81
0.80
0.79
0.77
0.63
0.62
0.61
0.60
0.59
0.59
0.58
0.57
0.57
0.56
0.55
0.28
0.27
0.25
0.24
0.23
0.12
0.12
1.35
1.04
1.08
1.29
0.67
1.89
0.96
8.92
1.05
1.08
9.00
8
7
6
5
4
3
2
1
0
δ (ppm)
OTMS
Me
Me
Me

S12

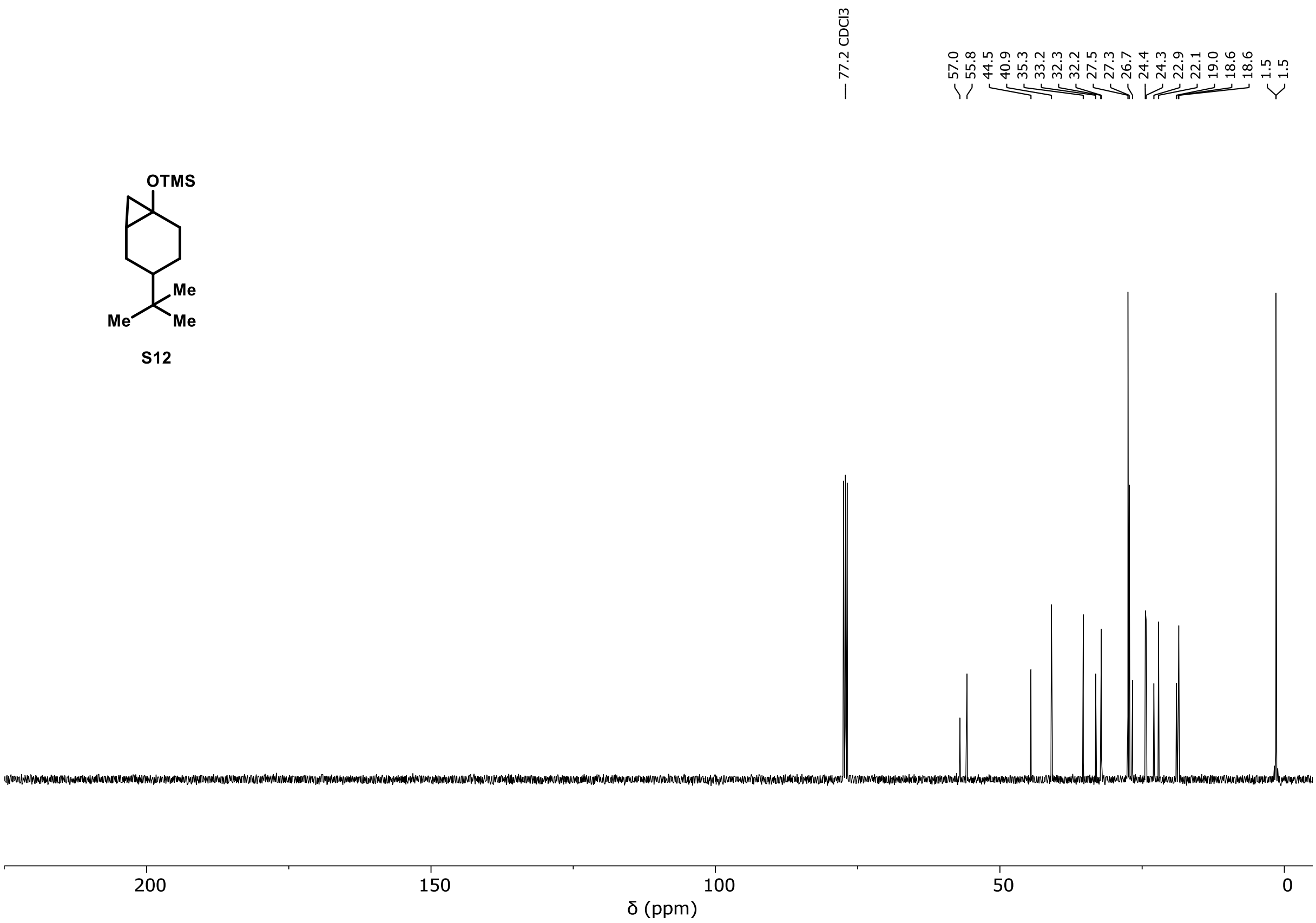

77.2 CDCl3
57.0
55.8
44.5
40.9
35.3
33.2
32.3
32.2
27.5
27.3
26.7
24.4
24.3
22.9
22.1
19.0
18.6
18.6
1.5
1.5
200
150
100
50
0
δ (ppm)
OTMS
Me
Me
Me

S12

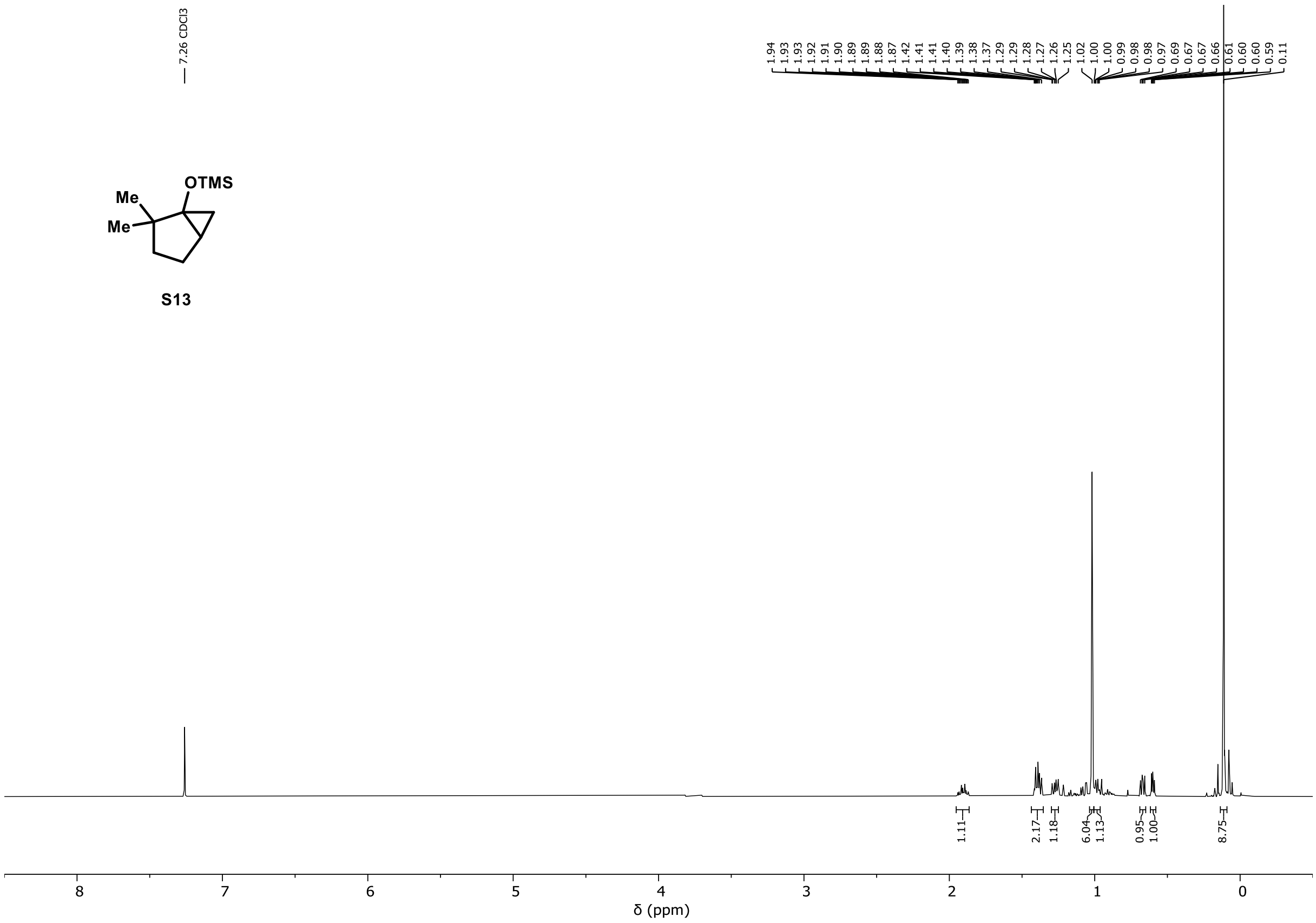
7.26 CDCl3
1.94
1.93
1.93
1.92
1.91
1.90
1.89
1.89
1.88
1.87
1.42
1.41
1.41
1.40
1.39
1.38
1.37
1.29
1.29
1.28
1.27
1.26
1.25
1.02
1.00
1.00
0.99
0.98
0.98
0.97
0.69
0.67
0.67
0.66
0.61
0.60
0.60
0.59
0.11
OTMS
Me
Me
S13
1.11
2.17
1.18
6.04
1.13
0.95
1.00
8.75
8
7
6
5
4
3
2
1
0
δ (ppm)

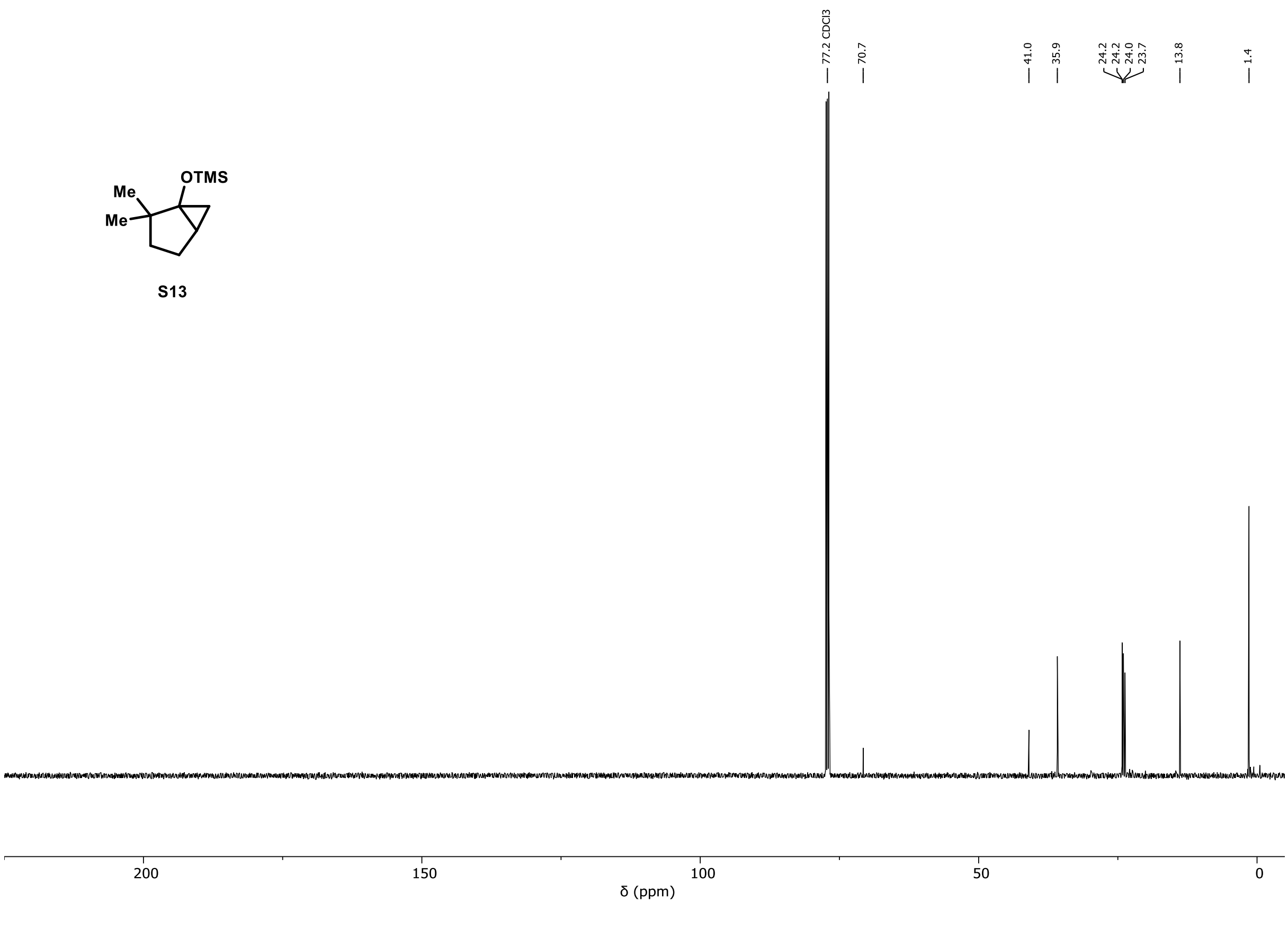
77.2 CDCl3
70.7
41.0
35.9
24.2
24.2
24.0
23.7
13.8
1.4
OTMS
Me
Me
S13
200
150
100
50
0
δ (ppm)

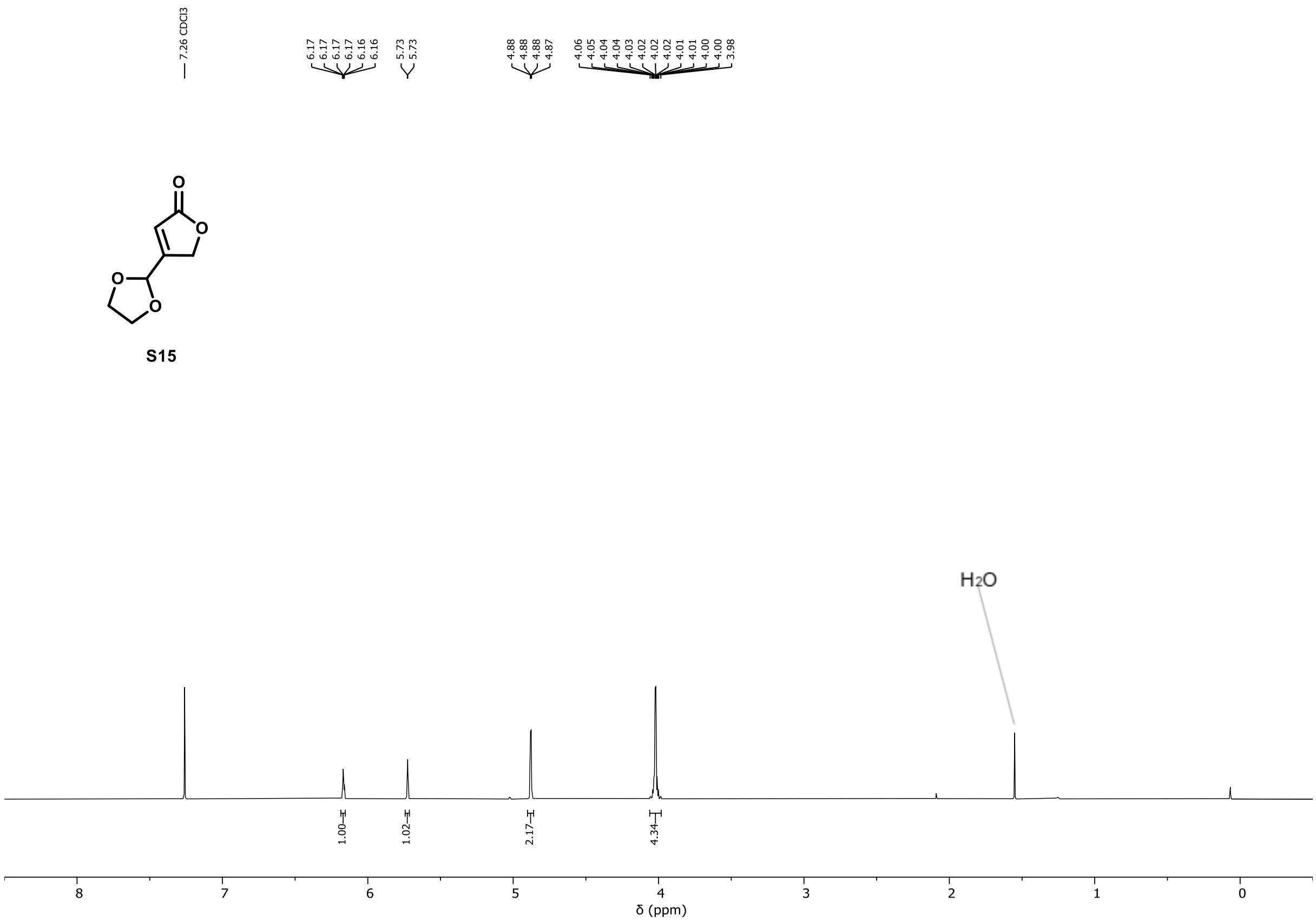
7.26 CDCl3
6.17
6.17
6.17
6.17
6.16
6.16
5.73
5.73
4.88
4.88
4.88
4.87
4.06
4.05
4.04
4.04
4.03
4.02
4.02
4.02
4.01
4.01
4.00
4.00
3.98
H2O
1.00
1.02
2.17
4.34
8
7
6
5
4
3
2
1
0
δ (ppm)

S15

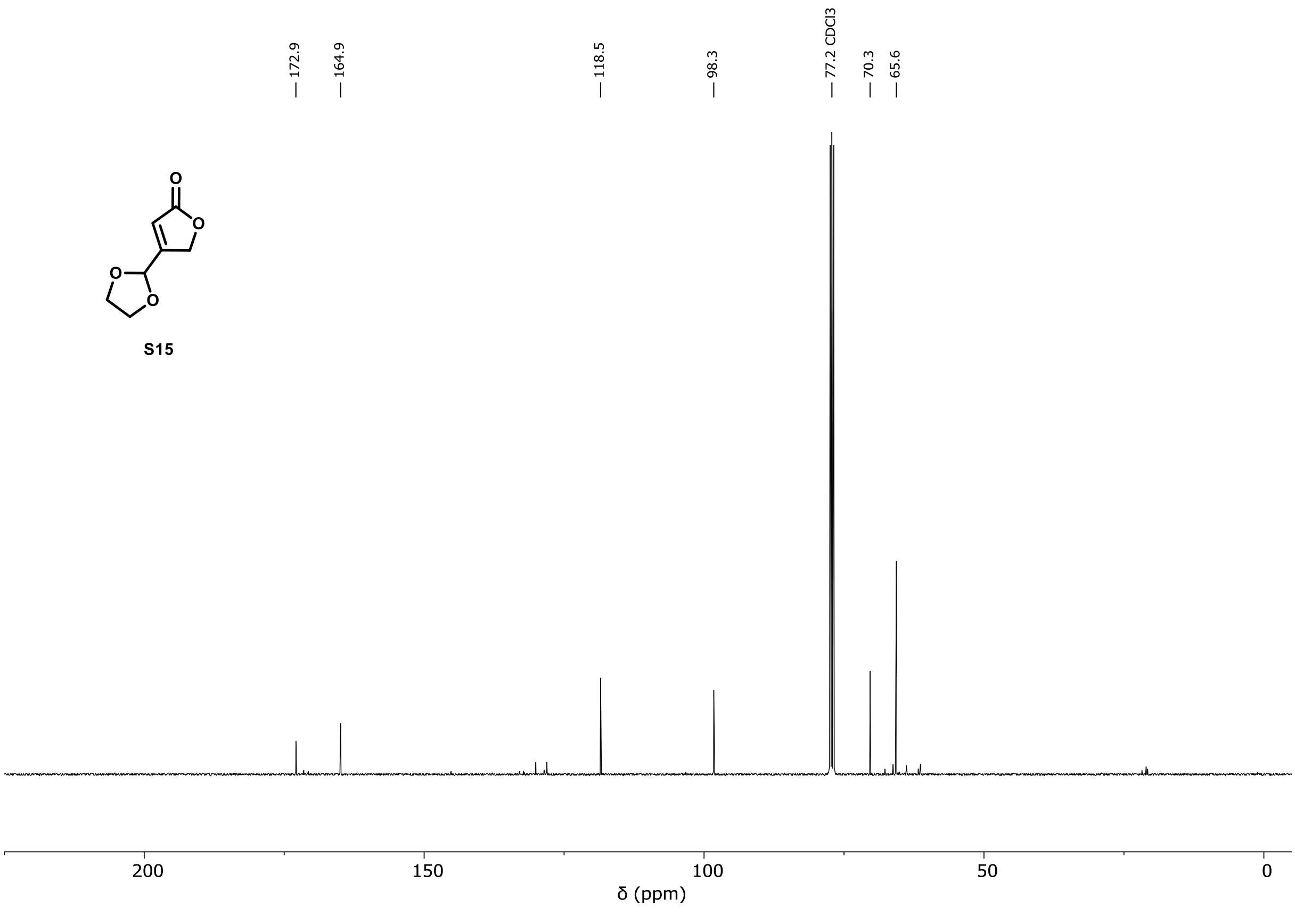
172.9
164.9
118.5
98.3
77.2 CDCl3
70.3
65.6
200
150
100
50
0
δ (ppm)

S15

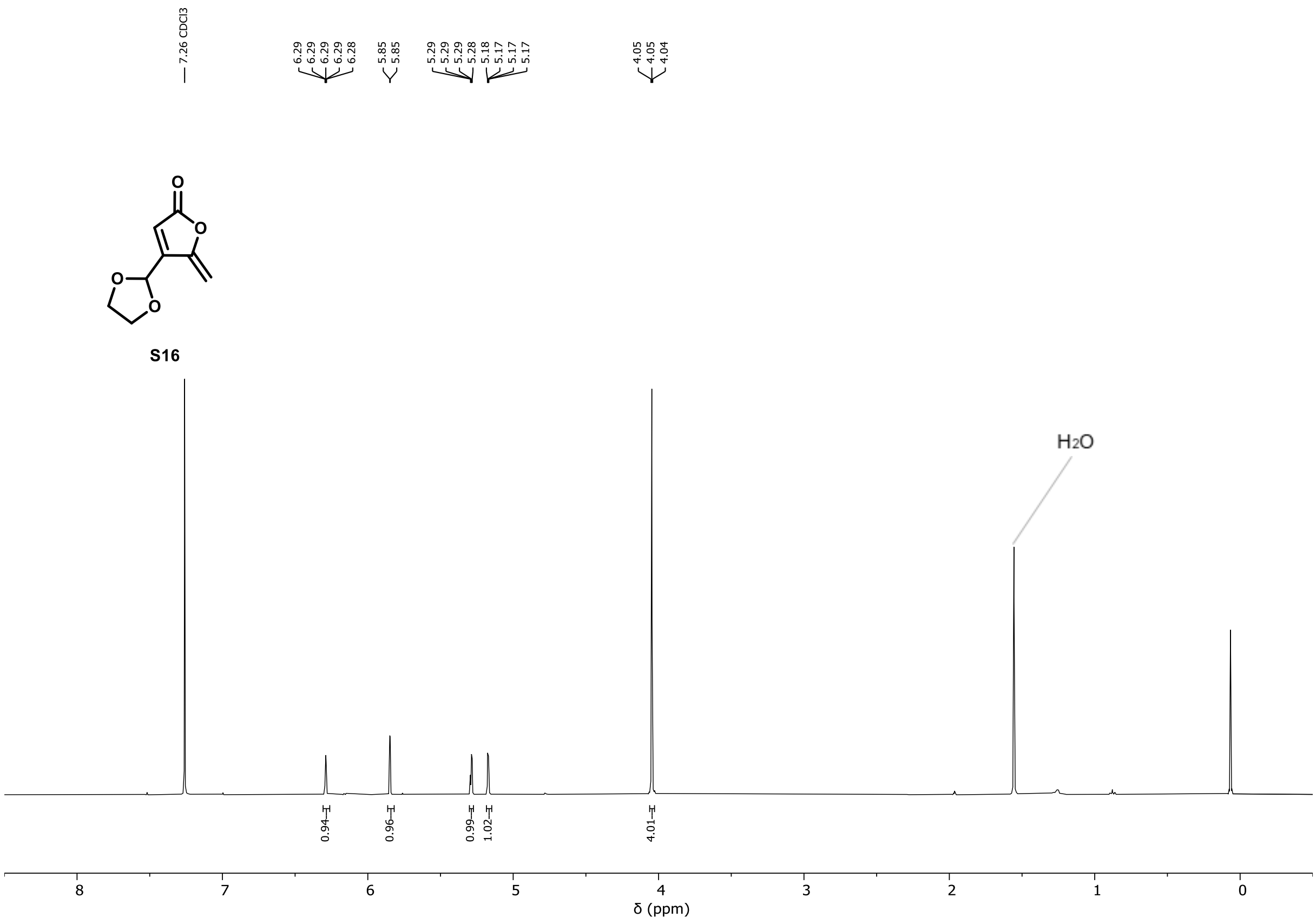
7.26 CDCl3
6.29
6.29
6.29
6.29
6.28
5.85
5.85
5.29
5.29
5.29
5.28
5.18
5.17
5.17
5.17
4.05
4.05
4.04
S16
H2O
0.94
0.96
0.99
1.02
4.01
δ (ppm)

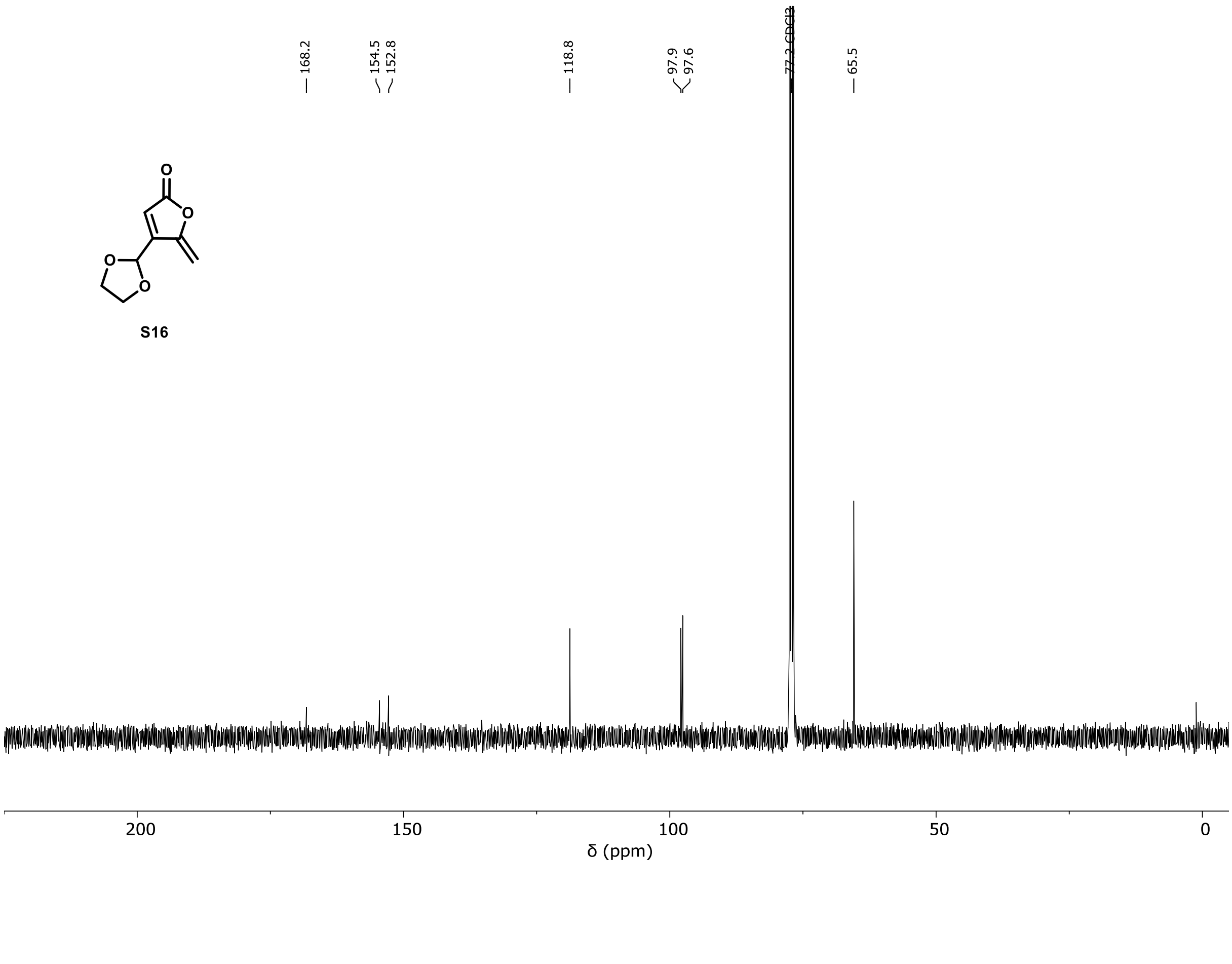
168.2
154.5
152.8
118.8
97.9
97.6
77.2 CDCl3
65.5
S16
δ (ppm)

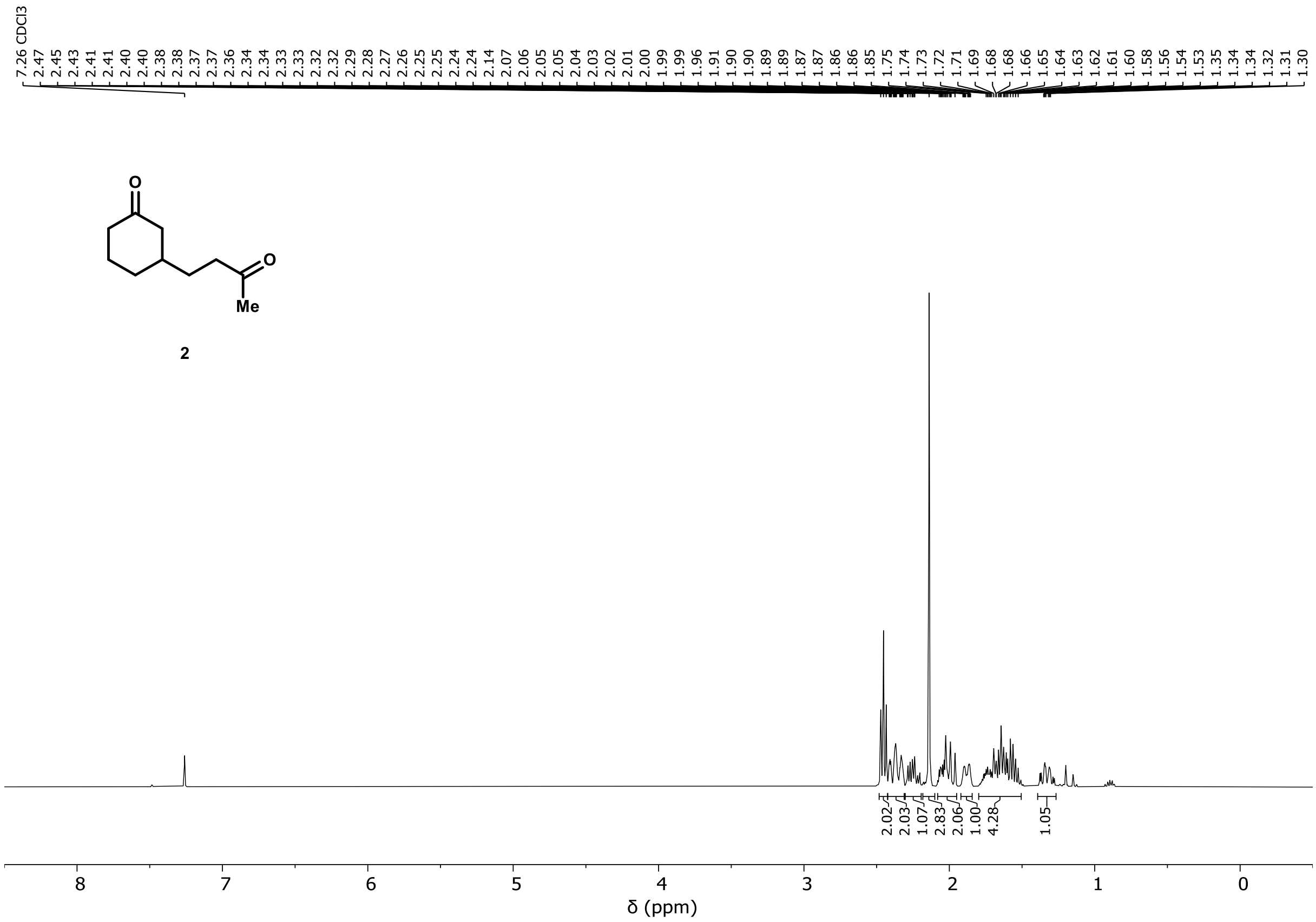
7.26 CDCl3
2.47
2.45
2.43
2.41
2.41
2.40
2.40
2.38
2.38
2.37
2.37
2.36
2.34
2.34
2.33
2.33
2.32
2.32
2.29
2.28
2.27
2.26
2.25
2.25
2.24
2.24
2.14
2.07
2.06
2.05
2.05
2.04
2.03
2.02
2.01
2.00
1.99
1.99
1.96
1.91
1.90
1.90
1.89
1.89
1.87
1.87
1.86
1.86
1.85
1.75
1.74
1.73
1.72
1.71
1.69
1.68
1.68
1.66
1.65
1.64
1.63
1.62
1.61
1.60
1.58
1.56
1.54
1.53
1.35
1.34
1.34
1.32
1.31
1.30
O
O
Me
2
2.02
2.03
1.07
2.83
2.06
1.00
4.28
1.05
8
7
6
5
4
3
2
1
0
δ (ppm)

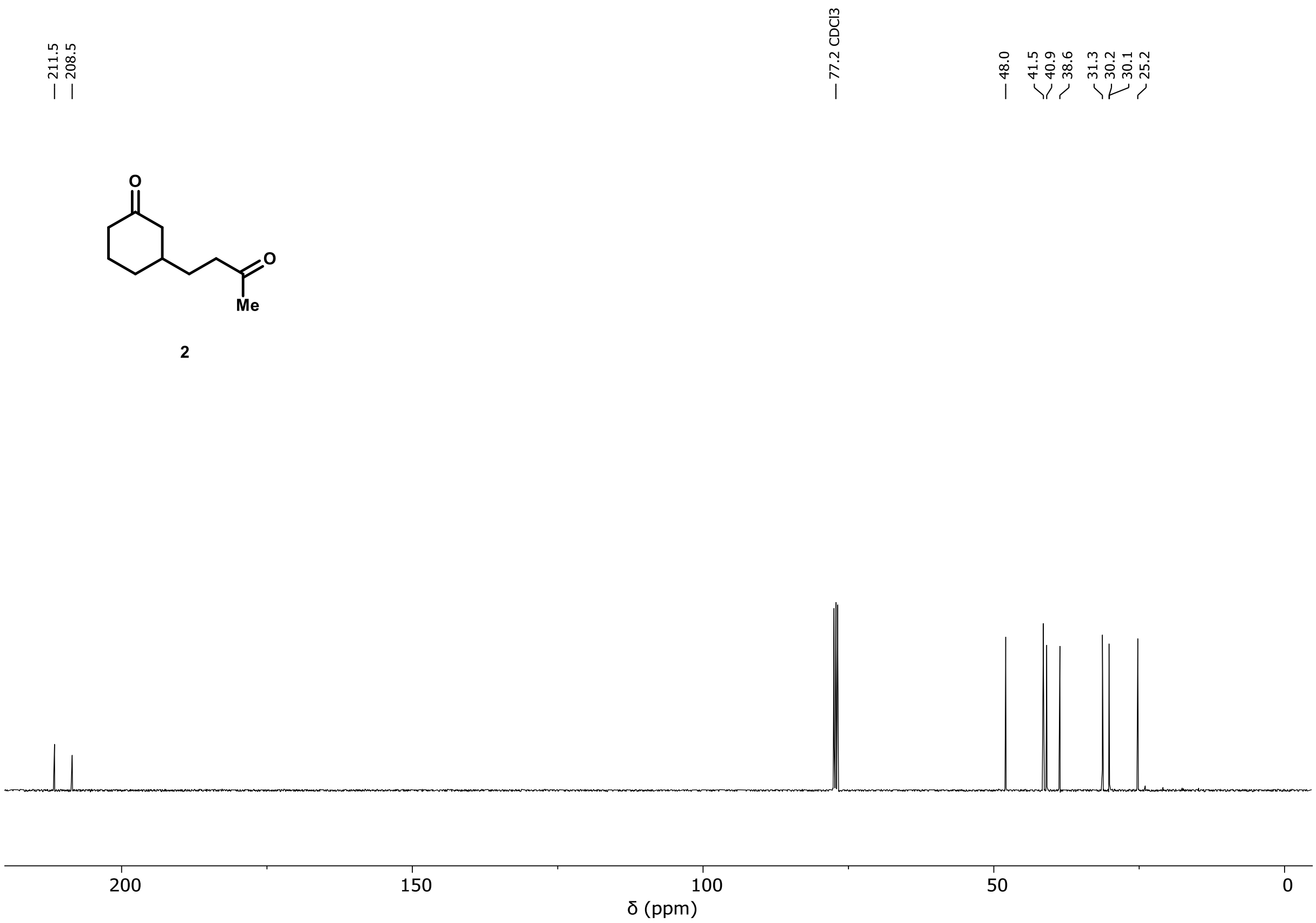
211.5
208.5
77.2 CDCl3
48.0
41.5
40.9
38.6
31.3
30.2
30.1
25.2
O
O
Me
2
200
150
100
50
0
δ (ppm)

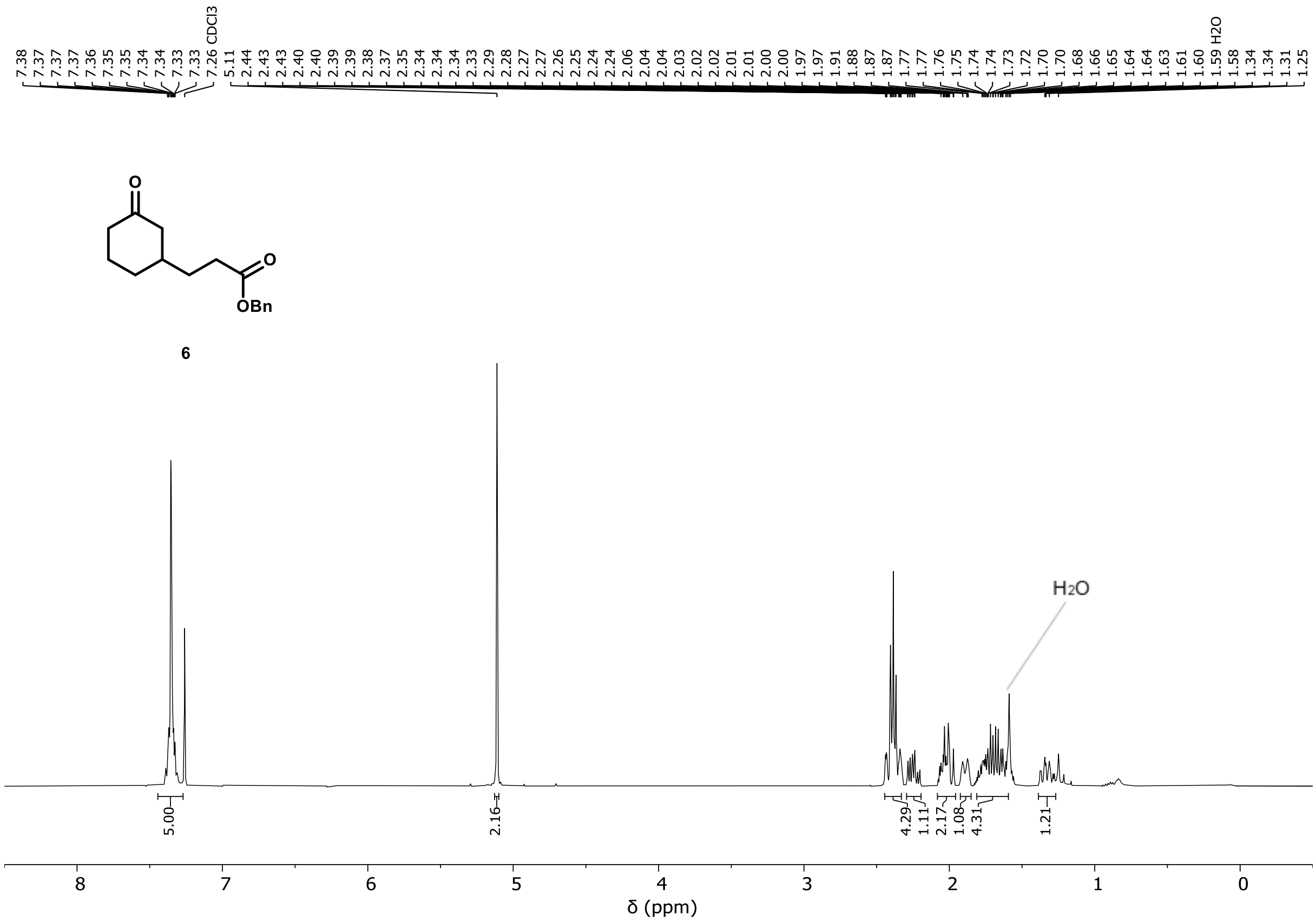
7.38 7.37 7.37 7.37 7.36 7.35 7.35 7.34 7.34 7.33 7.33 7.26 CDCl3 5.11 2.44 2.43 2.43 2.40 2.40 2.39 2.39 2.38 2.37 2.35 2.34 2.34 2.34 2.33 2.29 2.28 2.27 2.27 2.26 2.25 2.24 2.24 2.06 2.04 2.04 2.03 2.02 2.02 2.01 2.01 2.00 2.00 1.97 1.97 1.91 1.88 1.87 1.87 1.77 1.77 1.76 1.75 1.74 1.74 1.73 1.72 1.70 1.70 1.68 1.66 1.65 1.64 1.64 1.63 1.61 1.60 1.59 H2O 1.58 1.34 1.34 1.31 1.25
O
O
OBn
H2O
5.00
2.16
4.29
1.11
2.17
1.08
4.31
1.21
δ (ppm)

6

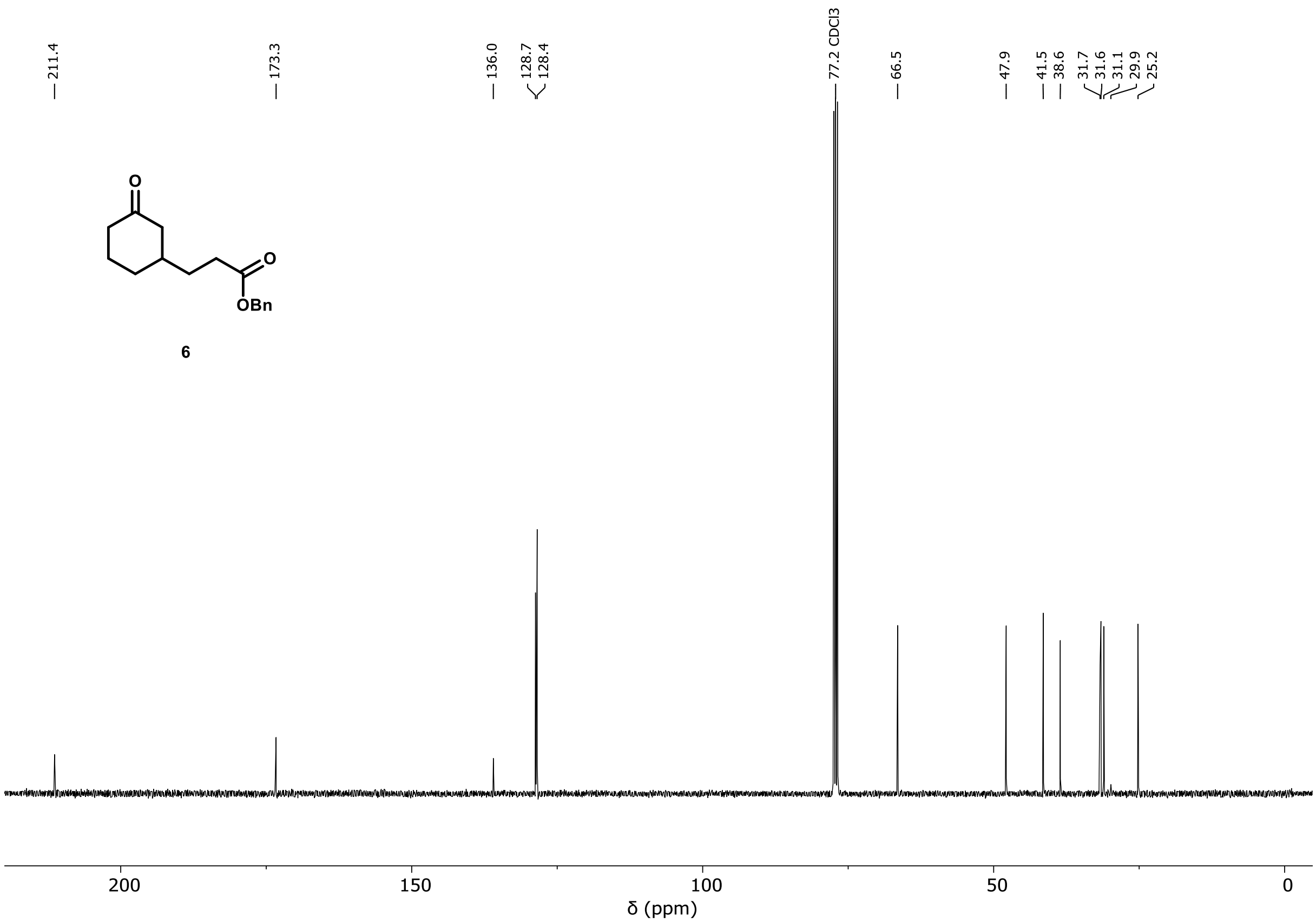
211.4
173.3
136.0
128.7
128.4
77.2 CDCl3
66.5
47.9
41.5
38.6
31.7
31.6
31.1
29.9
25.2
O
O
OBn
δ (ppm)

6

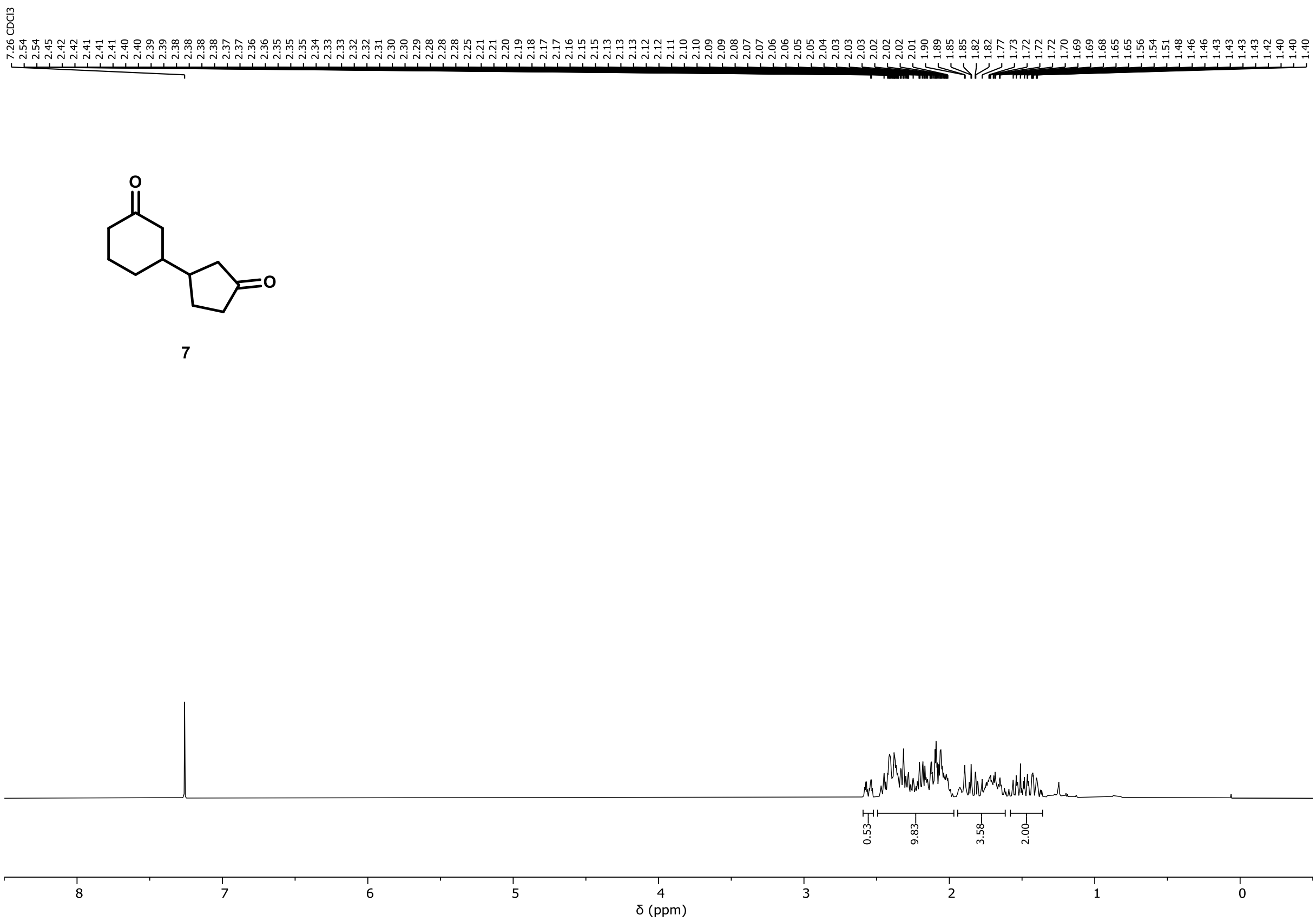
7.26 CDCl3
2.54 2.54 2.45 2.42 2.42 2.41 2.41 2.41 2.40 2.39 2.39 2.38 2.38 2.38 2.38 2.37 2.37 2.36 2.36 2.35 2.35 2.35 2.34 2.33 2.33 2.32 2.32 2.31 2.30 2.30 2.29 2.28 2.28 2.28 2.25 2.21 2.21 2.20 2.19 2.18 2.17 2.17 2.16 2.15 2.15 2.13 2.13 2.13 2.12 2.12 2.11 2.10 2.09 2.09 2.08 2.07 2.07 2.06 2.06 2.05 2.05 2.04 2.03 2.03 2.02 2.02 2.02 2.01 1.90 1.89 1.85 1.85 1.82 1.82 1.77 1.73 1.72 1.72 1.72 1.70 1.69 1.69 1.68 1.65 1.65 1.56 1.54 1.51 1.48 1.46 1.43 1.43 1.43 1.43 1.42 1.40 1.40 1.40
O
O
7
0.53
9.83
3.58
2.00
8
7
6
5
4
3
2
1
0
δ (ppm)

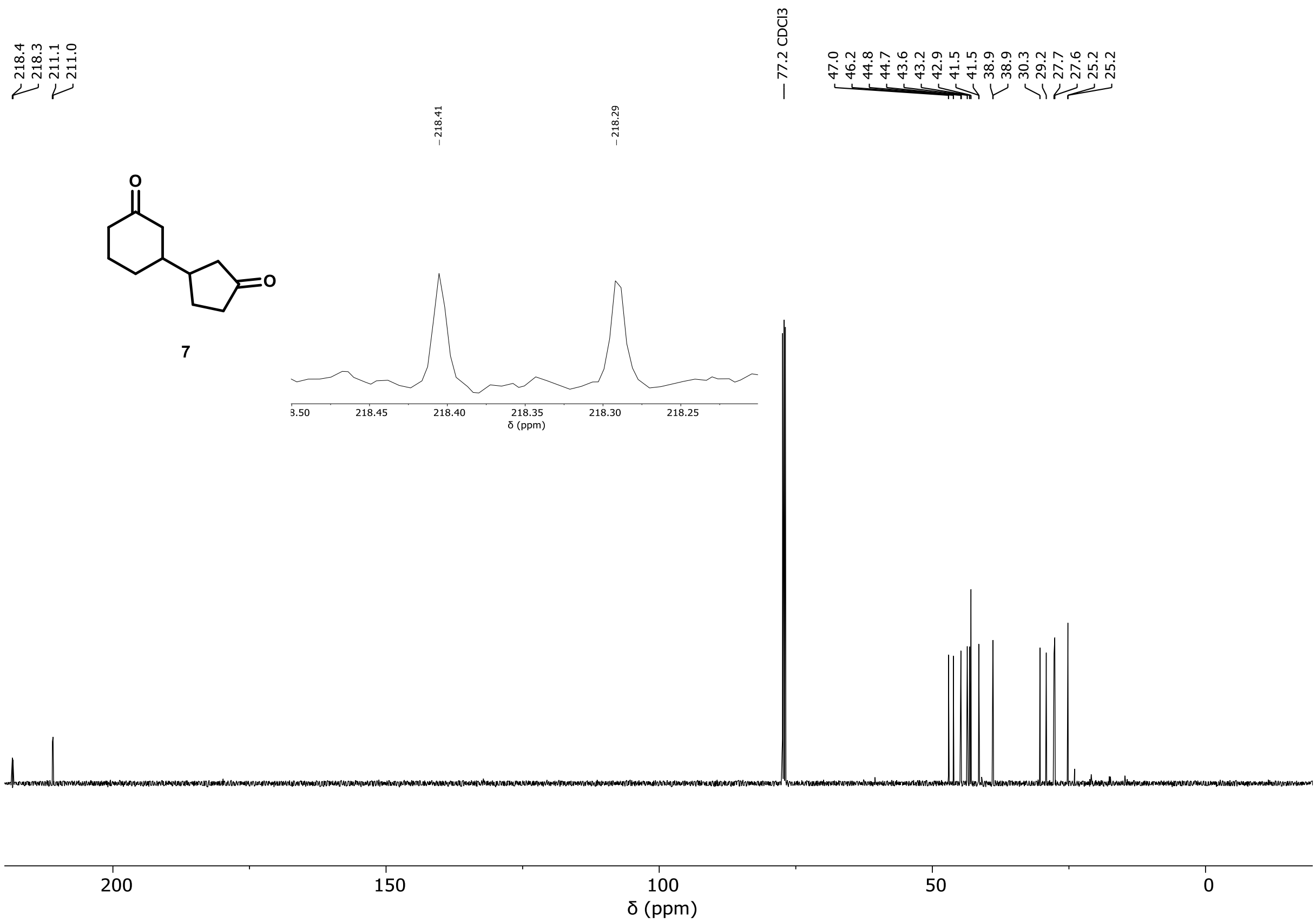
218.4
218.3
211.1
211.0
77.2 CDCl3
47.0 46.2 44.8 44.7 43.6 43.2 42.9 41.5 41.5 38.9 38.9 30.3 29.2 27.7 27.6 25.2 25.2
218.41
218.29
O
O
7
3.50
218.45
218.40
218.35
218.30
218.25
δ (ppm)
200
150
100
50
0
δ (ppm)

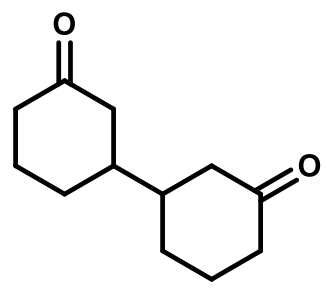

8

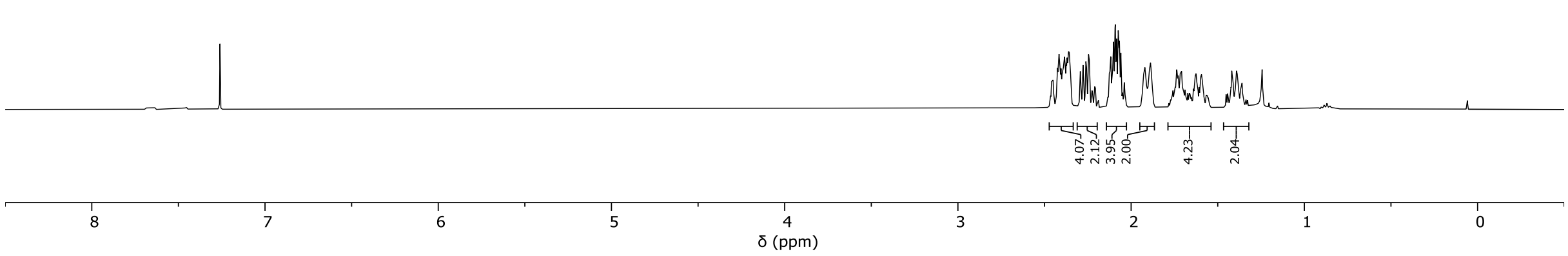
7.26 CDCl3
4.07
2.12
3.95
2.00
4.23
2.04
δ (ppm)

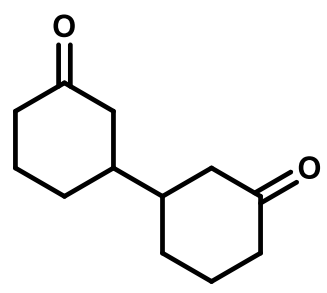

8

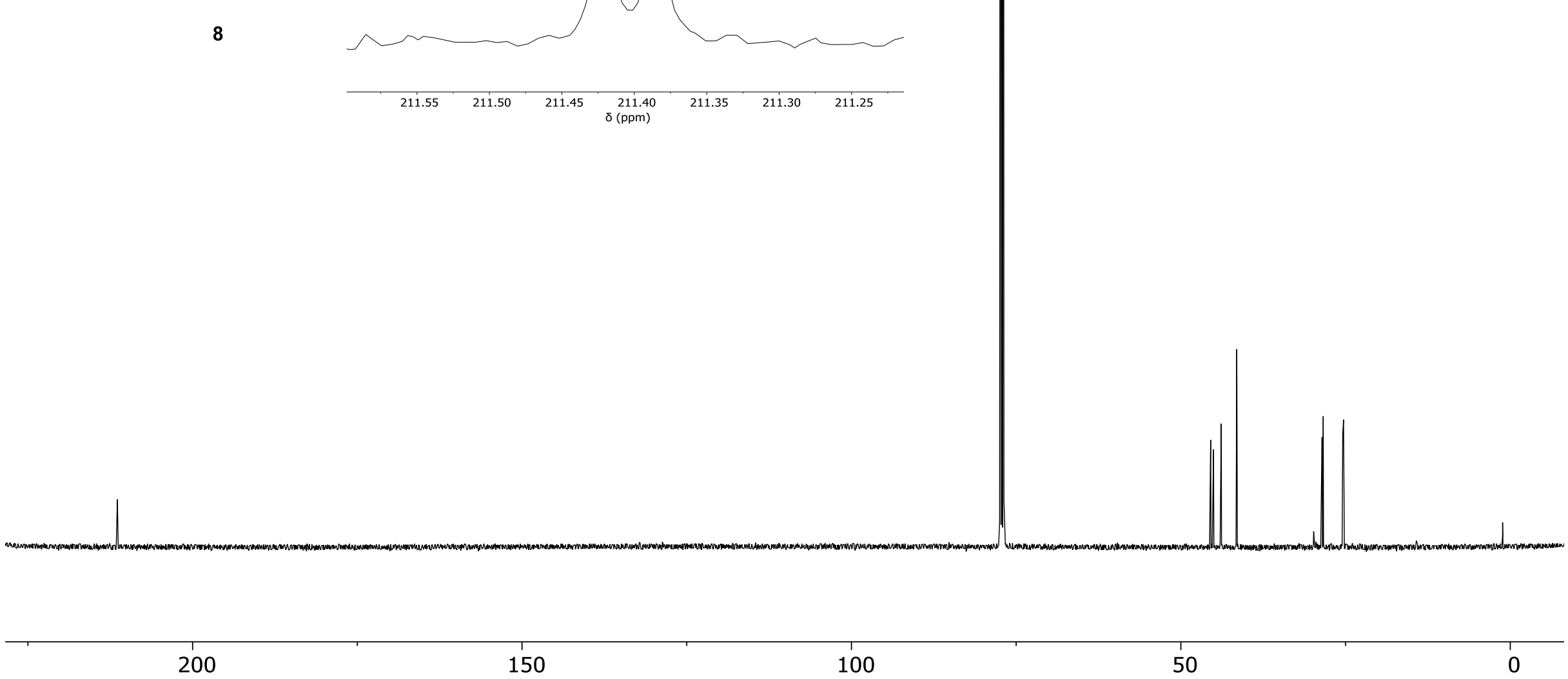
211.4
211.4
77.2 CDCl3
45.5
45.0
43.9
43.9
41.5
28.6
28.4
25.4
25.3
211.42
211.39
δ (ppm)

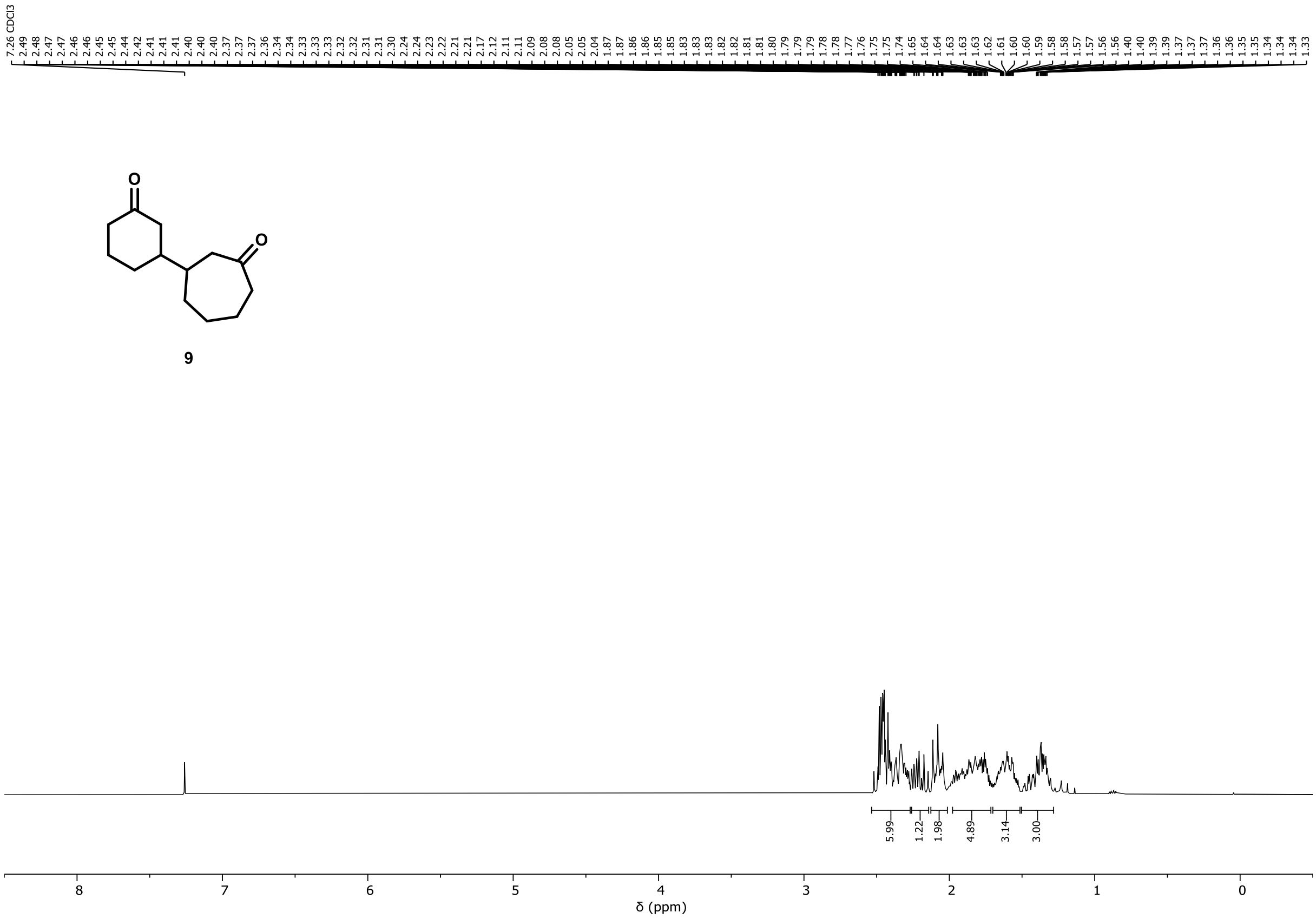
7.26 CDCl3
2.49 2.48 2.47 2.47 2.46 2.46 2.45 2.45 2.44 2.42 2.41 2.41 2.41 2.40 2.40 2.40 2.37 2.37 2.37 2.36 2.34 2.34 2.33 2.33 2.33 2.32 2.32 2.31 2.31 2.30 2.24 2.24 2.23 2.22 2.21 2.21 2.17 2.12 2.11 2.11 2.09 2.08 2.08 2.05 2.05 2.04 1.87 1.87 1.86 1.86 1.85 1.85 1.83 1.83 1.83 1.82 1.82 1.81 1.81 1.80 1.79 1.79 1.79 1.78 1.78 1.77 1.76 1.75 1.75 1.74 1.65 1.64 1.64 1.63 1.63 1.63 1.62 1.61 1.61 1.60 1.60 1.59 1.58 1.58 1.57 1.57 1.56 1.56 1.40 1.40 1.39 1.39 1.37 1.37 1.37 1.36 1.36 1.35 1.35 1.34 1.34 1.34 1.33
O
O
9
5.99
1.22
1.98
4.89
3.14
3.00
8
7
6
5
4
3
2
1
0
δ (ppm)

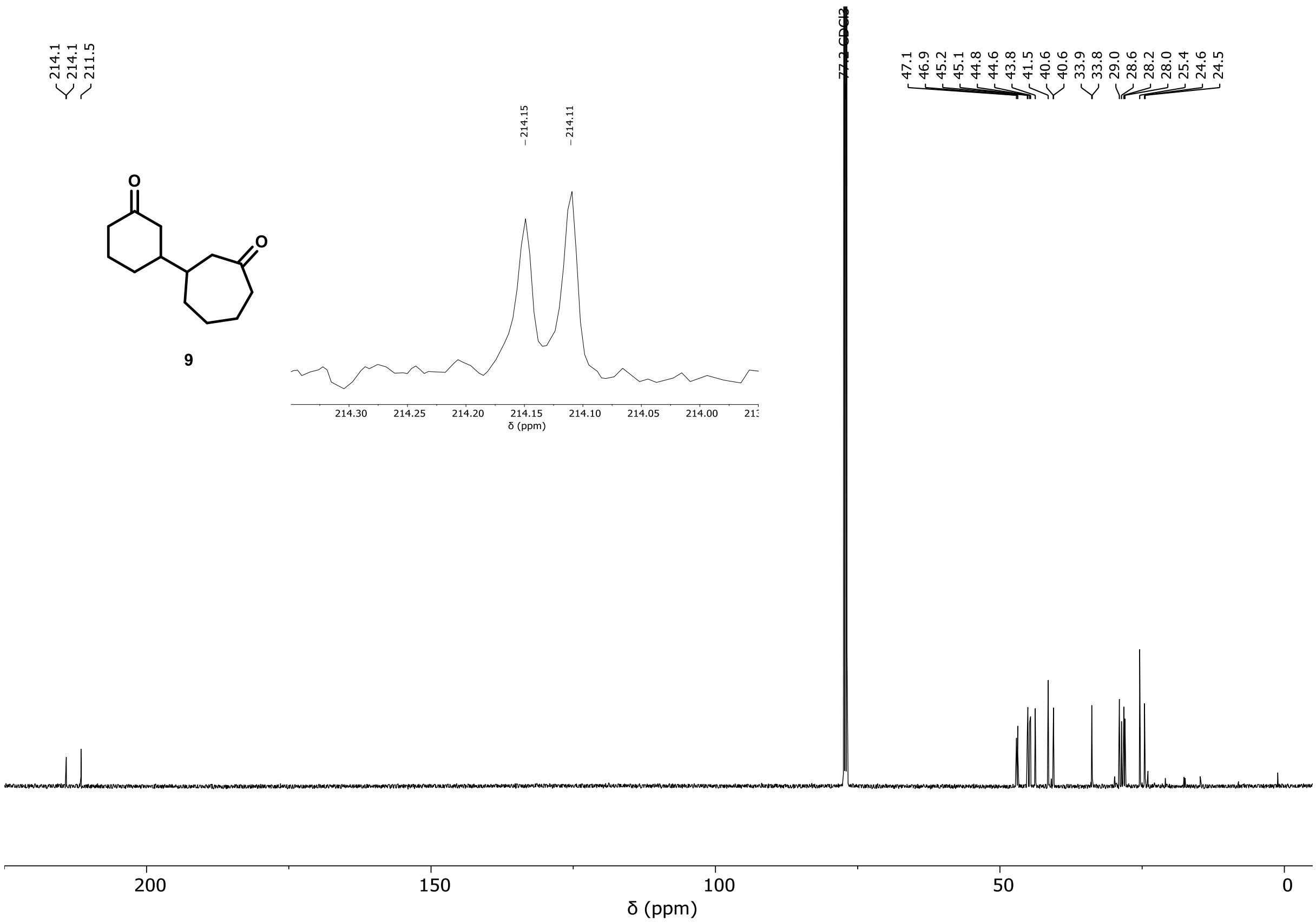
214.1
214.1
211.5
77.2 CDCl3
47.1 46.9 45.2 45.1 44.8 44.6 43.8 41.5 40.6 40.6 33.9 33.8 29.0 28.6 28.2 28.0 25.4 24.6 24.5
O
O
9
214.15
214.11
214.30 214.25 214.20 214.15 214.10 214.05 214.00
δ (ppm)
200
150
100
50
0
δ (ppm)

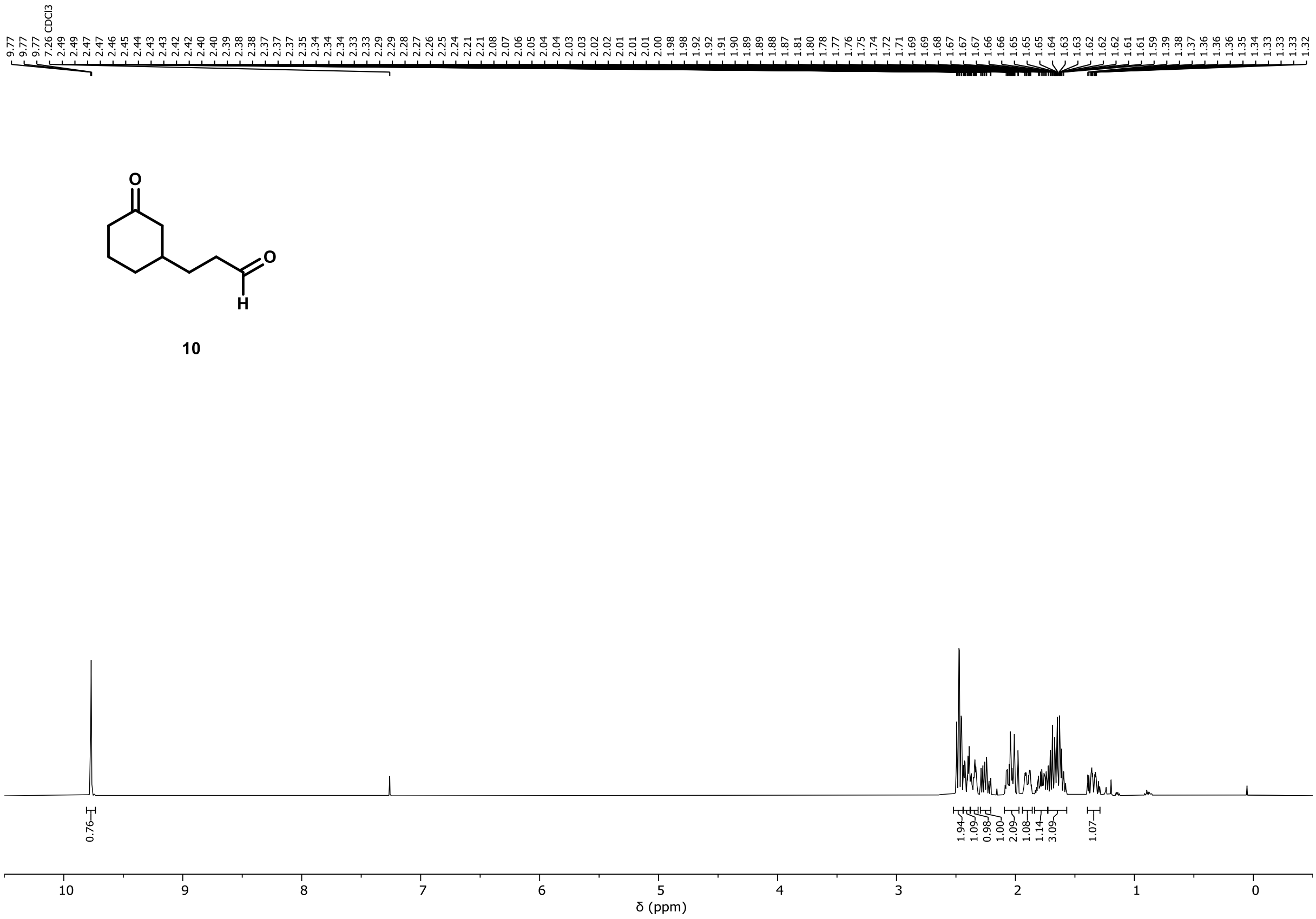
9.77 9.77 9.77 7.26 CDCl3 2.49 2.49 2.47 2.47 2.46 2.45 2.44 2.43 2.43 2.42 2.42 2.40 2.39 2.38 2.38 2.37 2.37 2.35 2.34 2.34 2.34 2.33 2.33 2.29 2.29 2.28 2.27 2.26 2.25 2.24 2.21 2.21 2.08 2.07 2.06 2.05 2.04 2.04 2.03 2.03 2.02 2.02 2.01 2.01 2.01 2.00 1.98 1.98 1.92 1.92 1.91 1.90 1.89 1.88 1.87 1.81 1.80 1.78 1.77 1.76 1.75 1.74 1.72 1.71 1.69 1.69 1.68 1.67 1.67 1.67 1.66 1.66 1.65 1.65 1.65 1.64 1.63 1.63 1.62 1.62 1.62 1.61 1.61 1.59 1.39 1.38 1.37 1.36 1.36 1.36 1.35 1.34 1.33 1.33 1.33 1.32
O
O
H
0.76
1.94
1.09
0.98
1.00
2.09
1.08
1.14
3.09
1.07
10 9 8 7 6 5 4 3 2 1 0
δ (ppm)

10

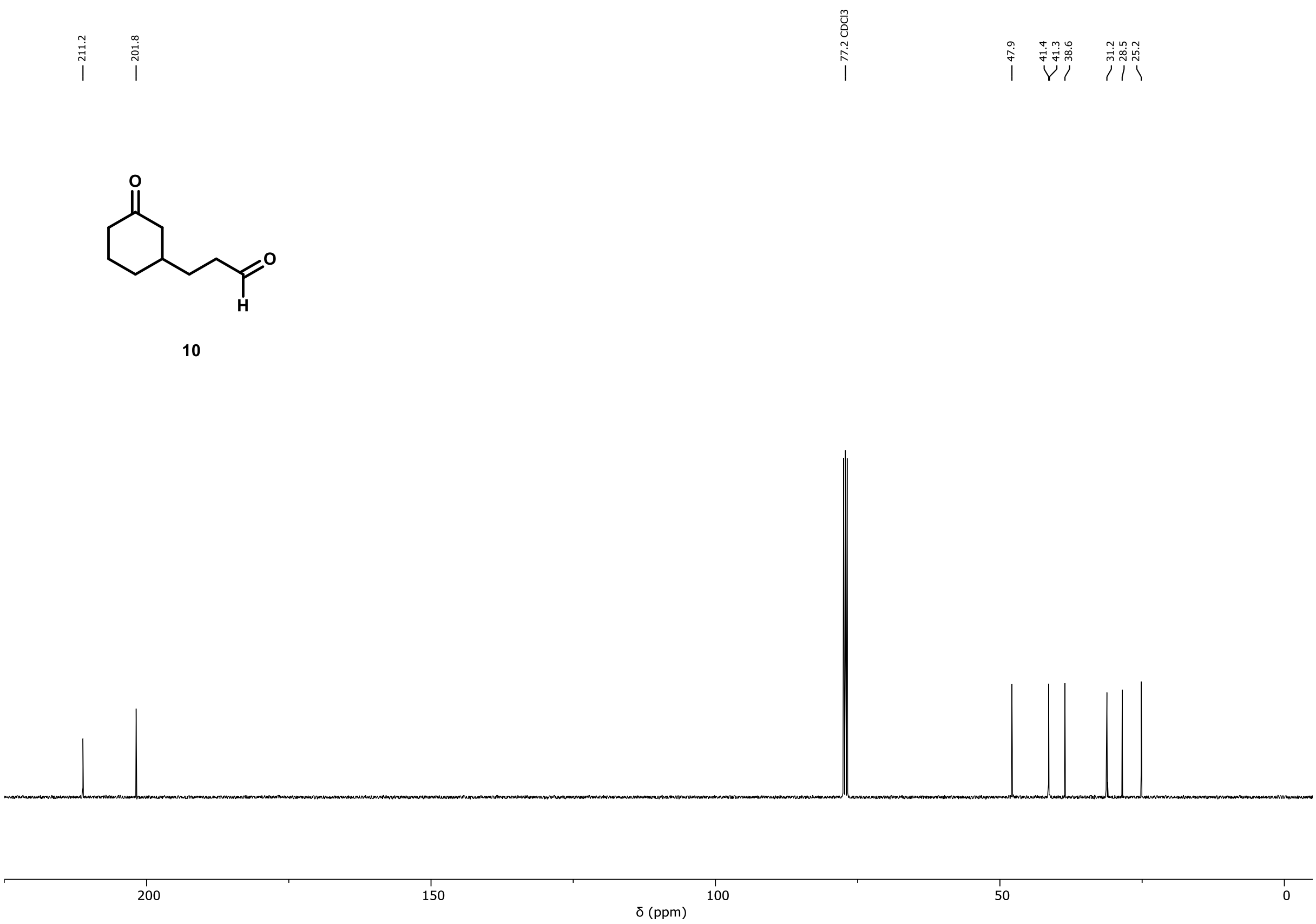
211.2
201.8
77.2 CDCl3
47.9
41.4
41.3
38.6
31.2
28.5
25.2
O
O
H
200 150 100 50 0
δ (ppm)

10

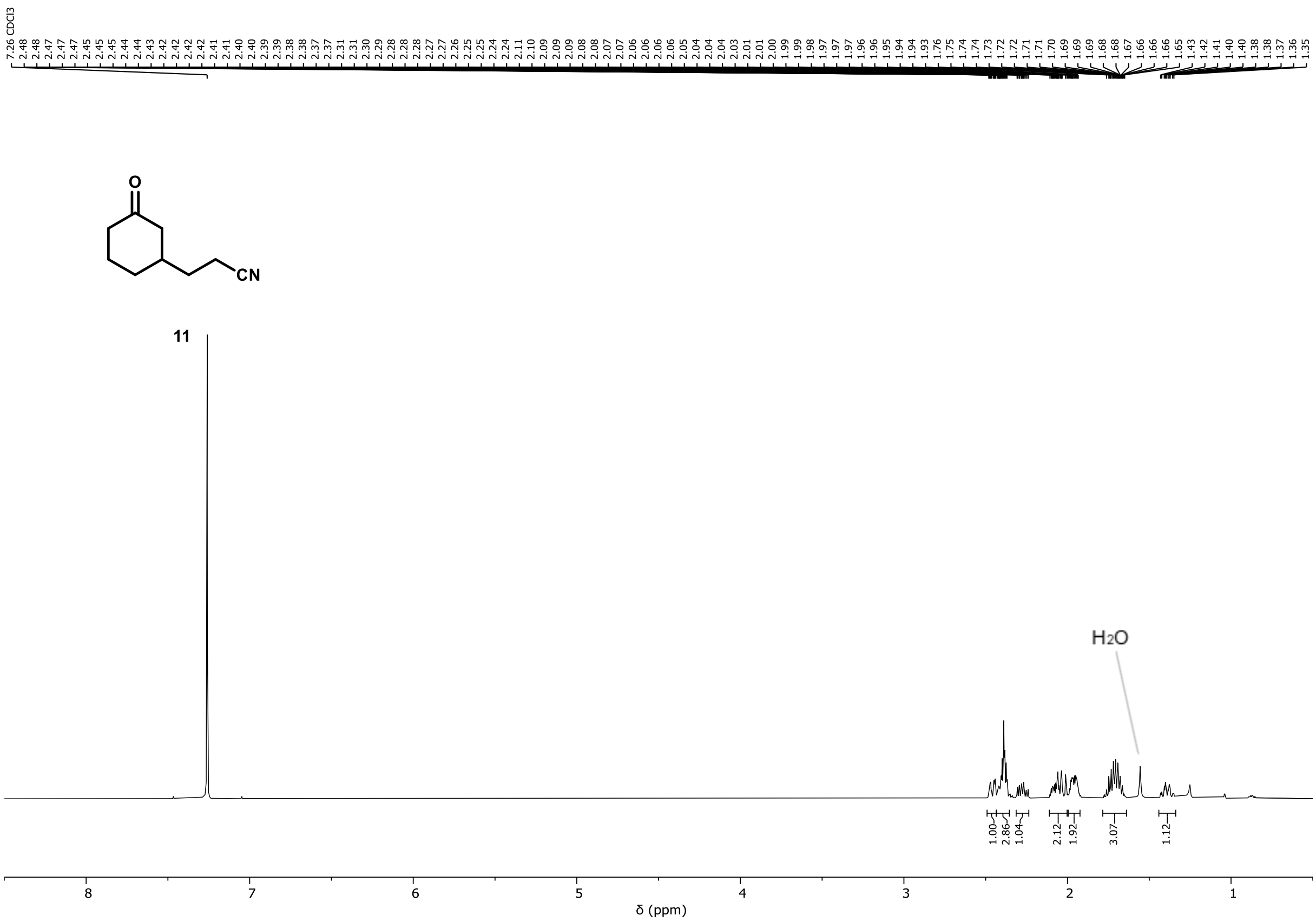
7.26 CDCl3
O
CN
11
H2O
1.00
2.86
1.04
2.12
1.92
3.07
1.12
8
7
6
5
4
3
2
1
δ (ppm)

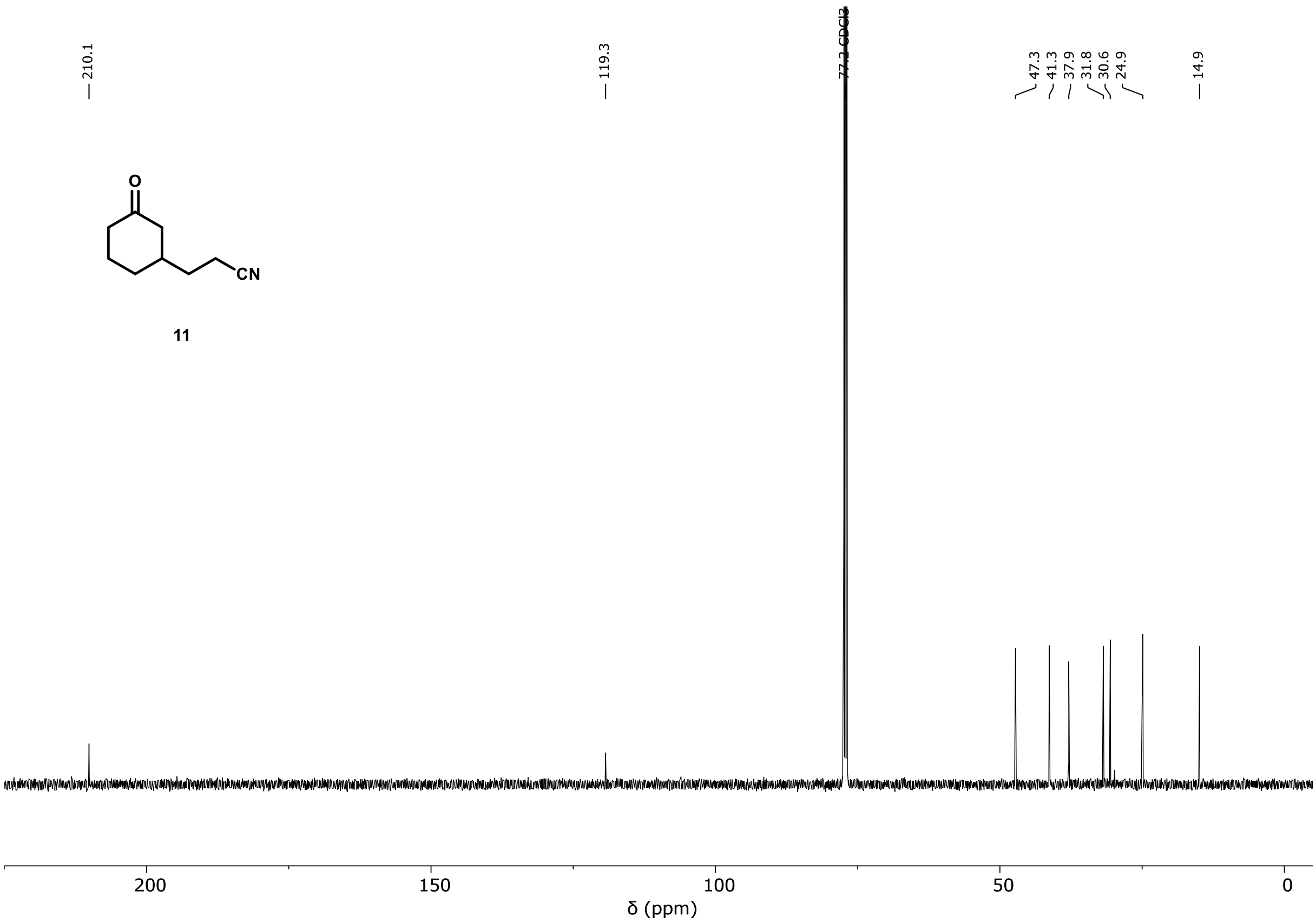
210.1
119.3
77.2 CDCl3
47.3
41.3
37.9
31.8
30.6
24.9
14.9
O
CN
11
200
150
100
50
0
δ (ppm)

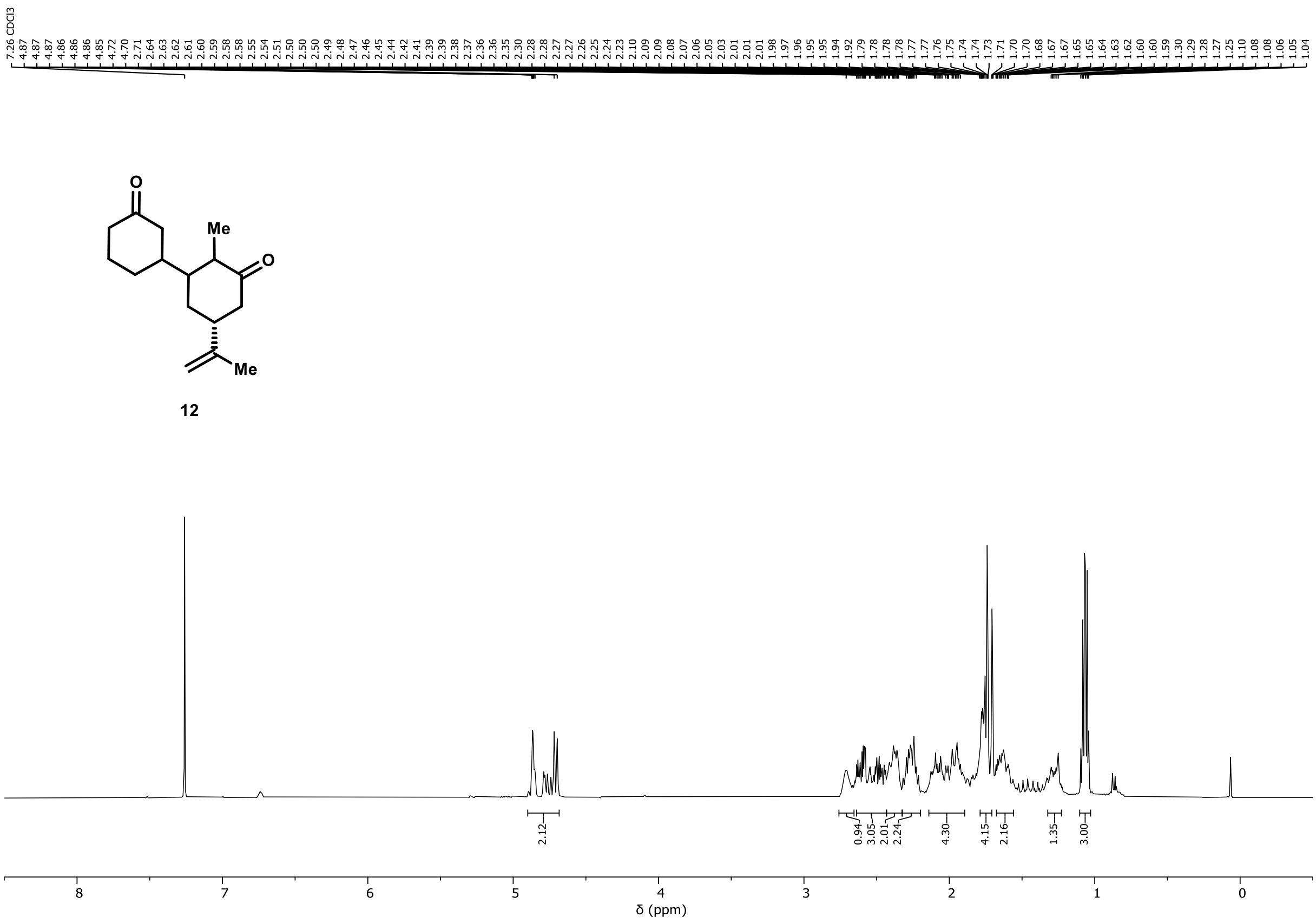
12
δ (ppm)

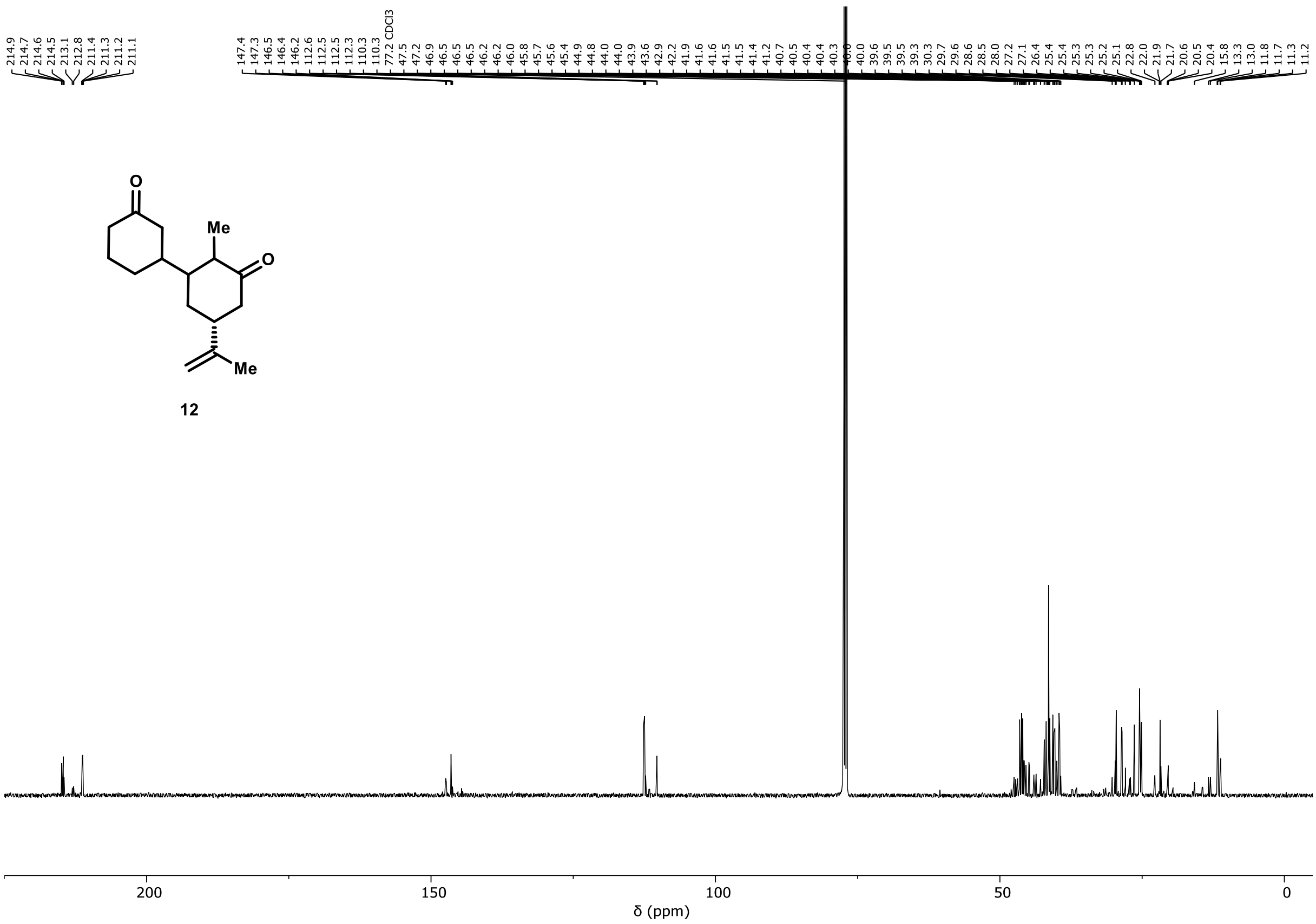
12
δ (ppm)

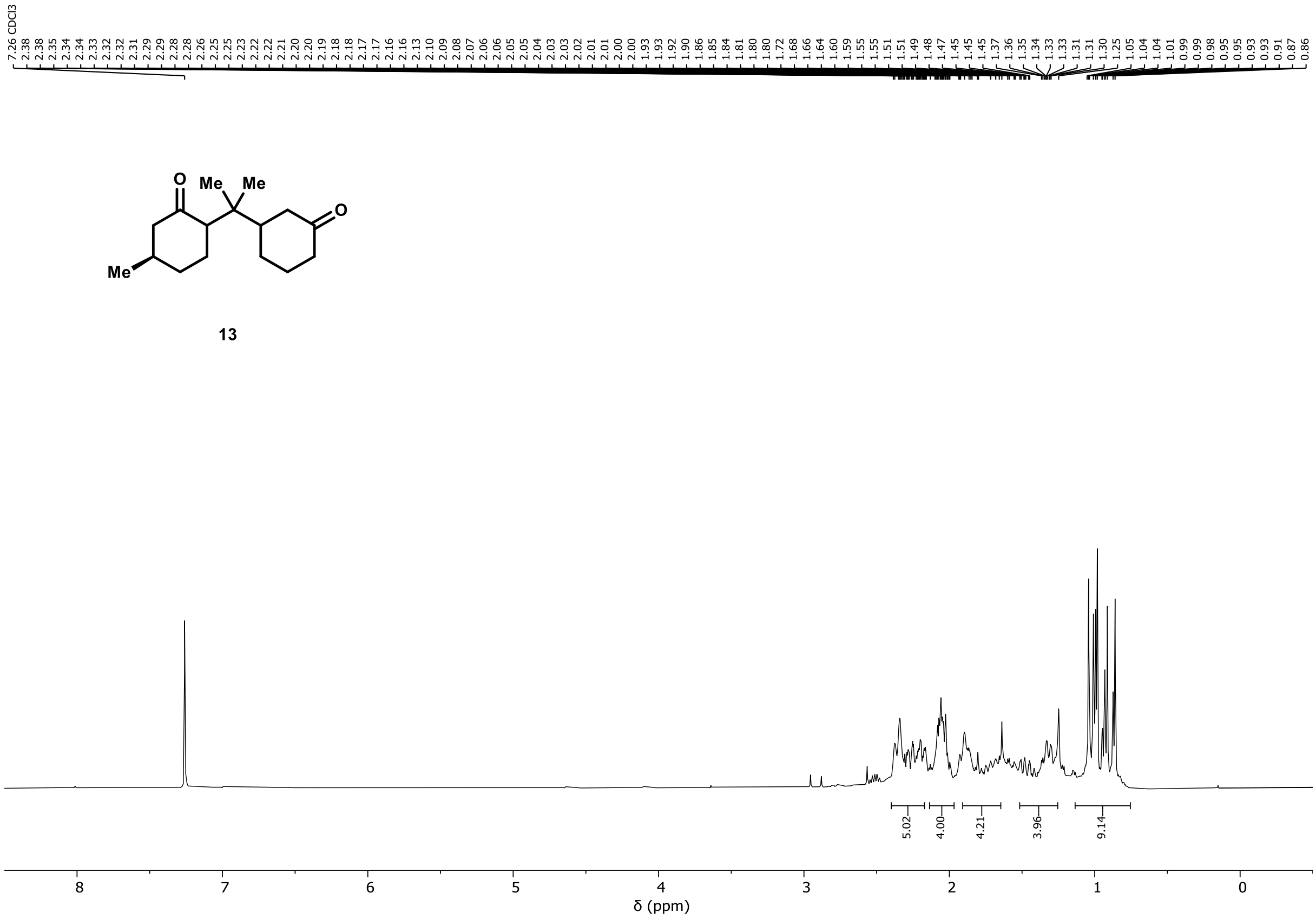
7.26 CDCl3
2.38 2.38 2.35 2.34 2.34 2.33 2.32 2.32 2.31 2.29 2.29 2.28 2.28 2.26 2.25 2.25 2.23 2.22 2.22 2.21 2.20 2.20 2.19 2.18 2.18 2.17 2.17 2.16 2.16 2.13 2.10 2.09 2.08 2.07 2.06 2.06 2.05 2.05 2.04 2.03 2.03 2.02 2.01 2.01 2.00 2.00 1.93 1.93 1.92 1.90 1.86 1.85 1.84 1.81 1.80 1.80 1.72 1.68 1.66 1.64 1.60 1.59 1.55 1.55 1.51 1.51 1.49 1.48 1.47 1.45 1.45 1.37 1.36 1.35 1.34 1.33 1.33 1.31 1.31 1.30 1.25 1.05 1.04 1.04 1.01 0.99 0.99 0.98 0.95 0.95 0.93 0.93 0.91 0.87 0.86
O
Me
Me
O
Me
13
5.02
4.00
4.21
3.96
9.14
8
7
6
5
4
3
2
1
0
δ (ppm)

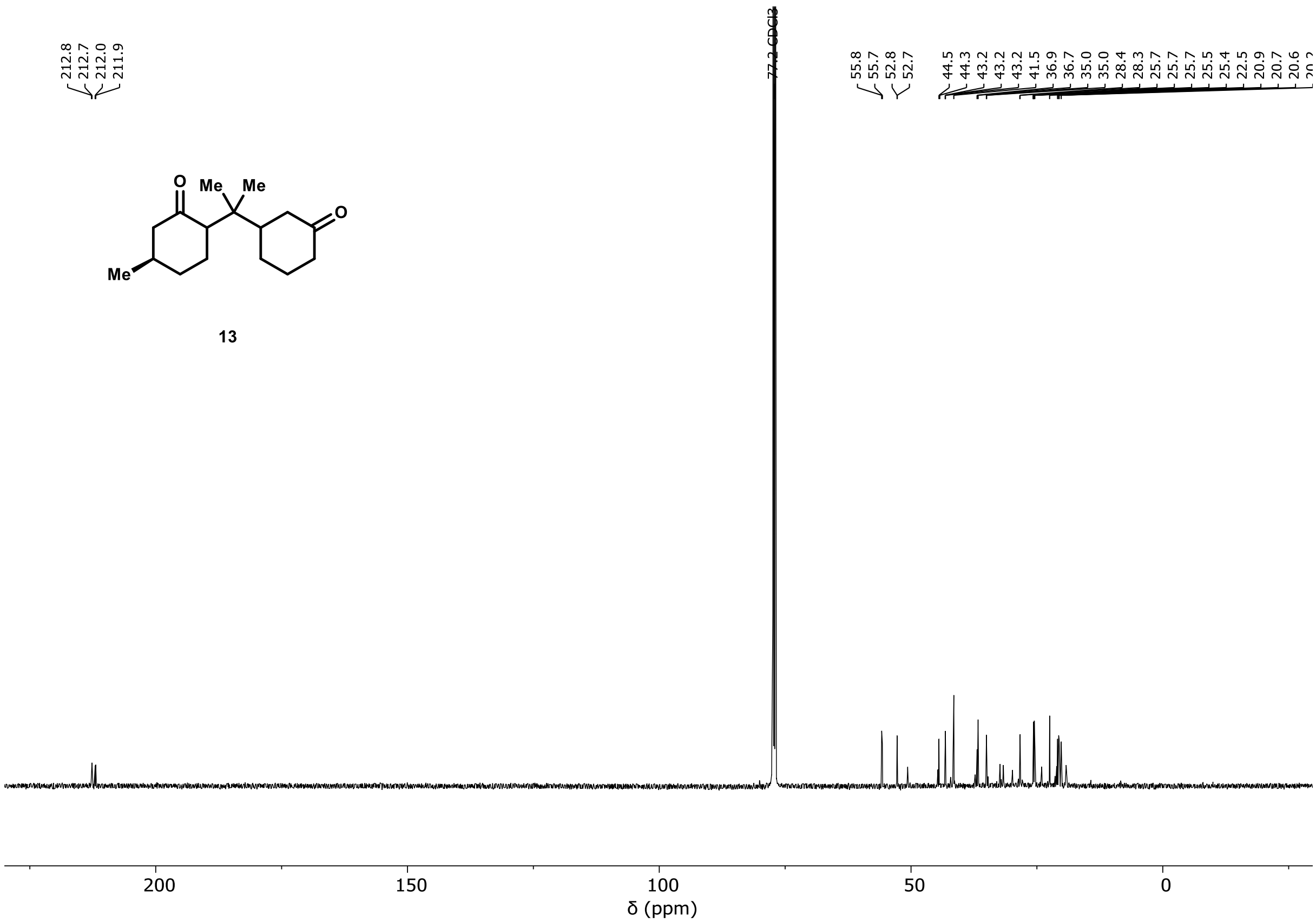
212.8
212.7
212.0
211.9
77.2 CDCl3
55.8
55.7
52.8
52.7
44.5
44.3
43.2
43.2
43.2
41.5
36.9
36.7
35.0
35.0
28.4
28.3
25.7
25.7
25.7
25.5
25.4
22.5
20.9
20.7
20.6
O
Me
Me
O
Me
13
200
150
100
50
0
δ (ppm)

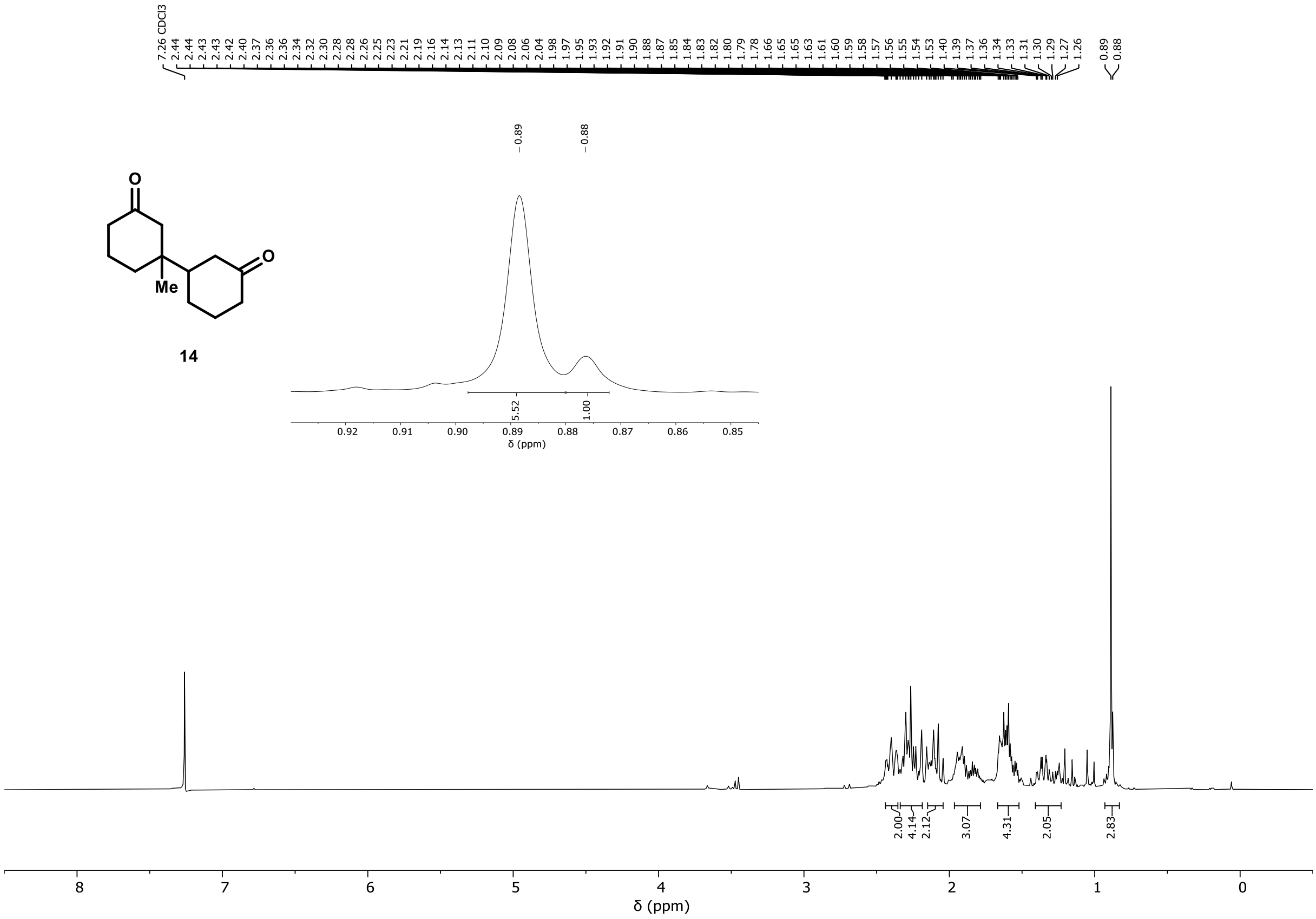

14

7.26 CDCl3
2.44
2.44
2.43
2.43
2.42
2.40
2.37
2.36
2.36
2.34
2.32
2.30
2.28
2.28
2.26
2.25
2.23
2.21
2.19
2.16
2.14
2.13
2.11
2.10
2.09
2.08
2.06
2.04
1.98
1.97
1.95
1.93
1.92
1.91
1.90
1.88
1.87
1.85
1.84
1.83
1.82
1.80
1.79
1.78
1.66
1.65
1.65
1.63
1.61
1.60
1.59
1.58
1.57
1.56
1.55
1.54
1.53
1.40
1.39
1.37
1.36
1.34
1.33
1.31
1.30
1.29
1.27
1.26
0.89
0.88
0.89
0.88
5.52
1.00
0.92
0.91
0.90
0.89
0.88
0.87
0.86
0.85
δ (ppm)
2.00
4.14
2.12
3.07
4.31
2.05
2.83
8
7
6
5
4
3
2
1
0
δ (ppm)

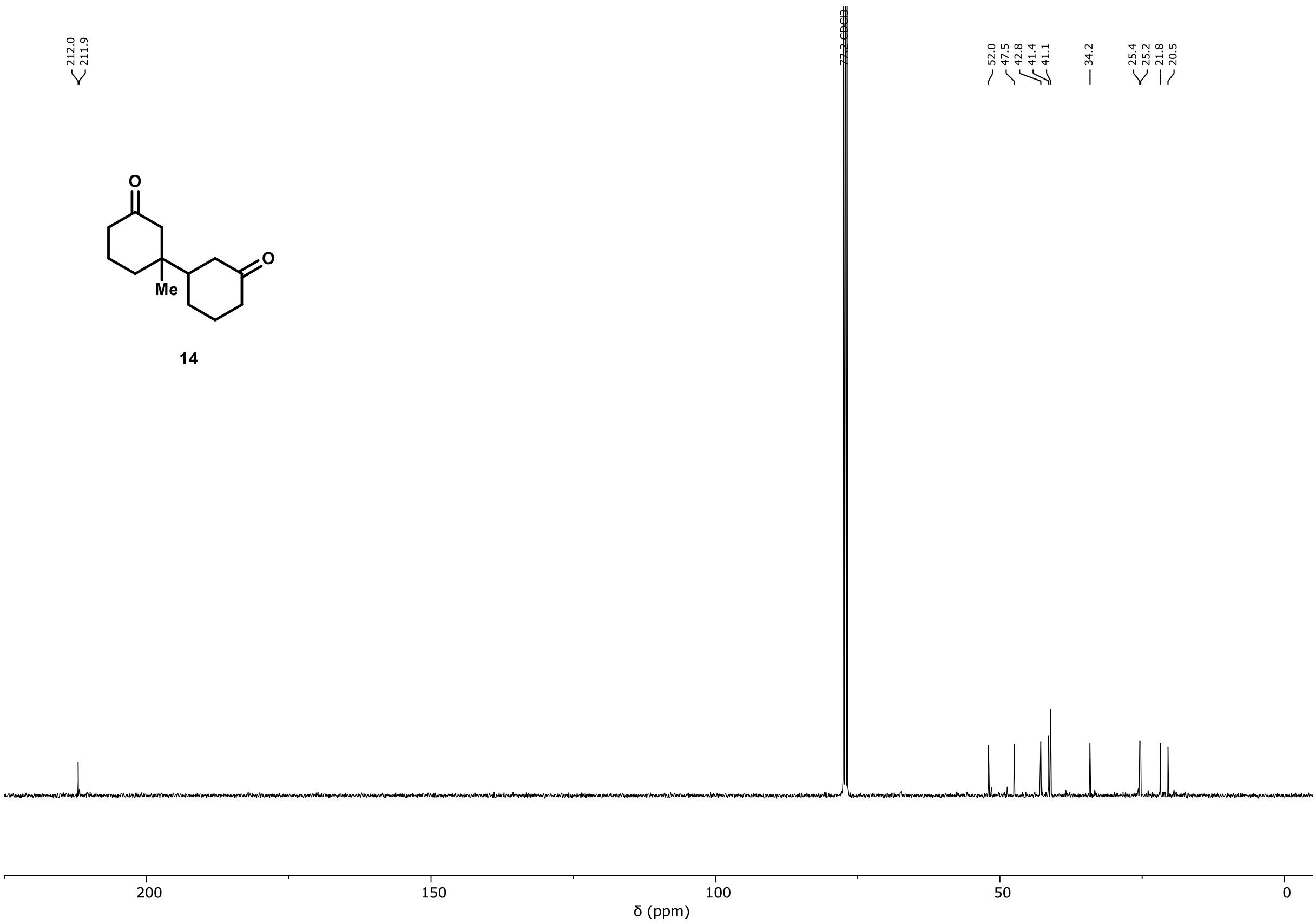

14

212.0
211.9
77.2 CDCl3
52.0
47.5
42.8
41.4
41.1
34.2
25.4
25.2
21.8
20.5
200
150
100
50
0
δ (ppm)

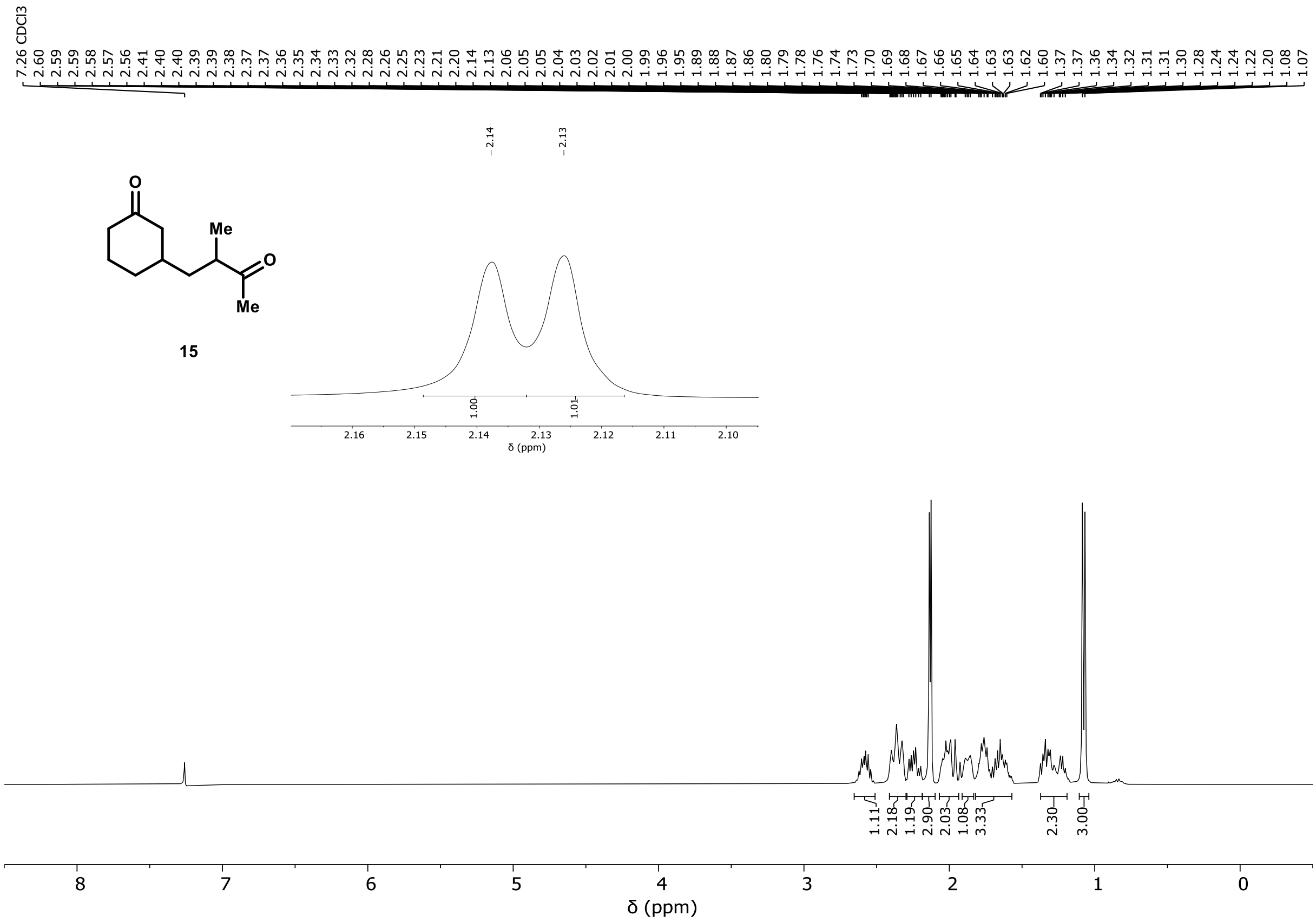

7.26 CDCl3
2.60 2.59 2.59 2.58 2.57 2.56 2.41 2.40 2.40 2.39 2.39 2.38 2.37 2.37 2.36 2.35 2.34 2.33 2.32 2.28 2.26 2.25 2.23 2.21 2.20 2.14 2.13 2.06 2.05 2.05 2.04 2.03 2.02 2.01 2.00 1.99 1.96 1.95 1.89 1.88 1.87 1.86 1.80 1.79 1.78 1.76 1.74 1.73 1.70 1.69 1.68 1.67 1.66 1.65 1.64 1.63 1.63 1.62 1.60 1.37 1.37 1.36 1.34 1.32 1.31 1.31 1.30 1.28 1.24 1.22 1.20 1.08 1.07
2.14
2.13
1.00
1.01
δ (ppm)
1.11 2.18 1.19 2.90 2.03 1.08 3.33 2.30 3.00
δ (ppm)

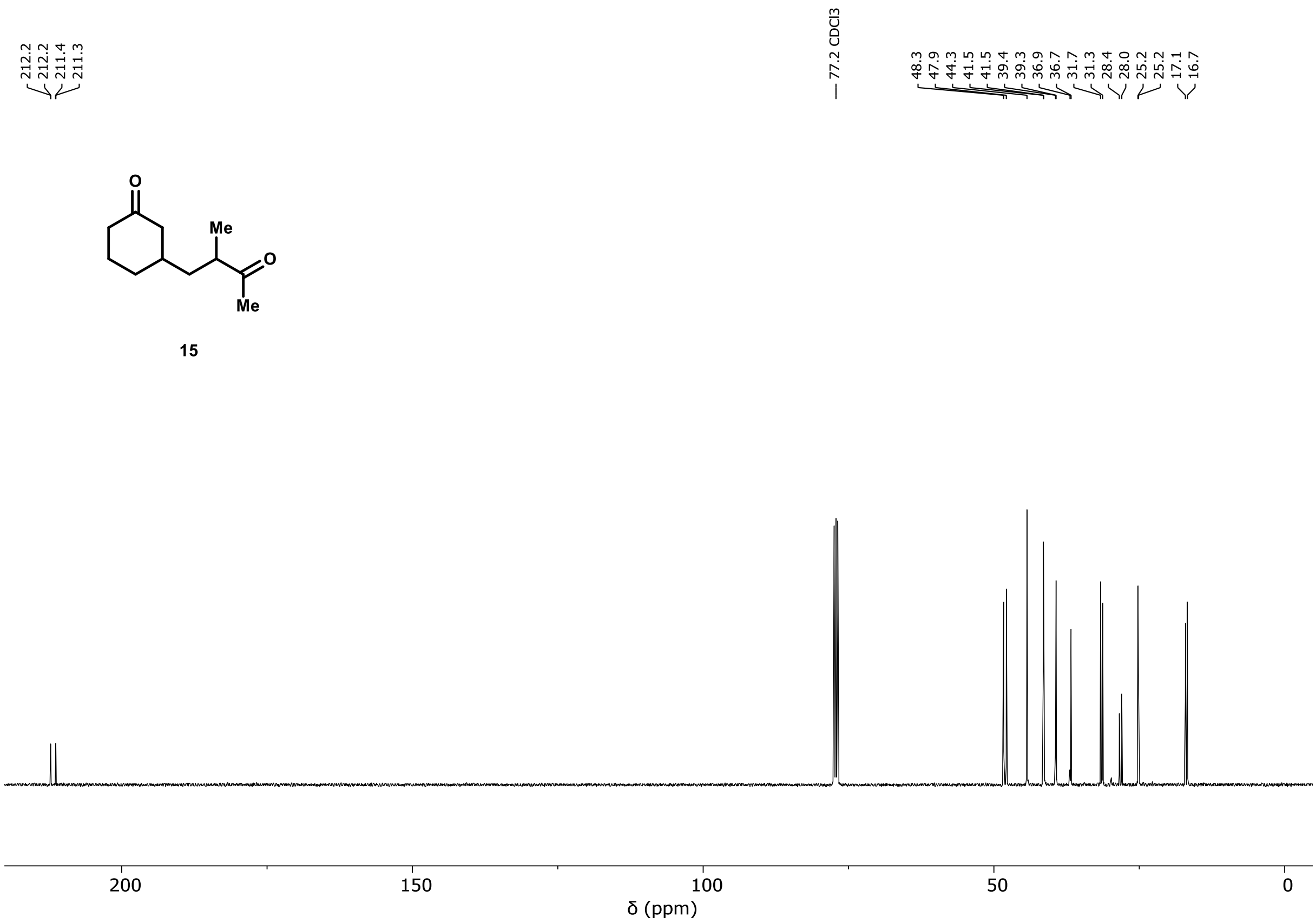

212.2 212.2 211.4 211.3
77.2 CDCl3
48.3 47.9 44.3 41.5 41.5 39.4 39.3 36.9 36.7 31.7 31.3 28.4 28.0 25.2 25.2 17.1 16.7
δ (ppm)

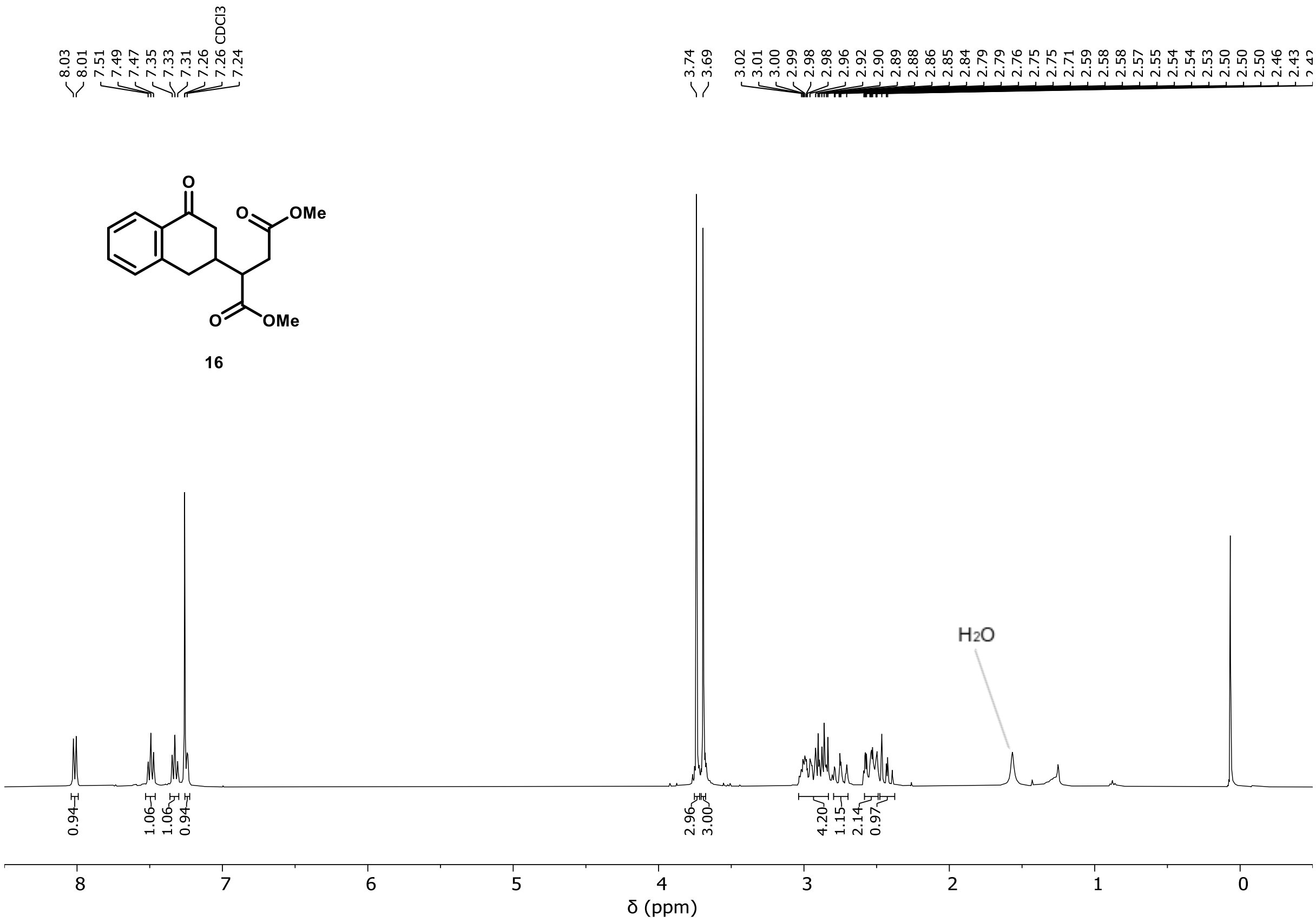

8.03
8.01
7.51
7.49
7.47
7.35
7.33
7.31
7.26
7.26 CDCl3
7.24
3.74
3.69
3.02
3.01
3.00
2.99
2.98
2.98
2.96
2.92
2.90
2.89
2.88
2.86
2.85
2.84
2.79
2.79
2.76
2.75
2.75
2.71
2.59
2.58
2.57
2.55
2.54
2.53
2.50
2.50
2.50
2.46
2.43
O
OMe
O
O
OMe
16
H2O
0.94
1.06
1.06
0.94
2.96
3.00
4.20
1.15
2.14
0.97
8
7
6
5
4
3
2
1
0
δ (ppm)

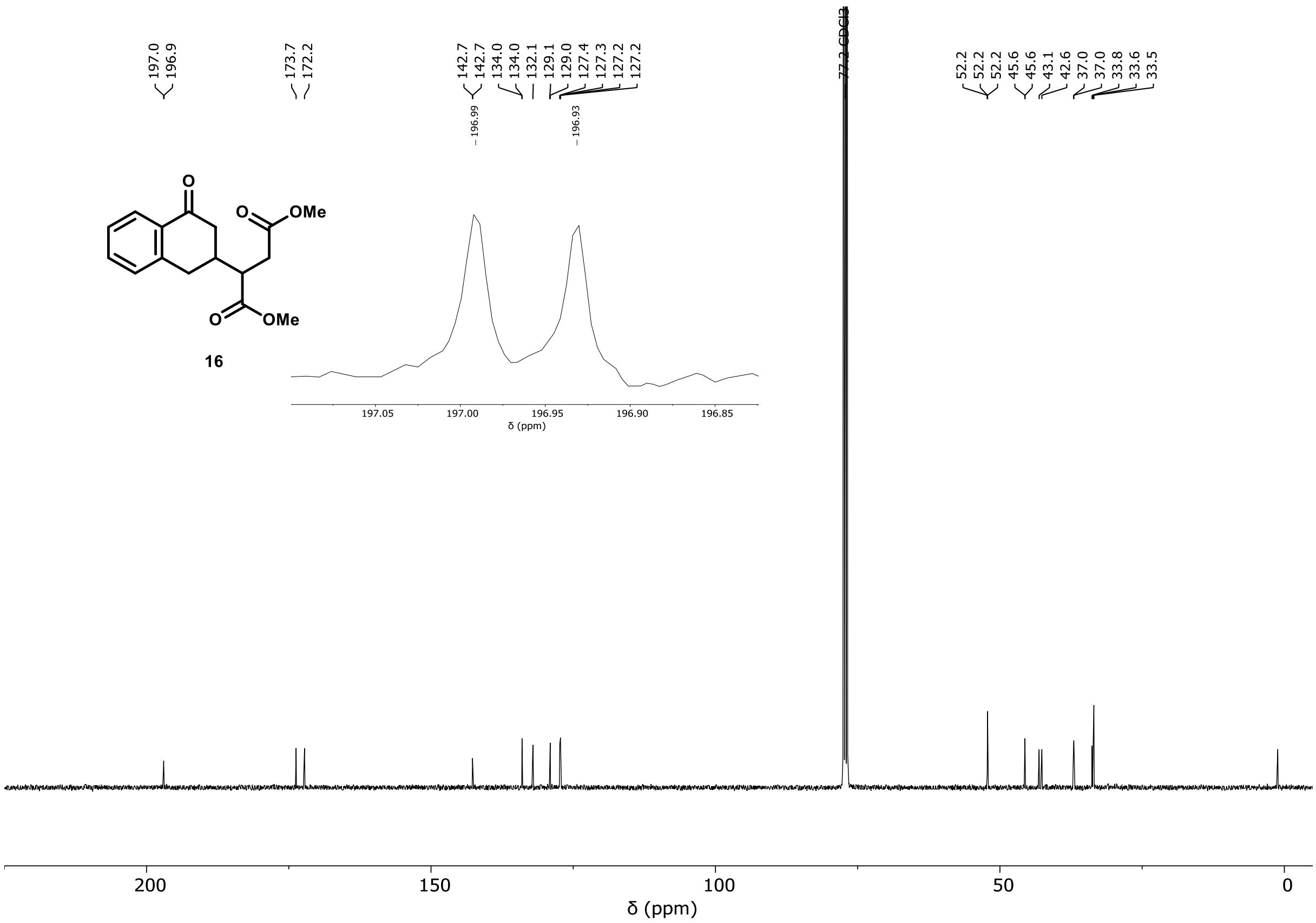

197.0
196.9
173.7
172.2
142.7
142.7
134.0
134.0
132.1
129.1
129.0
127.4
127.3
127.2
127.2
77.2 CDCl3
52.2
52.2
52.2
45.6
45.6
43.1
42.6
37.0
37.0
33.8
33.6
33.5
196.99
196.93
O
OMe
O
O
OMe
16
197.05
197.00
196.95
196.90
196.85
δ (ppm)
200
150
100
50
0
δ (ppm)

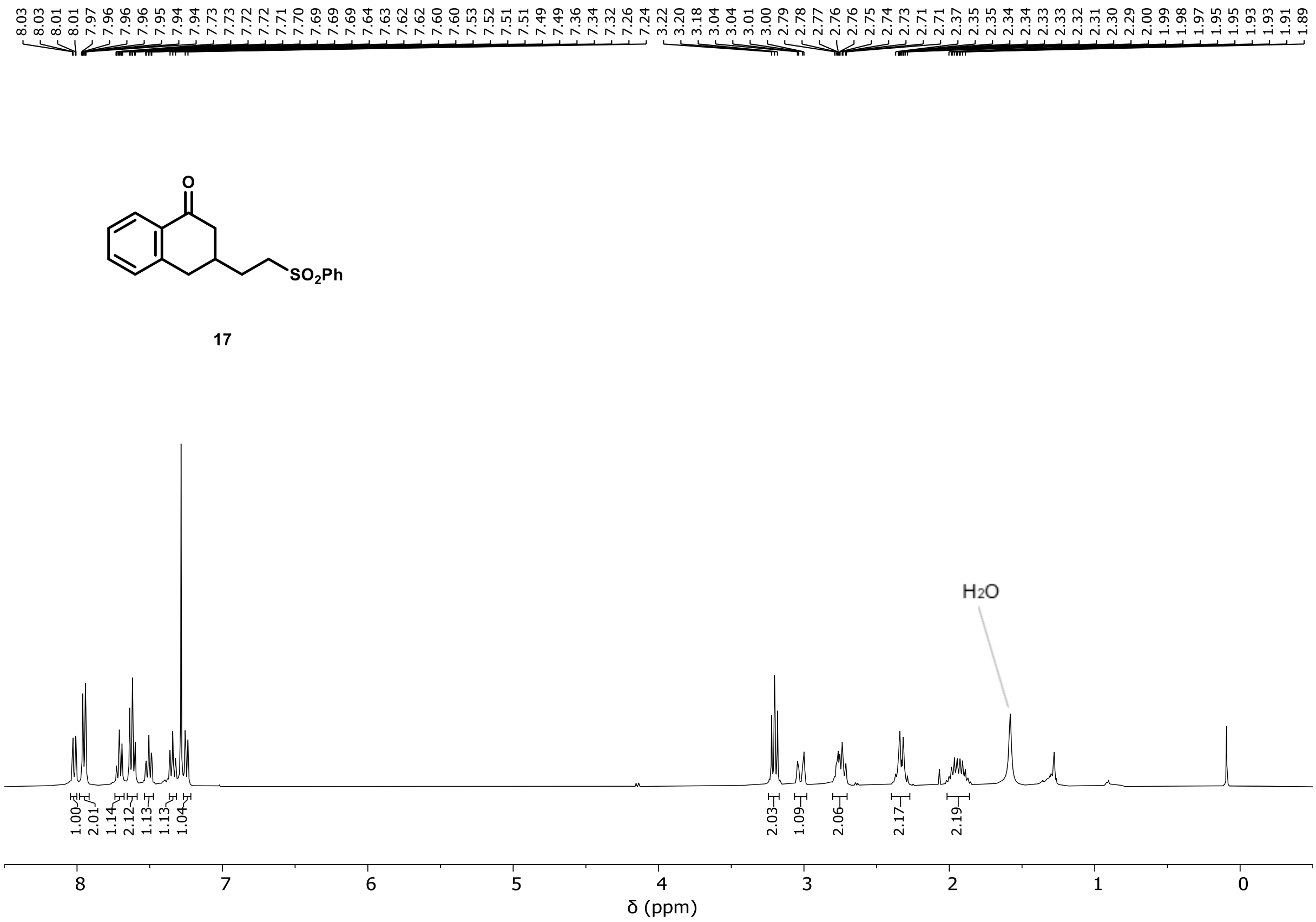
8.03 8.03 8.01 8.01 7.97 7.96 7.96 7.96 7.95 7.94 7.94 7.73 7.73 7.72 7.72 7.71 7.70 7.69 7.69 7.69 7.64 7.63 7.62 7.62 7.60 7.53 7.52 7.51 7.51 7.49 7.49 7.36 7.34 7.32 7.26 7.24 3.22 3.20 3.18 3.04 3.01 3.00 2.79 2.78 2.77 2.76 2.76 2.75 2.74 2.73 2.71 2.71 2.37 2.35 2.35 2.34 2.34 2.33 2.33 2.32 2.31 2.30 2.29 2.00 1.99 1.98 1.97 1.95 1.93 1.93 1.91 1.89
O
SO2Ph
17
H2O
1.00 2.01 1.14 2.12 1.13 1.13 1.04
2.03 1.09 2.06 2.17 2.19
8 7 6 5 4 3 2 1 0
δ (ppm)

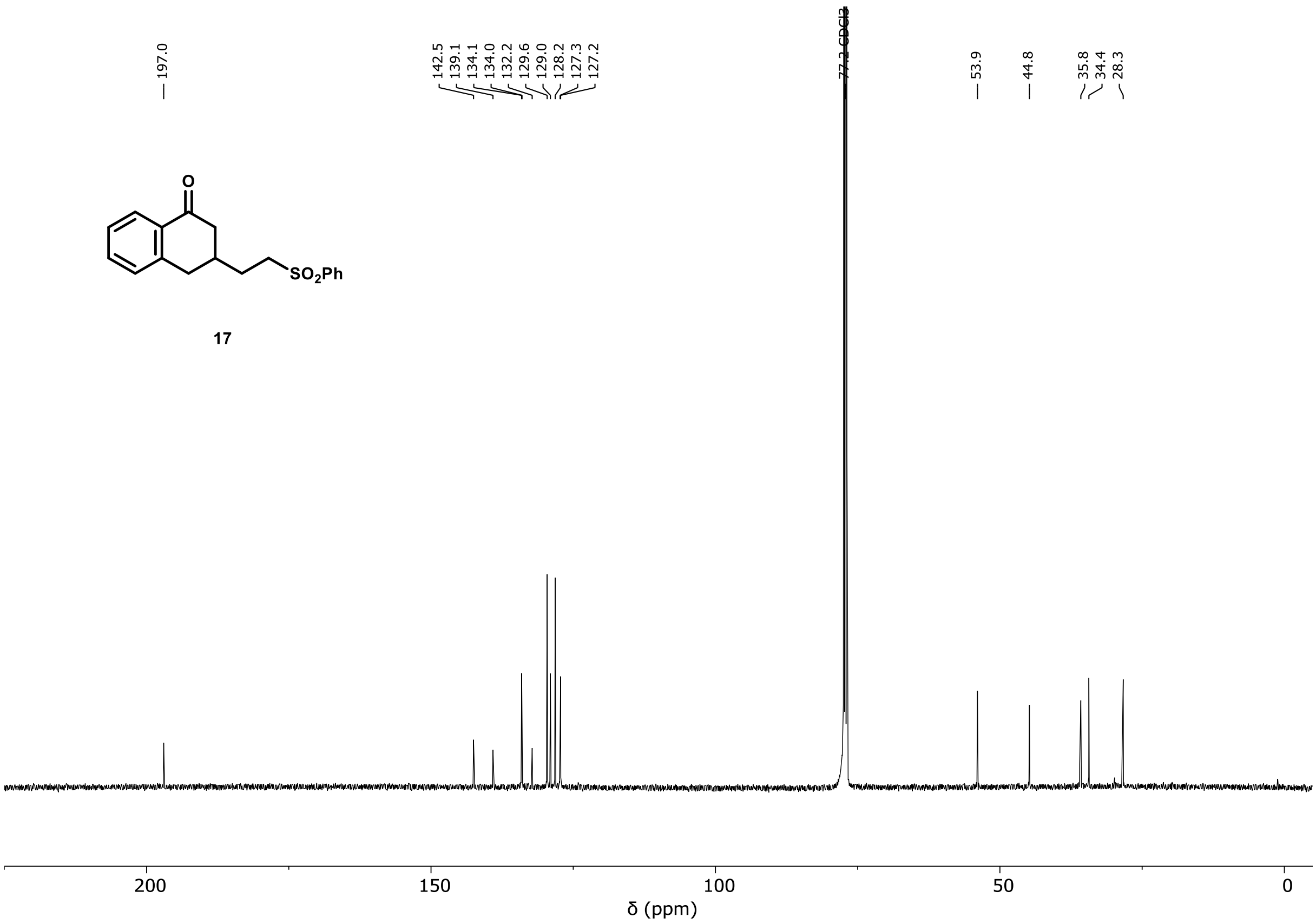
197.0
142.5 139.1 134.1 134.0 132.2 129.6 129.0 128.2 127.3 127.2
77.2 CDCl3
53.9
44.8
35.8 34.4 28.3
O
SO2Ph
17
200 150 100 50 0
δ (ppm)

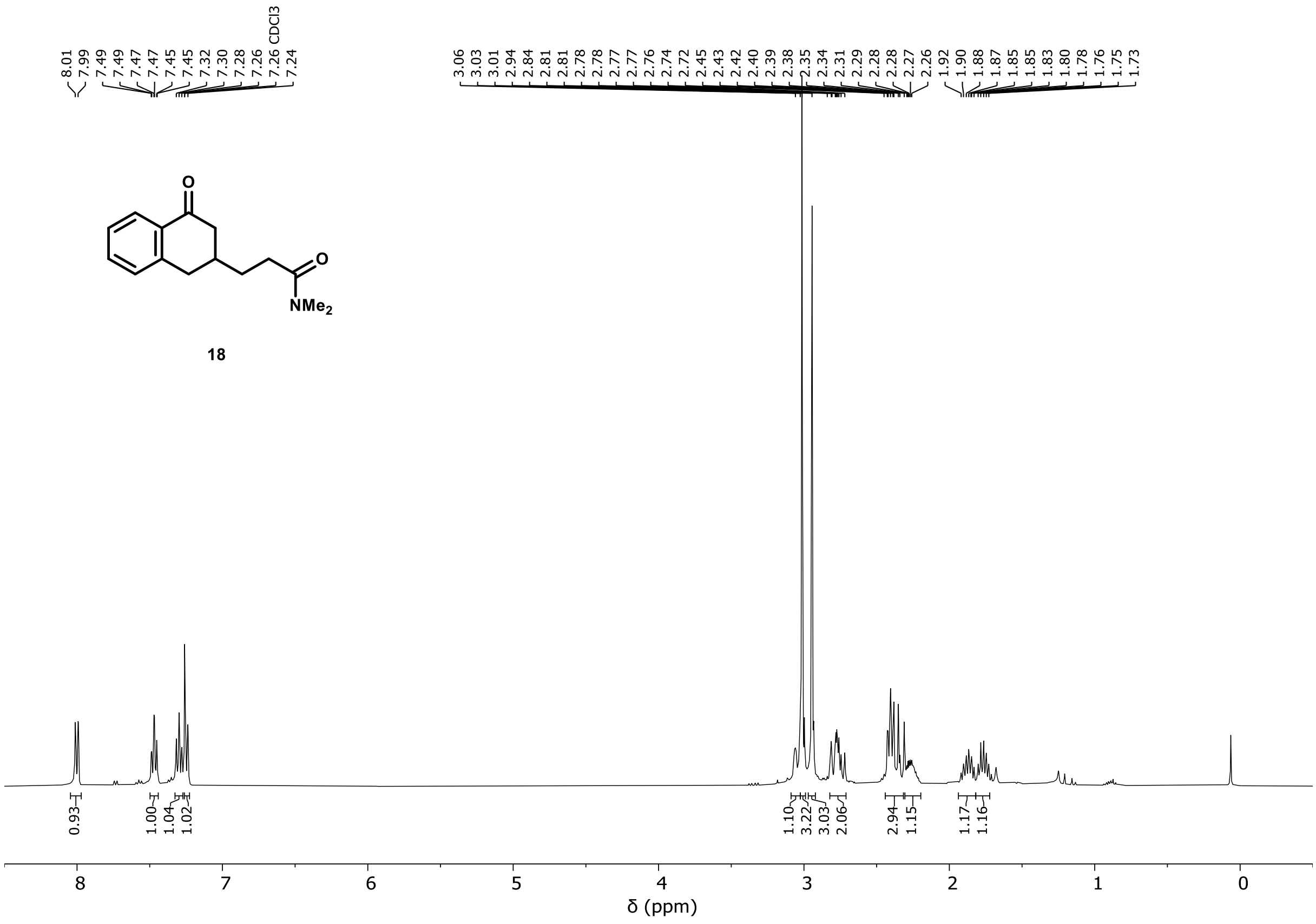
8.01
7.99
7.49
7.49
7.47
7.47
7.45
7.45
7.32
7.30
7.28
7.26
7.26 CDCl3
7.24
3.06
3.03
3.01
2.94
2.84
2.81
2.81
2.78
2.78
2.77
2.77
2.76
2.74
2.72
2.45
2.43
2.42
2.40
2.39
2.38
2.35
2.34
2.31
2.29
2.28
2.28
2.27
2.26
1.92
1.90
1.88
1.87
1.85
1.85
1.83
1.80
1.78
1.76
1.75
1.73
O
NMe2
18
0.93
1.00
1.04
1.02
1.10
3.22
3.03
2.06
2.94
1.15
1.17
1.16
8
7
6
5
4
3
2
1
0
δ (ppm)

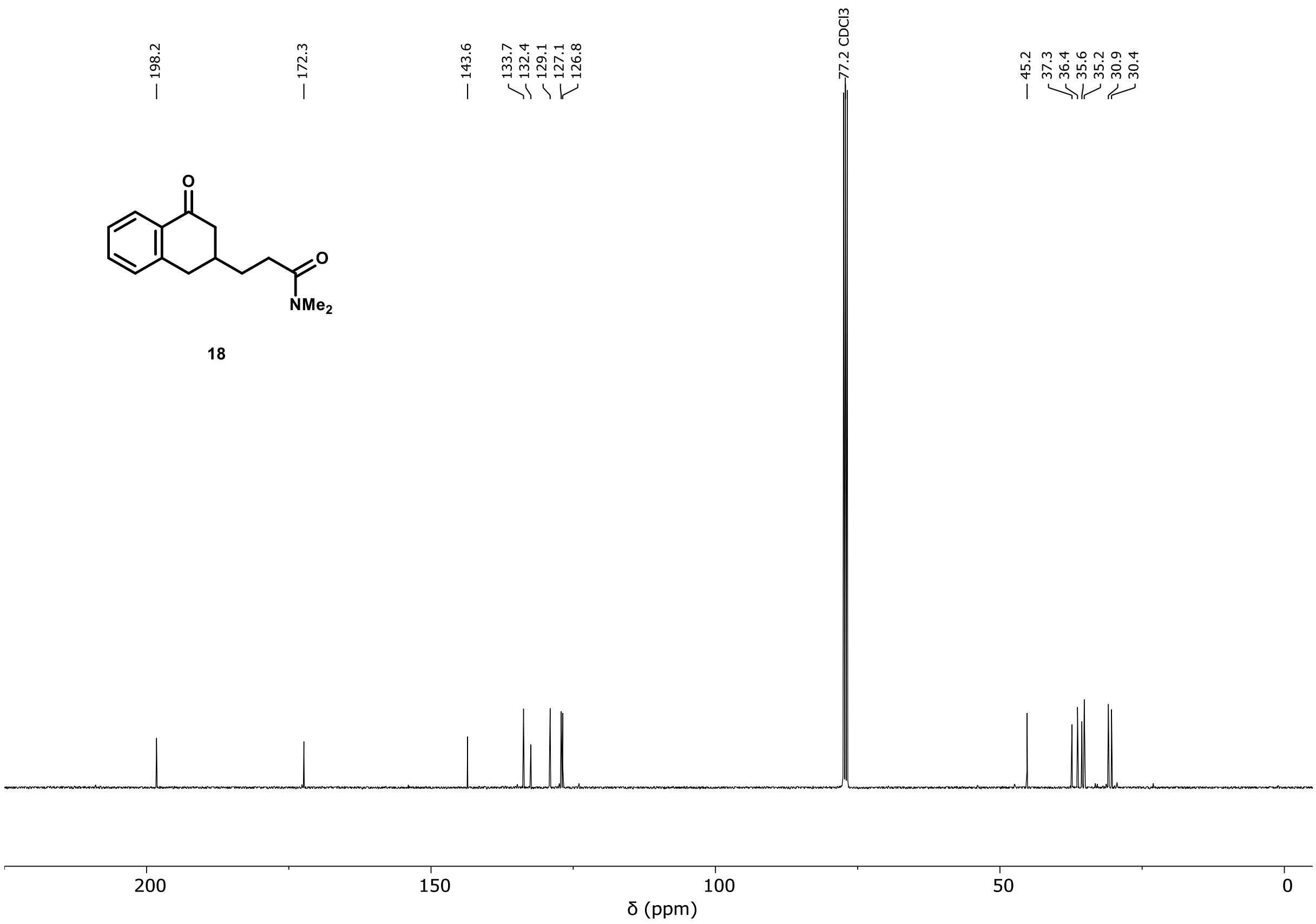
198.2
172.3
143.6
133.7
132.4
129.1
127.1
126.8
77.2 CDCl3
45.2
37.3
36.4
35.6
35.2
30.9
30.4
O
NMe2
18
200
150
100
50
0
δ (ppm)

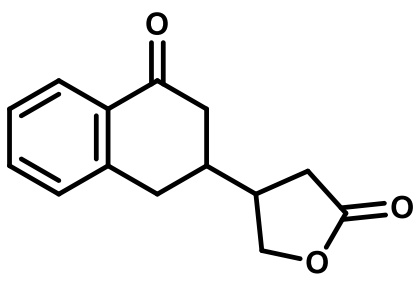

19

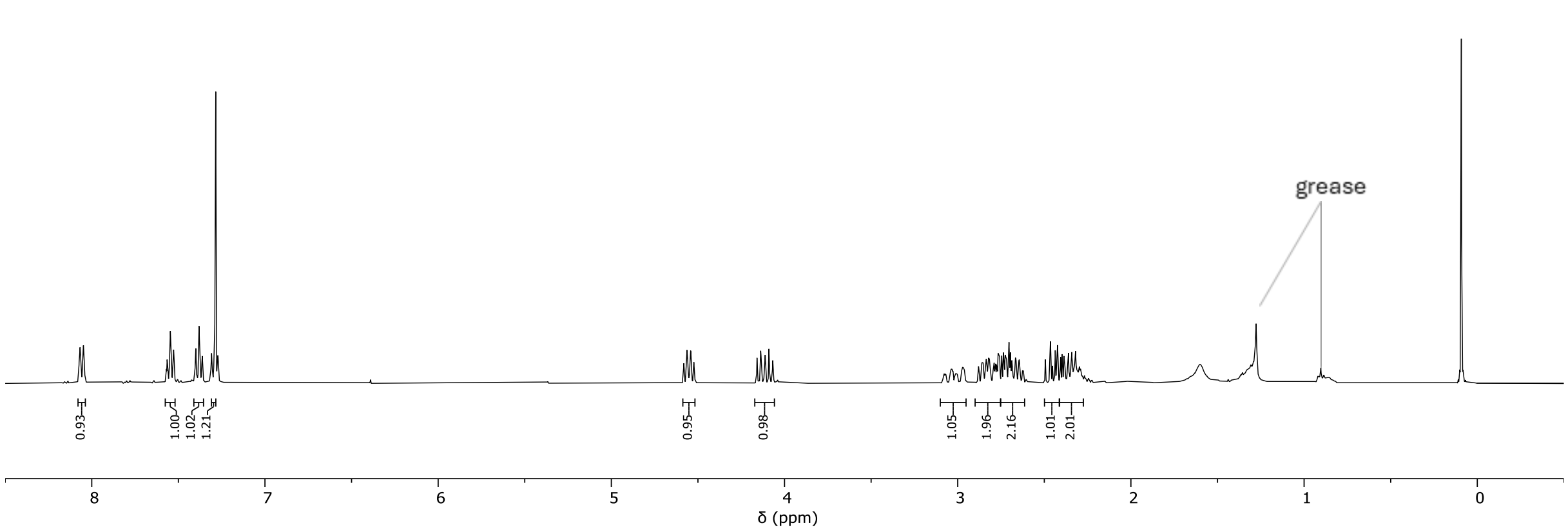

grease
δ (ppm)

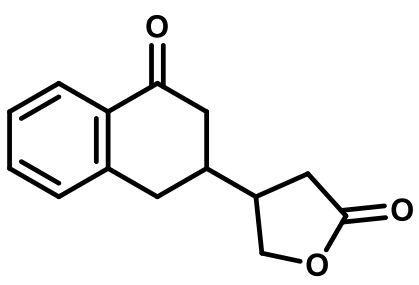

19

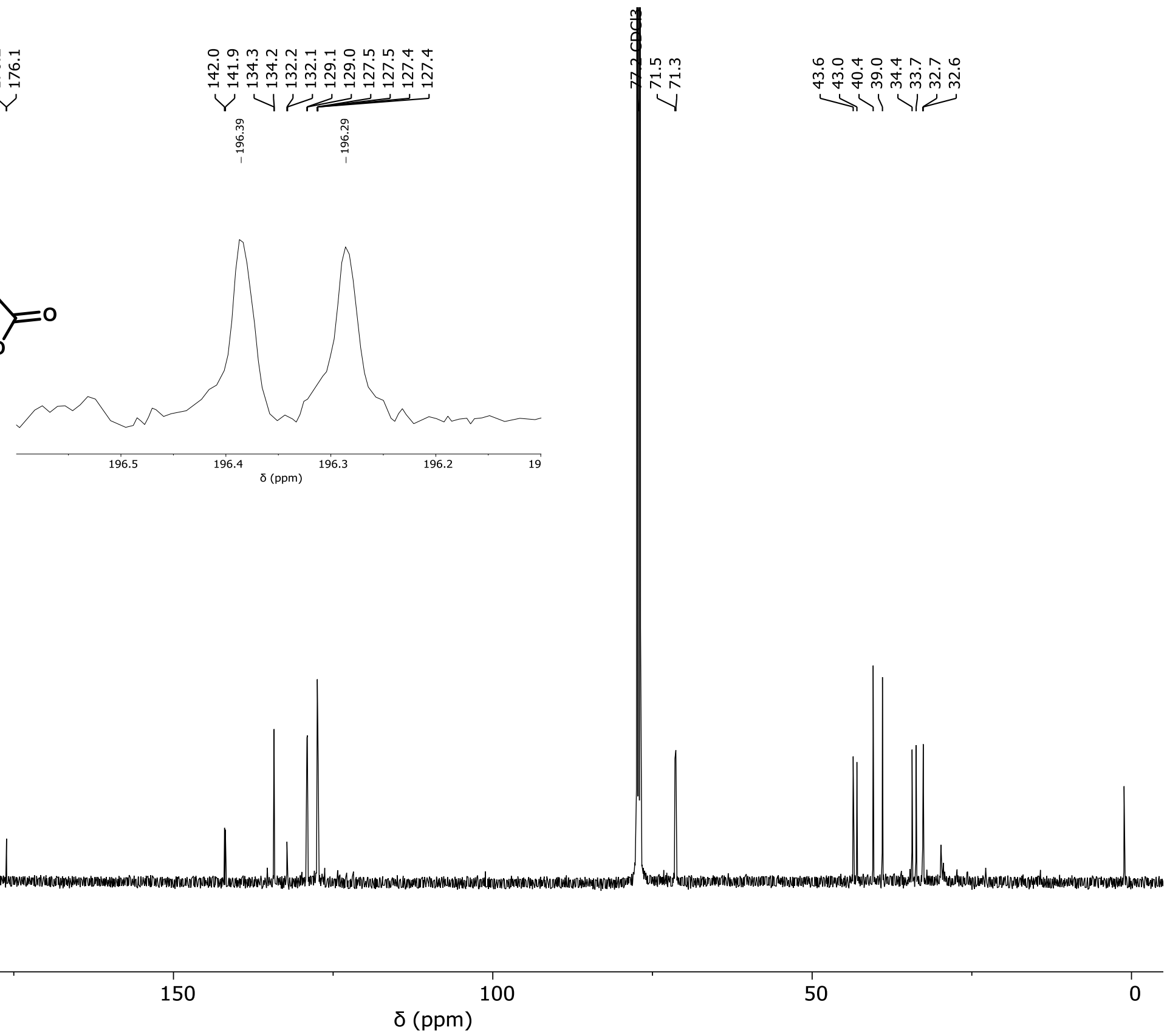

196.4
196.3
176.2
176.1
142.0
141.9
134.3
134.2
132.2
132.1
129.1
129.0
127.5
127.5
127.4
127.4
77.2 CDCl3
71.5
71.3
43.6
43.0
40.4
39.0
34.4
33.7
32.7
32.6
196.39
196.29
δ (ppm)

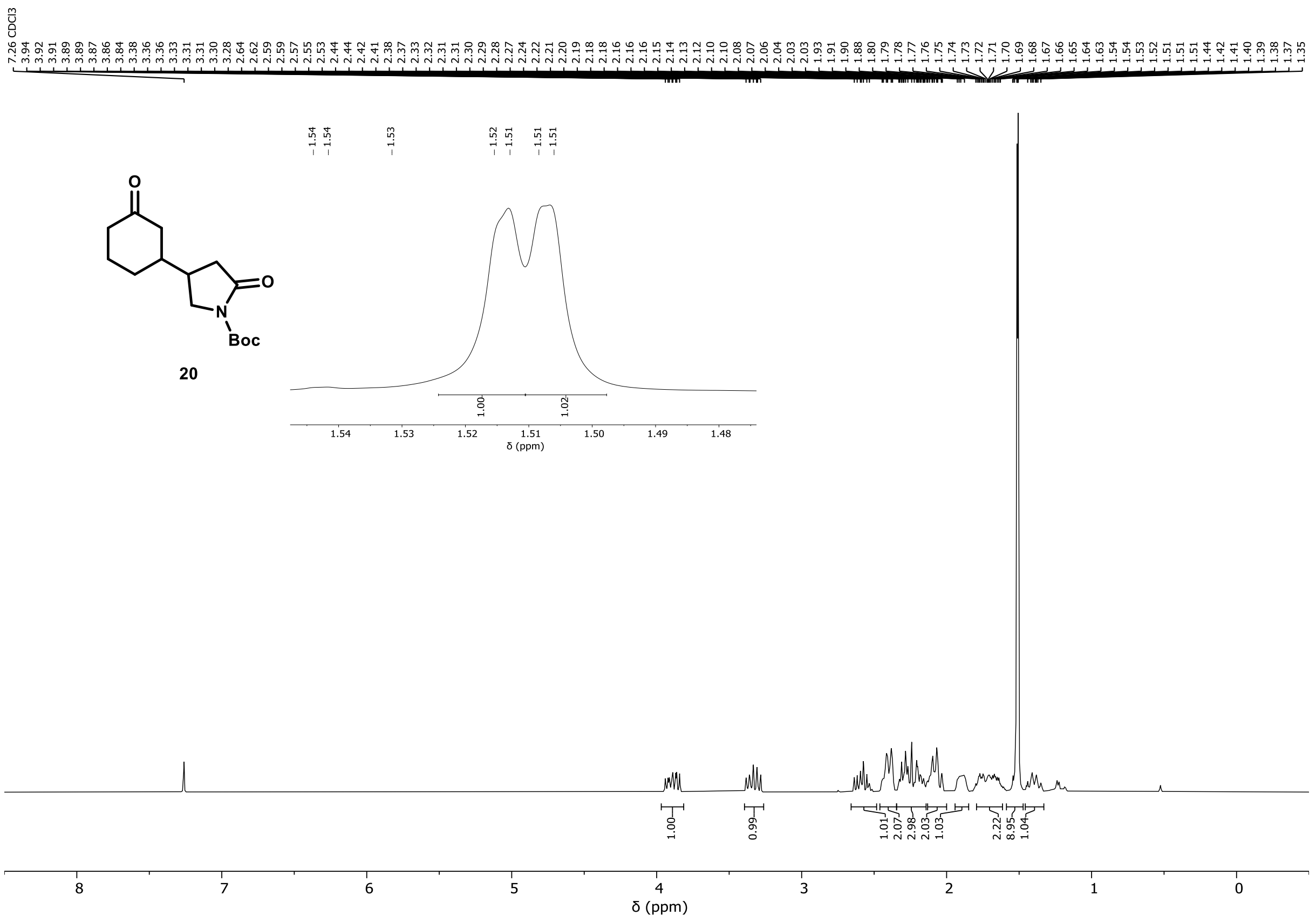

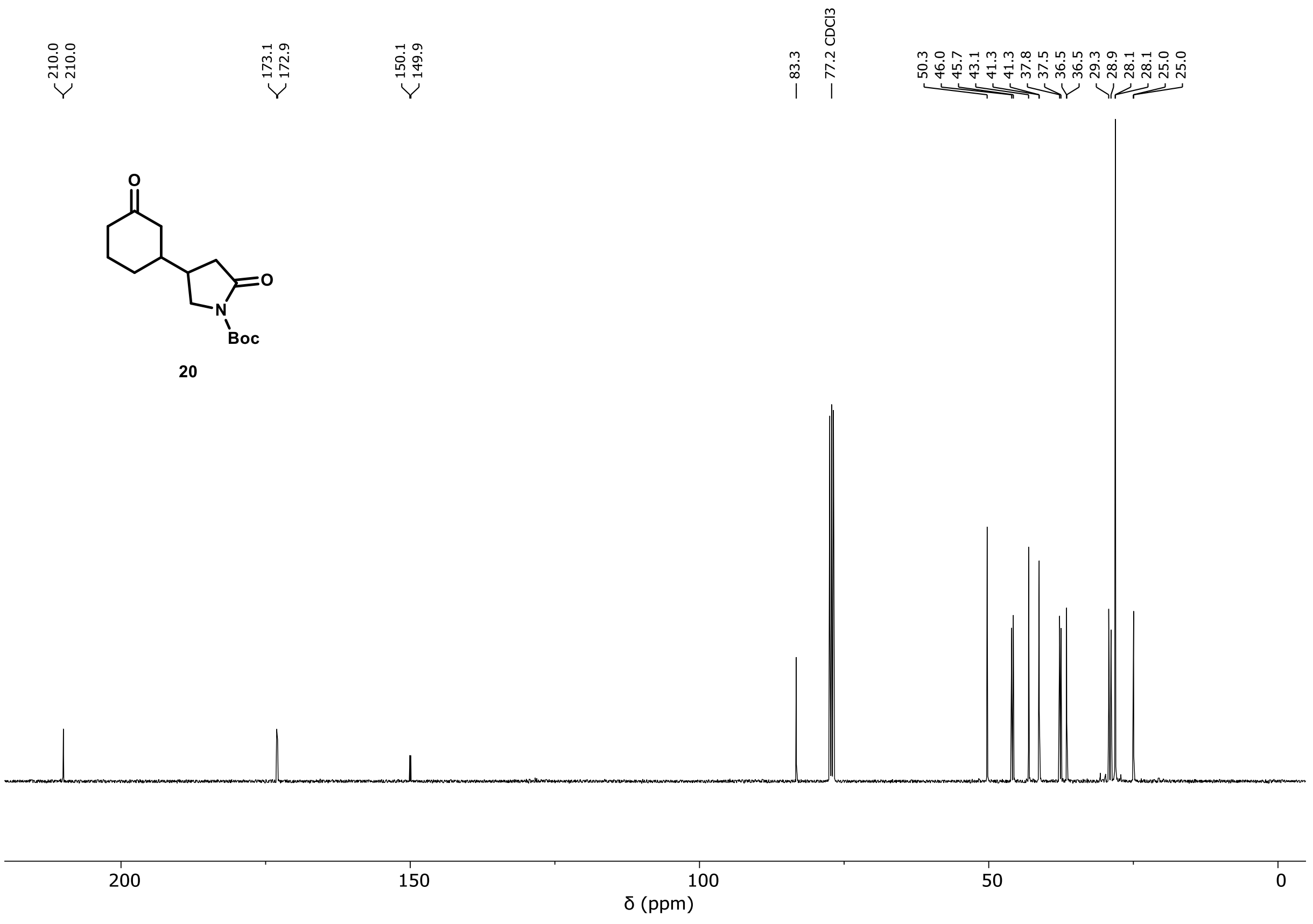

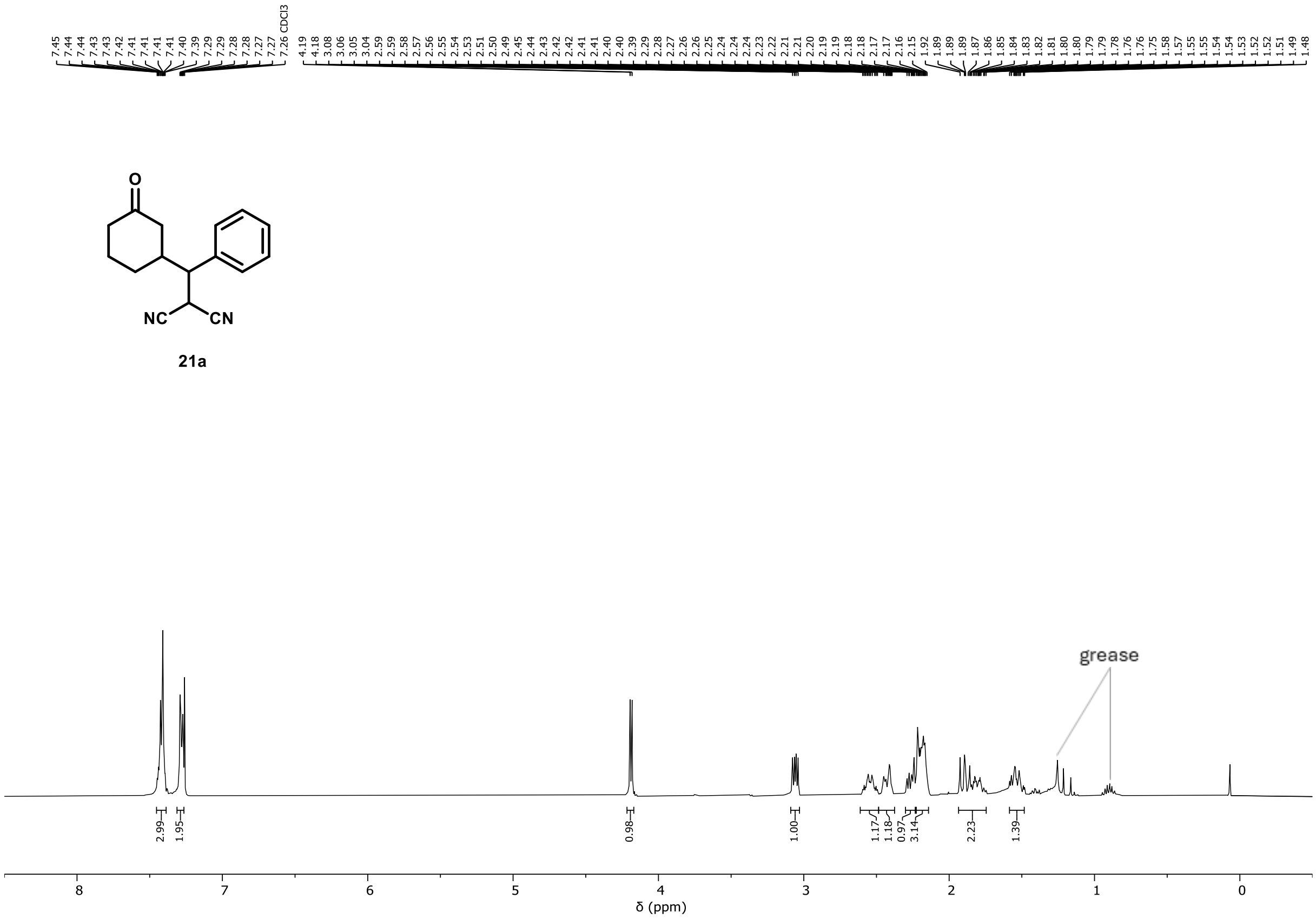
7.45 7.44 7.44 7.43 7.43 7.42 7.41 7.41 7.41 7.41 7.40 7.39 7.29 7.29 7.28 7.28 7.27 7.27 7.26 CDCl3
grease
2.99 1.95 0.98 1.00 1.17 1.18 0.97 3.14 2.23 1.39
8 7 6 5 4 3 2 1 0
δ (ppm)
O
NC
CN
21a

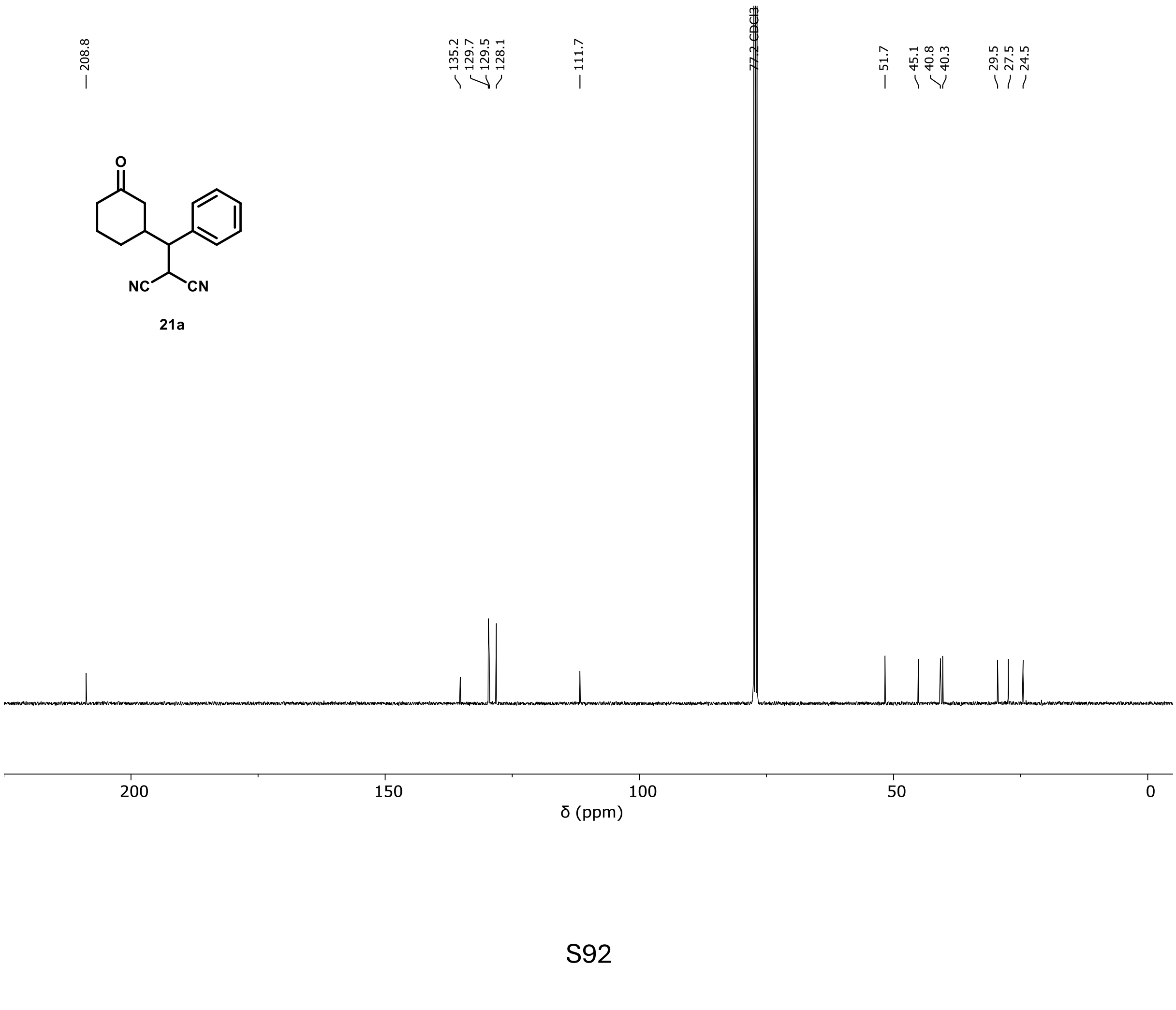
208.8
135.2 129.7 129.5 128.1
111.7
77.2 CDCl3
51.7
45.1 40.8 40.3
29.5 27.5 24.5
200 150 100 50 0
δ (ppm)
O
NC
CN
21a

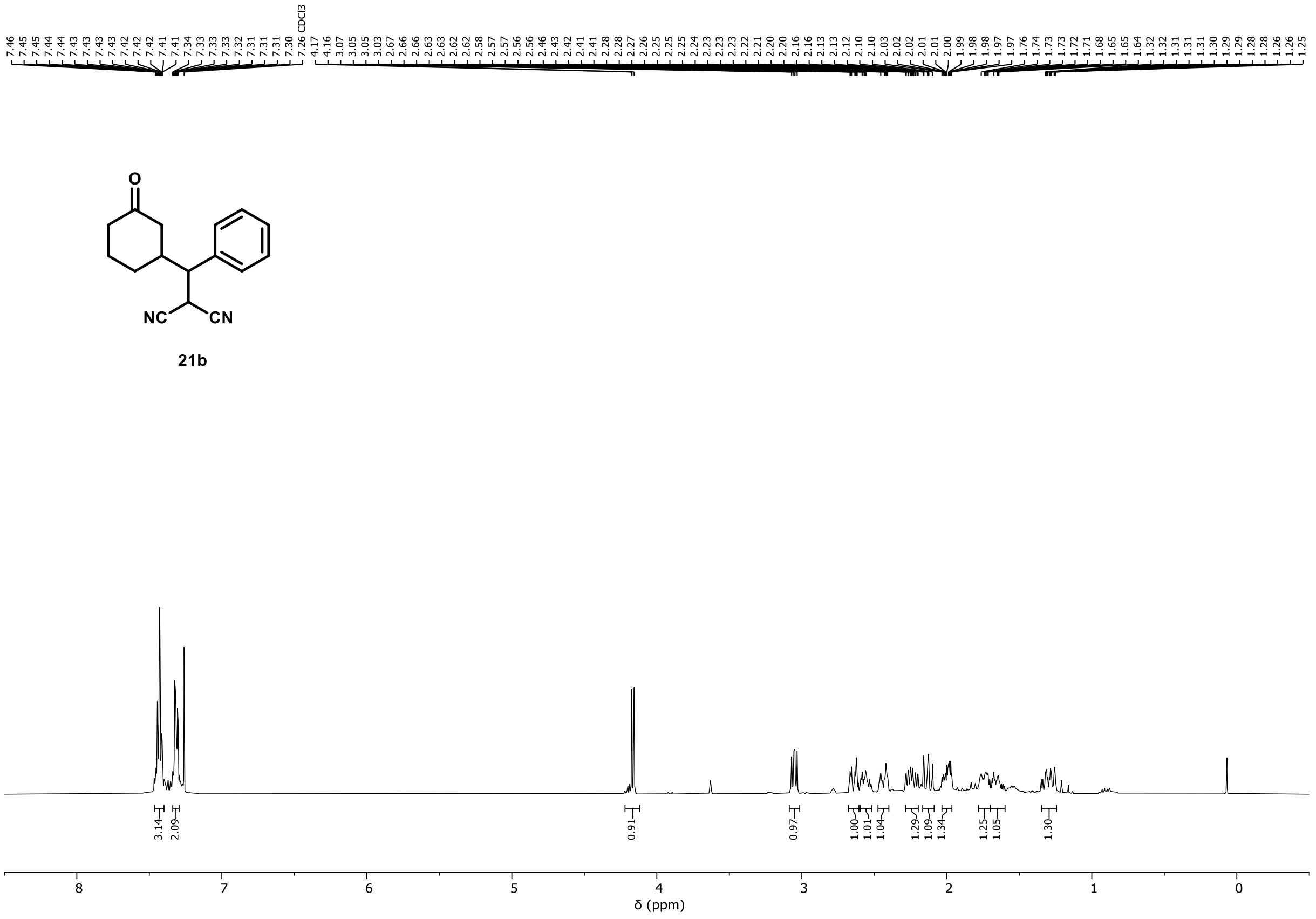

7.46 7.45 7.45 7.44 7.44 7.43 7.43 7.43 7.43 7.42 7.42 7.42 7.41 7.41 7.34 7.33 7.33 7.33 7.32 7.31 7.31 7.30 7.26 CDCl3 4.17 4.16 3.07 3.05 3.05 3.03 2.67 2.66 2.63 2.63 2.62 2.62 2.58 2.57 2.57 2.56 2.56 2.46 2.43 2.42 2.41 2.41 2.28 2.28 2.27 2.26 2.25 2.25 2.24 2.23 2.23 2.23 2.22 2.21 2.20 2.20 2.16 2.16 2.13 2.13 2.13 2.12 2.10 2.10 2.03 2.02 2.02 2.01 2.01 2.00 1.99 1.98 1.98 1.97 1.97 1.76 1.74 1.73 1.73 1.72 1.71 1.68 1.65 1.65 1.64 1.32 1.32 1.31 1.31 1.30 1.29 1.29 1.28 1.28 1.26 1.26 1.25
O
NC
CN
21b
3.14 2.09 0.91 0.97 1.00 1.01 1.04 1.29 1.09 1.34 1.25 1.05 1.30
8 7 6 5 4 3 2 1 0
δ (ppm)

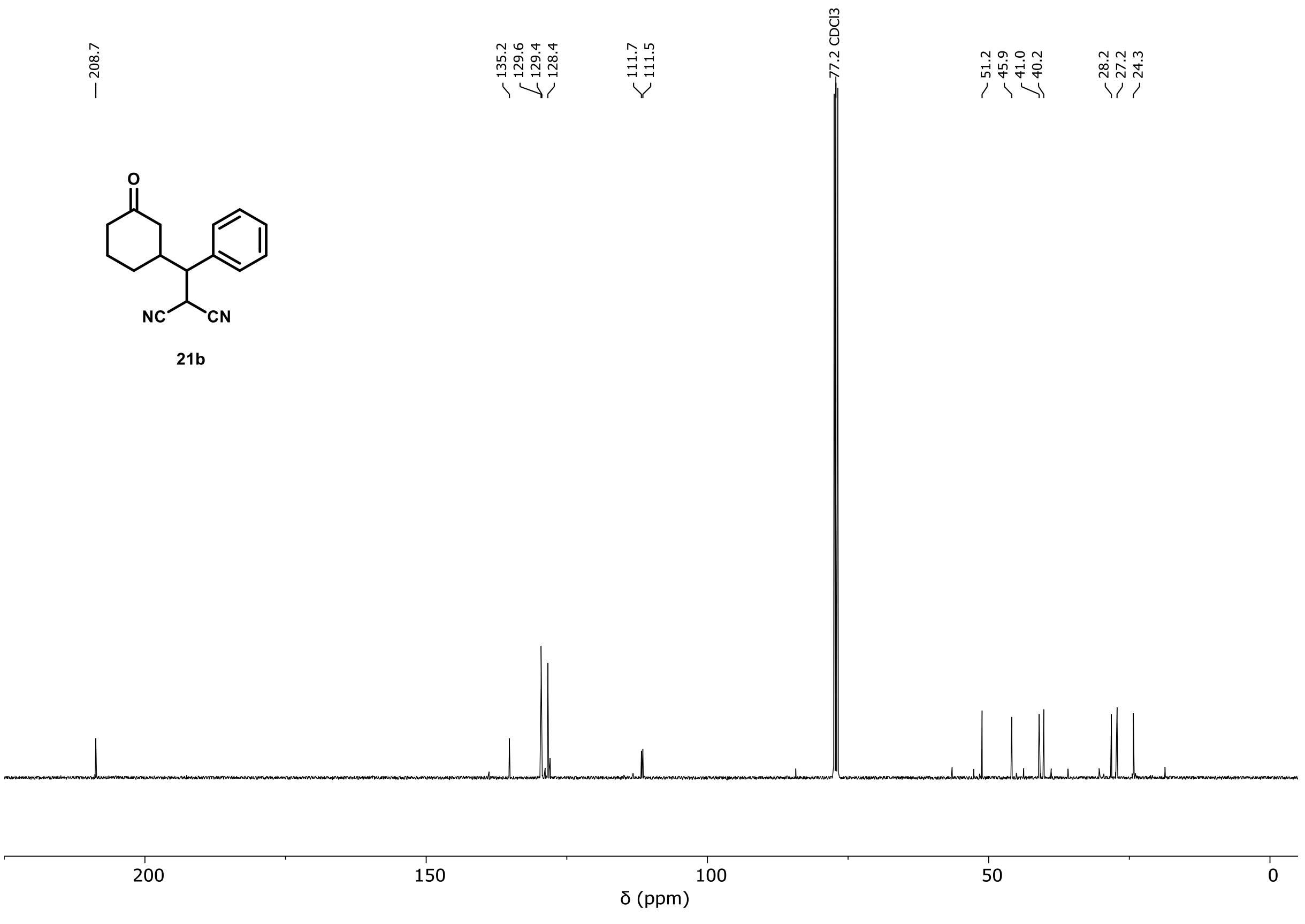

208.7
135.2 129.6 129.4 128.4
111.7 111.5
77.2 CDCl3
51.2 45.9 41.0 40.2
28.2 27.2 24.3
O
NC
CN
21b
200 150 100 50 0
δ (ppm)

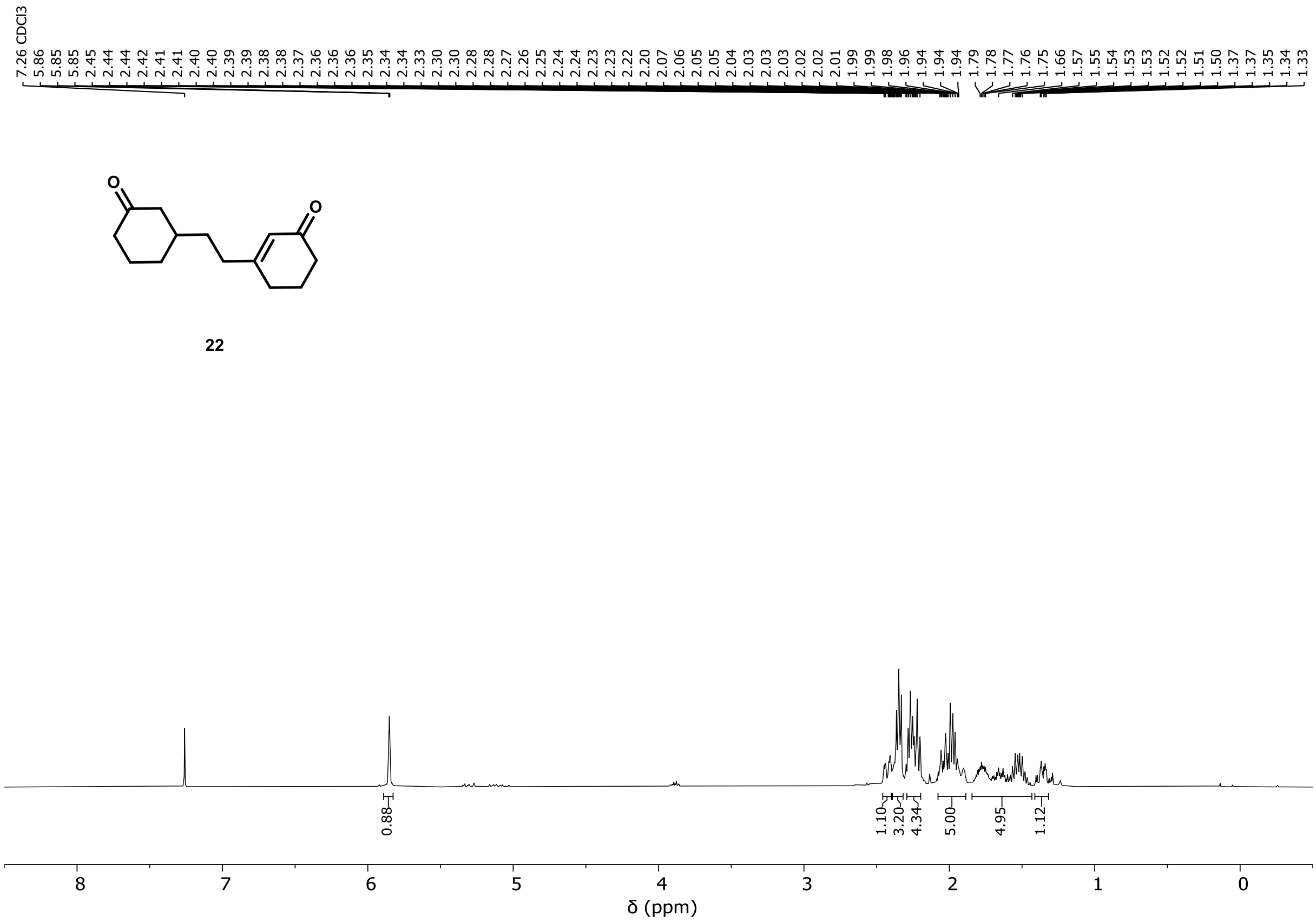

7.26 CDCl3
5.86
5.85
5.85
2.45
2.44
2.44
2.42
2.41
2.41
2.40
2.39
2.39
2.38
2.38
2.37
2.36
2.36
2.36
2.35
2.34
2.34
2.33
2.30
2.30
2.28
2.28
2.27
2.26
2.25
2.24
2.24
2.23
2.23
2.22
2.20
2.07
2.06
2.05
2.05
2.04
2.03
2.03
2.03
2.02
2.02
2.01
1.99
1.99
1.98
1.96
1.94
1.94
1.94
1.79
1.78
1.77
1.76
1.75
1.66
1.57
1.55
1.54
1.53
1.53
1.52
1.52
1.51
1.50
1.37
1.37
1.35
1.34
1.33
O
O
22
0.88
1.10
3.20
4.34
5.00
4.95
1.12
8
7
6
5
4
3
2
1
0
δ (ppm)

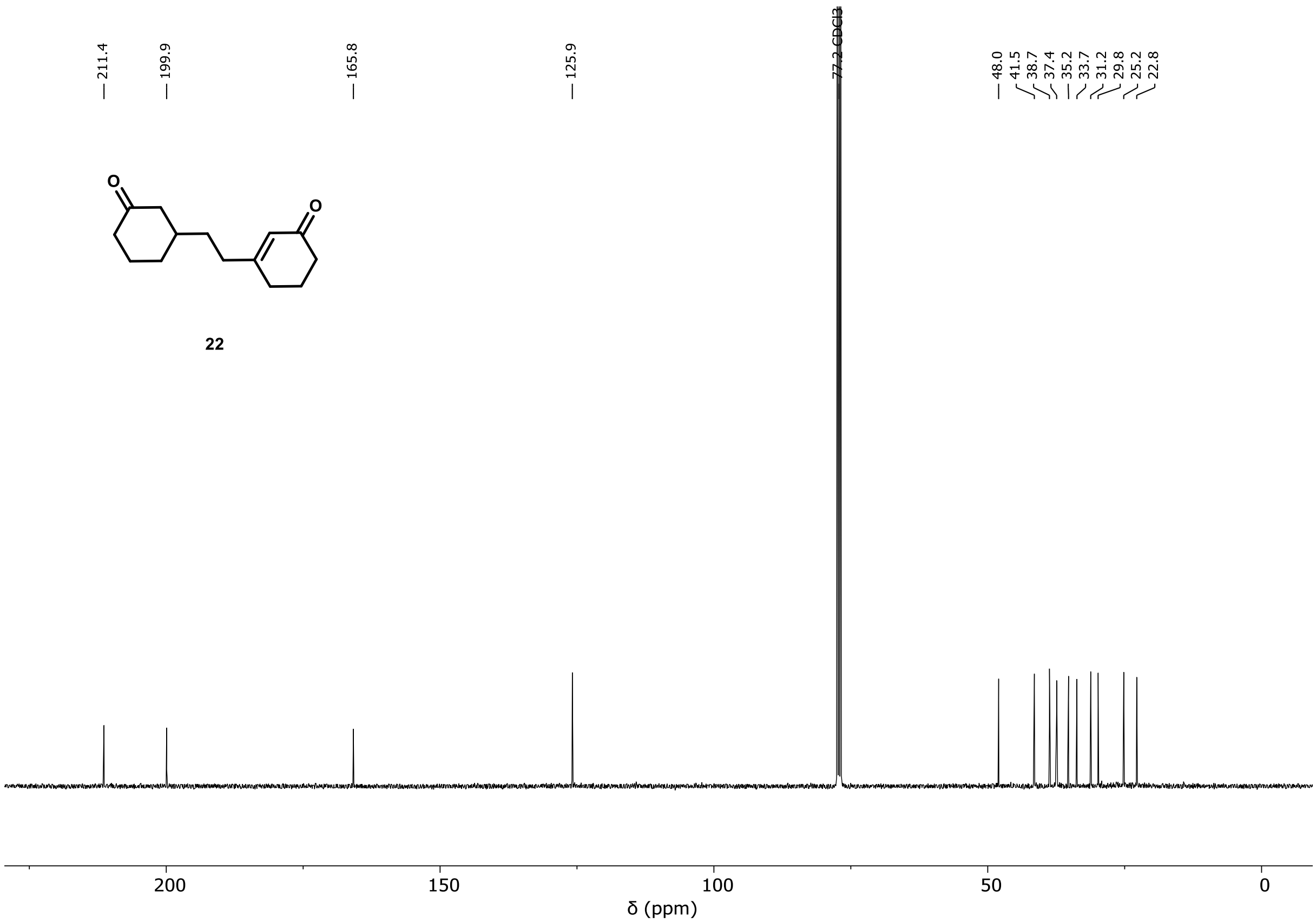

211.4
199.9
165.8
125.9
77.2 CDCl3
48.0
41.5
38.7
37.4
35.2
33.7
31.2
29.8
25.2
22.8
O
O
22
200
150
100
50
0
δ (ppm)

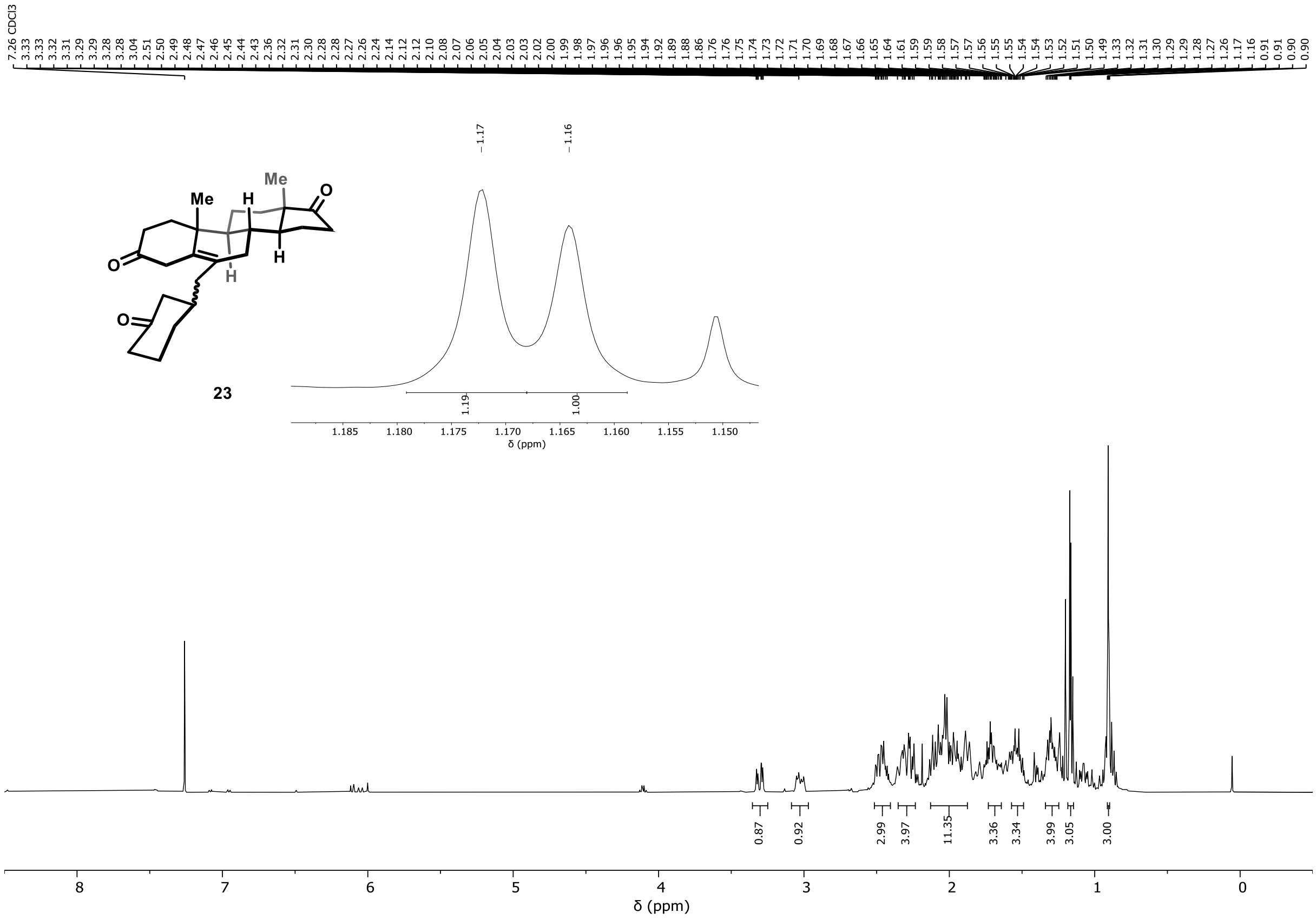

7.26 CDCl3
3.33 3.33 3.32 3.31 3.29 3.29 3.28 3.28 3.04 2.51 2.50 2.49 2.48 2.47 2.46 2.45 2.44 2.43 2.36 2.32 2.31 2.30 2.28 2.28 2.27 2.26 2.24 2.14 2.12 2.12 2.10 2.08 2.07 2.06 2.05 2.04 2.03 2.03 2.02 2.00 1.99 1.98 1.97 1.96 1.96 1.95 1.94 1.92 1.89 1.88 1.86 1.76 1.76 1.75 1.74 1.73 1.72 1.71 1.70 1.69 1.68 1.67 1.66 1.65 1.64 1.61 1.59 1.59 1.58 1.57 1.57 1.56 1.55 1.55 1.54 1.54 1.53 1.52 1.51 1.50 1.49 1.33 1.32 1.31 1.30 1.29 1.29 1.28 1.27 1.26 1.17 1.16 0.91 0.91 0.90 0.90
1.17
1.16
1.19
1.00
1.185 1.180 1.175 1.170 1.165 1.160 1.155 1.150
δ (ppm)
0.87 0.92 2.99 3.97 11.35 3.36 3.34 3.99 3.05 3.00
23
8 7 6 5 4 3 2 1 0
δ (ppm)

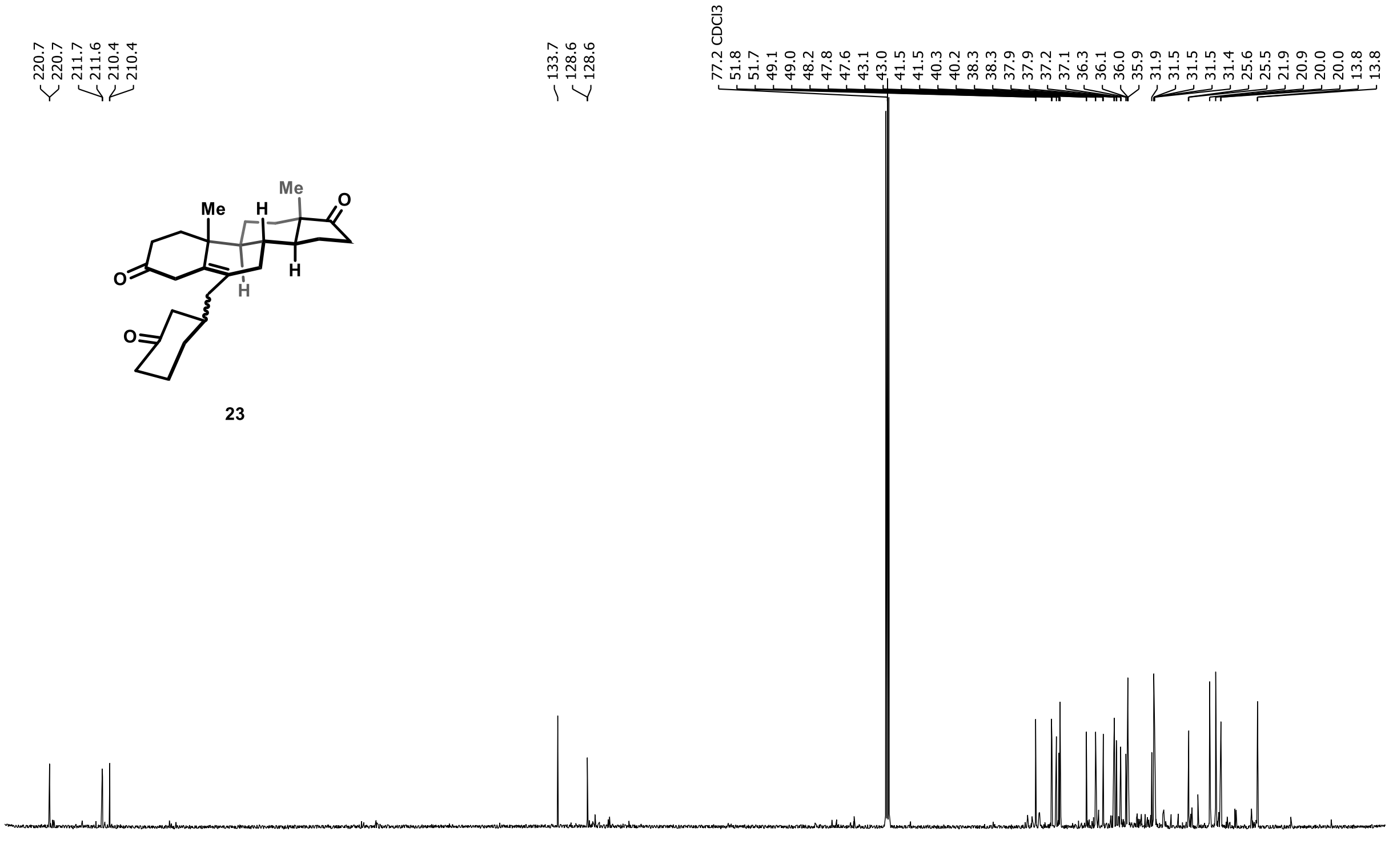

220.7 220.7 211.7 211.6 210.4 210.4
133.7 128.6 128.6
77.2 CDCl3
51.8 51.7 49.1 49.0 48.2 47.8 47.6 43.1 43.0 41.5 41.5 40.3 40.2 38.3 38.3 37.9 37.9 37.2 37.1 36.3 36.1 36.0 35.9 31.9 31.5 31.5 31.5 31.4 25.6 25.5 21.9 20.9 20.0 20.0 13.8 13.8
23
200 150 100 50 0
δ (ppm)

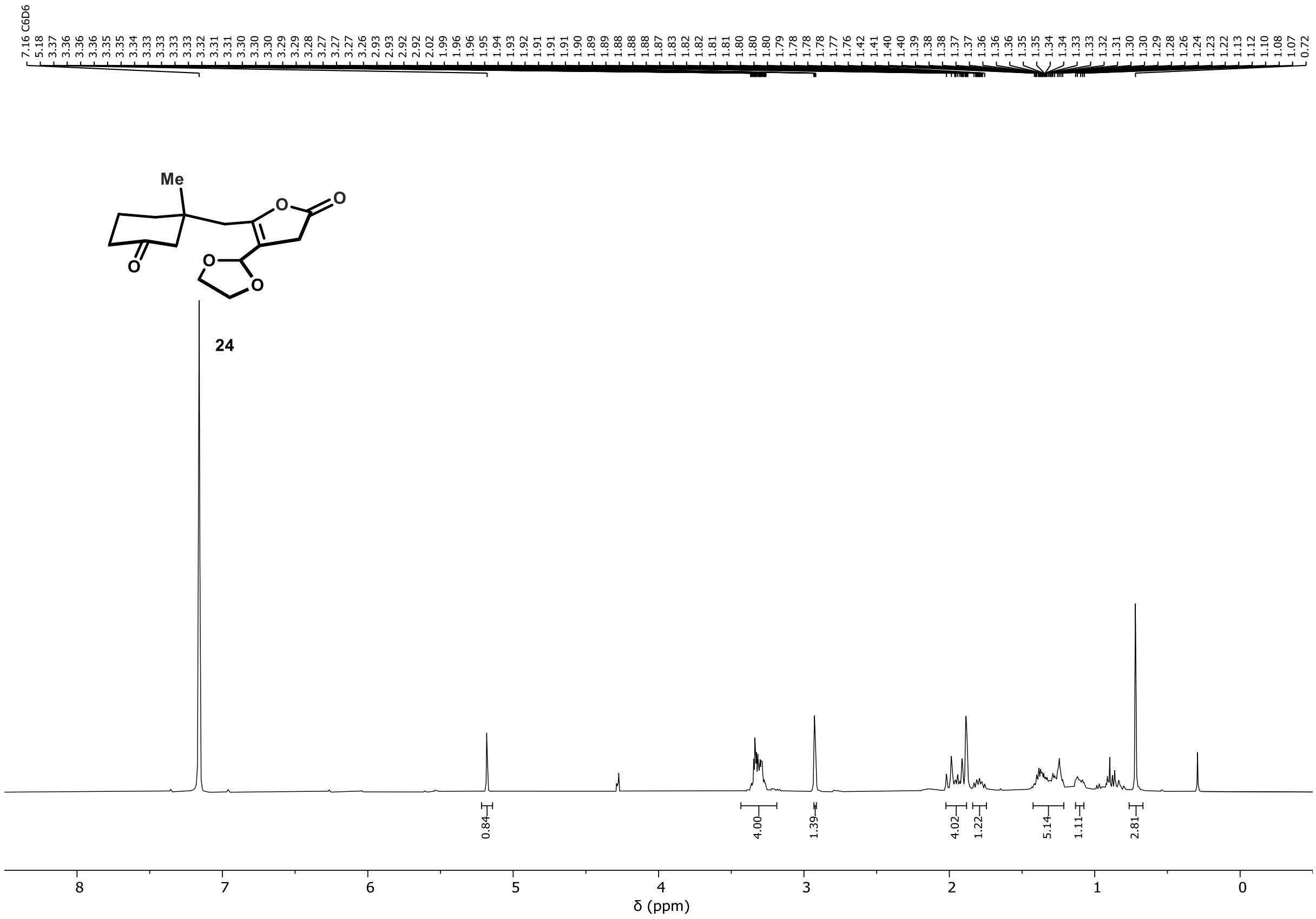
Me
O
O
O
O
O
24
0.84
4.00
1.39
4.02
1.22
5.14
1.11
2.81
δ (ppm)

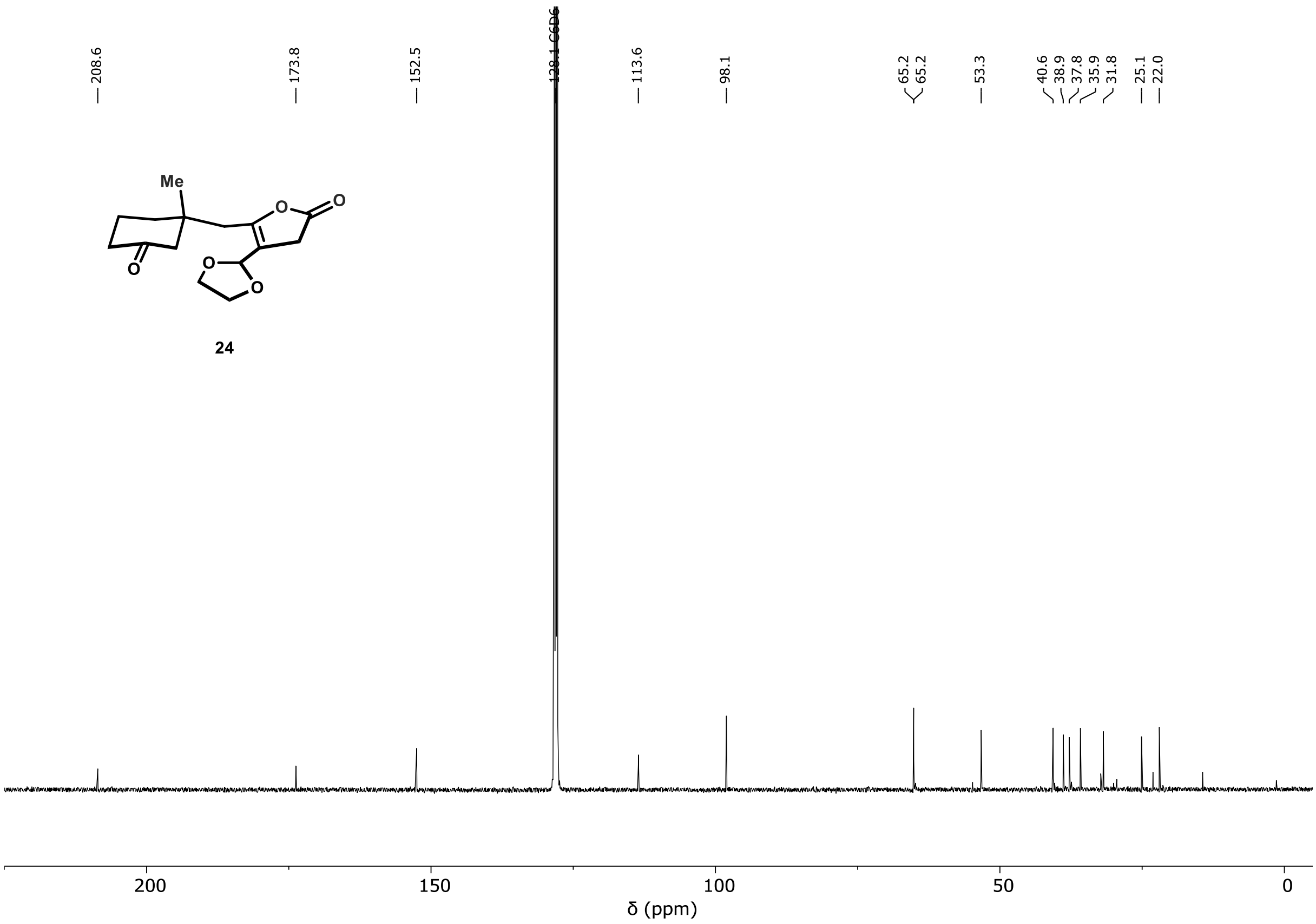
208.6
173.8
152.5
128.1 C6D6
113.6
98.1
65.2
65.2
53.3
40.6
38.9
37.8
35.9
31.8
25.1
22.0
Me
O
O
O
O
O
24
δ (ppm)

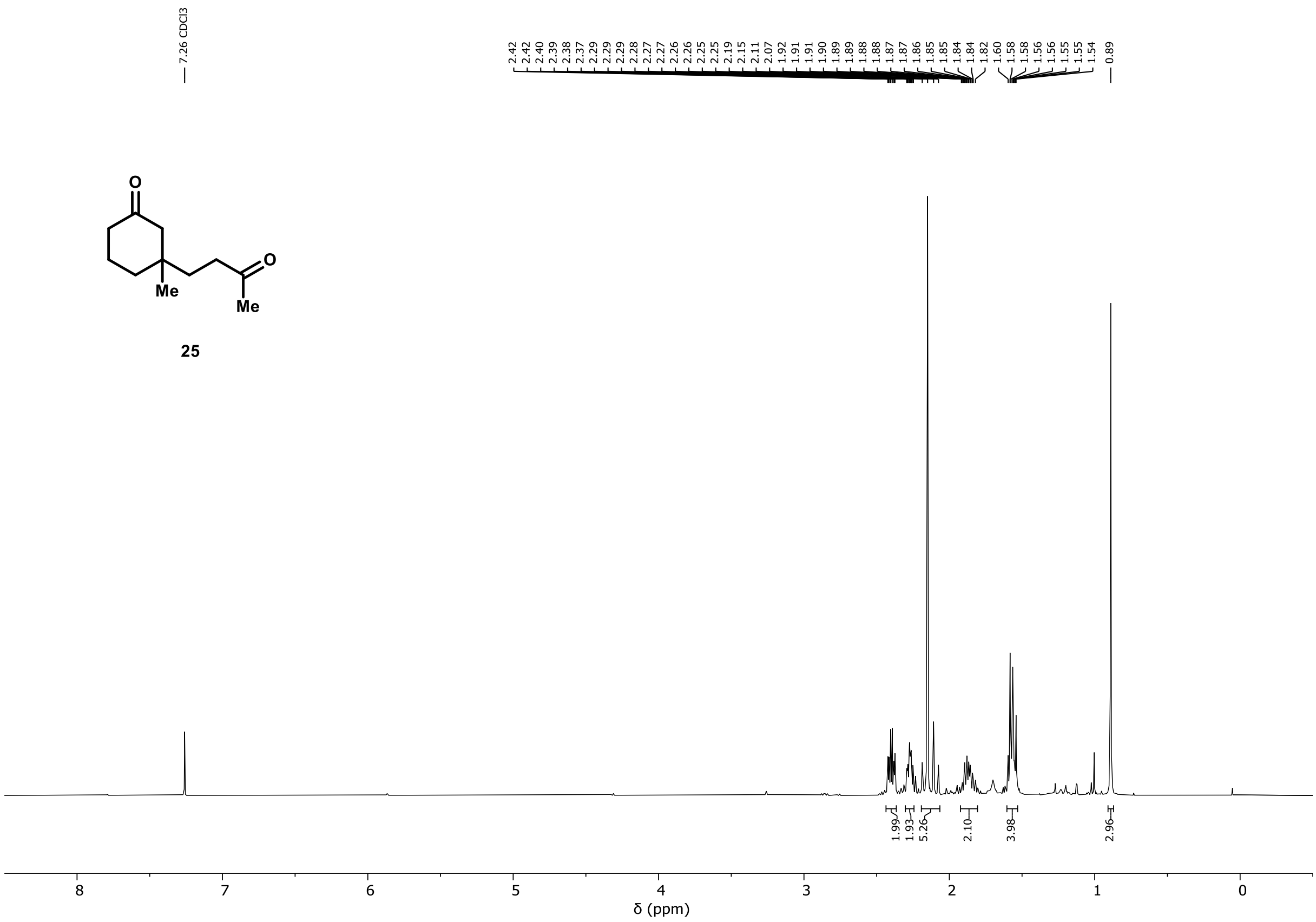
7.26 CDCl3
2.42
2.42
2.40
2.39
2.38
2.37
2.29
2.29
2.29
2.28
2.27
2.27
2.26
2.26
2.25
2.25
2.19
2.15
2.11
2.07
1.92
1.91
1.91
1.90
1.89
1.89
1.88
1.87
1.87
1.86
1.85
1.85
1.84
1.84
1.82
1.60
1.58
1.58
1.56
1.56
1.55
1.55
1.54
0.89
O
O
Me
Me
25
1.99
1.93
5.26
2.10
3.98
2.96
8
7
6
5
4
3
2
1
0
δ (ppm)

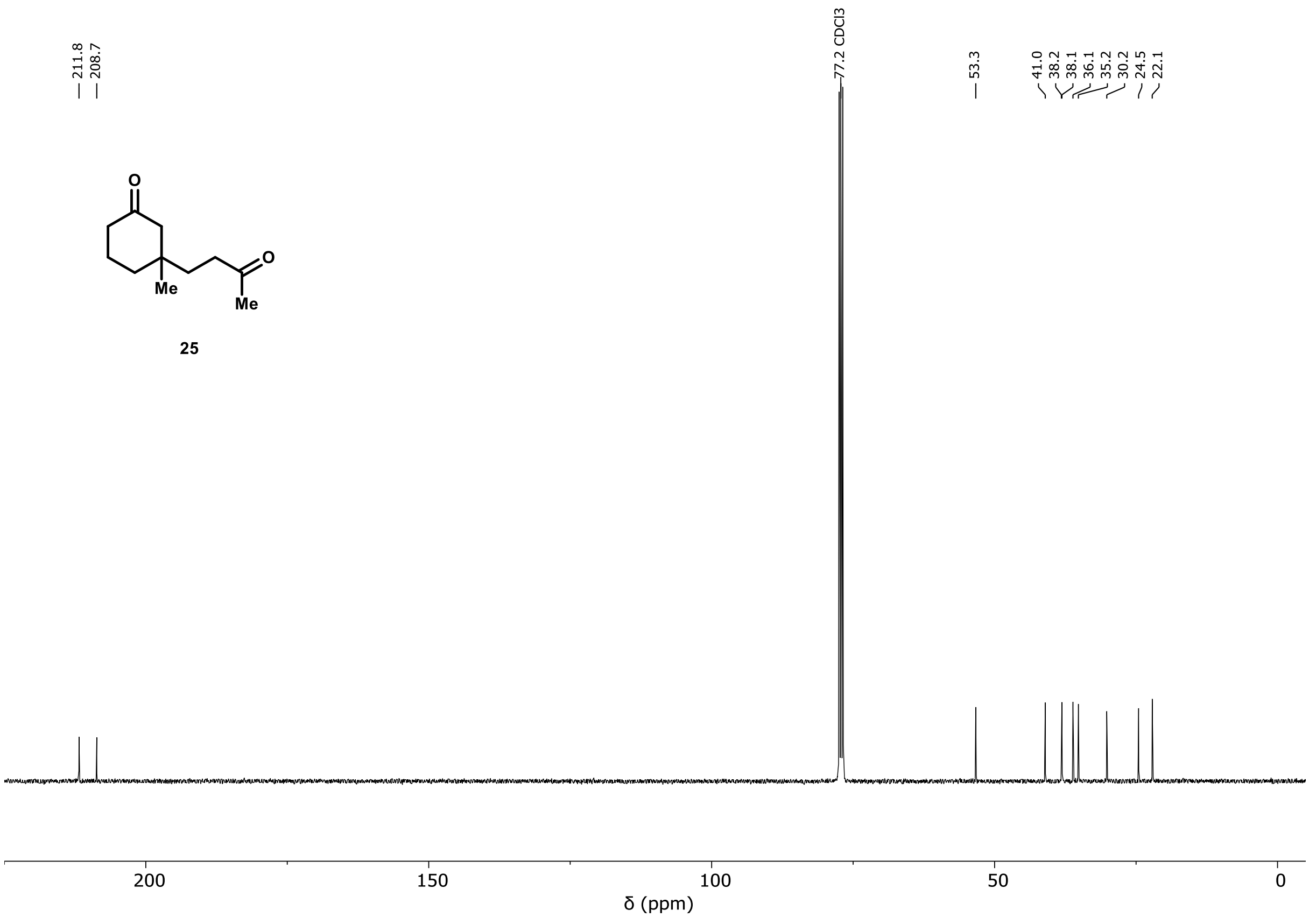
211.8
208.7
77.2 CDCl3
53.3
41.0
38.2
38.1
36.1
35.2
30.2
24.5
22.1
O
O
Me
Me
25
200
150
100
50
0
δ (ppm)

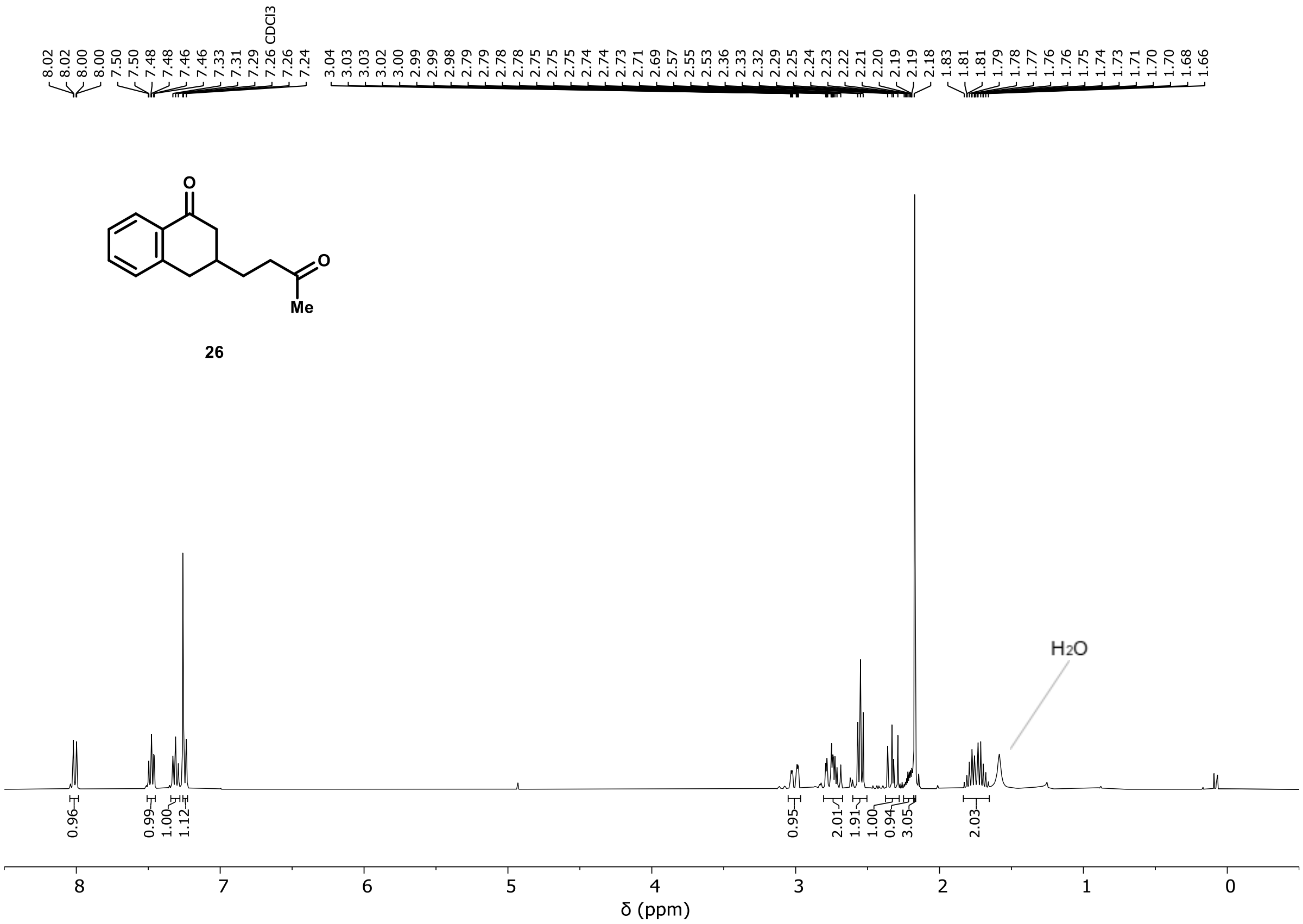
8.02
8.02
8.00
8.00
7.50
7.48
7.48
7.46
7.46
7.33
7.31
7.29
7.26 CDCl3
7.26
7.24
3.04
3.03
3.03
3.02
3.00
2.99
2.99
2.98
2.79
2.79
2.79
2.78
2.78
2.75
2.75
2.74
2.74
2.73
2.71
2.69
2.57
2.55
2.53
2.36
2.33
2.32
2.29
2.25
2.24
2.23
2.22
2.21
2.20
2.19
2.19
2.18
1.83
1.81
1.81
1.79
1.78
1.77
1.76
1.76
1.75
1.74
1.73
1.71
1.70
1.70
1.68
1.66
O
O
Me
26
H2O
0.96
0.99
1.00
1.12
0.95
2.01
1.91
1.00
0.94
3.05
2.03
δ (ppm)

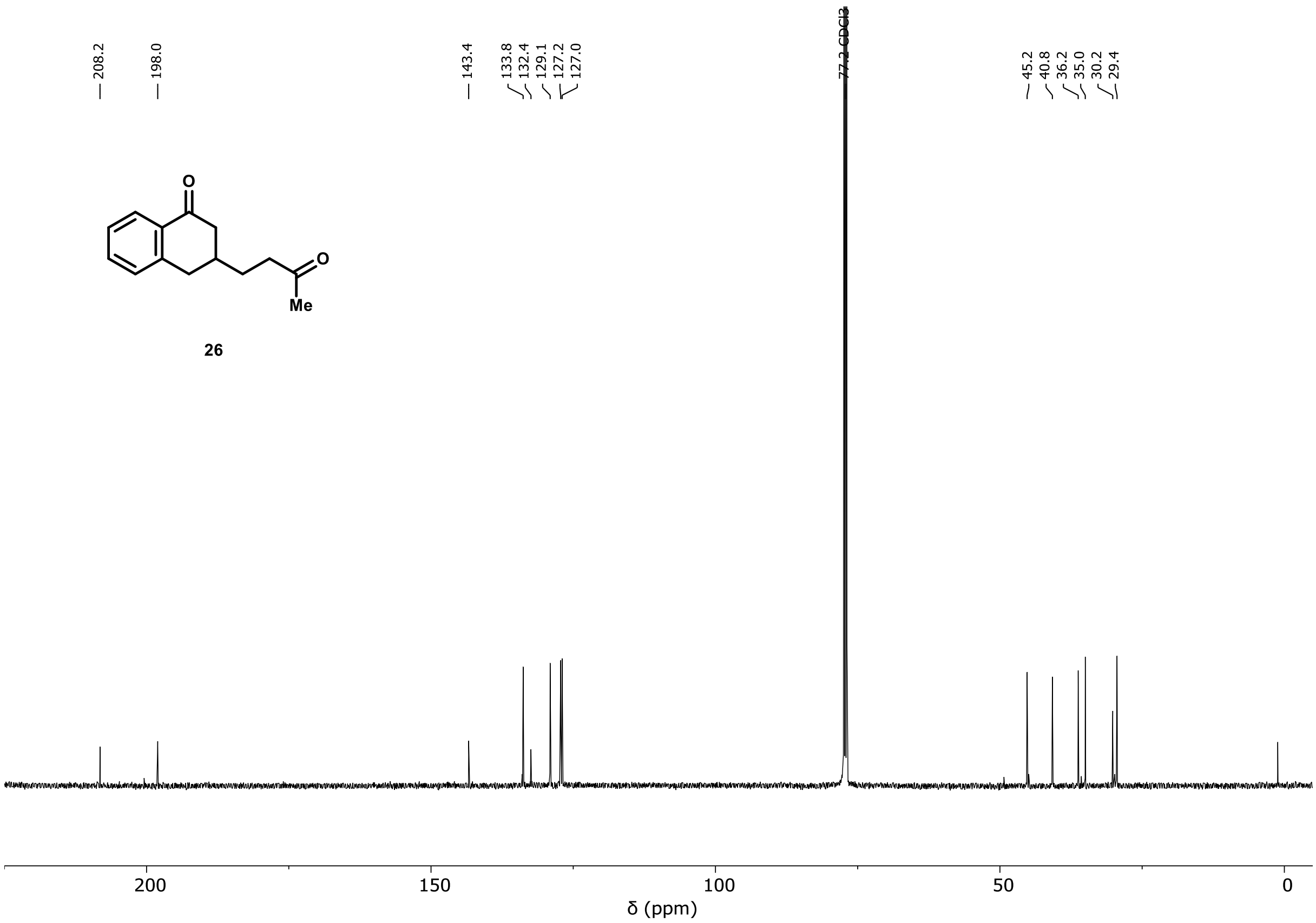
208.2
198.0
143.4
133.8
132.4
129.1
127.2
127.0
77.2 CDCl3
45.2
40.8
36.2
35.0
30.2
29.4
O
O
Me
26
δ (ppm)

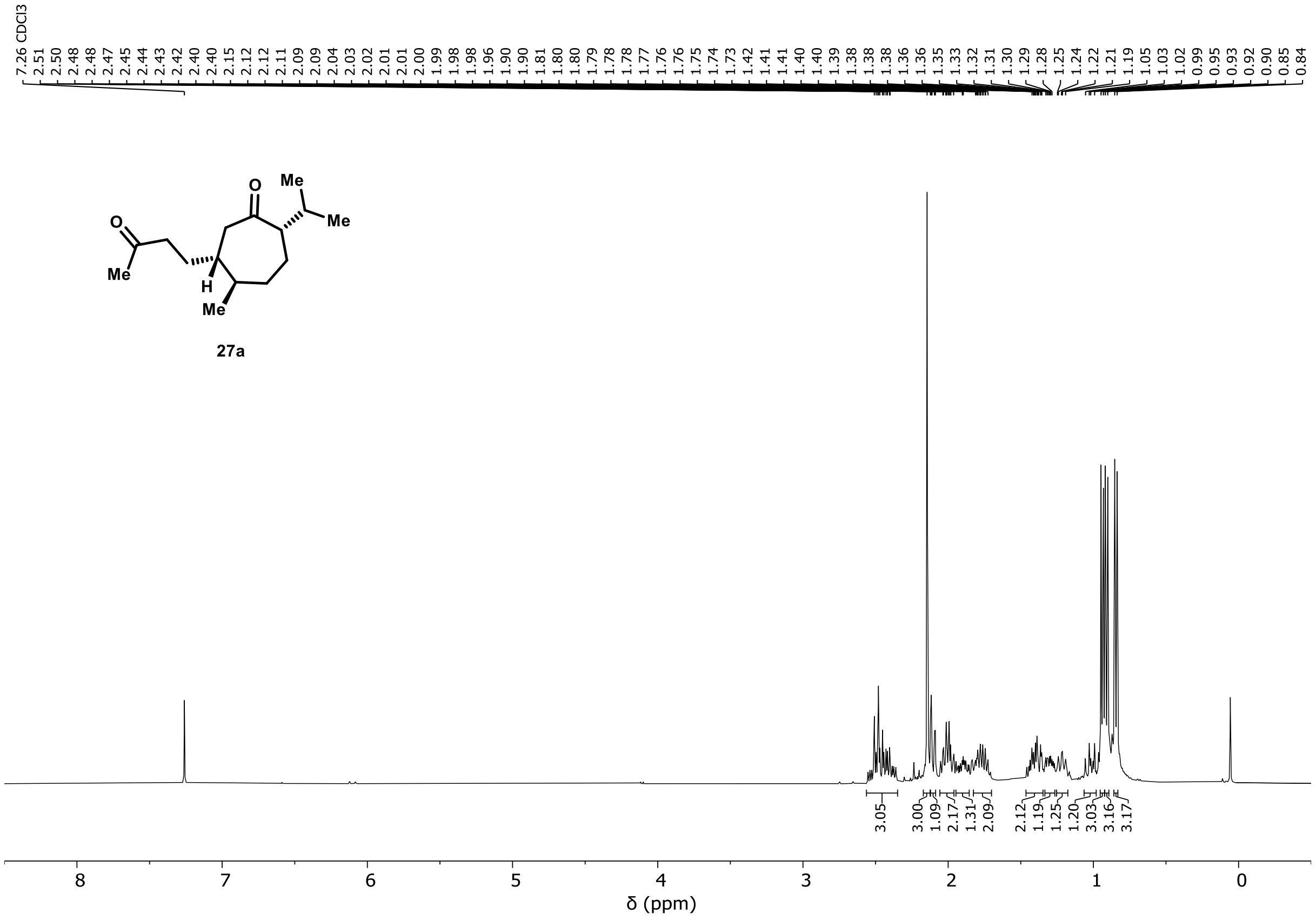
7.26 CDCl3
2.51
2.50
2.48
2.48
2.47
2.45
2.44
2.43
2.42
2.40
2.40
2.15
2.12
2.12
2.11
2.09
2.09
2.04
2.03
2.02
2.01
2.01
2.00
1.99
1.98
1.98
1.96
1.90
1.90
1.81
1.80
1.80
1.79
1.78
1.78
1.77
1.76
1.76
1.75
1.74
1.73
1.42
1.41
1.41
1.40
1.40
1.39
1.38
1.38
1.38
1.38
1.36
1.36
1.35
1.33
1.32
1.31
1.30
1.29
1.28
1.25
1.24
1.22
1.21
1.19
1.05
1.03
1.02
0.99
0.95
0.93
0.92
0.90
0.85
0.84
O
Me
Me
O
Me
H
Me
27a
3.05
3.00
1.09
2.17
1.31
2.09
2.12
1.19
1.25
1.20
3.03
3.16
3.17
8
7
6
5
4
3
2
1
0
δ (ppm)

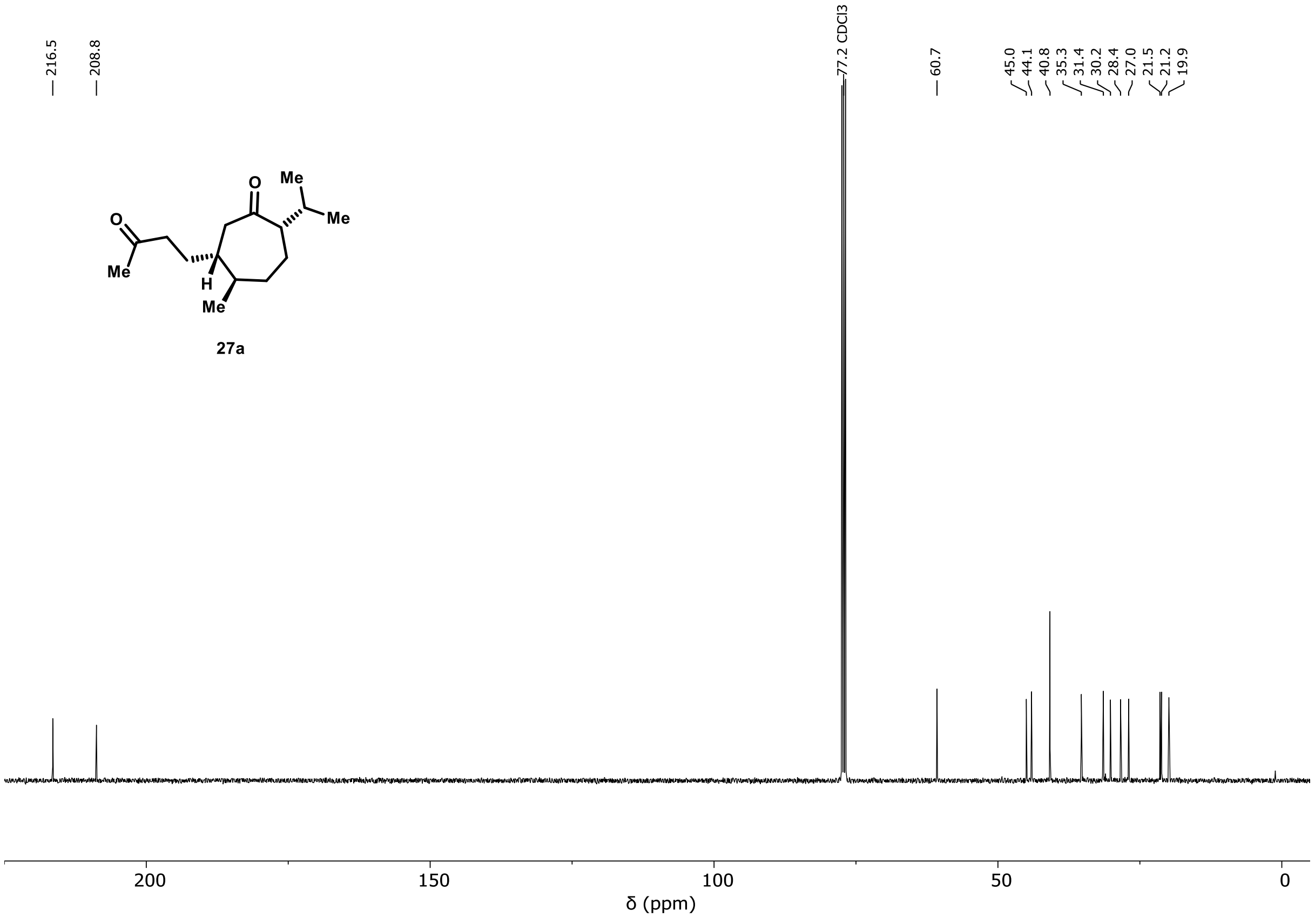
216.5
208.8
77.2 CDCl3
60.7
45.0
44.1
40.8
35.3
31.4
30.2
28.4
27.0
21.5
21.2
19.9
O
Me
Me
O
Me
H
Me
27a
200
150
100
50
0
δ (ppm)

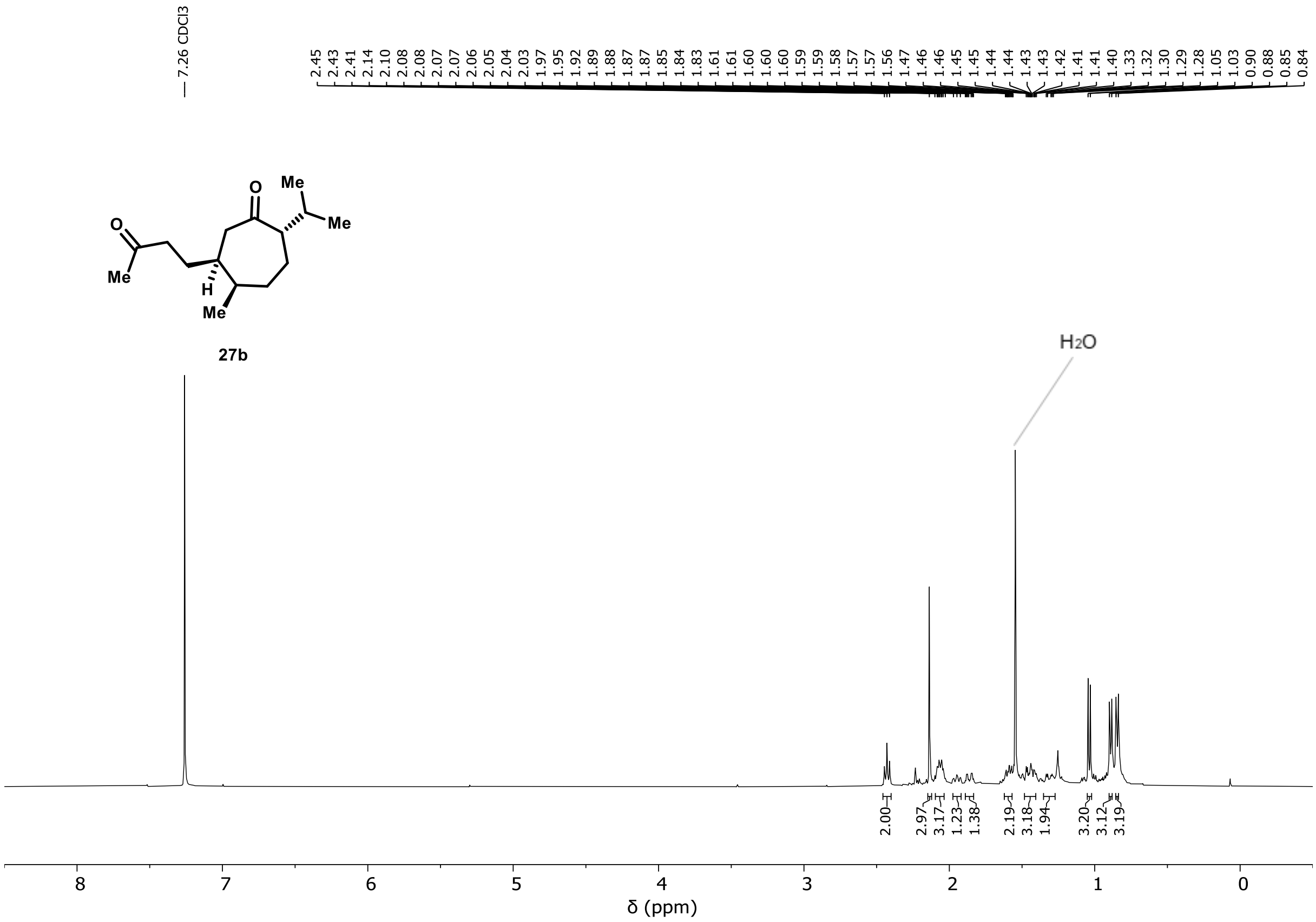
7.26 CDCl3
2.45 2.43 2.41 2.14 2.10 2.08 2.07 2.07 2.06 2.05 2.04 2.03 1.97 1.95 1.92 1.89 1.88 1.87 1.87 1.85 1.84 1.83 1.61 1.61 1.60 1.60 1.60 1.59 1.59 1.58 1.57 1.57 1.56 1.47 1.46 1.46 1.45 1.45 1.44 1.44 1.43 1.43 1.42 1.41 1.41 1.40 1.33 1.32 1.30 1.29 1.28 1.05 1.03 0.90 0.88 0.85 0.84
O
Me
Me
Me
H
Me
O
27b
H2O
2.00 2.97 3.17 1.23 1.38 2.19 3.18 1.94 3.20 3.12 3.19
8 7 6 5 4 3 2 1 0
δ (ppm)

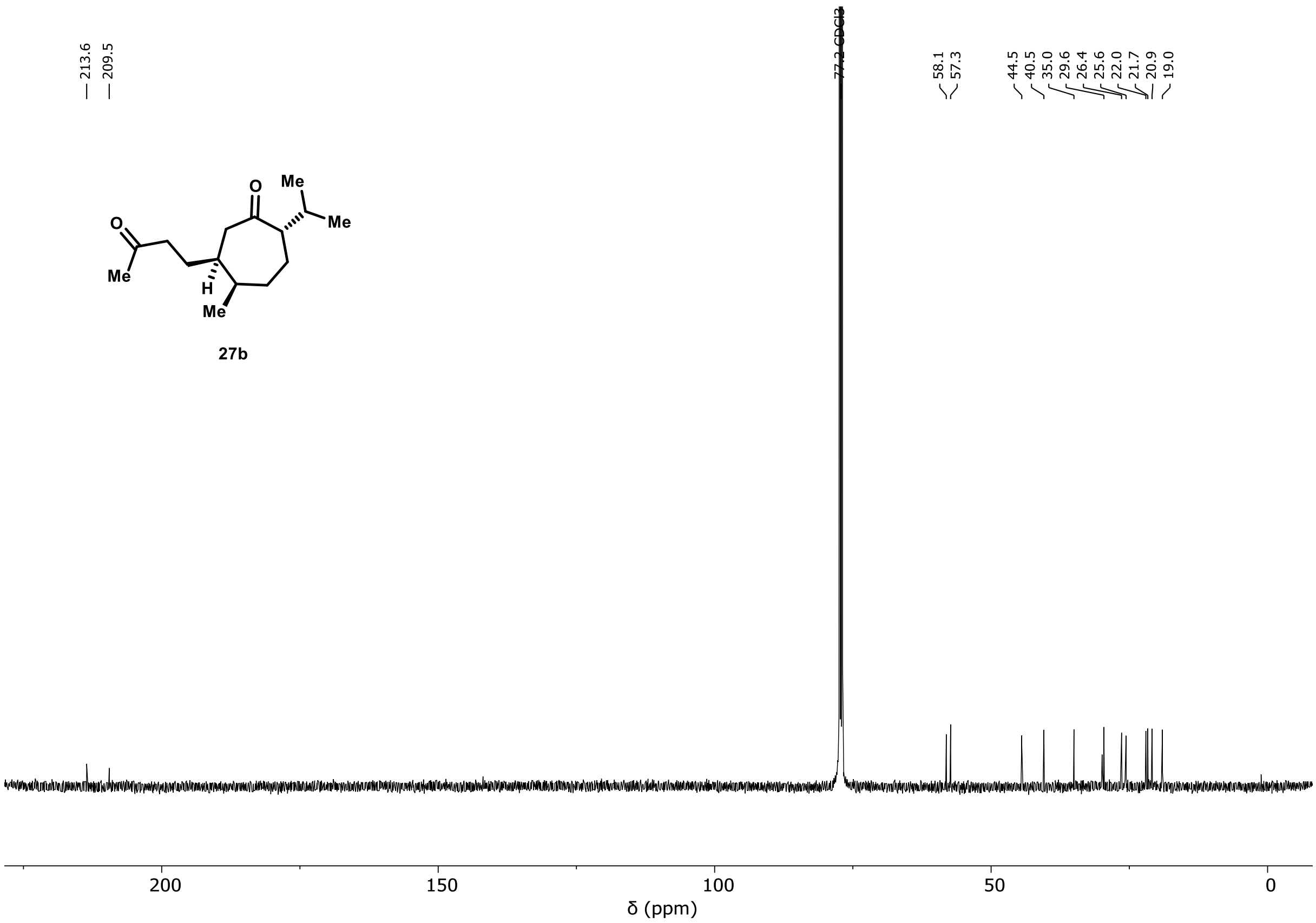
213.6
209.5
77.2 CDCl3
58.1 57.3
44.5 40.5 35.0 29.6 26.4 25.6 22.0 21.7 20.9 19.0
O
Me
Me
Me
H
Me
O
27b
200 150 100 50 0
δ (ppm)

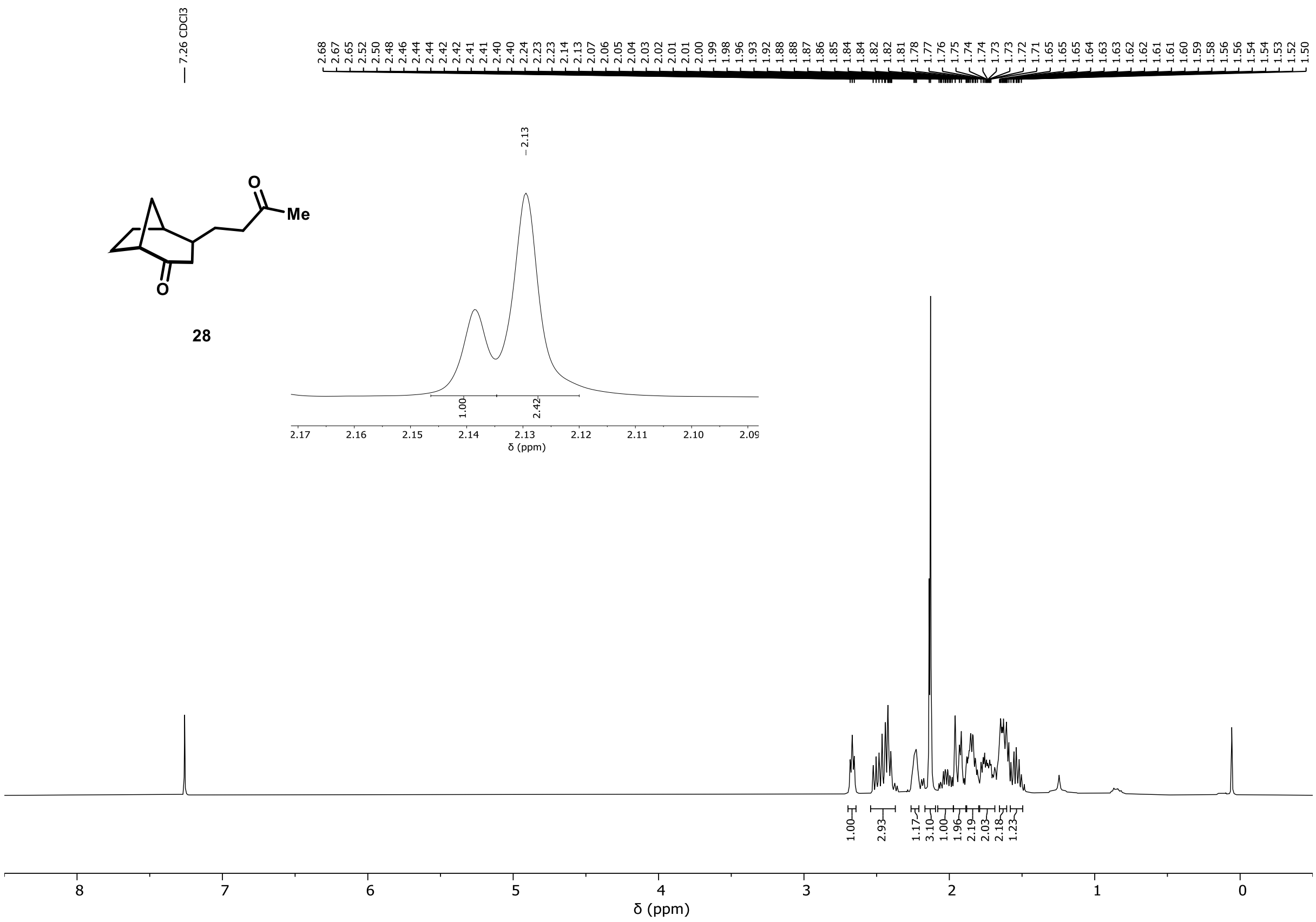
7.26 CDCl3
2.68 2.67 2.65 2.52 2.50 2.48 2.46 2.44 2.44 2.42 2.42 2.41 2.41 2.40 2.40 2.24 2.23 2.23 2.14 2.13 2.07 2.06 2.05 2.04 2.03 2.02 2.01 2.01 2.00 1.99 1.98 1.96 1.93 1.92 1.88 1.88 1.87 1.86 1.85 1.84 1.84 1.82 1.82 1.81 1.78 1.77 1.76 1.75 1.74 1.74 1.73 1.73 1.72 1.71 1.65 1.65 1.65 1.64 1.63 1.63 1.62 1.62 1.61 1.61 1.60 1.59 1.58 1.56 1.56 1.54 1.53 1.52 1.50
O
Me
O
2.13
1.00
2.42
δ (ppm)
1.00 2.93 1.17 3.10 1.00 1.96 2.19 2.03 2.18 1.23
δ (ppm)

28

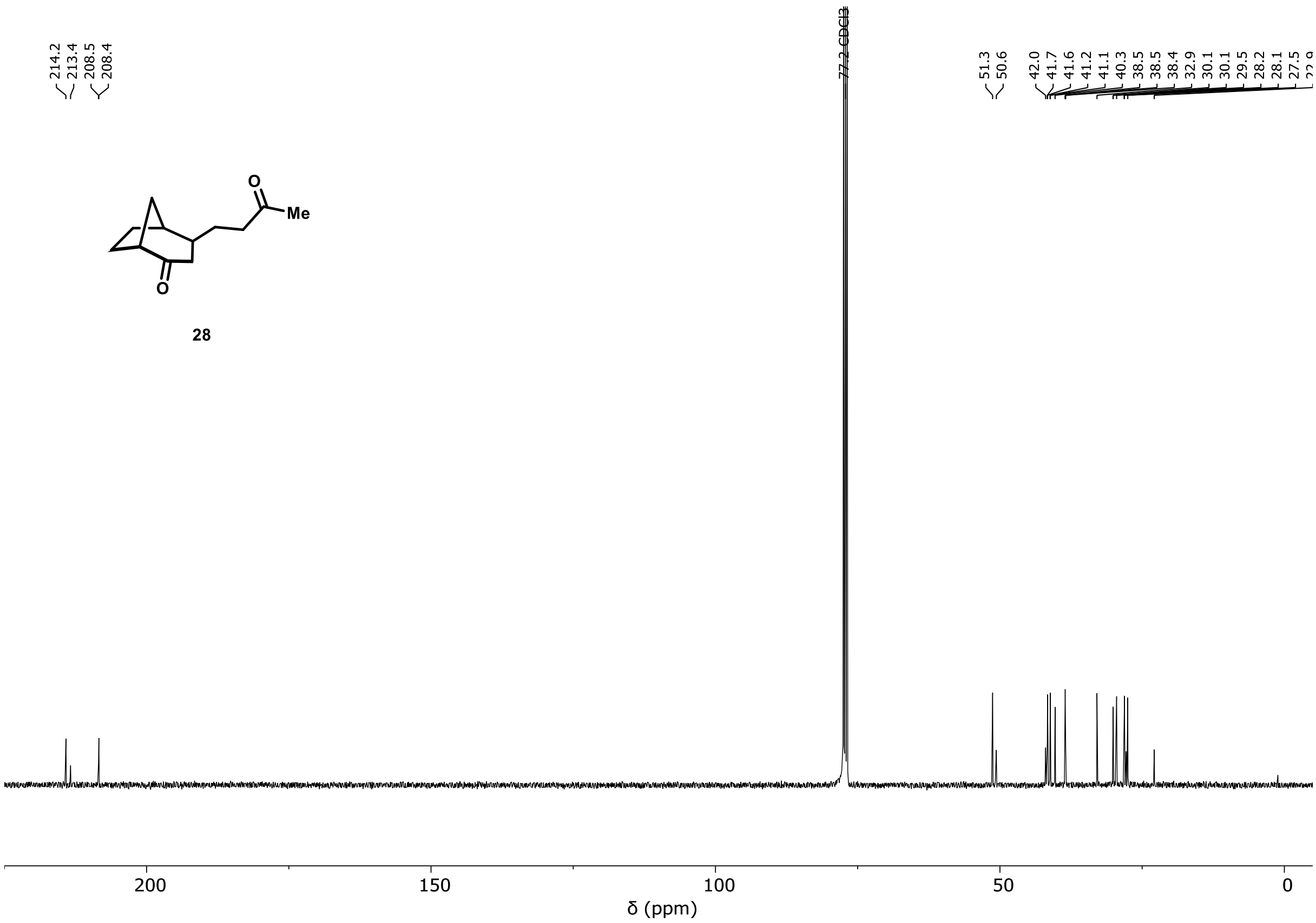
214.2 213.4 208.5 208.4
77.2 CDCl3
51.3 50.6
42.0 41.7 41.6 41.2 41.1 40.3 38.5 38.4 32.9 30.1 30.1 29.5 28.2 28.1 27.5
O
Me
O
δ (ppm)

28

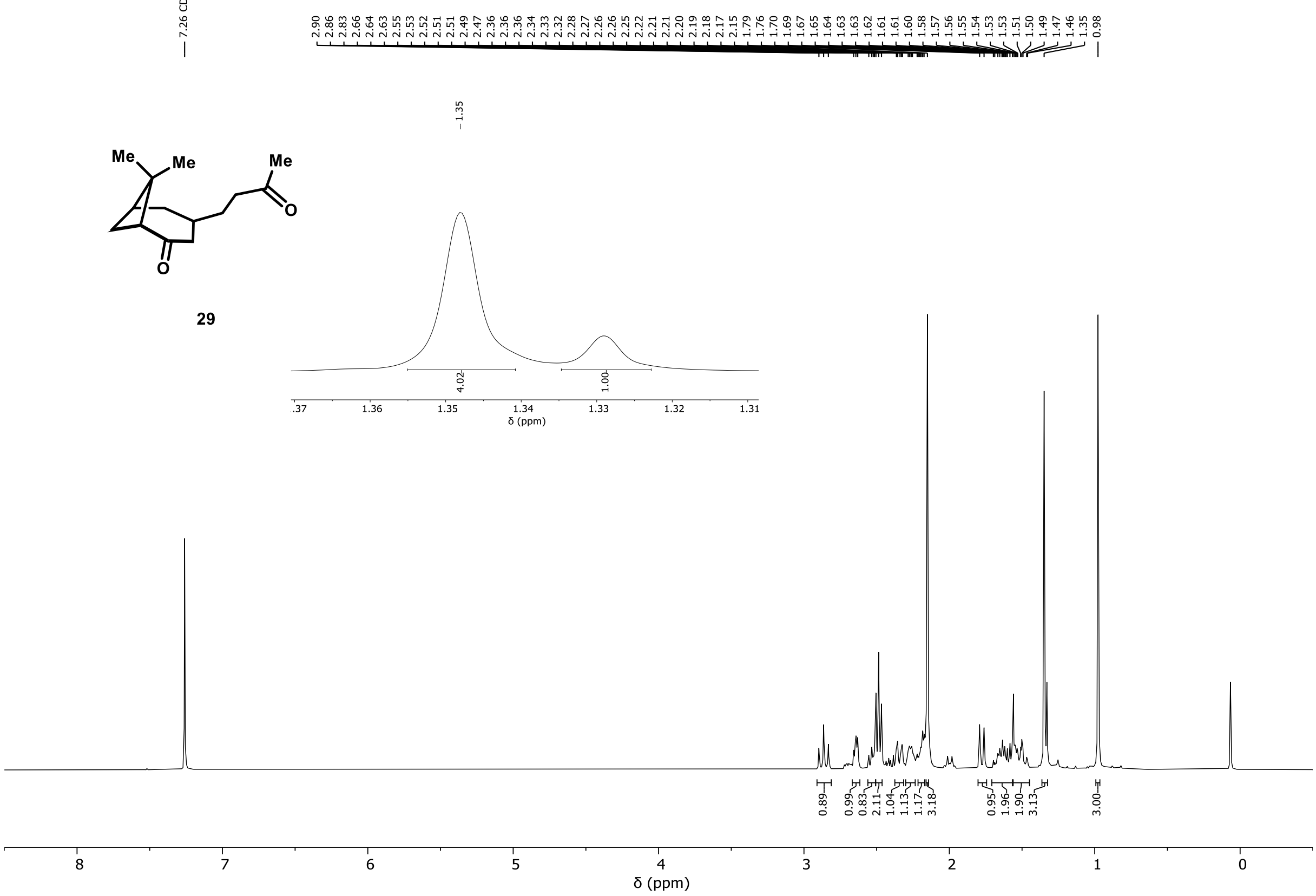
7.26 CDCl3
2.90 2.86 2.83 2.66 2.64 2.63 2.55 2.53 2.52 2.51 2.49 2.47 2.36 2.36 2.36 2.34 2.33 2.32 2.28 2.27 2.26 2.26 2.25 2.22 2.21 2.21 2.20 2.19 2.18 2.17 2.15 1.79 1.76 1.70 1.69 1.67 1.65 1.64 1.63 1.63 1.62 1.61 1.61 1.60 1.58 1.57 1.56 1.55 1.54 1.53 1.53 1.51 1.50 1.49 1.47 1.46 1.35 0.98
Me
Me
Me
O
O
1.35
4.02
1.00
δ (ppm)
0.89
0.99
0.83
2.11
1.04
1.13
1.17
3.18
0.95
1.96
1.90
3.13
3.00
δ (ppm)

29

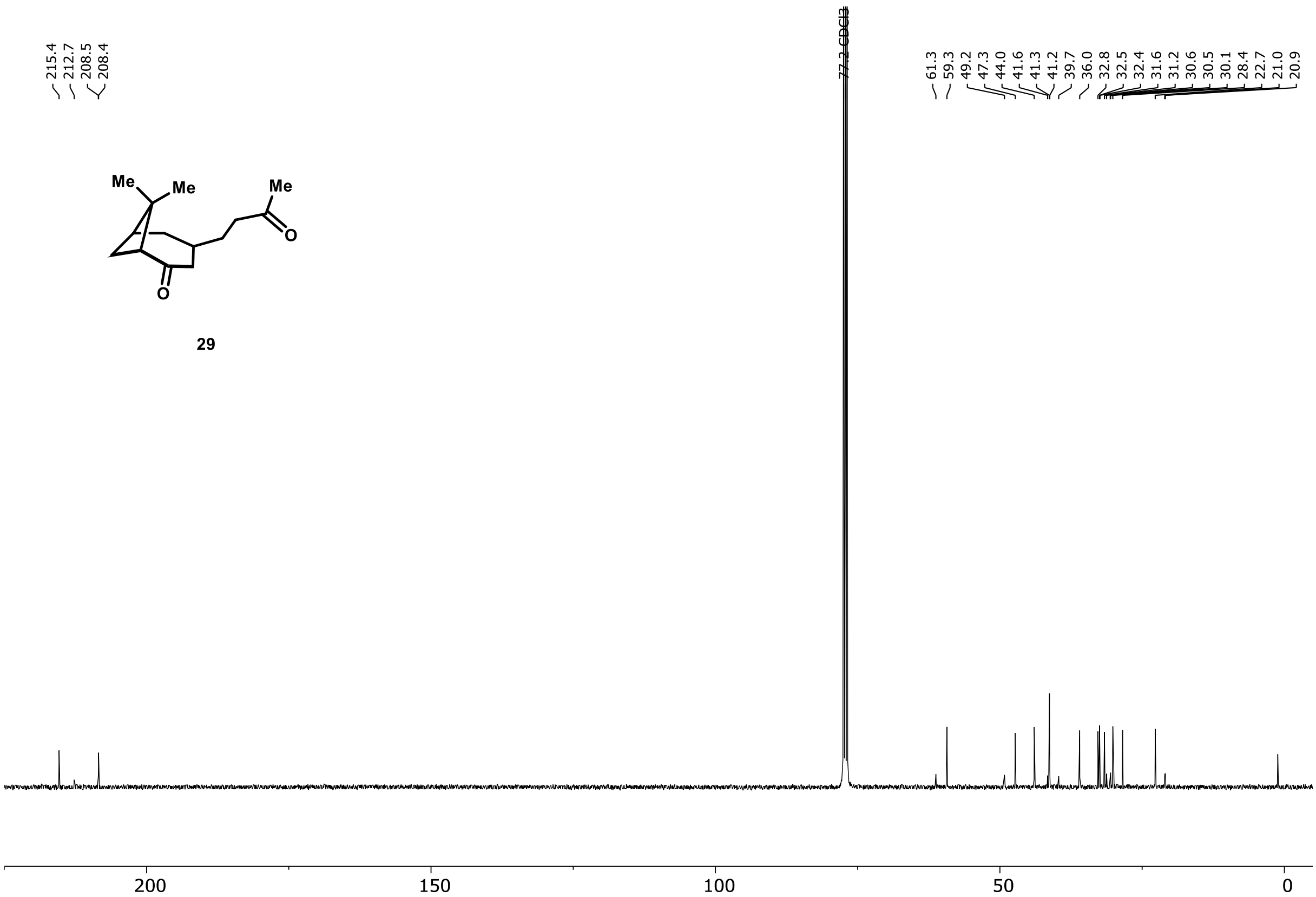
215.4
212.7
208.5
208.4
77.2 CDCl3
61.3
59.3
49.2
47.3
44.0
41.6
41.3
41.2
39.7
36.0
32.8
32.5
32.4
31.6
31.2
30.6
30.5
30.1
28.4
22.7
21.0
20.9
Me
Me
Me
O
O
δ (ppm)

29

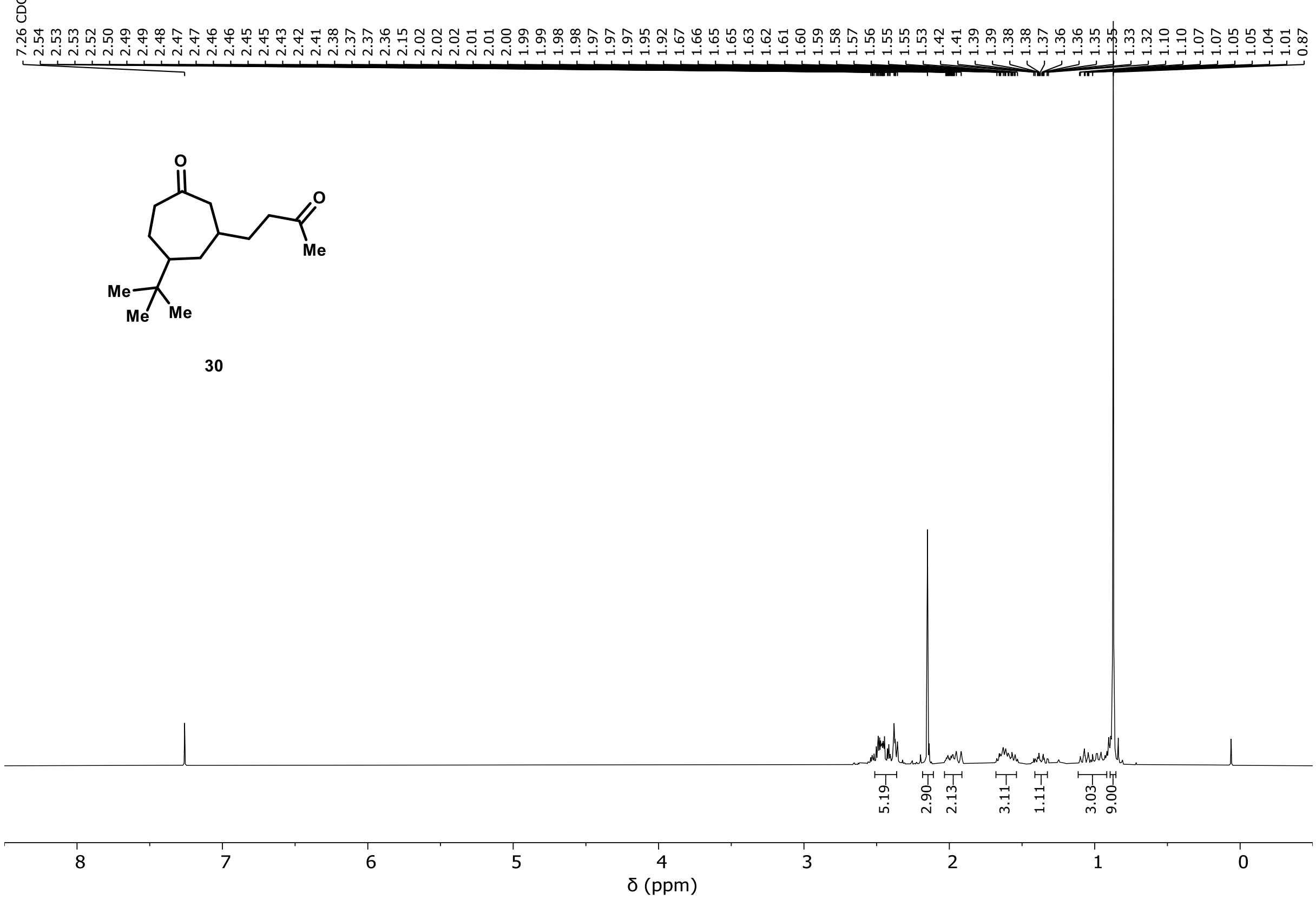

30

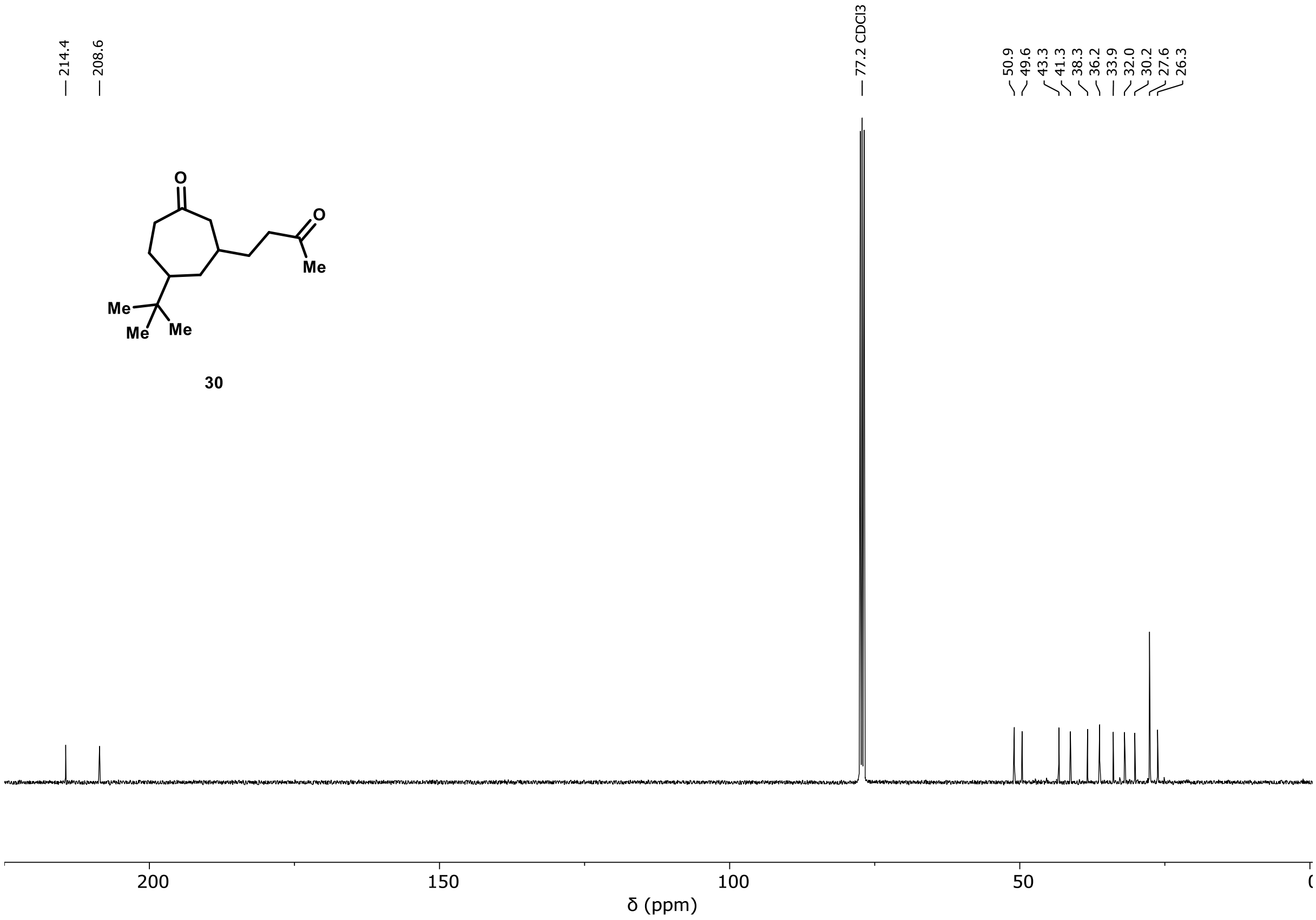

30

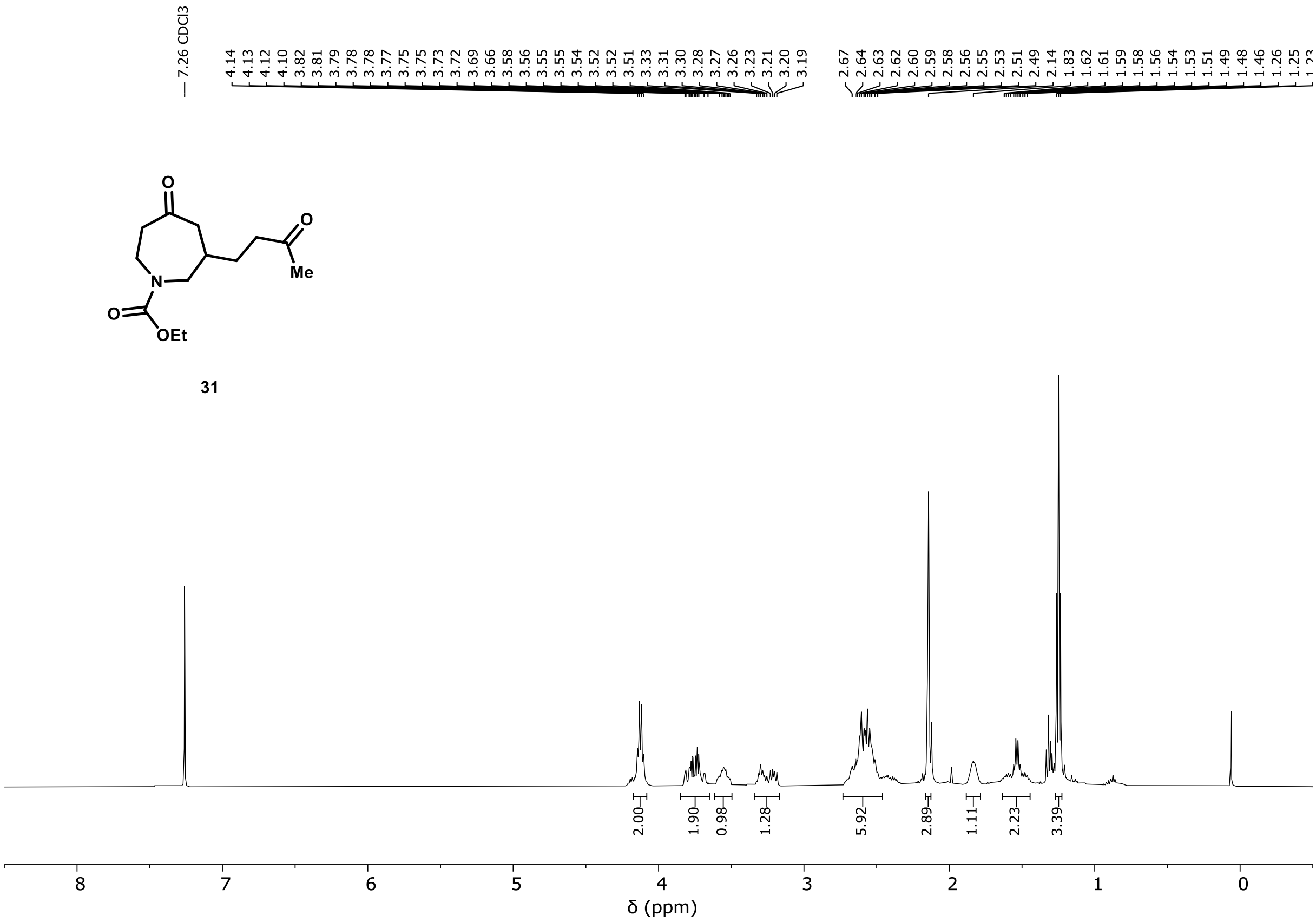
O
O
Me
N
O
OEt
31
7.26 CDCl3
4.14 4.13 4.12 4.10 3.82 3.81 3.79 3.78 3.78 3.77 3.75 3.75 3.73 3.72 3.69 3.66 3.58 3.56 3.55 3.55 3.54 3.52 3.52 3.51 3.33 3.31 3.30 3.28 3.27 3.26 3.23 3.21 3.20 3.19
2.67 2.64 2.63 2.62 2.60 2.59 2.58 2.56 2.55 2.53 2.51 2.49 2.14 1.83 1.62 1.61 1.59 1.58 1.56 1.54 1.53 1.51 1.49 1.48 1.46 1.26 1.25
2.00 1.90 0.98 1.28 5.92 2.89 1.11 2.23 3.39
8 7 6 5 4 3 2 1 0
δ (ppm)

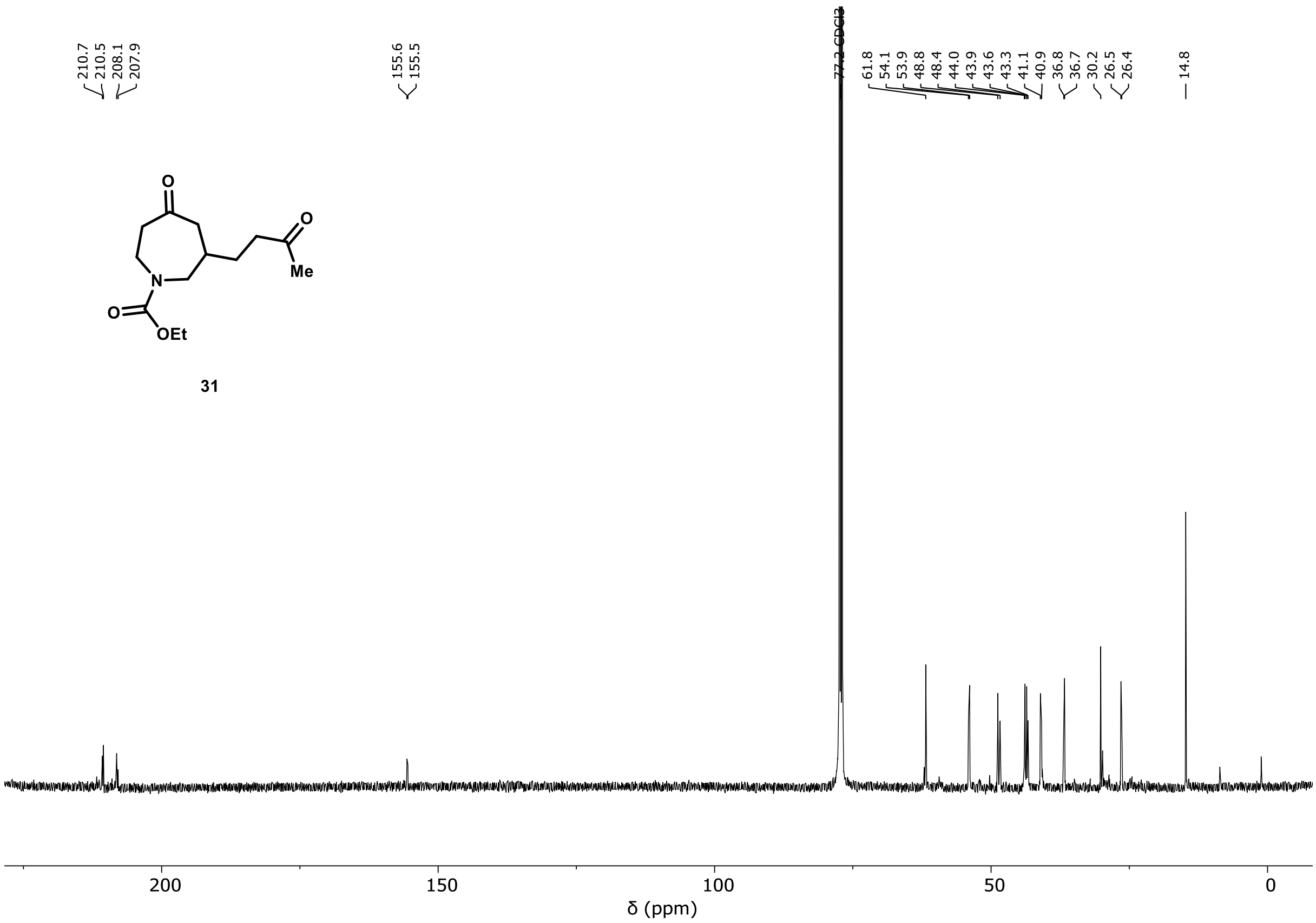
O
O
Me
N
O
OEt
31
210.7 210.5 208.1 207.9
155.6 155.5
61.8 54.1 53.9 48.8 48.4 44.0 43.9 43.6 43.3 41.1 40.9 36.8 36.7 30.2 26.5 26.4
14.8
200 150 100 50 0
δ (ppm)

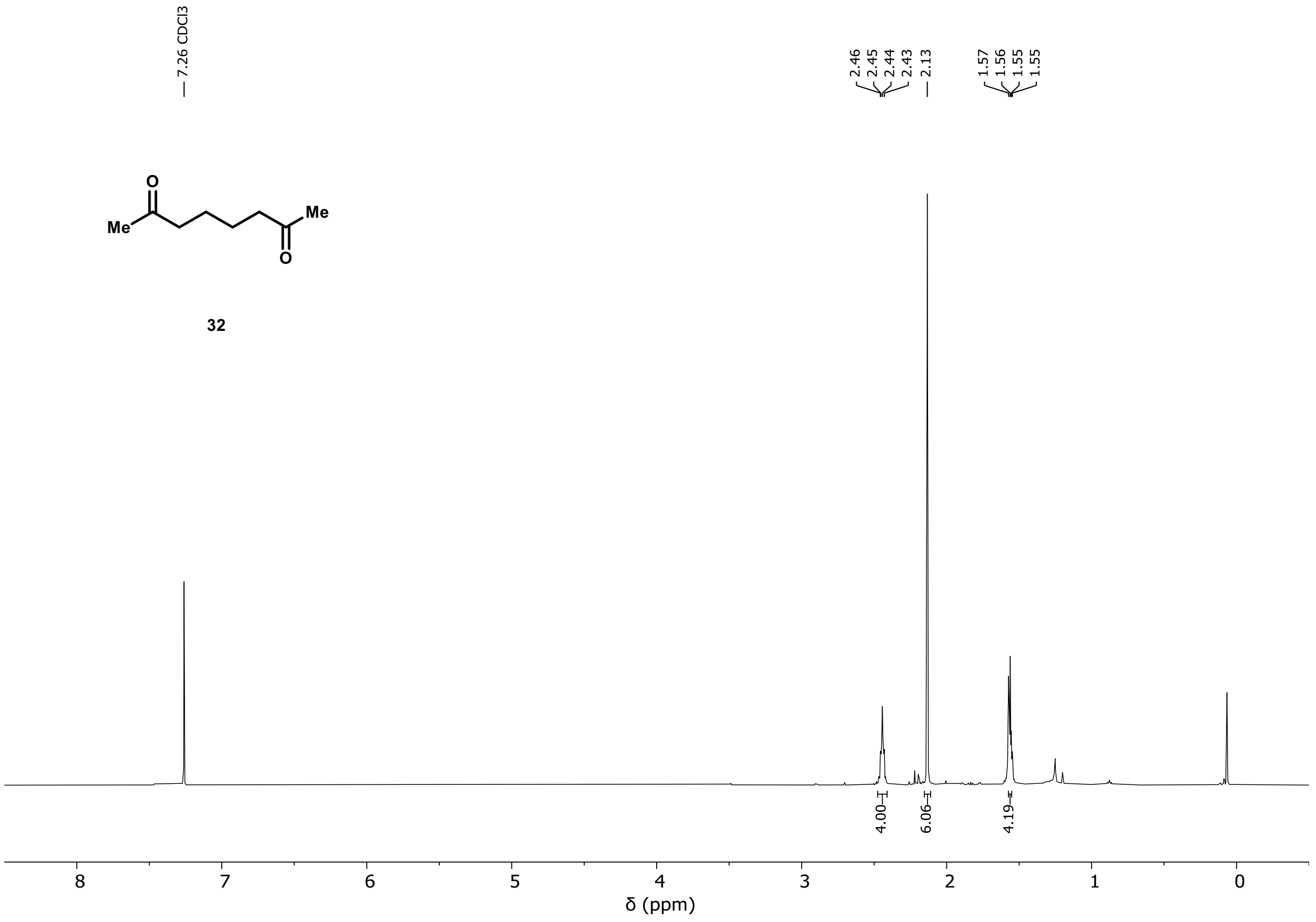
7.26 CDCl3
2.46
2.45
2.44
2.43
2.13
1.57
1.56
1.55
1.55
Me
O
Me
O
32
4.00
6.06
4.19
8
7
6
5
4
3
2
1
0
δ (ppm)

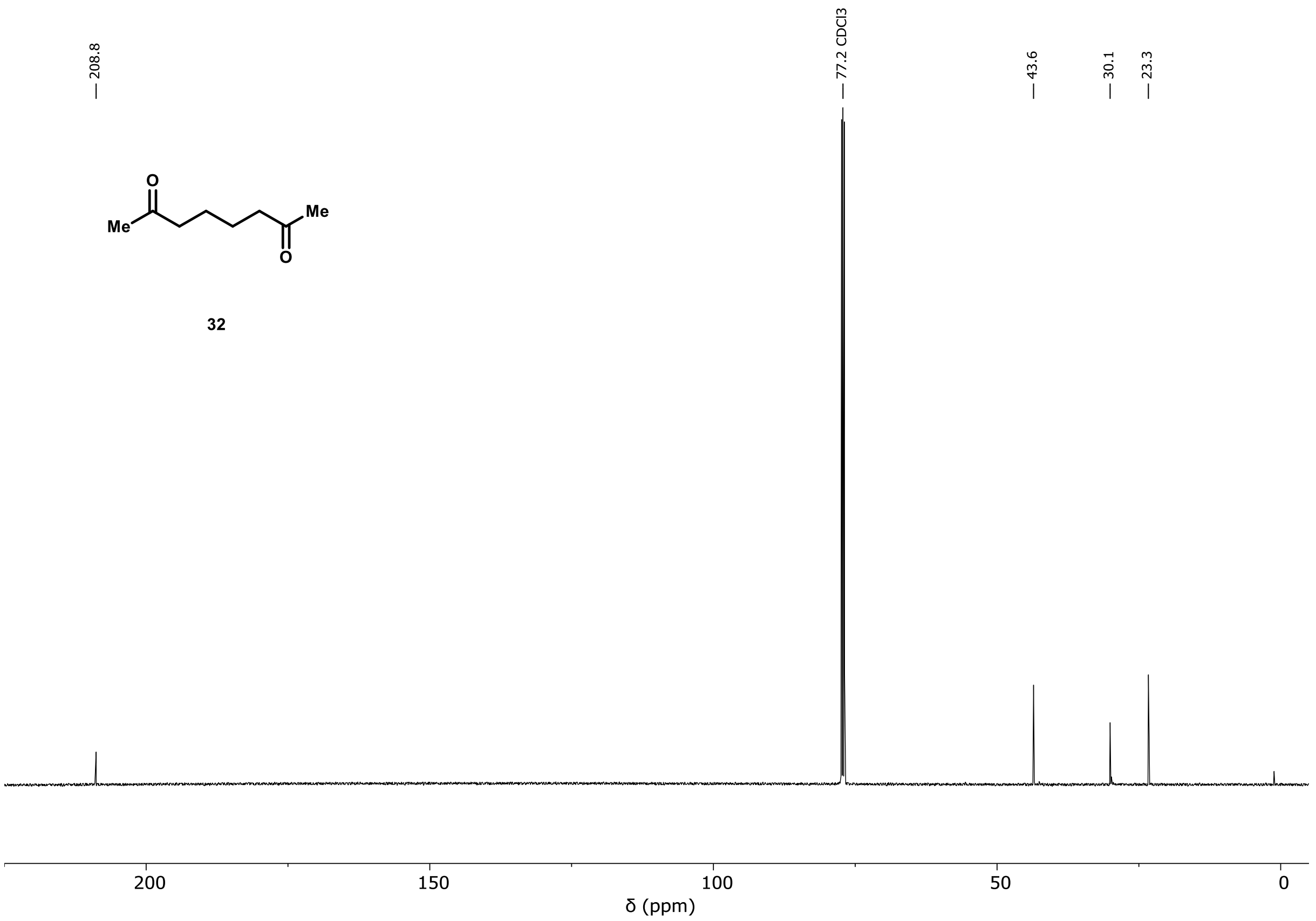
208.8
77.2 CDCl3
43.6
30.1
23.3
Me
O
Me
O
32
200
150
100
50
0
δ (ppm)

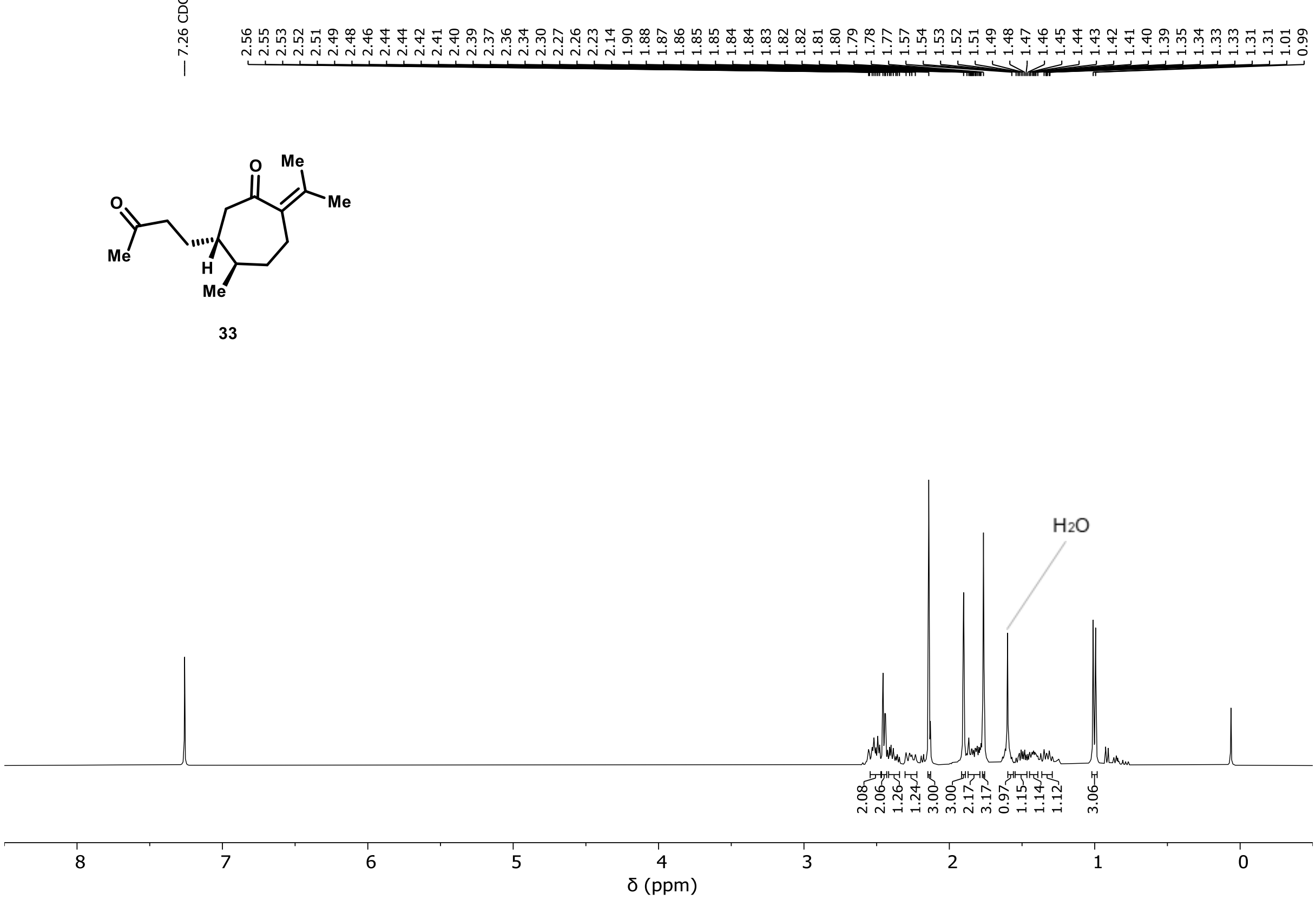

7.26 CDCl3
2.56
2.55
2.53
2.52
2.51
2.49
2.48
2.46
2.44
2.44
2.42
2.41
2.40
2.39
2.37
2.36
2.34
2.30
2.27
2.26
2.23
2.14
1.90
1.88
1.87
1.86
1.85
1.84
1.84
1.83
1.82
1.82
1.81
1.80
1.79
1.78
1.77
1.57
1.54
1.53
1.52
1.51
1.49
1.48
1.47
1.46
1.45
1.45
1.44
1.43
1.42
1.41
1.40
1.39
1.35
1.34
1.33
1.33
1.31
1.31
1.01
0.99
O
Me
Me
O
Me
H
Me
33
H2O
2.08
2.06
1.26
1.24
3.00
3.00
2.17
3.17
0.97
1.15
1.14
1.12
3.06
8
7
6
5
4
3
2
1
0
δ (ppm)

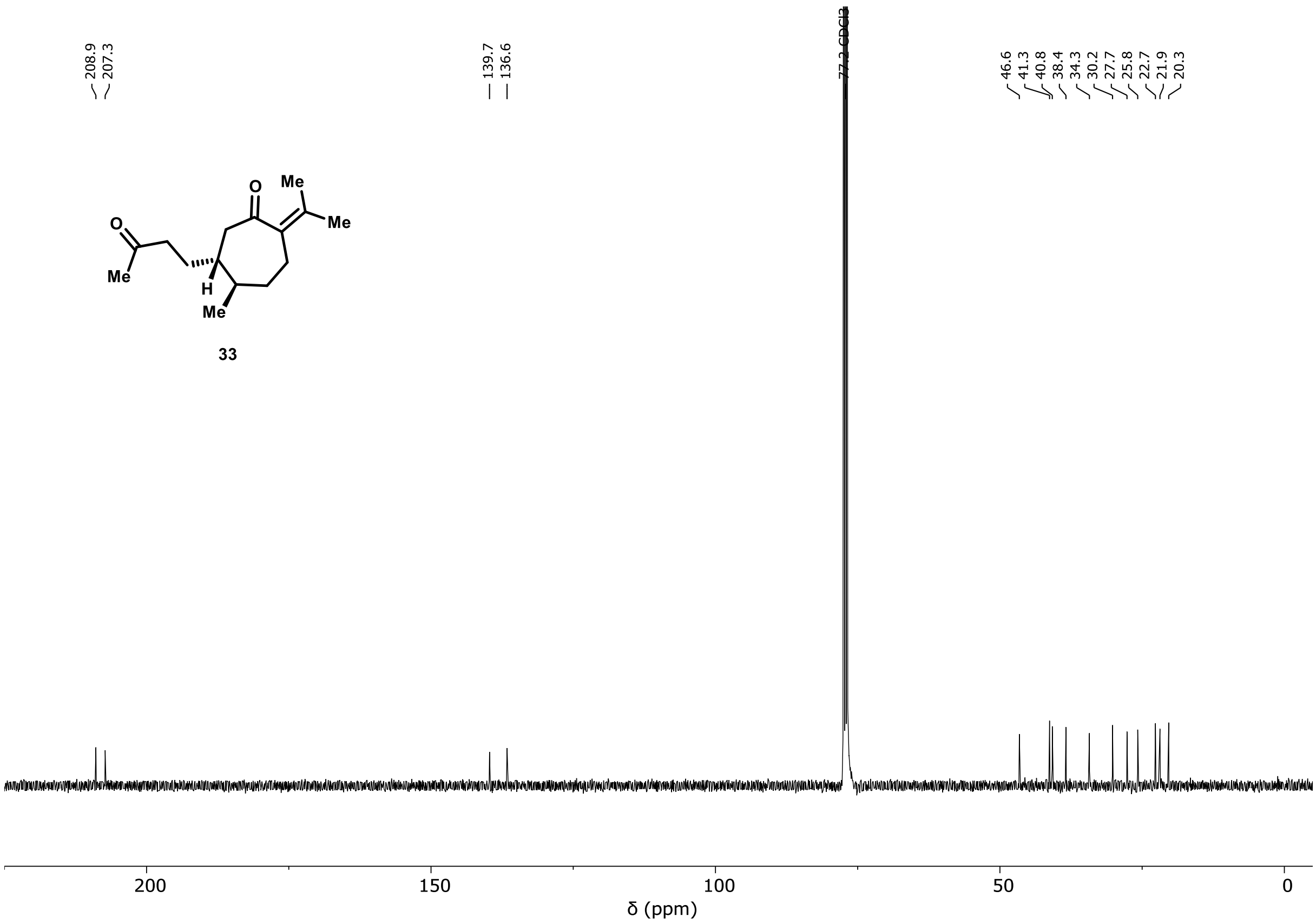

208.9
207.3
139.7
136.6
77.2 CDCl3
46.6
41.3
40.8
38.4
34.3
30.2
27.7
25.8
22.7
21.9
20.3
O
Me
Me
O
Me
H
Me
33
200
150
100
50
0
δ (ppm)

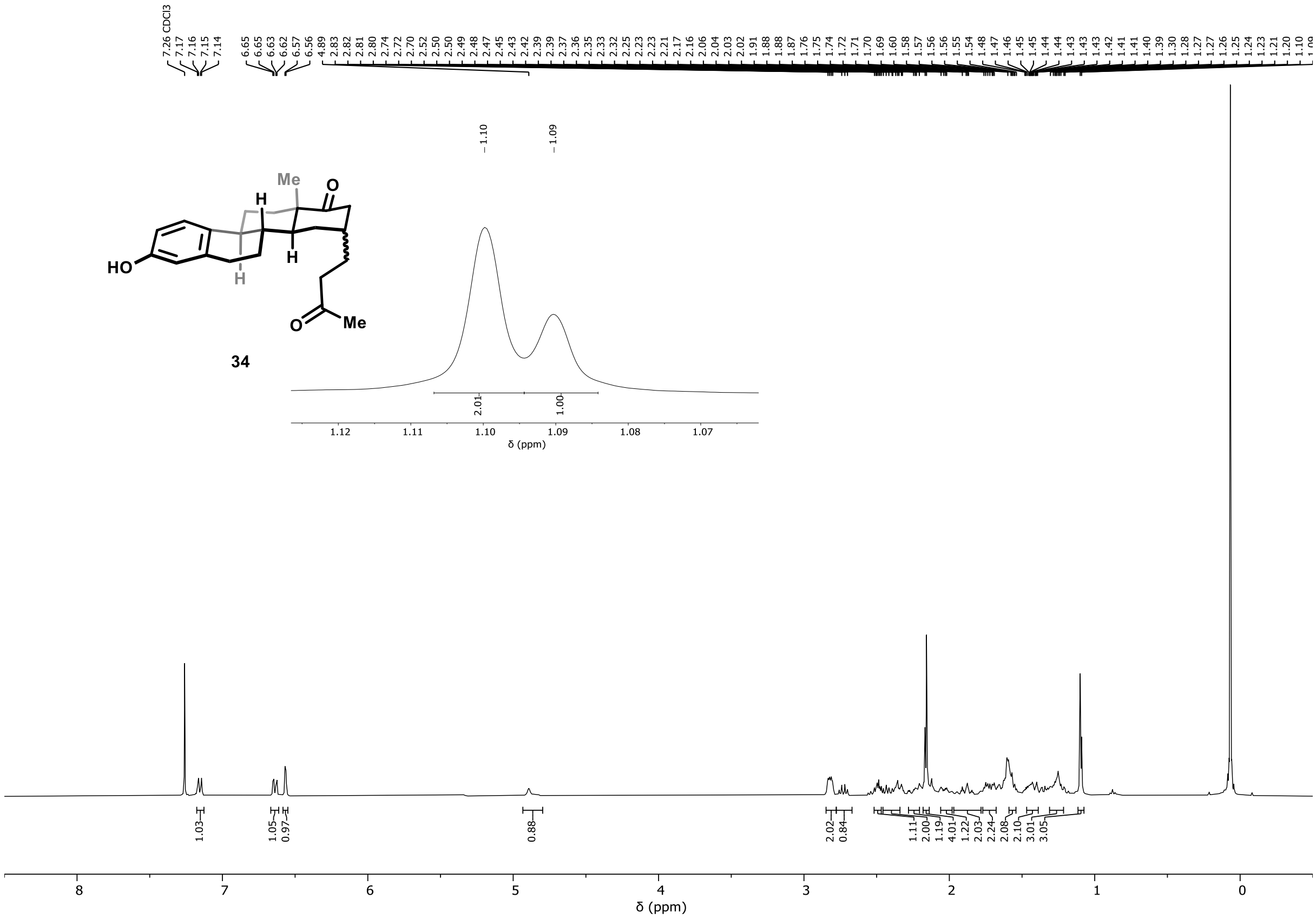
Me
O
H
HO
H
H
Me
O
34
δ (ppm)
δ (ppm)

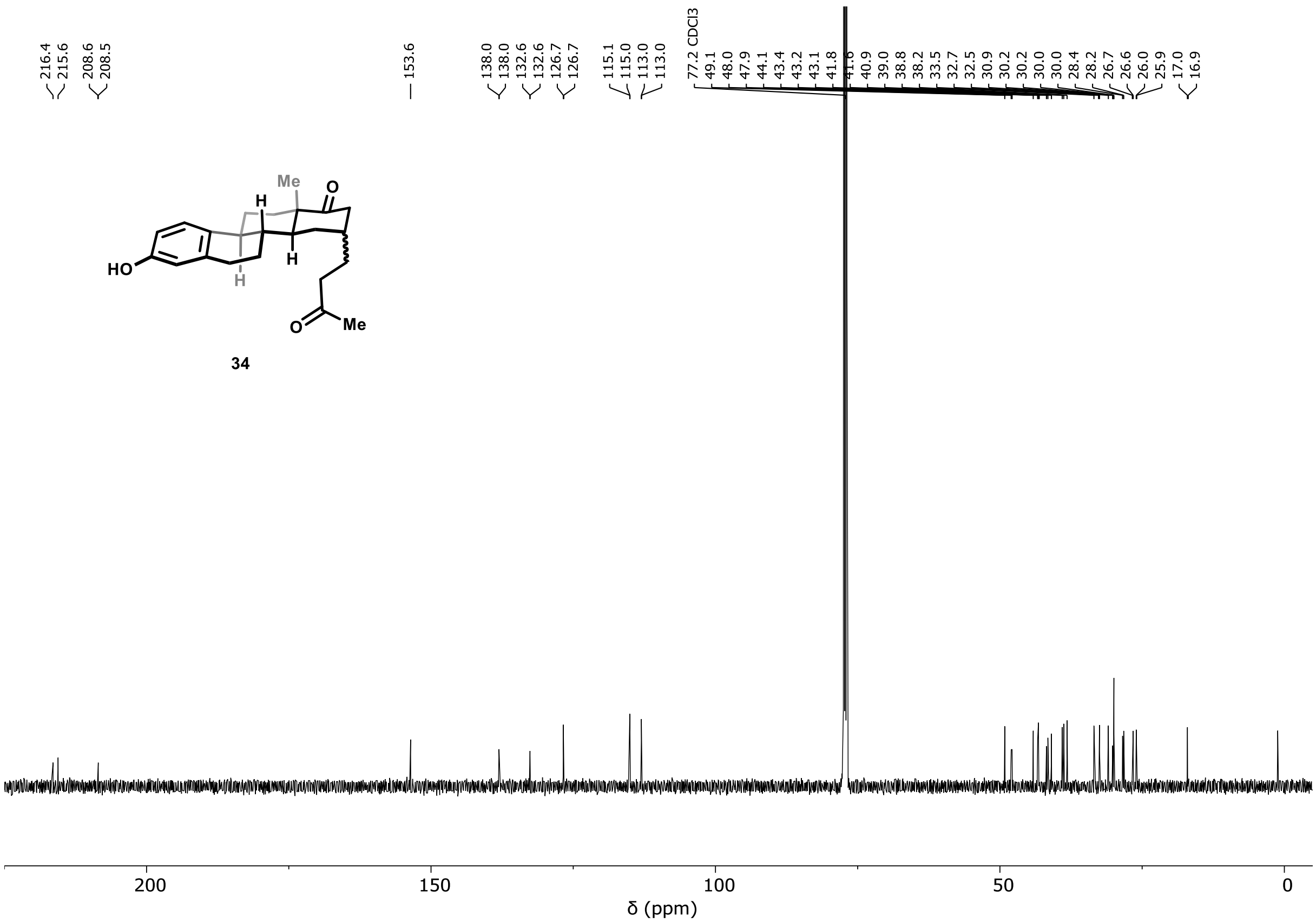
216.4
215.6
208.6
208.5
153.6
138.0
138.0
132.6
132.6
126.7
126.7
115.1
115.0
113.0
113.0
77.2 CDCl3
Me
O
H
HO
H
H
Me
O
34
δ (ppm)

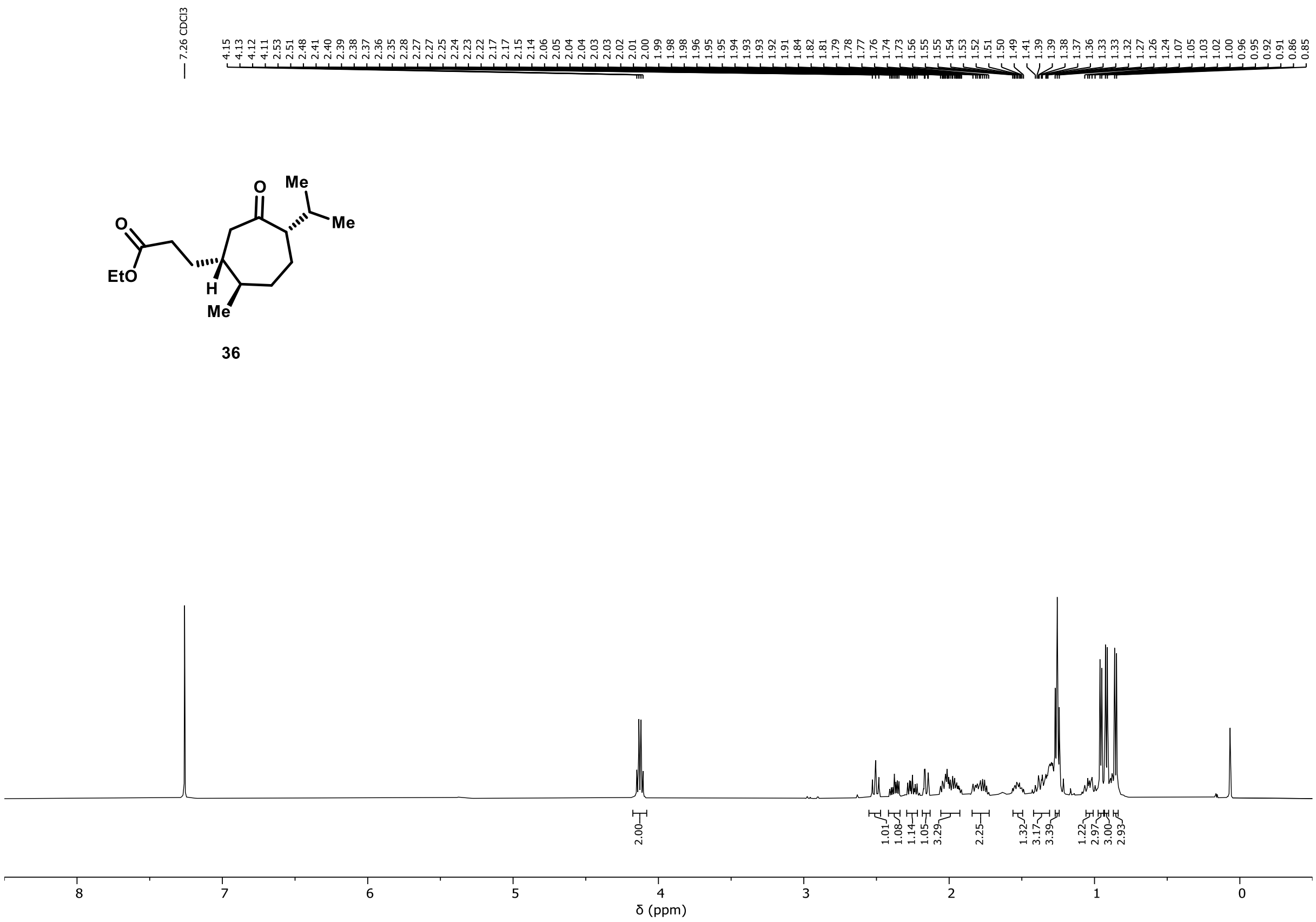
7.26 CDCl3
4.15
4.13
4.12
4.11
2.53
2.51
2.48
2.41
2.40
2.39
2.38
2.37
2.36
2.35
2.28
2.27
2.27
2.25
2.24
2.23
2.22
2.17
2.17
2.15
2.14
2.06
2.05
2.04
2.04
2.03
2.03
2.02
2.01
2.00
1.99
1.98
1.98
1.96
1.95
1.95
1.94
1.93
1.93
1.92
1.91
1.84
1.82
1.81
1.79
1.78
1.77
1.76
1.74
1.73
1.56
1.55
1.55
1.54
1.53
1.52
1.51
1.50
1.49
1.41
1.39
1.39
1.38
1.37
1.36
1.33
1.33
1.32
1.27
1.26
1.24
1.07
1.05
1.03
1.02
1.00
0.96
0.95
0.92
0.91
0.86
0.85
O
Me
Me
O
EtO
H
Me
36
2.00
1.01
1.08
1.14
1.05
3.29
2.25
1.32
3.17
3.39
1.22
2.97
3.00
2.93
8
7
6
5
4
3
2
1
0
δ (ppm)

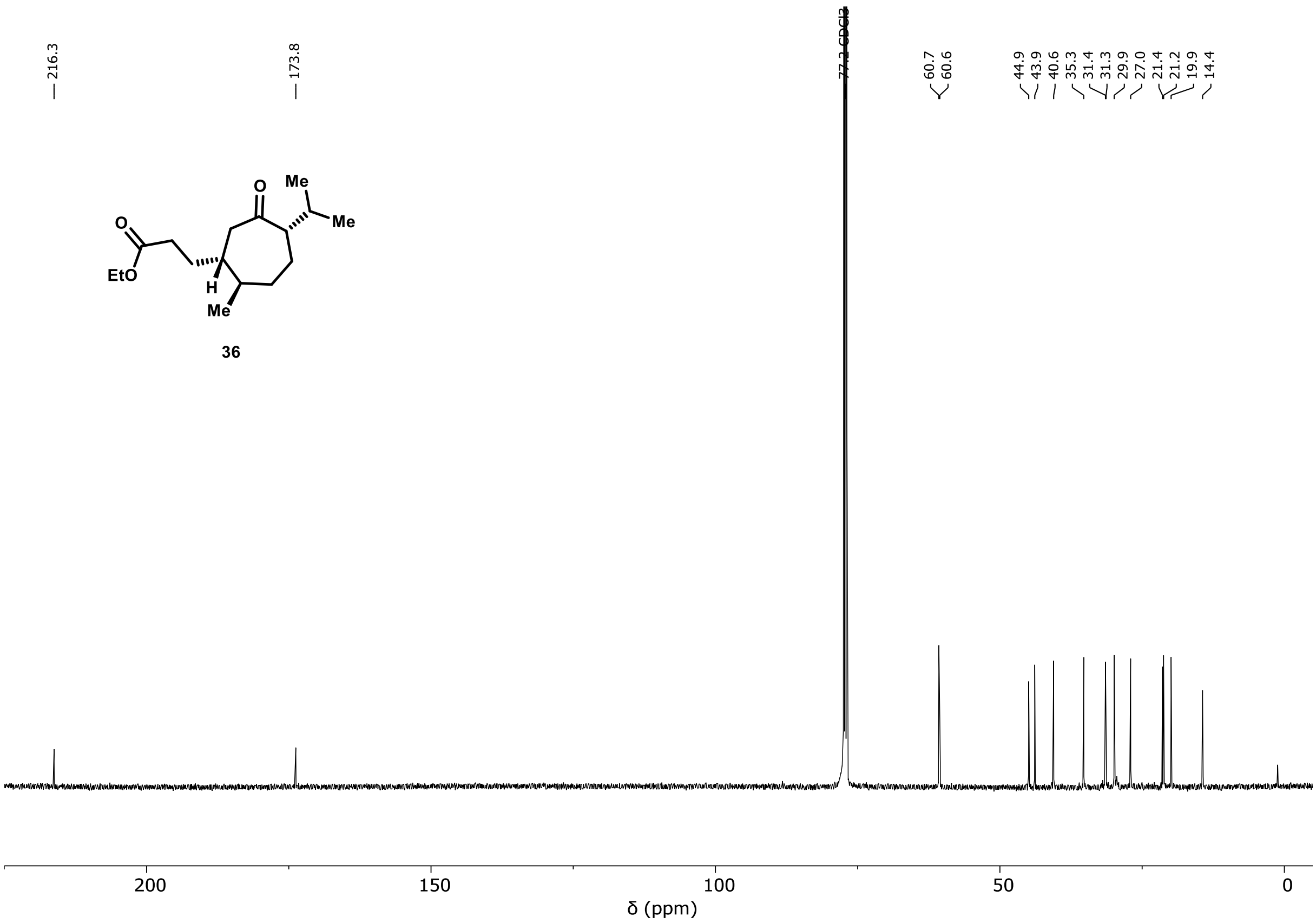
216.3
173.8
77.2 CDCl3
60.7
60.6
44.9
43.9
40.6
35.3
31.4
31.3
29.9
27.0
21.4
21.2
19.9
14.4
O
Me
Me
O
EtO
H
Me
36
200
150
100
50
0
δ (ppm)

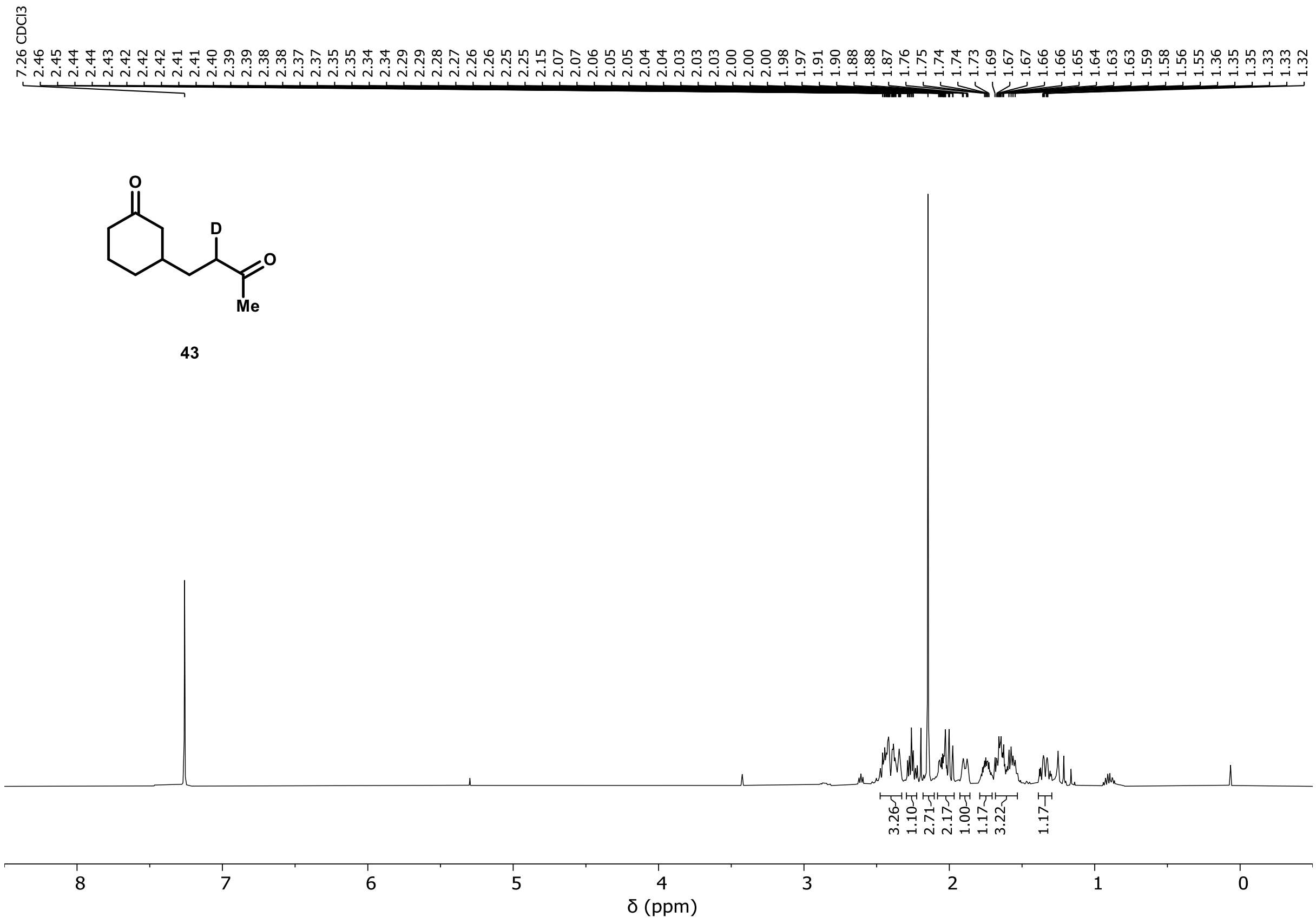
7.26 CDCl3
2.46 2.45 2.44 2.44 2.43 2.42 2.42 2.42 2.41 2.41 2.40 2.39 2.39 2.38 2.38 2.37 2.37 2.35 2.35 2.34 2.34 2.29 2.29 2.28 2.27 2.26 2.26 2.25 2.25 2.15 2.07 2.07 2.06 2.05 2.05 2.04 2.04 2.03 2.03 2.03 2.00 2.00 1.98 1.97 1.91 1.90 1.88 1.88 1.87 1.76 1.75 1.74 1.74 1.73 1.69 1.67 1.67 1.66 1.66 1.65 1.64 1.63 1.63 1.59 1.58 1.56 1.55 1.36 1.35 1.35 1.33 1.33 1.32
O
D
O
Me
43
3.26
1.10
2.71
2.17
1.00
1.17
3.22
1.17
8 7 6 5 4 3 2 1 0
δ (ppm)

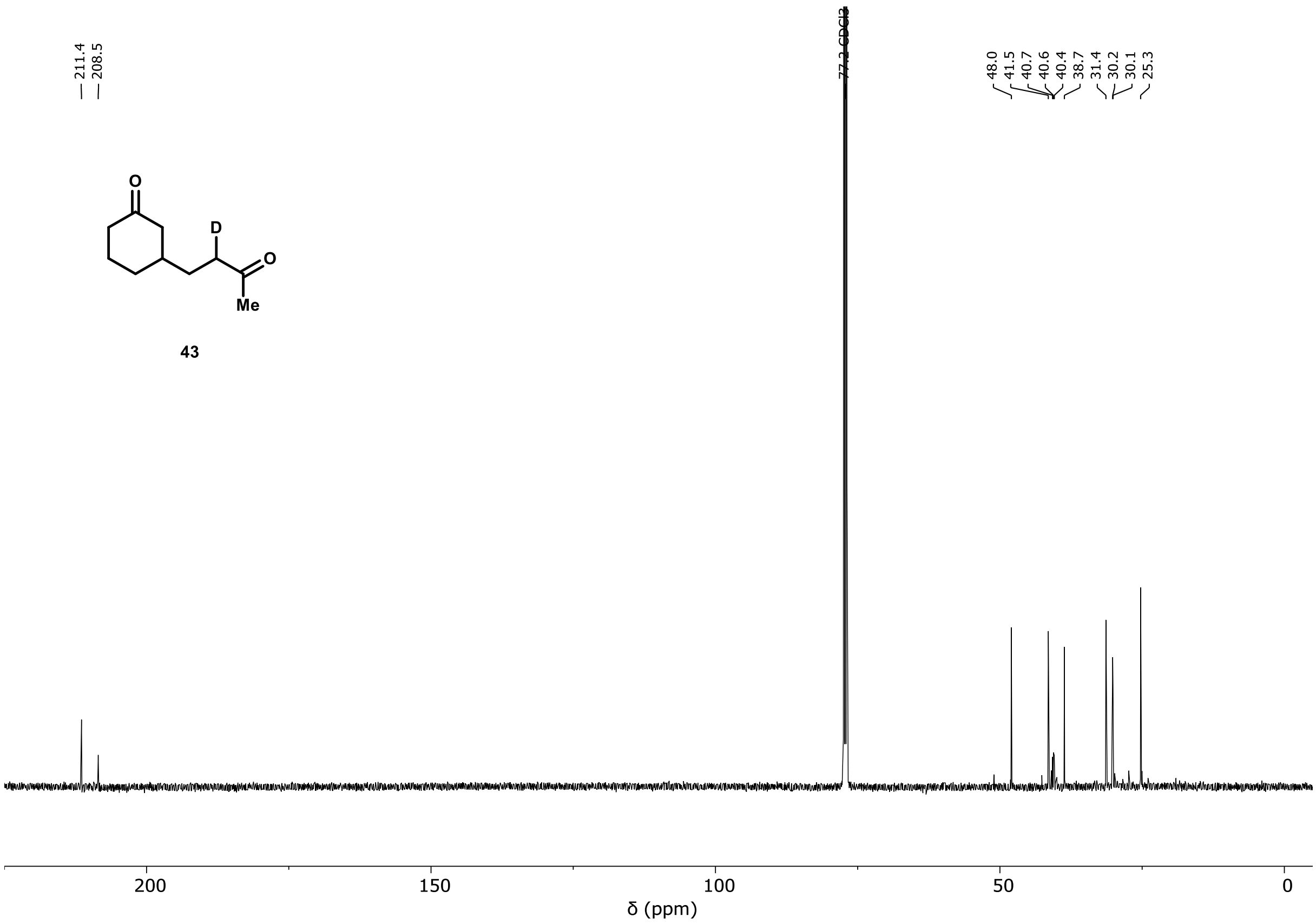
211.4
208.5
77.2 CDCl3
48.0 41.5 40.7 40.6 40.4 38.7 31.4 30.2 30.1 25.3
O
D
O
Me
43
200 150 100 50 0
δ (ppm)

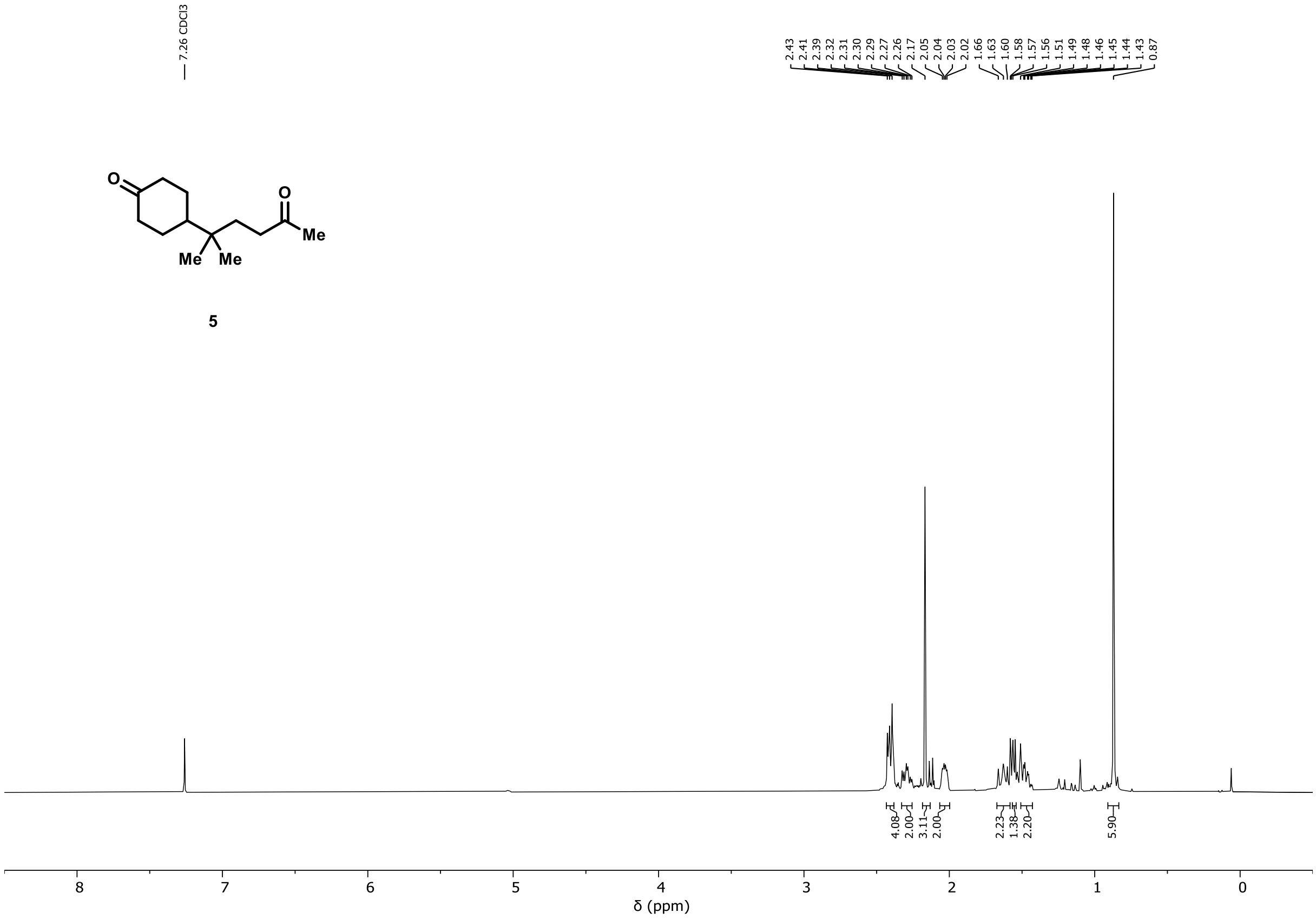
7.26 CDCl3
2.43
2.41
2.39
2.32
2.31
2.30
2.29
2.27
2.26
2.17
2.05
2.04
2.03
2.02
1.66
1.63
1.60
1.58
1.57
1.56
1.51
1.49
1.48
1.46
1.45
1.44
1.43
0.87
O
O
Me
Me
Me
5
4.08
2.00
3.11
2.00
2.23
1.38
2.20
5.90
8
7
6
5
4
3
2
1
0
δ (ppm)

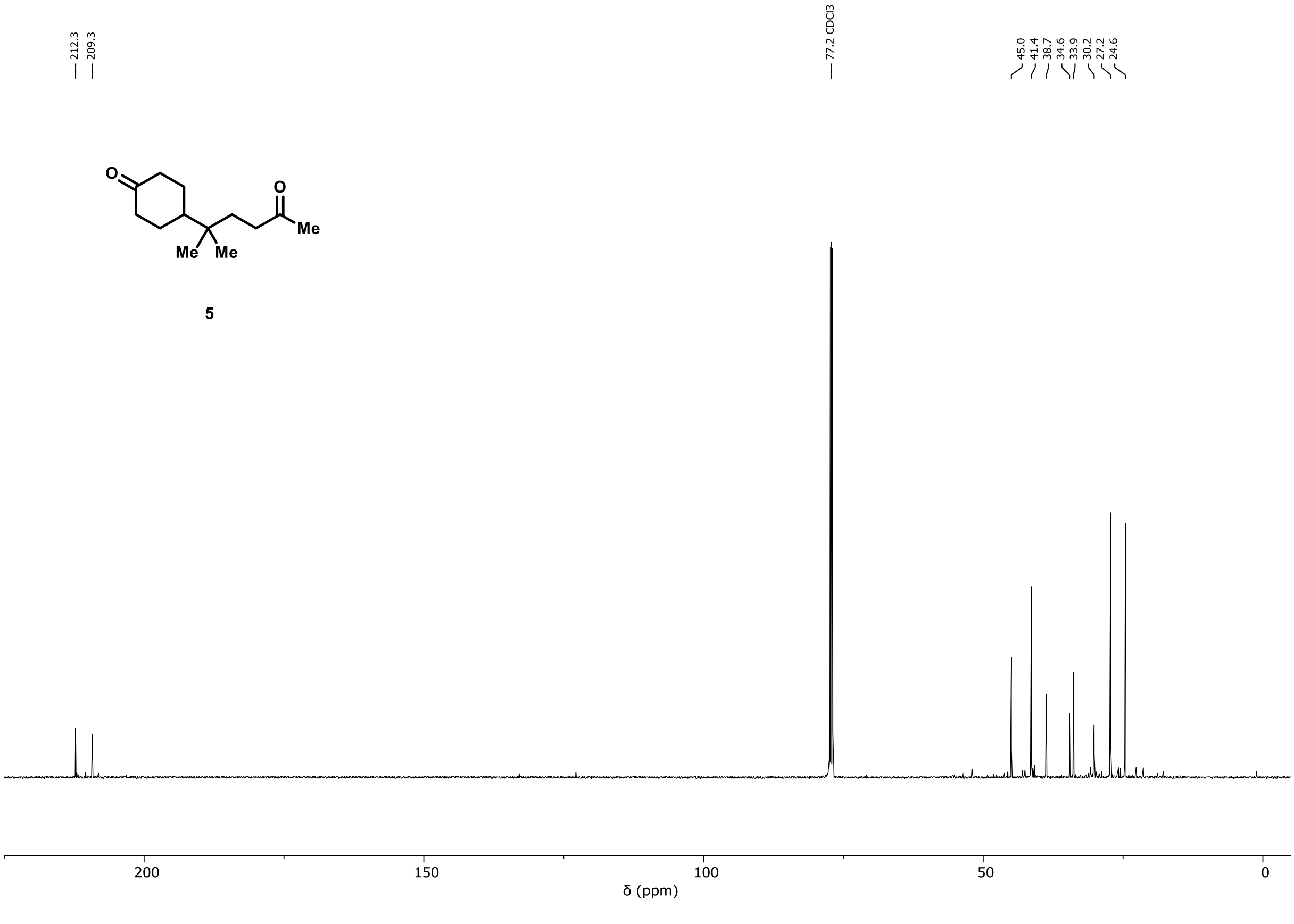
212.3
209.3
77.2 CDCl3
45.0
41.4
38.7
34.6
33.9
30.2
27.2
24.6
O
O
Me
Me
Me
5
200
150
100
50
0
δ (ppm)

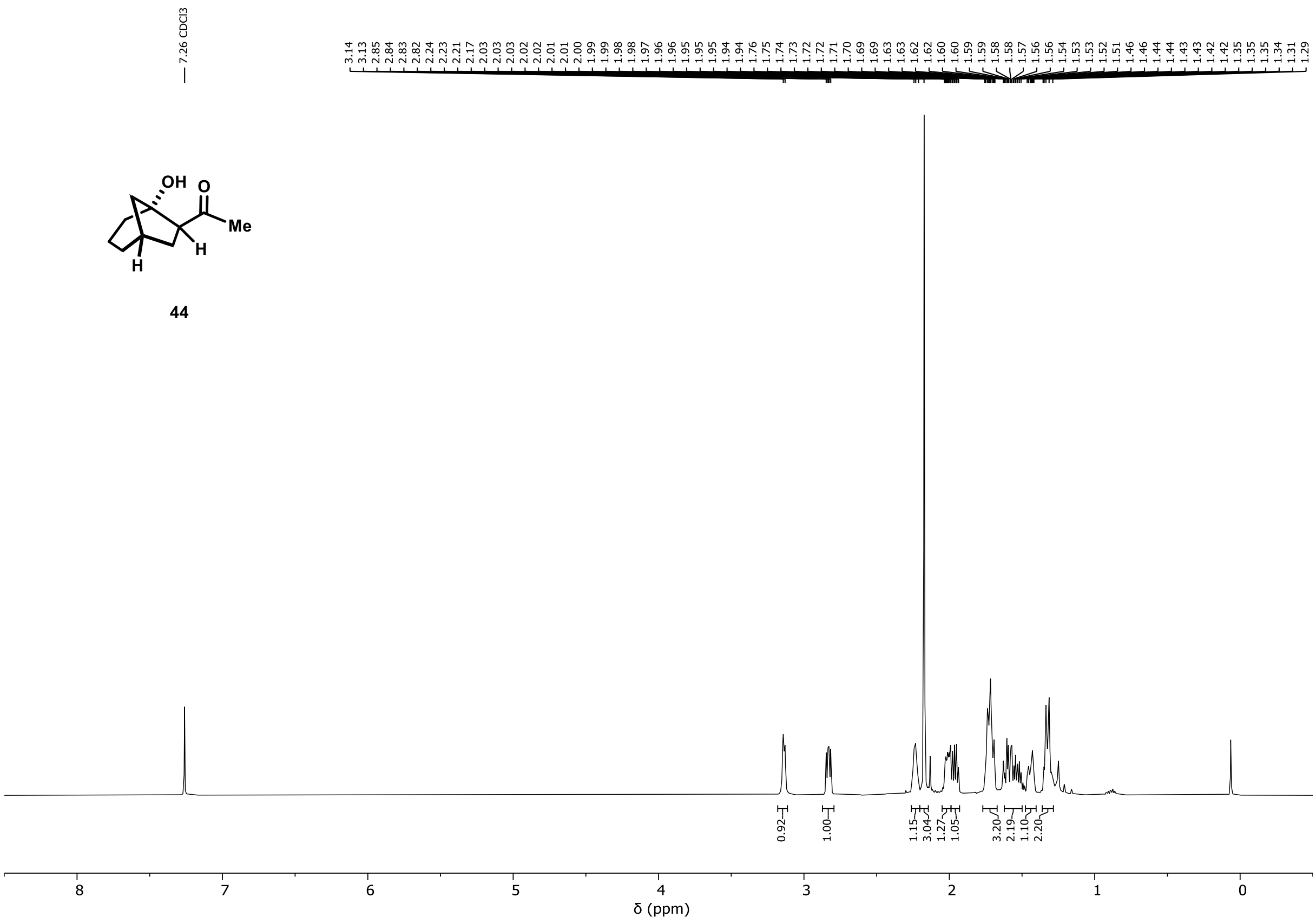
7.26 CDCl3
3.14 3.13 2.85 2.84 2.83 2.82 2.24 2.23 2.21 2.17 2.03 2.03 2.03 2.02 2.02 2.01 2.01 2.00 1.99 1.99 1.98 1.98 1.97 1.96 1.96 1.95 1.95 1.94 1.94 1.76 1.75 1.74 1.73 1.72 1.71 1.70 1.69 1.69 1.63 1.63 1.62 1.62 1.60 1.60 1.59 1.59 1.58 1.58 1.57 1.56 1.56 1.54 1.53 1.53 1.52 1.51 1.46 1.44 1.44 1.43 1.43 1.42 1.42 1.35 1.35 1.35 1.34 1.31 1.29
OH
O
Me
H
H
44
0.92
1.00
1.15
3.04
1.27
1.05
3.20
2.19
1.10
2.20
8
7
6
5
4
3
2
1
0
δ (ppm)

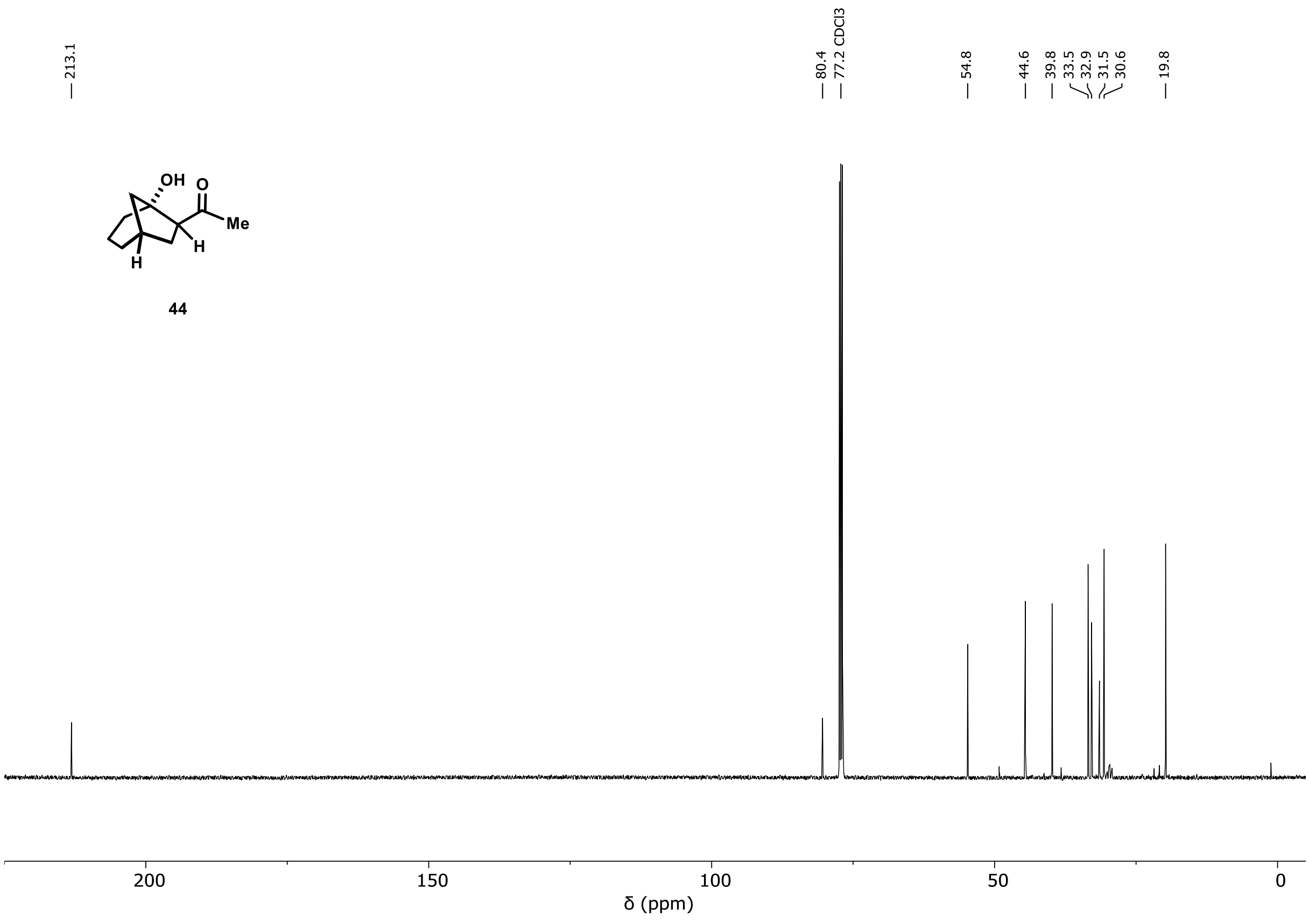
213.1
80.4
77.2 CDCl3
54.8
44.6
39.8
33.5
32.9
31.5
30.6
19.8
OH
O
Me
H
H
44
200
150
100
50
0
δ (ppm)

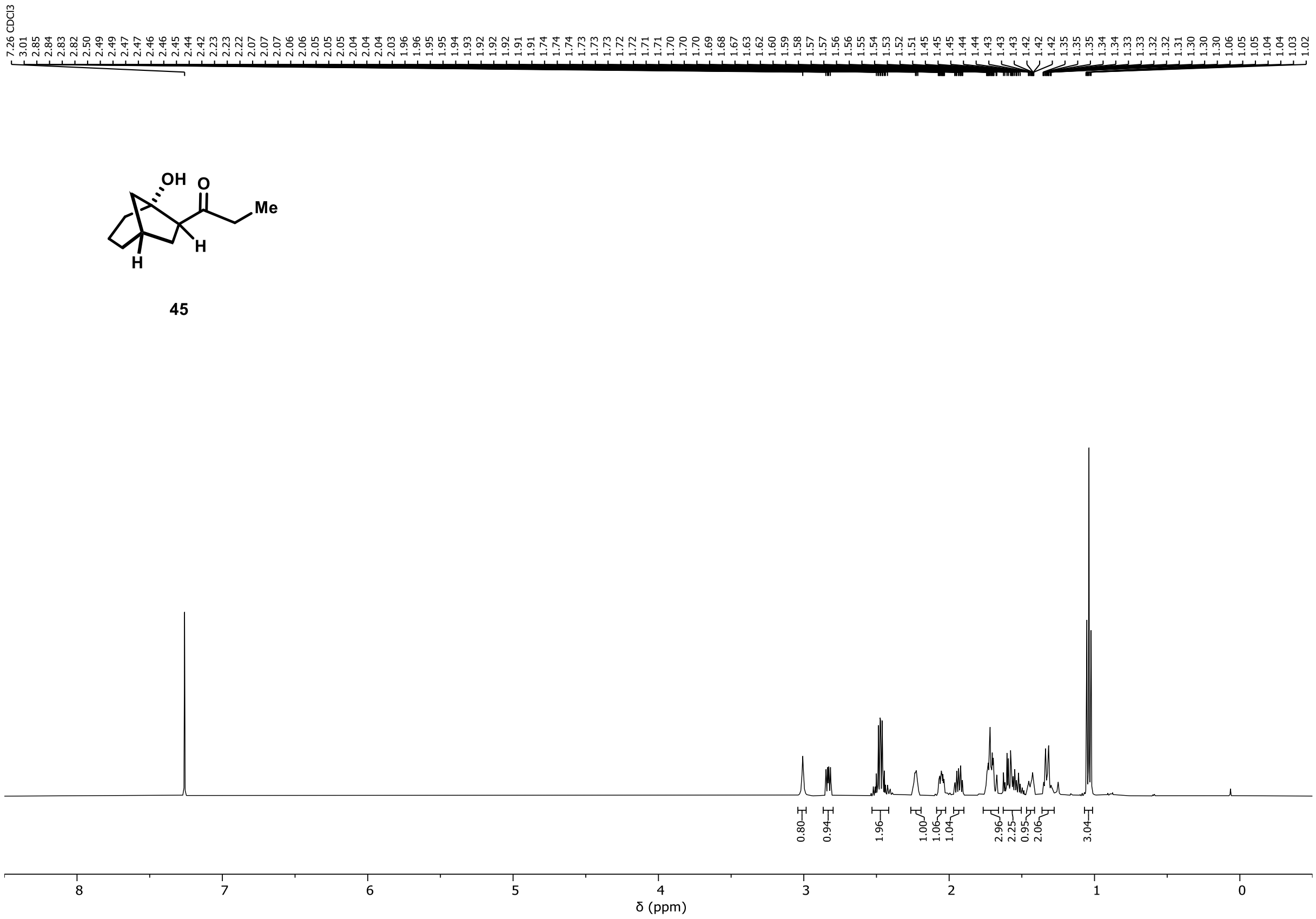
7.26 CDCl3
3.01
2.85
2.84
2.83
2.82
2.50
2.49
2.49
2.47
2.46
2.46
2.45
2.44
2.42
2.23
2.22
2.07
2.07
2.07
2.06
2.06
2.05
2.05
2.05
2.04
2.04
2.04
2.03
1.96
1.96
1.95
1.95
1.94
1.93
1.92
1.92
1.92
1.91
1.91
1.74
1.74
1.74
1.73
1.73
1.73
1.72
1.72
1.71
1.71
1.70
1.70
1.69
1.68
1.67
1.63
1.62
1.60
1.59
1.58
1.57
1.56
1.55
1.54
1.53
1.52
1.51
1.45
1.45
1.45
1.44
1.44
1.43
1.43
1.43
1.42
1.42
1.42
1.35
1.35
1.35
1.34
1.34
1.33
1.33
1.32
1.32
1.31
1.30
1.30
1.30
1.06
1.05
1.05
1.04
1.04
1.03
1.02
OH
O
Me
H
H
45
0.80
0.94
1.96
1.00
1.06
1.04
2.96
2.25
0.95
2.06
3.04
8
7
6
5
4
3
2
1
0
δ (ppm)

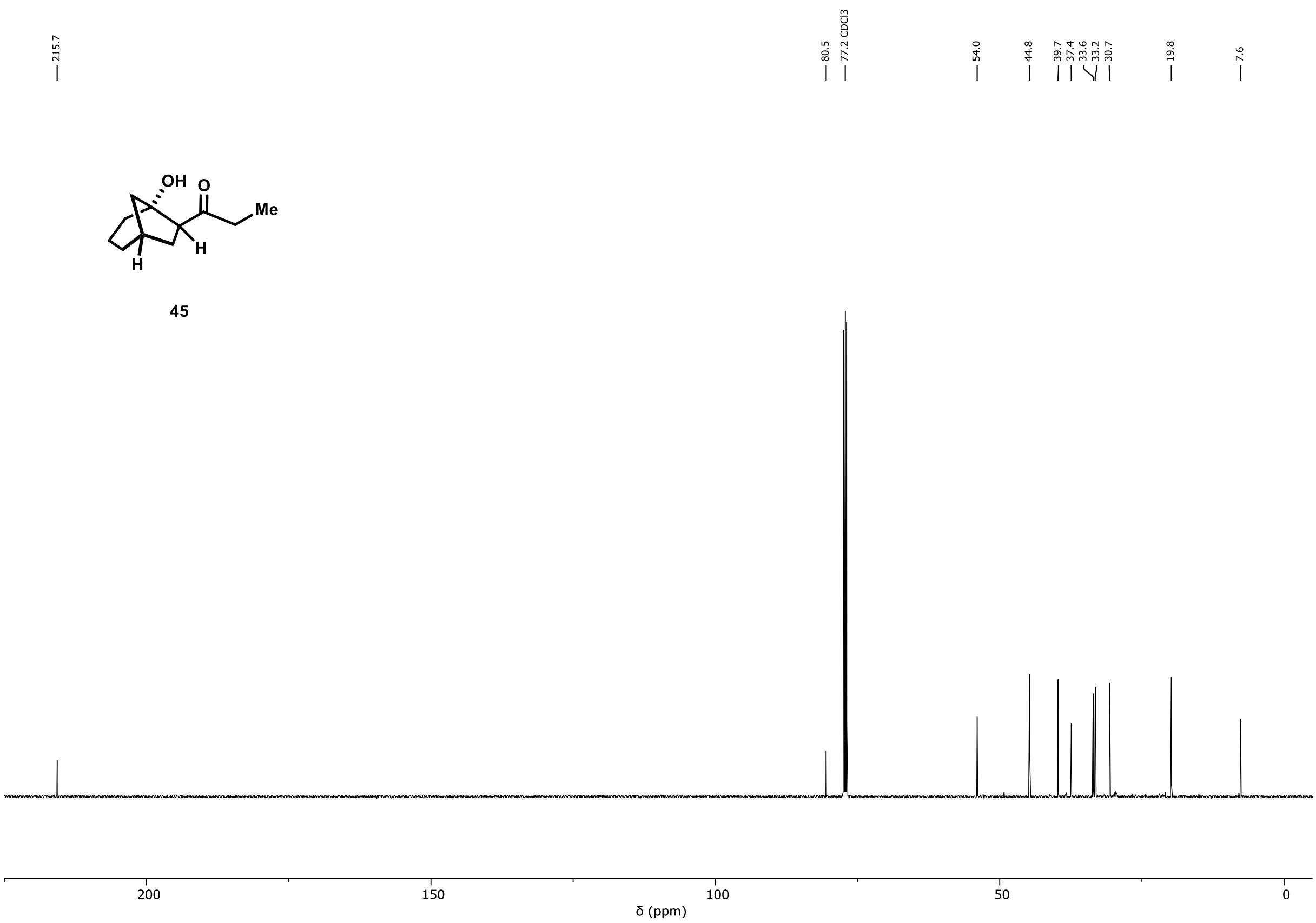
215.7
80.5
77.2 CDCl3
54.0
44.8
39.7
37.4
33.6
33.2
30.7
19.8
7.6
OH
O
Me
H
H
45
200
150
100
50
0
δ (ppm)

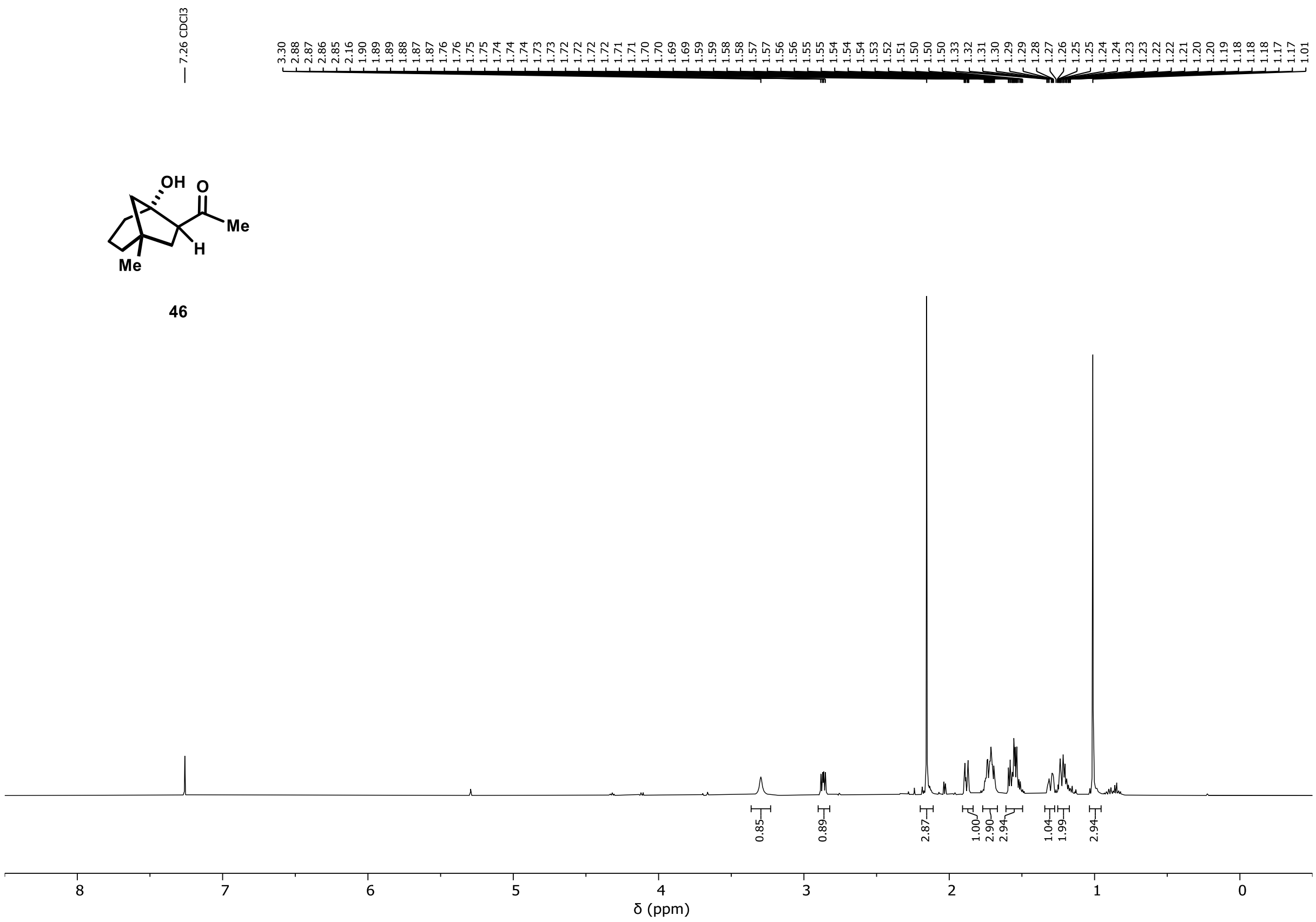
7.26 CDCl3
3.30
2.88
2.87
2.86
2.85
2.16
1.90
1.89
1.89
1.88
1.87
1.87
1.76
1.76
1.75
1.75
1.74
1.74
1.73
1.73
1.72
1.72
1.72
1.72
1.71
1.71
1.70
1.69
1.69
1.59
1.59
1.58
1.58
1.57
1.57
1.56
1.56
1.55
1.55
1.54
1.54
1.54
1.53
1.52
1.51
1.50
1.50
1.50
1.33
1.32
1.31
1.30
1.29
1.29
1.28
1.27
1.26
1.25
1.25
1.24
1.24
1.23
1.23
1.22
1.22
1.21
1.20
1.20
1.19
1.18
1.18
1.18
1.17
1.17
1.01
OH
O
Me
H
Me
46
0.85
0.89
2.87
1.00
2.90
2.94
1.04
1.99
2.94
8
7
6
5
4
3
2
1
0
δ (ppm)

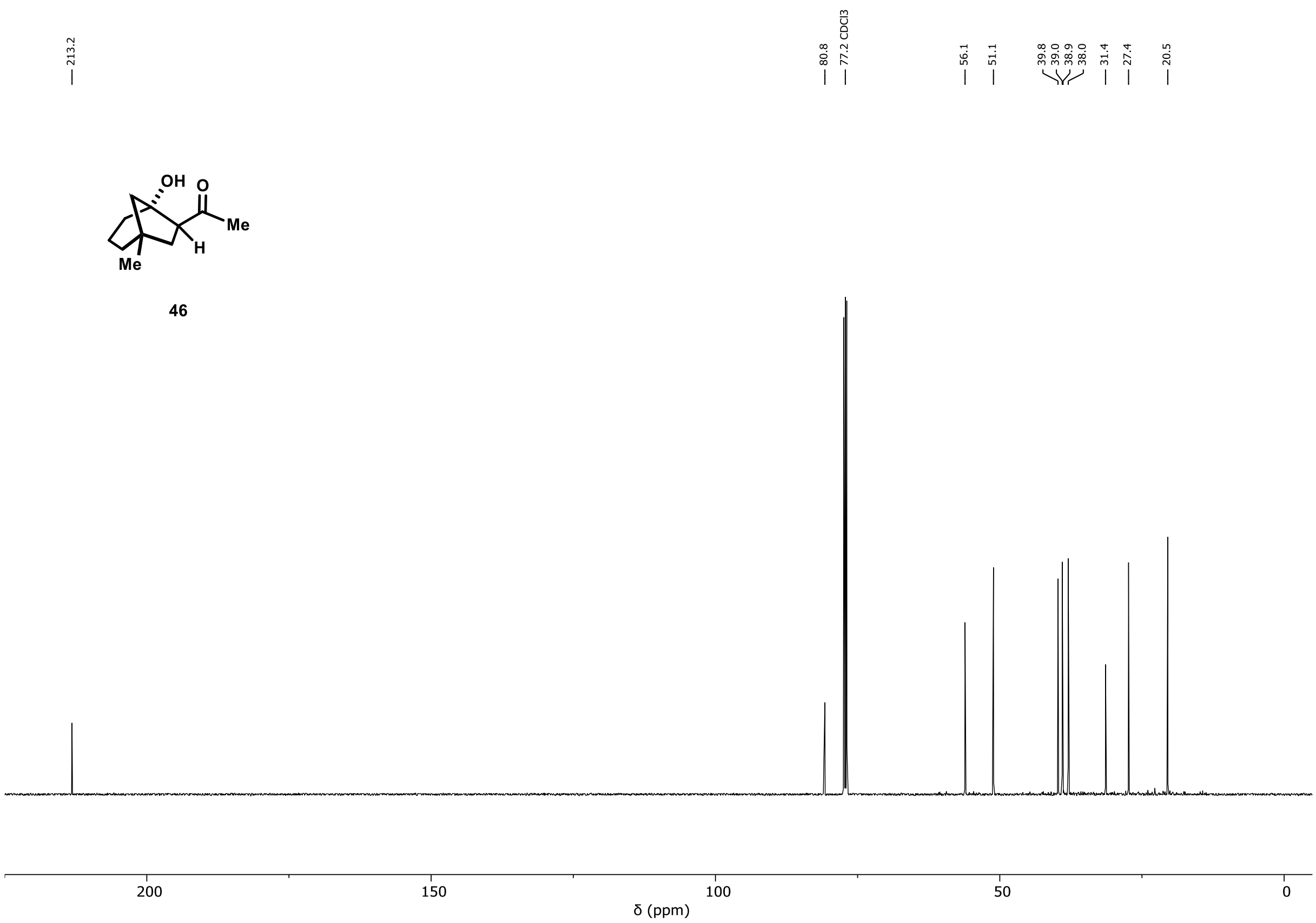
213.2
80.8
77.2 CDCl3
56.1
51.1
39.8
39.0
38.9
38.0
31.4
27.4
20.5
OH
O
Me
H
Me
46
200
150
100
50
0
δ (ppm)

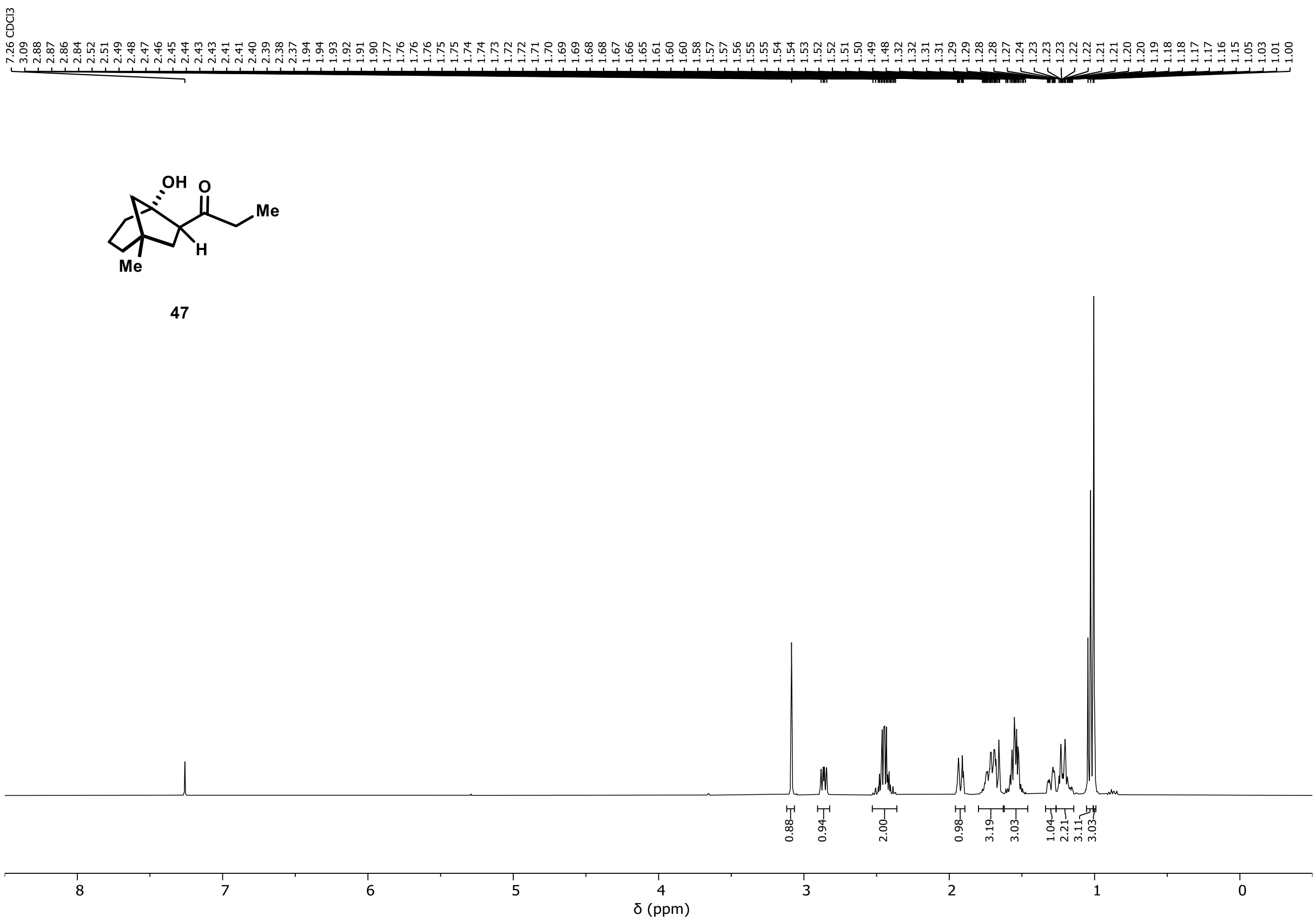
7.26 CDCl3
3.09
2.88
2.87
2.86
2.84
2.52
2.51
2.49
2.48
2.47
2.46
2.45
2.44
2.43
2.43
2.41
2.40
2.39
2.38
2.37
1.94
1.94
1.93
1.92
1.91
1.90
1.77
1.76
1.76
1.76
1.75
1.75
1.74
1.74
1.73
1.72
1.72
1.71
1.70
1.69
1.69
1.68
1.68
1.67
1.66
1.65
1.61
1.60
1.60
1.58
1.57
1.57
1.56
1.56
1.55
1.55
1.54
1.54
1.53
1.52
1.52
1.51
1.50
1.49
1.48
1.32
1.32
1.32
1.31
1.31
1.29
1.29
1.28
1.28
1.27
1.24
1.23
1.23
1.23
1.22
1.22
1.21
1.21
1.21
1.20
1.20
1.19
1.18
1.18
1.17
1.17
1.16
1.15
1.05
1.03
1.01
1.00
OH
O
Me
H
Me
47
0.88
0.94
2.00
0.98
3.19
3.03
1.04
2.21
3.11
3.03
8
7
6
5
4
3
2
1
0
δ (ppm)

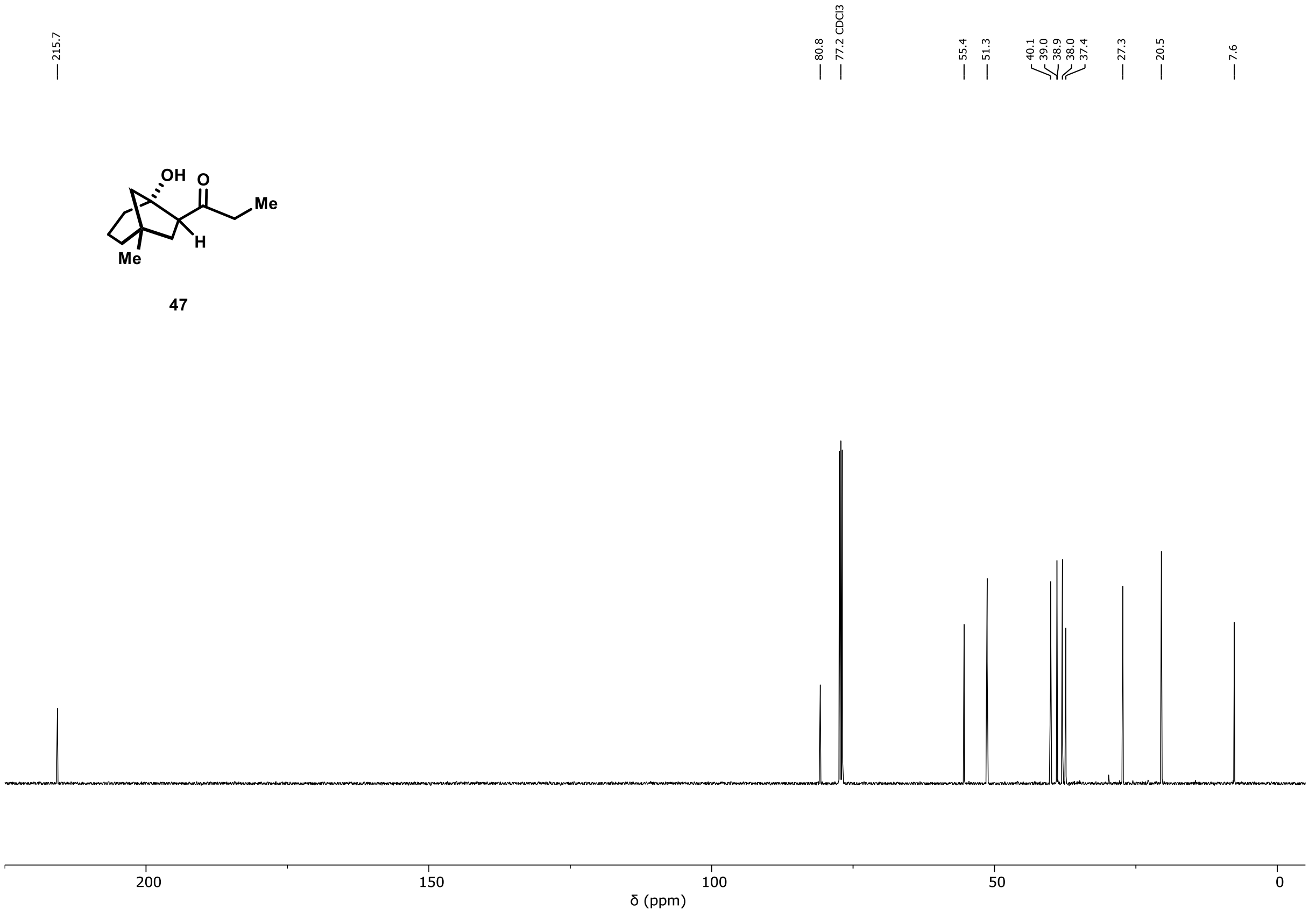
215.7
80.8
77.2 CDCl3
55.4
51.3
40.1
39.0
38.9
38.0
37.4
27.3
20.5
7.6
OH
O
Me
H
Me
47
200
150
100
50
0
δ (ppm)

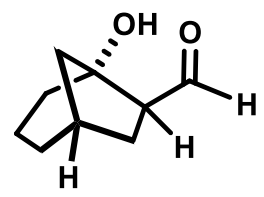

48

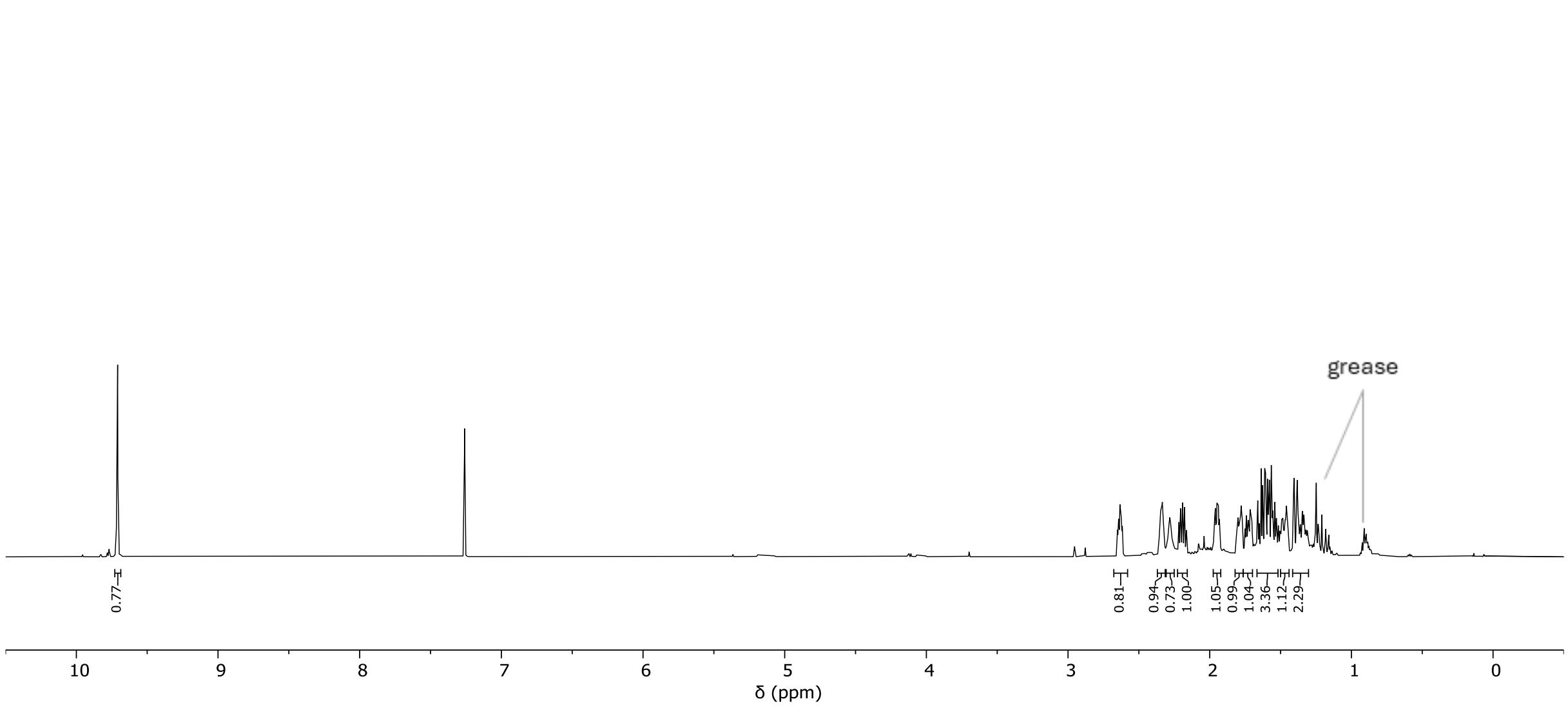
9.71 9.71 7.26 CDCl3 2.64 2.63 2.63 2.62 2.35 2.34 2.33 2.33 2.28 2.22 2.22 2.21 2.20 2.20 2.19 2.19 2.18 2.18 2.18 1.97 1.96 1.96 1.96 1.95 1.95 1.95 1.94 1.94 1.93 1.93 1.93 1.80 1.80 1.79 1.79 1.78 1.78 1.77 1.77 1.74 1.74 1.73 1.73 1.72 1.71 1.70 1.70 1.66 1.65 1.64 1.63 1.61 1.61 1.60 1.59 1.59 1.58 1.58 1.57 1.56 1.56 1.55 1.54 1.53 1.49 1.49 1.48 1.47 1.47 1.46 1.46 1.46 1.45 1.41 1.40 1.40 1.39 1.38 1.38 1.38 1.37 1.37 1.36 1.35 1.35 1.34 1.34 1.33
grease
0.77
0.81
0.94
0.73
1.00
1.05
0.99
1.04
3.36
1.12
2.29
10 9 8 7 6 5 4 3 2 1 0
δ (ppm)

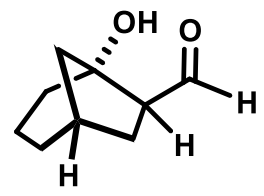

48

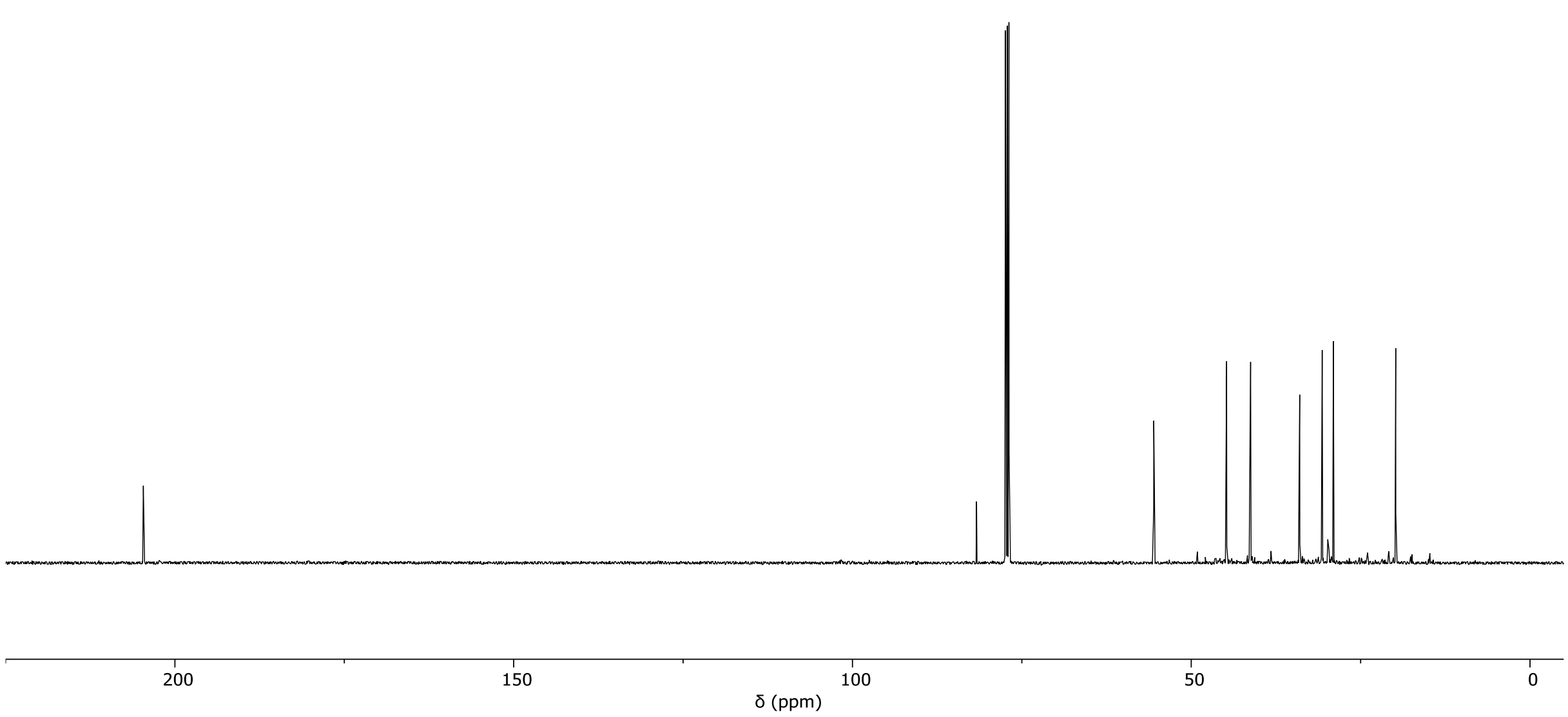
204.7
81.7
77.2 CDCl3
55.5
44.8
41.2
34.0
30.7
29.0
19.8
200 150 100 50 0
δ (ppm)

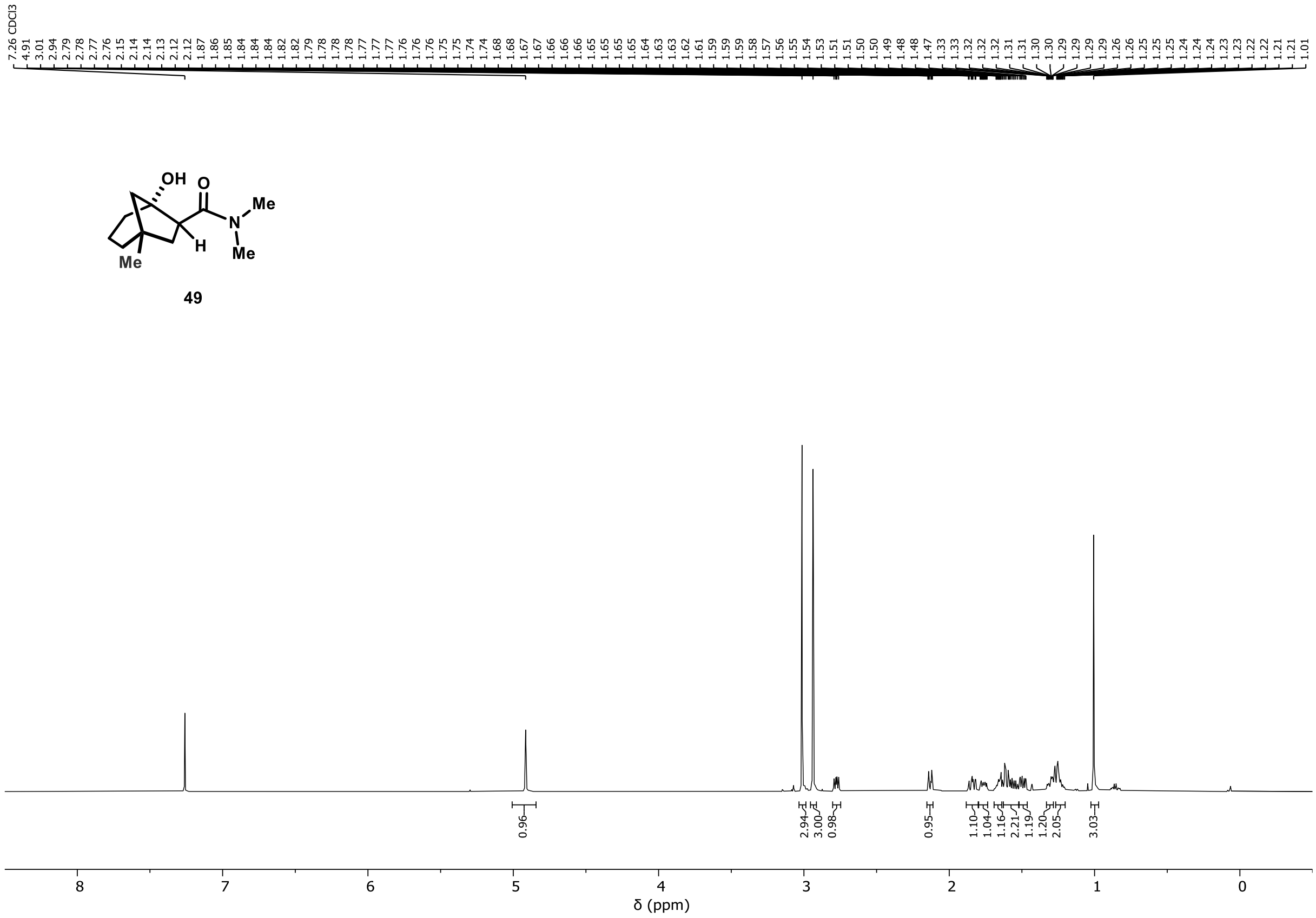
7.26 CDCl3
4.91
3.01
2.94
2.79
2.78
2.77
2.76
2.15
2.14
2.14
2.13
2.12
2.12
1.87
1.86
1.85
1.84
1.84
1.84
1.82
1.82
1.79
1.78
1.78
1.78
1.77
1.77
1.77
1.76
1.76
1.76
1.75
1.75
1.74
1.74
1.68
1.68
1.67
1.67
1.66
1.66
1.65
1.65
1.65
1.65
1.64
1.63
1.63
1.62
1.61
1.59
1.59
1.59
1.59
1.58
1.57
1.56
1.55
1.54
1.53
1.51
1.51
1.51
1.50
1.50
1.49
1.48
1.48
1.47
1.33
1.33
1.32
1.32
1.32
1.31
1.31
1.30
1.30
1.29
1.29
1.29
1.29
1.26
1.25
1.25
1.25
1.24
1.24
1.24
1.23
1.23
1.22
1.22
1.21
1.21
1.01
OH
O
Me
N
H
Me
Me
49
0.96
2.94
3.00
0.98
0.95
1.10
1.04
1.16
2.21
1.19
1.20
2.05
3.03
8
7
6
5
4
3
2
1
0
δ (ppm)

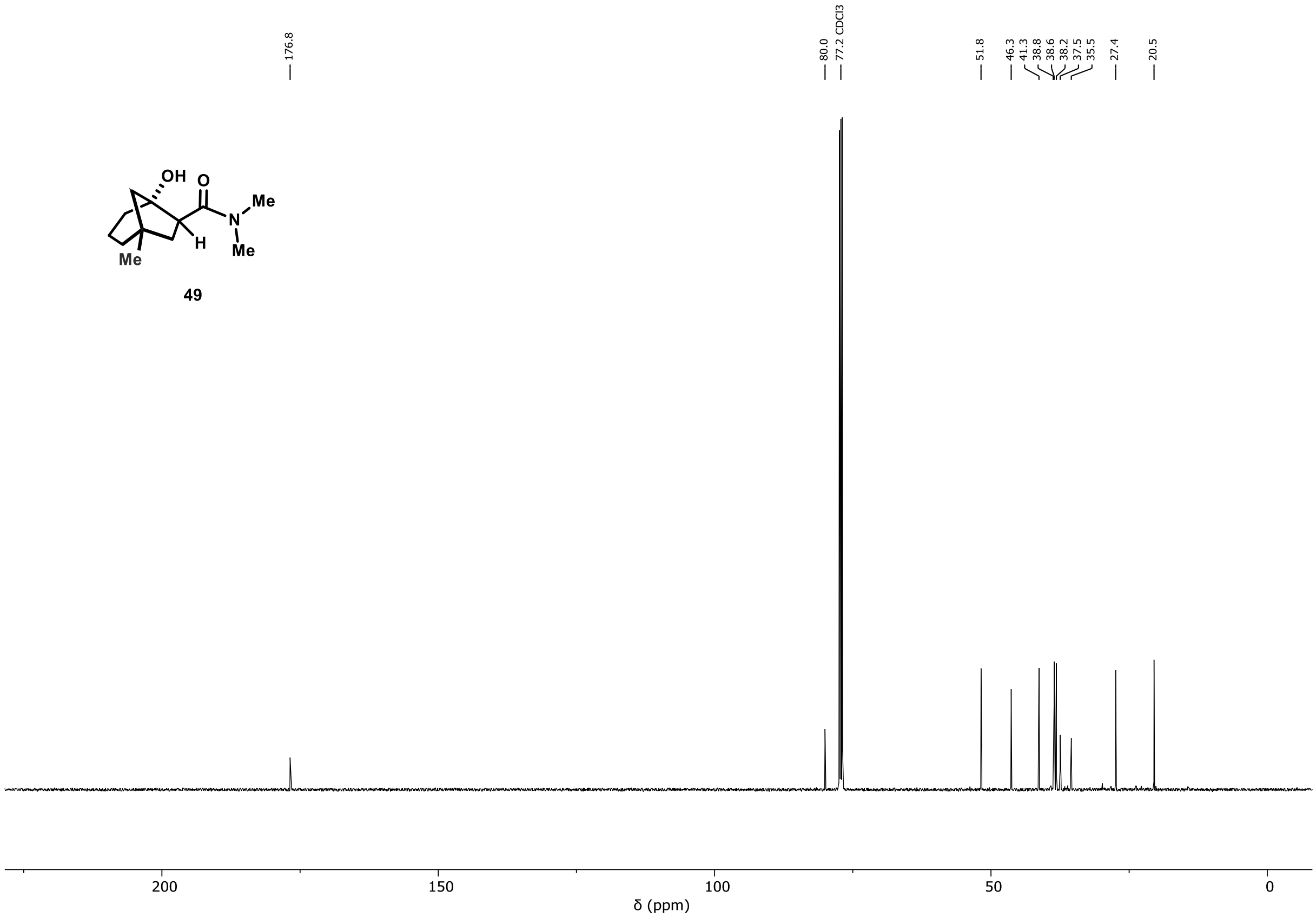
176.8
80.0
77.2 CDCl3
51.8
46.3
41.3
38.8
38.6
38.2
37.5
35.5
27.4
20.5
OH
O
Me
N
H
Me
Me
49
200
150
100
50
0
δ (ppm)

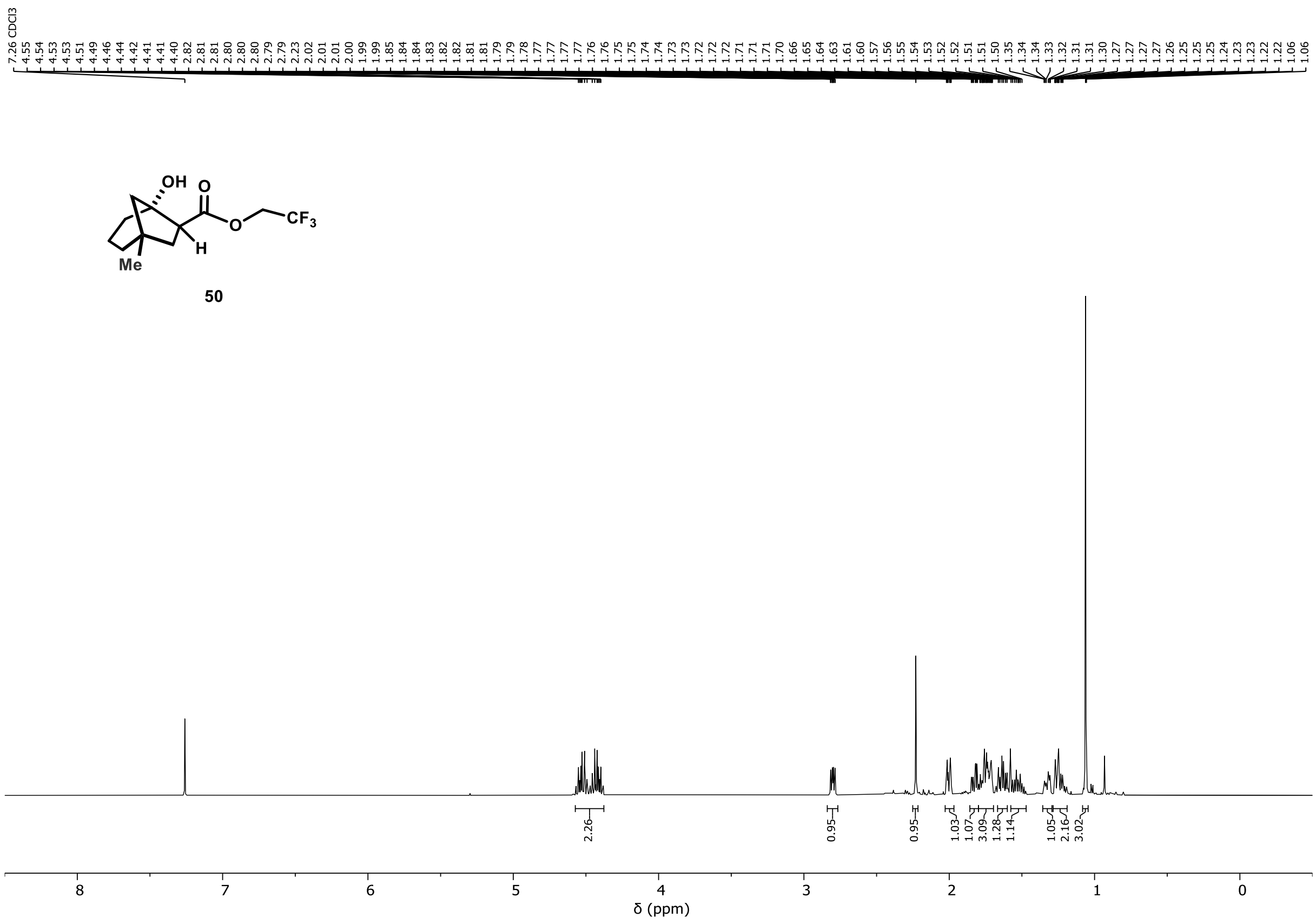

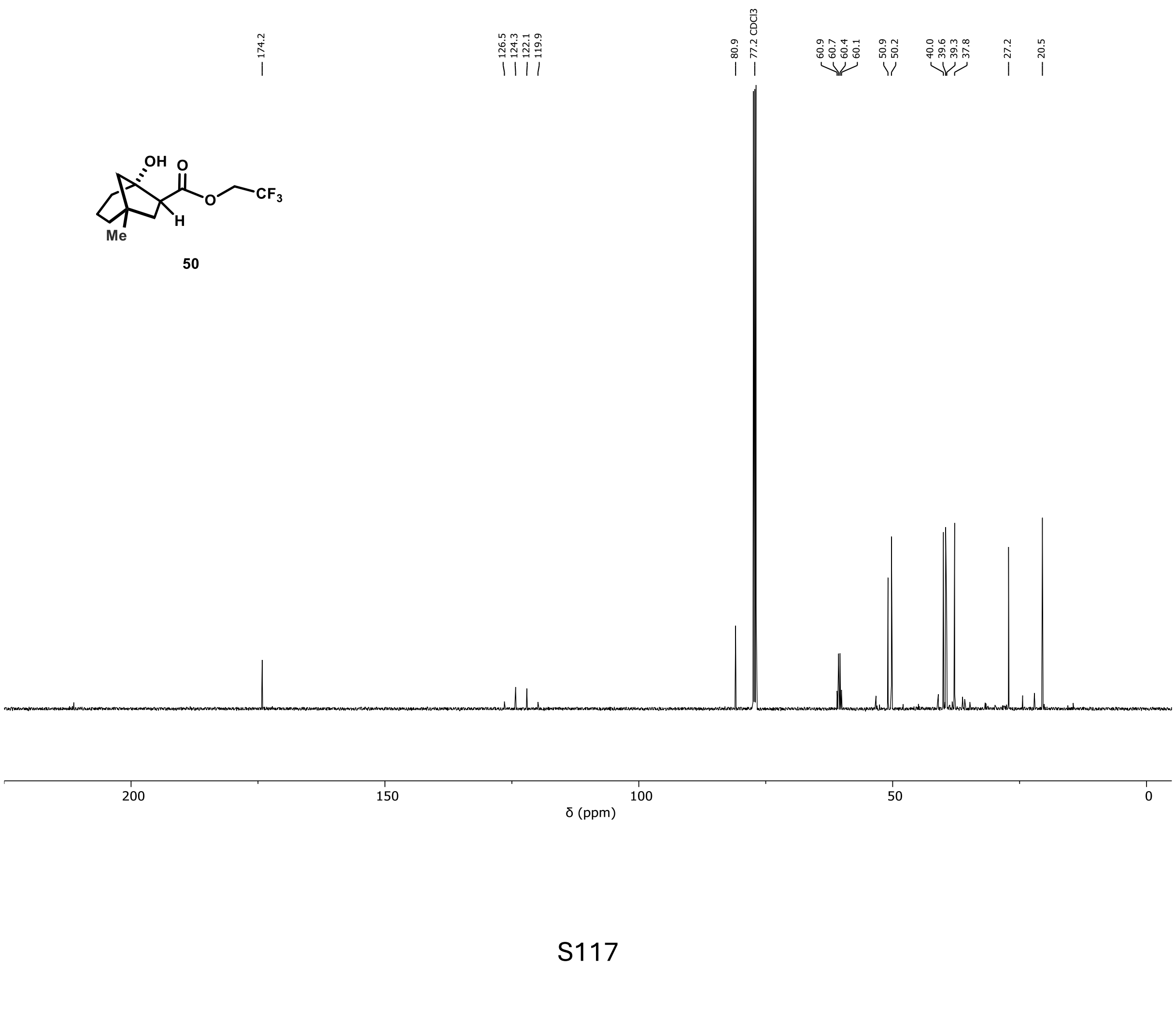

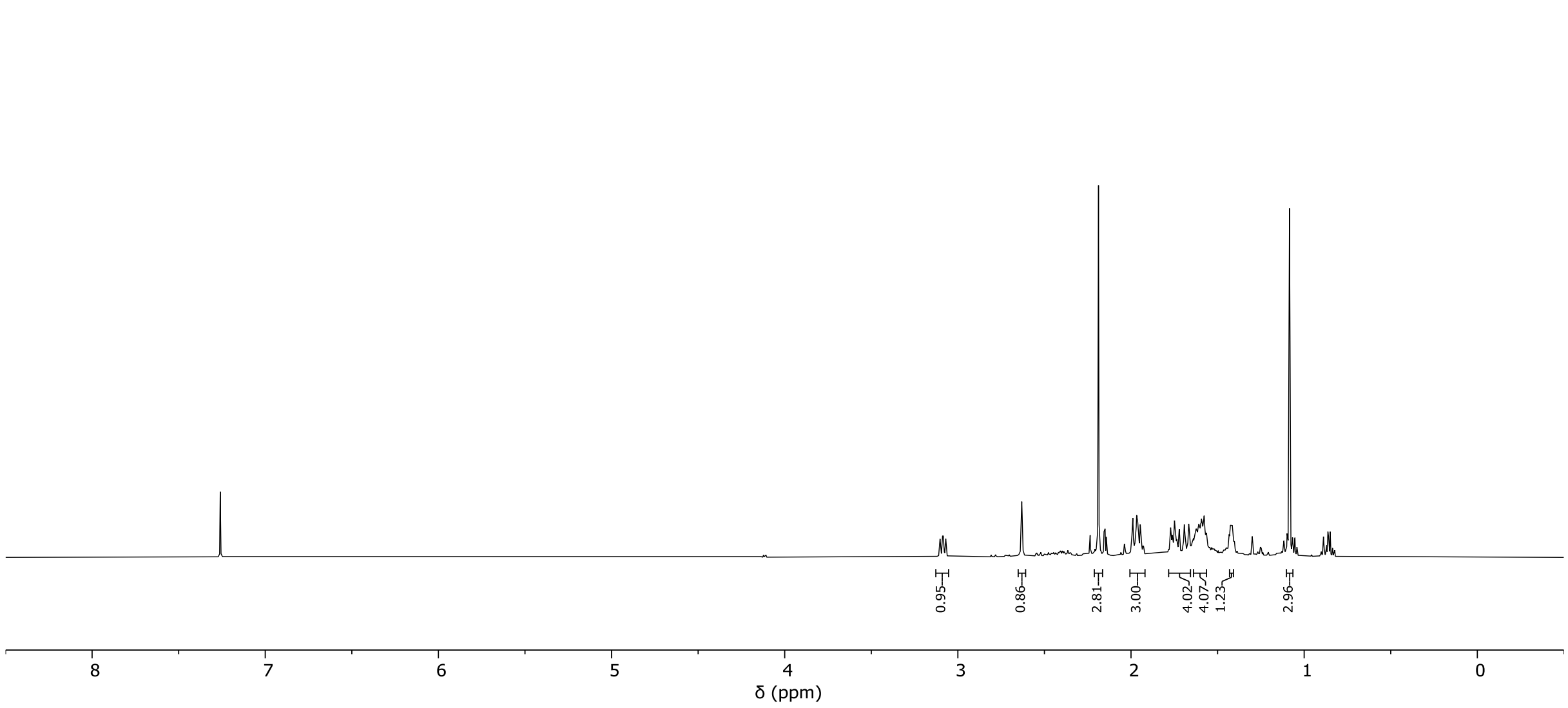

7.26 CDCl3
0.95
0.86
2.81
3.00
4.02
4.07
1.23
2.96
δ (ppm)

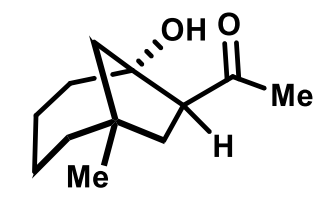

OH
O
Me
H
Me

51

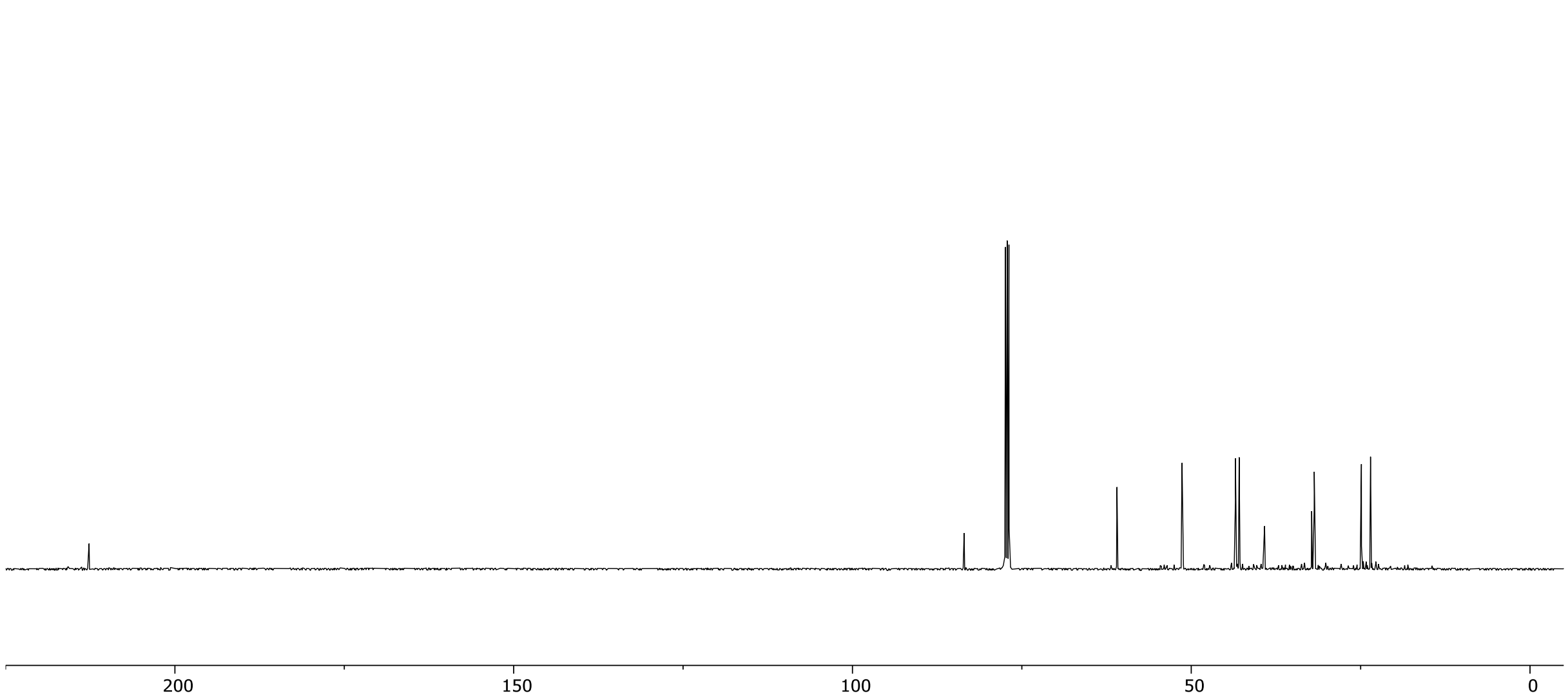

212.7
83.5
77.2 CDCl3
61.0
51.4
43.5
43.4
42.9
39.2
32.2
31.8
24.9
23.5
δ (ppm)

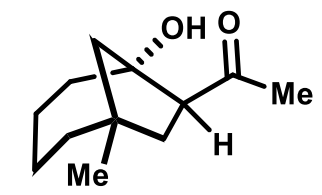

OH
O
Me
H
Me

51

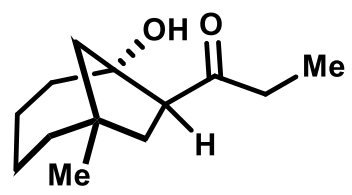
OH
O
Me
H
Me

52

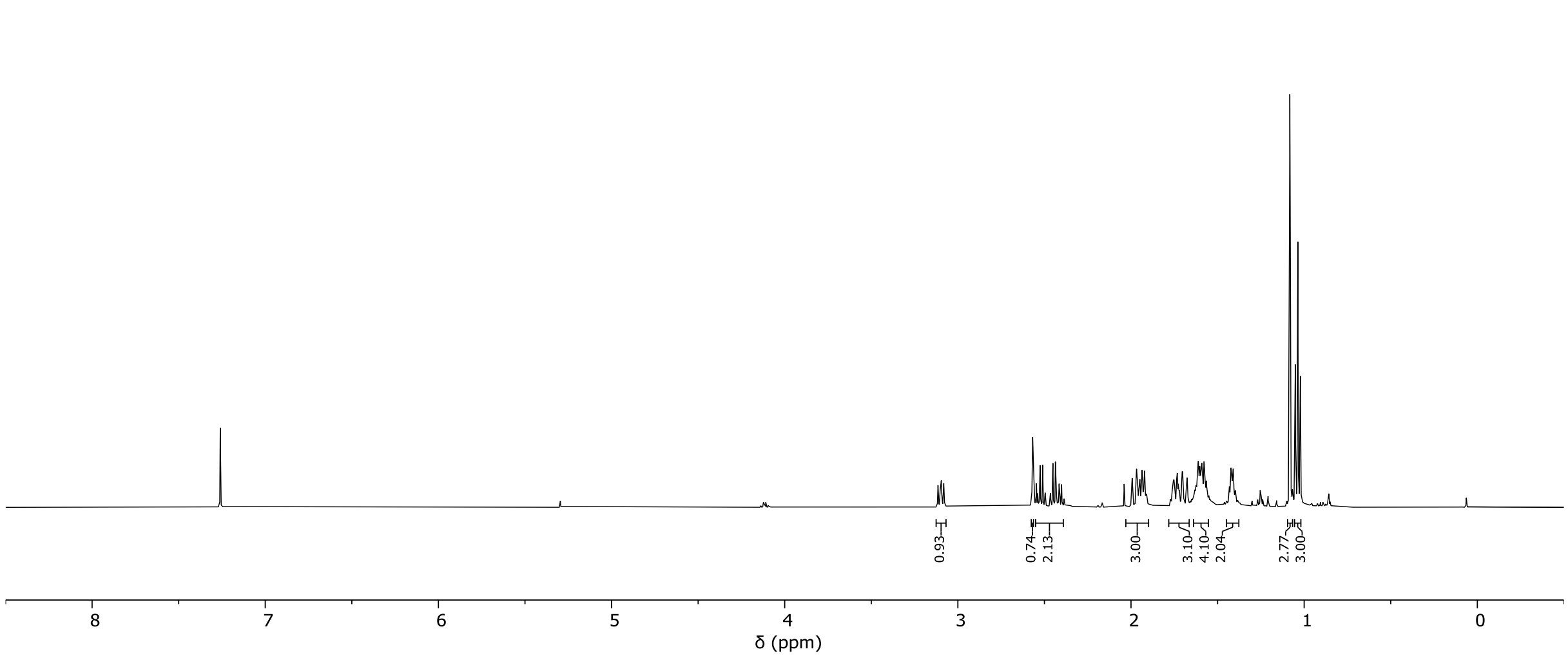
7.26 CDCl3
3.11
3.10
3.10
3.08
2.57
2.55
2.54
2.53
2.52
2.51
2.50
2.47
2.45
2.44
2.43
2.42
2.42
2.00
1.99
1.97
1.97
1.96
1.96
1.95
1.95
1.95
1.94
1.94
1.93
1.93
1.93
1.92
1.92
1.91
1.91
1.77
1.77
1.76
1.76
1.75
1.75
1.74
1.73
1.73
1.72
1.72
1.71
1.70
1.70
1.70
1.68
1.68
1.67
1.67
1.66
1.65
1.64
1.64
1.63
1.62
1.62
1.61
1.61
1.60
1.60
1.59
1.59
1.59
1.59
1.58
1.58
1.57
1.57
1.57
1.56
1.56
1.55
1.54
1.54
1.43
1.43
1.43
1.42
1.42
1.41
1.41
1.40
1.40
1.08
1.05
1.04
1.02
0.93
0.74
2.13
3.00
3.10
4.10
2.04
2.77
3.00
8
7
6
5
4
3
2
1
0
δ (ppm)

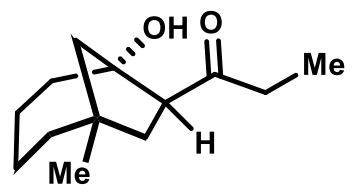
OH
O
Me
H
Me

52

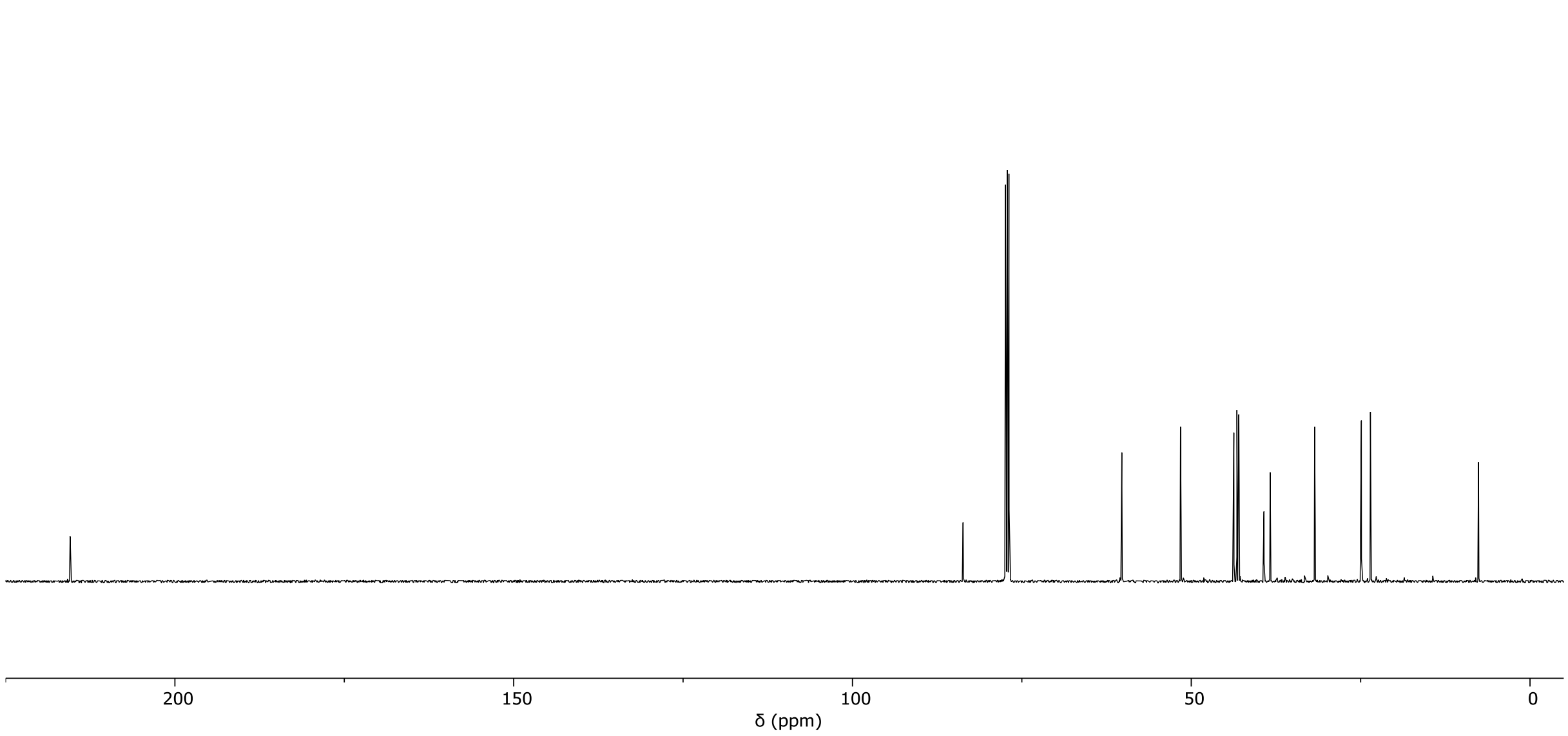
215.5
83.7
77.2 CDCl3
60.2
51.6
43.7
43.3
43.0
39.3
38.3
31.8
24.9
23.6
7.6
200
150
100
50
0
δ (ppm)

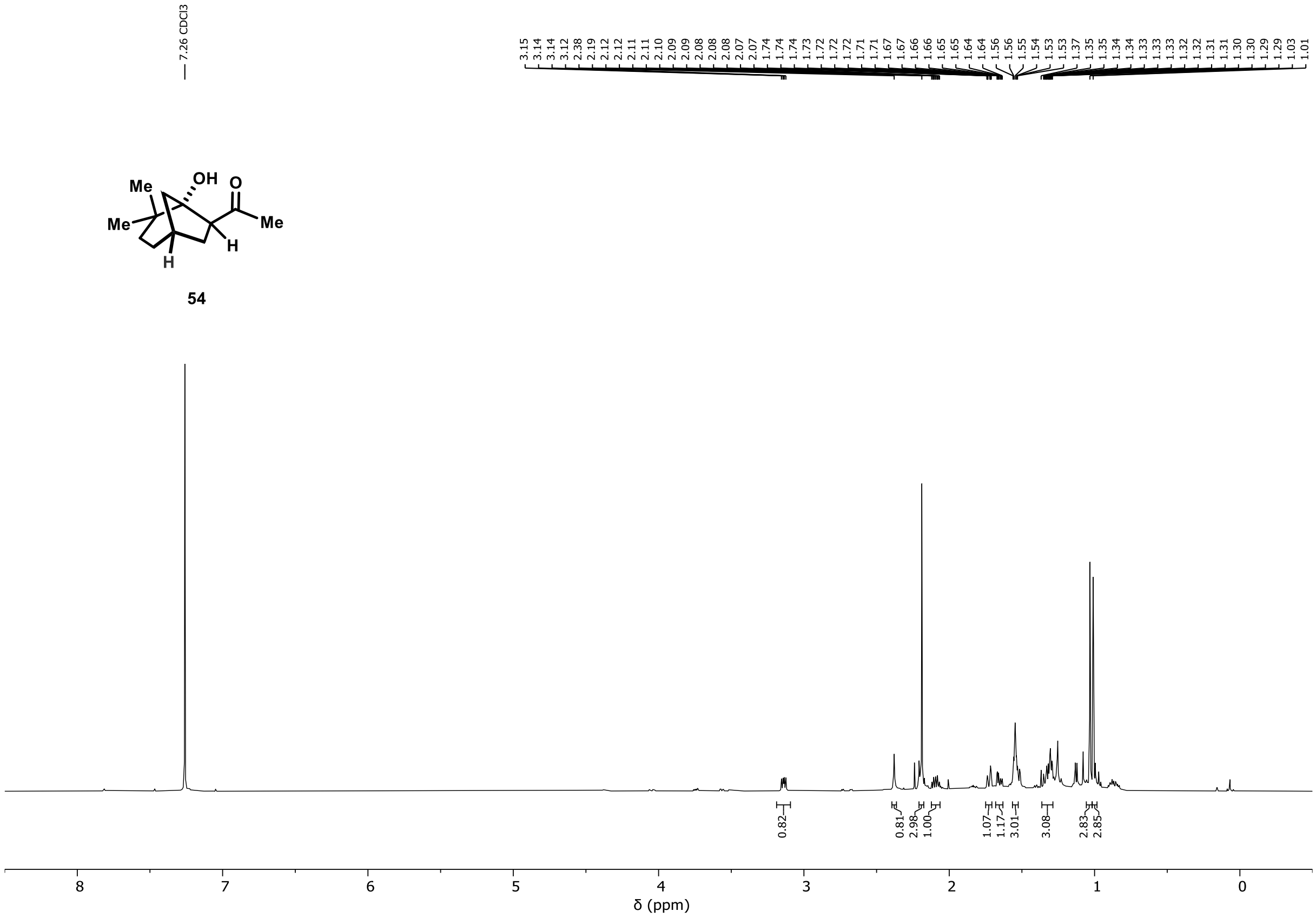
7.26 CDCl3
3.15 3.14 3.14 3.12 2.38 2.19 2.12 2.12 2.11 2.11 2.10 2.09 2.09 2.08 2.08 2.08 2.07 2.07 1.74 1.74 1.74 1.73 1.72 1.72 1.72 1.71 1.71 1.67 1.67 1.66 1.66 1.65 1.65 1.64 1.64 1.56 1.56 1.55 1.54 1.53 1.53 1.37 1.35 1.35 1.34 1.34 1.33 1.33 1.33 1.32 1.32 1.31 1.31 1.30 1.30 1.29 1.29 1.03 1.01
Me
OH
O
Me
Me
H
H
54
0.82
0.81
2.98
1.00
1.07
1.17
3.01
3.08
2.83
2.85
δ (ppm)

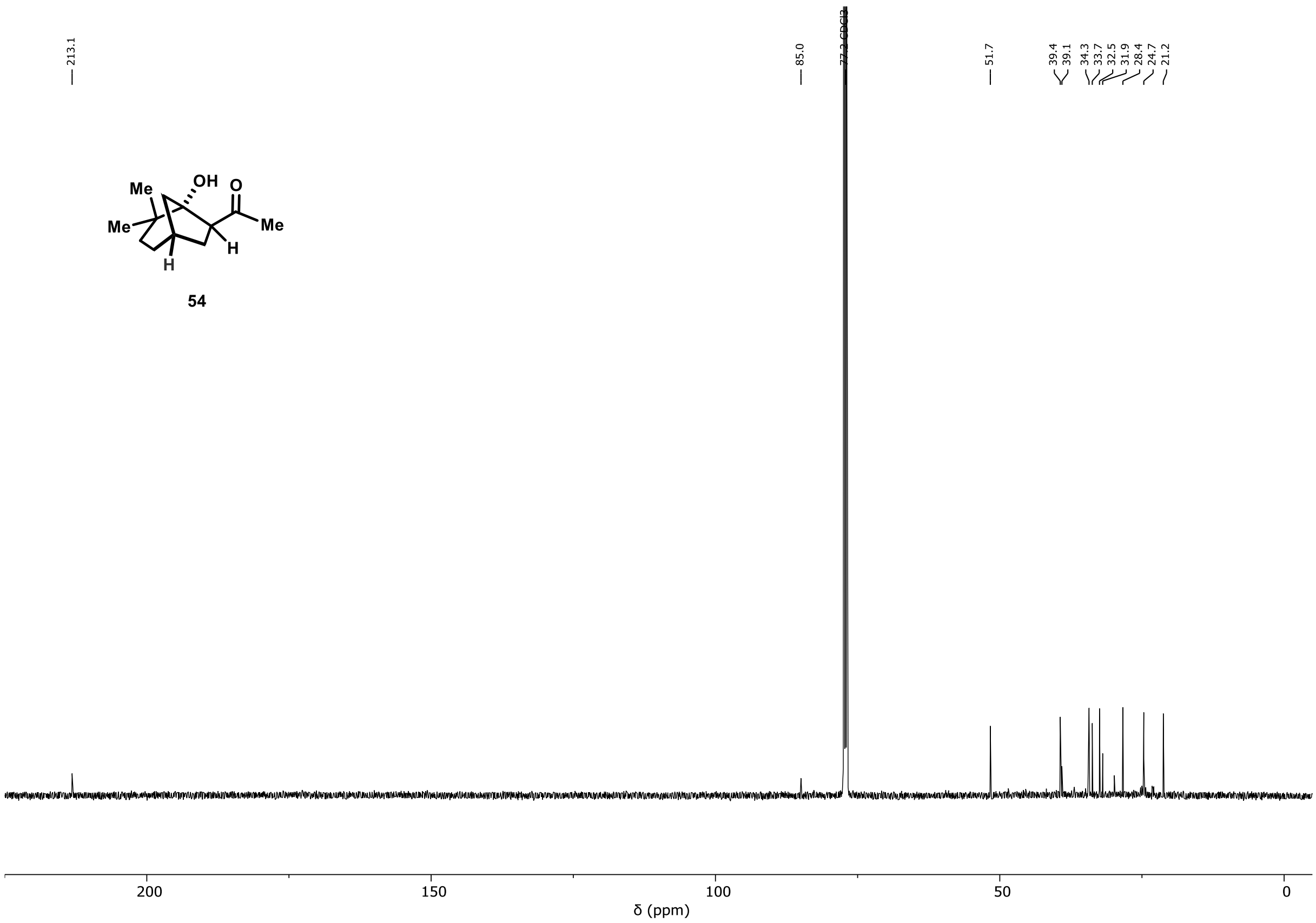
213.1
85.0
77.2 CDCl3
51.7
39.4 39.1 34.3 33.7 32.5 31.9 28.4 24.7 21.2
Me
OH
O
Me
Me
H
H
54
δ (ppm)

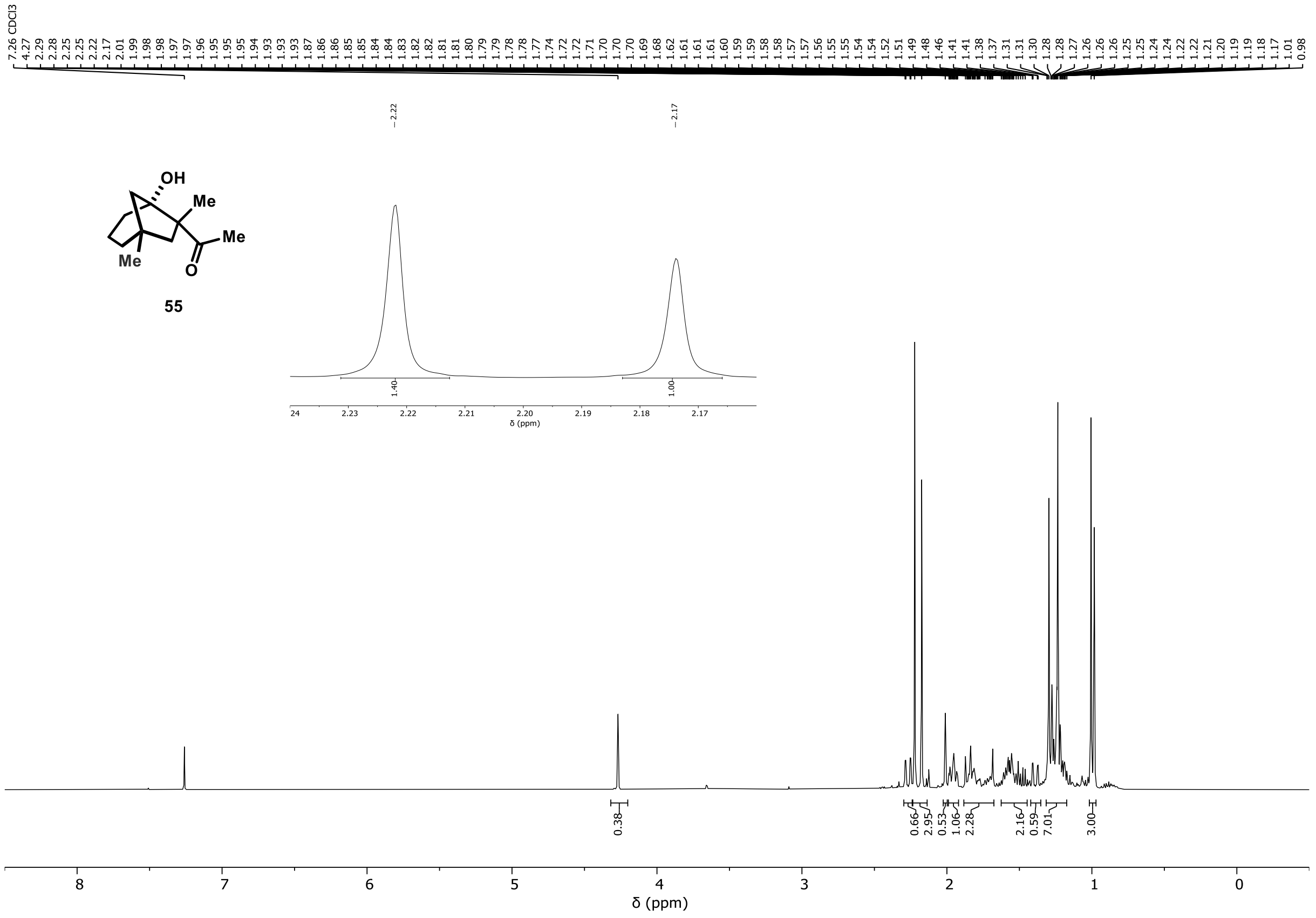

7.26 CDCl3
4.27
2.29
2.28
2.25
2.25
2.22
2.17
2.01
1.99
1.98
1.98
1.97
1.97
1.96
1.95
1.95
1.95
1.94
1.93
1.93
1.93
1.87
1.86
1.86
1.85
1.85
1.84
1.84
1.83
1.82
1.82
1.81
1.81
1.80
1.79
1.79
1.78
1.78
1.77
1.74
1.72
1.72
1.71
1.70
1.70
1.70
1.69
1.68
1.62
1.61
1.61
1.61
1.60
1.59
1.59
1.58
1.58
1.57
1.57
1.56
1.55
1.55
1.54
1.54
1.52
1.51
1.49
1.48
1.46
1.41
1.41
1.38
1.37
1.31
1.31
1.30
1.28
1.28
1.28
1.27
1.26
1.26
1.25
1.25
1.24
1.24
1.22
1.22
1.21
1.20
1.19
1.19
1.18
1.17
1.01
0.98
OH
Me
Me
Me
O
55
2.22
2.17
1.40
1.00
0.38
0.66
2.95
0.53
1.06
2.28
2.16
0.59
7.01
3.00
δ (ppm)

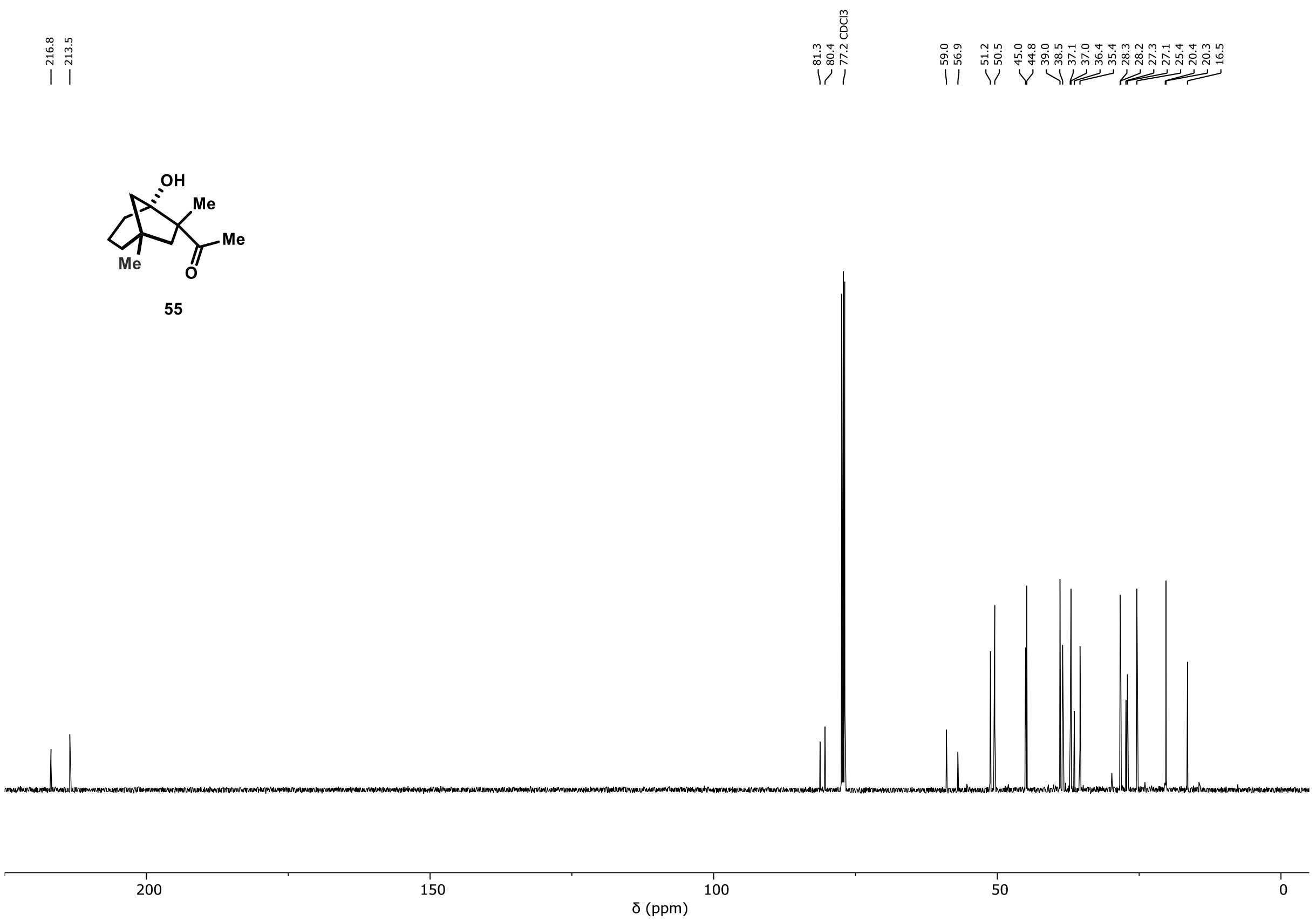

216.8
213.5
81.3
80.4
77.2 CDCl3
59.0
56.9
51.2
50.5
45.0
44.8
39.0
38.5
37.1
37.0
36.4
35.4
28.3
28.2
27.3
27.1
25.4
20.4
20.3
16.5
OH
Me
Me
Me
O
55
δ (ppm)

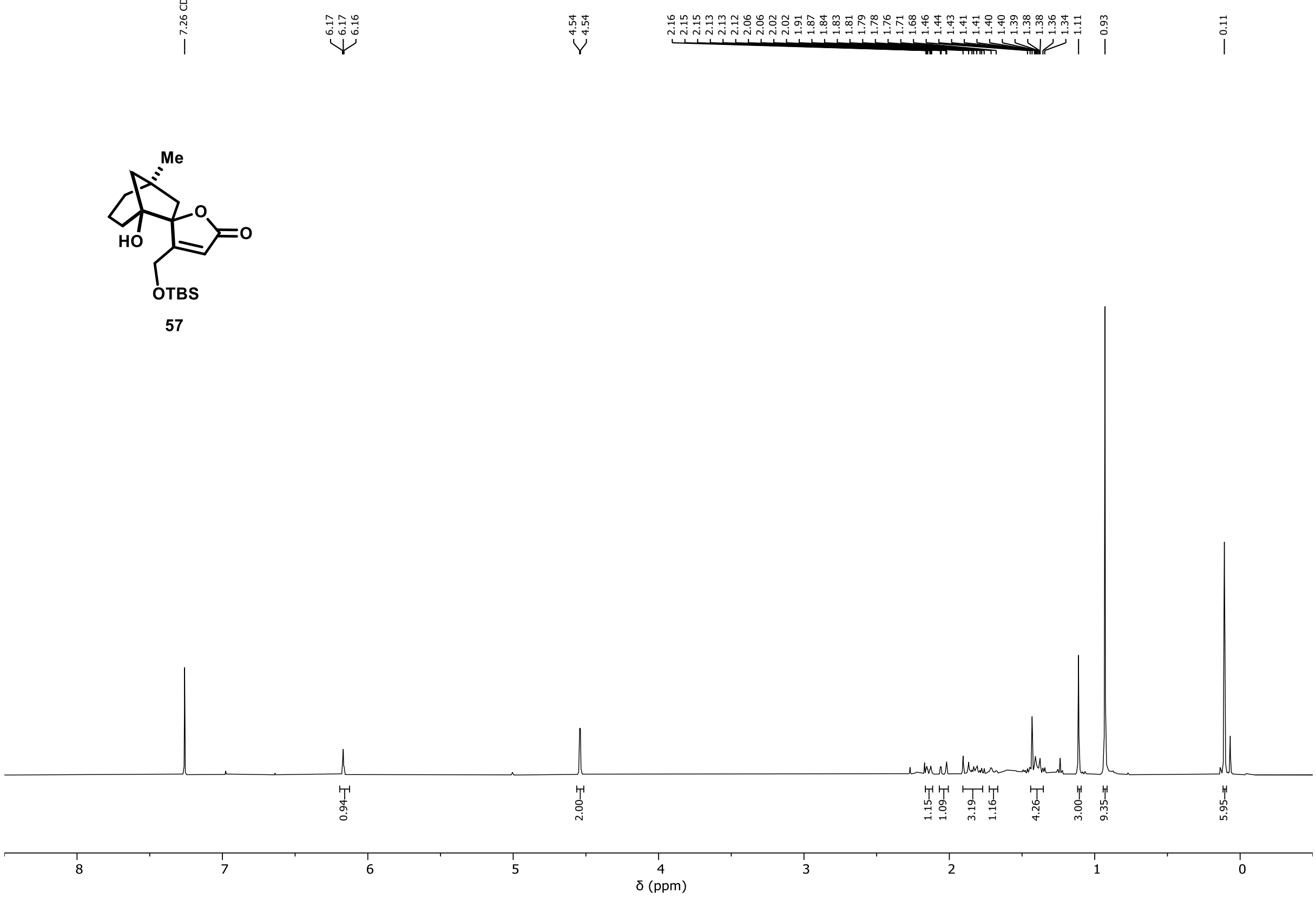
7.26 CDCl3
6.17
6.17
6.16
4.54
4.54
0.93
0.11
Me
O
O
HO
OTBS
57
0.94
2.00
1.15
1.09
3.19
1.16
4.26
3.00
9.35
5.95
δ (ppm)

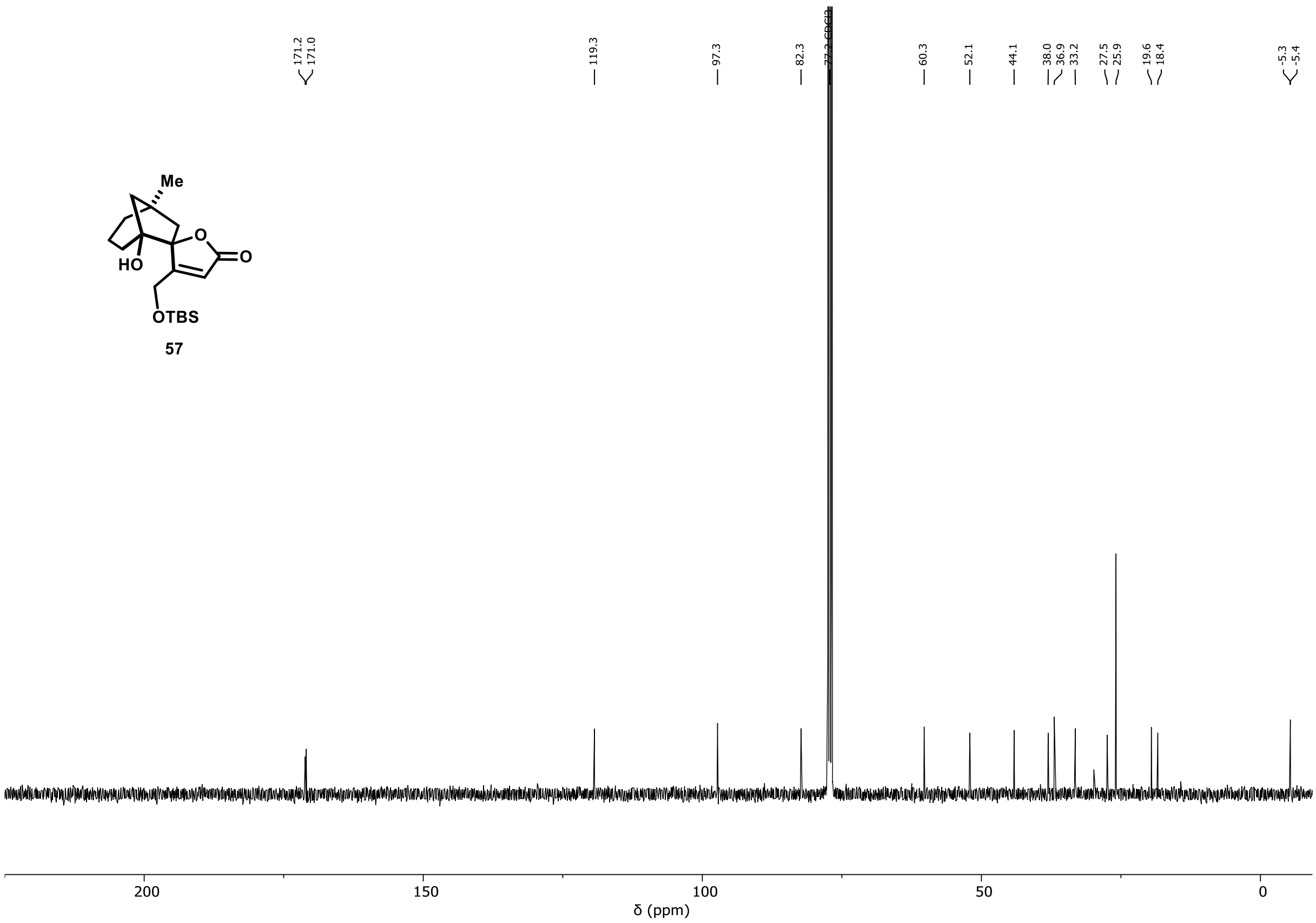
171.2
171.0
119.3
97.3
82.3
60.3
52.1
44.1
38.0
36.9
33.2
27.5
25.9
19.6
18.4
-5.3
-5.4
Me
O
O
HO
OTBS
57
δ (ppm)

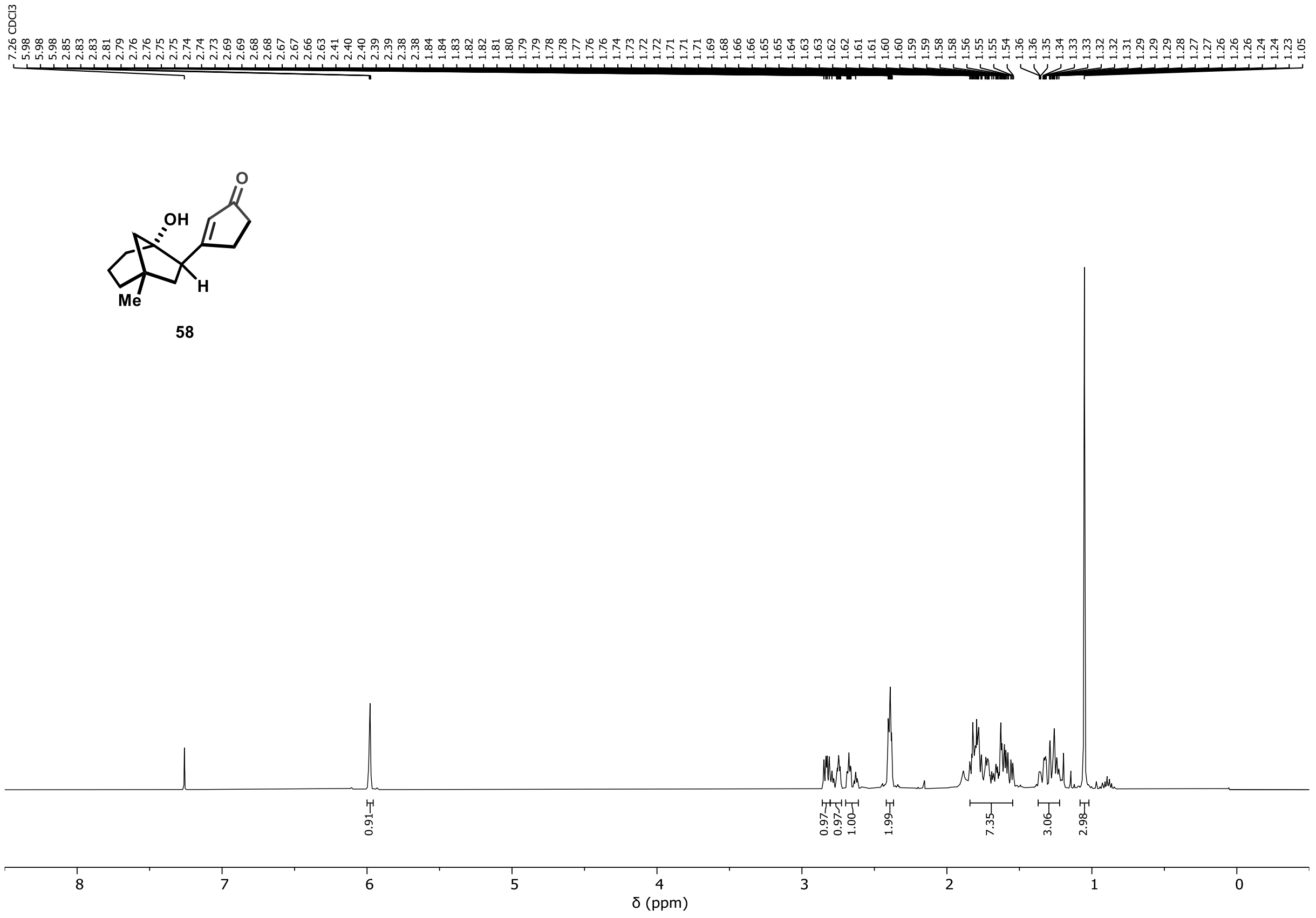
7.26 CDCl3
5.98 5.98 5.98 5.98 2.85 2.83 2.83 2.81 2.79 2.76 2.76 2.75 2.75 2.74 2.74 2.73 2.69 2.69 2.68 2.67 2.67 2.66 2.63 2.41 2.40 2.40 2.39 2.39 2.38 2.38 1.84 1.84 1.83 1.82 1.82 1.81 1.80 1.79 1.79 1.78 1.77 1.76 1.76 1.74 1.73 1.72 1.72 1.71 1.71 1.69 1.68 1.66 1.66 1.65 1.65 1.64 1.63 1.62 1.62 1.61 1.61 1.60 1.60 1.59 1.59 1.58 1.58 1.56 1.55 1.55 1.54 1.36 1.36 1.35 1.34 1.33 1.33 1.32 1.32 1.31 1.29 1.29 1.28 1.27 1.27 1.26 1.26 1.24 1.23 1.05
O
OH
H
Me
58
0.91
0.97
0.97
1.00
1.99
7.35
3.06
2.98
8 7 6 5 4 3 2 1 0
δ (ppm)

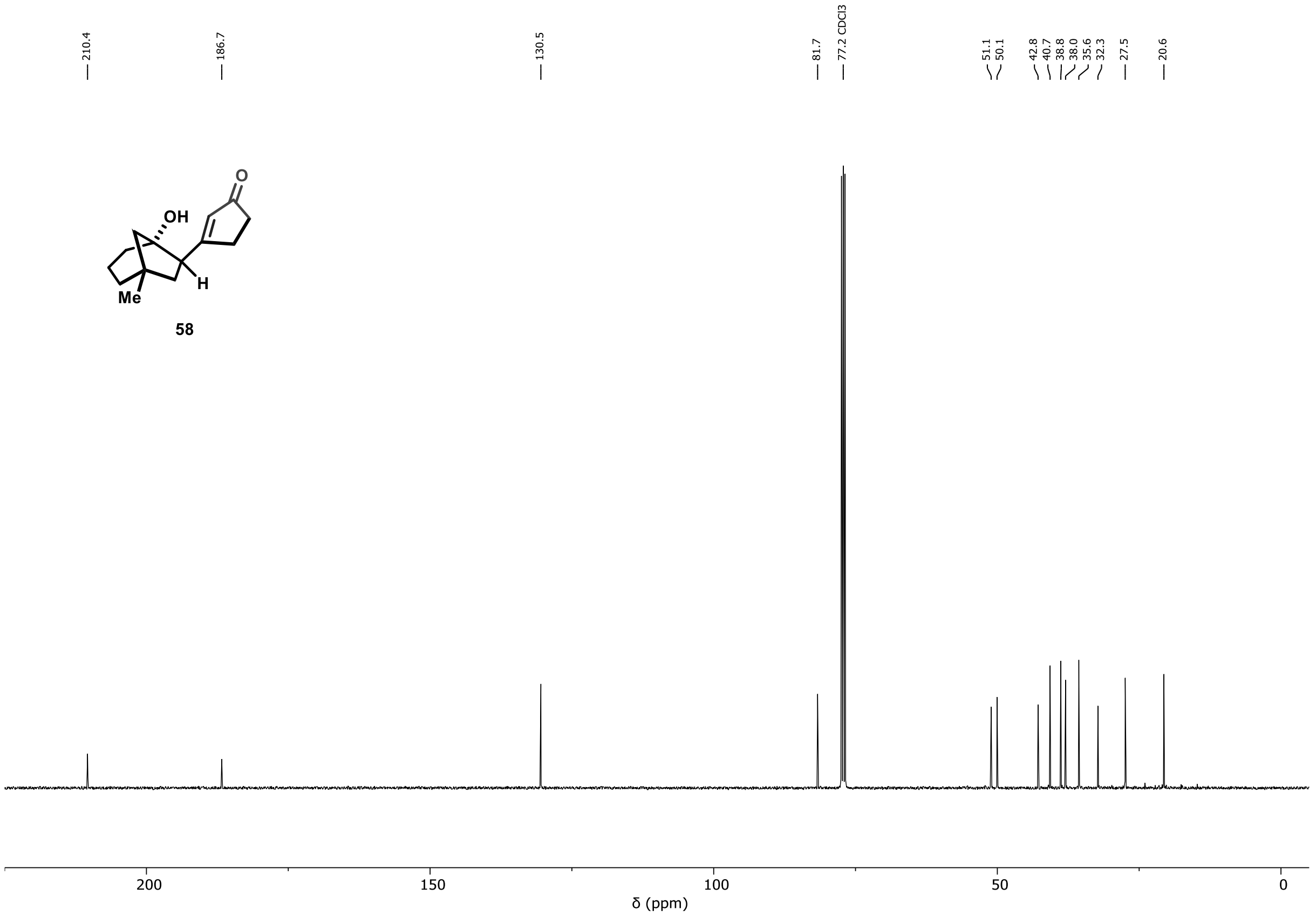
210.4
186.7
130.5
81.7
77.2 CDCl3
51.1 50.1
42.8 40.7 38.8 38.0 35.6 32.3
27.5
20.6
O
OH
H
Me
58
200 150 100 50 0
δ (ppm)

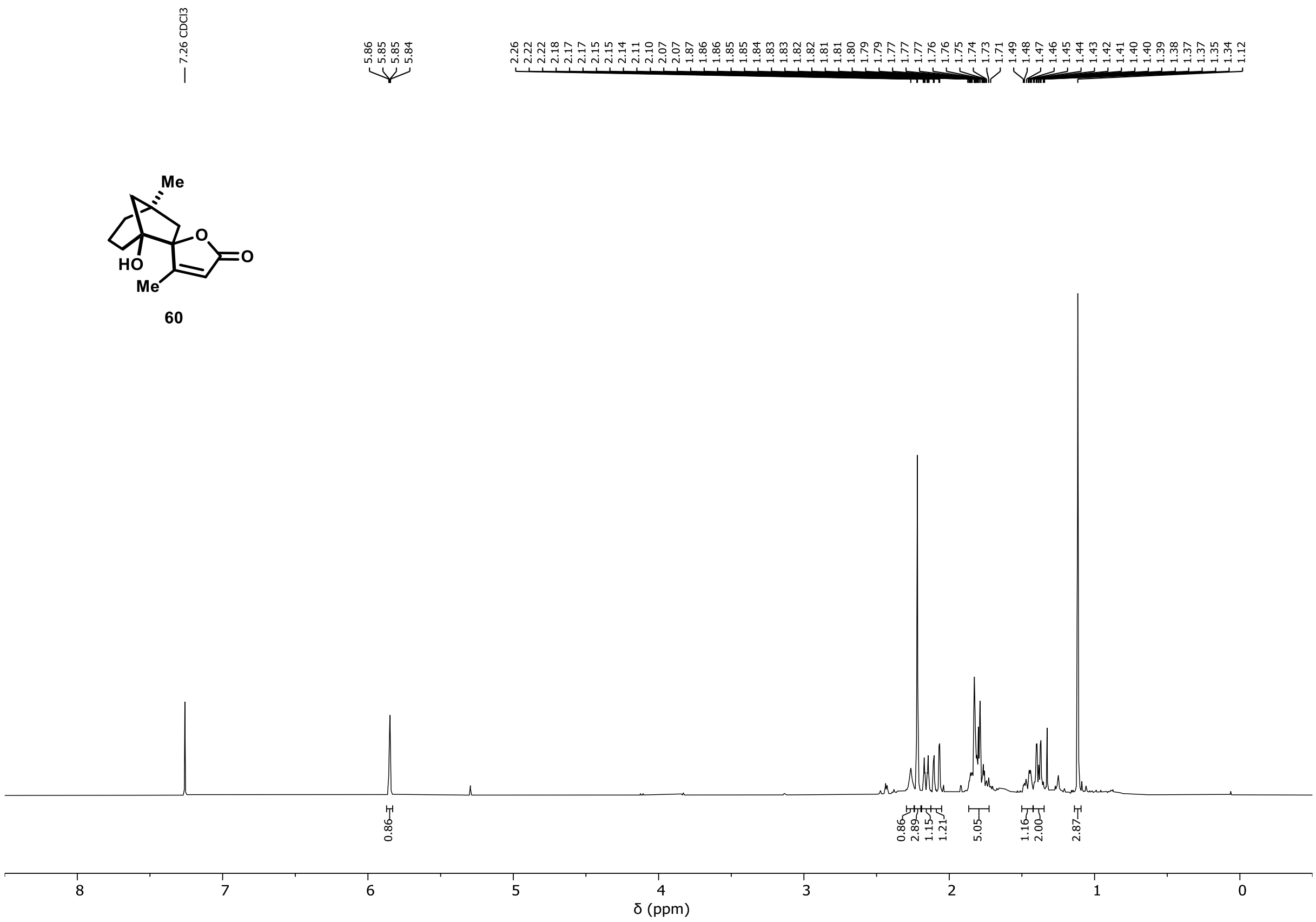
7.26 CDCl3
5.86 5.85 5.85 5.84
2.26 2.22 2.22 2.18 2.17 2.17 2.15 2.15 2.14 2.11 2.10 2.07 2.07 1.87 1.86 1.86 1.85 1.85 1.84 1.83 1.83 1.82 1.82 1.81 1.81 1.80 1.79 1.79 1.77 1.77 1.77 1.76 1.76 1.75 1.74 1.73 1.73 1.71
1.49 1.48 1.47 1.46 1.45 1.44 1.43 1.42 1.41 1.40 1.40 1.39 1.38 1.37 1.37 1.35 1.34 1.12
Me
O
HO
O
Me
60
0.86
0.86 2.89 1.15 1.21
5.05
1.16 2.00
2.87
8 7 6 5 4 3 2 1 0
δ (ppm)

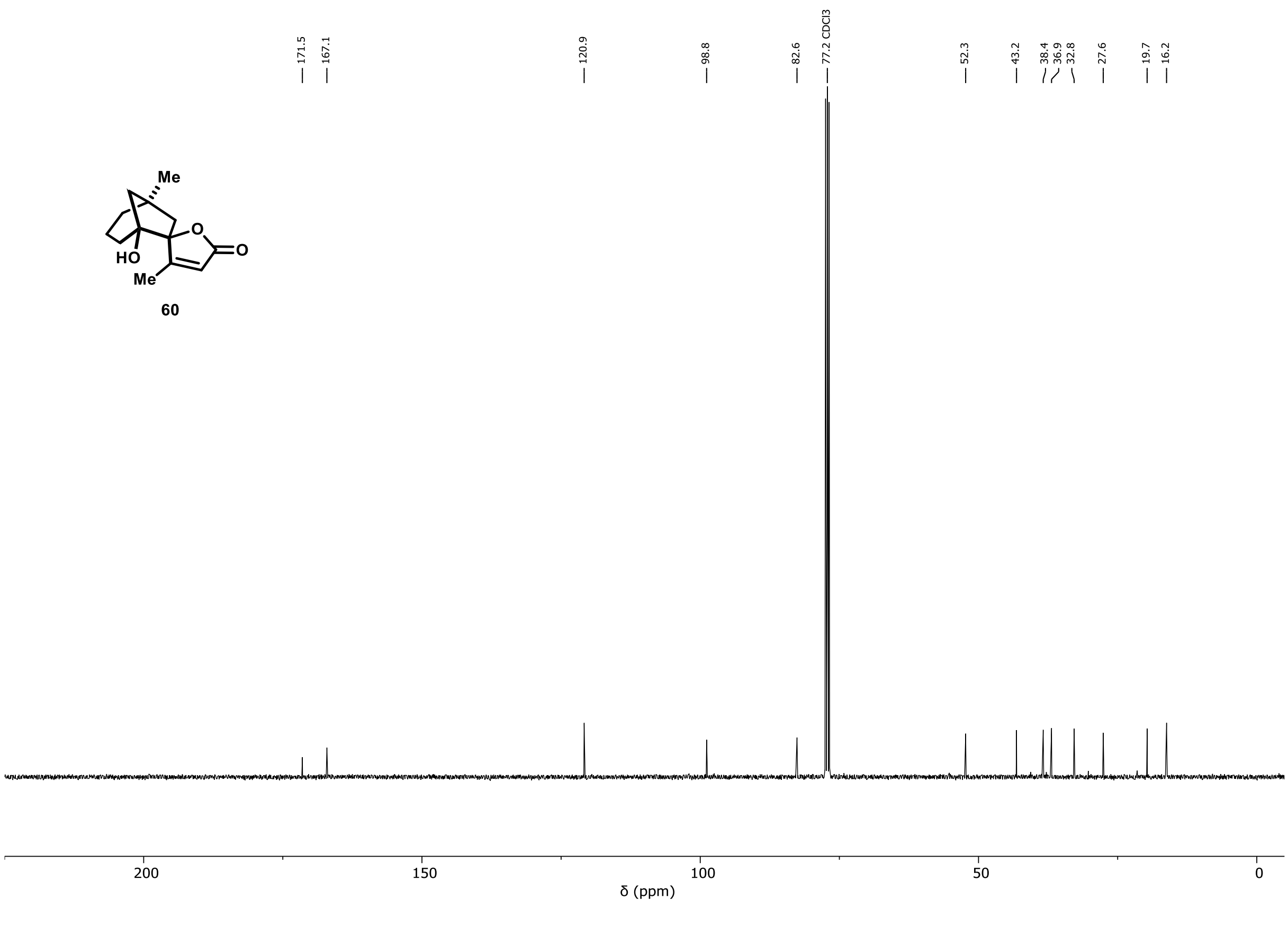
171.5 167.1
120.9
98.8
82.6
77.2 CDCl3
52.3
43.2
38.4 36.9 32.8
27.6
19.7 16.2
Me
O
HO
O
Me
60
200 150 100 50 0
δ (ppm)

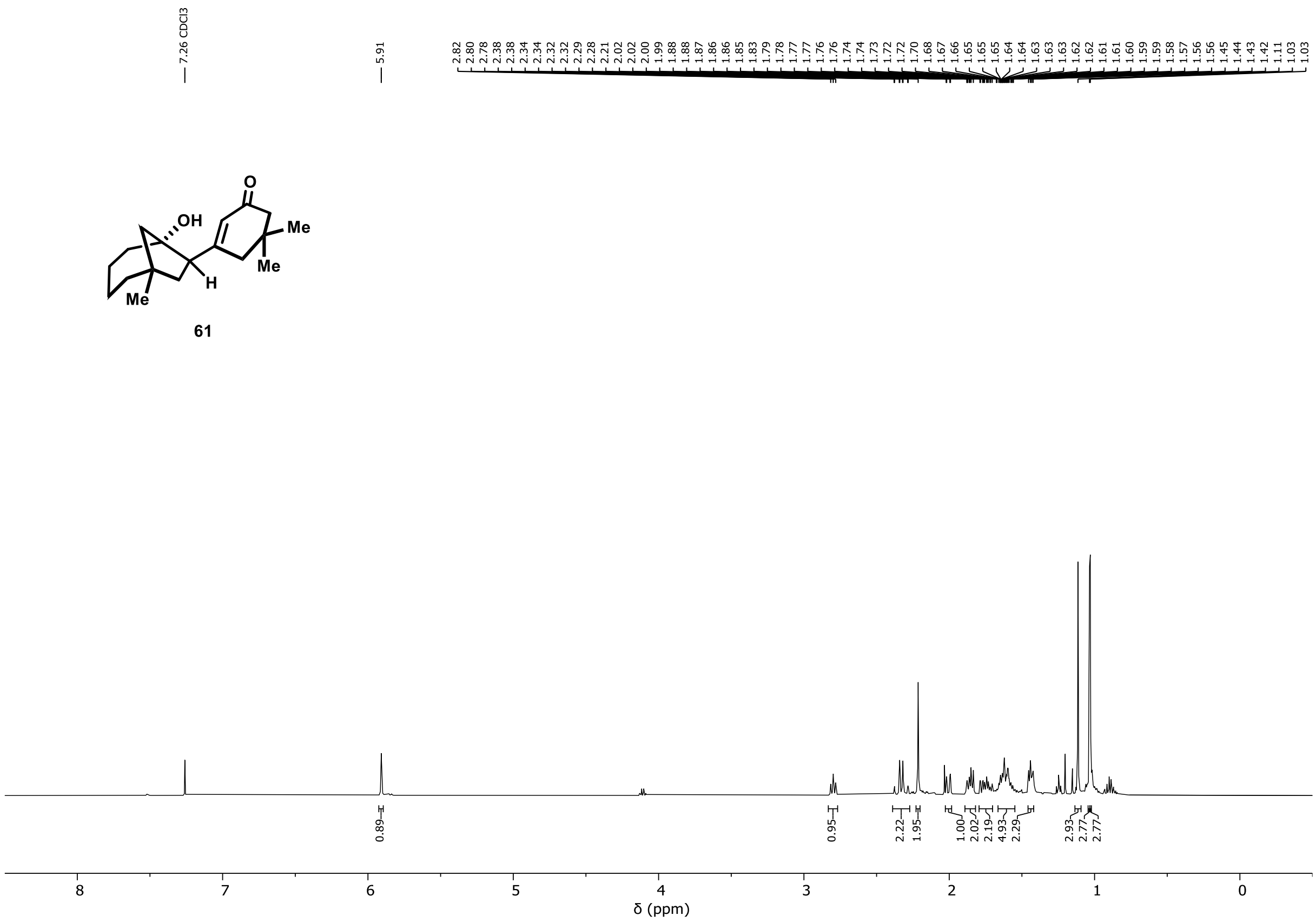
7.26 CDCl3
5.91
2.82 2.80 2.78 2.38 2.38 2.34 2.34 2.32 2.32 2.29 2.28 2.21 2.02 2.02 2.00 1.99 1.88 1.88 1.87 1.86 1.86 1.85 1.83 1.79 1.78 1.77 1.77 1.76 1.74 1.74 1.73 1.72 1.72 1.70 1.68 1.67 1.66 1.65 1.65 1.65 1.64 1.64 1.63 1.63 1.62 1.62 1.61 1.61 1.60 1.59 1.59 1.58 1.57 1.56 1.56 1.45 1.44 1.43 1.42 1.11 1.03 1.03
O
OH
Me
Me
H
Me
61
0.89
0.95
2.22
1.95
1.00
2.02
2.19
4.93
2.29
2.93
2.77
2.77
8 7 6 5 4 3 2 1 0
δ (ppm)

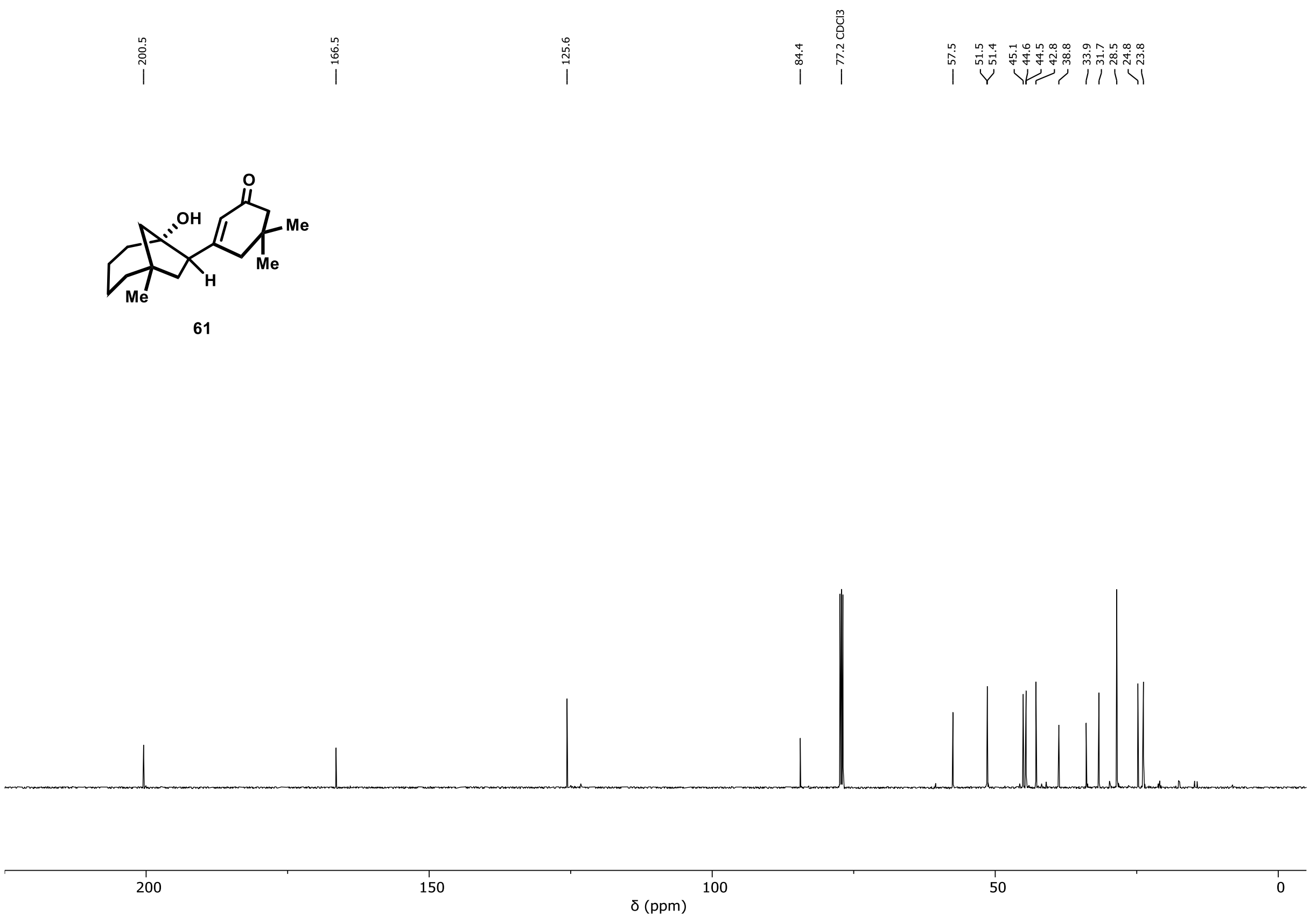
200.5
166.5
125.6
84.4
77.2 CDCl3
57.5
51.5
51.4
45.1
44.6
44.5
42.8
38.8
33.9
31.7
28.5
24.8
23.8
O
OH
Me
Me
H
Me
61
200 150 100 50 0
δ (ppm)

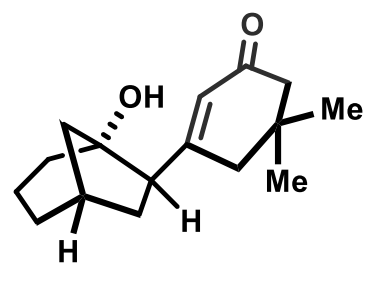

O
OH
Me
Me
H
H

62

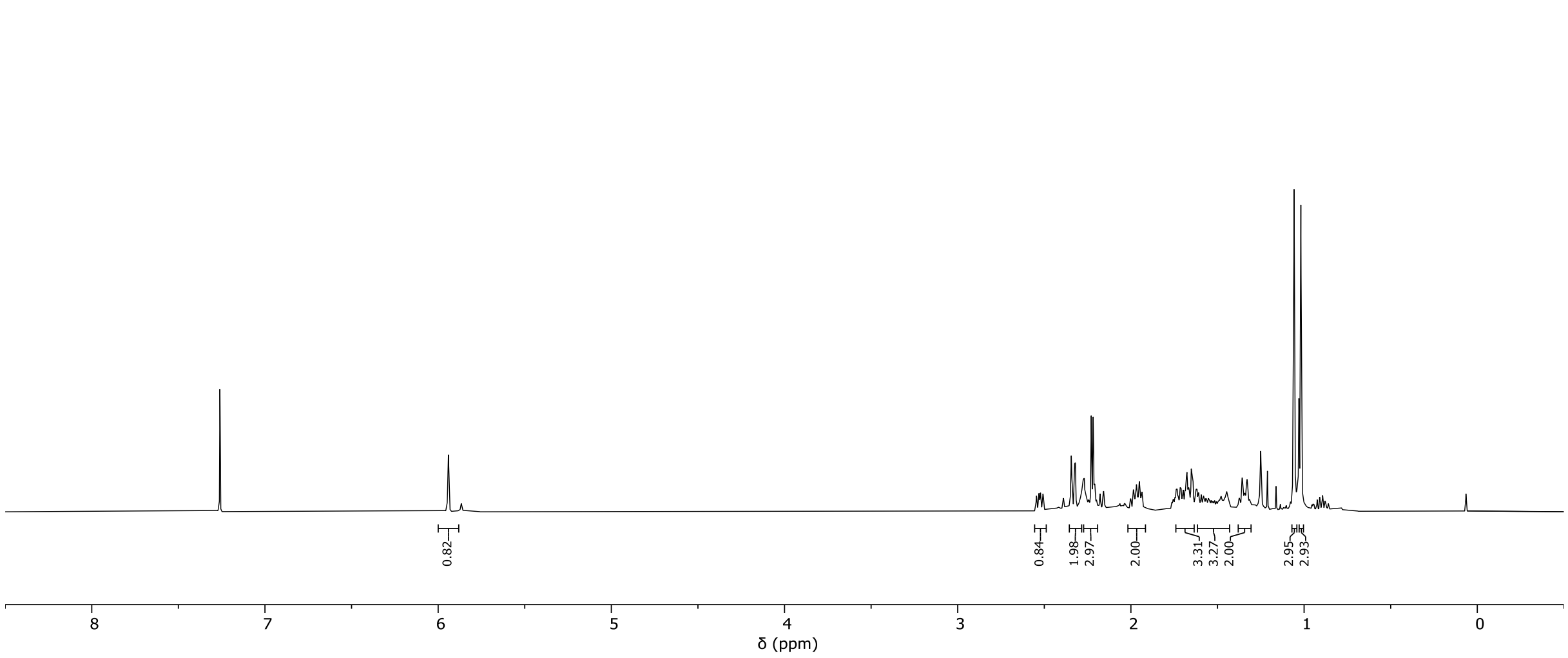

7.26 CDCl3
5.94 2.54 2.53 2.52 2.51 2.35 2.34 2.32 2.32 2.28 2.28 2.27 2.23 2.22 2.21 2.00 2.00 1.99 1.98 1.97 1.97 1.97 1.96 1.95 1.95 1.95 1.94 1.93 1.76 1.75 1.74 1.73 1.73 1.72 1.71 1.70 1.69 1.68 1.68 1.67 1.67 1.65 1.64 1.63 1.62 1.62 1.61 1.61 1.59 1.58 1.57 1.56 1.55 1.54 1.53 1.52 1.51 1.48 1.46 1.45 1.44 1.38 1.37 1.37 1.37 1.36 1.35 1.34 1.34 1.33 1.33 1.33 1.32 1.30 1.06 1.02
0.82
0.84
1.98
2.97
2.00
3.31
3.27
2.00
2.95
2.93
δ (ppm)

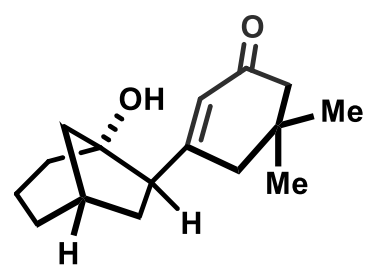

O
OH
Me
Me
H
H

62

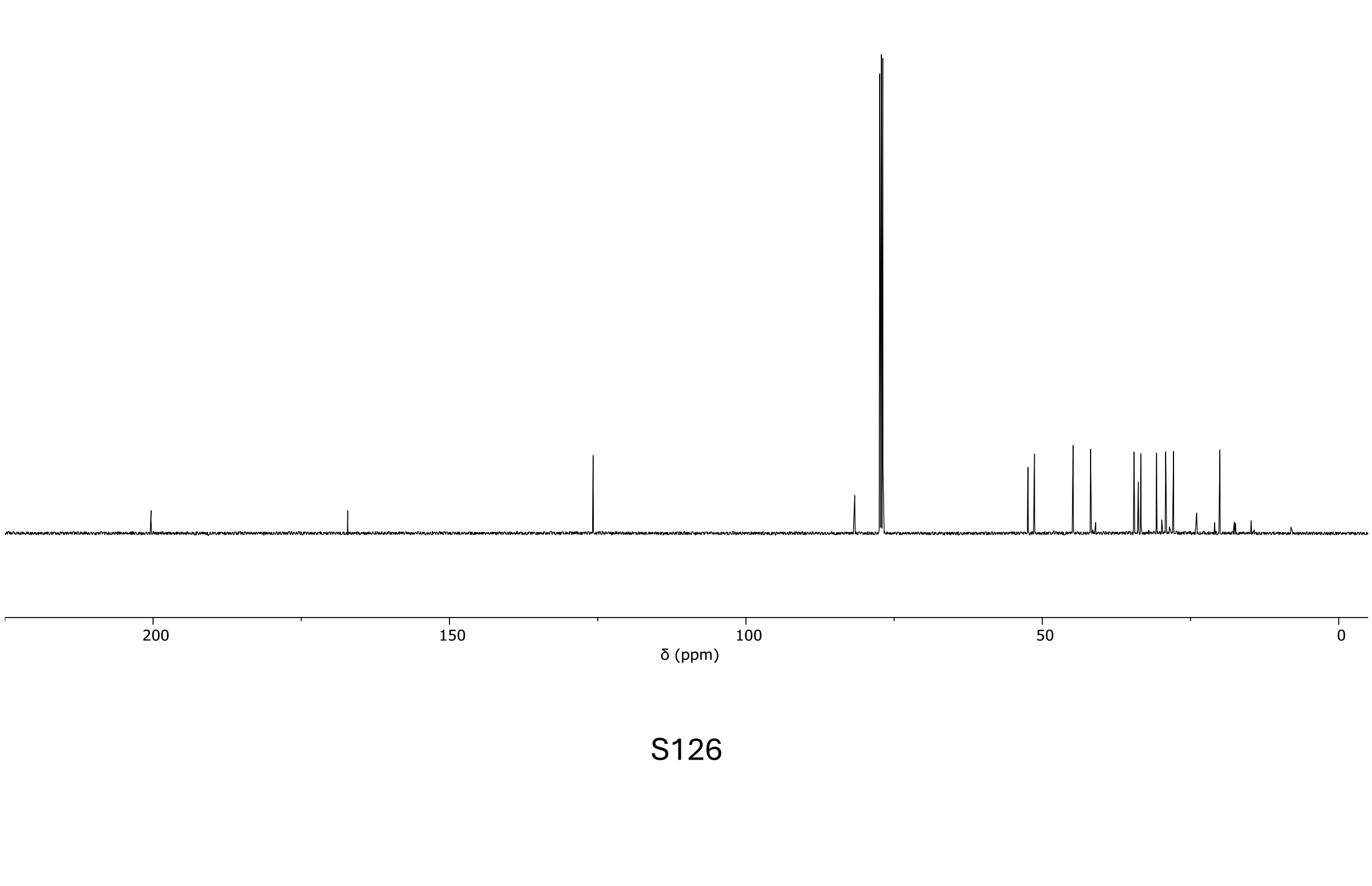

200.3
167.2
125.8
81.6
77.2 CDCl3
52.4
51.3
44.8
44.8
41.9
34.5
33.8
33.4
30.8
29.2
27.9
20.0
δ (ppm)

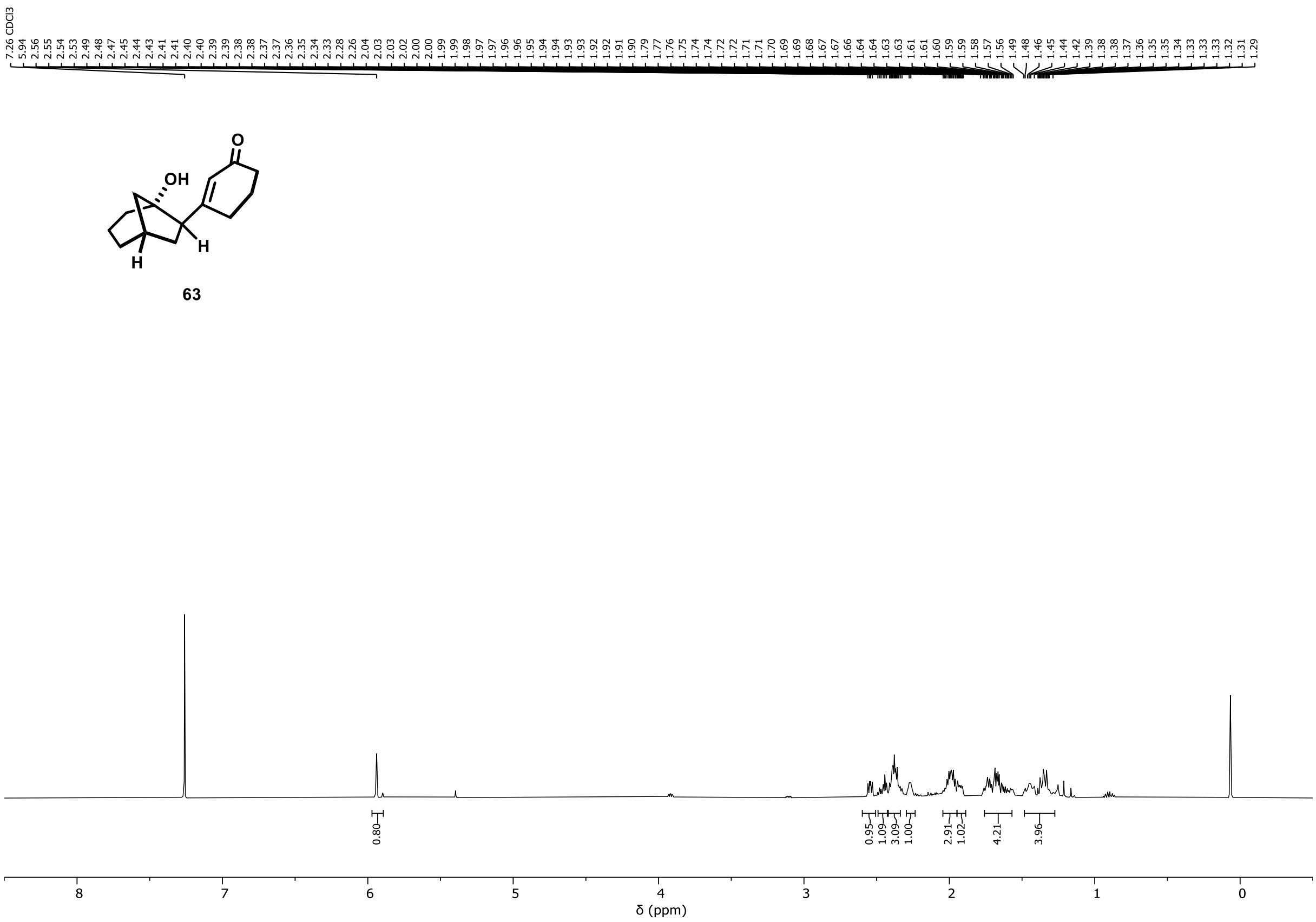

7.26 CDCl3
5.94
2.56 2.55 2.54 2.53 2.49 2.48 2.47 2.45 2.44 2.43 2.41 2.41 2.40 2.40 2.39 2.39 2.38 2.38 2.37 2.37 2.36 2.35 2.34 2.33 2.28 2.26 2.04 2.03 2.03 2.02 2.00 2.00 1.99 1.99 1.98 1.97 1.97 1.96 1.96 1.95 1.94 1.94 1.93 1.93 1.92 1.92 1.91 1.90 1.79 1.77 1.76 1.75 1.74 1.74 1.72 1.72 1.71 1.71 1.70 1.69 1.69 1.68 1.67 1.67 1.66 1.64 1.64 1.63 1.63 1.61 1.61 1.60 1.59 1.59 1.58 1.57 1.56 1.49 1.48 1.46 1.45 1.44 1.42 1.39 1.38 1.38 1.37 1.36 1.35 1.35 1.34 1.33 1.33 1.32 1.31 1.29
OH
O
H
H
63
0.80
0.95 1.09 3.09 1.00 2.91 1.02 4.21 3.96
8 7 6 5 4 3 2 1 0
δ (ppm)

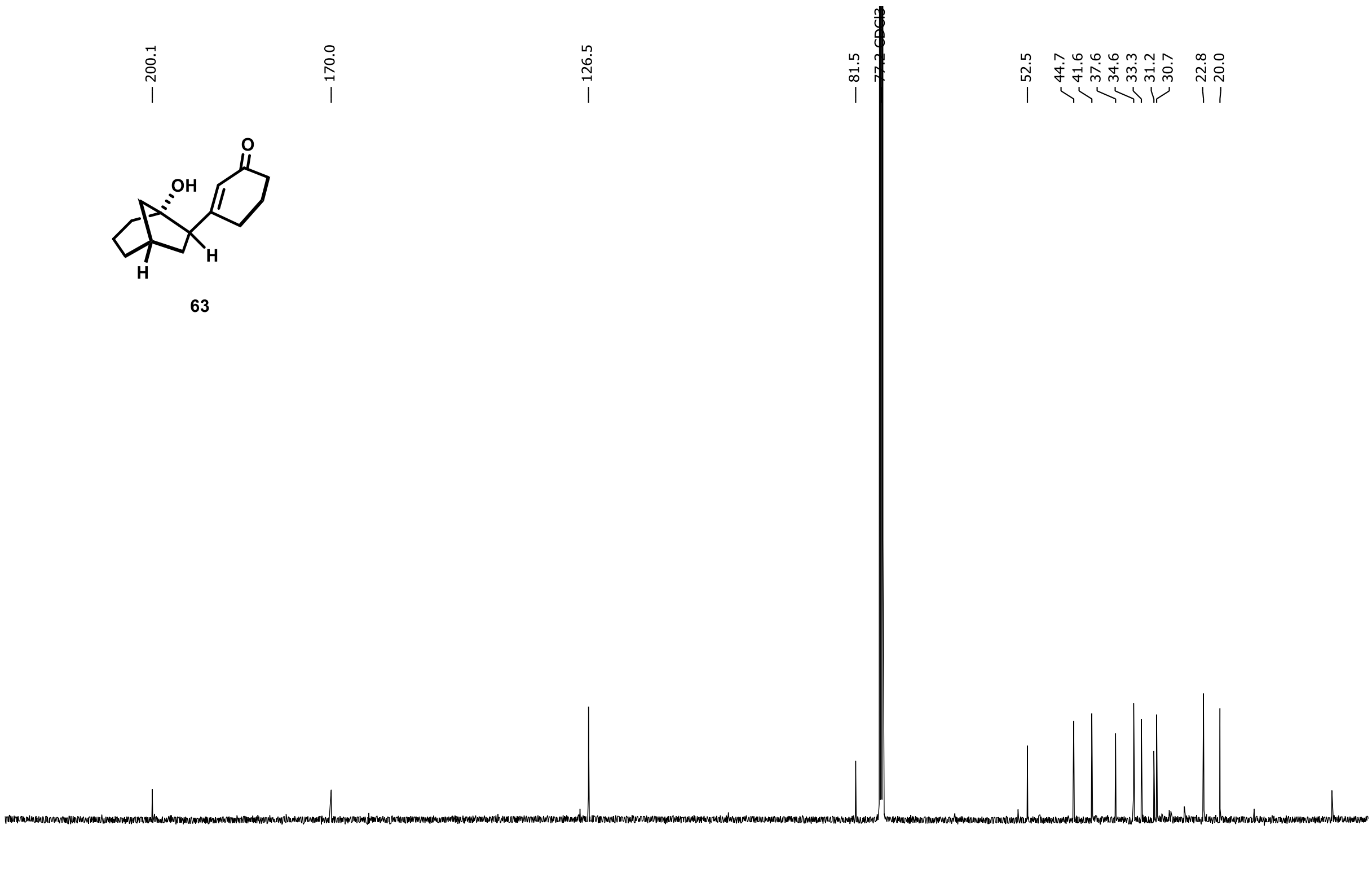

200.1
170.0
126.5
81.5
77.2 CDCl3
52.5 44.7 41.6 37.6 34.6 33.3 31.2 30.7 22.8 20.0
OH
O
H
H
63
200 150 100 50 0
δ (ppm)

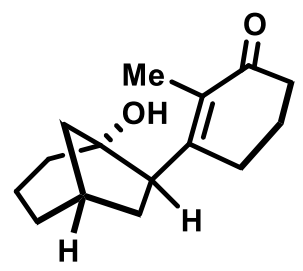
Me
OH
O
H
H

64

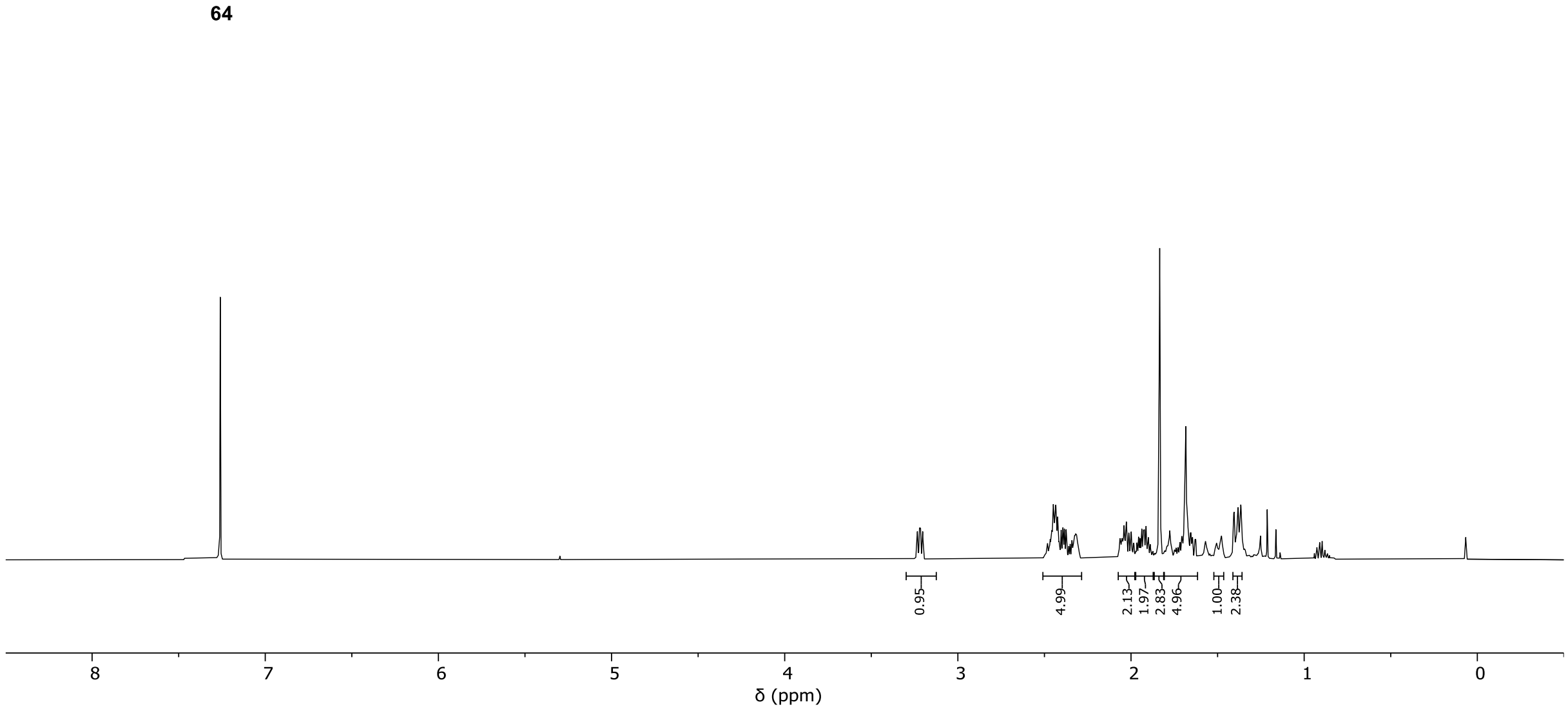
7.26 CDCl3
3.23
3.22
3.22
3.20
2.48
2.47
2.47
2.46
2.46
2.45
2.44
2.44
2.44
2.43
2.43
2.42
2.42
2.40
2.39
2.39
2.38
2.37
2.37
2.35
2.34
2.33
2.33
2.32
2.31
2.31
2.06
2.06
2.05
2.05
2.04
2.04
2.03
2.03
2.01
2.01
2.00
2.00
1.99
1.98
1.97
1.96
1.95
1.95
1.94
1.94
1.93
1.93
1.92
1.92
1.91
1.91
1.90
1.90
1.89
1.84
1.83
1.83
1.79
1.78
1.78
1.72
1.71
1.70
1.70
1.69
1.69
1.68
1.68
1.67
1.67
1.66
1.66
1.65
1.65
1.64
1.63
1.63
1.51
1.51
1.50
1.48
1.48
1.48
1.41
1.40
1.39
1.39
1.39
1.38
1.37
0.95
4.99
2.13
1.97
2.83
4.96
1.00
2.38
8
7
6
5
4
3
2
1
0
δ (ppm)

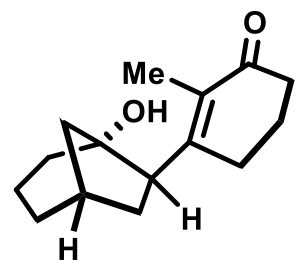
Me
OH
O
H
H

64

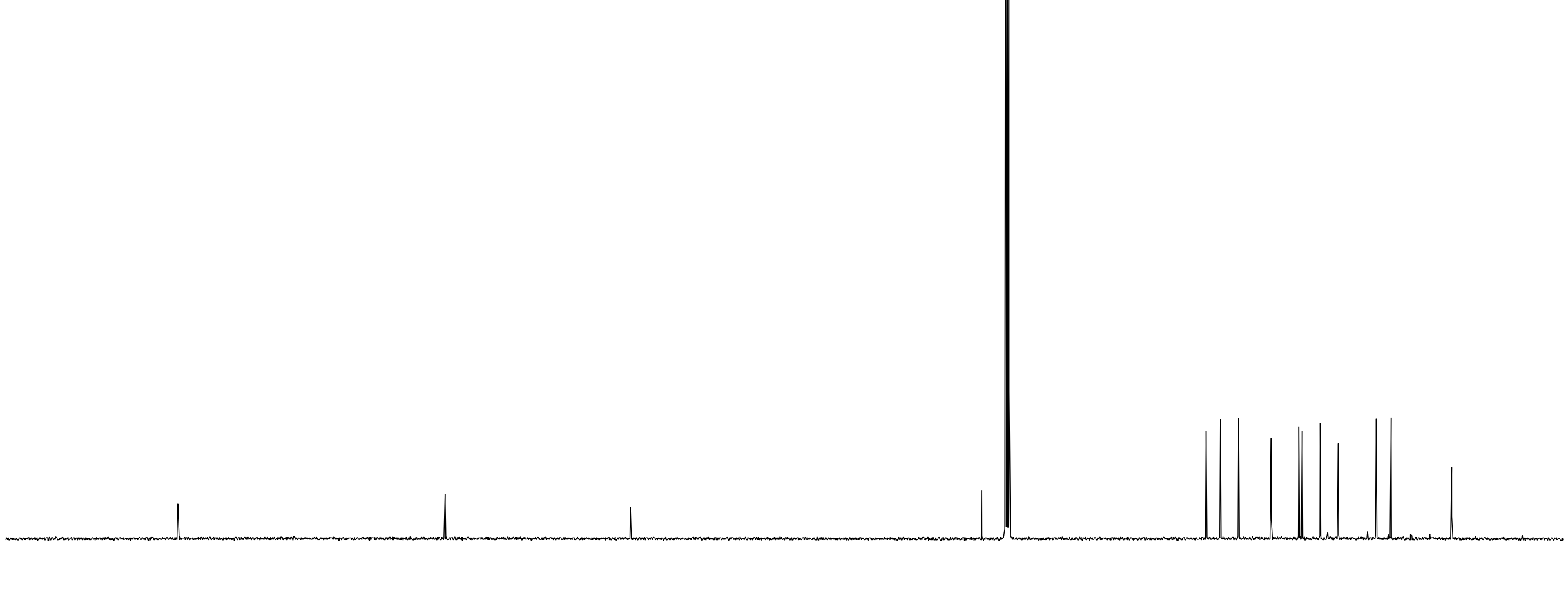
199.6
160.1
132.8
80.9
77.2 CDCl3
47.8
45.7
43.0
38.2
34.1
33.6
31.0
28.3
22.7
20.5
11.6
200
150
100
50
0
δ (ppm)

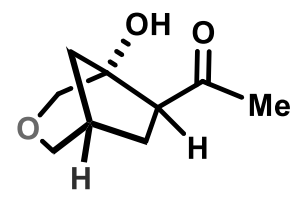

53

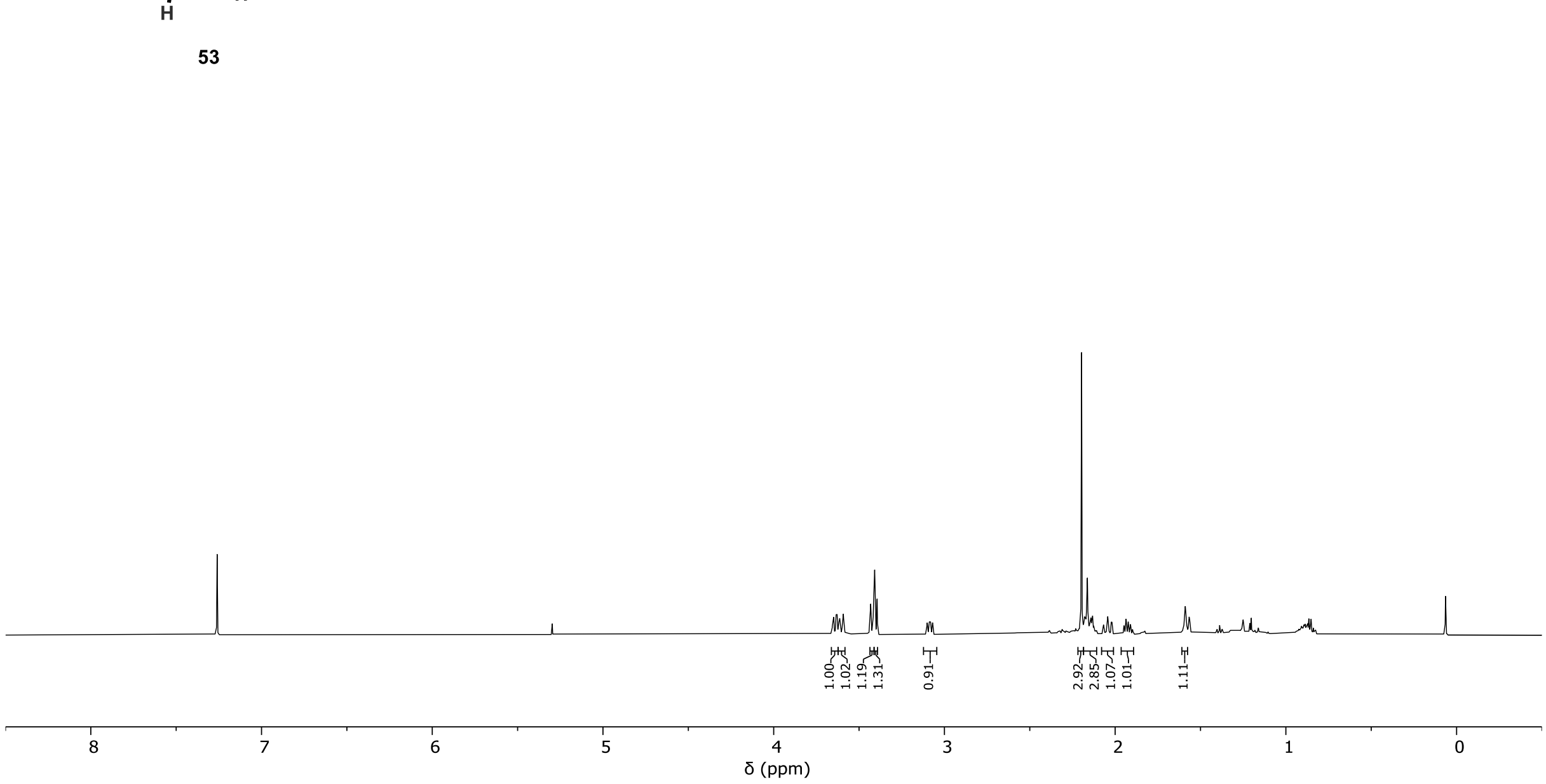

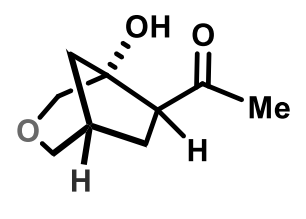

53

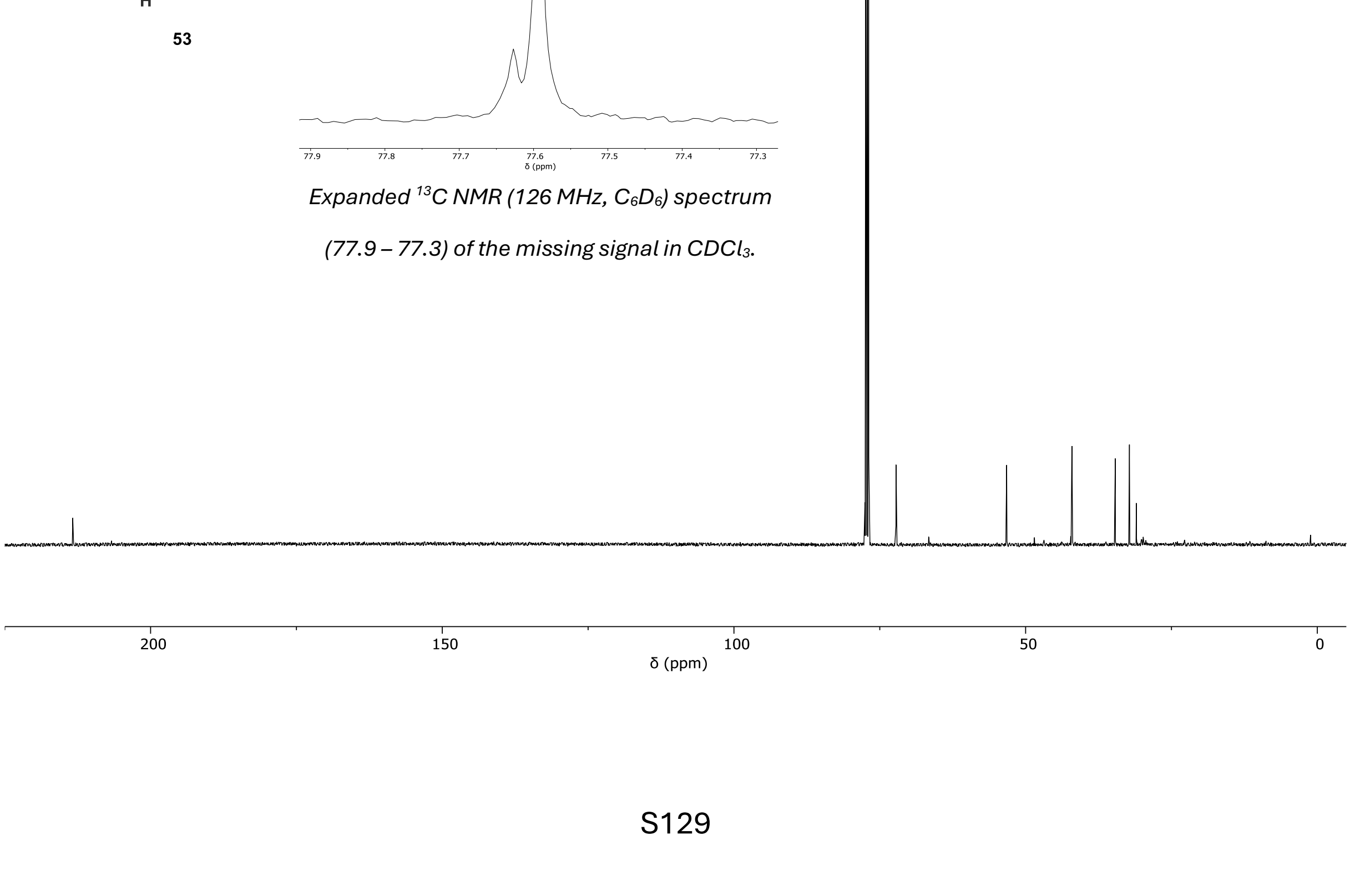

Expanded ^{13}C NMR (126 MHz, C_6D_6) spectrum (77.9 – 77.3) of the missing signal in $CDCl_3$.

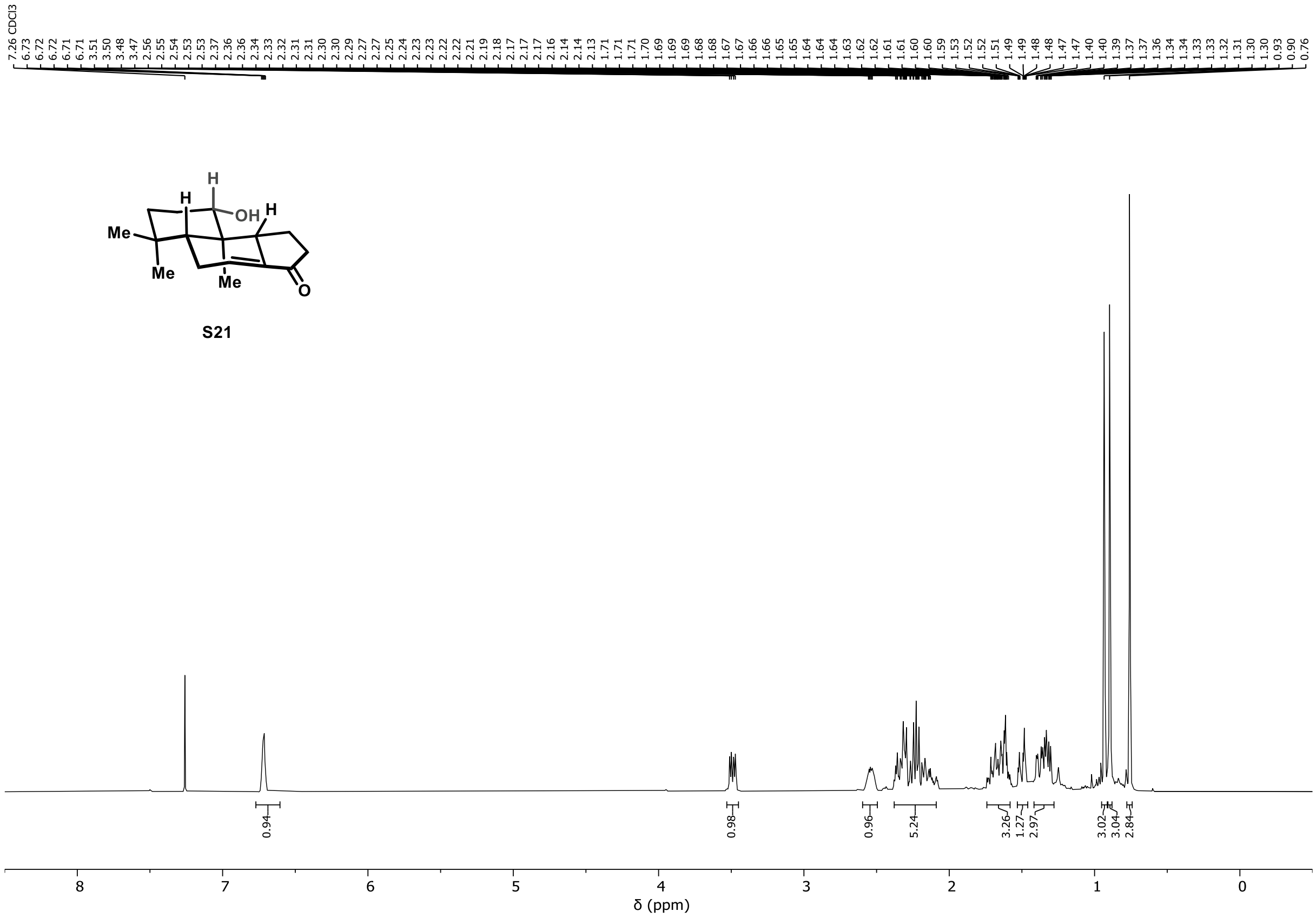
7.26 CDCl3
6.73
6.72
6.72
6.71
6.71
3.51
3.50
3.48
3.47
2.56
2.55
2.54
2.53
2.53
2.37
2.36
2.34
2.33
2.32
2.31
2.31
2.30
2.30
2.29
2.27
2.27
2.25
2.24
2.23
2.23
2.22
2.22
2.21
2.19
2.18
2.17
2.17
2.17
2.16
2.14
2.14
2.13
1.71
1.71
1.71
1.70
1.69
1.69
1.68
1.67
1.67
1.66
1.66
1.65
1.64
1.64
1.63
1.62
1.62
1.61
1.61
1.60
1.60
1.59
1.53
1.52
1.52
1.51
1.49
1.49
1.48
1.48
1.47
1.47
1.40
1.40
1.39
1.37
1.37
1.36
1.34
1.34
1.33
1.33
1.32
1.31
1.30
0.93
0.90
0.76
H
H
OH
H
Me
Me
Me
O
S21
0.94
0.98
0.96
5.24
3.26
1.27
2.97
3.02
3.04
2.84
8
7
6
5
4
3
2
1
0
δ (ppm)

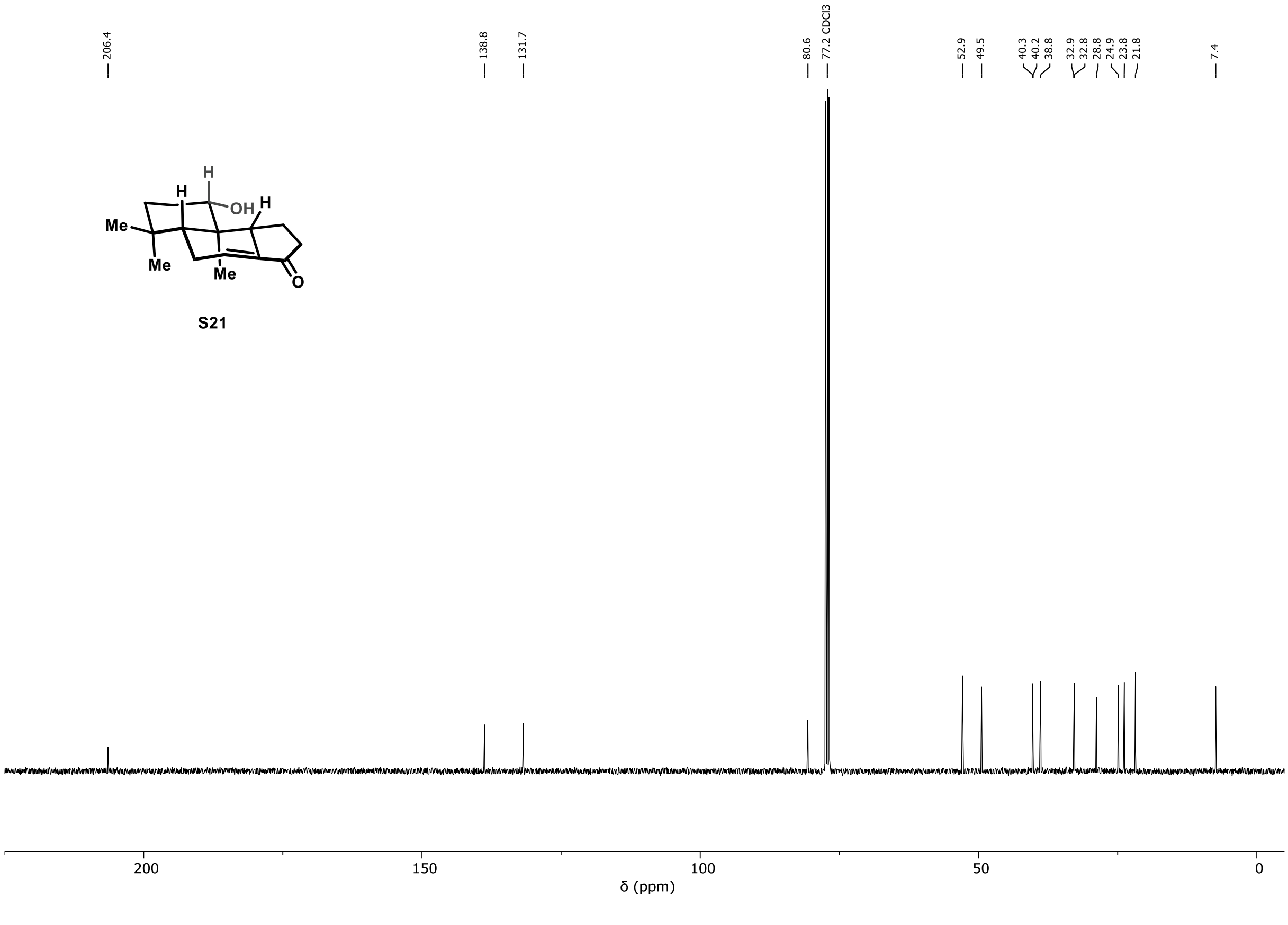
206.4
138.8
131.7
80.6
77.2 CDCl3
52.9
49.5
40.3
40.2
38.8
32.9
32.8
28.8
24.9
23.8
21.8
7.4
H
H
OH
H
Me
Me
Me
O
S21
200
150
100
50
0
δ (ppm)

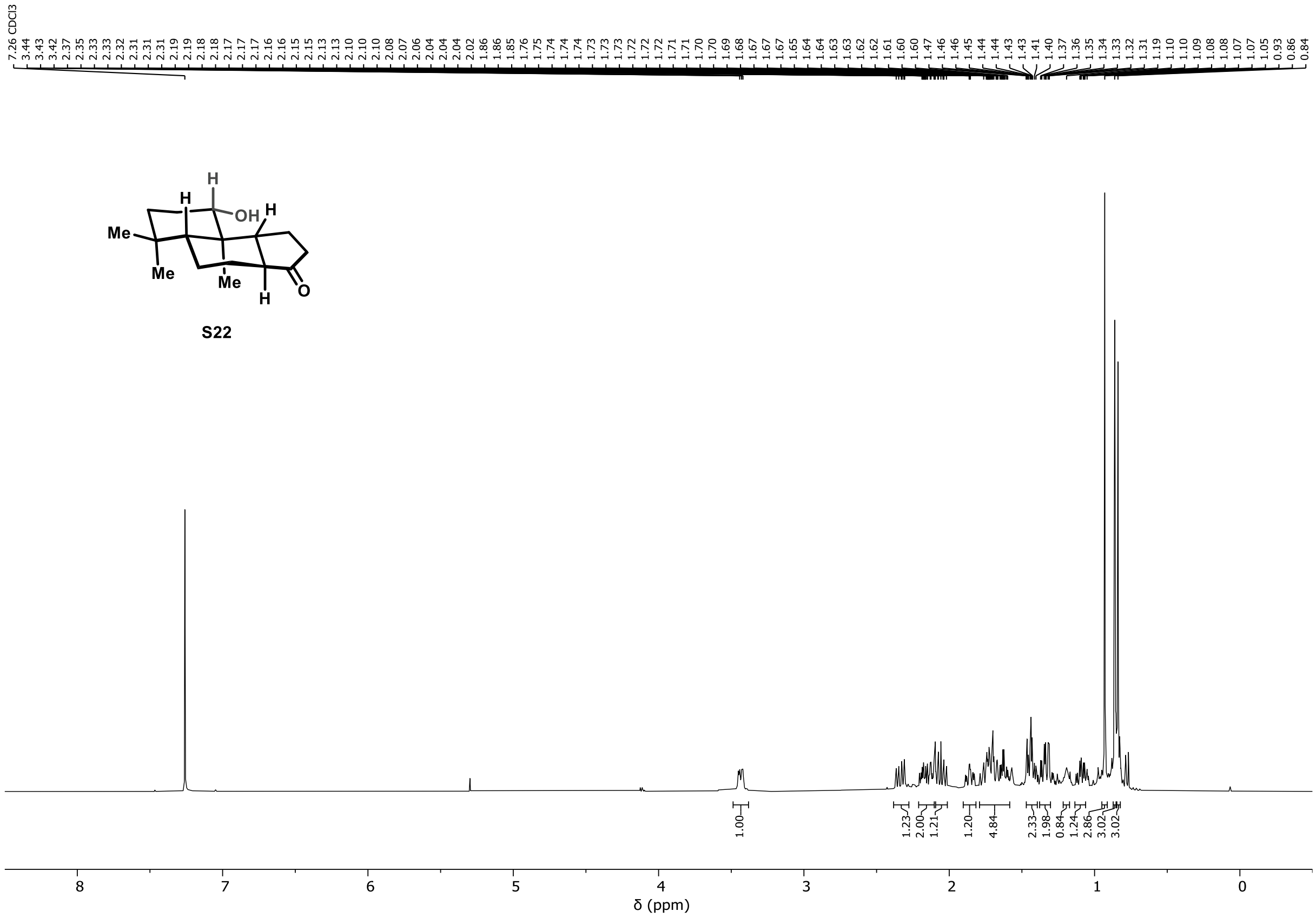
7.26 CDCl3
3.44
3.43
3.42
2.37
2.35
2.33
2.33
2.32
2.31
2.31
2.31
2.19
2.19
2.18
2.18
2.17
2.17
2.17
2.16
2.16
2.15
2.15
2.13
2.13
2.10
2.10
2.10
2.08
2.07
2.06
2.04
2.04
2.04
2.02
1.86
1.86
1.85
1.76
1.75
1.74
1.74
1.73
1.73
1.73
1.72
1.72
1.72
1.71
1.71
1.70
1.69
1.68
1.67
1.67
1.65
1.64
1.63
1.63
1.62
1.61
1.60
1.60
1.47
1.46
1.46
1.45
1.44
1.44
1.43
1.43
1.41
1.40
1.37
1.36
1.35
1.34
1.33
1.32
1.31
1.19
1.10
1.10
1.09
1.08
1.08
1.07
1.07
1.05
0.93
0.86
0.84
H
H
OH
H
Me
Me
Me
H
O
S22
1.00
1.23
2.00
1.21
1.20
4.84
2.33
1.98
0.84
1.24
2.86
3.02
3.02
8
7
6
5
4
3
2
1
0
δ (ppm)

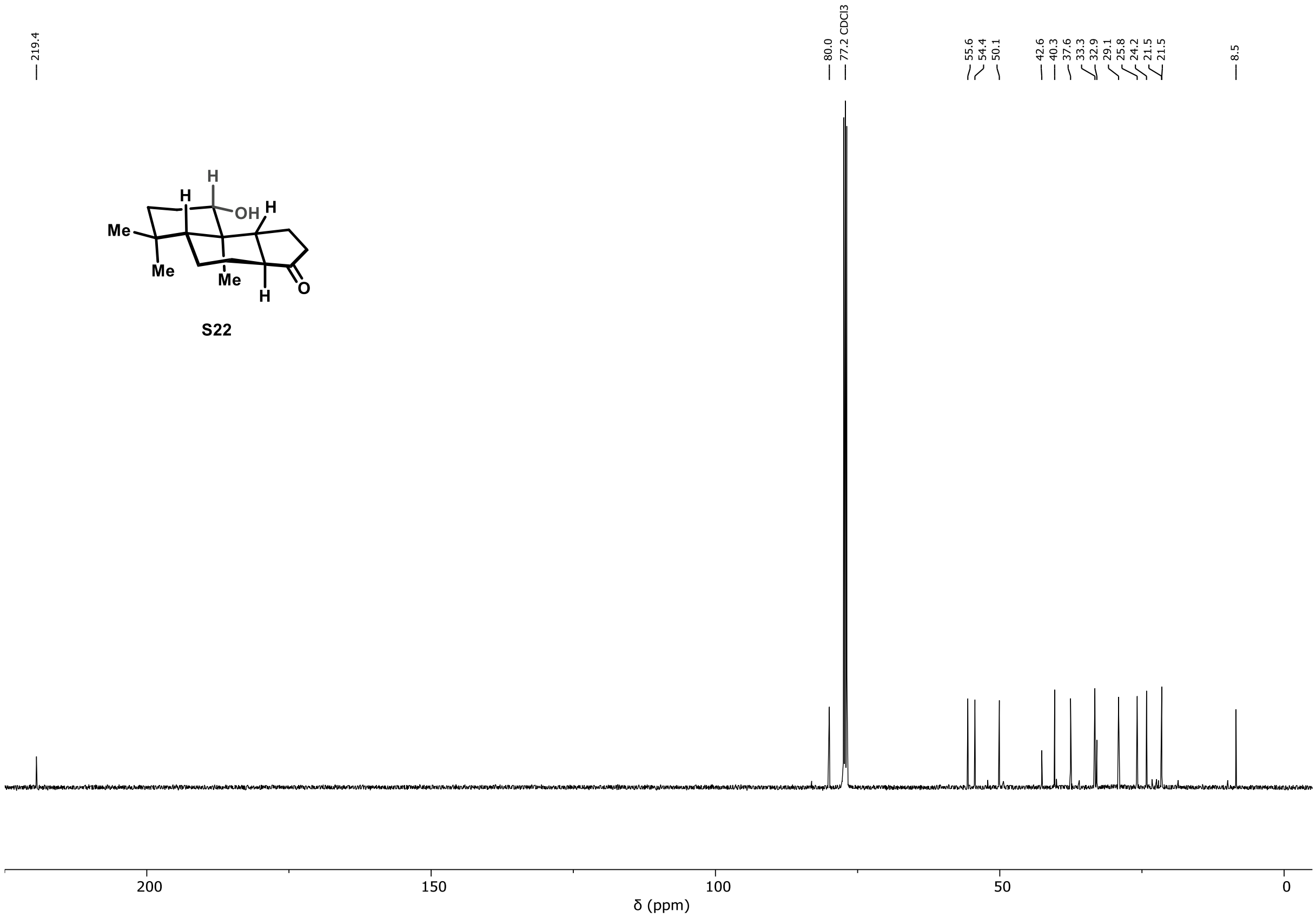
219.4
80.0
77.2 CDCl3
55.6
54.4
50.1
42.6
40.3
37.6
33.3
32.9
29.1
25.8
24.2
21.5
21.5
8.5
H
H
OH
H
Me
Me
Me
H
O
S22
200
150
100
50
0
δ (ppm)

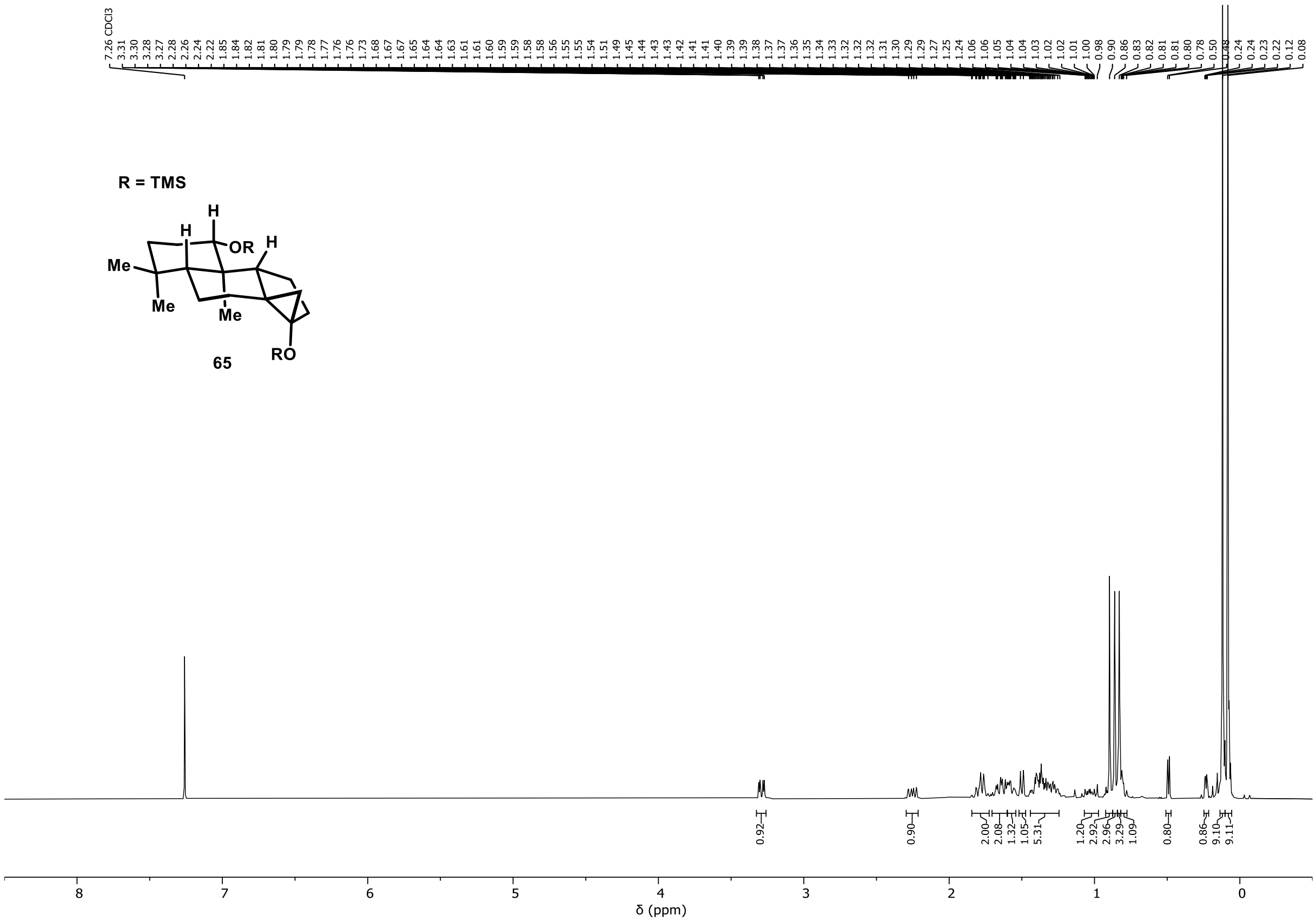
7.26 CDCl3
R = TMS
H
H
OR
H
Me
Me
Me
RO
65
δ (ppm)

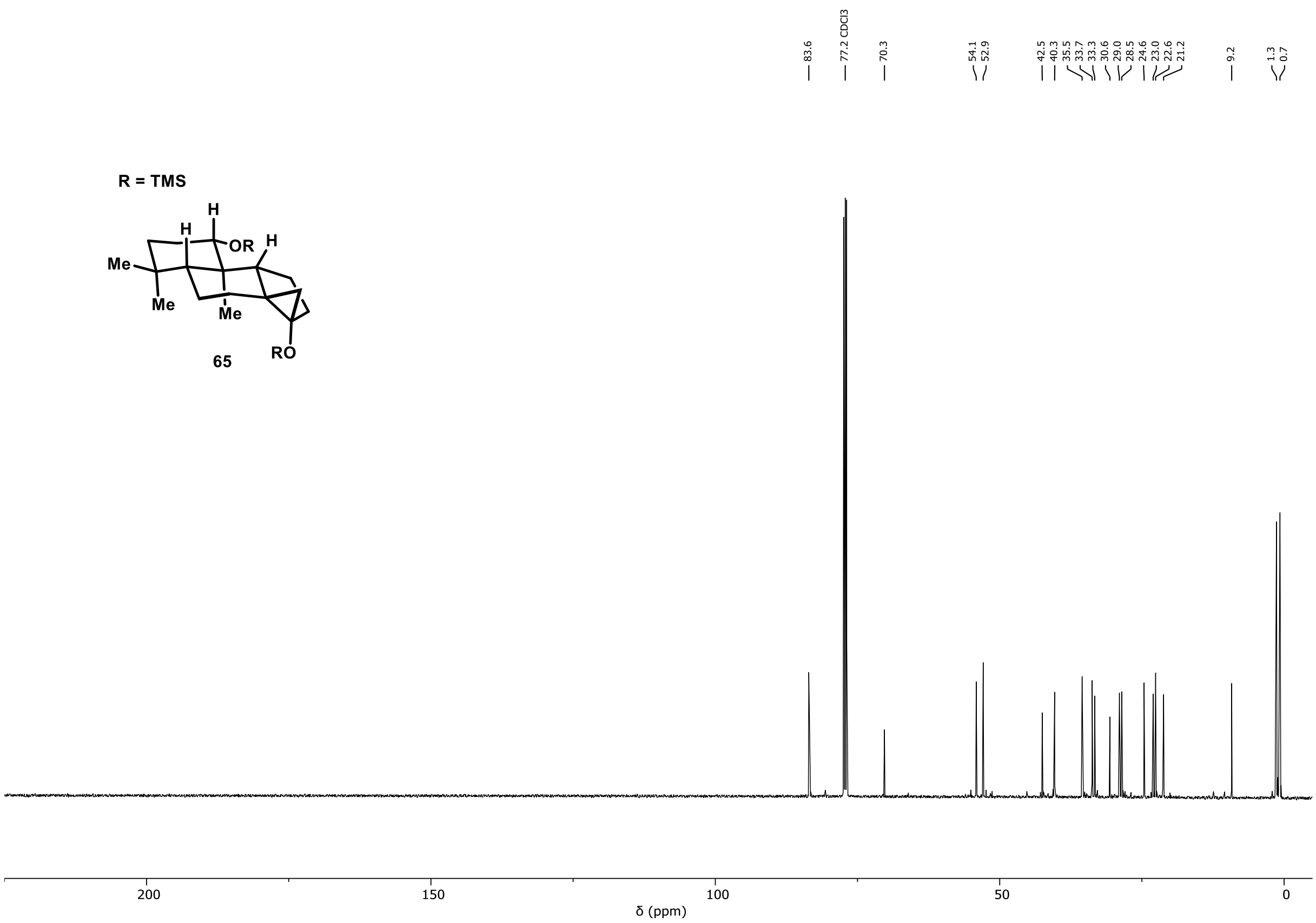
83.6
77.2 CDCl3
70.3
54.1
52.9
42.5
40.3
35.5
33.7
33.3
30.6
29.0
28.5
24.6
23.0
22.6
21.2
9.2
1.3
0.7
R = TMS
H
H
OR
H
Me
Me
Me
RO
65
δ (ppm)

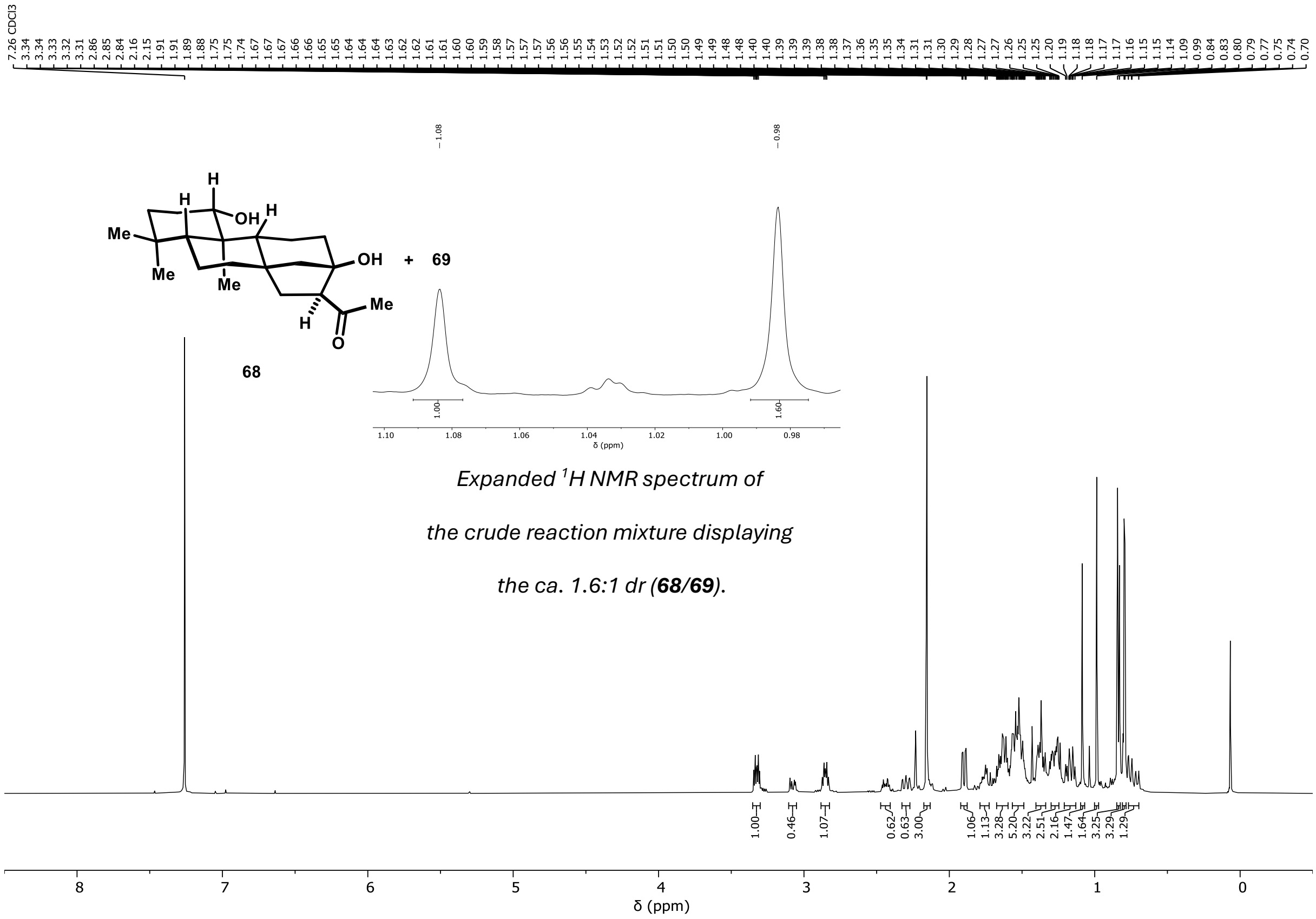

Me
Me
Me
H
H
OH
H
OH
+ 69
H
O
Me
68
Expanded ^{1}H NMR spectrum of
the crude reaction mixture displaying
the ca. 1.6:1 dr (**68**/**69**).
δ (ppm)

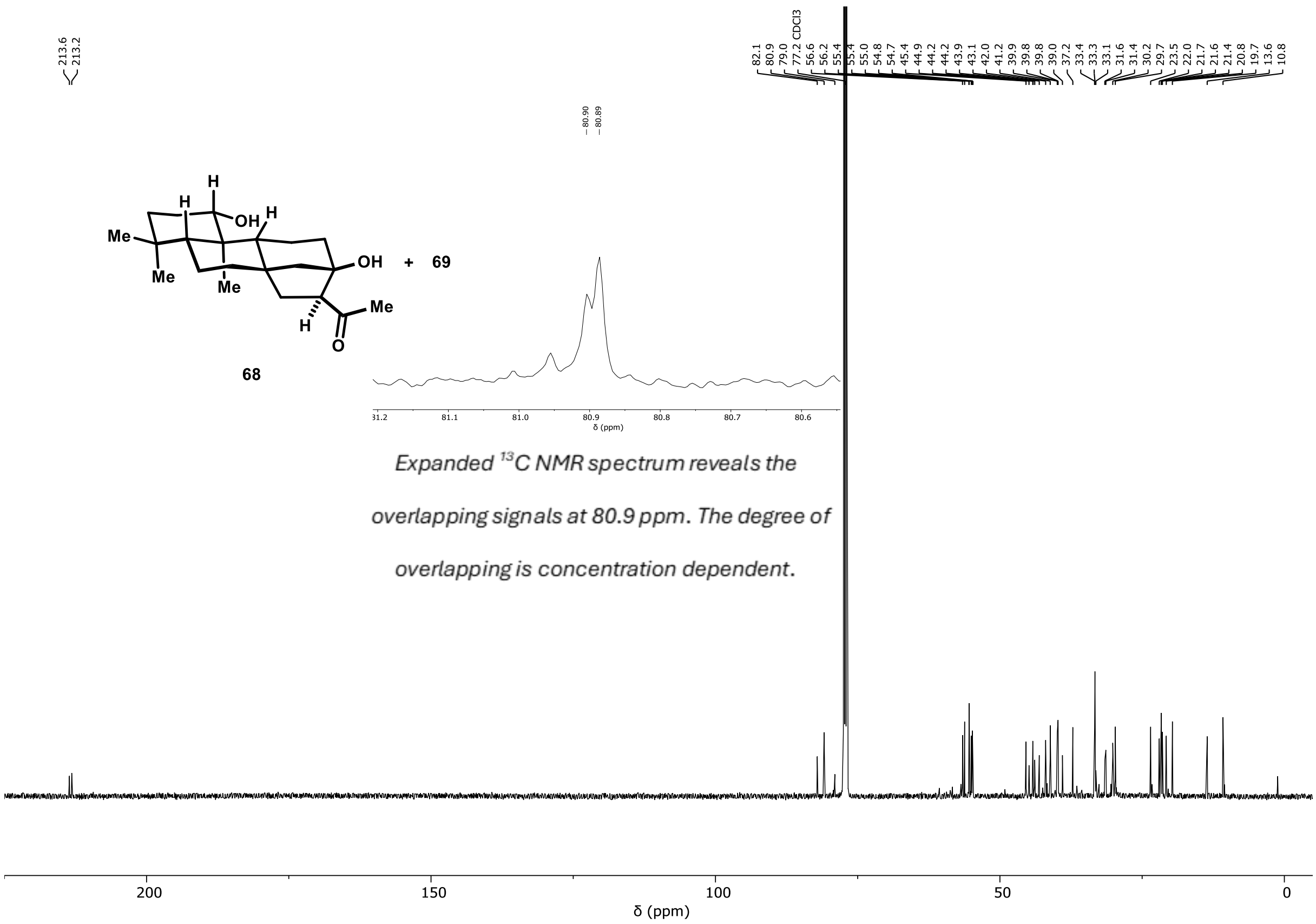

Me
Me
Me
H
H
OH
H
OH
+ 69
H
O
Me
68
Expanded ^{13}C NMR spectrum reveals the
overlapping signals at 80.9 ppm. The degree of
overlapping is concentration dependent.
δ (ppm)

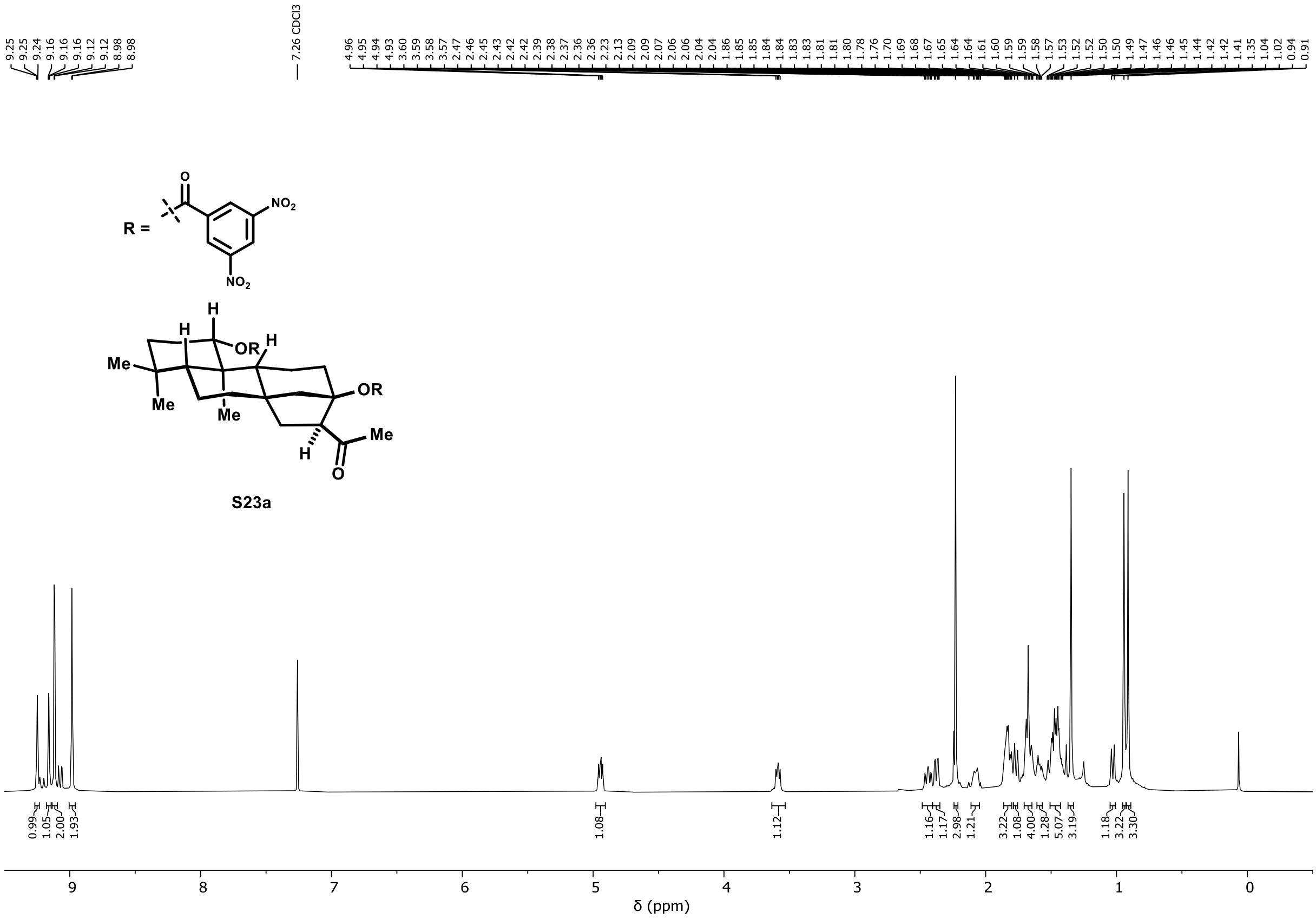
9.25 9.25 9.24 9.16 9.16 9.16 9.12 9.12 8.98 8.98
7.26 CDCl3
4.96 4.95 4.94 4.93 3.60 3.59 3.58 3.57 2.47 2.46 2.45 2.43 2.42 2.39 2.38 2.37 2.36 2.36 2.23 2.13 2.09 2.09 2.07 2.06 2.04 2.04 1.86 1.85 1.85 1.84 1.84 1.83 1.83 1.81 1.81 1.80 1.78 1.76 1.70 1.69 1.68 1.67 1.65 1.64 1.64 1.61 1.60 1.59 1.59 1.58 1.57 1.53 1.52 1.52 1.50 1.50 1.49 1.47 1.46 1.46 1.45 1.44 1.42 1.42 1.41 1.35 1.04 1.02 0.94 0.91
R =
NO_2
NO_2
O
H
H
OR
H
Me
Me
Me
OR
Me
H
O
S23a
0.99 1.05 2.00 1.93
1.08
1.12
1.16 1.17 2.98 1.21
3.22 1.08 4.00 1.28 5.07 3.19
1.18 3.22 3.30
δ (ppm)

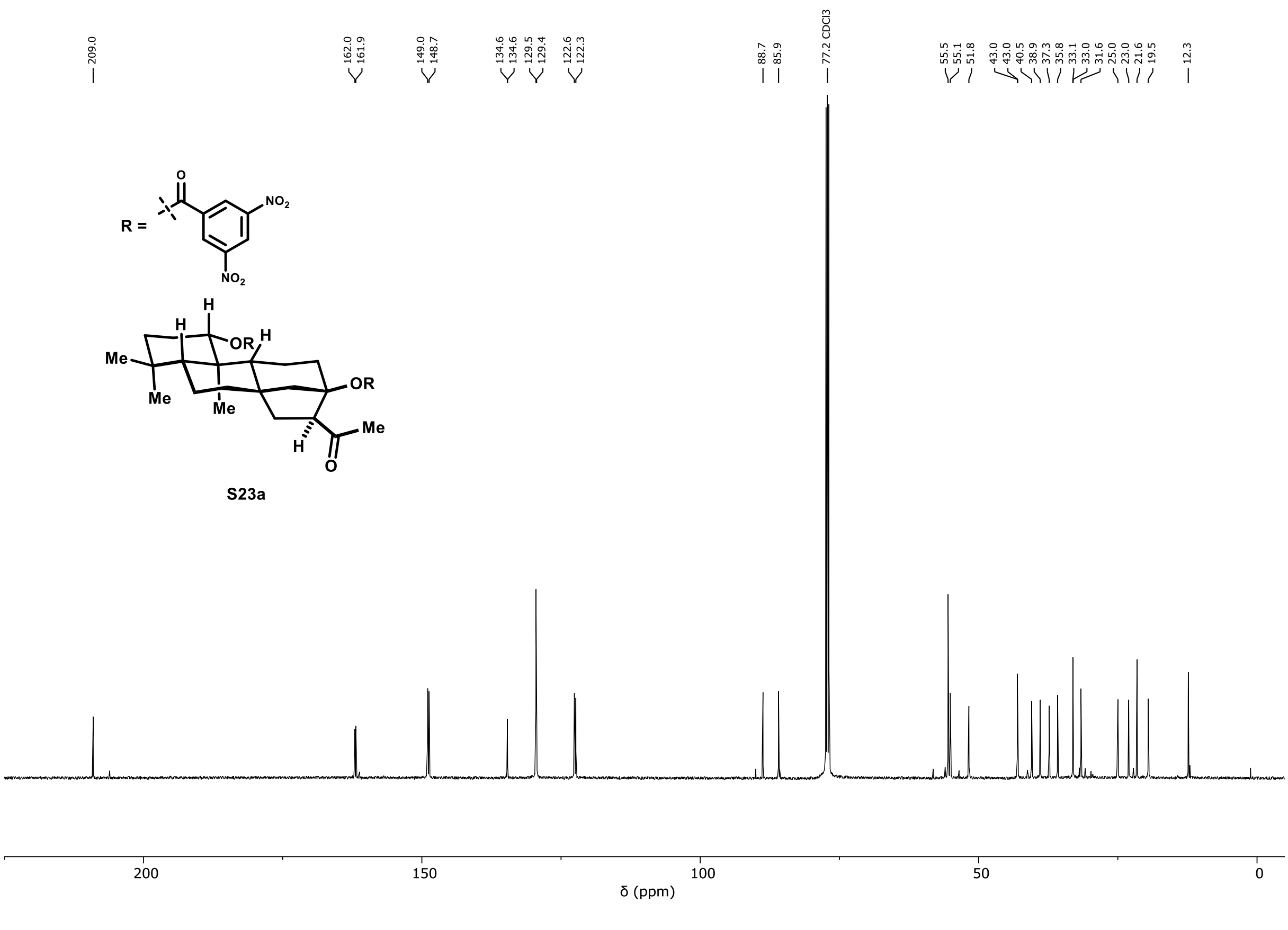
209.0
162.0 161.9
149.0 148.7
134.6 134.6 129.5 129.4
122.6 122.3
88.7 85.9
77.2 CDCl3
55.5 55.1 51.8
43.0 43.0 40.5 38.9 37.3 35.8 33.1 33.0 31.6 25.0 23.0 21.6 19.5
12.3
R =
NO_2
NO_2
O
H
H
OR
H
Me
Me
Me
OR
Me
H
O
S23a
δ (ppm)

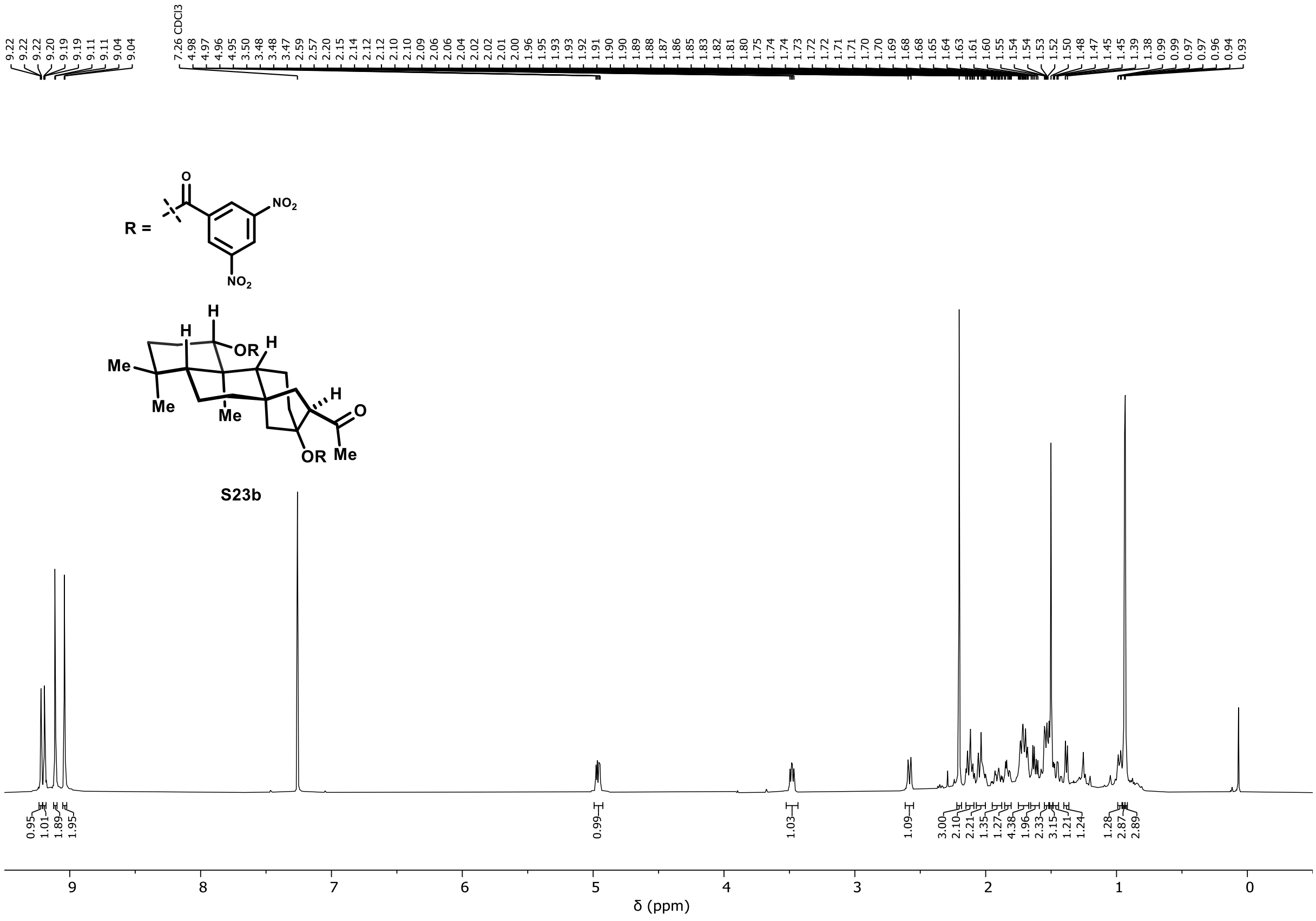

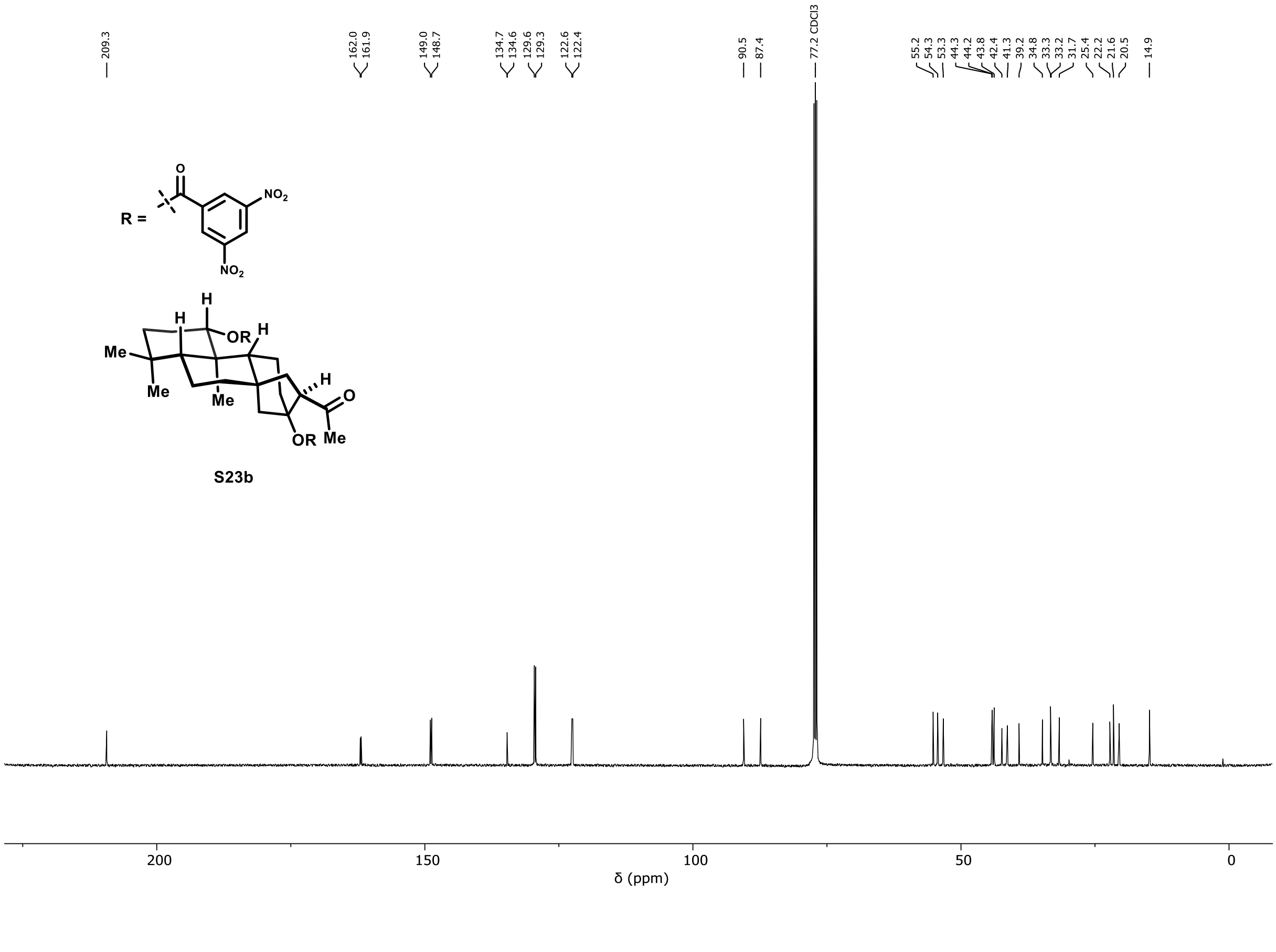

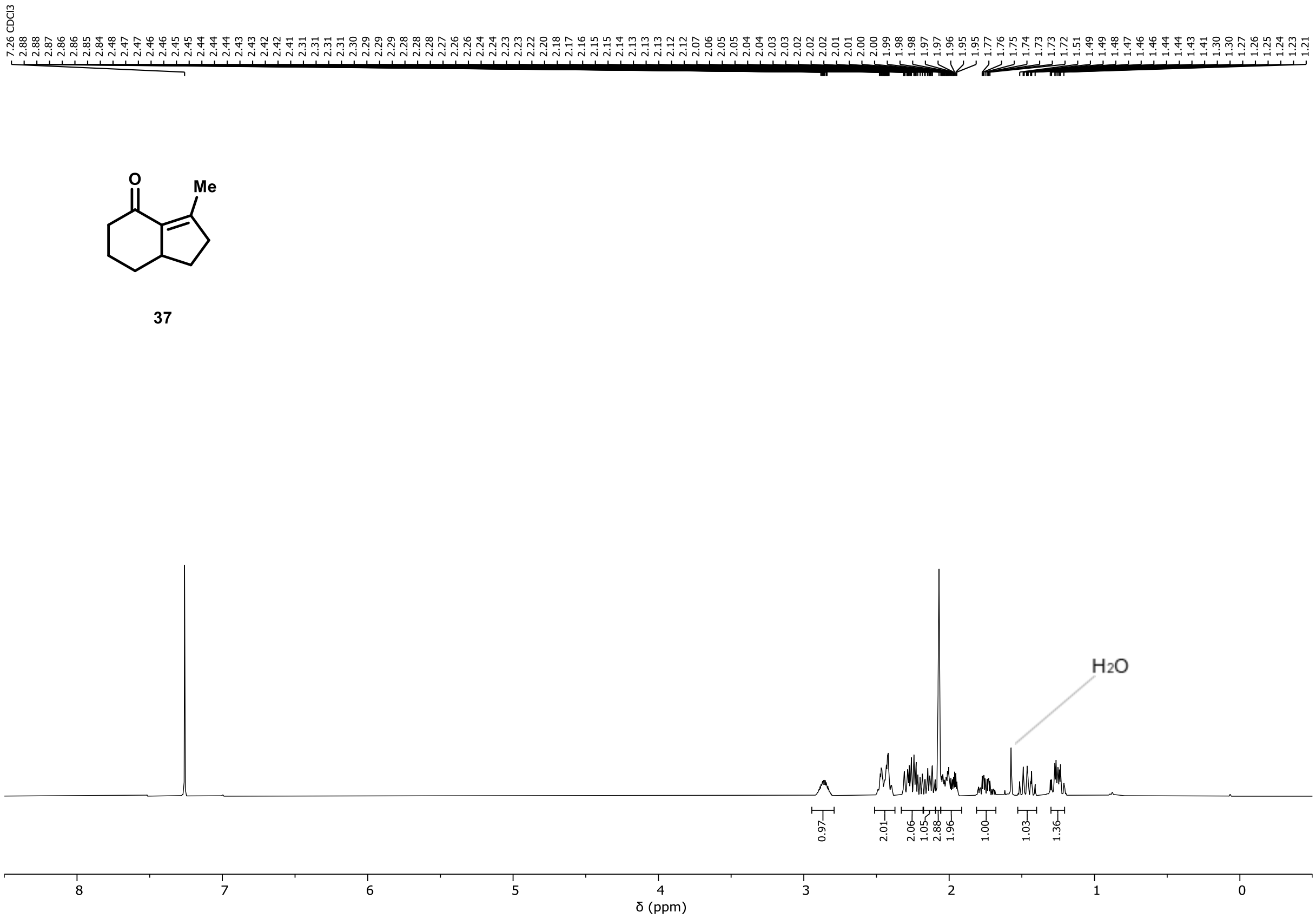
Me
O
37
H2O
0.97
2.01
2.06
1.05
2.88
1.96
1.00
1.03
1.36
δ (ppm)

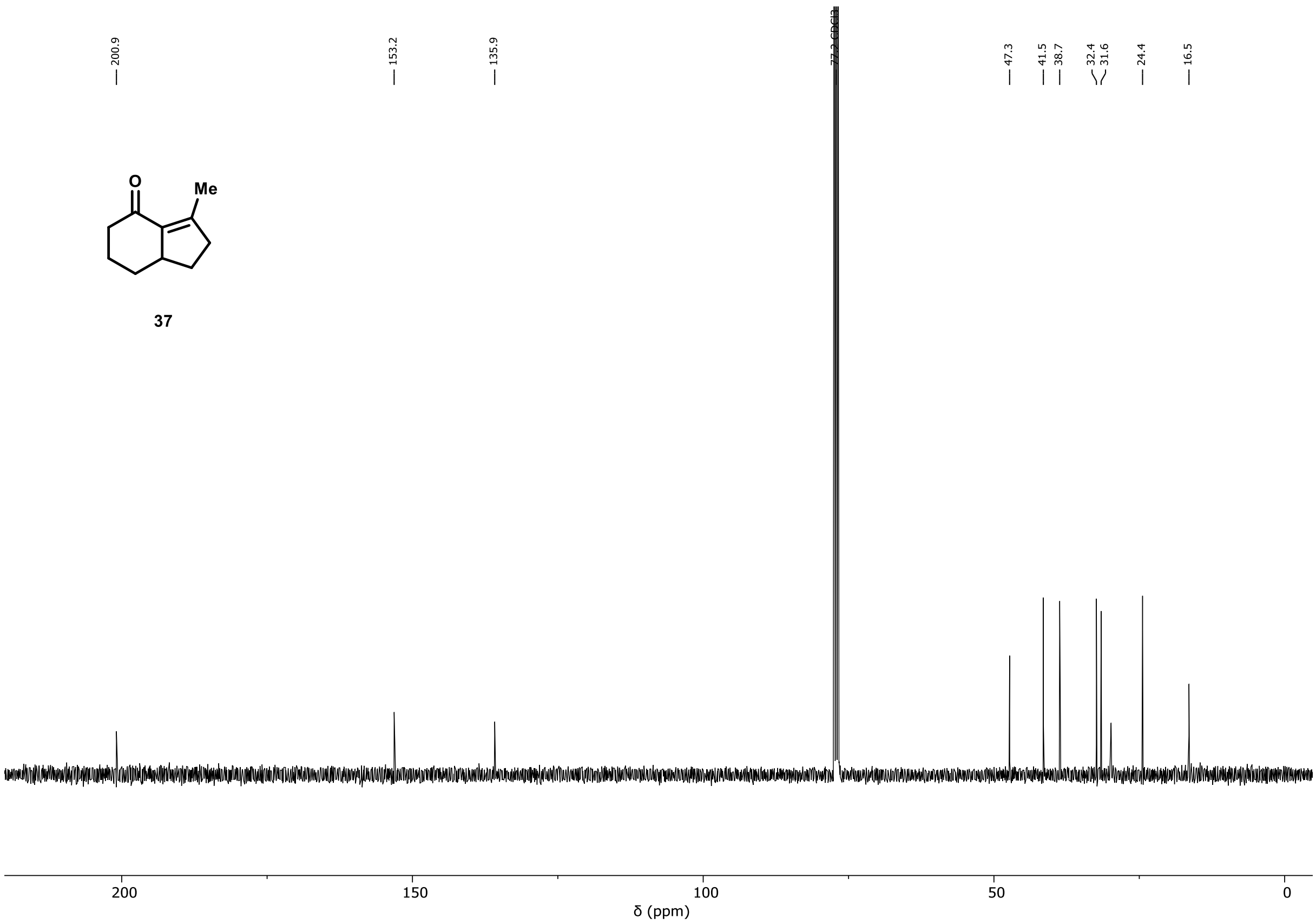
200.9
153.2
135.9
47.3
41.5
38.7
32.4
31.6
24.4
16.5
Me
O
37
δ (ppm)

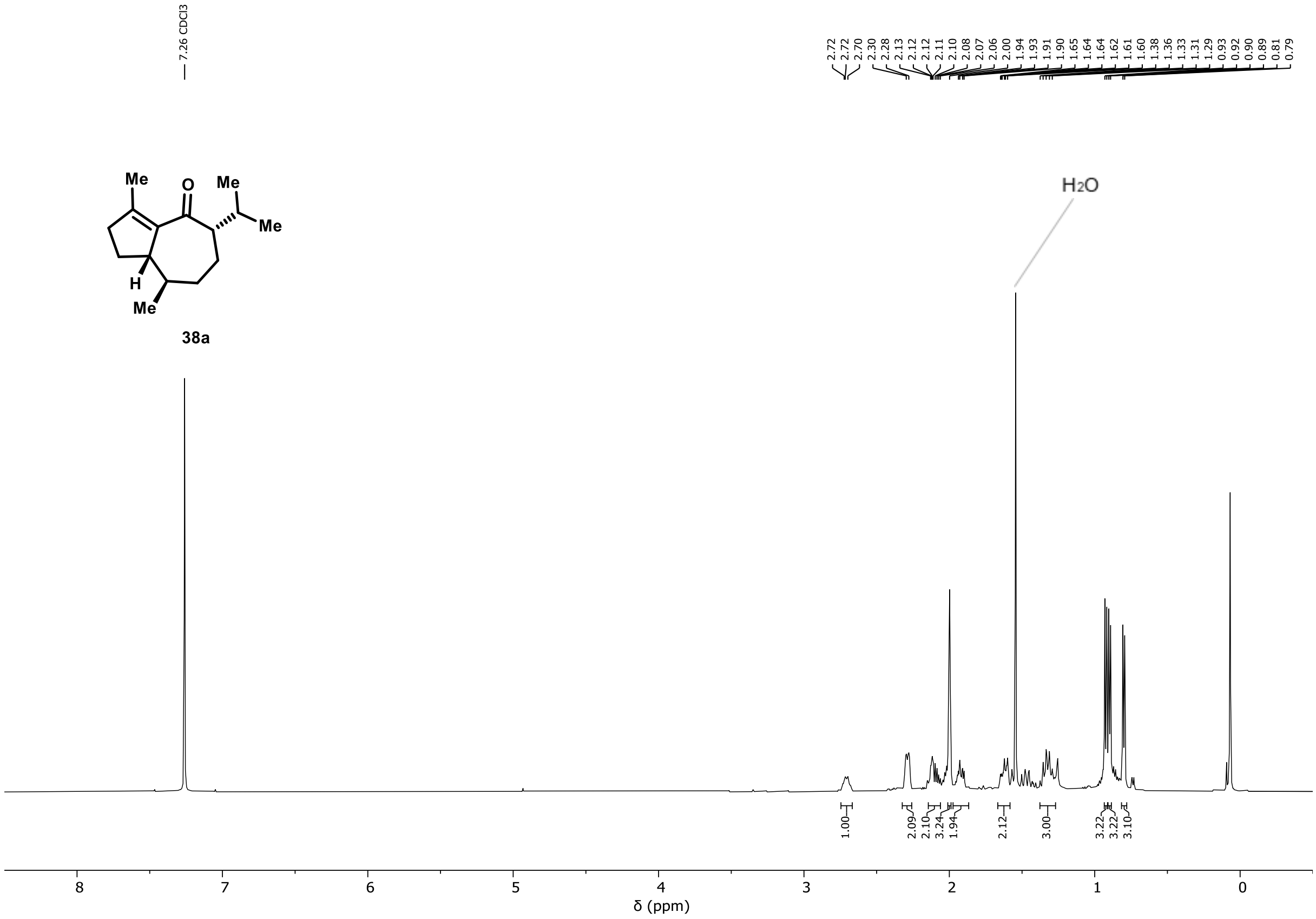
38a
H2O
δ (ppm)

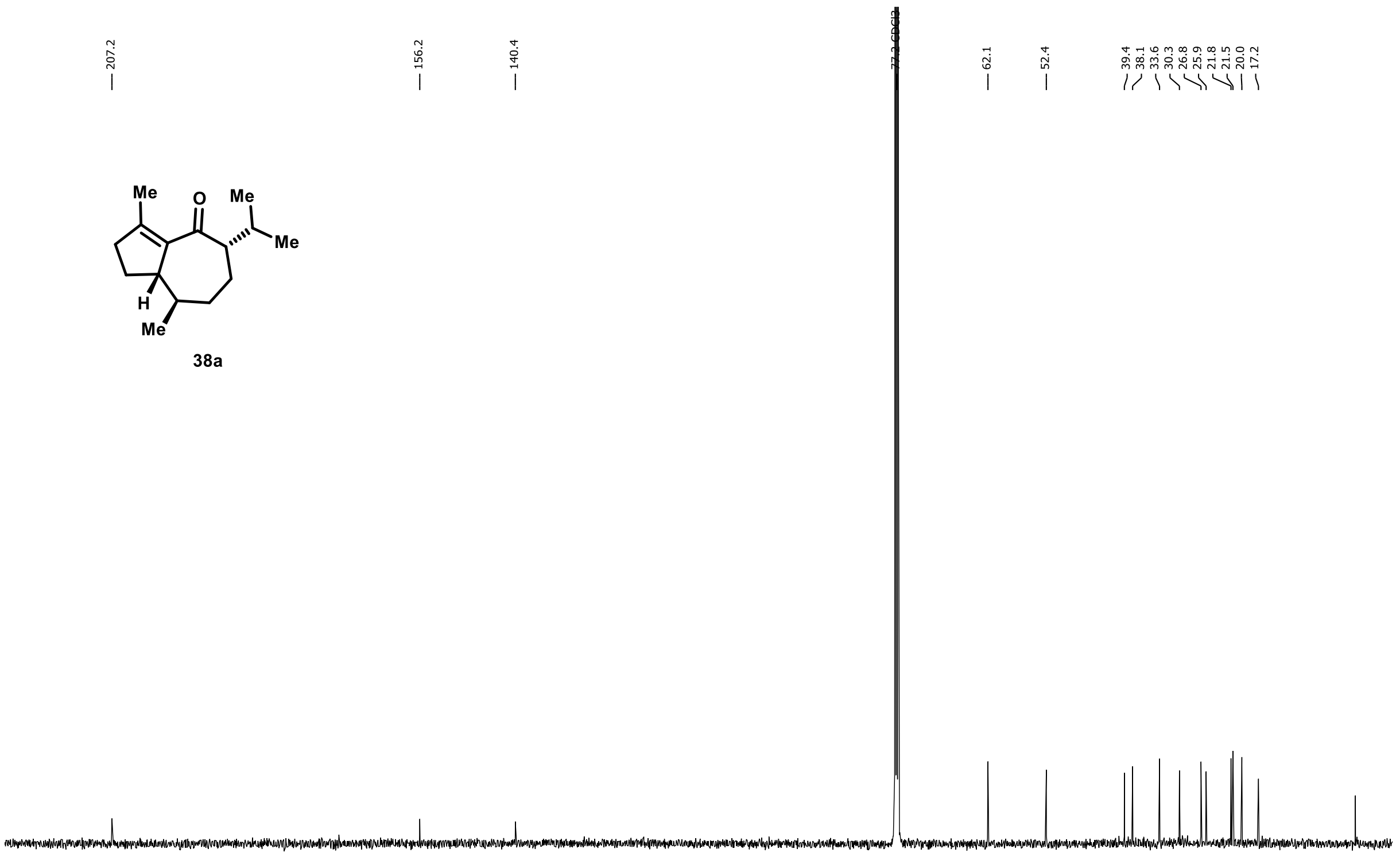
38a
δ (ppm)

38b

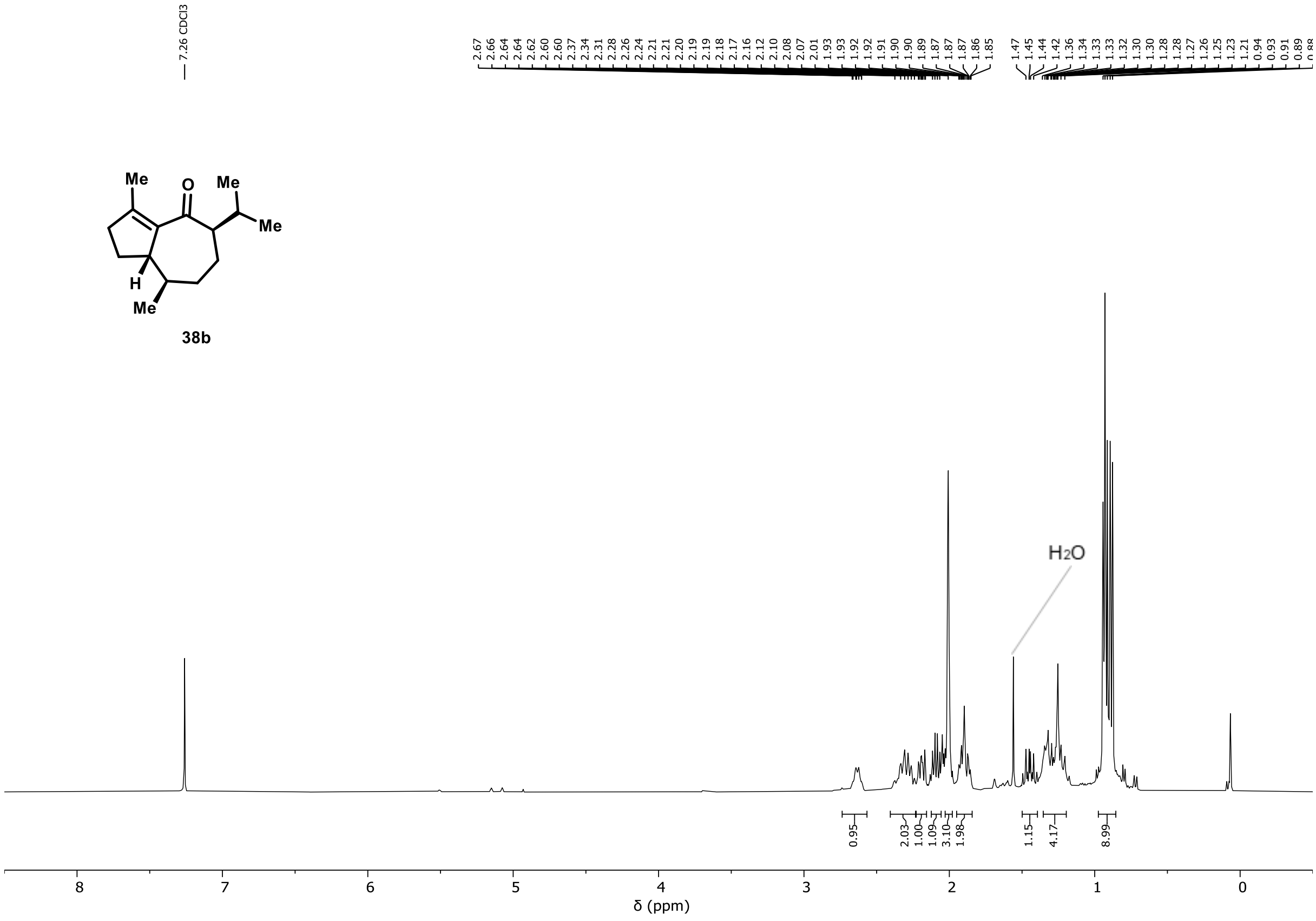
7.26 CDCl3
2.67 2.66 2.64 2.64 2.62 2.60 2.60 2.37 2.34 2.31 2.28 2.26 2.24 2.21 2.21 2.20 2.19 2.19 2.18 2.17 2.16 2.12 2.10 2.08 2.07 2.01 1.93 1.93 1.92 1.92 1.91 1.90 1.90 1.89 1.87 1.87 1.87 1.86 1.85
1.47 1.45 1.44 1.42 1.36 1.34 1.33 1.33 1.32 1.30 1.30 1.28 1.28 1.27 1.26 1.23 1.21 0.94 0.93 0.91 0.89
H2O
0.95
2.03 1.00 1.09 3.10 1.98
1.15 4.17
8.99
8 7 6 5 4 3 2 1 0
δ (ppm)

38b

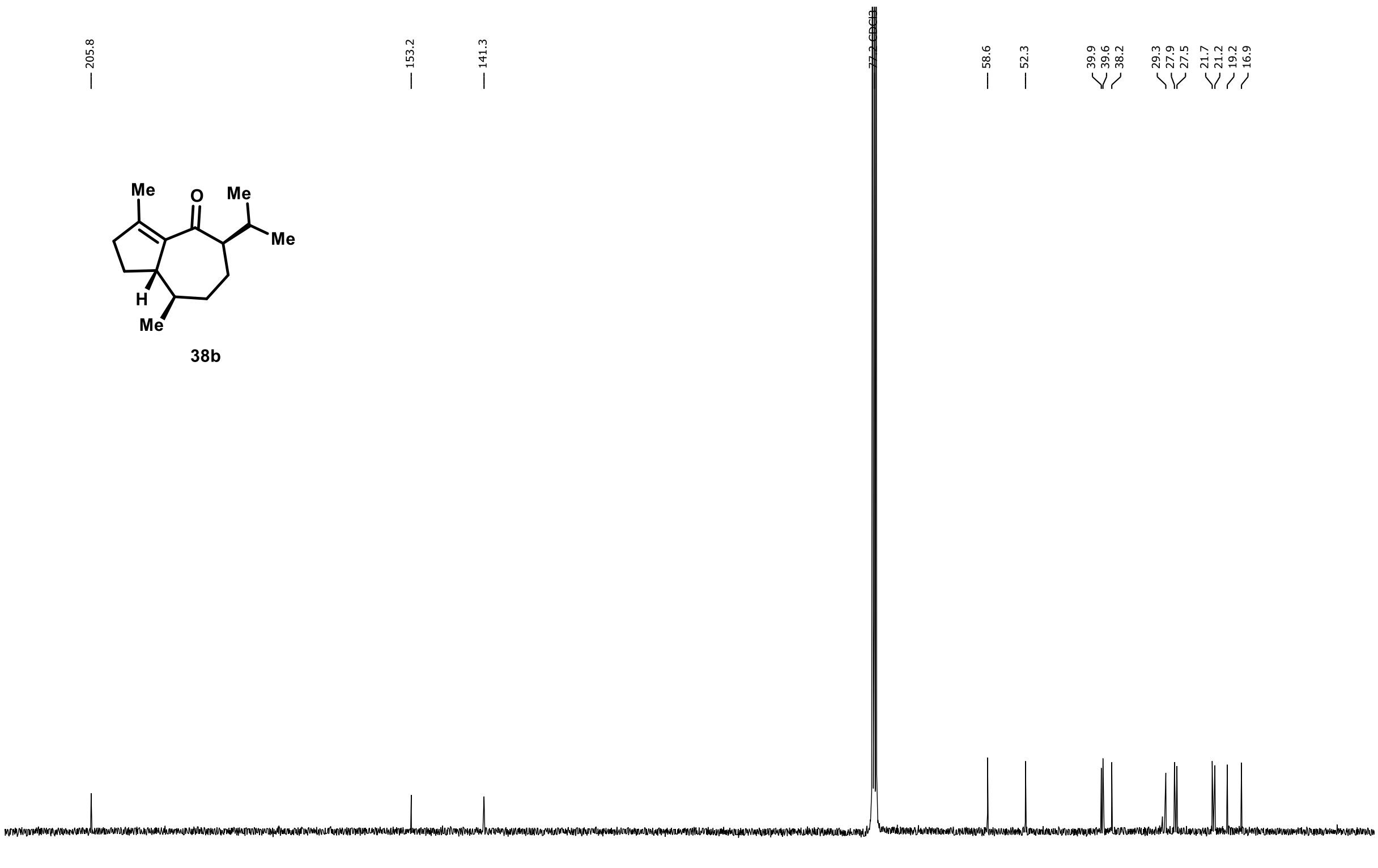
205.8
153.2
141.3
77.2 CDCl3
58.6
52.3
39.9 39.6 38.2
29.3 27.9 27.5
21.7 21.2 19.2 16.9
200 150 100 50 0
δ (ppm)

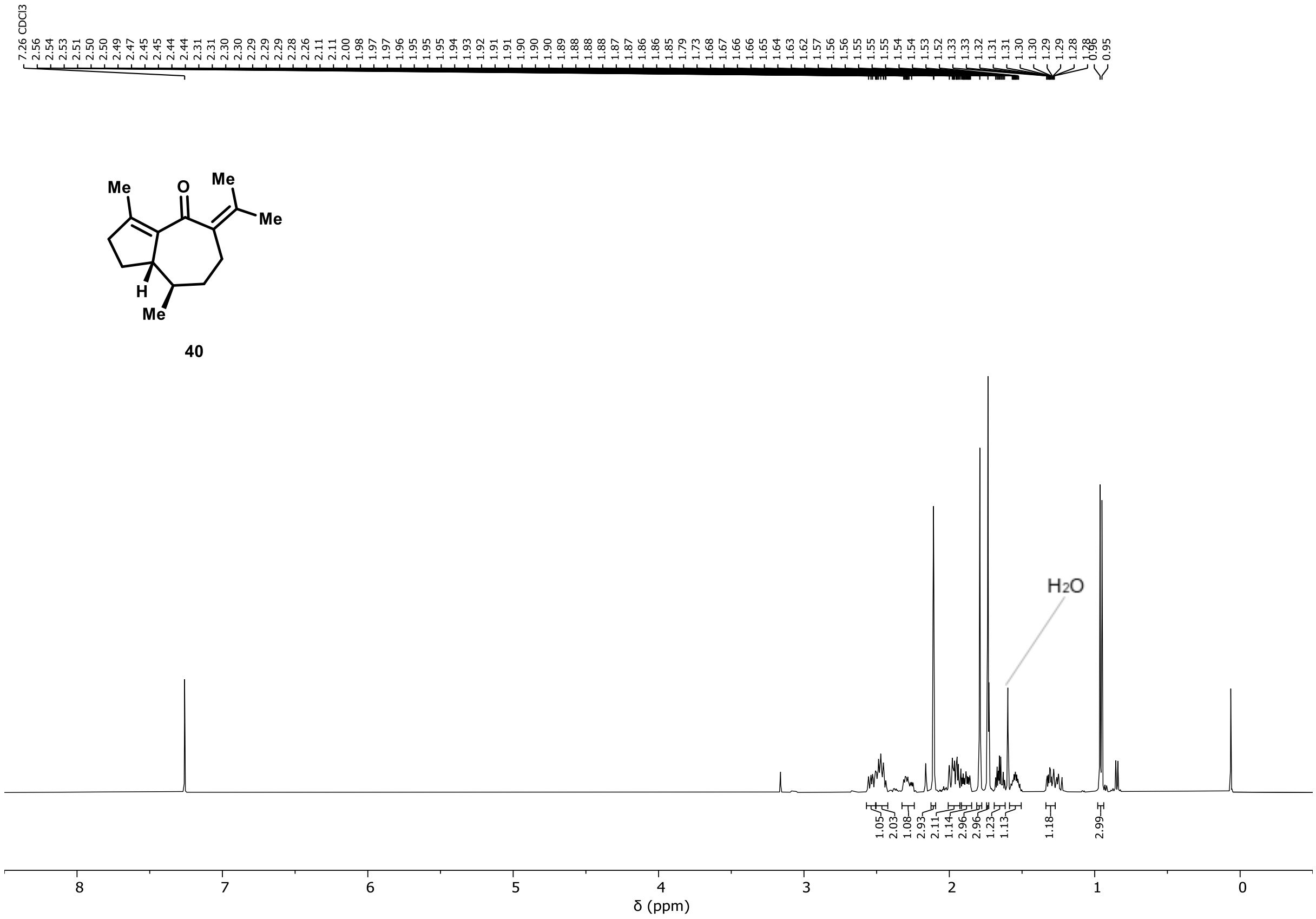
7.26 CDCl3
2.56
2.54
2.53
2.51
2.50
2.50
2.49
2.47
2.45
2.45
2.44
2.44
2.31
2.31
2.30
2.30
2.29
2.29
2.29
2.28
2.26
2.11
2.11
2.00
1.98
1.97
1.97
1.96
1.95
1.95
1.95
1.94
1.93
1.92
1.91
1.91
1.90
1.90
1.90
1.89
1.88
1.88
1.88
1.87
1.87
1.86
1.85
1.79
1.73
1.68
1.67
1.66
1.66
1.65
1.64
1.63
1.62
1.57
1.56
1.56
1.55
1.55
1.55
1.55
1.54
1.54
1.53
1.52
1.33
1.33
1.32
1.31
1.31
1.30
1.29
1.29
1.28
1.28
0.96
0.95
Me
O
Me
Me
H
Me
40
H2O
1.05
2.03
1.08
2.93
2.11
1.14
2.96
2.96
1.23
1.13
1.18
2.99
δ (ppm)

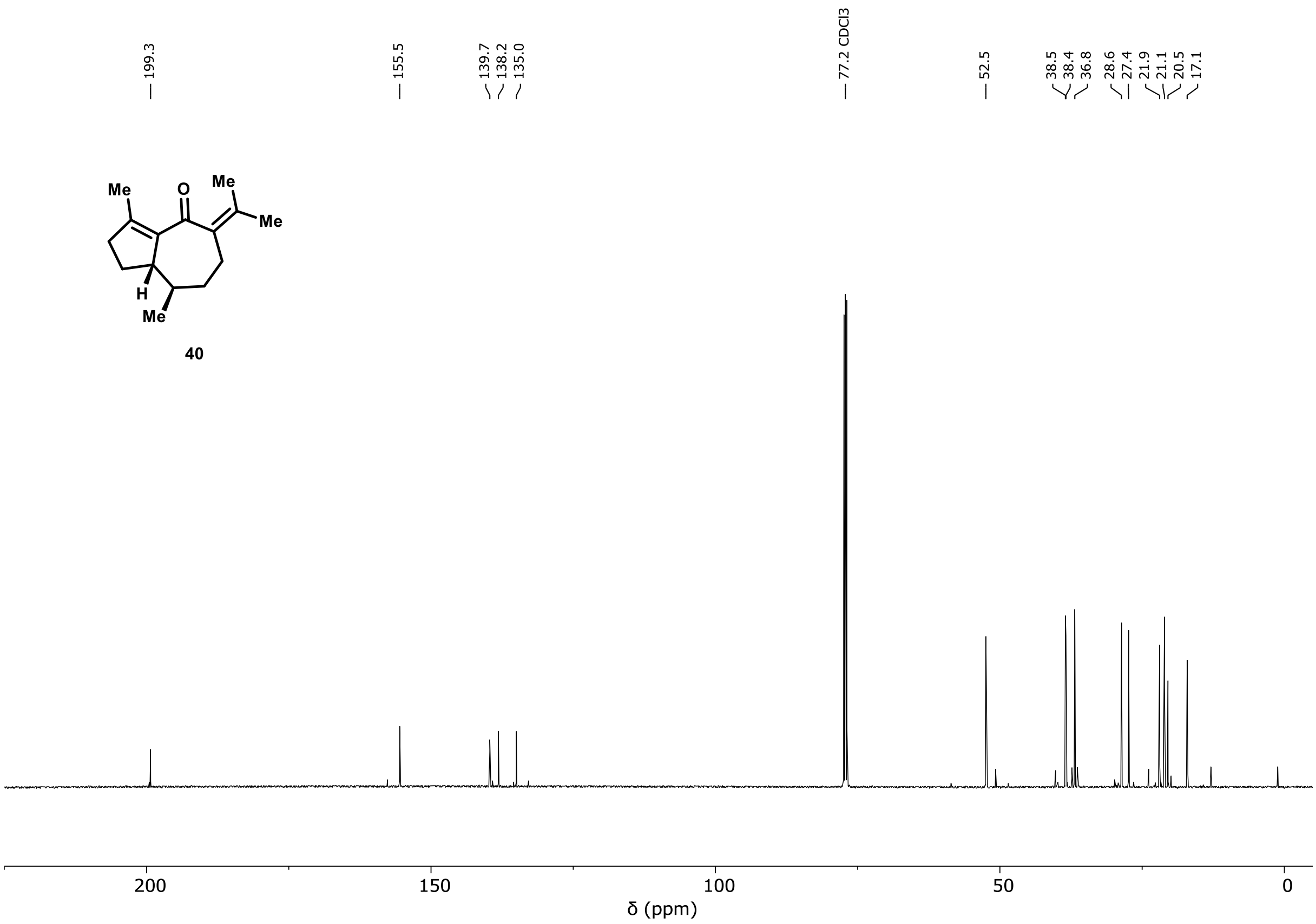
199.3
155.5
139.7
138.2
135.0
77.2 CDCl3
52.5
38.5
38.4
36.8
28.6
27.4
21.9
21.1
20.5
17.1
Me
O
Me
Me
H
Me
40
δ (ppm)

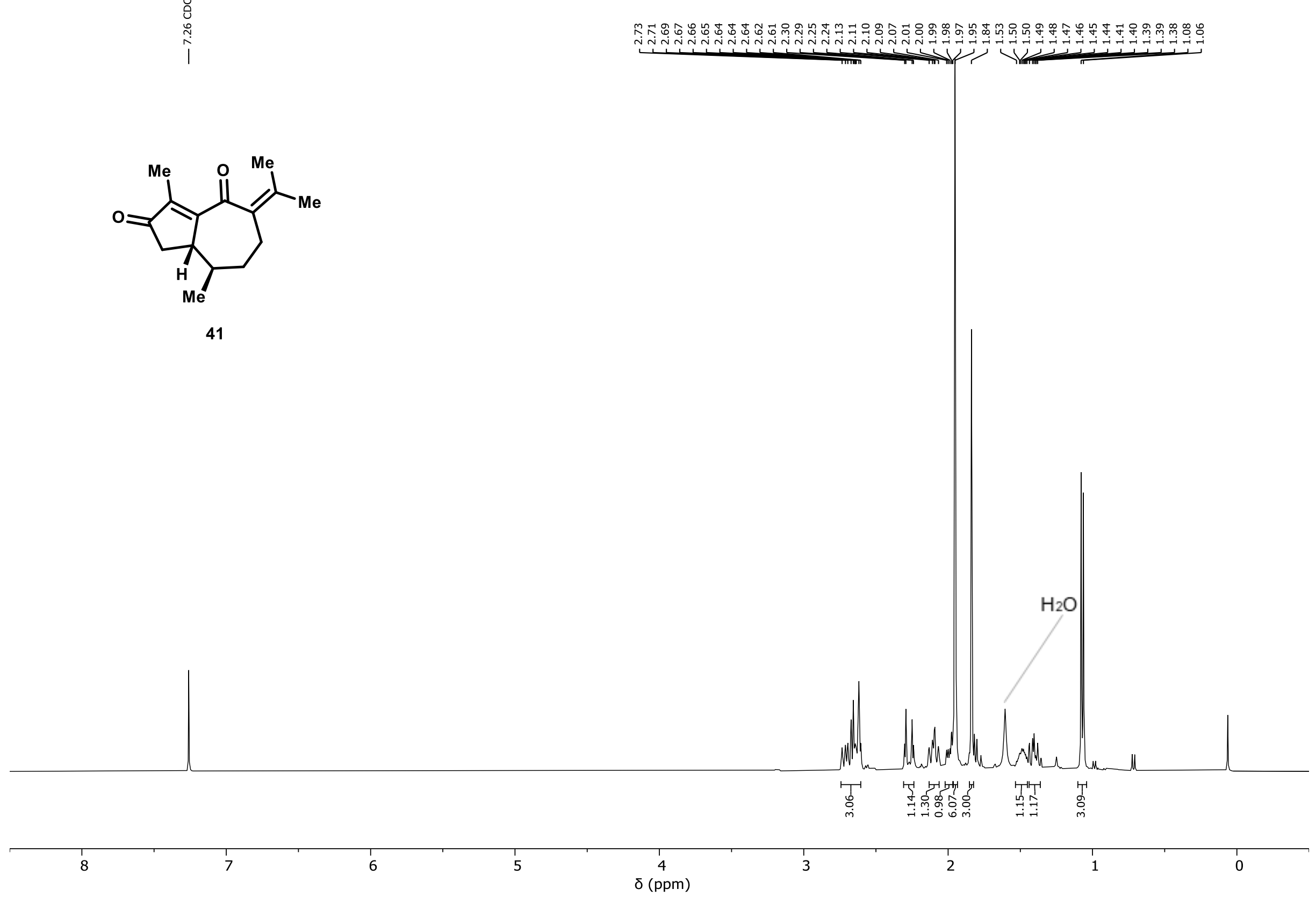
7.26 CDCl3
2.73 2.71 2.69 2.67 2.66 2.65 2.64 2.64 2.62 2.61 2.30 2.29 2.25 2.24 2.13 2.11 2.10 2.09 2.07 2.01 2.00 1.99 1.98 1.97 1.95 1.84 1.53 1.50 1.50 1.49 1.48 1.47 1.46 1.45 1.44 1.41 1.40 1.39 1.38 1.08 1.06
Me
O
Me
Me
O
H
Me
41
H2O
3.06
1.14
1.30
0.98
6.07
3.00
1.15
1.17
3.09
8 7 6 5 4 3 2 1 0
δ (ppm)

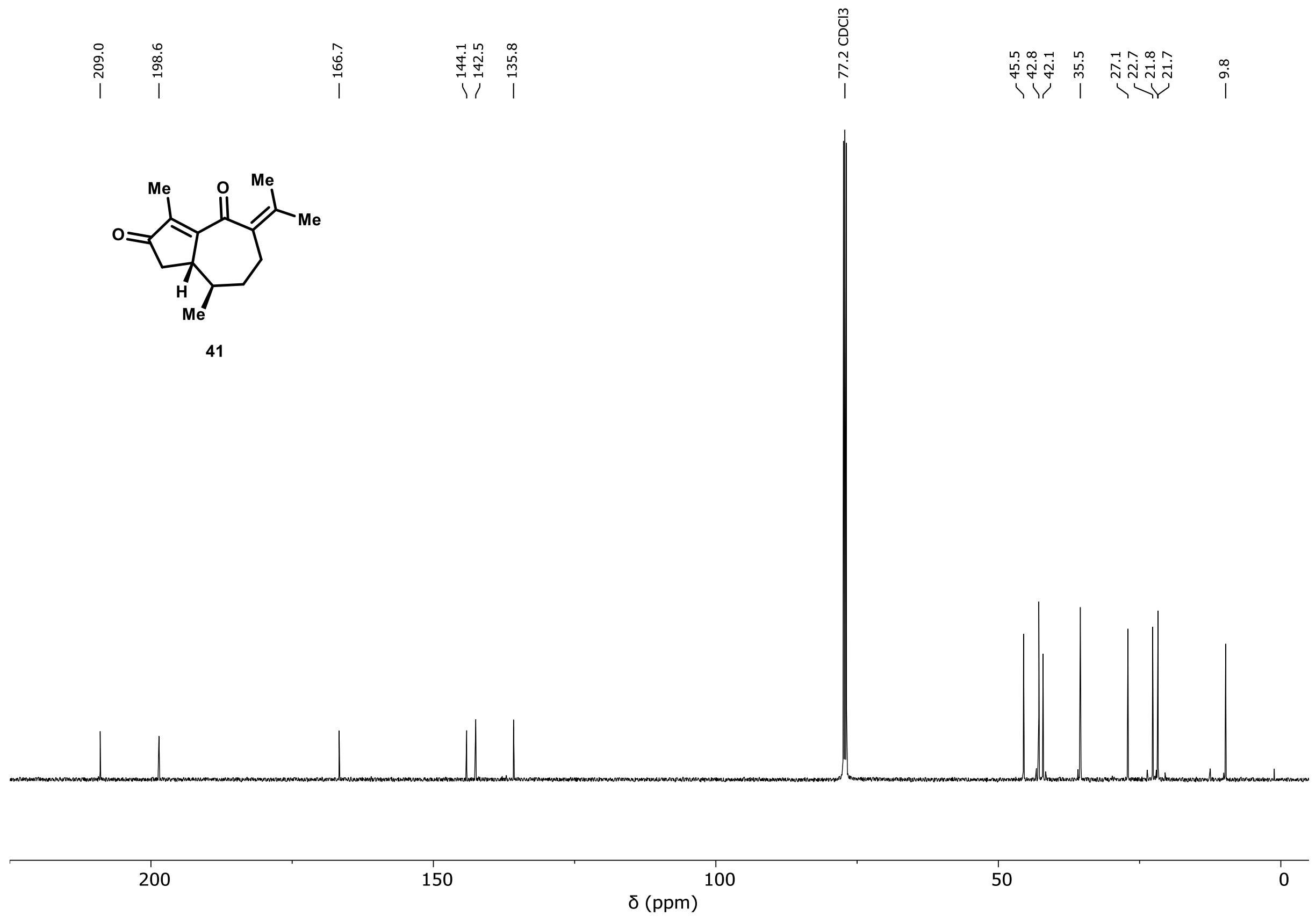
209.0
198.6
166.7
144.1
142.5
135.8
77.2 CDCl3
45.5
42.8
42.1
35.5
27.1
22.7
21.8
21.7
9.8
Me
O
Me
Me
O
H
Me
41
200 150 100 50 0
δ (ppm)

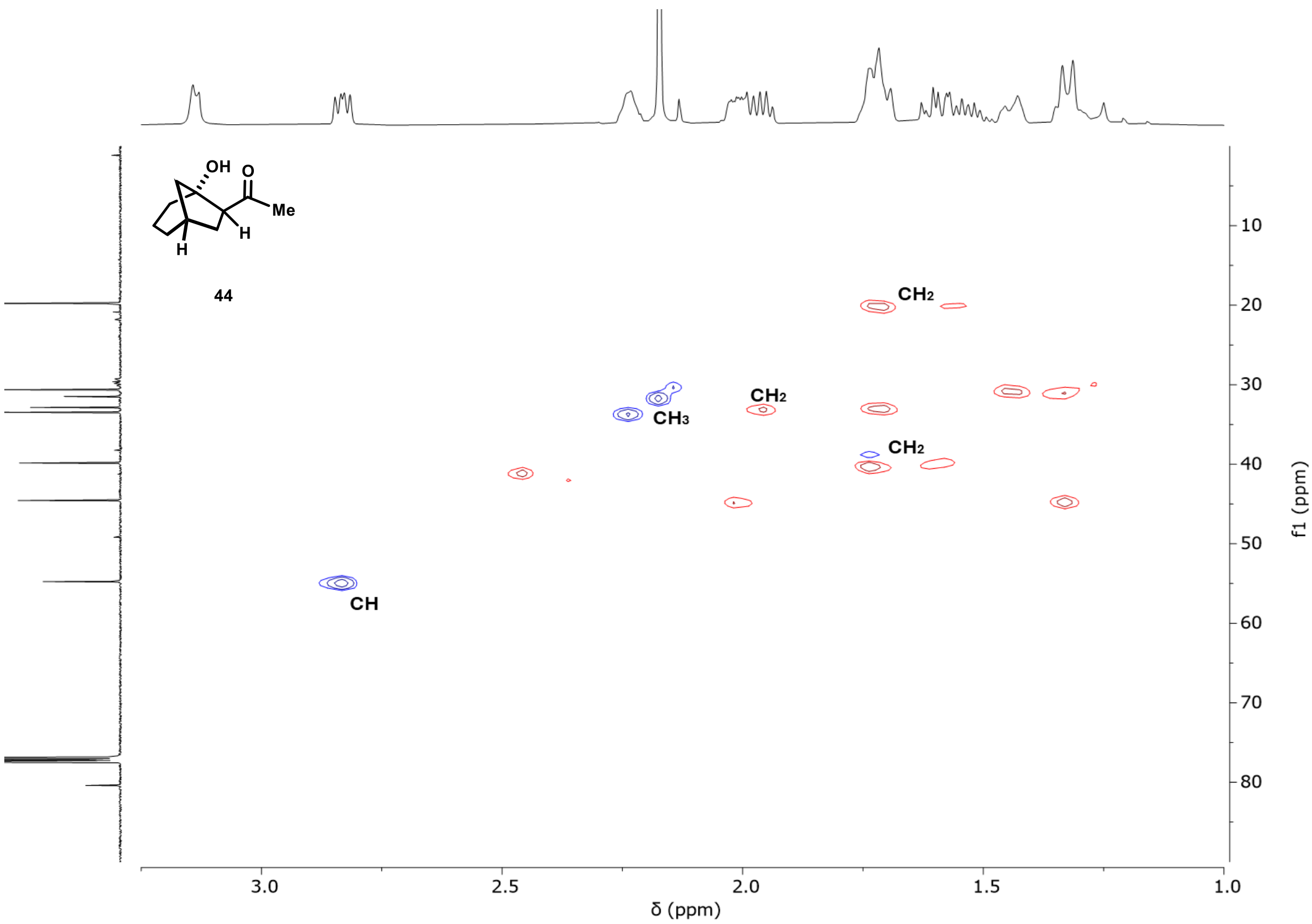

Figure S14. Edited HSQC NMR (500 MHz, $CDCl_3$) of compound **44.**

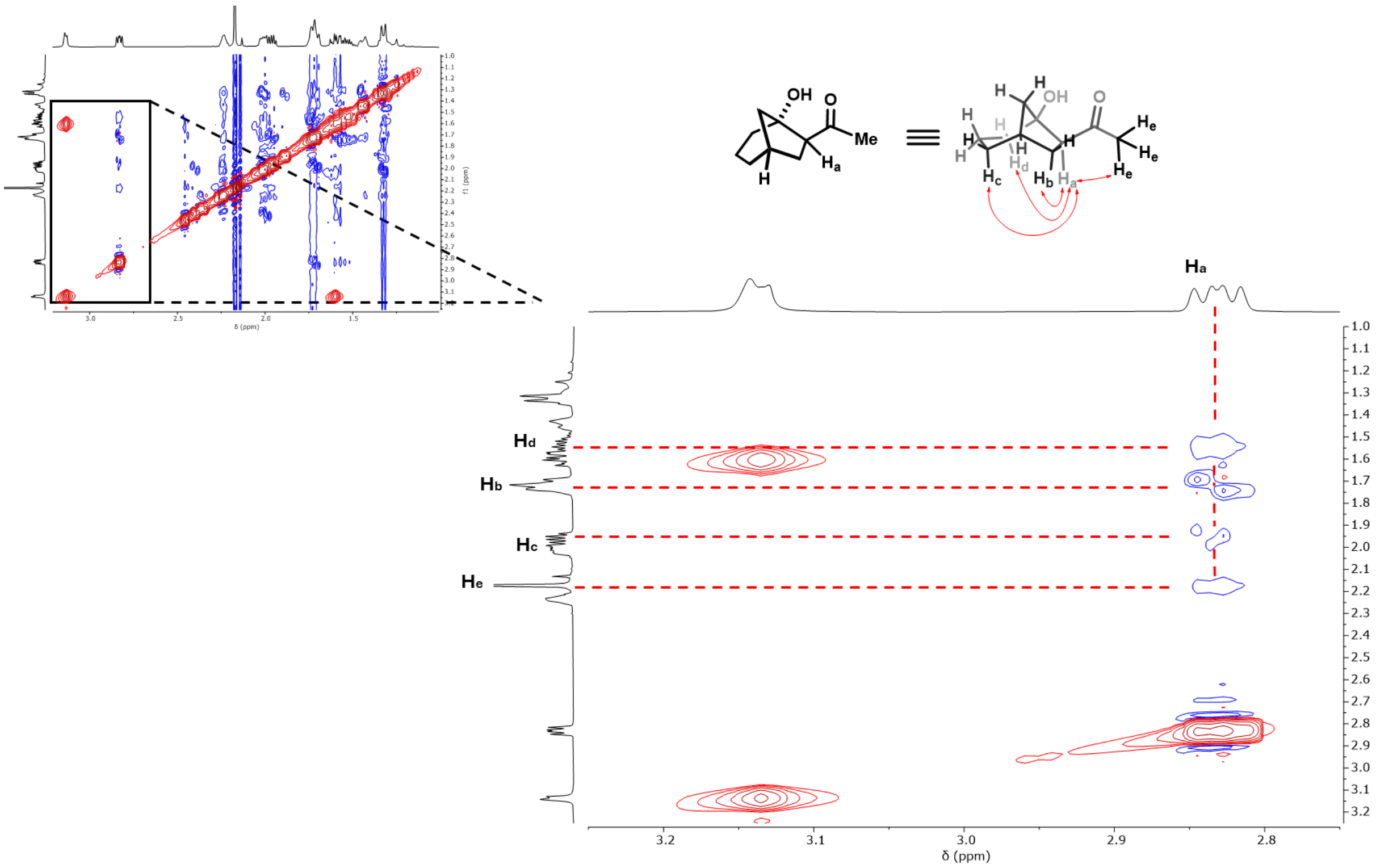

Figure S15. NOESY NMR (^{1}H 500 MHz, $CDCl_3$) of compound **44**, showing NOE correlations between the CH proton of interest (H_a) and the methyl group (H_e), as well as three methylene protons ($H_{b\text{-}d}$) on the seven-membered ring, which are expected only for this relative configuration.

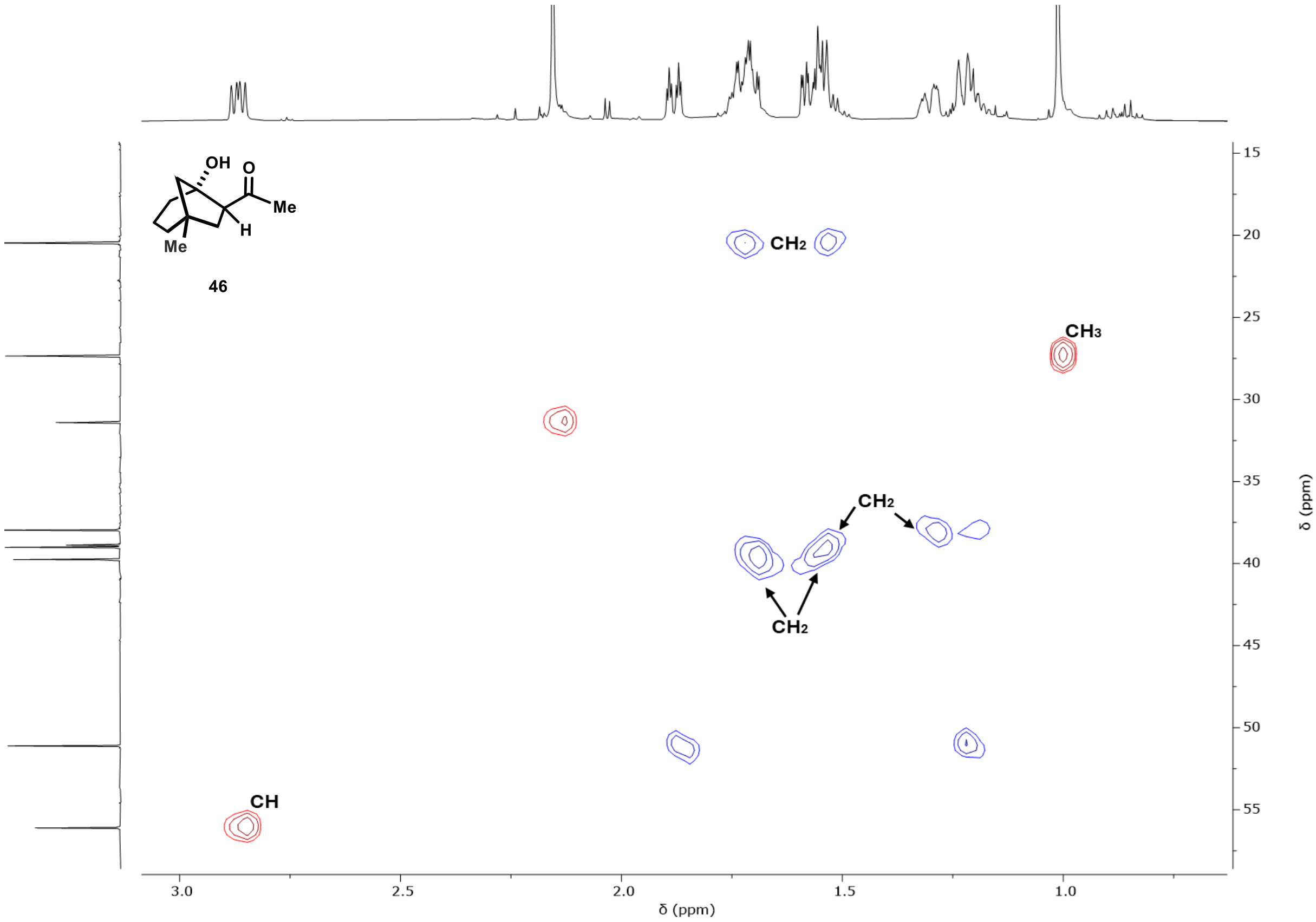

Figure S16. Edited HSQC NMR (500 MHz, $CDCl_3$) of compound **46.**

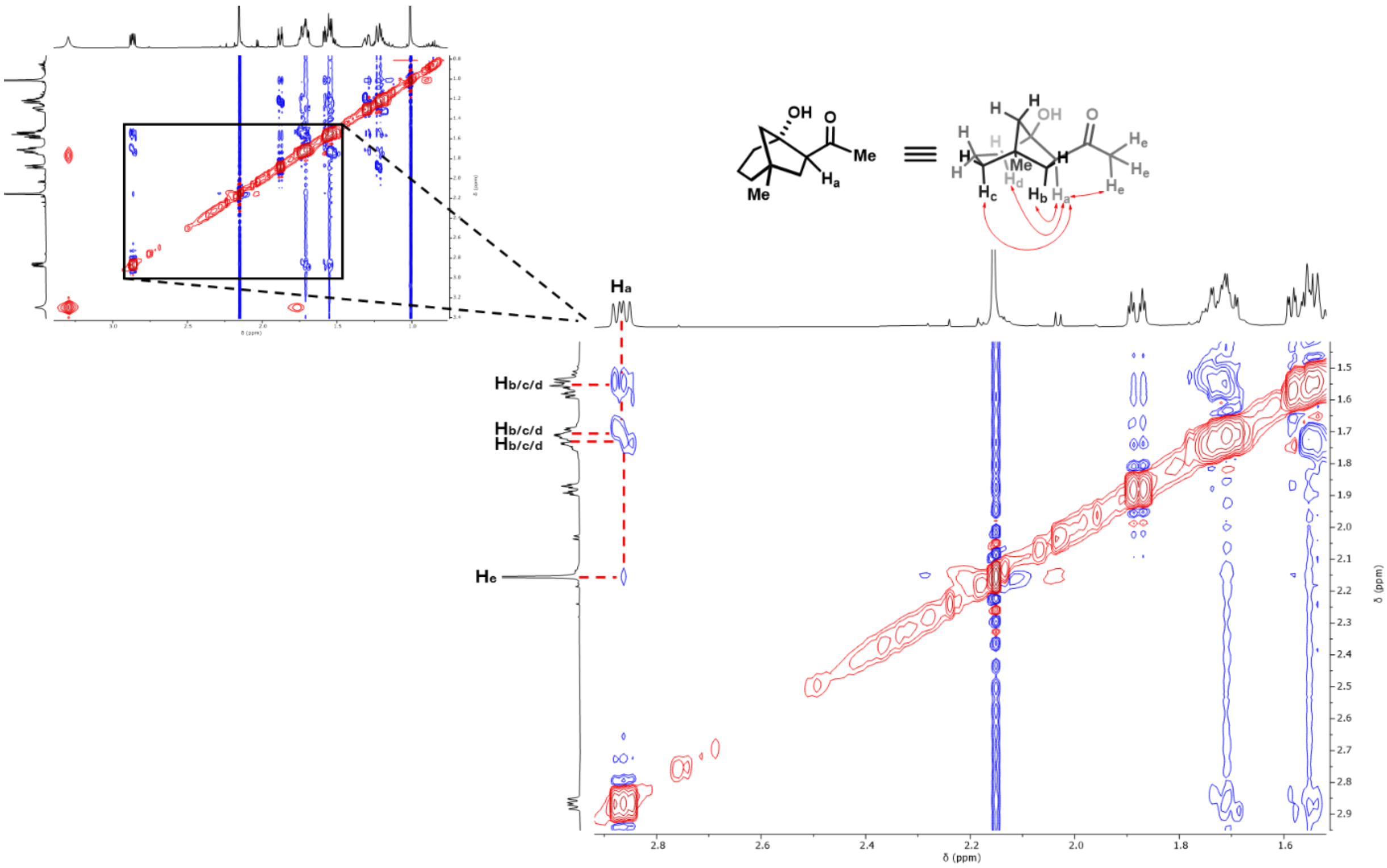

Figure S17. NOESY NMR (^{1}H 500 MHz, $CDCl_3$) of compound **46**, showing NOE correlations between the CH proton of interest (H_a) and the methyl group (H_e), as well as three methylene protons ($H_{b\text{-}d}$) on the seven-membered ring, which are expected only for this relative configuration.

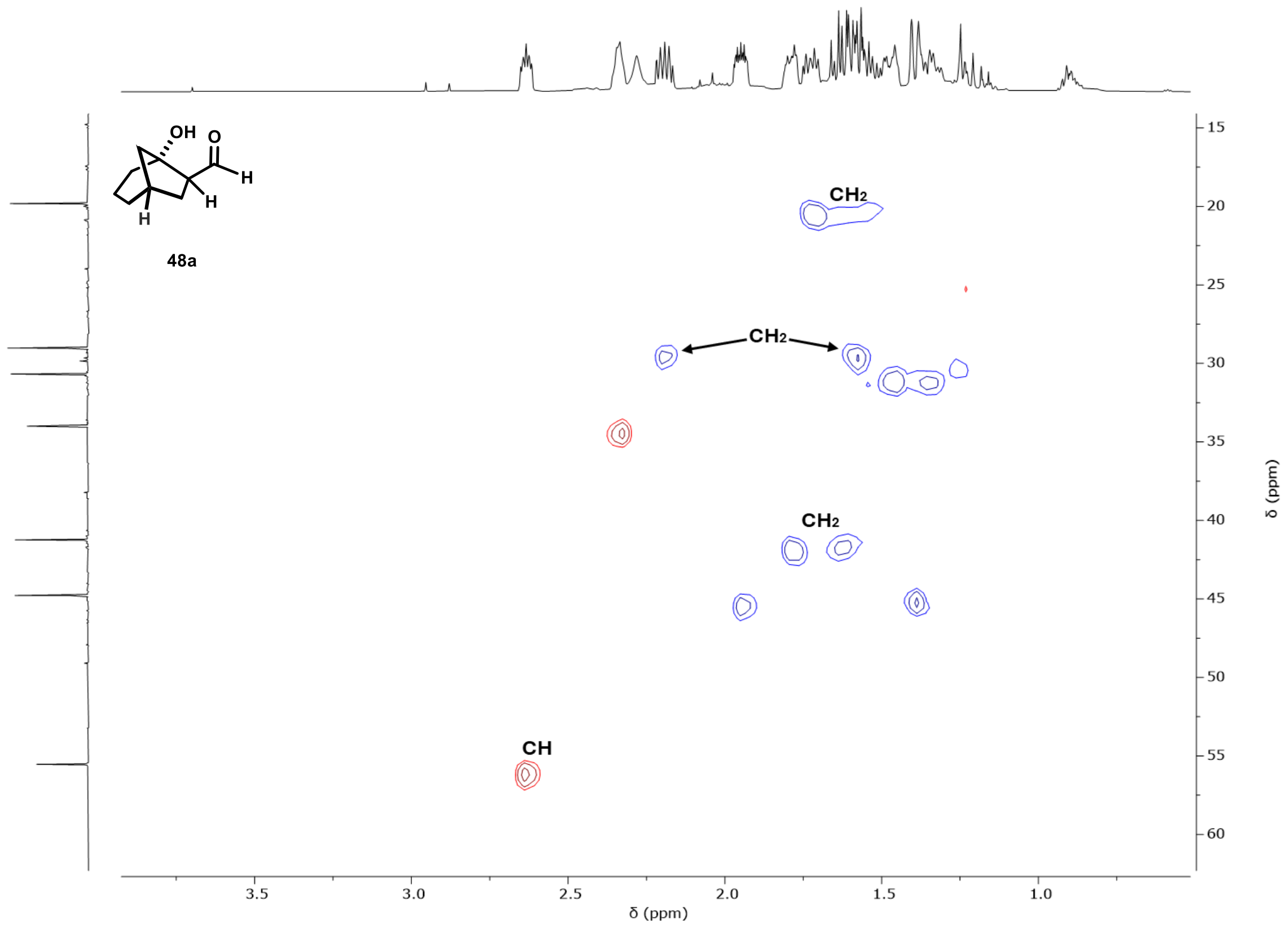

Figure S18. Edited HSQC NMR (500 MHz, $CDCl_3$) of compound **48a.**

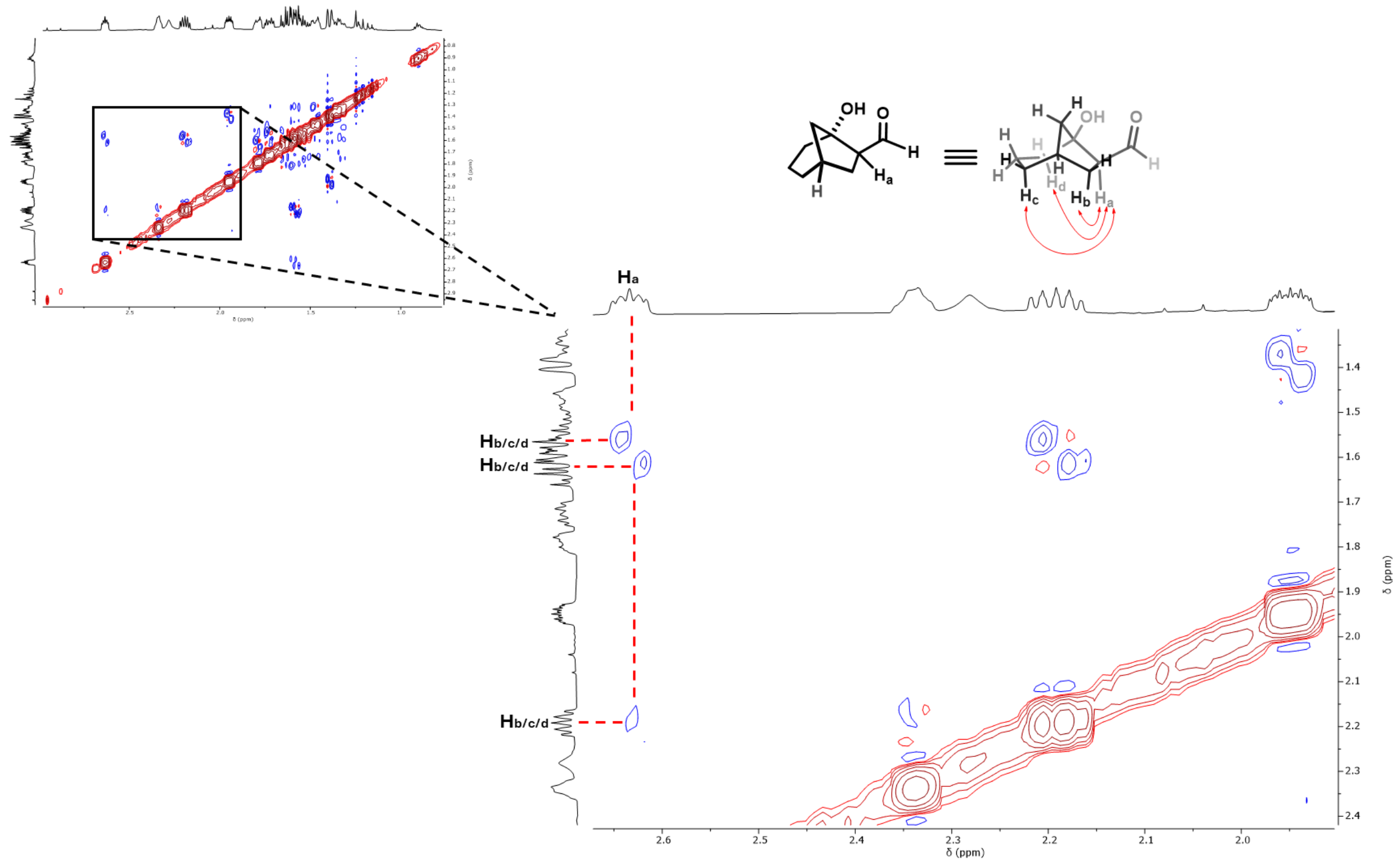

Figure S19. NOESY NMR (^1H 500 MHz, $CDCl_3$) of compound **48a**, showing NOE correlations between the CH proton of interest (H_a) and three methylene protons ($H_{b\text{-}d}$) on the seven-membered ring, which are expected only for this relative configuration.

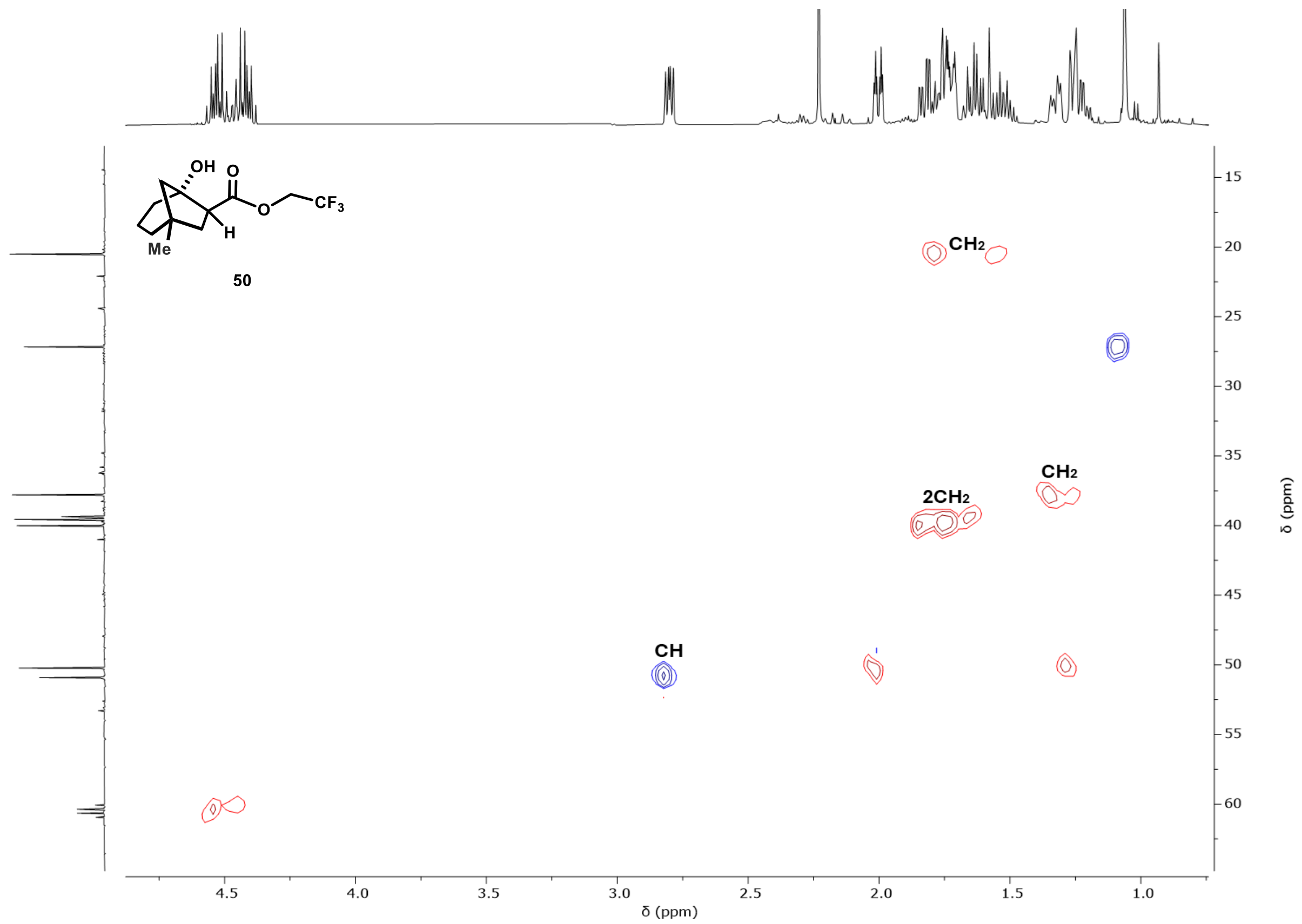

Figure S20. Edited HSQC NMR (500 MHz, $CDCl_3$) of compound **50.**

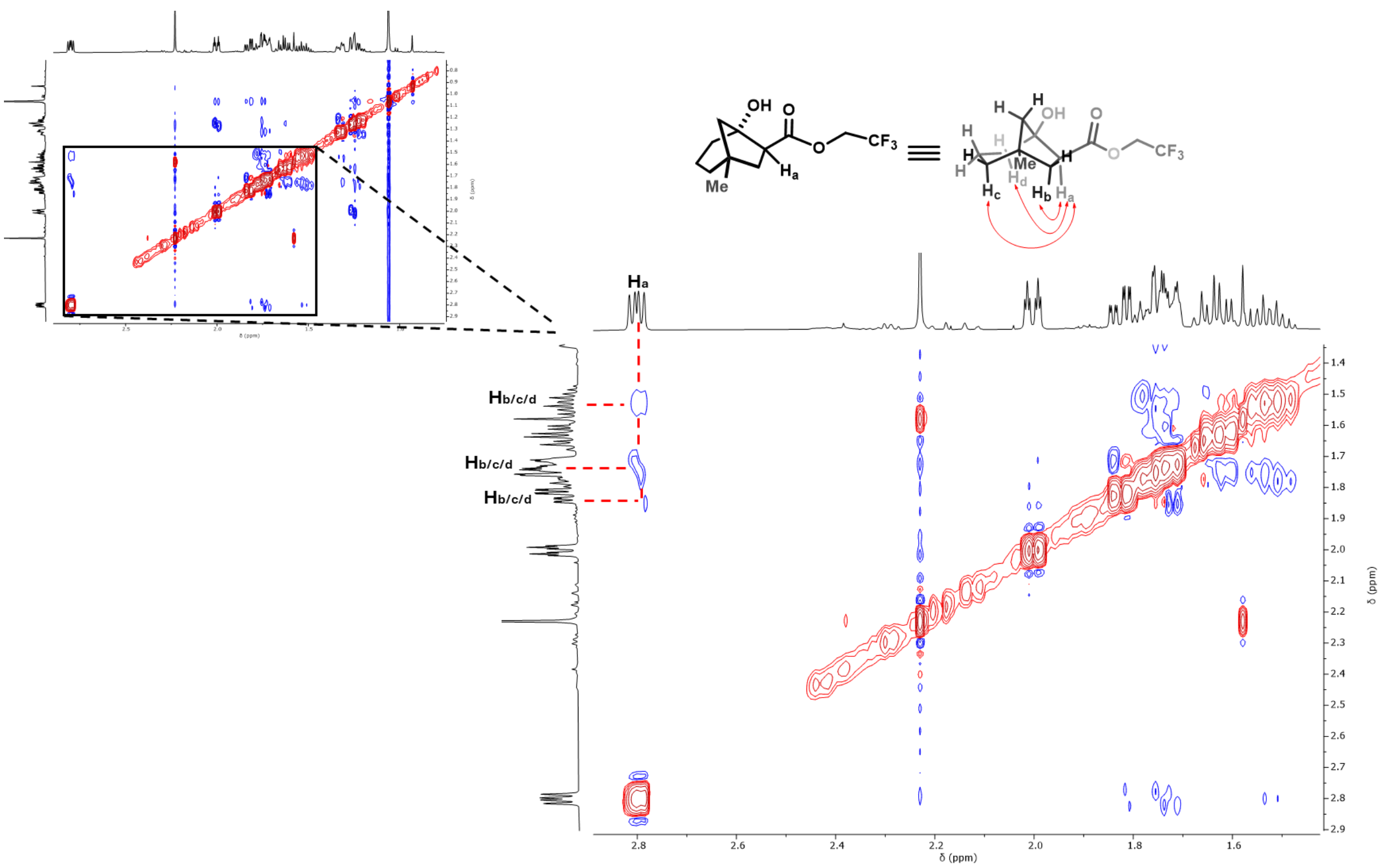

Figure S21. NOESY NMR (^{1}H 500 MHz, $CDCl_3$) of compound **50**, showing NOE correlations between the CH proton of interest (H_a) and three methylene protons (H_{b-d}) on the seven-membered ring, which are expected only for this relative configuration.

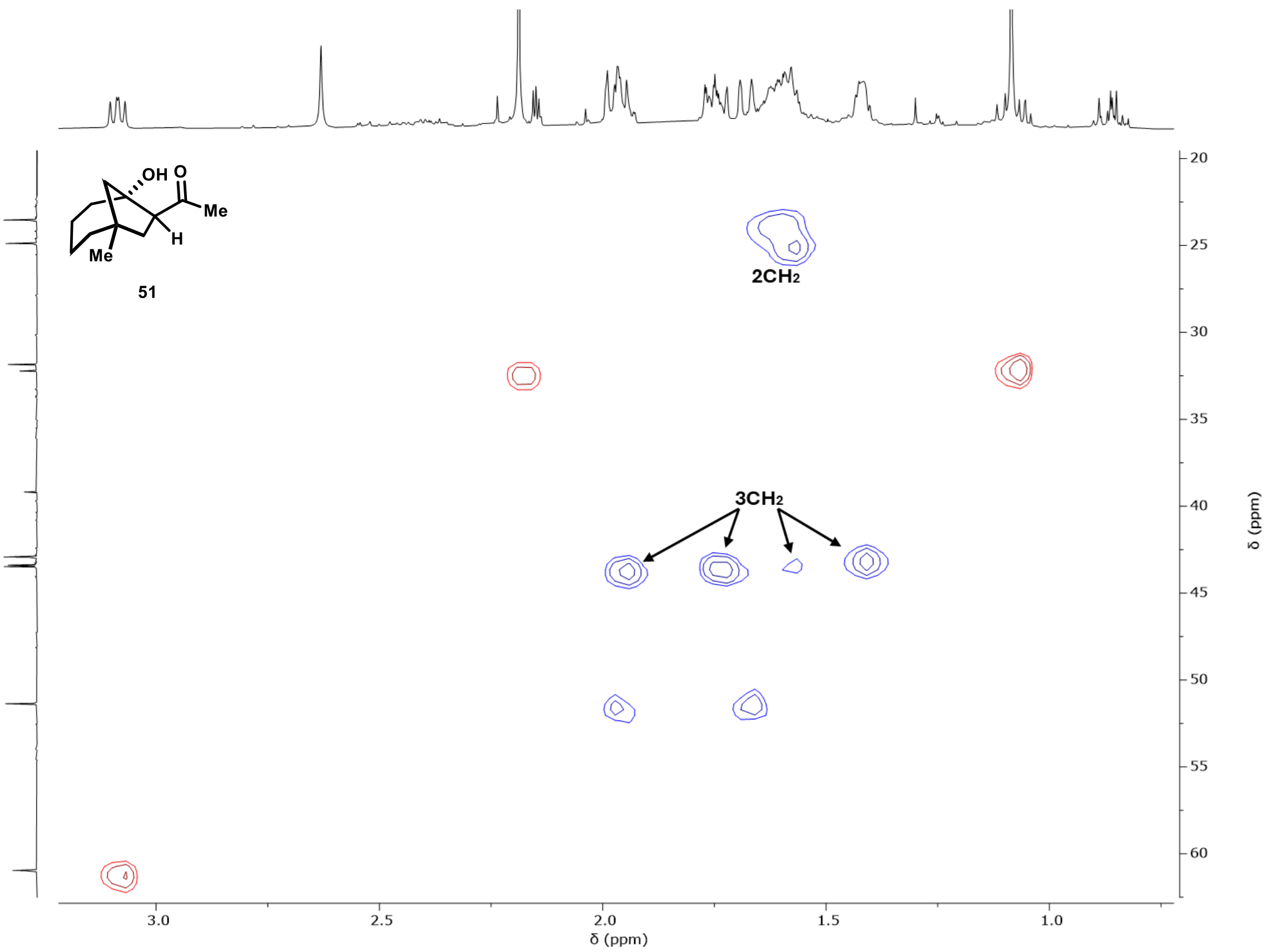

Figure S22. Edited HSQC NMR (500 MHz, $CDCl_3$) of compound **51.**

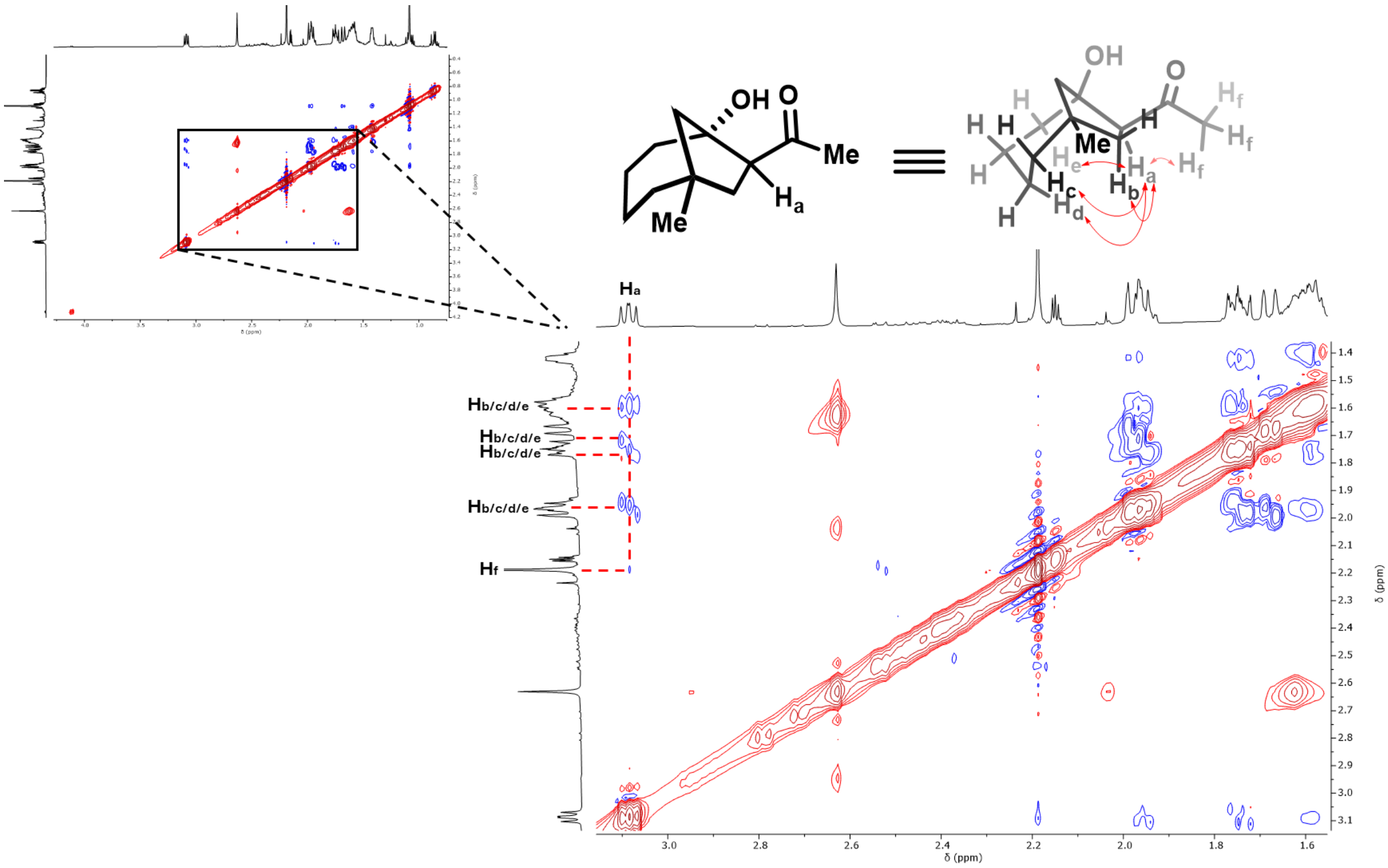

Figure S23. NOESY NMR (^1H 500 MHz, $CDCl_3$) of compound **51**, showing NOE correlations between the CH proton of interest (H_a) and the methyl group (H_f), as well as four methylene protons (H_{b-e}) on the eight-membered ring, which are expected only for this relative configuration.

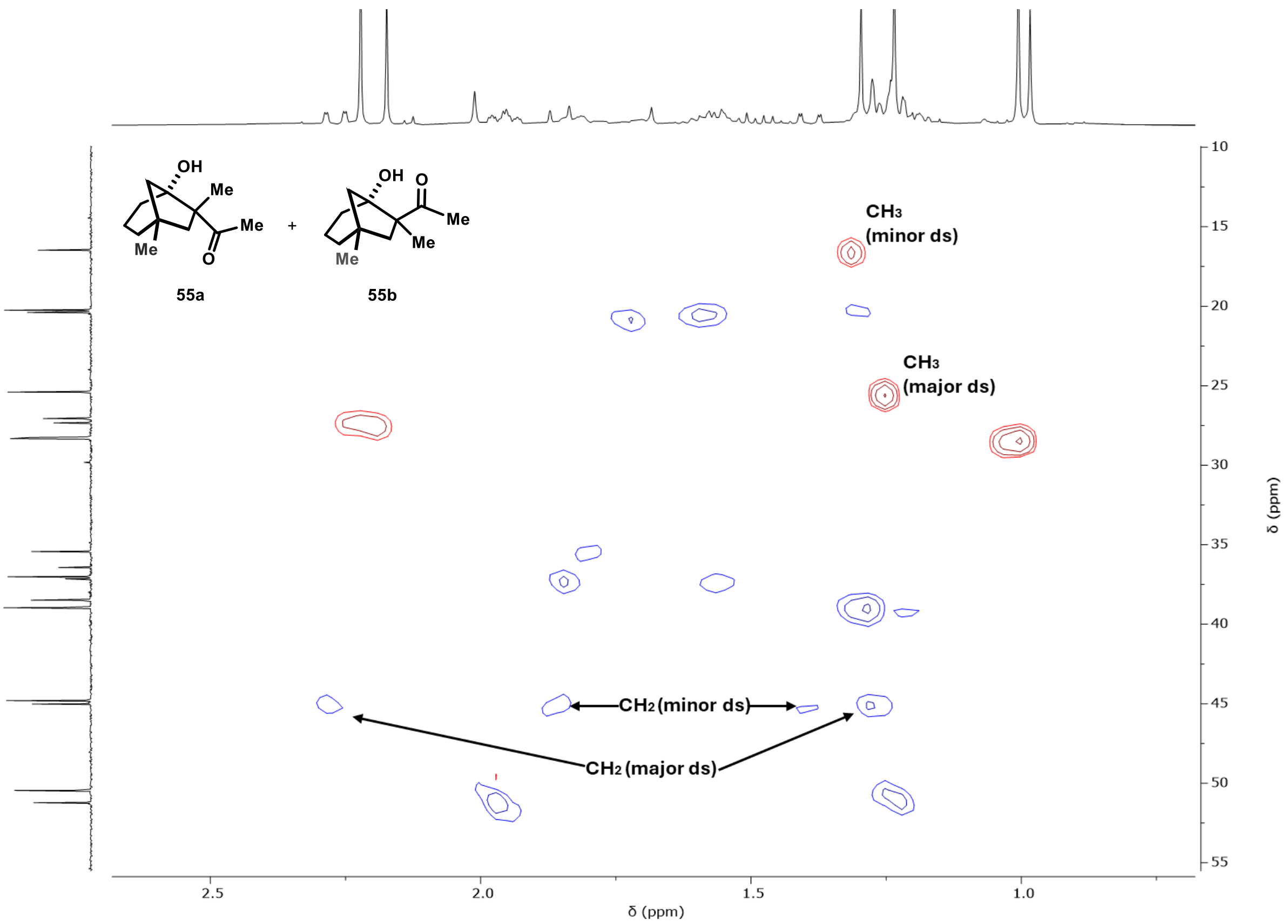

Figure S24. Edited HSQC NMR (500 MHz, $CDCl_3$) of a mixture of compounds **55a** and **55b.**

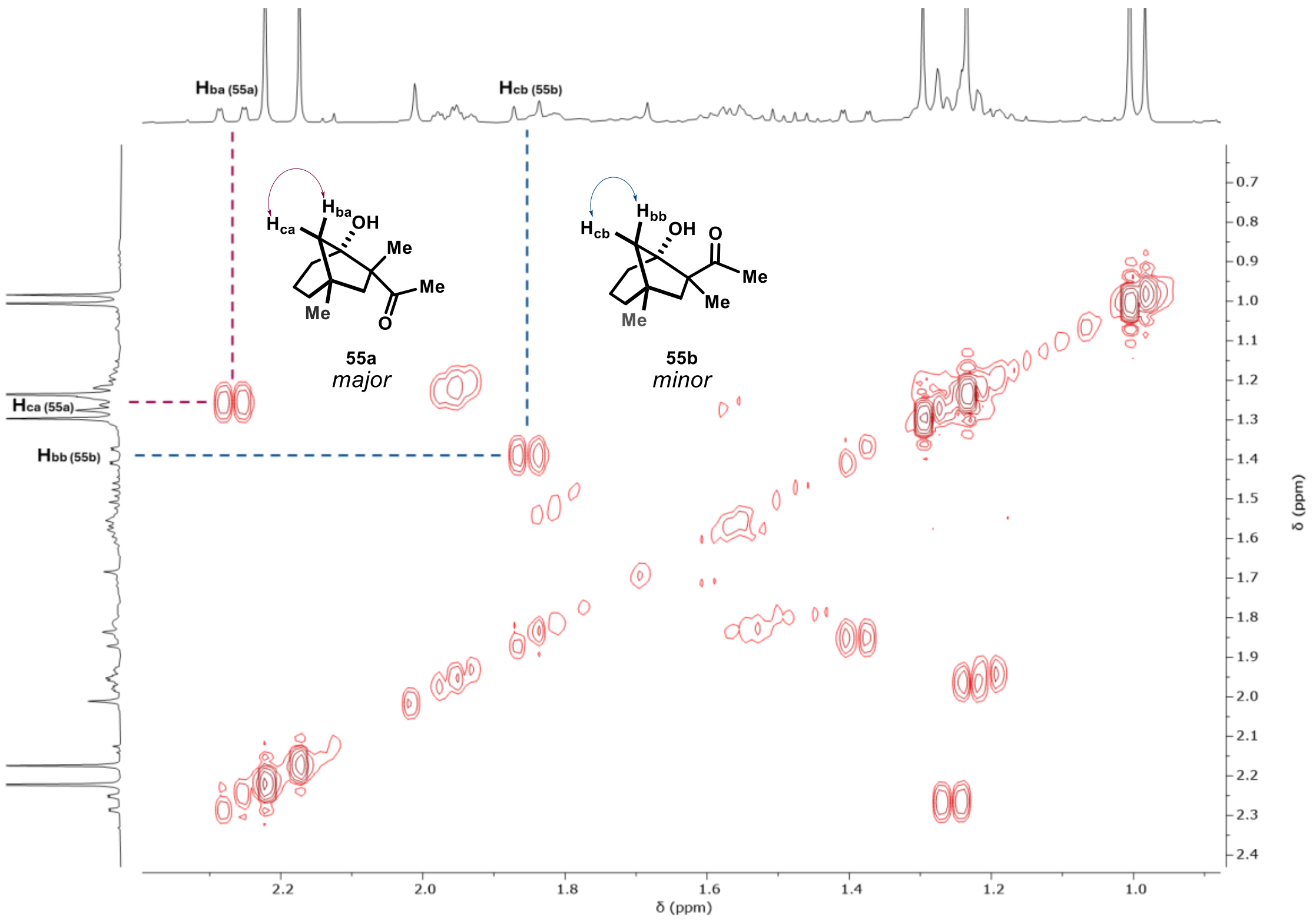

Figure S25. COSY NMR (^1H 500 MHz, $CDCl_3$) of a mixture of compounds **55a** and **55b**, showing COSY correlations between the bridgehead protons, H_b and H_c, in both diasteromers.

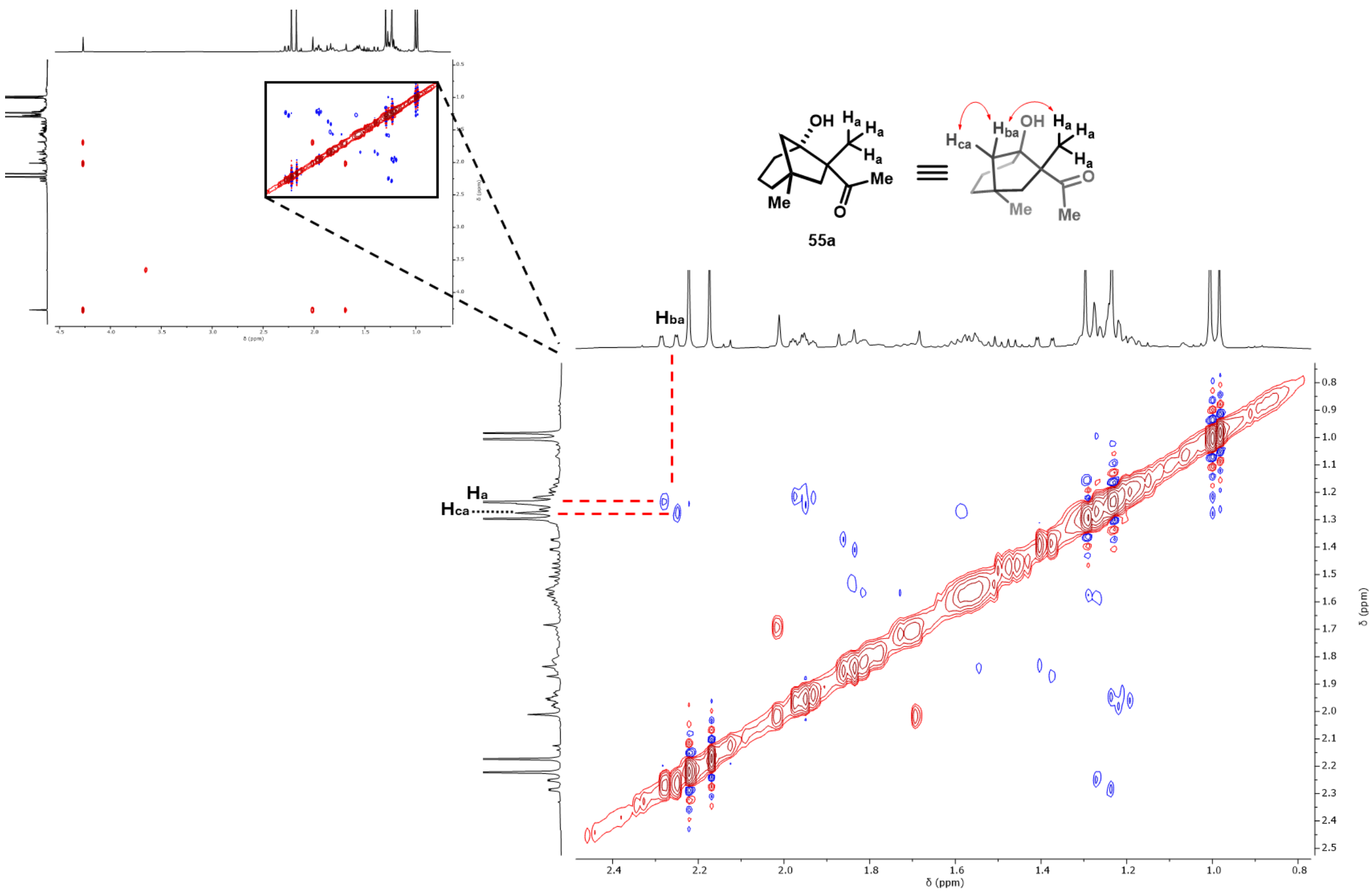

Figure S26. **NOESY NMR** (^{1}H 500 MHz, $CDCl_3$) of a mixture of compounds **55a** and **55b**, showing NOE correlations in the major diastereomer **55a** between the methyl group of interest (H_a) and one of bridgehead protons (H_{ba}). An additional correlation between the geminal bridgehead protons (H_{ba} and H_{ca}) is also observed. The H_a–H_{ba}. correlation is expected only for this relative configuration.

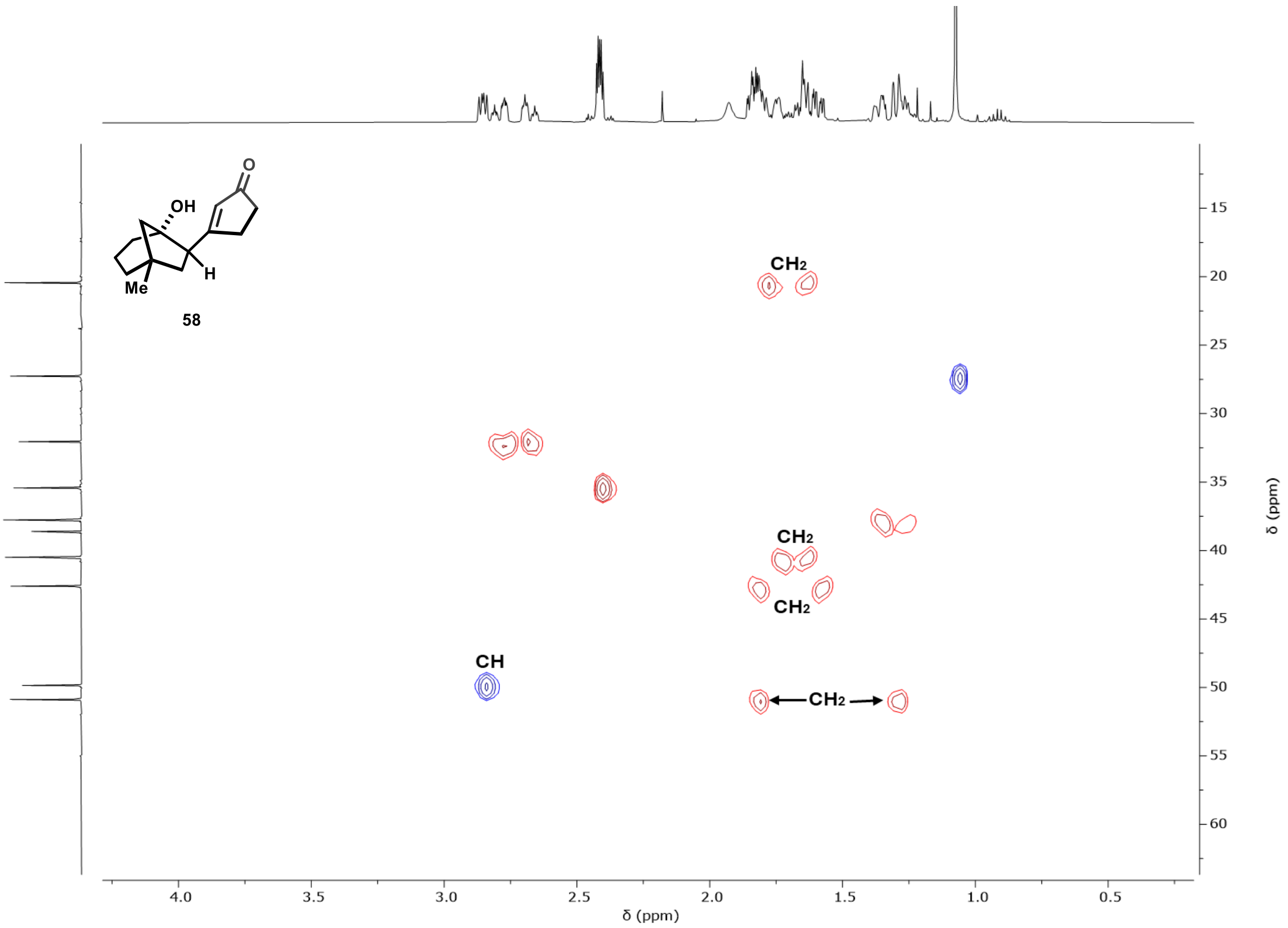

Figure S27. Edited HSQC NMR (500 MHz, $CDCl_3$) of compound **58.**

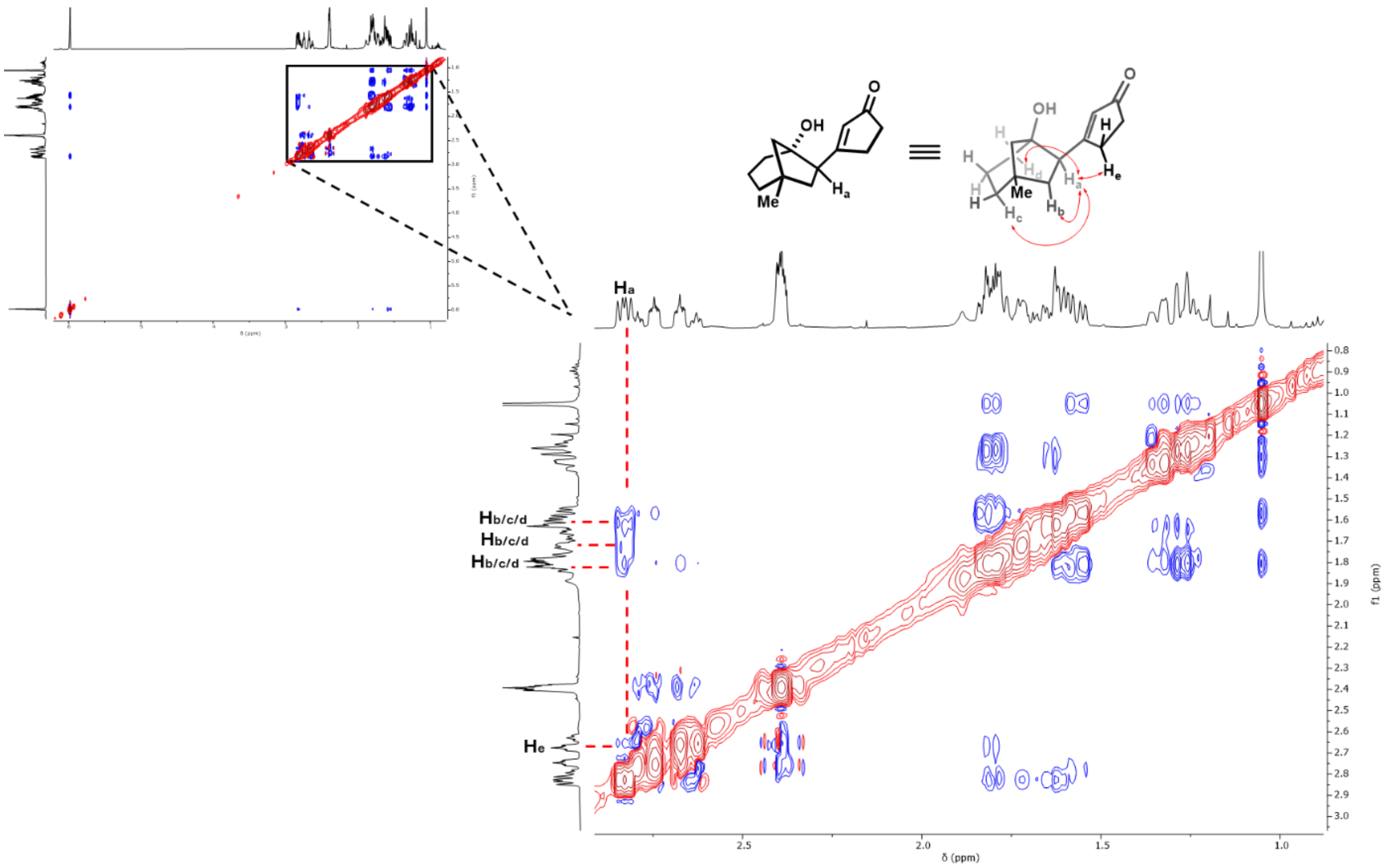

Figure S28. NOESY NMR (^{1}H 400 MHz, $CDCl_3$) of compound **58**, showing NOE correlations between the CH proton of interest (H_a) and one methylene proton (H_e) on the pentenone ring, as well as three methylene protons ($H_{b\text{-}d}$) on the seven-membered ring, which are expected only for this relative configuration.

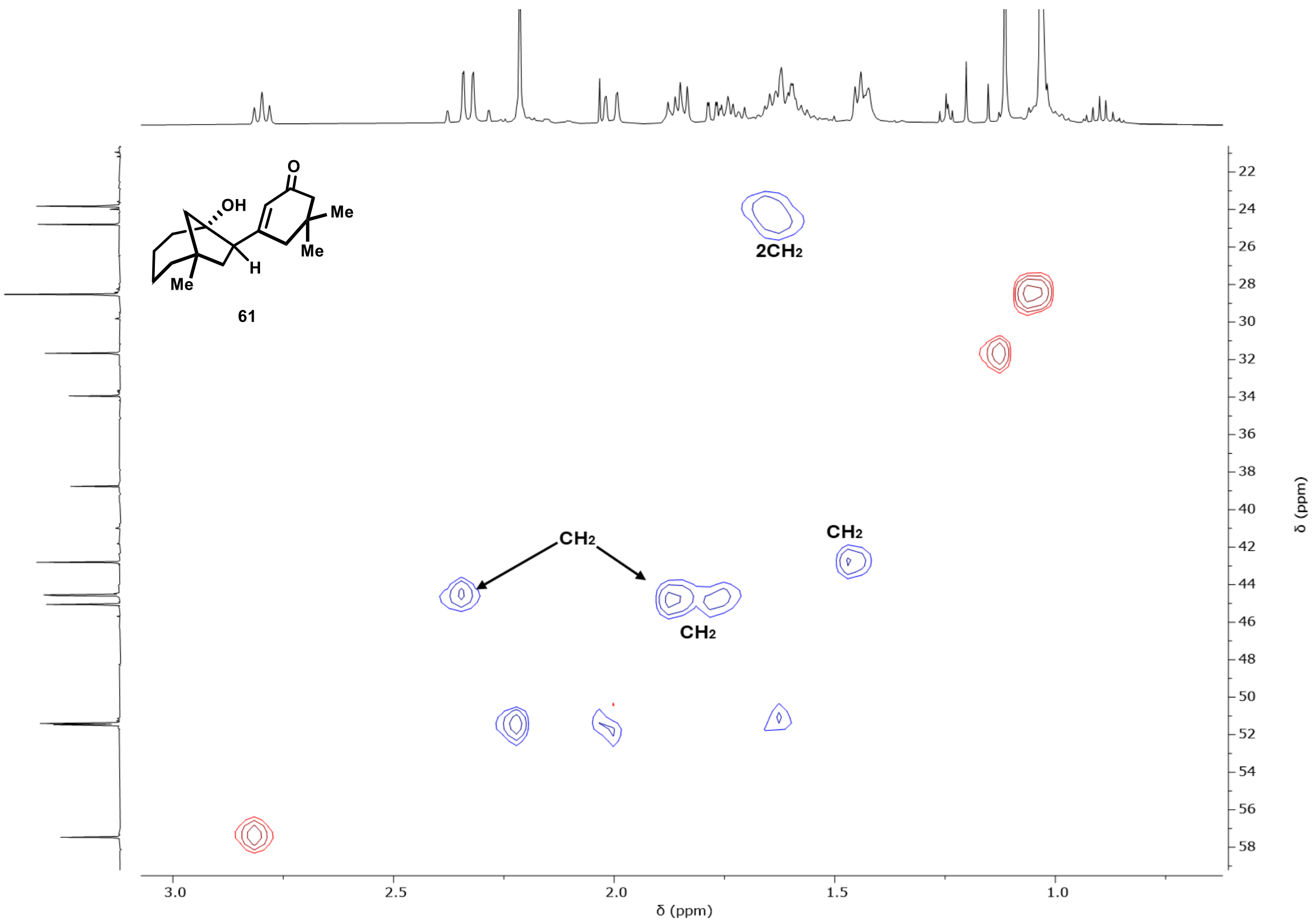

Figure S29. Edited HSQC NMR (500 MHz, $CDCl_3$) of compound **61.**

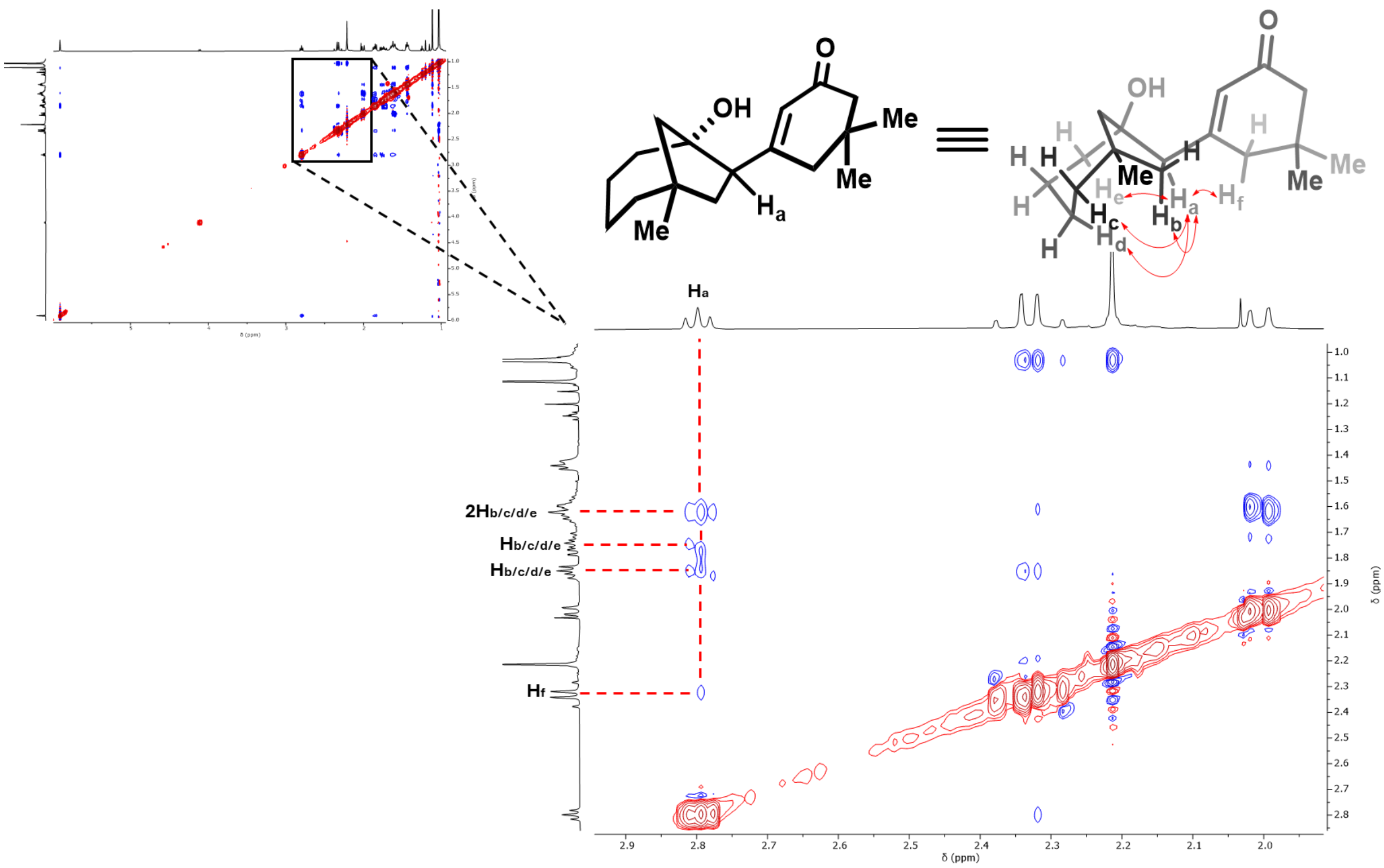

Figure S30. NOESY NMR (^{1}H 400 MHz, $CDCl_3$) of compound **61**, showing NOE correlations between the CH proton of interest (H_a) and one methylene proton (H_f) on the hexenone ring, as well as four methylene protons (H_{b-e}) on the eight-membered ring, which are expected only for this relative configuration.

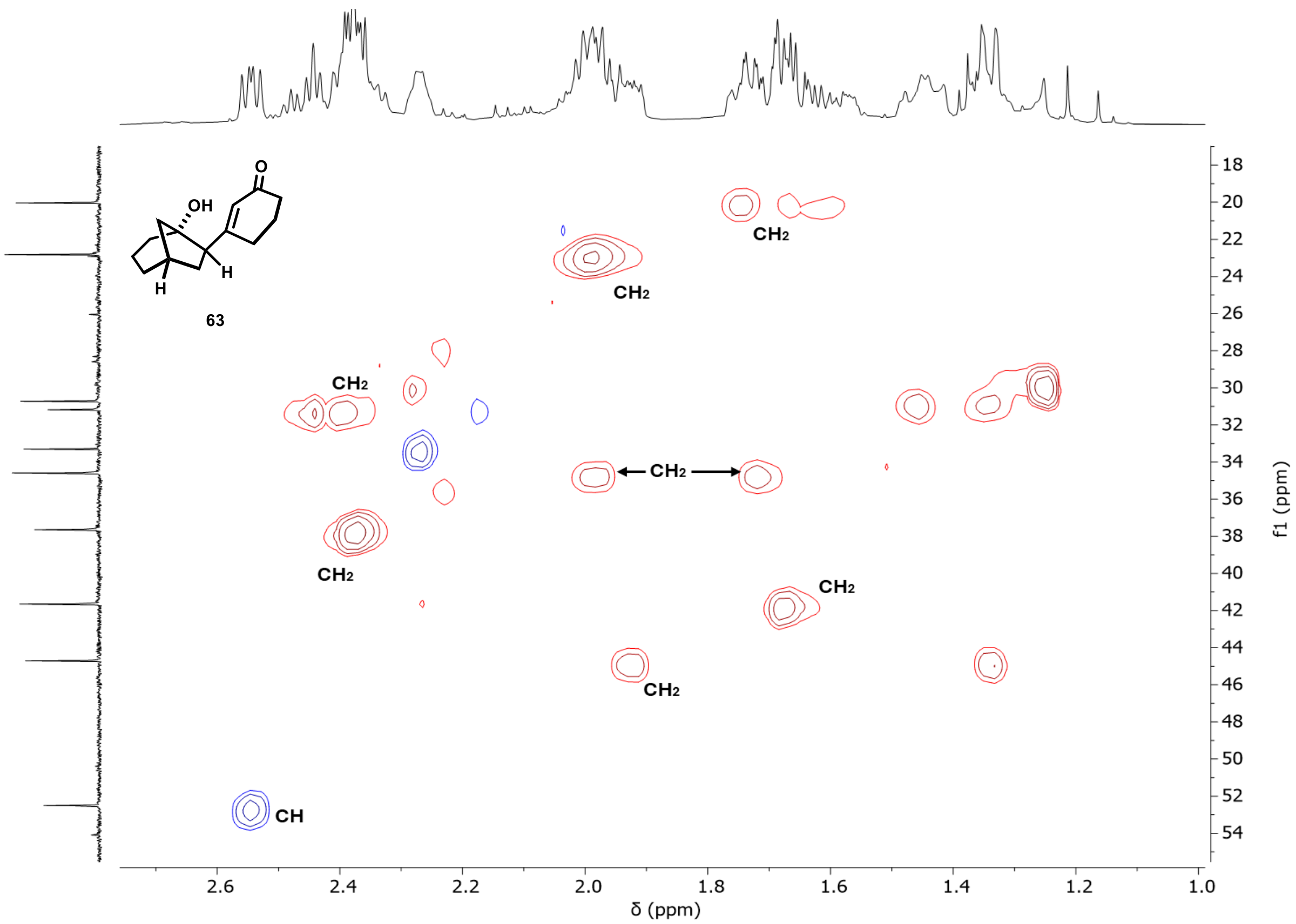

Figure S31. Edited HSQC NMR (500 MHz, $CDCl_3$) of compound **63.**

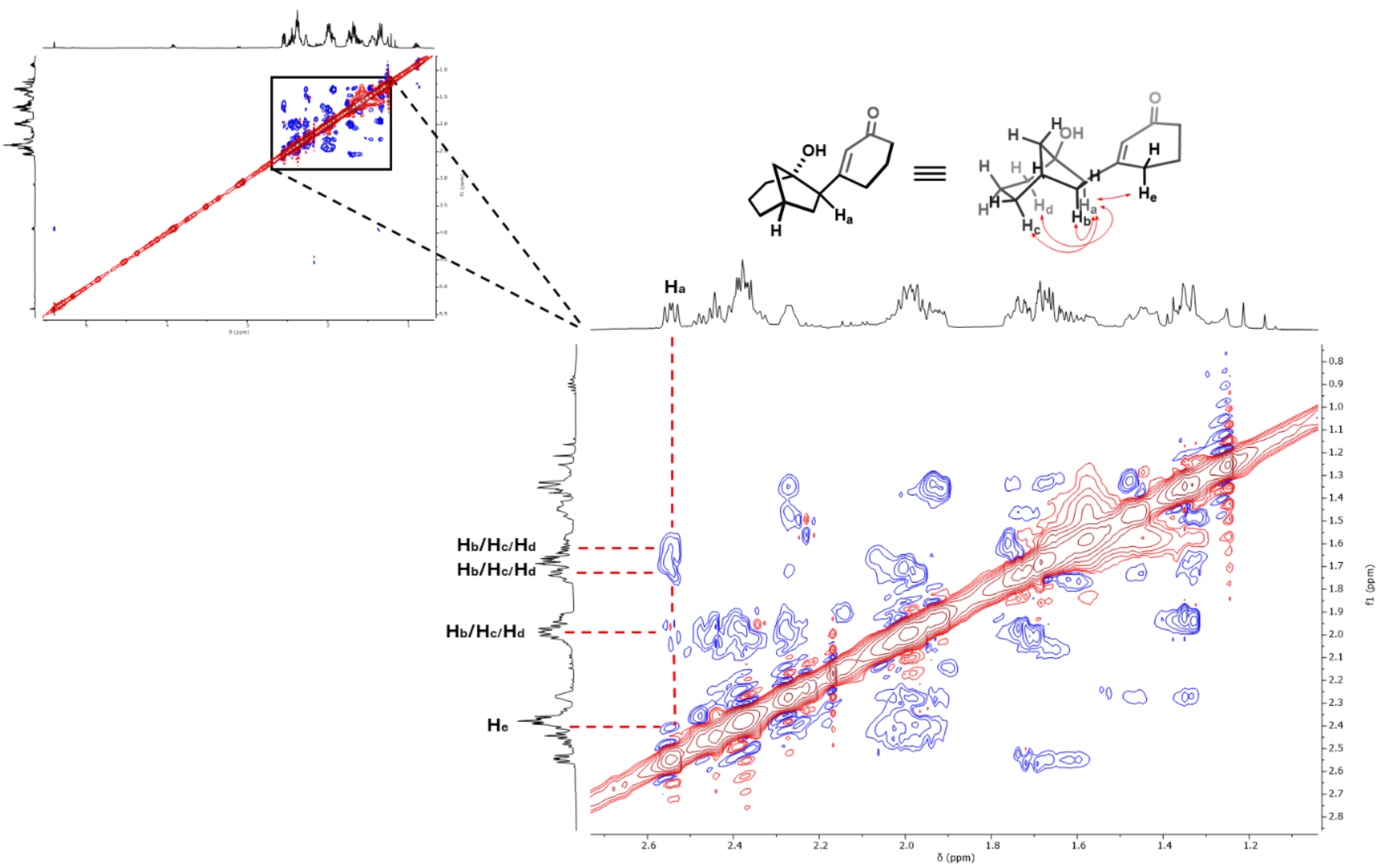

Figure S32. NOESY NMR (^{1}H 400 MHz, $CDCl_3$) of compound **63**, showing NOE correlations between the CH proton of interest (H_a) and one methylene proton (H_e) on the pentanone ring, as well as three methylene protons (H_{b-d}) on the seven-membered ring, which are expected only for this relative configuration.

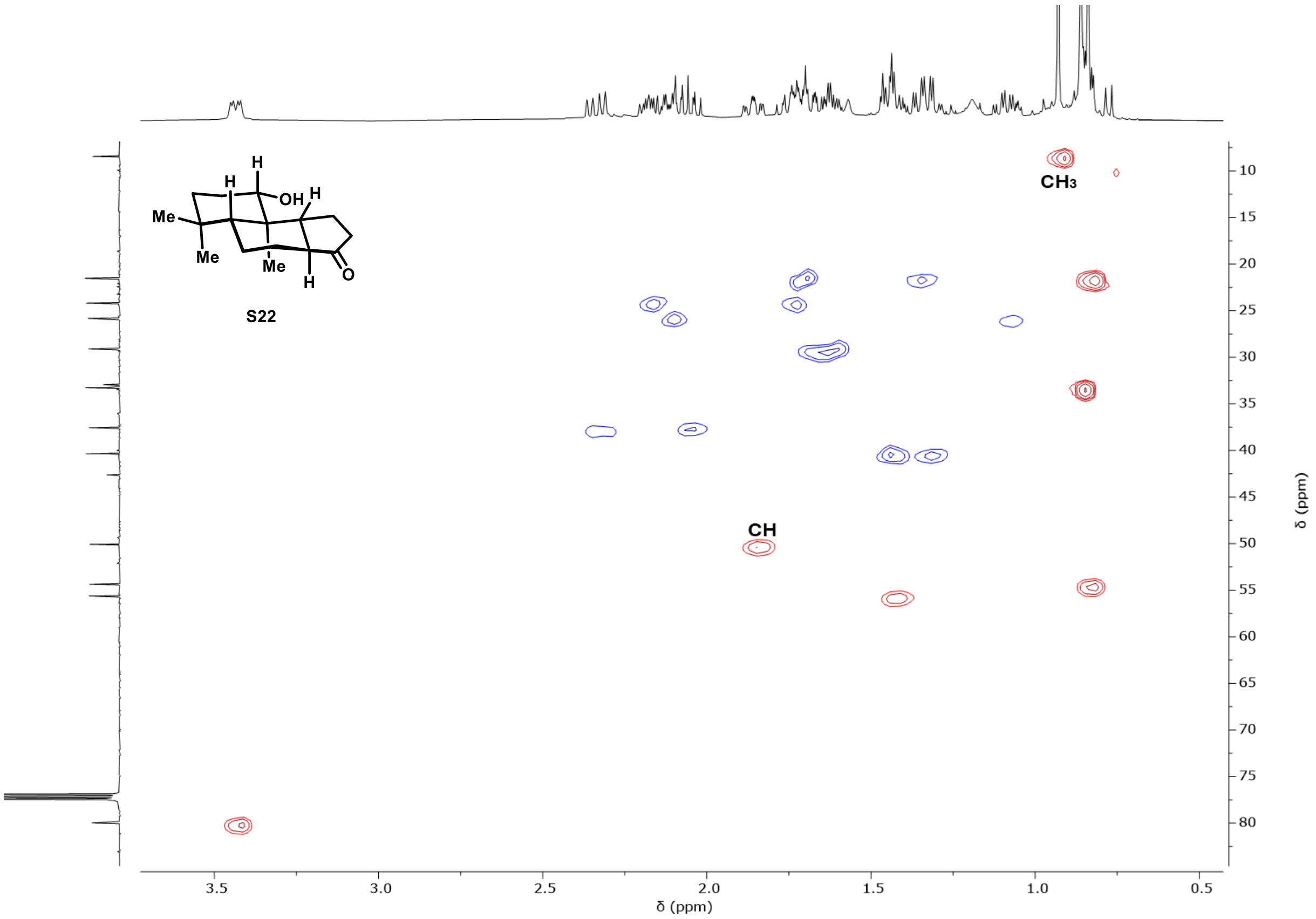

Figure S33. Edited HSQC NMR (500 MHz, $CDCl_3$) of compound **S22.**

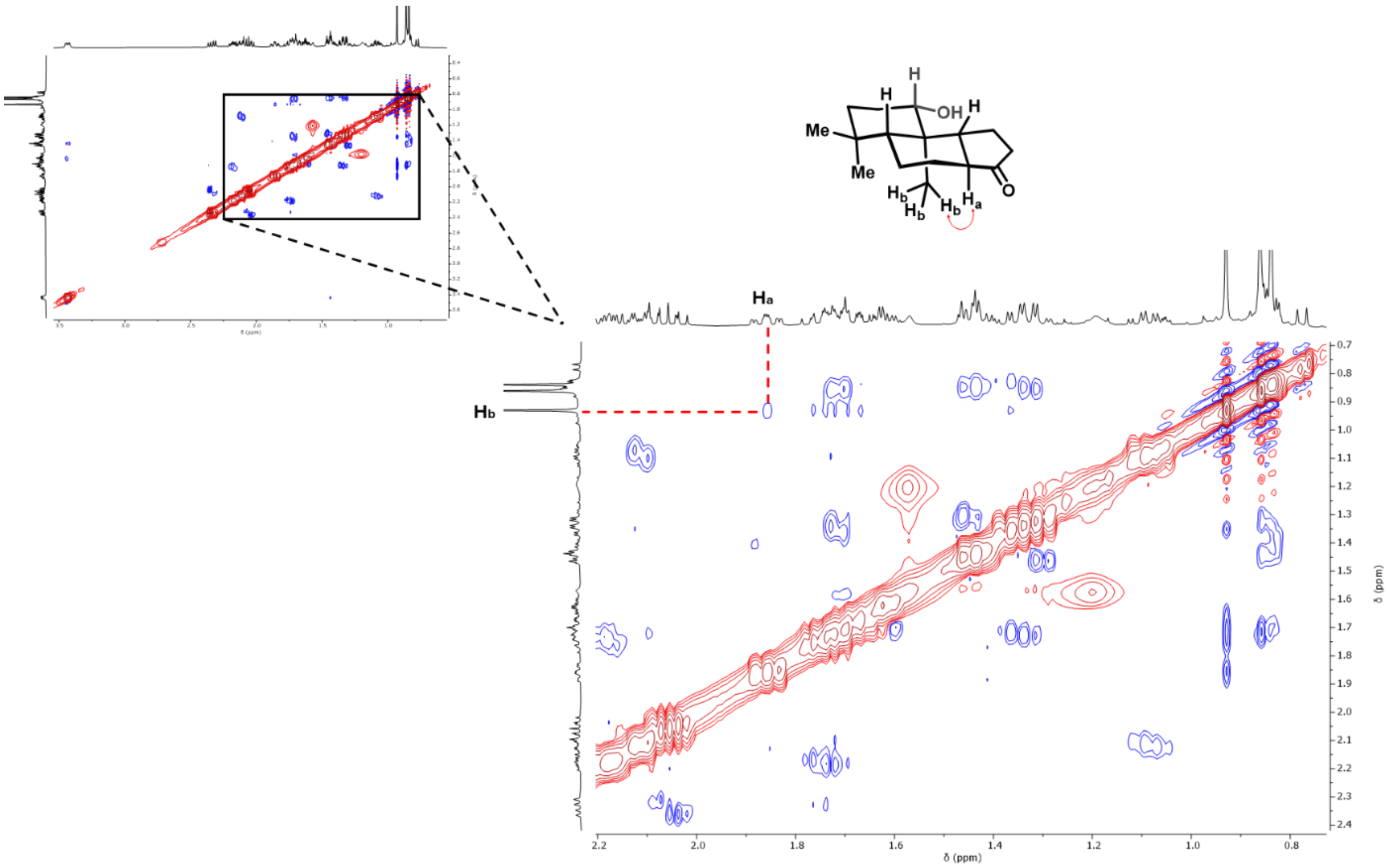

Figure S34. NOESY NMR (^{1}H 500 MHz, $CDCl_3$) of compound **S22**, showing NOE correlations between the CH proton of interest (H_a) and the methyl group (H_b), which are expected only for this relative configuration.

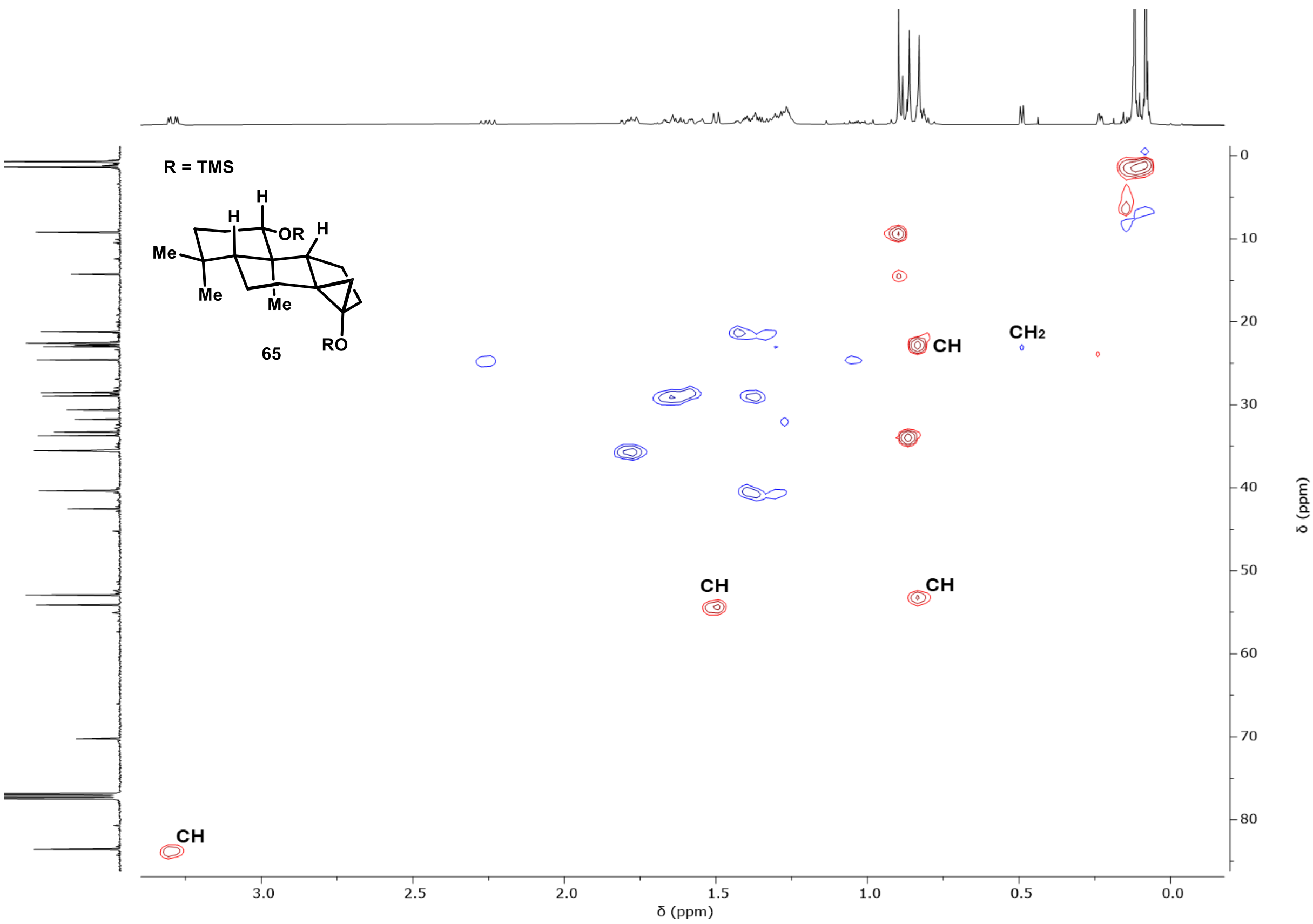

Figure S35. Edited HSQC NMR (500 MHz, $CDCl_3$) of compound **65.**

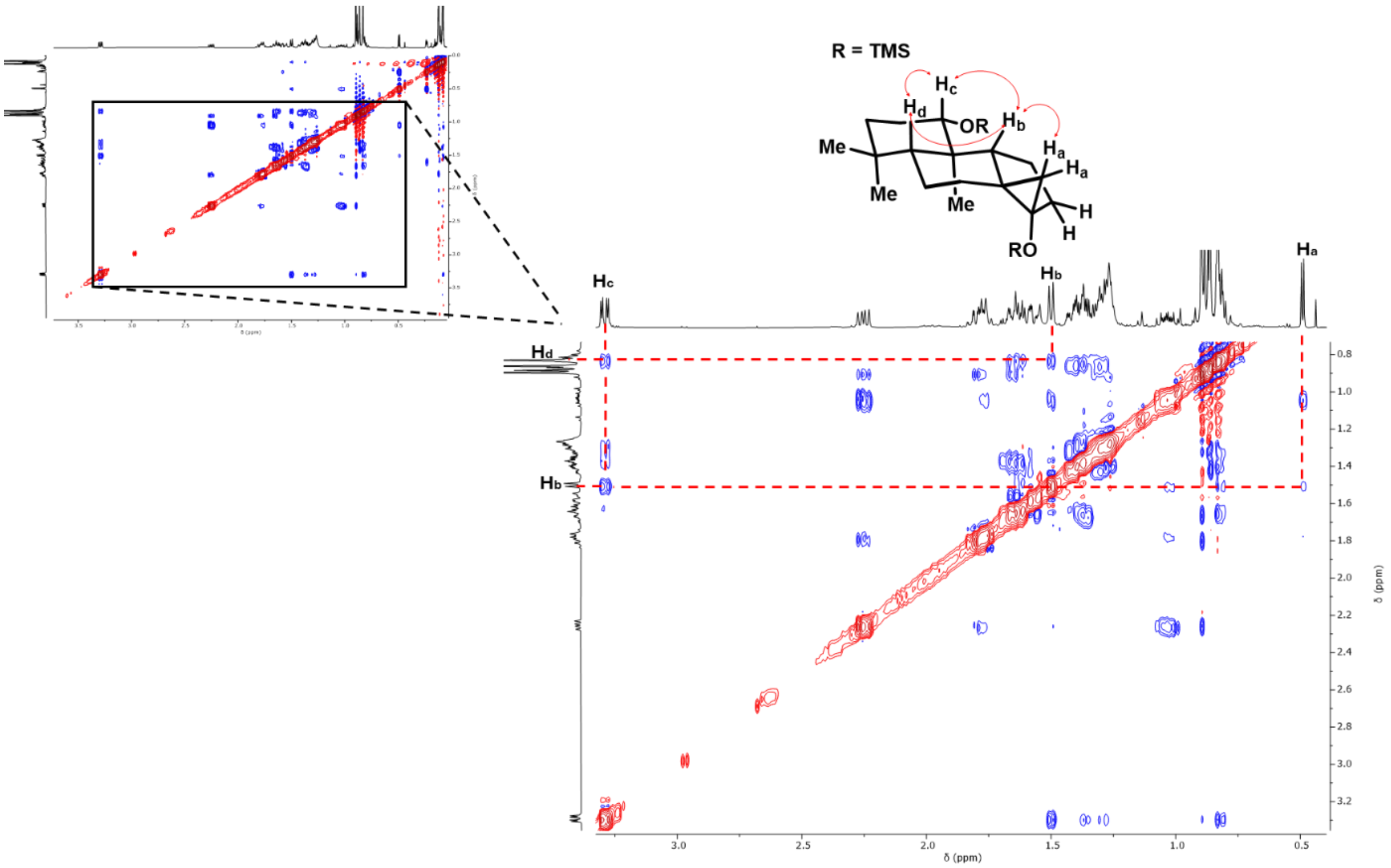

Figure S36. NOESY NMR (^{1}H 500 MHz, $CDCl_3$) of compound **65**, showing NOE correlations between the CH proton of interest (H_a) and one CH proton (H_b) on the pentane ring. Mutual NOE cross-correlations between the three axial protons (H_b, H_c and H_d) are also shown. This is only expected for this relative configuration.

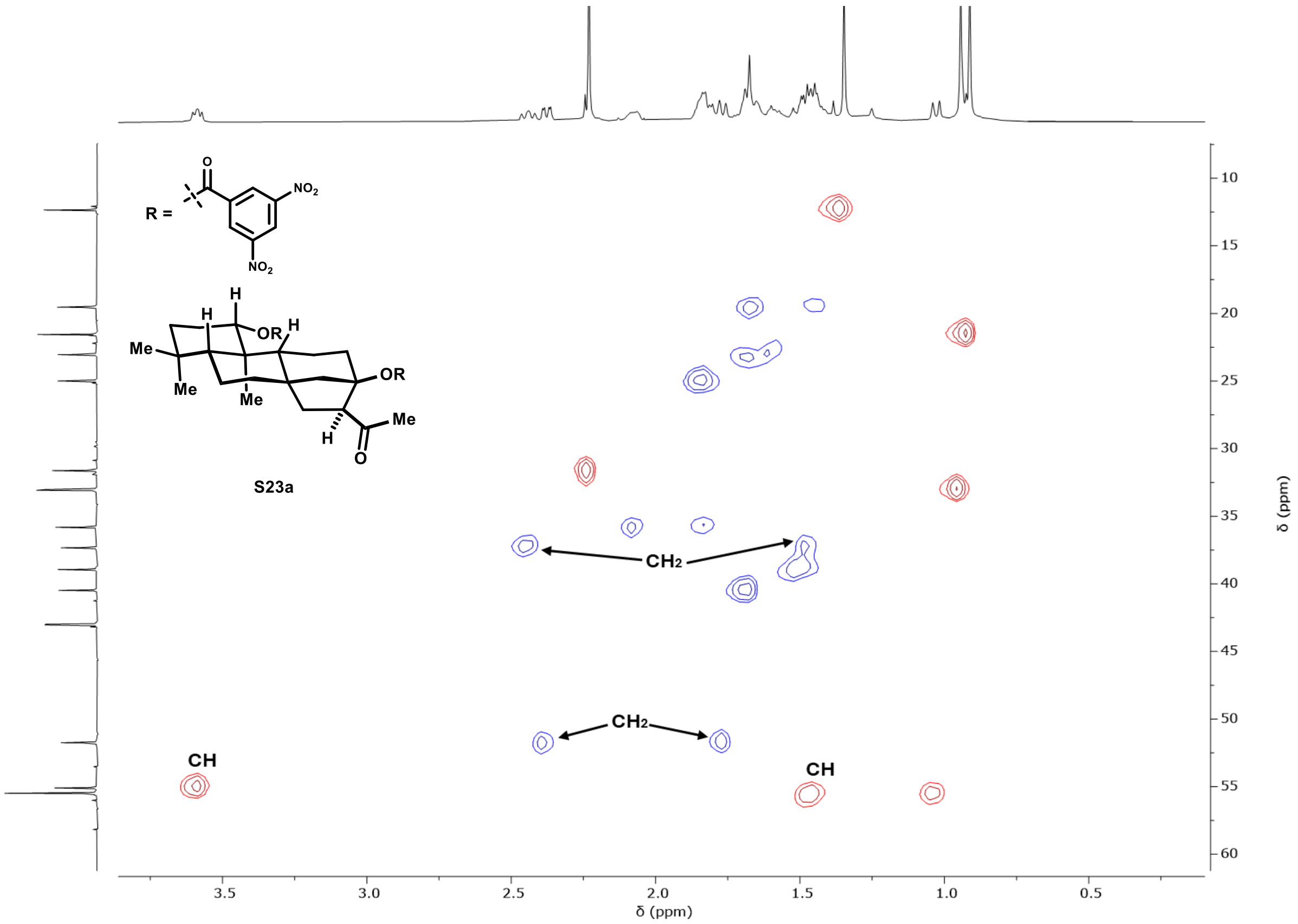

Figure S37. Edited HSQC NMR (500 MHz, $CDCl_3$) of compound **S23a.** *R = 3,5-dinitrobenzoyl group.*

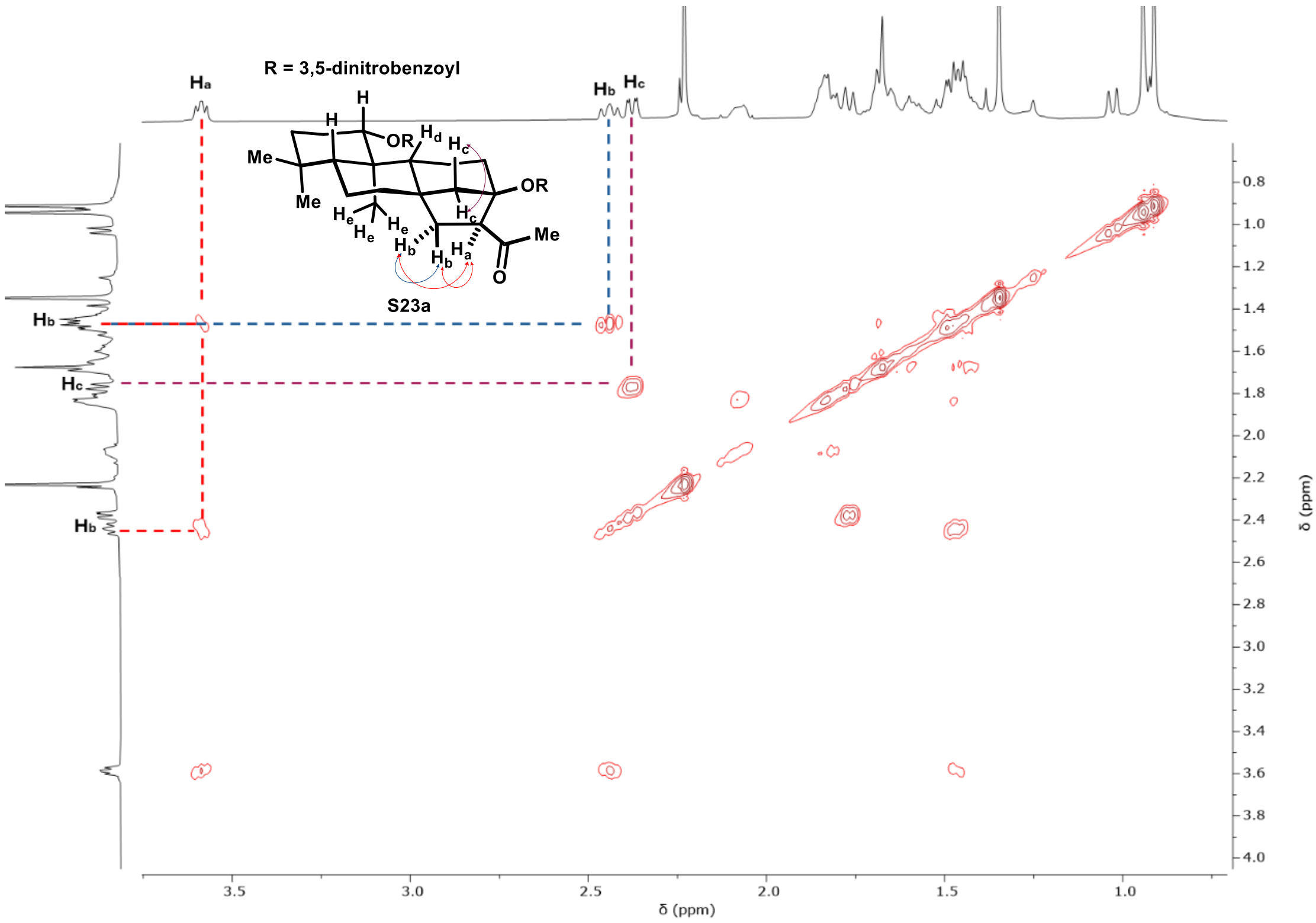

Figure S38. COSY NMR (^{1}H 500 MHz, $CDCl_3$) of compound **S23a**, showing COSY correlations between the CH proton of interest (H_a) and the neighbouring methylene protons H_b. The geminal COSY correlation within this methylene group is also shown, together with the geminal correlation between the methylene protons H_c.

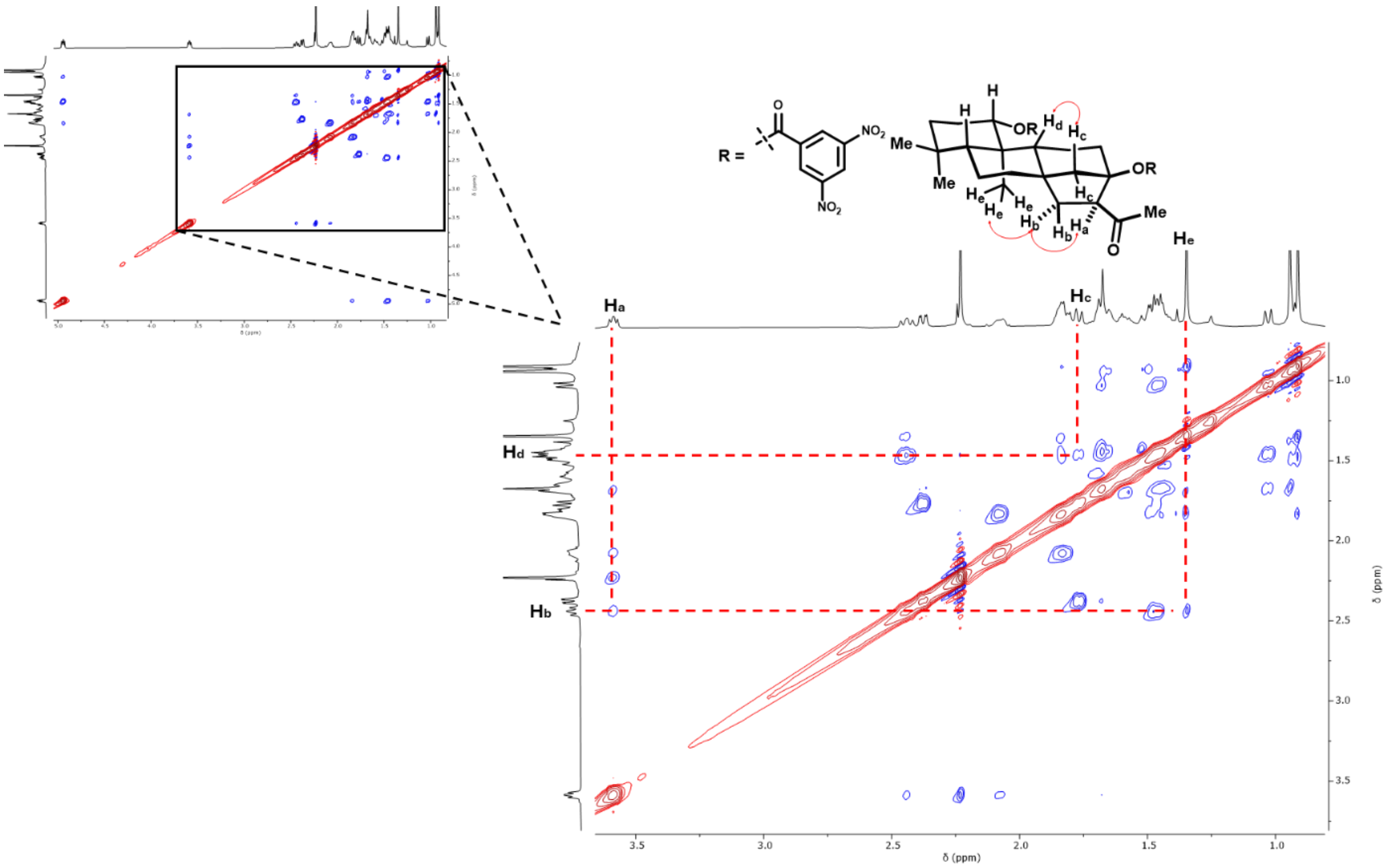

Figure S39. NOESY NMR (^{1}H 500 MHz, $CDCl_3$) of compound **S23a**, showing NOE correlations between the CH proton of interest (H_a) and the neighbouring methylene proton H_b. The NOE correlation between the methylene proton H_b and the methyl group (H_e) is also shown, together with the correlation between the methylene proton H_c and CH proton H_d.

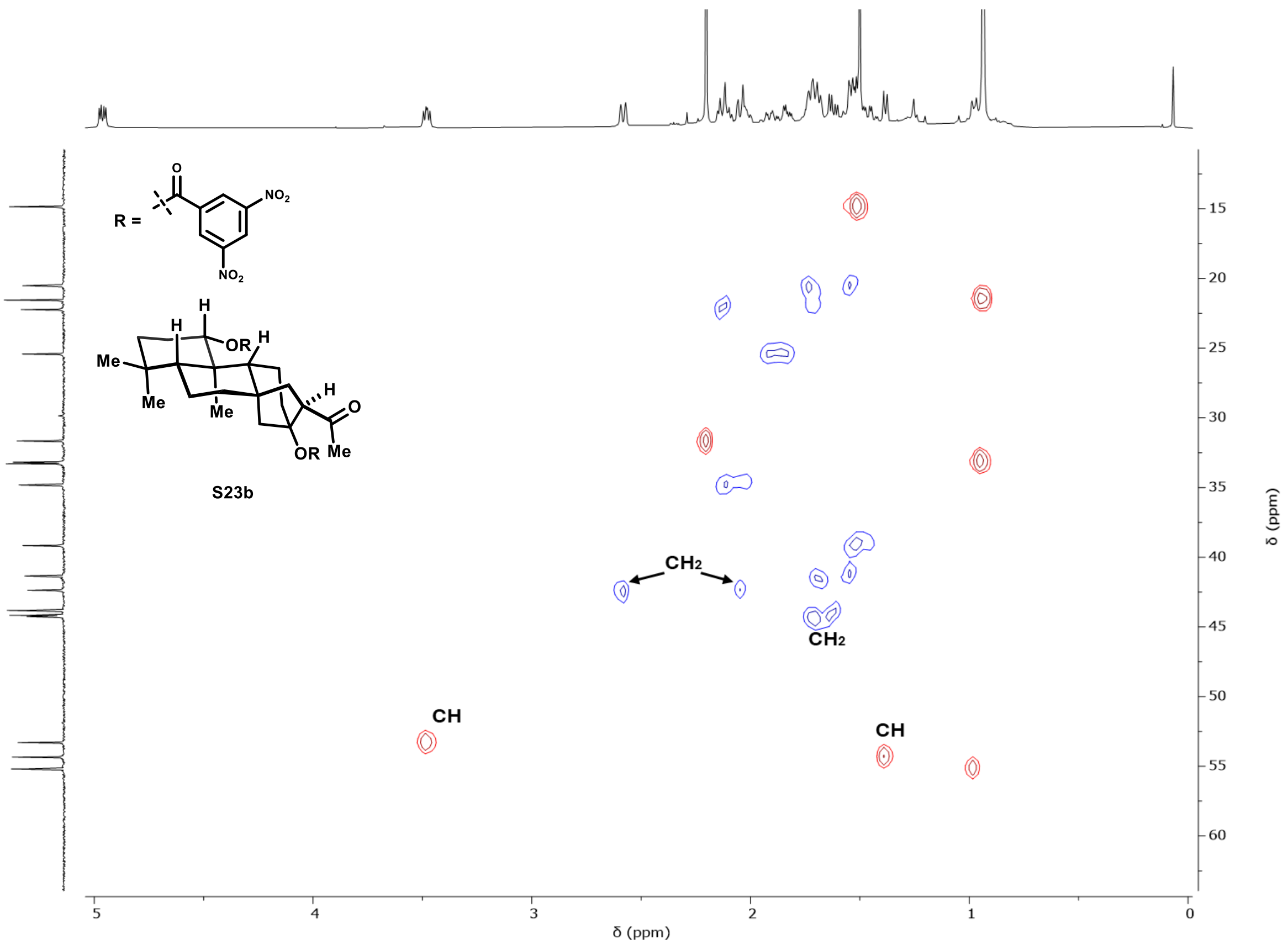

Figure S40. Edited HSQC NMR (500 MHz, $CDCl_3$) of compound **S23b.**

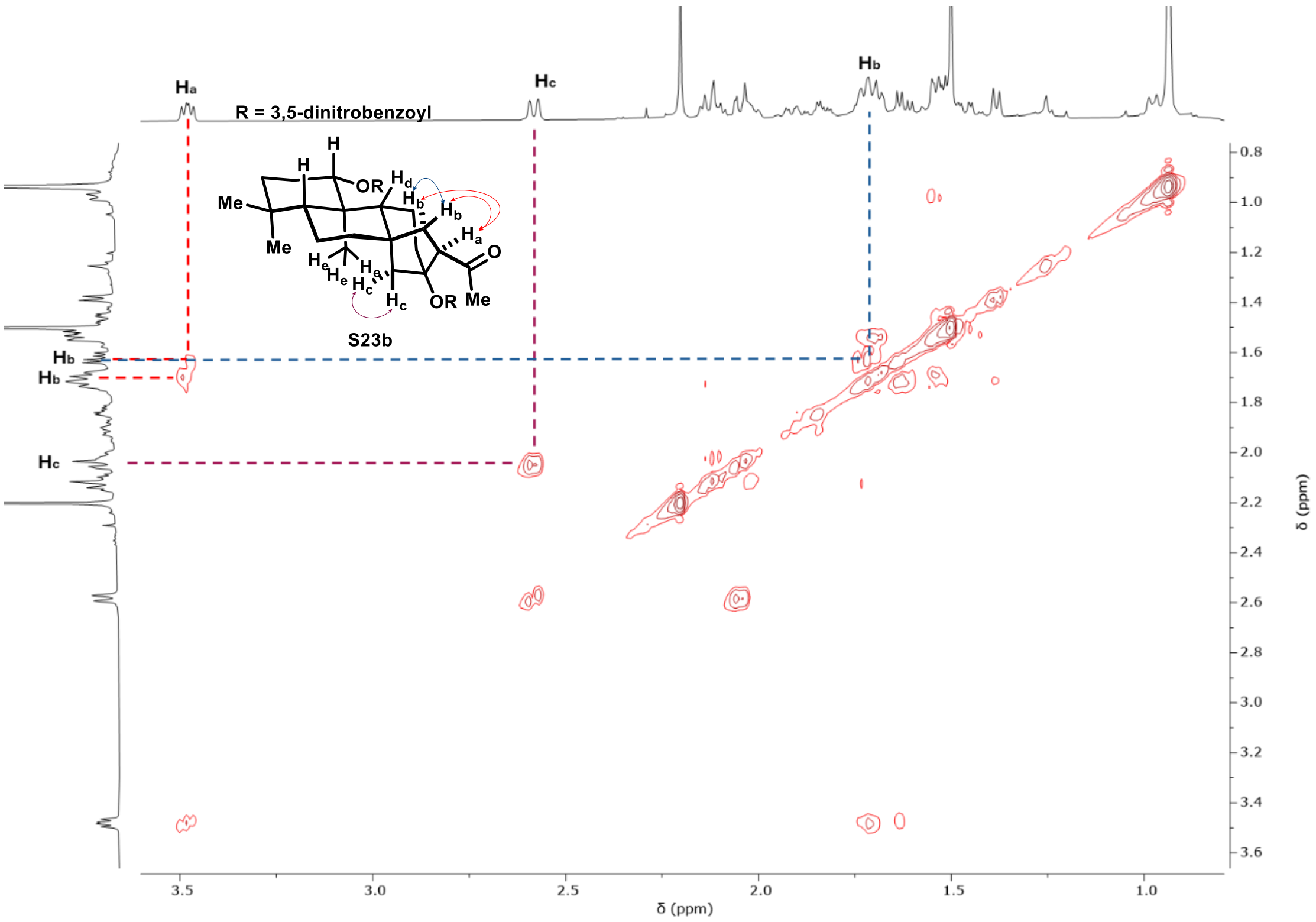

Figure S41. COSY NMR (^{1}H 500 MHz, $CDCl_3$) of compound **S23b**, showing COSY correlations between the CH proton of interest (H_a) and the neighbouring methylene protons H_b. The geminal COSY correlation within this methylene group is also shown, together with the geminal correlation between the methylene protons H_c.

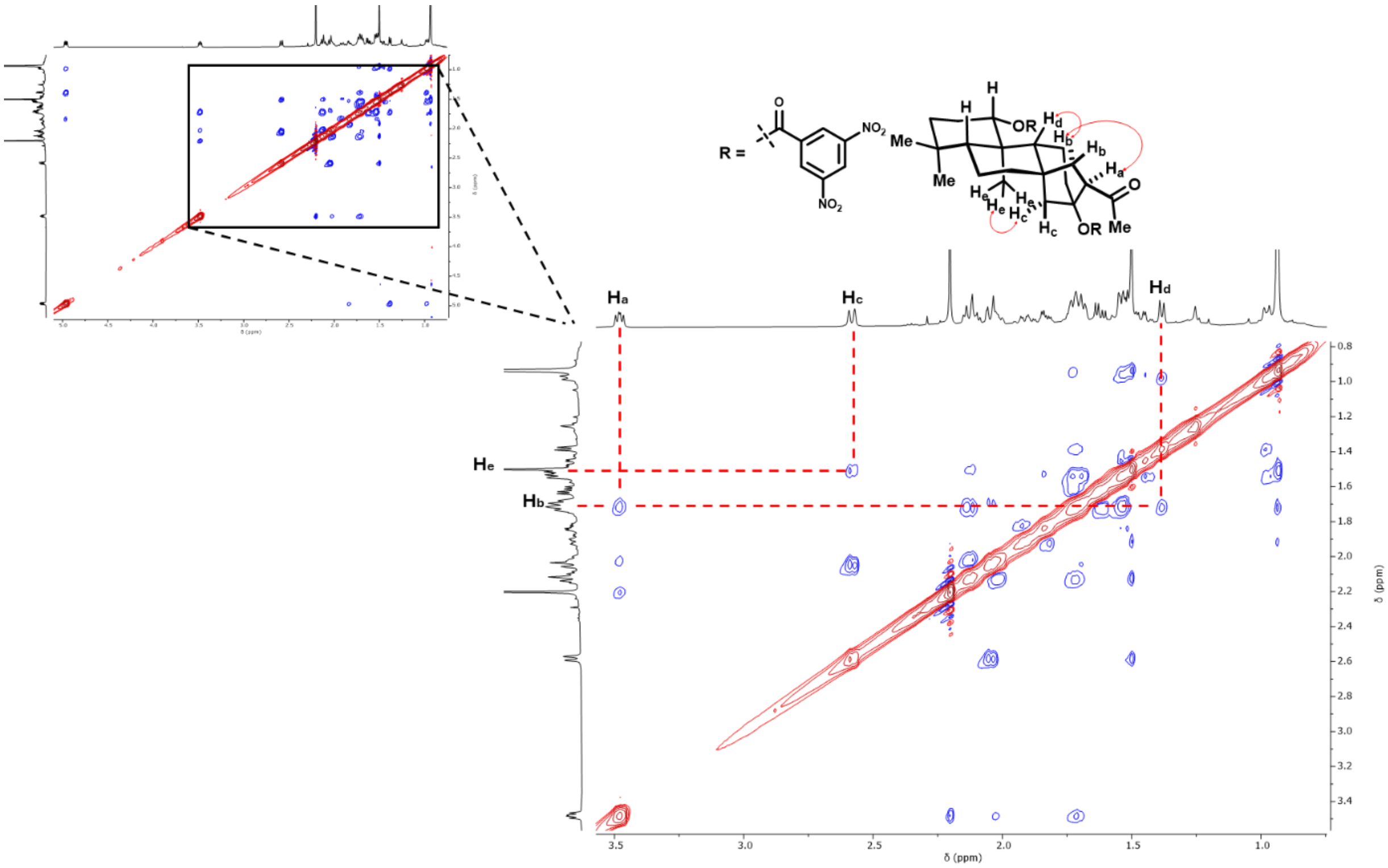

Figure S42. NOESY NMR (^{1}H 500 MHz, $CDCl_3$) of compound **S23b**, showing NOE correlations between the CH proton of interest (H_a) and the neighbouring methylene proton H_b. The NOE correlation between the methylene proton (H_b) and CH proton H_d is also shown, together with the correlation between the methylene proton H_c and the methyl group (H_e).

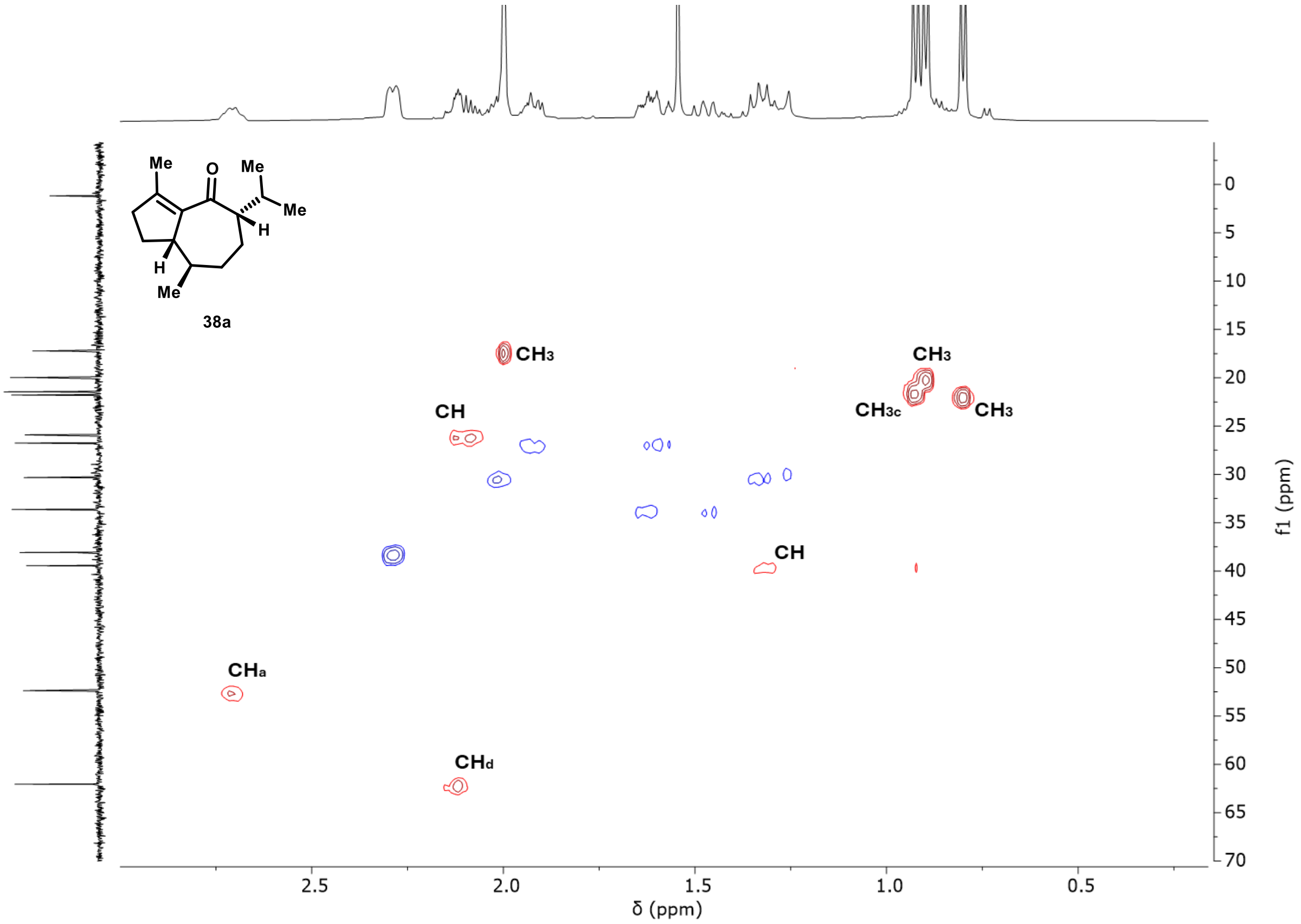

Figure S43. Edited HSQC NMR (500 MHz, $CDCl_3$) of compound **38a.**

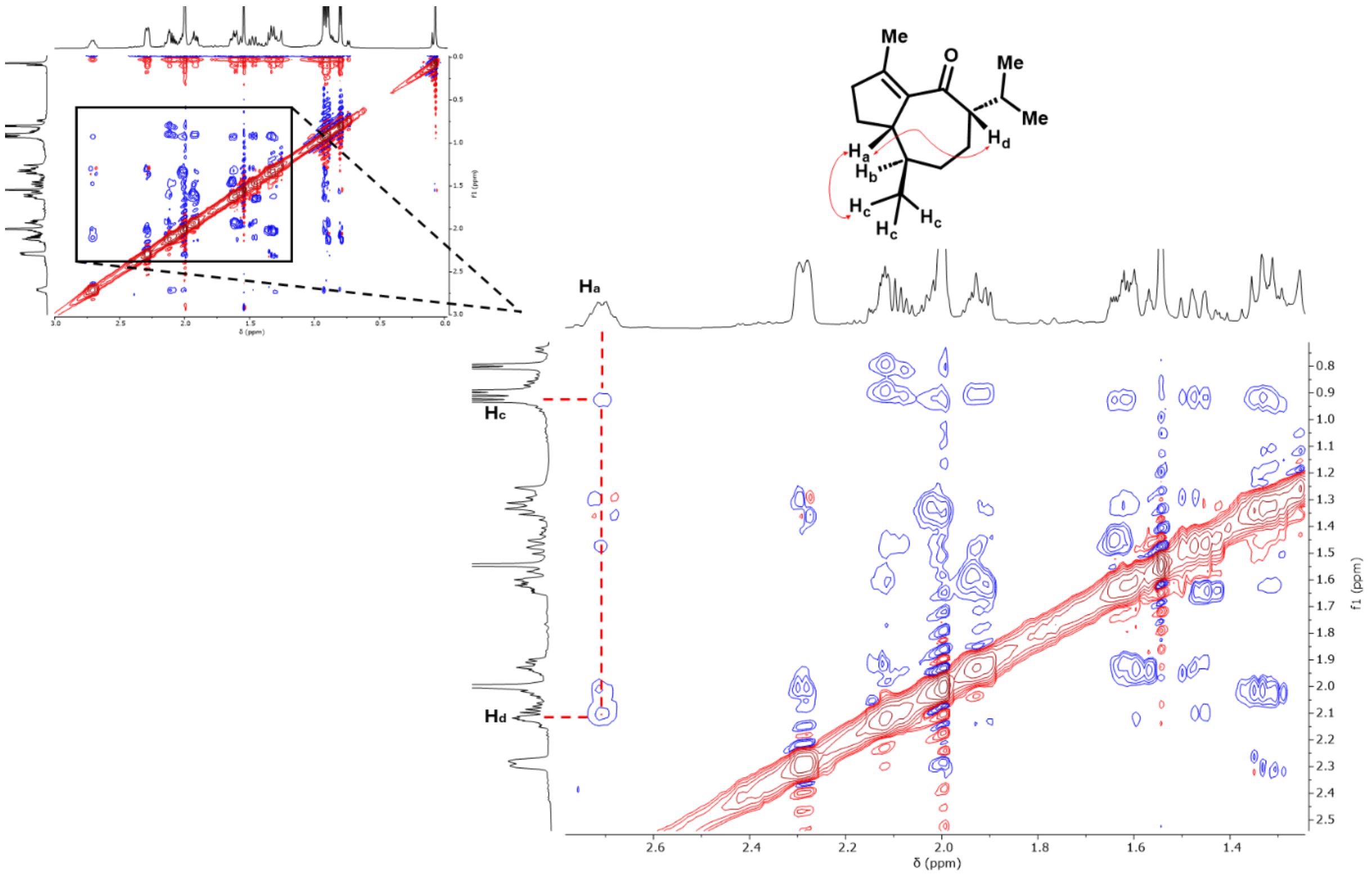

Figure S44. NOESY NMR (^{1}H 500 MHz, $CDCl_3$) of compound **38a**, showing NOE correlations between the CH proton of interest (H_a) and the methyl group (H_c), as well as the α-proton (H_d), which are expected only for this relative configuration.
